# CSF-resident CD4^+^ T-cells display a distinct gene expression profile with relevance to immune surveillance and multiple sclerosis

**DOI:** 10.1093/braincomms/fcab155

**Published:** 2021-07-13

**Authors:** James Hrastelj, Robert Andrews, Samantha Loveless, Joanne Morgan, Stefan Mark Bishop, Nicholas J Bray, Nigel M Williams, Neil P Robertson

**Affiliations:** 1 Division of Psychological Medicine and Clinical Neuroscience, Cardiff University, Cardiff CF14 4XW, UK; 2 School of Medicine, Cardiff University, Cardiff CF14 4XW, UK; 3 European Cancer Stem Cell Research Institute, Cardiff University, Cardiff CF24 4HQ, UK

**Keywords:** multiple sclerosis, gene expression, CSF, CD4+, T-cells, immune surveillance

## Abstract

The CNS has traditionally been considered an immune privileged site, but is now understood to have a system of immune surveillance, predominantly involving CD4^+^ T-cells. Identifying functional differences between CNS and blood CD4^+^ T-cells, therefore, have relevance to CNS immune surveillance as well as to neurological conditions, such as multiple sclerosis, in which CD4^+^ T-cells play a central role. Here, CD4^+^ T-cells were purified from CSF and blood from 21 patients with newly diagnosed treatment-naïve multiple sclerosis and 20 individuals with non-inflammatory disorders using fluorescence-activated cell sorting, and their transcriptomes were profiled by RNA sequencing. Paired comparisons between CD4^+^ T-cells from CSF and blood identified 5156 differentially expressed genes in controls and 4263 differentially expressed in multiple sclerosis patients at false discovery rate <5%. Differential expression analysis of CD4^+^ T-cells collected from the CSF highlighted genes involved in migration, activation, cholesterol biosynthesis and signalling, including those with known relevance to multiple sclerosis pathogenesis and treatment. Expression of markers of CD4^+^ T-cell subtypes suggested an increased proportion of Th1 and Th17 cells in CSF. Gene ontology terms significant only in multiple sclerosis were predominantly those involved in cellular proliferation. A two-way comparison of CSF versus blood CD4^+^ T-cells in multiple sclerosis compared with non-inflammatory disorder controls identified four significant genes at false discovery rate <5% (*CYP51A1*, *LRRD1*, *YES1* and *PASK*), further implicating cholesterol biosynthesis and migration mechanisms. Analysis of CSF CD4^+^ T-cells in an extended cohort of multiple sclerosis cases (total *N* = 41) compared with non-inflammatory disorder controls (total *N* = 38) identified 140 differentially expressed genes at false discovery rate < 5%, many of which have known relevance to multiple sclerosis, including *XBP1*, *BHLHE40*, *CD40LG*, *DPP4* and *ITGB1*. This study provides the largest transcriptomic analysis of purified cell subpopulations in CSF to date and has relevance for the understanding of CNS immune surveillance, as well as multiple sclerosis pathogenesis and treatment discovery.

## Teaser

Hrastelj et al. report the largest transcriptomic comparison of CSF and blood CD4^+^ T-cells to date. The study identifies thousands of significant genes, implicating cellular migration and cholesterol metabolism, as well as Th1, Th17 and effector memory function in immune surveillance of the CNS in multiple sclerosis and controls.

## Graphical Abstract

**Graphical Abstract:**
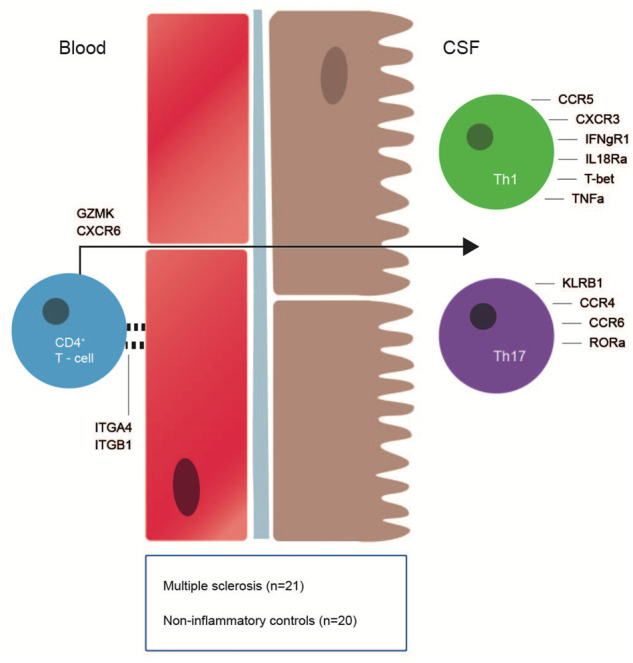

## Introduction

The CNS has, historically, been considered an immune-privileged site devoid of immunological activity in health, but is now known to be a site of highly regulated immune surveillance and homeostasis. Immune cells have been shown to cross the blood–brain barrier at post-capillary venules and the blood–CSF barrier at the choroid plexus and subarachnoid venules.^1^ Cytological analysis has shown that CSF contains stable populations of immune cells in health and that these have a significantly different composition to those in blood.^2^ CD4^+^ T-cells are the predominant cell type in CSF, accounting for ∼70% of cells and the majority of these (∼90%) are antigen-experienced memory CD4^+^ T-cells.^3^^,^^4^ It has been proposed that the constellation of adhesion molecules expressed by the CNS endothelium (e.g. selectins and addressins) and their interactions with adhesion molecules expressed by T-cells (e.g. integrins) could be responsible for ‘homing’ of CNS antigen-specific CD4^+^ T-cells to the CNS.^1^ Such mechanisms are likely to be relevant to the pathogenesis of multiple sclerosis, as multiple lines of evidence, including genetic, immunological and animal model data, implicate CD4^+^ T-cells in the condition.^5^ Furthermore, one of the most potent disease-modifying treatments for multiple sclerosis, natalizumab, is a monoclonal antibody against integrin-α4, an adhesion molecule that facilitates CD4^+^ T-cell extravasation into the CNS. Thus, identifying differences between CD4^+^ T-cells in the CNS and blood could provide novel treatment targets for the disorder.

Characterization of CD4^+^ T-cells within the CNS in health and disease is challenged by the difficulty in obtaining sizeable cohorts, isolating sufficient numbers of cells and performing timely functional analysis. Genome-wide gene expression (or ‘transcriptomic’) analyses can provide valuable insights into cell state and function, but while several such analyses have been performed on CD4^+^ T-cells from peripheral blood,^6–11^ few studies have investigated gene expression in CSF CD4^+^ T-cells. To date, CSF analyses have either been restricted to a limited number of assayed genes, used unsorted CSF cells or used small patient cohorts.^12–14^ Although single-cell gene expression analyses have great potential for characterizing CSF immune cells, costs and technical challenges have so far restricted sample sizes to no more than 12 participants.^15–17^ Large single-cell studies would allow high-resolution analysis of the roles of functional subtypes of CSF cells; however, the extent of transcriptome coverage currently lags behind that provided by standard ‘bulk’ RNA-sequencing.^18^ An alternative, more powerful strategy for detecting differentially expressed genes is to perform transcriptomic analyses on purified CSF CD4^+^ T-cell populations from large cohorts. In this study, we apply whole transcriptome sequencing to fluorescence-activated cell sorted CD4^+^ T-cells from the CSF and blood of large cohorts of multiple sclerosis patients and non-inflammatory disorder (NID) controls, identifying thousands of genes that are differentially expressed between CD4^+^ cells from CSF and blood, as well as genes that are differentially expressed in CSF CD4^+^ cells in multiple sclerosis.

## Materials and methods

### Patient recruitment and sample acquisition

Potential participants were identified from patients referred for diagnostic lumbar punctures as part of their routine clinical care to the Neurology Ambulatory Unit at the University Hospital of Wales, Cardiff. Participants were recruited to the South Wales Multiple Sclerosis Epidemiology Project (South East Wales REC panel C, ref 05/WSE03/111) or Welsh Neuroscience Research Tissue Bank (WNRTB; Wales REC panel 3, ref 19/WA/0058) project. Written consent was obtained from each participant and lumbar puncture and venepuncture were performed according to standard clinical practice. Twelve millilitres of blood and 10 ml CSF was collected from each participant concomitantly and transferred on ice to the laboratory.

RNA sequencing (RNA-seq) data were available for a final cohort of 79 participants: 41 with multiple sclerosis (21 of which had both CSF and blood data) and 38 with NID (20 of which had both CSF and blood data). The diagnoses of the NID group included idiopathic intracranial hypertension (14), functional disorder (5), cerebral small vessel disease (5), migraine (3) and a range of other non-inflammatory disorders (Supplementary Table 1). The demographic characteristics of the included patients are shown in Supplementary Table 2. The NID controls were more frequently female than patients with multiple sclerosis (85.0% and 65.0%, respectively; *P* = 0.05), but were well matched for age at sampling and ethnicity. Diagnosis of multiple sclerosis was made according to the McDonald criteria appropriate at the time of diagnosis. None of the multiple sclerosis patients had experienced clinical relapse in the four weeks preceding sampling and no patient had been treated with immunomodulatory drugs. Disease-specific characteristics of the patients with multiple sclerosis are shown in Supplementary Table 3.

### Fluorescence-activated cell sorting

Fluorescence-activated cell sorting (FACS) uses fluorescently labelled antibodies against specific cell surface molecules to count and sort specific cell types into separate tubes. Labelled antibodies were chosen to isolate pure populations of live CD19^−^CD14^−^CD3^+^CD4^+^ T-cells (Supplementary Table 4 and Supplementary Figs. 1 and 2).

Within one hour of sample collection, CSF was centrifuged at 200 × *g* for 10 min at 4°C. At room temperature, 1 μl LIVE/DEAD^®^ ViViD aqua stain (Invitrogen/ThermoFisher, Paisley, UK; pre-diluted) was added to 39 μl phosphate-buffered saline (PBS) and the mixture was vortexed and centrifuged down. CSF supernatant was transferred into a 50 ml tube and placed on ice. The cell pellet was resuspended in 50 µl of PBS and transferred into a FACS tube (BD Biosciences, Oxford, UK). At room temperature, 8 μl of diluted LIVE/DEAD^®^ ViViD aqua stain mixture was added to the cell suspension and incubated at room temperature for 10 min, shielded from the light. Each labelled antibody was then added to the CSF, which was incubated at 4°C for 20 min.

Compensation tubes were prepared containing antibody-binding and inert polystyrene beads (BD Biosciences, Oxford, UK). Fifty microlitres of bead suspension was added to five FACS tubes. Each fluorescently labelled antibody was added to a separate bead suspension tube and one bead suspension tube was left unstained. The emission range of the three labels used to exclude cells overlap almost completely (LIVE/DEAD^®^, anti-CD14 and anti-CD19), so only one compensation tube was necessary for all of these. The bead suspension and labelled antibody mixtures were incubated at room temperature for 10 min, shielded from the light. Once the CSF incubation was complete, 2 ml PBS was added and the mixture was centrifuged at 200 × *g* for 3 min to wash the cells. The supernatant was removed and the cell pellet was resuspended in 250 µl PBS and placed on ice.

Two millilitres of the blood sample was poured into a 15 ml tube, PBS was added to a total volume of 10 ml and the suspension was mixed gently. Fifteen millilitres of density gradient cell separation media (Ficoll-Paque^TM^ PLUS, GE Healthcare, Little Chalfont, UK) was added to two SepMate^TM^ tubes (STEMCELL Technologies, Cambridge, UK). Half of the diluted blood sample was then carefully added to each SepMate^TM^ tube to form a layer on the surface of the Ficoll-Paque^TM^. The tubes were centrifuged at 1200 × *g* for 15 min at room temperature. The supernatant, containing the layer of mononuclear cells, in each tube was poured into a 50 ml tube. PBS was added to a total volume of 30 ml in each tube. The cell suspensions were centrifuged at 300 × *g* for 10 min at 4°C. The supernatant was poured away and the cell pellets were resuspended in 1 ml PBS and transferred to a single FACS tube. The cell suspension then underwent the same staining protocol as the CSF cell suspension (above), including staining with LIVE/DEAD^®^, anti-CD14 V500, anti-CD19 V500, anti-CD3 APC-H7, anti-CD4 PE-Cy5.5 and anti-CD8 BV711. The washed cell pellet was resuspended in 3 ml PBS and placed on ice.

Cell sorting was performed using the FACSaria Fusion. First, the compensation tubes were loaded and any overlaps in fluorescence emission ranges for the stains/antibodies were adjusted for. Next, the stained CSF cell suspension was loaded and the first 200 cells were used to apply the compensated gates. The rest of the sample was then sorted into purified CD4^+^ T-cell populations, which were collected into genomic DNA extraction columns (AllPrep Micro kit, Qiagen, Manchester, UK) containing 350 µl RLT lysis buffer (AllPrep Micro kit, Qiagen, Manchester, UK) and 1% beta-mercaptoethanol (irreversible RNase inhibitor; Invitrogen, Paisley, UK), and placed on ice. The number of CSF CD4^+^ T-cells sorted ranged from 2 to 6073 cells/ml of CSF (median 108.1 cells/ml, mean 320.6 cells/ml) (Supplementary Fig. 3). After the CSF cell sorting was complete, the stained mononuclear cell suspension was loaded. Up to 150 000 purified CD4^+^ T-cells were collected into a 15 ml tube containing 350 µl RLT lysis buffer and 1% beta-mercaptoethanol, and placed on ice.

### Extraction of nucleic acids, library preparation and sequencing

Extraction and purification of nucleic acids were performed using the Qiagen AllPrep Micro kit (Qiagen, Manchester, UK). RNA was eluted in RNase-free water and stored at −80°C. RNA quality and quantity were measured using the Agilent Bioanalyzer (Agilent Technologies, Santa Clara, CA, USA). The range of RNA concentrations from CSF-resident CD4^+^ T-cells was 56–948 pg/µl (median 85 pgµ/l, mean 151 pg/µl). In multiple sclerosis, the mean RNA integrity number (RIN) for the samples that were included in the paired analysis was 8.4 for CSF and 7.7 for blood (*t*-test comparison *P* = 0.18). In NID controls, the mean RIN for the samples that were included in the paired analysis was 6.8 for CSF and 6.3 for NID controls (*t*-test comparison *P* = 0.50). In the extended CSF cohort, the mean RIN was 6.7 in multiple sclerosis and 5.5 in NID controls (*t*-test comparison *P* = 0.06).

RNA-seq libraries were prepared from 500 pg RNA from each sample using the Tecan Ovation SoLo RNA-seq System (Tecan Genomics, The Netherlands), a system designed for low RNA input libraries, according to the manufacturer’s protocol. In order to minimize batch effects, each batch was designed to contain paired CSF and blood RNA samples from two patients with multiple sclerosis and two NID control patients. The RNA-seq libraries underwent QC using the Agilent Bioanalyzer and were then sequenced to a median depth of 157,417,402 reads using the Illumina HiSeq4000.

### Mapping, read counting and quality control

FASTQ files containing raw RNA-seq data underwent QC using FastQC. FastQC reports for all samples were inspected for abnormalities both before and after read trimming using Trimmomatic^19^ to remove adapter sequences and bases with low sequence quality scores. Reads were mapped to the human genome (GRCh38, gene build 91) using STAR.^20^ MarkDuplicates (within the Picard suite; Broad Institute) was used to identify duplicate reads and mapping statistics were generated using BamTools.^21^ A file containing raw read counts for each gene for each sample was created using featureCounts.^22^ Normalization was performed during subsequent analyses.

### Differential gene expression and gene ontology analysis

#### Experimental design

The differential gene expression analysis had four components. The first analysis compared CSF CD4^+^ T-cells with blood CD4^+^ T-cells in 20 NID controls (paired analysis) with RIN as a covariate. This sought to identify differentially expressed genes in the absence of CNS inflammation that could be important in immune surveillance of the CNS by CD4^+^ T-cells. The second analysis compared CSF CD4^+^ T-cells with blood CD4^+^ T-cells in 21 multiple sclerosis patients (paired analysis) with RIN as a covariate. This sought to identify differentially expressed genes in multiple sclerosis. The third analysis was a two-way paired analysis of CSF CD4^+^ T-cells versus blood CD4^+^ T-cells in these 21 cases compared with the 20 controls. This sought to identify genes differentially expressed in CSF CD4^+^ T-cells when compared with blood CD4^+^ T-cells, specifically in multiple sclerosis. The final analysis compared multiple sclerosis CSF CD4^+^ T-cells (*n* = 41, including the 21 cases included in previous analyses) with NID control CSF CD4^+^ T-cells (*n* = 38, including the 20 controls used in the previous analyses) (group-wise comparison) with RIN, batch, age at sampling and sex as covariates. This analysis sought to identify genes differentially expressed between CSF CD4^+^ T-cells in multiple sclerosis and NID controls.

As CSF and blood samples for each patient were taken at the same time and each batch was arranged to minimize batch effects, the first three analyses were paired to increase power to detect differences. This design controlled for patient- and sample-specific factors that influence gene expression e.g. age, sex, batch, etc. Mapping statistics for all cohorts are shown in Supplementary Tables 5 and 6.

#### Differential expression analyses

All analyses of gene expression were completed using the Bioconductor R package, edgeR,^23^ employing multi-dimensional scaling to identify samples with outlying expression data (Supplementary Figs. 4 and 5, Supplementary Tables 7 and 8). Data for genes with low expression were removed using filterByExpr,^24^ leaving 10 457 genes for differential analysis.

#### Gene ontology analysis

Gene ontology (GO) analysis was performed using the online Database for Annotation, Visualisation and Integrated Discovery v6.8.^25^ The list of expressed genes derived from filterByExpr was used as the background gene list. Significantly differentially expressed genes at a false discovery rate (FDR) <5% were uploaded to Database for Annotation, Visualization and Integrated Discovery and pathways were considered significant at FDR <5%.

### Data availability

Gene expression data will be made available via the European Genome-phenome Archive (https://www.ebi.ac.uk/ega/home Accessed 20 July 2021).

## Results

### CSF CD4^+^ T-cells display a distinct gene expression profile to blood CD4^+^ T-cells, which is similar in non-inflammatory controls and multiple sclerosis and is predominated by migration molecules

**Figure 1:**
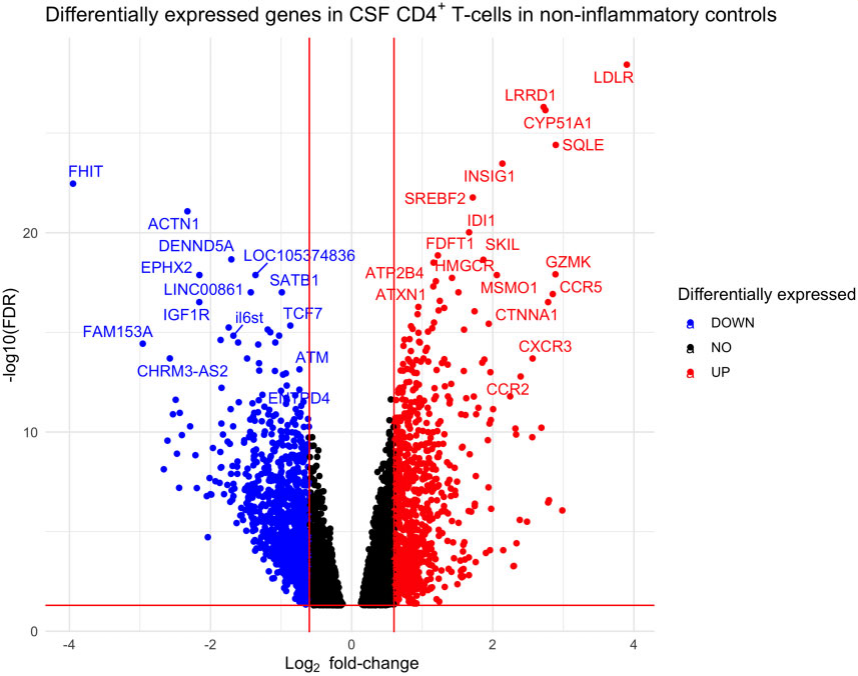
**Volcano plot showing differentially expressed genes between CSF and blood CD4^+^ T-cells in non-inflammatory controls. Log_2_ fold-change thresholds of ±0.6 and FDR 0.05 are drawn as red lines for visualization purposes**.

**Figure 2:**
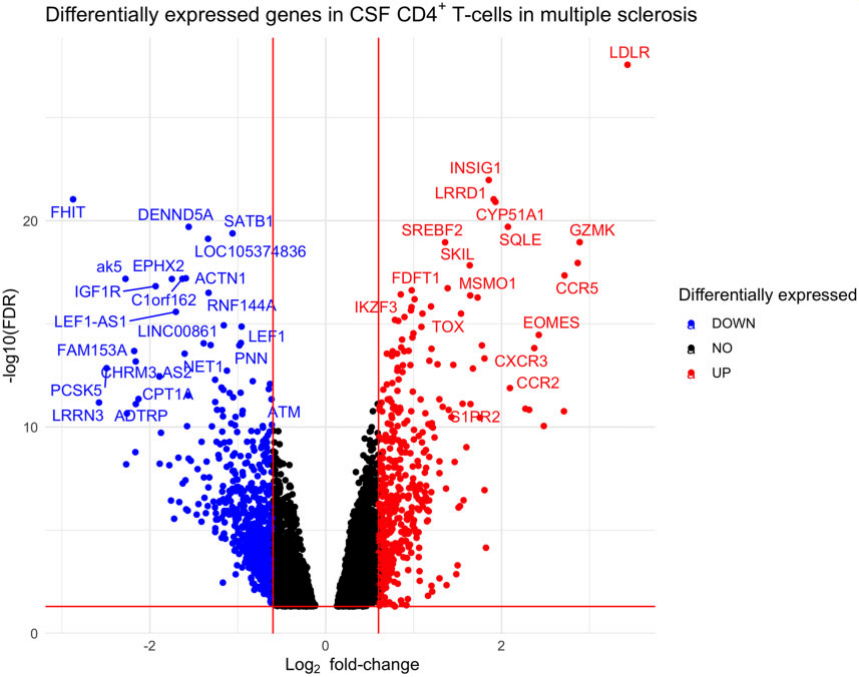
**Volcano plot showing differentially expressed genes between CSF and blood CD4^+^ T-cells in multiple sclerosis. Log_2_ fold-change thresholds of ±0.6 and FDR 0.05 are drawn as red lines for visualization purposes**.

The differential gene expression analysis between CSF CD4^+^ T-cells and blood CD4^+^ T-cells in NID controls identified 5156 significantly differentially expressed genes at FDR <5% (Fig. 1 and Supplementary Table 9): 2556 up-regulated and 2600 down-regulated. The differential gene expression analysis between CSF CD4^+^ T-cells and blood CD4^+^ T-cells in patients with multiple sclerosis identified 4263 differentially expressed genes at FDR <5% (Fig. 2 and Supplementary Table 10): 2102 up-regulated and 2161 down-regulated. Of the 4263 genes found to be significantly differentially expressed between CSF and blood in multiple sclerosis, 3511 (82%) were also significantly differentially expressed in NID controls.

The significantly differentially expressed genes in both NID controls and multiple sclerosis were predominantly genes involved in cellular migration and activation. Interestingly, genes associated with Th1 phenotype (e.g. *CXCR3*, *CCR5*; Supplementary Table 11)^26^ were significantly up-regulated in CSF. *CCR4*, a gene expressed by Th2 cells,^26^ was up-regulated in CSF (log_2_ fold-change 0.93 in NID and 1.05 in multiple sclerosis), but to a lesser extent than Th1-related genes (*CCR5* log_2_ fold change 2.85 in NID and 2.72 in multiple sclerosis) and other Th2-related genes were down-regulated in CSF (e.g. *IL4RA*, *IRF4*, *STAT5* and *STAT6*). Genes expressed by Th17 cells (e.g. *KLRB1*)^27^ and tissue-resident T-cells (e.g. *CCL5*, *LGALS1* and *PTGER2*) were significantly up-regulated in CSF CD4^+^ T-cells. Furthermore, genes associated with effector functions were also significantly up-regulated in CSF CD4^+^ T-cells, including *GZMK*, *GZMA* and *CST7*.

GO analysis of the 5156 significantly differentially expressed genes between blood and CSF CD4^+^ cells in NID controls identified 61 significant GO terms at FDR <5% (Supplementary Table 12). GO analysis of the 4263 significantly differentially expressed genes between blood and CSF CD4^+^ cells in multiple sclerosis identified 176 significant GO terms at FDR <5% (Supplementary Table 13). The GO term ‘Movement of cell or subcellular component’ was the most significant term in both NID controls (fold enrichment 1.23, FDR 5 × 10^−7^) and multiple sclerosis (fold enrichment 1.32, FDR 2.7 × 10^−10^). Fifty-eight of the 61 significant GO terms in NID controls were also significant in multiple sclerosis (Table 1), leaving 118 GO terms significant only in multiple sclerosis (Table 2) and three GO terms significant only in NID controls (Supplementary Table 14). The GO terms only significant in multiple sclerosis were predominated by those involved in cellular proliferation. There was consistency in the top GO terms when considering all significant (FDR < 0.05) genes, or taking only 1000, 500 and 100 most significant genes, with the 100 most significantly differentially expressed genes producing pronounced fold-change enrichments (Supplementary Table 15).

**Table 1:** Ten most significant gene ontology terms common to both multiple sclerosis and non-inflammatory disorder controls

GO term	Fold enrichment	FDR (NID)
Movement of cell or subcellular component	1.32	1.3 × 10^–9^
Single organismal cell-cell adhesion	1.43	1.1 × 10^–7^
Cell migration	1.35	2.0 × 10^–7^
Cell activation	1.36	2.0 × 10^–7^
Locomotion	1.31	2.0 × 10^–7^
Single organism cell adhesion	1.39	3.5 × 10^–7^
Immune response	1.27	4.5 × 10^–7^
Cell motility	1.31	7.3 × 10^–7^
Localization of cell	1.31	7.3 × 10^–7^
Biological adhesion	1.25	2.1 × 10^–6^

**Table 2:** Gene ontology terms significant only in multiple sclerosis (ten most significant shown)

GO term	Fold enrichment	FDR
Mononuclear cell proliferation	1.62	3.5 × 10^–6^
Leukocyte proliferation	1.61	3.6 × 10^–6^
Lymphocyte proliferation	1.61	7.2 × 10^–6^
Leukocyte activation	1.34	8.9 × 10^–6^
Regulation of mononuclear cell proliferation	1.68	1.3 × 10^–5^
Regulation of leukocyte proliferation	1.66	1.5 × 10^–5^
Regulation of lymphocyte proliferation	1.67	1.7 × 10^–5^
Regulation of cell adhesion	1.34	1.0 × 10^–4^
Regulation of cell proliferation	1.22	1.3 × 10^–4^
Regulation of cell activation	1.38	1.9 × 10^–4^

### Two-way paired analysis of CSF and blood CD4^+^ T-cells in multiple sclerosis compared with non-inflammatory controls identifies four genes that further implicate cholesterol biosynthesis and migration mechanisms

Gene expression differences specific to CSF CD4^+^ T-cells from patients with multiple sclerosis could be relevant to pathogenesis. It is possible that CD4^+^ T-cells in multiple sclerosis could exploit disease-specific extravasation mechanisms, are more likely to extravasate or display pro-inflammatory tendencies. To investigate this, a two-way paired differential gene expression analysis was performed between CSF CD4^+^ T-cells and blood CD4^+^ T-cells in patients with multiple sclerosis compared with NID controls. This analysis sought to identify genes where the difference in expression between CSF and blood CD4^+^ T-cells differed significantly between multiple sclerosis and NID controls. Four genes were identified that were differentially expressed in multiple sclerosis CSF CD4^+^ T-cells at FDR <5% (Table 3): one up-regulated and three down-regulated (Supplementary Fig. 6).

**Table 3:** Genes significantly differentially expressed in CSF CD4^+^ T-cells compared with blood CD4^+^ T-cells in patients with multiple sclerosis compared with NID controls

Gene symbol	Molecule	Log fold-change	*P*-value	FDR
*LRRD1*	Leucine-rich repeat and death domain-containing protein 1	−0.81	0.000003	0.02
*CYP51A1*	Lanosterol 14α-demethylase	−0.81	0.000004	0.02
*PASK*	PAS domain-containing serine-threonine-protein kinase	0.62	0.000007	0.02
*Yes1*	Tyrosine-protein kinase Yes	−1.14	0.000009	0.02

### CSF CD4^+^ T-cells in patients with multiple sclerosis compared with non-inflammatory controls identifies 140 differentially expressed genes

**Figure 3:**
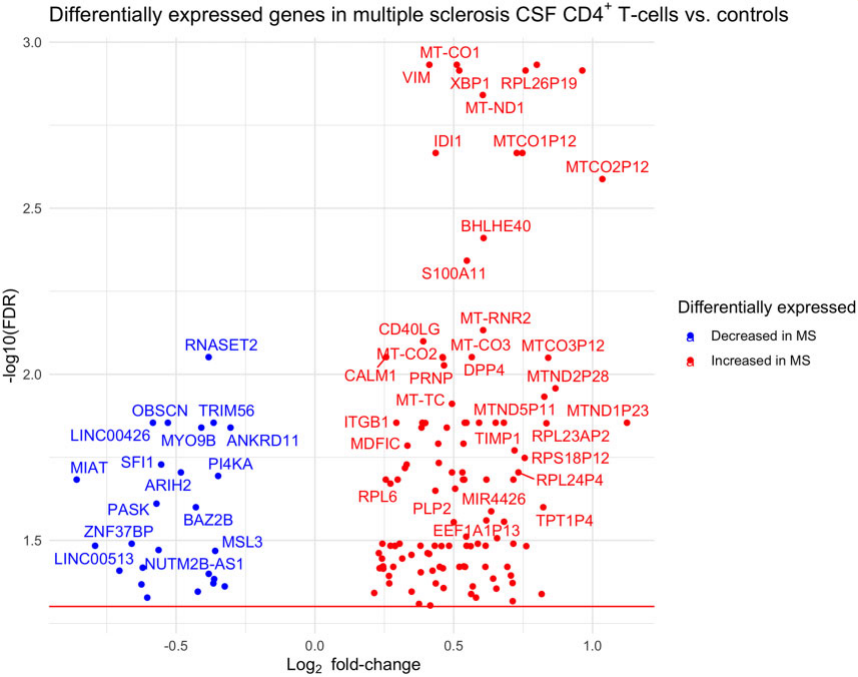
**Volcano plot showing differentially expressed genes between multiple sclerosis and non-inflammatory control CSF CD4^+^ T-cells**.

To seek further genes relevant to multiple sclerosis, a differential expression analysis was performed between an extended cohort of CSF CD4^+^ T-cells in 41 multiple sclerosis samples versus 38 NID control samples, which identified 140 significant genes at FDR <5% (Fig. 3 and Supplementary Table 16): 115 up-regulated and 25 down-regulated.

GO analysis was performed of the 140 significantly differentially expressed genes. This identified 31 significant GO terms at FDR <5% (Supplementary Table 17), which broadly relate to mitochondrial function, with many genes involved in oxidative phosphorylation.

## Discussion

Immune surveillance of the CNS plays an important role in protection from infection and maintaining health.^28–30^ The majority of cells found in CSF are CD4^+^ T-cells^4^ and the ways in which they differ from those in blood are likely to be important in the pathogenesis of multiple sclerosis and other CNS inflammatory disorders, but are incompletely understood. Here, we report thousands of differentially expressed genes in CSF CD4^+^ T-cells compared with blood CD4^+^ T-cells, irrespective of diagnosis. The results suggest substantial differences in activation status, expression of adhesion molecules and functionality of CD4^+^ T-cells within the CSF when compared with those in blood. The results also suggest that functional differences between CSF and blood CD4^+^ T-cells are far greater than differences between CSF CD4^+^ T-cells in multiple sclerosis and NID controls. Furthermore, the results also highlight genes and cellular processes that may be relevant to multiple sclerosis pathogenesis and treatment target discovery.

It has been reported that CSF cells differ from those in blood in several ways. CD4^+^ T-cells make up a higher proportion of total CSF cells compared with blood and these cells are larger than those in blood.^4^ Several studies have reported that, in health, CSF CD4^+^ T-cells express different surface proteins, such as those associated with different functional subtypes. In particular, a high proportion of CSF CD4^+^ T-cells have been reported to display a memory phenotype, either central memory or effector memory.^28^^,^^31^^,^^32^ The data we report here suggest that CSF CD4^+^ T-cells from both multiple sclerosis and NID controls are antigen-experienced. The most significantly differentially expressed gene in both multiple sclerosis and NID controls was *LDLR*, the receptor for low-density lipoprotein (LDL). The LDL receptor mediates endocytosis of cholesterol-rich LDL and has been shown to be up-regulated on the surface of T cells following mitogenic stimulation.^33^^,^^34^ Activated CD4^+^ T-cells undergo rapid proliferation as part of the adaptive immune response. Cholesterol and other sterols are required to build membranes and for intracellular signalling,^35^ so the up-regulation of sterol synthesis and cholesterol uptake observed in CSF CD4^+^ T-cells here is consistent with proliferating cells.^36^

Several studies of CNS inflammatory disorders have shown increased proportions of T-cells displaying a memory phenotype.^37–40^ Previous studies have reported that the majority of CD4^+^ T-cells in inflamed brain parenchyma are T_EM_ (effector memory) cells,^41^ which lack expression of the chemokine receptor *CCR7* and are capable of immediate effector functions. In our data, *CCR7* was significantly downregulated in CSF CD4^+^ T-cells, suggesting enhanced effector functionality. Other markers of effector function (*GZMK*, *GZMA*, *CST7*) were also up-regulated in CSF CD4^+^ T-cells in this study. These data replicate two recent single-cell transcriptomic studies in small numbers of patients, which reported that CSF CD4^+^ T-cells predominantly express markers associated with effector functions.^17^^,^^42^ Furthermore, expression of *CCR5*, a Th1 cell marker, was significantly higher in CSF CD4^+^ T-cells and it has been reported that immune surveillance of brain parenchyma is performed by T_EM_ cells expressing *GZMK* and high levels of *CCR5*.^41^

Lymphocyte homing mechanisms that permit CNS-specific CD4^+^ T-cell extravasation contain potential targets for treating CNS inflammatory disorders, such as multiple sclerosis. One of the most effective disease modifying drugs for multiple sclerosis, natalizumab, is a monoclonal antibody against integrin-α4 (ITGA4). ITGA4 is expressed on the surface of antigen-experienced CD4^+^ T-cells. By interfering with the interaction between ITGA4 and its receptor (VCAM-1), natalizumab significantly reduces extravasation of CD4^+^ T-cells into the CNS. In this study, *ITGA4* expression was significantly increased in CSF CD4^+^ T-cells when compared with blood CD4^+^ T-cells (log fold change 1.7; FDR = 6.7 × 10^−16^ and 6.5 × 10^−16^ in NID controls and multiple sclerosis, respectively). Other molecules involved in cellular migration reported here may contain novel treatment targets for multiple sclerosis and further study is warranted.

The 118 GO terms significant only in multiple sclerosis were predominated by those involved in cellular proliferation. On activation, naïve CD4^+^ T-cells proliferate, and the local cytokine milieu determines the phenotype of the differentiated cells.^43^ CSF CD4^+^ T-cell counts in multiple sclerosis were significantly higher than those of NID controls in this study (median in multiple sclerosis and NID controls 298.9 cells/ml and 81.6 cells/ml, respectively; *P* = 1.4 × 10^−6^) and this is consistent with published data.^44^ Whilst increased blood–brain- or blood–CSF-barrier permeability in multiple sclerosis is likely to be contributory, increased CD4^+^ T-cell proliferation could also be relevant. Indeed, differences in activation responses and clonal expansion of CD4^+^ T-cells has been implicated in multiple sclerosis pathogenesis.^10^^,^^45^

The two-way analysis to identify genes differentially expressed in CSF versus blood in multiple sclerosis versus non-inflammatory controls yielded four significant genes: *LRRD1, CYP51A1*, *PASK* and *Yes1*. Within the human genome, *LRRD1* and *CYP51A1* are in close proximity (within ∼30 kb). *CYP51A1* encodes lanosterol 14α-demethylase, which is an enzyme that catalyses a critical step in cholesterol synthesis in T-cells.^46^^,^^47^ Cholesterol synthesis intermediates have been shown to play important roles in regulating T-cell differentiation and inflammatory responses.^48^ The effect of pharmacological manipulation of cholesterol biosynthesis in multiple sclerosis warrants further study. *Yes1* is an Src-family kinase involved in T-cell migration through interaction with CXCL12,^49^ which is up-regulated in multiple sclerosis lesions.^50^

The direct comparison of gene expression between multiple sclerosis and non-inflammatory control CSF CD4^+^ T-cells yielded 140 significant genes and GO analysis identified 31 enriched terms. The fourth most significant gene, *XBP1* (X-box binding protein 1), is a transcription factor that regulates expression of HLA class II genes.^51–53^ HLA class II is the major multiple sclerosis genetic risk locus and is expressed on activated CD4^+^ T-cells.^54^ In this study *HLA-DRB1* was found to be significantly up-regulated in CSF CD4^+^ T-cells in both multiple sclerosis and NID controls. The function of HLA class II molecules in this context is debated and may involve activation or apoptosis of other CD4^+^ T-cells^55^ and warrants further investigation. The significant genes included many with plausible links with multiple sclerosis pathogenesis, such as *BHLHE40*,^56^ *CD40LG*,^57^ *DPP4* and *ITGB1.* The GO analysis identified 31 enriched terms, which were all related to mitochondrial function, and there is growing evidence of mitochondrial dysfunction in multiple sclerosis.^58^

To our knowledge, this is the largest transcriptomic study in purified subpopulations of CSF cells in health or disease to date. Although single-cell analysis has the potential to provide more definitive conclusions about the relative proportions of functional subtypes of CD4^+^ T-cells present in CSF, the current costs are prohibitive and restrict analysis to patient cohorts much smaller than that in this study. Whilst CD4^+^ T-cells play a key role in multiple sclerosis pathogenesis other immune cells are also important. The majority of CSF cells are CD4^+^ T-cells, but CSF also contains CD8^+^ T-cells and a limited number of other cell types. Analysis of other cell types could be informative, but the technical challenge of performing RNA-seq on sorted CSF cells is restricting. In this study, a large cohort of individuals with non-inflammatory disorders was used as controls. The non-inflammatory disorders largely consisted of idiopathic intracranial hypertension, a disorder thought to be due to impaired CSF drainage or over production.^59^ A range of other disorders made up the remaining controls. Whilst none of these disorders are thought to be primarily inflammatory, it is possible that the function of CSF immune cells is abnormal in some of these individuals. CSF from healthy individuals would be an ideal control, but it is an extremely scarce resource. In addition, inclusion of controls with non-multiple sclerosis CNS inflammatory disorders could identify genes that are more specific to multiple sclerosis pathogenesis, but the low prevalence of such disorders would have restricted the group size significantly. Finally, it is possible that the gene expression differences between multiple sclerosis and NID controls were a consequence of disease. The aetiological significance of observed gene expression differences could be explored by expression quantitative trait locus analyses to identify gene expression differences that are additionally associated with genetic risk for multiple sclerosis.

In summary, we report significant differences in gene expression between CD4^+^ T-cells from CSF and blood, and between multiple sclerosis and NID controls. Major differences were identified in activation and migration mechanisms in CD4^+^ T-cells, as well as cholesterol uptake and biosynthesis, which play a crucial role in orchestrating CD4^+^ T-cell differentiation into effector cells. The results implicate an array of adhesion molecules and chemokine receptors in extravasation of CD4^+^ T-cells into the CNS. Identified genes warrant further investigation as potential therapeutic targets for multiple sclerosis.

## Supplementary material

Supplementary material is available at *Brain Communications* online.

## Supplementary Material

fcab155_Supplementary_Data

## Abbreviations

FACS =: fluorescence-activated cell sorting
FDR =: false discovery rate
GO =: gene ontology
NID =: non-inflammatory disorder
PBS =: phosphate-buffered saline
RIN =: RNA integrity number
RNA-seq =: RNA sequencing
T_EM_ =: effector memory T-cells
