# Supplementary material for: CSF-resident CD4+ T-cells display a distinct gene expression profile with relevance to immune surveillance and multiple sclerosis
Source: Brain Commun. 2021 Jul 13;3(3):fcab155. doi: 10.1093/braincomms/fcab155 (PMC8574295; doi:10.1093/braincomms/fcab155)
Supplement: fcab155_Supplementary_Data [file fcab155_Supplementary_Data.zip › Supplementary table 10_CSFvBLOODinMS.pdf]

Supplementary table 10_CSFvBLOODinMS_FDR0.05

ID	Gene	logFC	logCPM	F	PValue	FDR
**ENSG00000130164**	LDLR	3.432395822	6.692584337	824.4146806	2.65E-32	2.77E-28
**ENSG00000186480**	INSIG1	1.854514755	6.39668639	359.5903015	2.05E-26	1.07E-22
**ENSG00000240720**	LRRD1	1.910073003	5.482373821	321.7472349	3.18E-25	9.2E-22
**ENSG00000189283**	FHIT	-2.872938192	5.817150685	397.1537114	3.52E-25	9.2E-22
**ENSG00000001630**	CYP51A1	1.930400685	5.520396658	313.7757597	5.87E-25	1.23E-21
**ENSG00000104549**	SQLE	2.072216979	5.034519901	276.4618409	1.25E-23	1.99E-20
**ENSG00000184014**	DENND5A	-1.558029178	6.121900821	275.6778394	1.33E-23	1.99E-20
**ENSG00000182568**	SATB1	-1.059813363	8.789586776	265.900998	3.15E-23	4.12E-20
**ENSG00000186854**	LOC105374836	-1.338339651	7.683379591	271.8844007	6.51E-23	7.57E-20
**ENSG00000113088**	GZMK	2.888385252	6.170775125	381.3706166	1.05E-22	1.1E-19
**ENSG00000198911**	SREBF2	1.357888037	6.835835724	251.4709064	1.18E-22	1.12E-19
**ENSG00000240535**		2.866280999	5.70691013	317.7299544	1.28E-21	1.12E-18
**ENSG00000136603**	SKIL	1.637821555	6.283337272	238.5774928	1.81E-21	1.46E-18
**ENSG00000160791**	CCR5	2.71593834	5.3340748	266.531055	6.05E-21	4.52E-18
**ENSG00000072110**	ACTN1	-1.590521699	6.576191379	230.717974	8.88E-21	6.19E-18
**ENSG00000143110**	C1orf162	-1.629460829	5.377753024	206.9185068	1.05E-20	6.59E-18
**ENSG00000154027**	ak5	-2.27716161	4.874366803	206.7661849	1.07E-20	6.59E-18
**ENSG00000120915**	EPHX2	-1.74954958	5.03751136	206.0554887	1.16E-20	6.72E-18
**ENSG00000140443**	IGF1R	-1.934320031	6.362304826	249.697556	2.73E-20	1.5E-17
**ENSG00000135842**	FAM129A	1.386725623	7.055032485	217.7865271	3.59E-20	1.88E-17
**ENSG00000079459**	FDFT1	0.977772066	6.588039196	193.4736178	4.75E-20	2.37E-17
**ENSG00000151692**	RNF144A	-1.332449393	6.051622986	190.6702667	6.58E-20	3.13E-17
**ENSG00000161405**	IKZF3	0.853481831	8.933283	188.7420695	8.24E-20	3.75E-17
**ENSG00000112972**	HMGCS1	1.643274295	5.925185172	199.8093679	9.64E-20	4.2E-17
**ENSG00000052802**	MSMO1	1.727529504	5.482285791	185.7232159	1.27E-19	5.31E-17
**ENSG00000124788**	ATXN1	1.010428719	7.230087061	183.1829027	1.59E-19	6.41E-17
**ENSG00000067064**	IDI1	1.19766307	6.140645338	176.2119858	3.73E-19	1.44E-16
**ENSG00000188452**	CERKL	0.97603014	7.285477859	175.3566241	4.14E-19	1.55E-16
**ENSG00000113532**	ST8SIA4	0.969059222	6.665145311	172.1375095	6.2E-19	2.23E-16
**ENSG00000232021**	LEF1-AS1	-1.704082262	5.112578717	170.6062377	7.52E-19	2.62E-16
**ENSG00000069974**	RAB27A	1.100061396	6.643567108	168.6209166	9.68E-19	3.2E-16
**ENSG00000198846**	TOX	1.538621365	5.309171796	168.538432	9.78E-19	3.2E-16
**ENSG00000058668**	ATP2B4	0.895724387	8.312857767	165.3771931	1.47E-18	4.66E-16
**ENSG00000115232**	ITGA4	0.787434633	9.411335899	162.6268564	2.1E-18	6.47E-16
**ENSG00000152518**	ZFP36L2	0.824827572	9.594675682	161.5979648	2.41E-18	7.2E-16

**ENSG00000245164**	LINC00861	-1.159369267	9.348106282	179.7751724	4.05E-18	1.18E-15
**ENSG00000138795**	LEF1	-0.957205159	9.210650256	157.9644619	4.8E-18	1.36E-15
**ENSG00000113161**	HMGCR	1.088453705	5.974734733	156.0054033	5.09E-18	1.4E-15
**ENSG00000160007**	arhgap35	0.997439085	6.75098677	150.5391611	1.08E-17	2.89E-15
**ENSG00000163508**	EOMES	2.42340421	4.597149605	165.1651962	1.32E-17	3.44E-15
**ENSG00000124126**	PREX1	0.986056528	7.689225117	152.5076799	1.8E-17	4.59E-15
**ENSG00000111266**	DUSP16	0.86836849	7.169869234	145.0594743	2.33E-17	5.81E-15
**ENSG00000237943**		-0.964782259	7.26620926	142.2941779	3.47E-17	8.45E-15
**ENSG00000262211**		-1.388263763	5.871197041	146.4920178	3.7E-17	8.78E-15
**ENSG00000228956**		-1.308703614	5.614502628	140.3296119	4.62E-17	1.07E-14
**ENSG00000101665**	SMAD7	1.776548441	4.188309881	139.9165272	4.91E-17	1.11E-14
**ENSG00000100941**	PNN	-0.982229282	7.869423442	147.2746778	5E-17	1.11E-14
**ENSG00000125827**	TMX4	0.858721819	7.235736175	138.1058182	6.41E-17	1.4E-14
**ENSG00000186810**	CXCR3	2.372437137	5.301991824	177.9998359	7.05E-17	1.5E-14
**ENSG00000111728**	ST8SIA1	1.203267607	5.473482147	136.8126913	7.77E-17	1.62E-14
**ENSG00000151553**	FAM160B1	0.943550562	6.416103428	135.1967311	9.89E-17	2.03E-14
**ENSG00000170074**	FAM153A	-2.17824751	5.296242863	164.5195241	1.03E-16	2.08E-14
**ENSG00000149177**	PTPRJ	0.892961449	7.168129043	134.5454538	1.09E-16	2.14E-14
**ENSG00000140575**	iqgap1	0.760925541	9.068556814	134.4510305	1.11E-16	2.14E-14
**ENSG00000101096**	NFATC2	0.6951104	7.937864262	132.5466511	1.48E-16	2.79E-14
**ENSG00000012660**	ELOVL5	0.811960765	7.247336613	132.4735357	1.49E-16	2.79E-14
**ENSG00000173848**	NET1	-1.604489523	4.527210419	132.3345259	1.53E-16	2.8E-14
**ENSG00000116824**	CD2	0.752874948	8.082335181	130.5153638	2.02E-16	3.64E-14
**ENSG00000183508**	FAM46C	1.805615356	5.540051039	151.577812	2.69E-16	4.77E-14
**ENSG00000130402**	ACTN4	1.17867614	7.271872003	148.2957749	3.54E-16	6.18E-14
**ENSG00000233355**	CHRM3-AS2	-2.160778522	6.914246084	188.9013273	3.9E-16	6.69E-14
**ENSG00000148175**	STOM	1.271815853	6.72342457	143.0464208	5.44E-16	9.17E-14
**ENSG00000100385**	IL2RB	1.451087586	6.544775659	151.8716538	5.88E-16	9.76E-14
**ENSG00000028137**	MIR7846	0.962981989	6.53180697	123.4578326	6.11E-16	9.98E-14
**ENSG00000101445**	PPP1R16B	0.877629583	6.024623718	123.2199186	6.35E-16	1.02E-13
**ENSG00000047365**	ARAP2	0.818462911	7.868589409	121.4023408	8.5E-16	1.35E-13
**ENSG00000099139**	PCSK5	-2.492102167	5.137272195	155.8893319	9.03E-16	1.41E-13
**ENSG00000125735**	TNFSF14	1.673926675	5.272016142	129.8657185	9.54E-16	1.47E-13
**ENSG00000177311**	ZBTB38	0.81400095	7.624430946	119.0331965	1.25E-15	1.87E-13
**ENSG00000246223**	LINC01550	-1.123682335	5.75349394	119.0249229	1.25E-15	1.87E-13
**ENSG00000204977**	TRIM13	0.872981665	6.175185195	118.0342481	1.47E-15	2.17E-13
**ENSG00000110090**	CPT1A	-1.890947137	5.253371193	132.9419138	2.48E-15	3.6E-13

**ENSG00000197442**	MAP3K5	0.828713195	6.683770745	112.9411313	3.46E-15	4.96E-13
**ENSG00000134352**	il6st	-1.25822798	9.002741151	147.2419233	3.63E-15	5.13E-13
**ENSG00000146285**	SCML4	-0.829958585	6.768903667	111.6331218	4.33E-15	6.04E-13
**ENSG00000054654**	SYNE2	0.757140917	10.56060203	110.3402442	5.41E-15	7.44E-13
**ENSG00000135426**	TESPA1	-0.634238662	7.741376136	109.7046434	6.04E-15	8.2E-13
**ENSG00000091490**	SEL1L3	0.695005221	6.824878685	109.2955126	6.48E-15	8.69E-13
**ENSG00000144802**	nfkbiz	-1.185202816	7.195295618	129.1918123	9.32E-15	1.23E-12
**ENSG00000121807**	CCR2	2.095312564	6.31666687	151.8788109	9.96E-15	1.3E-12
**ENSG00000081059**	TCF7	-0.647458703	9.449192462	106.1516772	1.13E-14	1.45E-12
**ENSG00000120063**	GNA13	0.654825719	8.108627617	105.7206806	1.21E-14	1.55E-12
**ENSG00000034053**	APBA2	-1.163972166	5.509694163	105.5570226	1.25E-14	1.58E-12
**ENSG00000117090**	SLAMF1	0.815776191	6.510870762	104.8852093	1.41E-14	1.76E-12
**ENSG00000153094**	BCL2L11	0.990224183	5.950410098	104.2544425	1.58E-14	1.94E-12
**ENSG00000147408**	CSGALNACT1	-0.968198802	6.049468005	103.532744	1.8E-14	2.19E-12
**ENSG00000136153**	LMO7	-1.093031564	5.628080161	103.3000765	1.87E-14	2.25E-12
**ENSG00000181690**	PLAG1	-1.563095478	4.482750629	101.7553163	2.48E-14	2.95E-12
**ENSG00000160593**	JAML	-1.052145385	6.35314142	104.2533095	3.11E-14	3.65E-12
**ENSG00000165527**	ARF6	0.727040094	7.873421368	99.42869438	3.8E-14	4.4E-12
**ENSG00000111863**	ADTRP	-2.128932044	4.793221524	115.3278774	3.83E-14	4.4E-12
**ENSG00000149311**	ATM	-0.616909118	10.2696002	99.18289614	3.98E-14	4.52E-12
**ENSG00000057657**	PRDM1	1.292564034	7.57518954	130.1225907	4.04E-14	4.54E-12
**ENSG00000092820**	EZR	0.705078124	8.03567466	98.9757677	4.13E-14	4.6E-12
**ENSG00000213719**	CLIC1	1.079505208	5.798772878	98.90076934	4.19E-14	4.61E-12
**ENSG00000173597**	SULT1B1	-1.256941476	5.614003254	100.1123177	5.64E-14	6.14E-12
**ENSG00000166949**	SMAD3	0.746535013	7.016052229	97.24965578	5.7E-14	6.15E-12
**ENSG00000173114**	LRRN3	-2.578469228	5.75969735	135.6491163	6.1E-14	6.51E-12
**ENSG00000119314**	PTBP3	0.634583796	8.590667651	96.77450506	6.24E-14	6.59E-12
**ENSG00000267534**	S1PR2	1.556624213	3.84281062	96.04727312	7.15E-14	7.48E-12
**ENSG00000180370**	PAK2	0.590657331	8.029485298	95.8315745	7.45E-14	7.71E-12
**ENSG00000281106**	LINC00282	-2.159806894	4.448562801	106.3777899	7.58E-14	7.74E-12
**ENSG00000261604**		1.646248017	4.298385823	95.71068078	7.62E-14	7.74E-12
**ENSG00000088179**	PTPN4	0.61291236	8.24899438	94.32107721	9.93E-14	9.99E-12
**ENSG00000044115**	CTNNA1	1.330696779	4.753366731	93.9613185	1.06E-13	1.05E-11
**ENSG00000181467**	RAP2B	0.787096653	7.160339978	93.94572186	1.07E-13	1.05E-11
**ENSG00000109861**	CTSC	0.844306185	6.624906021	93.3431093	1.2E-13	1.17E-11
**ENSG00000106546**	AHR	0.765608656	7.006653171	93.29005697	1.21E-13	1.17E-11
**ENSG00000078596**	ITM2A	0.765087286	6.969864282	93.01927506	1.28E-13	1.22E-11

**ENSG00000077984**	CST7	2.271051452	5.333146335	123.1512408	1.37E-13	1.31E-11
**ENSG00000120137**	PANK3	0.611161398	7.479778677	92.09117845	1.53E-13	1.44E-11
**ENSG00000138814**	PPP3CA	0.849241433	6.253301484	91.97475912	1.56E-13	1.46E-11
**ENSG00000181104**	F2R	2.314614275	4.633074556	112.1747749	1.59E-13	1.47E-11
**ENSG00000168056**	LTBP3	-1.235820756	6.29274441	106.1135621	1.61E-13	1.47E-11
**ENSG00000059804**	SLC2A3	0.792466884	7.258760277	92.53943231	1.62E-13	1.47E-11
**ENSG00000146376**	arhgap18	1.398401238	4.276575994	91.72361632	1.64E-13	1.48E-11
**ENSG00000117643**	MAN1C1	-1.178018017	5.448720283	91.57876845	1.69E-13	1.51E-11
**ENSG00000166323**	c11orf65	-0.94469591	5.296592676	91.1492208	1.83E-13	1.63E-11
**ENSG00000085832**	EPS15	0.536335371	7.916854409	90.79525405	1.97E-13	1.72E-11
**ENSG00000163171**	CDC42EP3	0.972536799	7.243359239	102.1916732	1.97E-13	1.72E-11
**ENSG00000145649**	GZMA	2.709608903	5.521090081	125.8473589	2E-13	1.73E-11
**ENSG00000136167**	LCP1	0.642310014	9.651482141	90.56803608	2.05E-13	1.76E-11
**ENSG00000169756**	LIMS1	0.827074844	7.342678526	93.56395006	2.36E-13	2E-11
**ENSG00000244509**	APOBEC3C	1.133109573	6.030089886	96.09303934	2.57E-13	2.17E-11
**ENSG00000182230**	LOC100507387	-2.260662075	4.598290625	105.5566711	2.66E-13	2.22E-11
**ENSG00000198932**	GPRASP1	-0.949292113	6.042104792	88.88945765	2.86E-13	2.37E-11
**ENSG00000135272**	MDFIC	0.747791997	8.16868833	90.60087351	3.3E-13	2.71E-11
**ENSG00000126353**	CCR7	-1.168176529	7.772400492	112.3243479	3.75E-13	3.06E-11
**ENSG00000185946**	RNPC3	-1.025930776	6.849878416	97.63205029	4.01E-13	3.25E-11
**ENSG00000152492**	CCDC50	1.429509733	4.949806984	89.8048588	4.15E-13	3.34E-11
**ENSG00000172215**	Cxcr6	1.752884037	4.579913587	92.85532162	4.46E-13	3.56E-11
**ENSG00000163297**	ANTXR2	0.754774181	6.947828917	86.50452048	4.61E-13	3.65E-11
**ENSG00000137710**	RDX	0.9264095	5.419405819	84.96865303	6.3E-13	4.95E-11
**ENSG00000178573**	MAF	1.20685811	7.987688721	108.9696468	8.36E-13	6.53E-11
**ENSG00000119900**	OGFRL1	0.894901401	5.772654152	83.38016582	8.73E-13	6.74E-11
**ENSG00000121578**	B4GALT4	-1.074841109	4.801720775	83.36013378	8.76E-13	6.74E-11
**ENSG00000067955**	CBFB	0.705536829	7.103611495	83.31018403	8.85E-13	6.76E-11
**ENSG00000182718**	ANXA2	1.16780883	6.527986528	96.92364737	9.64E-13	7.3E-11
**ENSG00000169499**	PLEKHA2	0.73992234	6.877867879	82.59085107	1.03E-12	7.73E-11
**ENSG00000247774**	PCED1B-AS1	-0.612947847	7.420240556	82.49631698	1.05E-12	7.83E-11
**ENSG00000074966**	TXK	-1.031760336	6.825965263	93.33088944	1.12E-12	8.28E-11
**ENSG00000100599**	rin3	-0.716405767	6.190415215	81.7423345	1.23E-12	8.97E-11
**ENSG00000283321**		0.817497248	6.223325793	81.71294327	1.23E-12	8.97E-11
**ENSG00000271503**	CCL5	2.480538089	7.814701142	111.7334601	1.23E-12	8.97E-11
**ENSG00000087074**	PPP1R15A	-1.576788648	4.914858178	88.13236065	1.26E-12	9.12E-11
**ENSG00000143409**	FAM63A	-1.293900468	4.752167287	81.45317276	1.3E-12	9.33E-11

**ENSG00000169710**	FASN	1.20443632	5.826378486	89.39627944	1.33E-12	9.43E-11
**ENSG00000150867**	PIP4K2A	0.522997243	8.251778143	80.96717306	1.44E-12	1.02E-10
**ENSG00000104490**	NCALD	1.218708161	4.688230051	80.75575065	1.51E-12	1.06E-10
**ENSG00000257621**	PSMA3-AS1	-0.713588121	7.733134693	82.32646093	1.53E-12	1.07E-10
**ENSG00000128245**	YWHAH	1.003484405	5.388903833	79.4215828	2E-12	1.38E-10
**ENSG00000103187**	COTL1	0.769000172	7.502816101	82.66242263	2.21E-12	1.52E-10
**ENSG00000169641**	LUZP1	0.759229485	5.78076795	78.92967505	2.22E-12	1.52E-10
**ENSG00000091409**	ITGA6	-0.593037541	8.139382912	78.86345085	2.25E-12	1.53E-10
**ENSG00000229619**	MBNL1-AS1	-0.845878628	6.809924699	81.51812027	2.32E-12	1.56E-10
**ENSG00000247556**		0.510390777	8.245000123	78.56766303	2.4E-12	1.6E-10
**ENSG00000120948**	TARDBP	-0.539263335	7.591322118	78.55108457	2.41E-12	1.6E-10
**ENSG00000009790**	TRAF3IP3	-0.776606029	8.641075485	84.68865617	2.52E-12	1.66E-10
**ENSG00000145703**	IQGAP2	0.501376217	8.3447009	78.3256429	2.53E-12	1.66E-10
**ENSG00000277726**		-0.637510148	6.888444669	78.27622714	2.55E-12	1.67E-10
**ENSG00000157796**	WDR19	-1.176605272	4.613928165	78.20552701	2.59E-12	1.68E-10
**ENSG00000235437**	LINC01278	-1.007270616	5.500284646	77.99293459	2.71E-12	1.75E-10
**ENSG00000002586**	CD99	0.781344224	6.47857606	77.94724806	2.74E-12	1.76E-10
**ENSG00000196914**	ARHGEF12	1.101892544	5.242590719	77.77412626	2.84E-12	1.81E-10
**ENSG00000213366**	GSTM2	-1.873663499	4.388066749	82.58769244	3.01E-12	1.91E-10
**ENSG00000213064**	SFT2D2	0.590316691	7.809401804	77.35069915	3.11E-12	1.96E-10
**ENSG00000162739**	SLAMF6	0.681351411	6.999994816	76.32976629	3.88E-12	2.43E-10
**ENSG00000184384**	MAML2	-0.658056183	7.785636436	76.18716817	4.01E-12	2.49E-10
**ENSG00000239713**	APOBEC3G	1.057649149	5.515252023	75.78298312	4.39E-12	2.72E-10
**ENSG00000186469**	GNG2	0.607517151	7.606660561	75.26100266	4.91E-12	3.02E-10
**ENSG00000111859**	NEDD9	0.769804733	6.072356393	75.09930641	5.08E-12	3.11E-10
**ENSG00000138172**	CALHM2	1.237375807	4.81547331	74.91897694	5.29E-12	3.22E-10
**ENSG00000197217**	ENTPD4	-0.681047504	7.427137277	74.39790115	5.93E-12	3.59E-10
**ENSG00000113448**	PDE4D	0.712518595	6.903204209	74.28956968	6.08E-12	3.64E-10
**ENSG00000060237**	WNK1	0.442858185	9.381213736	74.28353995	6.08E-12	3.64E-10
**ENSG00000124942**	AHNAK	0.721269042	11.69858562	74.10329823	6.33E-12	3.76E-10
**ENSG00000111640**	GAPDH	0.686792304	8.063696697	75.95345852	6.49E-12	3.84E-10
**ENSG00000147044**	CASK	0.633977252	6.541355163	73.91593303	6.6E-12	3.88E-10
**ENSG00000164430**	MB21D1	1.001878806	4.963244314	73.70485177	6.92E-12	4.04E-10
**ENSG00000251562**	MALAT1	-0.688720884	15.3114953	73.44141688	7.33E-12	4.26E-10
**ENSG00000006125**	AP2B1	0.502648759	7.820807613	73.28155359	7.6E-12	4.38E-10
**ENSG00000168807**	SNTB2	0.825688102	6.171927443	73.26302887	7.63E-12	4.38E-10
**ENSG00000268027**		-0.687488181	6.443447988	73.17002447	7.79E-12	4.45E-10

**ENSG00000005302**	MSL3	-0.703562867	7.068760988	72.91851695	8.24E-12	4.68E-10
**ENSG00000163659**	TIPARP	0.733965458	5.897393683	72.84011235	8.38E-12	4.74E-10
**ENSG00000163820**	FYCO1	0.685998174	6.645061229	72.8004099	8.46E-12	4.76E-10
**ENSG00000137504**	CREBZF	-0.712238694	7.151374657	72.83900786	8.71E-12	4.87E-10
**ENSG00000132485**	ZRANB2	-0.830694613	7.853385337	81.5401422	9.08E-12	5.05E-10
**ENSG00000120798**	NR2C1	-0.894648356	5.820139622	72.32880875	9.4E-12	5.2E-10
**ENSG00000130338**	TULP4	0.580089109	7.110964942	72.28739344	9.49E-12	5.22E-10
**ENSG00000163596**	ICA1L	-1.411609709	4.453724175	72.21256586	9.65E-12	5.24E-10
**ENSG00000213186**	trim59	0.755783788	6.304850131	72.21239604	9.65E-12	5.24E-10
**ENSG00000107104**	KANK1	-1.241257642	4.565475531	72.1791305	9.72E-12	5.24E-10
**ENSG00000186814**	ZSCAN30	-1.094548789	5.04969899	72.17752095	9.72E-12	5.24E-10
**ENSG00000145220**	LYAR	0.886384036	5.335990585	72.04484539	1E-11	5.37E-10
**ENSG00000121210**	KIAA0922	-0.584089233	7.663443044	71.83717509	1.05E-11	5.58E-10
**ENSG00000181847**	Tigit	1.095021762	5.651208654	75.54252777	1.05E-11	5.58E-10
**ENSG00000108654**	Mir3064	-0.668126233	10.50672841	71.77503806	1.06E-11	5.6E-10
**ENSG00000235532**		-1.152158062	5.823611347	79.01408214	1.07E-11	5.6E-10
**ENSG00000197930**	ERO1A	0.555177819	7.887240892	71.52090836	1.13E-11	5.89E-10
**ENSG00000196329**	GIMAP5	-0.524951401	8.198779243	70.83823279	1.32E-11	6.83E-10
**ENSG00000204219**	TCEA3	-1.200083719	4.629713181	70.82852365	1.32E-11	6.83E-10
**ENSG00000164111**	ANXA5	0.859554088	5.654622615	70.69312681	1.36E-11	7.01E-10
**ENSG00000131504**	DIAPH1	0.500784926	8.492946829	70.36999285	1.46E-11	7.5E-10
**ENSG00000278217**		-0.723776356	11.00855619	71.89248061	1.61E-11	8.21E-10
**ENSG00000113368**	LMNB1	0.917217119	5.293204571	69.76042649	1.68E-11	8.54E-10
**ENSG00000118263**	KLF7	-0.823663766	6.042508858	69.23397335	1.9E-11	9.58E-10
**ENSG00000111796**	Klrb1	1.599894765	7.094117504	90.81008705	1.91E-11	9.58E-10
**ENSG00000156299**	TIAM1	-0.669435887	6.759475428	68.94591903	2.03E-11	1.01E-09
**ENSG00000196405**	EVL	-0.734668877	8.817948657	74.27951245	2.09E-11	1.04E-09
**ENSG00000102780**	DGKH	0.731156309	6.754687294	68.55143059	2.22E-11	1.1E-09
**ENSG00000147065**	MSN	0.536638109	9.244651726	68.43673271	2.28E-11	1.13E-09
**ENSG00000246790**		-1.128970005	4.664858306	68.2474113	2.38E-11	1.17E-09
**ENSG00000258377**		0.846093847	5.301600432	67.92823781	2.57E-11	1.25E-09
**ENSG00000168282**	mgat2	0.845355913	5.306828135	67.90501376	2.58E-11	1.26E-09
**ENSG00000109107**	ALDOC	1.139436605	4.23864556	67.69289471	2.71E-11	1.31E-09
**ENSG00000235750**	KIAA0040	0.516463104	6.941192587	67.56328122	2.79E-11	1.35E-09
**ENSG00000007944**	MYLIP	-0.813396641	5.844724913	67.41071632	2.9E-11	1.39E-09
**ENSG00000135185**	TMEM243	-0.596352147	6.105698248	67.04669343	3.15E-11	1.51E-09
**ENSG00000077044**	DGKD	-0.665139934	6.762888051	66.98184591	3.2E-11	1.52E-09

**ENSG00000269968**		0.648512669	7.160337989	66.92110743	3.25E-11	1.54E-09
**ENSG00000261371**	PECAM1	-2.164800119	4.807057363	81.53638279	3.53E-11	1.66E-09
**ENSG00000150093**	ITGB1	0.862123602	9.128056157	77.46800338	3.63E-11	1.7E-09
**ENSG00000105953**	OGDH	0.650655551	6.767230978	66.37779811	3.69E-11	1.71E-09
**ENSG0000008517**	IL32	0.907102419	8.096432528	78.88604173	3.69E-11	1.71E-09
**ENSG00000188529**	SRSF10	-0.575802867	7.741390773	66.35525114	3.71E-11	1.71E-09
**ENSG00000241839**	PLEKHO2	1.116749097	4.309549653	66.34692425	3.72E-11	1.71E-09
**ENSG00000122862**	SRGN	0.962556006	7.125803418	77.2339185	4.08E-11	1.87E-09
**ENSG00000172292**	CERS6	-1.034651684	5.526830823	67.29043531	4.11E-11	1.88E-09
**ENSG00000154229**	PRKCA	-0.692381055	7.246256682	66.97386689	4.47E-11	2.03E-09
**ENSG00000170802**	FOXN2	0.597102147	7.305037083	65.15966226	4.92E-11	2.23E-09
**ENSG00000146021**	KLHL3	-1.22018026	4.9490354	66.47970521	5.04E-11	2.27E-09
**ENSG00000169045**	HNRNPH1	-0.706398018	10.20731317	68.2654429	5.26E-11	2.36E-09
**ENSG00000010404**	IDS	0.428194015	8.083405263	64.85504884	5.29E-11	2.36E-09
**ENSG00000132424**	PNISR	-1.055691189	8.869846838	81.11794568	5.55E-11	2.47E-09
**ENSG00000089060**	SLC8B1	-0.878167785	5.749853835	64.52822815	5.72E-11	2.53E-09
**ENSG00000145075**	LOC101928882	-0.969671377	6.169621994	69.5925783	5.8E-11	2.56E-09
**ENSG00000267598**		1.17786412	5.471442898	68.98737039	6.41E-11	2.82E-09
**ENSG00000128989**	ARPP19	0.533528092	7.803198113	63.70996212	6.96E-11	3.05E-09
**ENSG00000112394**	SLC16A10	-1.675146331	4.684273034	71.06924575	7.07E-11	3.08E-09
**ENSG00000089280**	FUS	-0.624431167	8.696098389	64.90582933	7.22E-11	3.12E-09
**ENSG00000171316**	CHD7	-0.674960104	6.773598878	63.55254681	7.23E-11	3.12E-09
**ENSG00000086062**	B4GALT1	0.616905311	6.587309902	63.49121129	7.34E-11	3.16E-09
**ENSG00000147457**	CHMP7	-0.564981438	7.478611162	63.36959188	7.56E-11	3.24E-09
**ENSG00000171310**	CHST11	0.651017036	6.232834756	63.32729271	7.63E-11	3.26E-09
**ENSG00000114423**	CBLB	0.468731202	7.502500005	63.26935947	7.74E-11	3.29E-09
**ENSG00000182463**	TSHZ2	-1.212754446	6.043722709	74.13148914	7.88E-11	3.34E-09
**ENSG00000148429**	USP6NL	-1.559147874	3.70763644	63.10356122	8.06E-11	3.4E-09
**ENSG00000214262**	ANKRD36BP1	0.722956088	5.778363412	62.7818595	8.71E-11	3.66E-09
**ENSG00000176871**	WSB2	0.845816437	5.201979992	62.61500337	9.07E-11	3.79E-09
**ENSG00000171867**	PRNP	0.795505264	6.413318793	63.59765814	1.03E-10	4.31E-09
**ENSG00000125347**	IRF1	-0.632441782	7.702300668	63.31390749	1.04E-10	4.33E-09
**ENSG00000163931**	TKT	0.749796005	6.090371222	61.98167676	1.06E-10	4.36E-09
**ENSG00000166405**	RIC3	-1.533811462	4.746262702	67.85962704	1.06E-10	4.36E-09
**ENSG00000183813**	CCR4	1.045236735	6.599340738	72.39349507	1.08E-10	4.45E-09
**ENSG00000140471**	LINS1	-0.538347274	7.190203515	61.85209992	1.09E-10	4.46E-09
**ENSG00000177200**	CHD9	0.493257489	7.27926879	61.76928641	1.11E-10	4.54E-09

**ENSG00000112419**	PHACTR2	0.760127791	7.153009391	65.96587296	1.13E-10	4.59E-09
**ENSG00000138386**	NAB1	0.797209267	5.374198693	61.69212845	1.14E-10	4.59E-09
**ENSG00000047188**	YTHDC2	-0.692499415	6.829231372	61.67345197	1.14E-10	4.59E-09
**ENSG00000134107**	BHLHE40	1.293850317	6.403102352	76.01531876	1.16E-10	4.64E-09
**ENSG00000145012**	LPP	0.607038481	7.19859348	61.53993833	1.18E-10	4.71E-09
**ENSG00000166432**	ZMAT1	-1.354394808	6.198550103	75.9822155	1.2E-10	4.76E-09
**ENSG00000101752**	MIB1	0.520883619	6.955664878	61.38281463	1.23E-10	4.85E-09
**ENSG00000075426**	FOSL2	1.46675507	4.941783101	67.8071673	1.25E-10	4.94E-09
**ENSG00000188811**	NHLRC3	-0.894934915	5.314118498	60.95745544	1.36E-10	5.35E-09
**ENSG00000160310**	PRMT2	-0.503681143	7.466705739	60.75239718	1.43E-10	5.61E-09
**ENSG00000174500**	GCSAM	-0.914356232	5.355740542	60.55513716	1.5E-10	5.86E-09
**ENSG00000064666**	CNN2	0.571646369	8.02143245	60.48722228	1.53E-10	5.94E-09
**ENSG00000161381**	PLXDC1	-1.888009682	4.532600244	69.09281466	1.56E-10	6.04E-09
**ENSG00000281649**	EBLN3P	-0.483290758	7.224755656	60.36757126	1.57E-10	6.07E-09
**ENSG00000266714**	MYO15B	-2.26939658	5.112036088	75.98621915	1.68E-10	6.44E-09
**ENSG00000136279**	MIR6837	-0.582772364	7.004828843	60.10451027	1.68E-10	6.44E-09
**ENSG00000165819**	mettl3	-0.658732229	6.428209252	59.99344409	1.73E-10	6.59E-09
**ENSG00000213930**	GALT	-1.082819903	6.239469772	69.29826586	1.75E-10	6.64E-09
**ENSG00000177034**	MTX3	-0.754826662	6.158955191	59.86121521	1.79E-10	6.76E-09
**ENSG00000185404**	SP140L	-0.589960191	6.556816715	59.81031442	1.81E-10	6.82E-09
**ENSG00000163961**	RNF168	0.581096098	6.477136079	59.79543115	1.81E-10	6.83E-09
**ENSG00000131051**	RBM39	-0.52154925	9.269927964	59.72144657	1.85E-10	6.93E-09
**ENSG00000111913**	FAM65B	-0.413473271	9.780101482	59.69641946	1.86E-10	6.95E-09
**ENSG00000104093**	DMXL2	-1.778644603	4.054261563	63.10769326	1.92E-10	7.13E-09
**ENSG00000129625**	REEP5	0.635228864	6.754039994	59.51361447	1.95E-10	7.22E-09
**ENSG00000170017**	ALCAM	1.053820851	4.64270152	59.30449152	2.05E-10	7.58E-09
**ENSG00000196352**	CD55	-0.59898313	6.780832768	59.20496409	2.1E-10	7.74E-09
**ENSG00000236213**		0.989747594	4.953598082	58.95016233	2.24E-10	8.22E-09
**ENSG00000163728**	TTC14	-0.839496068	7.768018525	68.44620011	2.29E-10	8.38E-09
**ENSG00000159128**	IFNGR2	-1.125816893	4.190222254	58.79107043	2.33E-10	8.5E-09
**ENSG00000133302**	SLF1	0.776019799	5.65965101	58.53358056	2.49E-10	9.02E-09
**ENSG00000123684**	LPGAT1	0.714289873	6.310881362	58.52782807	2.49E-10	9.02E-09
**ENSG00000110651**	CD81	0.805723925	5.639711881	58.49738547	2.51E-10	9.06E-09
**ENSG00000152061**	RABGAP1L	0.51014435	7.657668082	58.40365942	2.57E-10	9.24E-09
**ENSG00000020633**	RUNX3	0.638849588	6.715729365	58.27597635	2.66E-10	9.51E-09
**ENSG00000103257**	SLC7A5	1.142316362	3.725042436	58.25899913	2.67E-10	9.52E-09
**ENSG00000164951**	PDP1	0.65099832	6.171289012	58.22767216	2.69E-10	9.56E-09

**ENSG00000113580**	NR3C1	0.465328853	7.532716182	58.10418403	2.77E-10	9.83E-09
**ENSG00000141367**	CLTC	0.462522048	8.215046171	57.89123634	2.93E-10	1.03E-08
**ENSG00000109929**	SC5D	0.849170523	5.366446477	57.85974647	2.95E-10	1.04E-08
**ENSG00000100650**	SRSF5	-0.738347619	8.973218872	64.41997902	3.05E-10	1.07E-08
**ENSG00000134909**	ARHGAP32	-1.457044815	4.068619827	57.6198221	3.14E-10	1.1E-08
**ENSG00000139644**	TMBIM6	0.537279851	8.707798634	57.45466284	3.27E-10	1.14E-08
**ENSG00000170540**	ARL6IP1	0.627346105	6.905229308	57.36144212	3.35E-10	1.16E-08
**ENSG00000154814**	OXNAD1	-0.575118364	7.85455766	57.22093165	3.47E-10	1.2E-08
**ENSG00000147894**	C9orf72	-0.817020689	5.45311812	57.17100418	3.51E-10	1.21E-08
**ENSG00000198668**	CALM1	0.453029699	9.646588237	57.16931737	3.52E-10	1.21E-08
**ENSG00000117280**	RAB29	0.614428192	6.160224767	56.95387996	3.71E-10	1.27E-08
**ENSG00000183918**	SH2D1A	0.543708032	6.561413618	56.80942146	3.85E-10	1.32E-08
**ENSG00000212694**	LINC01089	-1.059086551	5.584733919	60.61095171	3.94E-10	1.34E-08
**ENSG00000120662**	MTRF1	-1.043867315	5.052907617	56.71107762	3.95E-10	1.34E-08
**ENSG00000164924**	YWHAZ	0.457923846	9.598698121	56.41785853	4.26E-10	1.44E-08
**ENSG00000023516**	AKAP11	0.462215202	8.128145324	56.25604004	4.44E-10	1.5E-08
**ENSG00000240303**	ACAD11	-0.945657611	5.093237403	56.15992826	4.55E-10	1.53E-08
**ENSG00000134308**	YWHAQ	0.631584466	6.812047773	56.15001675	4.56E-10	1.53E-08
**ENSG00000115935**	WIPF1	0.342988313	9.399506427	56.14385074	4.57E-10	1.53E-08
**ENSG00000154153**	fam134b	-0.706580957	5.460180541	56.11315133	4.61E-10	1.53E-08
**ENSG00000159445**	THEM4	-0.617314861	6.722490658	56.08488978	4.64E-10	1.54E-08
**ENSG00000135679**	MDM2	0.459884047	7.335221321	56.02596928	4.71E-10	1.56E-08
**ENSG00000160888**	IER2	1.127373128	6.140443532	65.34696942	4.98E-10	1.64E-08
**ENSG00000138758**	40787	0.647579839	5.994478146	55.72198318	5.1E-10	1.68E-08
**ENSG00000151835**	SACS	0.489941781	7.672746542	55.60949556	5.25E-10	1.72E-08
**ENSG00000162783**	IER5	1.293734795	4.001729412	55.58346034	5.28E-10	1.73E-08
**ENSG00000176624**	MEX3C	0.569201899	6.626033883	55.37066202	5.58E-10	1.82E-08
**ENSG00000155893**	PXYLP1	-1.028843803	4.752901119	55.24672869	5.76E-10	1.86E-08
**ENSG00000162757**	C1orf74	-0.581903266	7.035490402	55.2429732	5.77E-10	1.86E-08
**ENSG00000213402**	PTPRCAP	0.825469397	6.015948487	56.65906241	5.77E-10	1.86E-08
**ENSG00000123240**	OPTN	0.476617354	7.324560891	55.10437257	5.98E-10	1.92E-08
**ENSG00000095951**	hivep1	-0.567559657	6.751310438	55.07921026	6.02E-10	1.93E-08
**ENSG00000110047**	EHD1	0.511596843	6.906221677	55.0704177	6.03E-10	1.93E-08
**ENSG00000152795**	HNRNPDL	-0.511677552	8.520703452	55.01382059	6.12E-10	1.95E-08
**ENSG00000027869**	SH2D2A	0.906769768	4.938758074	54.57712107	6.86E-10	2.18E-08
**ENSG00000204271**	SPIN3	-0.941458931	4.963567791	54.50482848	6.99E-10	2.21E-08
**ENSG00000112182**	BACH2	-0.774694069	6.856225606	58.06103762	7.61E-10	2.4E-08

**ENSG00000164483**	SAMD3	0.758402947	6.626448886	56.42091001	7.9E-10	2.49E-08
**ENSG00000179218**	CALR	0.496617987	7.347666707	53.86310938	8.27E-10	2.6E-08
**ENSG00000176595**	KBTBD11	-1.323768822	3.926504165	53.612586	8.83E-10	2.77E-08
**ENSG00000114529**	c3orf52	-1.385085797	4.018699133	53.59711267	8.87E-10	2.77E-08
**ENSG00000027697**	IFNGR1	0.759922349	5.531877558	53.55063022	8.98E-10	2.79E-08
**ENSG00000108946**	PRKAR1A	0.471078443	7.687835881	53.50151005	9.09E-10	2.82E-08
**ENSG00000145819**	ARHGAP26	0.669282145	5.962693534	53.48817766	9.13E-10	2.82E-08
**ENSG00000116489**	CAPZA1	0.427727205	8.197167286	53.46498257	9.18E-10	2.83E-08
**ENSG00000108175**	ZMIZ1	0.685933436	5.675924848	53.41670563	9.3E-10	2.86E-08
**ENSG00000123908**	AGO2	0.415386784	7.765613124	53.13962436	1E-09	3.07E-08
**ENSG00000175274**	TP53I11	0.994303155	3.983428006	53.06812193	1.02E-09	3.12E-08
**ENSG00000092964**	DPYSL2	0.848573232	5.507106828	52.92055365	1.06E-09	3.23E-08
**ENSG00000165792**	METTL17	-1.018081663	5.187220189	53.51807541	1.15E-09	3.51E-08
**ENSG00000107263**	RAPGEF1	0.458722551	7.308506338	52.57593324	1.16E-09	3.52E-08
**ENSG00000169813**	HNRNPF	0.53262708	7.267691275	52.55491159	1.17E-09	3.53E-08
**ENSG00000068796**	KIF2A	0.444142466	7.966477606	52.51704675	1.18E-09	3.55E-08
**ENSG00000187824**	TMEM220	-1.003124641	4.880941123	52.51029775	1.18E-09	3.55E-08
**ENSG00000168214**	RBPJ	0.490150666	7.00938428	52.43567928	1.21E-09	3.61E-08
**ENSG00000104419**	NDRG1	0.63711643	6.685876547	52.35824836	1.23E-09	3.68E-08
**ENSG00000116679**	IVNS1ABP	-0.459298048	7.911379321	52.23775409	1.27E-09	3.79E-08
**ENSG00000171522**	PTGER4	0.57972144	6.609956625	52.22541215	1.28E-09	3.79E-08
**ENSG00000047634**	SCML1	-1.595266409	4.708387466	58.73907375	1.29E-09	3.83E-08
**ENSG00000119772**	DNMT3A	-0.55706388	6.469873974	52.16247616	1.3E-09	3.83E-08
**ENSG00000175073**	VCPIP1	0.511618585	7.556471892	51.99836025	1.36E-09	3.99E-08
**ENSG00000108848**	LUC7L3	-1.052236179	8.677139474	63.87190839	1.43E-09	4.19E-08
**ENSG00000124193**	SRSF6	-0.537967895	7.815342502	51.76557233	1.44E-09	4.22E-08
**ENSG00000145287**	PLAC8	-0.589445379	7.803865618	53.0301801	1.47E-09	4.31E-08
**ENSG00000155158**	TTC39B	-0.555723418	6.867610436	51.48050166	1.56E-09	4.53E-08
**ENSG00000184743**	ATL3	0.491839733	7.142413201	51.32676929	1.62E-09	4.71E-08
**ENSG00000131149**	GSE1	0.738440071	5.608278616	51.10703247	1.72E-09	4.99E-08
**ENSG00000174718**	KIAA1551	0.37533854	10.15760417	51.06056568	1.74E-09	5.04E-08
**ENSG00000187239**	FNBP1	0.377839766	8.652477188	50.97582031	1.78E-09	5.14E-08
**ENSG00000108582**	CPD	0.532025279	6.381043573	50.9485765	1.8E-09	5.16E-08
**ENSG00000117360**	PRPF3	-0.712412961	6.384642305	51.75024958	1.83E-09	5.23E-08
**ENSG00000117616**	RSRP1	-0.841696746	7.981744912	59.98574158	1.84E-09	5.27E-08
**ENSG00000182796**	TMEM198B	-1.626506316	4.445732249	56.04014217	1.91E-09	5.45E-08
**ENSG00000124222**	STX16	-0.848614837	7.4502298	58.88835041	1.96E-09	5.54E-08

**ENSG00000166446**	CDYL2	0.987139508	4.500012143	50.63419379	1.96E-09	5.54E-08
**ENSG00000137076**	MIR6852	0.516144978	8.916250268	50.41932437	2.07E-09	5.86E-08
**ENSG00000184007**	PTP4A2	0.455754414	8.590064398	50.29827179	2.14E-09	6.04E-08
**ENSG00000204054**		1.071368281	4.401881098	50.24146494	2.18E-09	6.12E-08
**ENSG00000113384**	GOLPH3	0.645833908	6.311340897	50.17898899	2.21E-09	6.2E-08
**ENSG00000183864**	tob2	0.678854771	5.571615506	50.15303668	2.23E-09	6.23E-08
**ENSG00000099204**	ABLIM1	-0.521605207	8.693859481	50.01393343	2.31E-09	6.45E-08
**ENSG00000113263**	ITK	-0.408746441	9.128637372	49.78439316	2.46E-09	6.85E-08
**ENSG00000077238**	IL4R	-0.631960195	6.297015802	49.74226815	2.49E-09	6.91E-08
**ENSG00000124795**	DEK	0.576588108	7.915677872	51.03563497	2.55E-09	7.05E-08
**ENSG00000023171**	GRAMD1B	1.142821372	4.21783278	49.62882128	2.57E-09	7.09E-08
**ENSG00000197912**	SPG7	-0.672675295	5.907167756	49.54648123	2.63E-09	7.24E-08
**ENSG00000162241**	SLC25A45	-1.168041569	4.775618681	50.90381375	2.66E-09	7.29E-08
**ENSG00000152127**	MGAT5	0.571244839	6.986153834	49.40821707	2.73E-09	7.48E-08
**ENSG00000052126**	PLEKHA5	1.020489405	4.630063483	49.30733798	2.81E-09	7.67E-08
**ENSG00000107643**	MAPK8	-0.524667126	5.836461796	49.25134968	2.85E-09	7.76E-08
**ENSG00000136450**	SRSF1	-0.399606746	7.728080726	49.14438203	2.94E-09	7.98E-08
**ENSG00000166532**	RIMKLB	-1.011392179	4.769060746	49.13470655	2.94E-09	7.98E-08
**ENSG00000274627**		-0.995607168	5.118495074	49.84604788	2.96E-09	8E-08
**ENSG00000060339**	CCAR1	-0.413765719	7.589961347	49.07253092	2.99E-09	8.07E-08
**ENSG00000156675**	RAB11FIP1	0.827599956	6.047981072	51.61056565	3.02E-09	8.11E-08
**ENSG00000107679**	PLEKHA1	-0.544675618	7.110198653	49.03453723	3.03E-09	8.11E-08
**ENSG00000111276**	CDKN1B	0.472722373	7.694061224	49.0141739	3.04E-09	8.14E-08
**ENSG00000135473**	PAN2	-1.334858482	5.681065806	57.83350633	3.17E-09	8.47E-08
**ENSG00000139746**	RBM26	-0.517472376	8.060257651	48.72377099	3.3E-09	8.77E-08
**ENSG00000133935**	C14orf1	0.948273873	4.654059069	48.5974427	3.41E-09	9.04E-08
**ENSG00000122435**	TRMT13	-0.88052961	5.947626826	51.67626381	3.42E-09	9.04E-08
**ENSG00000206503**	hla-a	0.482719236	9.376646075	48.57427596	3.44E-09	9.07E-08
**ENSG00000150938**	CRIM1	0.993821171	4.710556763	48.53228754	3.48E-09	9.16E-08
**ENSG00000244879**	MIR4712	-0.963244124	7.009540605	57.11411215	3.59E-09	9.42E-08
**ENSG00000125124**	BBS2	-0.588845791	6.101241022	48.32314424	3.68E-09	9.65E-08
**ENSG00000196126**	HLA-DRB1	1.368167024	3.990995904	48.51078444	3.7E-09	9.67E-08
**ENSG00000145687**	SSBP2	-0.732051131	5.972260284	48.27611019	3.73E-09	9.73E-08
**ENSG00000103064**	SLC7A6	-0.51968091	7.850471111	48.26619537	3.74E-09	9.73E-08
**ENSG00000174136**	RGMB	-1.06135673	4.613913253	48.22648481	3.78E-09	9.82E-08
**ENSG00000134709**	HOOK1	-0.816826234	5.376149327	48.20991243	3.8E-09	9.84E-08
**ENSG00000147138**	GPR174	0.643975131	6.005346953	48.1570706	3.86E-09	9.96E-08

**ENSG00000142546**	NOSIP	-0.629533077	6.488180065	48.12501581	3.89E-09	1E-07
**ENSG00000105851**	PIK3CG	0.549259029	6.502439601	48.09087064	3.93E-09	1.01E-07
**ENSG00000114541**	FRMD4B	0.848778171	5.140224948	48.05916474	3.96E-09	1.02E-07
**ENSG00000235162**	C12orf75	1.073037385	4.652508469	48.02394683	4E-09	1.02E-07
**ENSG00000117505**	DR1	0.427001756	7.647828799	48.02332481	4E-09	1.02E-07
**ENSG00000111237**	VPS29	-0.643616285	6.068082842	47.99458178	4.03E-09	1.03E-07
**ENSG00000186815**	TPCN1	-0.796450593	5.859447876	48.86486969	4.06E-09	1.03E-07
**ENSG00000163636**	PSMD6	-0.629026483	7.066282425	49.18197471	4.17E-09	1.06E-07
**ENSG00000135164**	DMTF1	-0.585418819	7.750322382	49.63127192	4.2E-09	1.06E-07
**ENSG00000175727**	MLXIP	-0.543914692	6.912429229	47.74613225	4.32E-09	1.09E-07
**ENSG00000147443**	dok2	0.839554897	5.019881978	47.73951503	4.33E-09	1.09E-07
**ENSG00000124813**	RUNX2	0.646414569	6.70571195	48.32261622	4.42E-09	1.11E-07
**ENSG00000073861**	TBX21	1.806052223	4.068930932	52.59130646	4.61E-09	1.15E-07
**ENSG00000171488**	LRRC8C	0.448001447	8.133097104	47.45333918	4.69E-09	1.17E-07
**ENSG00000138380**	CARF	-0.840768367	5.493031802	47.42745853	4.73E-09	1.18E-07
**ENSG00000162804**	sned1	-0.750263012	5.667925803	47.23368263	4.99E-09	1.24E-07
**ENSG00000152256**	PDK1	-1.095676002	6.983910057	57.01009524	5.09E-09	1.26E-07
**ENSG00000143815**	LBR	0.43340091	7.588922821	47.14356821	5.12E-09	1.26E-07
**ENSG00000138439**	FAM117B	-0.826706029	6.462433518	51.41591404	5.14E-09	1.27E-07
**ENSG00000235194**	PPP1R3E	-0.931043179	5.541973631	49.15285867	5.21E-09	1.28E-07
**ENSG00000143889**	HNRNPLL	0.68367805	6.68054926	48.69390439	5.23E-09	1.28E-07
**ENSG00000107611**	CUBN	-1.021429746	5.360698263	49.59255815	5.25E-09	1.28E-07
**ENSG00000223501**	VPS52	-0.783505343	5.592304462	47.03649857	5.27E-09	1.29E-07
**ENSG00000144567**	FAM134A	0.510247876	6.054759797	46.95723673	5.39E-09	1.31E-07
**ENSG00000122085**	MTERF4	-0.524009349	6.642533106	46.95017776	5.4E-09	1.31E-07
**ENSG00000168675**	LDLRAD4	0.541903986	6.416460456	46.89916969	5.48E-09	1.33E-07
**ENSG00000167280**	ENGASE	-1.177185577	4.929531553	49.40222065	5.63E-09	1.36E-07
**ENSG00000196295**		-0.640770489	6.975547607	48.29241688	5.77E-09	1.39E-07
**ENSG00000034677**	RNF19A	0.440841811	7.200329187	46.61931711	5.93E-09	1.43E-07
**ENSG00000138413**	idh1	1.12506203	4.425269896	46.60027128	5.96E-09	1.43E-07
**ENSG00000255733**	IFNG-AS1	1.200287642	3.9536274	46.58384764	5.99E-09	1.44E-07
**ENSG00000162889**	MAPKAPK2	0.604430092	6.004148805	46.53363992	6.07E-09	1.45E-07
**ENSG00000146112**	PPP1R18	0.665140201	6.91405471	48.58198263	6.07E-09	1.45E-07
**ENSG00000140396**	NCOA2	0.427343453	7.370760078	46.52535545	6.09E-09	1.45E-07
**ENSG00000254870**	ATP6V1G2-DDX	-0.672660736	7.657772058	50.71860032	6.18E-09	1.47E-07
**ENSG00000164733**	CTSB	0.53973848	6.694409617	46.40762072	6.29E-09	1.49E-07
**ENSG00000134684**	YARS	0.646781591	5.511385132	46.36777701	6.36E-09	1.51E-07

**ENSG00000051825**	MPHOSPH9	-0.525607081	6.869218254	46.30327517	6.48E-09	1.53E-07
**ENSG00000095794**	CREM	1.023360039	4.216029151	46.27132122	6.54E-09	1.54E-07
**ENSG00000123612**	ACVR1C	-1.077374847	4.401129124	46.24472392	6.59E-09	1.55E-07
**ENSG00000118971**	CCND2	0.442111386	9.073366824	46.16208879	6.74E-09	1.58E-07
**ENSG00000033867**	SLC4A7	0.400558058	7.801927394	46.13165053	6.8E-09	1.59E-07
**ENSG00000068831**	RASGRP2	-0.955661848	7.178985956	54.75619691	6.91E-09	1.61E-07
**ENSG00000083844**	ZNF264	0.582699425	7.01839641	46.26513199	7.33E-09	1.71E-07
**ENSG00000184787**	UBE2G2	-0.803099155	7.122642644	51.90364094	7.35E-09	1.71E-07
**ENSG00000263798**		-0.960529009	5.939325489	50.52743364	7.66E-09	1.78E-07
**ENSG00000156504**	FAM122B	-0.649535089	6.017254609	45.61367887	7.88E-09	1.82E-07
**ENSG00000162613**	FUBP1	-0.378988555	8.12553972	45.60068061	7.91E-09	1.83E-07
**ENSG00000078589**	p2ry10	0.607703209	6.186376035	45.50152633	8.13E-09	1.87E-07
**ENSG00000125753**	VASP	0.674708595	5.249263493	45.49748401	8.14E-09	1.87E-07
**ENSG00000148730**	EIF4EBP2	0.446017049	7.737117398	45.49035793	8.16E-09	1.87E-07
**ENSG00000131236**	CAP1	0.406479007	8.187783657	45.36920406	8.45E-09	1.93E-07
**ENSG00000127022**	CANX	0.474202088	8.433112658	45.30996116	8.59E-09	1.96E-07
**ENSG00000172766**	NAA16	-0.654751586	6.591926579	46.02688188	8.65E-09	1.97E-07
**ENSG00000181472**	ZBTB2	0.589456656	5.45690172	45.2075316	8.85E-09	2.01E-07
**ENSG00000130414**	NDUFA10	-0.577476846	6.136673677	45.16852256	8.94E-09	2.03E-07
**ENSG00000249786**		0.807796913	4.681027883	45.09248	9.14E-09	2.07E-07
**ENSG00000157933**	SKI	0.520552843	7.070815457	45.06073954	9.22E-09	2.08E-07
**ENSG00000163599**	CTLA4	0.943230249	5.105055965	45.50263064	9.28E-09	2.09E-07
**ENSG00000138821**	slc39a8	0.744796945	5.921816162	45.65624442	9.48E-09	2.13E-07
**ENSG00000125384**	PTGER2	0.878252605	6.666199273	50.83927938	9.65E-09	2.17E-07
**ENSG00000141458**	NPC1	0.569736455	6.529241643	44.89310157	9.68E-09	2.17E-07
**ENSG00000213047**	DENND1B	0.505164574	6.448787152	44.76369861	1E-08	2.24E-07
**ENSG00000068366**	ACSL4	0.665808809	5.984520006	44.72826713	1.01E-08	2.26E-07
**ENSG00000180357**	ZNF609	-0.424284934	7.229176263	44.69537735	1.02E-08	2.28E-07
**ENSG00000137078**	SIT 1.00	1.032783774	4.628494055	44.67149391	1.03E-08	2.29E-07
**ENSG00000164574**	GALNT10	0.645082834	5.723394644	44.59275868	1.05E-08	2.34E-07
**ENSG00000068885**	IFT80	-0.817349882	5.755973272	45.69798747	1.06E-08	2.35E-07
**ENSG00000238197**	PAXBP1-AS1	-1.104739365	4.16575692	44.39163304	1.12E-08	2.47E-07
**ENSG00000144824**	PHLDB2	1.137884808	4.472953256	44.49587345	1.12E-08	2.47E-07
**ENSG00000182158**	CREB3L2	0.570731007	6.383046394	44.25357955	1.16E-08	2.56E-07
**ENSG00000122224**	LY9	-0.448851696	6.701443846	44.21396595	1.18E-08	2.58E-07
**ENSG00000118816**	CCNI	0.487694958	9.034760619	44.20369229	1.18E-08	2.58E-07
**ENSG00000254995**	STX16-NPEPL1	-0.904281403	6.502532526	50.01817813	1.2E-08	2.62E-07

**ENSG00000089775**	ZBTB25	-0.434011048	7.740760897	44.06223604	1.23E-08	2.68E-07
**ENSG00000151914**	DST	-1.378647912	4.955039246	48.76957185	1.24E-08	2.7E-07
**ENSG00000168071**	CCDC88B	-0.787998014	5.726066691	44.70068907	1.25E-08	2.71E-07
**ENSG00000105329**	TGFB1	0.474656198	7.655920772	43.94024885	1.27E-08	2.76E-07
**ENSG00000134453**	RBM17	-0.554026328	6.63462865	43.89324404	1.29E-08	2.79E-07
**ENSG00000188636**	LDOC1L	0.936789222	4.545706092	43.83515817	1.31E-08	2.83E-07
**ENSG00000184009**	ACTG1	0.566862936	9.796667662	45.44138734	1.34E-08	2.88E-07
**ENSG00000137449**	CPEB2	0.706256927	5.461884083	43.754692	1.34E-08	2.89E-07
**ENSG00000153914**	SREK1	-0.549957384	7.438462546	44.44704016	1.35E-08	2.89E-07
**ENSG00000099942**	CRKL	0.477852482	6.99516584	43.73616532	1.35E-08	2.89E-07
**ENSG00000114812**	VIPR1	-0.744850416	5.361871096	43.58609898	1.41E-08	3.01E-07
**ENSG00000166326**	TRIM44	-0.479905334	6.91333891	43.49485608	1.45E-08	3.09E-07
**ENSG00000187726**	DNAJB13	-1.163144988	3.837113717	43.41416495	1.48E-08	3.15E-07
**ENSG00000077782**	FGFR1	1.056231635	4.053806571	43.40346169	1.49E-08	3.16E-07
**ENSG00000132294**	EFR3A	0.401598058	7.036601075	43.33435182	1.52E-08	3.21E-07
**ENSG00000106133**	NSUN5P2	-0.937452566	4.951080104	43.33096831	1.52E-08	3.21E-07
**ENSG00000196821**	c6orf106	0.569745306	6.034394386	43.31850129	1.53E-08	3.22E-07
**ENSG00000105879**	CBLL1	0.504448665	7.101314593	43.30636449	1.53E-08	3.22E-07
**ENSG00000163565**	IFI16	0.427552913	7.63741245	43.30061921	1.53E-08	3.22E-07
**ENSG00000228784**	LINC00954	-1.167890949	4.693815989	45.01067175	1.56E-08	3.26E-07
**ENSG00000168172**	HOOK3	0.468423495	7.229188579	43.2362056	1.56E-08	3.27E-07
**ENSG00000162630**	B3GALT2	0.786909236	4.818928434	43.18867332	1.58E-08	3.31E-07
**ENSG00000091039**	OSBPL8	0.452932446	8.536736276	43.07856411	1.64E-08	3.41E-07
**ENSG00000121413**	ZSCAN18	-1.10726511	4.518797827	43.03433619	1.66E-08	3.44E-07
**ENSG00000140030**	GPR65	0.826330862	6.27396962	46.92995219	1.66E-08	3.44E-07
**ENSG00000269713**	NBPF9	-0.701070806	5.116331933	43.00481475	1.67E-08	3.46E-07
**ENSG00000198826**	ARHGAP11A	1.093820898	3.989678141	42.92463276	1.71E-08	3.54E-07
**ENSG00000097033**	SH3GLB1	0.440132412	6.957505038	42.8954713	1.73E-08	3.56E-07
**ENSG00000004399**	PLXND1	1.567748003	4.726098262	49.36976963	1.73E-08	3.56E-07
**ENSG00000159496**	RGL4	-0.873419302	4.74008453	42.83497103	1.76E-08	3.61E-07
**ENSG00000168016**	TRANK1	0.415806352	7.902347801	42.82174749	1.76E-08	3.61E-07
**ENSG00000197077**	KIAA1671	1.176419786	4.050456302	42.8159685	1.77E-08	3.61E-07
**ENSG00000277734**		0.485029889	9.291363113	42.81490402	1.77E-08	3.61E-07
**ENSG00000266086**		-0.595492671	5.602058257	42.78605283	1.78E-08	3.63E-07
**ENSG00000100346**	CACNA1I	-0.923556628	5.596910621	45.34268333	1.81E-08	3.67E-07
**ENSG00000255026**		-1.759159314	4.394988043	48.24503537	1.81E-08	3.67E-07
**ENSG00000110108**	TMEM109	0.762606795	5.080816517	42.72989355	1.81E-08	3.67E-07

**ENSG00000006576**	PHTF2	0.489503677	6.851860488	42.65106861	1.85E-08	3.75E-07
**ENSG00000168209**	DDIT4	1.144946441	4.789477078	44.59235993	1.89E-08	3.81E-07
**ENSG00000196924**	FLNA	0.544396544	9.878377069	43.72774341	1.91E-08	3.85E-07
**ENSG00000240038**	AMY2B	-1.20546211	5.567021943	48.850898	1.94E-08	3.91E-07
**ENSG00000166501**	PRKCB	0.479546758	7.876442104	42.43046478	1.98E-08	3.97E-07
**ENSG00000213015**	ZNF580	-1.080941252	4.445051255	42.42636643	1.98E-08	3.97E-07
**ENSG00000152969**	JAKMIP1	1.09552221	3.732746592	42.41848534	1.98E-08	3.97E-07
**ENSG00000116754**	SRSF11	-0.761392773	8.586279116	48.99282686	2.02E-08	4.03E-07
**ENSG00000102572**	STK24	0.379701851	7.435160336	42.29118729	2.06E-08	4.1E-07
**ENSG00000100596**	SPTLC2	0.559059297	6.267043579	42.24347205	2.09E-08	4.14E-07
**ENSG00000119707**	RBM25	-0.524336768	8.593363599	43.29886153	2.09E-08	4.14E-07
**ENSG00000272849**		-1.276115274	4.44687814	44.06561533	2.09E-08	4.14E-07
**ENSG00000117676**	RPS6KA1	0.578209313	5.97248923	42.08817538	2.19E-08	4.32E-07
**ENSG00000184371**	CSF1	1.09029626	4.219094517	42.05196215	2.21E-08	4.36E-07
**ENSG00000175265**	GOLGA8A	-1.665553027	6.998243282	50.59955515	2.21E-08	4.36E-07
**ENSG00000091527**	CDV3	0.428748388	8.061415053	42.01098244	2.24E-08	4.4E-07
**ENSG00000166046**	TCP11L2	-0.529318062	6.582514416	41.95457768	2.28E-08	4.46E-07
**ENSG00000182162**	P2RY8	0.489581697	7.376889357	41.9454703	2.28E-08	4.47E-07
**ENSG00000223745**		-1.209230658	6.035221924	49.51439911	2.34E-08	4.58E-07
**ENSG00000116191**	RALGPS2	-1.138434535	4.601837123	42.82446934	2.42E-08	4.73E-07
**ENSG00000010244**	Mir632	-0.402330654	8.857938931	41.7080339	2.45E-08	4.77E-07
**ENSG00000075624**	ACTB	0.506256738	11.77562199	41.6692327	2.48E-08	4.81E-07
**ENSG00000106780**	MEGF9	0.55045166	5.801787763	41.64594166	2.49E-08	4.84E-07
**ENSG00000128294**	TPST2	0.74468133	5.001277212	41.59302008	2.53E-08	4.9E-07
**ENSG00000154642**	c21orf91	0.554832843	6.938724865	41.64394413	2.57E-08	4.96E-07
**ENSG00000106799**	TGFBR1	0.457568911	6.888934444	41.49886303	2.6E-08	5.03E-07
**ENSG00000148158**	SNX30	0.687068529	5.583567688	41.43850219	2.65E-08	5.1E-07
**ENSG00000166913**	YWHAB	0.387134931	8.694845629	41.43587055	2.65E-08	5.1E-07
**ENSG00000165476**	REEP3	0.700977288	5.974256614	41.92748926	2.71E-08	5.19E-07
**ENSG00000019582**	CD74	0.56450154	7.130045725	42.08757674	2.79E-08	5.35E-07
**ENSG00000101104**	PABPC1L	-1.446949579	4.84838561	46.79117536	2.86E-08	5.46E-07
**ENSG00000110324**	IL10RA	0.493356907	8.419764943	41.59719923	2.86E-08	5.46E-07
**ENSG00000155307**	SAMSN1	0.621518904	6.07139531	41.17815597	2.87E-08	5.46E-07
**ENSG00000115524**	SF3B1	-0.648484053	9.651355229	45.14325865	2.89E-08	5.49E-07
**ENSG00000011275**	RNF216	-0.409082045	6.936436688	41.11988744	2.92E-08	5.52E-07
**ENSG00000144597**	EAF1	0.601398937	5.659247965	41.11923461	2.92E-08	5.52E-07
**ENSG00000101901**	ALG13	-0.582672018	6.684821281	41.07585735	2.98E-08	5.63E-07

**ENSG00000171552**	BCL2L1	0.801043626	4.469105566	41.04404769	2.98E-08	5.63E-07
**ENSG00000111605**	CPSF6	-0.470238989	7.69516193	40.97537354	3.04E-08	5.74E-07
**ENSG00000147010**	SH3KBP1	0.357605173	7.712345022	40.96019395	3.06E-08	5.74E-07
**ENSG00000237499**	LOC100130476	-0.968460041	4.668756394	40.95998928	3.06E-08	5.74E-07
**ENSG00000126453**	BCL2L12	-0.958495247	4.323013181	40.92699468	3.09E-08	5.79E-07
**ENSG00000175567**	UCP2	-0.454748923	7.878764873	40.87810002	3.13E-08	5.86E-07
**ENSG00000147649**	MTDH	0.413440867	7.534303036	40.78760465	3.22E-08	6.01E-07
**ENSG00000090621**	PABPC4	-0.418676121	6.999686648	40.58365405	3.42E-08	6.38E-07
**ENSG00000165071**	TMEM71	-0.542424886	6.410677817	40.54776698	3.46E-08	6.44E-07
**ENSG00000136238**	RAC1	0.482279386	6.405570132	40.51984733	3.49E-08	6.48E-07
**ENSG00000138640**	FAM13A	-0.749949319	6.461380522	43.28742912	3.52E-08	6.53E-07
**ENSG00000121310**	ECHDC2	-0.926875811	5.832953205	44.30166717	3.58E-08	6.62E-07
**ENSG00000164691**	TAGAP	-0.42577886	8.486190331	40.38408911	3.63E-08	6.71E-07
**ENSG00000184956**	MUC6	-1.341866587	4.036783132	41.41322432	3.68E-08	6.79E-07
**ENSG00000090104**	RGS1	1.526802846	5.354542275	47.68419985	3.72E-08	6.84E-07
**ENSG00000102007**	PLP2	0.745601898	6.521759769	43.41787971	3.75E-08	6.9E-07
**ENSG00000103061**	SLC7A6OS	-0.476238712	7.31345319	40.19302939	3.85E-08	7.06E-07
**ENSG00000113593**	ppwd1	-0.505375172	6.890812234	40.17478461	3.87E-08	7.09E-07
**ENSG00000131238**	PPT1	0.38747502	7.736571098	40.15497535	3.89E-08	7.12E-07
**ENSG00000231389**	HLA-DPA1	1.10326253	4.909509106	42.69336376	3.93E-08	7.18E-07
**ENSG00000224078**		-0.505682364	7.513027153	40.38281541	3.94E-08	7.18E-07
**ENSG00000166128**	RAB8B	0.461883018	7.414860827	40.08852684	3.97E-08	7.22E-07
**ENSG00000227372**	TP73-AS1	-0.941236814	4.432151126	40.07311534	3.99E-08	7.24E-07
**ENSG00000170340**	B3GNT2	0.641738105	5.04735941	40.01287039	4.06E-08	7.36E-07
**ENSG00000139514**	SLC7A1	0.636330977	5.619040215	40.00342929	4.07E-08	7.37E-07
**ENSG00000120437**	ACAT2	0.74446685	5.035379164	39.99364918	4.09E-08	7.38E-07
**ENSG00000204789**		-1.062423378	3.953087241	39.94380464	4.15E-08	7.47E-07
**ENSG00000096746**	HNRNPH3	-0.351566727	7.88214844	39.94213792	4.15E-08	7.47E-07
**ENSG00000158411**	MITD1	-0.636708104	5.656656984	39.85198314	4.27E-08	7.66E-07
**ENSG00000139289**	PHLDA1	0.979291629	4.266137903	39.77926501	4.36E-08	7.82E-07
**ENSG00000107736**	CDH23	-1.259196717	4.441462992	41.62028122	4.43E-08	7.93E-07
**ENSG00000261553**		-0.756686472	4.700827353	39.71768223	4.44E-08	7.94E-07
**ENSG00000232810**	TNF	1.510697775	4.111093442	42.84001802	4.51E-08	8.04E-07
**ENSG00000084733**	RAB10	0.468648713	6.740730775	39.63864403	4.55E-08	8.1E-07
**ENSG00000180776**	ZDHHC20	0.472532185	7.251243292	39.53536444	4.69E-08	8.35E-07
**ENSG00000143870**	PDIA6	0.614426312	6.215023382	39.51007258	4.73E-08	8.4E-07
**ENSG00000132680**	KIAA0907	-0.704424517	6.427350201	41.54523561	4.8E-08	8.5E-07

**ENSG00000107771**	CCSER2	0.373421412	7.977963575	39.41403035	4.87E-08	8.62E-07
**ENSG00000162892**	IL24	-0.624821568	6.198682925	39.40609347	4.89E-08	8.64E-07
**ENSG00000134186**	PRPF38B	-0.421318232	8.010777935	39.31111181	5.03E-08	8.86E-07
**ENSG00000162676**	GFI1	1.04173351	4.106819293	39.27975004	5.07E-08	8.93E-07
**ENSG00000234771**		-0.843537958	5.127428644	39.1976684	5.2E-08	9.14E-07
**ENSG00000136527**	TRA2B	-0.421490537	8.420564501	39.16123887	5.26E-08	9.23E-07
**ENSG00000139182**	clstn3	0.716437209	5.47653842	39.14789601	5.28E-08	9.25E-07
**ENSG00000138449**	SLC40A1	-0.704744732	6.130472252	40.22889036	5.36E-08	9.37E-07
**ENSG00000026103**	FAS	0.620134559	6.027705728	39.07255224	5.4E-08	9.43E-07
**ENSG00000138071**	ACTR2	0.377282399	8.905768459	39.06632208	5.41E-08	9.44E-07
**ENSG00000003400**	CASP10	-0.689369278	6.002197366	39.62453237	5.48E-08	9.53E-07
**ENSG00000162734**	PEA15	0.799251734	4.37118764	38.99599229	5.53E-08	9.61E-07
**ENSG00000183891**	ttc32	-1.013061115	4.069518773	38.96242142	5.59E-08	9.69E-07
**ENSG00000089335**	ZNF302	-0.605336412	5.612914362	38.92573261	5.65E-08	9.78E-07
**ENSG00000215252**	GOLGA8B	-1.586799875	7.235499508	46.2836961	5.68E-08	9.83E-07
**ENSG00000223509**		-0.938251645	4.70793672	38.84110778	5.8E-08	1E-06
**ENSG00000155926**	SLA	0.425938337	7.394016638	38.80270007	5.87E-08	1.01E-06
**ENSG00000121579**	NAA50	0.454333487	6.437359592	38.77573419	5.92E-08	1.02E-06
**ENSG00000113810**	SMC4	0.525933294	6.939280365	38.68742902	6.08E-08	1.04E-06
**ENSG00000158321**	AUTS2	1.062109492	6.077360438	44.75095588	6.1E-08	1.05E-06
**ENSG00000166483**	WEE1	1.154098146	4.186163382	38.79729431	6.12E-08	1.05E-06
**ENSG00000069702**	TGFBR3	0.864364759	6.094125089	42.60590087	6.26E-08	1.07E-06
**ENSG00000171115**	GIMAP8	-0.494385955	6.221781616	38.58367996	6.27E-08	1.07E-06
**ENSG00000196230**	TUBB	0.509405593	7.512598508	39.10756438	6.33E-08	1.08E-06
**ENSG00000102699**	PARP4	0.3998985	7.758745764	38.50775108	6.42E-08	1.09E-06
**ENSG00000128699**	ORMDL1	-0.744414342	6.682958563	41.90203213	6.55E-08	1.11E-06
**ENSG00000023909**	GCLM	0.682773912	5.220667285	38.40954133	6.62E-08	1.12E-06
**ENSG00000100100**	PIK3IP1	-0.422032467	7.594423885	38.39646471	6.64E-08	1.12E-06
**ENSG00000178038**	ALS2CL	-1.569322597	4.704082675	43.93572378	6.69E-08	1.13E-06
**ENSG00000065357**	DGKA	-0.556555527	9.209420664	40.59796374	6.72E-08	1.13E-06
**ENSG00000106477**	CEP41	-1.154239201	3.770121741	38.26905934	6.91E-08	1.16E-06
**ENSG00000173209**	AHSA2	-0.812406799	7.802763869	44.56351676	6.92E-08	1.16E-06
**ENSG00000253352**		-0.423296643	8.367710068	38.26036918	6.93E-08	1.16E-06
**ENSG00000133460**	SLC2A11	-1.120539846	4.200970384	38.25524041	6.94E-08	1.16E-06
**ENSG00000035115**	SH3YL1	-0.586860398	6.121822344	38.24554088	6.96E-08	1.16E-06
**ENSG00000138600**	SPPL2A	0.520645885	6.309508516	38.13795628	7.19E-08	1.2E-06
**ENSG00000248019**	FAM13A-AS1	-0.726180661	5.556385232	38.13187212	7.21E-08	1.2E-06

**ENSG00000135362**	PRR5L	1.171496693	4.951089538	41.44782713	7.24E-08	1.21E-06
**ENSG00000189319**	FAM53B	0.539235594	6.073756931	38.10039861	7.28E-08	1.21E-06
**ENSG00000135269**	TES	0.410910896	7.361756165	38.05148912	7.39E-08	1.23E-06
**ENSG00000072786**	STK10	0.323604698	8.504141371	38.04673286	7.4E-08	1.23E-06
**ENSG00000172795**	DCP2	0.364866165	7.5349972	37.9979771	7.51E-08	1.24E-06
**ENSG00000170004**	CHD3	-0.365816866	8.650846373	37.94424644	7.64E-08	1.26E-06
**ENSG00000078369**	GNB1	0.372275819	7.960886625	37.9304136	7.67E-08	1.26E-06
**ENSG00000227617**	CERS6-AS1	-1.062198331	4.373583553	37.92733715	7.68E-08	1.26E-06
**ENSG00000154144**	TBRG1	-0.684366689	6.097272507	38.83033445	7.77E-08	1.28E-06
**ENSG00000263072**		-0.919765133	4.300306164	37.88559285	7.78E-08	1.28E-06
**ENSG00000136802**	LRRC8A	0.55635218	5.21767101	37.86884878	7.82E-08	1.28E-06
**ENSG00000112242**	E2F3	0.780463689	4.826904703	37.86536612	7.83E-08	1.28E-06
**ENSG00000157193**	LRP8	0.778635109	4.561249126	37.78504761	8.02E-08	1.31E-06
**ENSG00000003756**	RBM5	-0.559862523	8.148577657	40.08101463	8.1E-08	1.32E-06
**ENSG00000227766**		0.62467105	7.595656585	41.108638	8.14E-08	1.33E-06
**ENSG00000284368**		-1.115878063	5.6976834	43.03084651	8.26E-08	1.34E-06
**ENSG00000198563**	DDX39B	-0.79892685	8.438602708	43.84666871	8.28E-08	1.34E-06
**ENSG00000112146**	FBXO9	-0.555264719	6.12098904	37.66992078	8.31E-08	1.35E-06
**ENSG00000100836**	PABPN1	-0.456936239	6.182544633	37.63871723	8.39E-08	1.35E-06
**ENSG00000047849**	MAP4	0.433503315	6.938193031	37.63511365	8.4E-08	1.35E-06
**ENSG00000122042**	UBL3	0.507139164	6.833420239	37.63464457	8.4E-08	1.35E-06
**ENSG00000165521**	EML5	-0.693245165	5.328791855	37.63430588	8.4E-08	1.35E-06
**ENSG00000198553**	KCNRG	0.973126669	3.924113494	37.60513623	8.48E-08	1.36E-06
**ENSG00000183735**	TBK1	0.65338744	5.395692187	37.54970021	8.63E-08	1.39E-06
**ENSG00000236287**	ZBED5	-0.44604196	7.065199789	37.54390336	8.64E-08	1.39E-06
**ENSG00000221944**	MIR5001	-0.93354065	4.476158351	37.34681555	9.19E-08	1.47E-06
**ENSG00000108797**	CNTNAP1	-1.363212921	4.084551276	38.84210804	9.41E-08	1.5E-06
**ENSG00000073605**	GSDMB	-1.024929016	5.117288664	39.53301743	9.53E-08	1.52E-06
**ENSG00000111412**	c12orf49	0.562856577	5.45113341	37.21647797	9.57E-08	1.53E-06
**ENSG00000113108**	MIR6831	-1.249542785	3.971991061	37.46196945	9.66E-08	1.54E-06
**ENSG00000227218**		-0.55989368	5.350143538	37.10618323	9.9E-08	1.57E-06
**ENSG00000203667**	cox20	-0.688891733	7.170138825	40.91637732	9.93E-08	1.58E-06
**ENSG00000279088**		-1.157087039	3.817956136	37.04007202	1.01E-07	1.6E-06
**ENSG00000101040**	ZMYND8	-0.459187409	6.637313766	37.01947241	1.02E-07	1.61E-06
**ENSG00000188735**	TMEM120B	0.461615409	6.711053745	37.01241951	1.02E-07	1.61E-06
**ENSG00000132718**	SYT11	0.934049527	4.778018269	37.26220761	1.04E-07	1.65E-06
**ENSG00000245849**	RAD51-AS1	-0.978458202	4.37445231	36.90788573	1.05E-07	1.66E-06

**ENSG00000103653**	CSK	0.455250801	6.539665528	36.87123073	1.07E-07	1.68E-06
**ENSG00000146757**	ZNF92	0.607043345	5.916010532	36.84668934	1.07E-07	1.69E-06
**ENSG00000183337**	BCOR	0.399877276	6.623961126	36.82527054	1.08E-07	1.69E-06
**ENSG00000161912**	ADCY10P1	-1.078920944	4.780662084	38.46212834	1.08E-07	1.69E-06
**ENSG00000114353**	GNAI2	0.44323348	7.672168697	36.71640612	1.12E-07	1.75E-06
**ENSG00000178951**	ZBTB7A	0.569466236	6.529945486	36.88110935	1.12E-07	1.75E-06
**ENSG00000108465**	CDK5RAP3	-1.098581567	6.05913247	42.59124516	1.12E-07	1.75E-06
**ENSG00000271601**	LIX1L	0.543981484	7.208037191	37.6990271	1.13E-07	1.76E-06
**ENSG00000204681**	GABBR1	-1.467669725	6.035545174	43.22688698	1.13E-07	1.76E-06
**ENSG00000129351**	ILF3	-0.412991145	7.881396916	36.65136075	1.14E-07	1.77E-06
**ENSG00000102172**	SMS	0.726969596	4.769654159	36.64160308	1.14E-07	1.77E-06
**ENSG00000095564**	BTAF1	-0.613957454	7.735655835	39.91321177	1.15E-07	1.78E-06
**ENSG00000167792**	NDUFV1	-0.810117709	5.386715818	37.16529496	1.15E-07	1.78E-06
**ENSG00000198265**	HELZ	0.310460463	8.381334024	36.58129529	1.17E-07	1.8E-06
**ENSG00000247828**	TMEM161B-AS1	-0.868524847	5.037490678	36.80918974	1.17E-07	1.8E-06
**ENSG00000206190**	ATP10A	-0.6277835	5.827221607	36.57113793	1.17E-07	1.8E-06
**ENSG00000222041**	CYTOR	0.924096607	4.071217186	36.57072791	1.17E-07	1.8E-06
**ENSG00000125354**	38961	-0.363737167	8.565418415	36.56212818	1.17E-07	1.8E-06
**ENSG00000100201**	DDX17	-0.709022703	10.53317721	41.0121796	1.21E-07	1.84E-06
**ENSG00000108469**	RECQL5	-0.884233289	4.14957163	36.47351451	1.21E-07	1.84E-06
**ENSG00000164548**	TRA2A	-0.482570619	6.963660839	36.44888876	1.22E-07	1.86E-06
**ENSG00000134698**	AGO4	0.589993423	6.113092867	36.42688965	1.22E-07	1.87E-06
**ENSG00000102710**	SUPT20H	-0.378563481	7.297709365	36.35660322	1.25E-07	1.91E-06
**ENSG00000110958**	PTGES3	0.399585666	7.437602104	36.33510348	1.26E-07	1.92E-06
**ENSG00000112624**	GLTSCR1L	0.371346495	7.342026491	36.32654954	1.26E-07	1.92E-06
**ENSG00000198648**	STK39	0.564516603	5.931734287	36.31233545	1.27E-07	1.92E-06
**ENSG00000182541**	LIMK2	-0.526456769	6.674811446	36.2910875	1.28E-07	1.93E-06
**ENSG00000059588**	TARBP1	-0.786476551	6.011611701	38.82390862	1.28E-07	1.94E-06
**ENSG00000172270**	BSG	0.635709506	5.226701537	36.21409835	1.31E-07	1.98E-06
**ENSG00000125772**	GPCPD1	-0.544452607	7.121384786	37.09274705	1.32E-07	1.99E-06
**ENSG00000107742**	SPOCK2	0.39361278	9.010286122	36.16982197	1.33E-07	2E-06
**ENSG00000168234**	TTC39C	0.36377332	7.884764953	36.15599112	1.33E-07	2E-06
**ENSG00000150347**	ARID5B	0.380311964	7.792482655	36.15572953	1.33E-07	2E-06
**ENSG00000204282**	TNRC6C-AS1	-0.600106523	6.410407868	36.63053243	1.34E-07	2.01E-06
**ENSG00000067560**	RHOA	0.456785647	8.17447356	36.28066976	1.35E-07	2.02E-06
**ENSG00000184677**	ZBTB40	-0.597245822	6.812287679	37.4036207	1.35E-07	2.02E-06
**ENSG00000131473**	ACLY	0.443150888	6.389929819	36.01645028	1.39E-07	2.08E-06

**ENSG00000170571**	EMB	0.328585583	8.619786732	36.01356498	1.39E-07	2.08E-06
**ENSG00000182944**	EWSR1	-0.422242257	7.930043775	35.98048944	1.41E-07	2.1E-06
**ENSG00000185477**	GPRIN3	0.435168945	8.688959591	35.96289318	1.42E-07	2.1E-06
**ENSG00000136485**	DCAF7	0.442273123	6.87090019	35.91849699	1.44E-07	2.13E-06
**ENSG00000131778**	CHD1L	-0.731694709	5.076201345	35.89781351	1.45E-07	2.14E-06
**ENSG00000157259**	GATAD1	-0.853378408	4.989063497	35.9315417	1.48E-07	2.19E-06
**ENSG00000115896**	PLCL1	-0.703070917	5.557969127	35.76303698	1.51E-07	2.23E-06
**ENSG00000112782**	CLIC5	0.964942928	4.463097766	35.72339009	1.53E-07	2.25E-06
**ENSG00000284554**		0.614734875	5.358435683	35.57544139	1.6E-07	2.36E-06
**ENSG00000069667**	RORA	0.388889861	9.20596249	35.51863487	1.63E-07	2.4E-06
**ENSG00000221963**	APOL6	0.431553171	8.373980522	35.48992616	1.65E-07	2.42E-06
**ENSG00000025156**	HSF2	-0.630185639	5.274986295	35.44620483	1.67E-07	2.45E-06
**ENSG00000145476**	CYP4V2	-0.443857385	6.70911025	35.37288959	1.71E-07	2.5E-06
**ENSG00000189007**	ADAT2	-1.183084017	4.830814159	38.28424213	1.72E-07	2.52E-06
**ENSG00000108375**	RNF43	-0.584197359	5.360080968	35.33209078	1.73E-07	2.53E-06
**ENSG00000122566**	HNRNPA2B1	-0.383423032	9.907385933	35.3285575	1.73E-07	2.53E-06
**ENSG00000108773**	KAT2A	-1.014455447	4.652512577	35.87713208	1.75E-07	2.55E-06
**ENSG00000101290**	CDS2	0.356783357	6.876496285	35.2669063	1.77E-07	2.57E-06
**ENSG00000168066**	SF1	-0.4116049	8.561245687	35.24151718	1.78E-07	2.59E-06
**ENSG00000266028**	SRGAP2	0.837091134	4.685856056	35.21413516	1.8E-07	2.61E-06
**ENSG00000070882**	OSBPL3	0.561822893	6.43836564	35.11810146	1.85E-07	2.68E-06
**ENSG00000204389**	HSPA1A	0.815710318	4.752213384	35.10465522	1.86E-07	2.69E-06
**ENSG00000198690**	FAN1	-0.589049014	5.895110093	35.07621908	1.88E-07	2.71E-06
**ENSG00000100813**	ACIN1	-0.348422835	7.849633103	35.06864888	1.88E-07	2.71E-06
**ENSG00000137955**	RABGGTB	-0.610325982	6.549565217	36.2041426	1.88E-07	2.71E-06
**ENSG00000167261**	DPEP2	-0.991534736	5.197580853	37.43105872	1.88E-07	2.71E-06
**ENSG00000243960**		-1.025898484	3.866847263	35.04692023	1.89E-07	2.72E-06
**ENSG00000148400**	notch1	0.506006472	6.240320912	35.002233	1.92E-07	2.76E-06
**ENSG00000057608**	GDI2	0.393878657	7.588334963	34.93500753	1.96E-07	2.81E-06
**ENSG00000120832**	MTERF2	-0.895420083	4.396888447	34.92495602	1.97E-07	2.81E-06
**ENSG00000143970**	ASXL2	0.389043867	7.821963928	34.92182721	1.97E-07	2.81E-06
**ENSG00000142102**	PGGHG	-1.723610846	6.487518709	40.92298021	1.97E-07	2.81E-06
**ENSG00000178607**	ERN 1.00	0.639394198	7.450456877	38.24622055	1.99E-07	2.84E-06
**ENSG00000159086**	PAXBP1	-0.522377194	6.762810648	34.87918853	2E-07	2.84E-06
**ENSG00000002834**	LASP1	0.418695456	6.896848179	34.81498485	2.04E-07	2.9E-06
**ENSG00000132199**	ENOSF1	-0.887688723	5.942327648	38.524773	2.06E-07	2.92E-06
**ENSG00000223865**	HLA-DPB1	0.922630891	4.629013958	34.78154129	2.06E-07	2.92E-06

**ENSG00000117036**	ETV3	0.596532976	5.754651013	34.77804185	2.06E-07	2.92E-06
**ENSG00000163660**	CCNL1	-0.760664507	8.496071028	40.06515913	2.07E-07	2.93E-06
**ENSG00000135074**	ADAM19	0.653857231	6.455743262	36.42731227	2.07E-07	2.93E-06
**ENSG00000029363**	BCLAF1	-0.334235844	8.426681303	34.69052845	2.12E-07	2.99E-06
**ENSG00000023902**	PLEKHO1	0.585261158	5.591045258	34.68914534	2.12E-07	2.99E-06
**ENSG00000144134**	RABL2A	-1.224950171	3.851248731	34.81777366	2.14E-07	3.01E-06
**ENSG00000139631**	CSAD	-1.246577936	4.772992259	37.96038041	2.2E-07	3.08E-06
**ENSG00000187446**	CHP1	0.718119195	5.073226725	34.55495724	2.22E-07	3.11E-06
**ENSG00000130958**	SLC35D2	0.929863502	4.21763103	34.54904654	2.22E-07	3.11E-06
**ENSG00000213639**	ppp1cb	0.426157418	8.55580686	34.51575078	2.25E-07	3.14E-06
**ENSG00000126456**	IRF3	-0.748879172	5.718255947	35.66102066	2.25E-07	3.14E-06
**ENSG00000101082**	SLA2	0.460586985	5.840665815	34.40843441	2.32E-07	3.24E-06
**ENSG00000238121**	LINC00426	0.680008838	5.36998593	34.40325537	2.33E-07	3.24E-06
**ENSG00000163191**	S100A11	1.003460209	6.588814941	39.81641321	2.36E-07	3.28E-06
**ENSG00000143486**	EIF2D	-0.520219654	5.747861546	34.35260614	2.37E-07	3.29E-06
**ENSG00000163082**	SGPP2	1.161932709	3.819325057	34.28233492	2.42E-07	3.36E-06
**ENSG00000112739**	PRPF4B	-0.392296278	8.123003991	34.19634619	2.49E-07	3.45E-06
**ENSG00000176903**	PNMA1	0.640162732	4.840610776	34.08458927	2.58E-07	3.57E-06
**ENSG00000175582**	RAB6A	0.427681063	6.949061598	34.06791824	2.59E-07	3.58E-06
**ENSG00000128394**	APOBEC3F	0.703356811	4.580669301	34.06114203	2.6E-07	3.58E-06
**ENSG00000120253**	NUP43	-0.541130434	5.973441531	34.05835742	2.6E-07	3.58E-06
**ENSG00000076641**	PAG1	0.312535279	8.964754049	34.01420927	2.64E-07	3.63E-06
**ENSG00000118680**	MYL12B	0.555633651	7.603490452	36.10919626	2.67E-07	3.67E-06
**ENSG00000176155**	CCDC57	-0.91161415	5.097533619	35.20203575	2.68E-07	3.67E-06
**ENSG00000144749**	LRIG1	0.452653615	6.42787115	33.94268038	2.7E-07	3.7E-06
**ENSG00000213585**	VDAC1	0.573118154	5.586068007	33.91525591	2.72E-07	3.73E-06
**ENSG00000269926**		1.152035065	4.373519375	35.01670996	2.73E-07	3.73E-06
**ENSG00000047662**	FAM184B	-0.69907289	5.650555386	34.28635301	2.76E-07	3.77E-06
**ENSG00000227039**	ITGB2-AS1	-1.412974562	5.217230005	38.92595982	2.85E-07	3.88E-06
**ENSG00000072778**	ACADVL	-1.021051726	5.246566979	36.68717371	2.86E-07	3.9E-06
**ENSG00000105866**	SP4	0.392268818	7.094876113	33.67444623	2.94E-07	4E-06
**ENSG00000112659**	CUL9	-0.645457469	5.885324585	34.03447088	2.99E-07	4.06E-06
**ENSG00000113597**	TRAPPC13	-0.524809674	5.918405033	33.60294287	3.01E-07	4.09E-06
**ENSG00000128563**	PRKRIP1	-0.748141202	4.643695038	33.5359296	3.08E-07	4.17E-06
**ENSG00000112303**	VNN 2.00	-1.149324103	4.130434555	33.91738376	3.1E-07	4.2E-06
**ENSG00000117984**	CTSD	0.61979615	5.526351985	33.46401268	3.15E-07	4.26E-06
**ENSG00000111885**	MAN1A1	0.717305403	5.311855423	33.44009142	3.18E-07	4.29E-06

**ENSG00000116455**	WDR77	-0.745705015	4.860080243	33.3470368	3.28E-07	4.41E-06
**ENSG00000197102**	DYNC1H1	0.286284183	9.124287261	33.31819107	3.31E-07	4.45E-06
**ENSG00000110422**	HIPK3	0.357367197	8.034200643	33.23262438	3.4E-07	4.57E-06
**ENSG00000180694**	TMEM64	0.773607075	4.739137484	33.21868694	3.41E-07	4.58E-06
**ENSG00000153214**	TMEM87B	0.417956058	6.405747388	33.21669988	3.42E-07	4.58E-06
**ENSG00000132589**	FLOT2	0.448667863	6.054750628	33.17135784	3.47E-07	4.64E-06
**ENSG00000156475**	PPP2R2B	0.994416648	4.192842421	33.13827869	3.51E-07	4.69E-06
**ENSG00000118418**	HMGN3	-0.804751994	4.937106581	33.12760188	3.52E-07	4.7E-06
**ENSG00000129197**	RPAIN	-0.602414095	5.4848664	33.12093494	3.53E-07	4.7E-06
**ENSG00000147526**	TACC1	0.372579278	7.419806366	33.09480242	3.56E-07	4.74E-06
**ENSG00000151623**	NR3C2	-0.469212841	6.257565267	33.05978928	3.6E-07	4.78E-06
**ENSG00000117450**	PRDX1	0.719981178	5.443938762	33.23562799	3.64E-07	4.83E-06
**ENSG00000004534**	RBM6	-0.584715774	7.483088805	35.49347821	3.66E-07	4.85E-06
**ENSG00000185684**	EP400NL	-0.756586786	5.266503908	33.04224749	3.67E-07	4.86E-06
**ENSG00000167978**	SRRM2	-0.443083128	10.3359862	32.99416987	3.67E-07	4.86E-06
**ENSG00000154845**	PPP4R1	0.557608155	5.567857907	32.99133247	3.68E-07	4.86E-06
**ENSG00000283189**		-0.901915698	4.723597472	33.15254037	3.71E-07	4.9E-06
**ENSG00000174013**	FBXO45	0.725485167	4.522567578	32.9550632	3.72E-07	4.91E-06
**ENSG00000153250**	MIR4785	-0.407230126	7.640015723	32.92684562	3.76E-07	4.95E-06
**ENSG00000134884**	ARGLU1	-0.836246069	7.723811485	37.98835175	3.82E-07	5.02E-06
**ENSG00000116560**	SFPQ	-0.333448903	8.789248719	32.81172112	3.9E-07	5.12E-06
**ENSG00000127824**	tuba4a	0.519070448	6.229804607	32.80292118	3.91E-07	5.13E-06
**ENSG00000124783**	SSR1	0.390593018	8.044340731	32.76354101	3.96E-07	5.19E-06
**ENSG00000148248**	SURF4	0.541838089	6.092597946	32.71869887	4.02E-07	5.26E-06
**ENSG00000133858**	ZFC3H1	-0.434075879	8.19685507	32.84011177	4.02E-07	5.26E-06
**ENSG00000121964**	GTDC1	0.856825601	4.471281278	32.70707174	4.04E-07	5.27E-06
**ENSG00000170881**	RNF139	0.480997582	5.825174716	32.69533842	4.05E-07	5.28E-06
**ENSG00000169926**	KLF13	0.352970133	7.704646814	32.635908	4.13E-07	5.38E-06
**ENSG00000205268**	PDE7A	-0.365783072	8.455319969	32.62832801	4.14E-07	5.39E-06
**ENSG00000141429**	GALNT1	0.639618975	5.542703246	32.58818718	4.2E-07	5.45E-06
**ENSG00000167470**	MIDN	0.714462102	4.989970505	32.51750264	4.29E-07	5.57E-06
**ENSG00000169592**	INO80E	-0.797308324	5.16778477	32.88477633	4.3E-07	5.58E-06
**ENSG00000074800**	ENO1	0.454719611	7.263641643	32.50544792	4.31E-07	5.58E-06
**ENSG00000167766**	ZNF83	-0.890452302	6.760516378	37.20049703	4.33E-07	5.6E-06
**ENSG00000103342**	GSPT1	0.359011485	7.730531075	32.46092014	4.38E-07	5.65E-06
**ENSG00000205744**	DENND1C	-0.520244966	6.402771489	32.4338302	4.41E-07	5.69E-06
**ENSG00000259007**		0.729012229	5.094982801	32.42359244	4.43E-07	5.7E-06

**ENSG00000141232**	TOB1	0.443763495	7.274889049	32.41367441	4.44E-07	5.72E-06
**ENSG00000181788**	SIAH2	0.732342404	4.845550519	32.37573501	4.5E-07	5.78E-06
**ENSG00000214106**	PAXIP1-AS2	-0.636567089	4.958166817	32.36858808	4.51E-07	5.79E-06
**ENSG00000197343**	ZNF655	-0.475873202	7.558433203	32.8947807	4.58E-07	5.87E-06
**ENSG00000111203**	LOC100507424	-0.560073677	5.834302104	32.31032045	4.6E-07	5.88E-06
**ENSG00000204388**	hspa1b	0.989548571	3.939178687	32.29856647	4.62E-07	5.9E-06
**ENSG00000177565**	TBL1XR1	0.372011369	8.282648444	32.28174084	4.64E-07	5.93E-06
**ENSG00000055917**	PUM2	0.303357584	8.601826238	32.26709626	4.66E-07	5.95E-06
**ENSG00000120699**	EXOSC8	-0.594086216	5.835120354	32.24364452	4.7E-07	5.99E-06
**ENSG00000026025**	VIM	0.445157904	9.839688013	32.42355128	4.74E-07	6.04E-06
**ENSG00000068400**	GRIPAP1	-0.634141927	5.86167541	32.4517851	4.8E-07	6.1E-06
**ENSG00000240053**	LY6G5B	-1.035839952	4.448465231	32.75591318	4.83E-07	6.13E-06
**ENSG00000178852**	Efcab13	-0.807842289	4.742667926	32.14053829	4.86E-07	6.16E-06
**ENSG00000092841**	MYL6	0.423264374	7.087890861	32.07454211	4.97E-07	6.29E-06
**ENSG00000178104**	PDE4DIP	0.448868599	6.325980323	32.06543582	4.98E-07	6.3E-06
**ENSG00000258890**	CEP95	-0.575449382	6.465235768	32.69558671	5E-07	6.31E-06
**ENSG00000165934**	CPSF2	0.357748462	6.900180596	31.98018209	5.13E-07	6.47E-06
**ENSG00000258728**		-0.946392344	5.007752229	33.47679832	5.14E-07	6.47E-06
**ENSG00000070081**	LOC105376575	-0.625736328	6.418639629	33.34628292	5.14E-07	6.47E-06
**ENSG00000147168**	IL2RG	0.361035031	8.505762623	31.91752495	5.23E-07	6.58E-06
**ENSG00000150054**	MPP7	-0.513981897	6.173457528	31.91240093	5.24E-07	6.58E-06
**ENSG00000145730**	PAM	0.659841556	5.221217806	31.87767813	5.3E-07	6.65E-06
**ENSG00000138658**	ZGRF1	-0.873729058	4.20159771	31.86305813	5.33E-07	6.67E-06
**ENSG00000130429**	ARPC1B	0.475727849	6.784452677	31.84035074	5.37E-07	6.72E-06
**ENSG00000256525**	POLG2	-0.758078244	5.022374988	31.82213595	5.4E-07	6.75E-06
**ENSG00000100243**	cyb5r3	0.601658763	5.032468455	31.80225388	5.44E-07	6.79E-06
**ENSG00000145882**	PCYOX1L	-0.649142504	5.317311864	31.70367725	5.62E-07	7E-06
**ENSG00000066027**	PPP2R5A	0.365089373	6.692650398	31.63785756	5.74E-07	7.15E-06
**ENSG00000160685**	ZBTB7B	0.570469758	5.295225119	31.6352778	5.75E-07	7.15E-06
**ENSG00000109756**	RAPGEF2	0.547129903	5.648290809	31.61891132	5.78E-07	7.17E-06
**ENSG00000118922**	KLF12	0.290057158	8.312606273	31.61803628	5.78E-07	7.17E-06
**ENSG00000071575**	TRIB2	-0.396658286	7.349892644	31.57780424	5.86E-07	7.26E-06
**ENSG00000118579**	MED28	-0.362801224	7.232982043	31.50519604	6E-07	7.43E-06
**ENSG00000113558**	SKP1	-0.369544782	8.40097501	31.48551658	6.04E-07	7.47E-06
**ENSG00000259431**	THTPA	-0.781054822	4.864723678	31.46232605	6.09E-07	7.51E-06
**ENSG00000198625**	MDM4	-0.375214082	8.467394683	31.45927558	6.09E-07	7.51E-06
**ENSG00000205356**	TECPR1	-0.610343089	5.860630474	31.48253996	6.14E-07	7.56E-06

**ENSG00000267002**		-0.941204454	4.438348059	31.41313252	6.19E-07	7.61E-06
**ENSG00000197956**	S100A6	0.634193179	5.898144066	31.87107367	6.2E-07	7.62E-06
**ENSG00000132334**	PTPRE	0.496358903	5.838418303	31.35215188	6.31E-07	7.75E-06
**ENSG00000152642**	GPD1L	0.531134007	5.285159786	31.34613523	6.33E-07	7.76E-06
**ENSG00000134242**	PTPN22	0.383444429	6.611479556	31.32475445	6.37E-07	7.8E-06
**ENSG00000119318**	RAD23B	0.355991661	6.808116814	31.30884643	6.41E-07	7.83E-06
**ENSG00000072736**	NFATC3	0.32225582	8.283304142	31.25043797	6.53E-07	7.97E-06
**ENSG00000271680**		-1.003964416	3.672120367	31.24887466	6.54E-07	7.97E-06
**ENSG00000225948**		-0.807786249	4.303363355	31.23513968	6.57E-07	8E-06
**ENSG00000005206**	SPPL2B	-1.255386709	4.467477733	33.53332457	6.65E-07	8.09E-06
**ENSG00000084090**	STARD7	0.373594538	6.425758715	31.19100113	6.66E-07	8.1E-06
**ENSG00000073331**	alpk1	-0.874428029	4.648617462	31.17159285	6.71E-07	8.14E-06
**ENSG00000017797**	RALBP1	0.480821456	5.923457759	31.1694778	6.71E-07	8.14E-06
**ENSG00000234608**	MAPKAPK5-AS1	-0.736627058	4.694123021	31.16220137	6.73E-07	8.15E-06
**ENSG00000061936**	SFSWAP	-0.529521664	6.710676823	31.5754103	6.75E-07	8.17E-06
**ENSG00000091542**	ALKBH5	0.467524076	6.372945744	31.14298272	6.77E-07	8.18E-06
**ENSG00000144445**	KANSL1L	-0.603983744	5.833729209	31.13792769	6.78E-07	8.19E-06
**ENSG00000184205**	TSPYL2	-0.528202602	6.248130549	31.12756146	6.81E-07	8.2E-06
**ENSG00000135932**	CAB39	0.361578977	7.805593899	31.12730855	6.81E-07	8.2E-06
**ENSG00000235079**		-0.76568306	4.842433766	31.08787019	6.9E-07	8.3E-06
**ENSG00000186575**	NF2	0.53237731	5.625808757	31.07114518	6.93E-07	8.34E-06
**ENSG00000126264**	HCST	0.899993274	5.034629581	32.30551987	7E-07	8.4E-06
**ENSG00000198730**	CTR9	0.390179556	7.058229999	31.01641989	7.06E-07	8.47E-06
**ENSG00000005020**	SKAP2	1.009305113	4.203484561	31.00828816	7.08E-07	8.48E-06
**ENSG00000119335**	SET	0.406415721	8.306454725	30.98622448	7.13E-07	8.54E-06
**ENSG00000136770**	DNAJC1	0.757401621	4.517827253	30.94300155	7.24E-07	8.65E-06
**ENSG00000165175**	MID1IP1	0.739156724	4.667219227	30.93500249	7.26E-07	8.66E-06
**ENSG00000228506**		-0.733585001	4.794390514	30.9102623	7.32E-07	8.73E-06
**ENSG00000142669**	SH3BGRL3	0.757332873	7.447366248	35.13627393	7.38E-07	8.79E-06
**ENSG00000100345**	MYH9	0.345978198	10.53747687	30.87618777	7.4E-07	8.8E-06
**ENSG00000274292**		-0.867262378	4.759714295	31.12830499	7.41E-07	8.8E-06
**ENSG00000250644**		0.63812191	5.183765584	30.8720905	7.41E-07	8.8E-06
**ENSG00000176225**	RTTN	-0.544727749	5.946057306	30.86033569	7.44E-07	8.82E-06
**ENSG00000177119**	ANO6	0.419122777	6.323445785	30.85152175	7.46E-07	8.84E-06
**ENSG00000122417**	ODF2L	-0.638366765	7.086474021	33.54833284	7.59E-07	8.98E-06
**ENSG00000139190**	VAMP1	-0.750958988	5.78276166	32.42163502	7.75E-07	9.15E-06
**ENSG00000167460**	TPM4	0.375695929	7.122902497	30.56643096	8.21E-07	9.69E-06

**ENSG00000105887**	MTPN	0.408478262	7.413967952	30.56459138	8.22E-07	9.69E-06
**ENSG00000225205**		-0.690856287	4.887469286	30.51152172	8.37E-07	9.85E-06
**ENSG00000159658**	EFCAB14	0.365911146	7.942575632	30.50709696	8.38E-07	9.86E-06
**ENSG00000178996**	SNX18	0.573461211	5.564085113	30.42765254	8.61E-07	1.01E-05
**ENSG00000091592**	NLRP1	-0.734586494	7.904539108	34.62196443	8.61E-07	1.01E-05
**ENSG00000137492**	THAP12	0.388358446	6.483115391	30.38939803	8.72E-07	1.02E-05
**ENSG00000143624**	INTS3	-0.515350918	6.185020979	30.38383171	8.73E-07	1.02E-05
**ENSG00000142227**	EMP3	0.629774832	6.788484364	32.53894989	8.81E-07	1.03E-05
**ENSG00000272888**		-0.695515684	7.56906358	34.14596717	8.95E-07	1.05E-05
**ENSG00000165359**	INTS6L	-0.727983239	6.255953207	32.82248324	8.98E-07	1.05E-05
**ENSG00000164284**	grpel2	0.482001785	6.386181995	30.2773377	9.05E-07	1.06E-05
**ENSG00000214900**	LINC01588	-0.874562396	4.826107033	30.75455665	9.12E-07	1.06E-05
**ENSG00000085063**	CD59	0.648771439	5.120934794	30.21135246	9.26E-07	1.08E-05
**ENSG00000108474**	PIGL	-0.854947158	5.210165451	31.44443347	9.4E-07	1.09E-05
**ENSG00000162601**	MYSM1	-0.38992063	7.251798584	30.1547778	9.44E-07	1.1E-05
**ENSG00000157654**	PALM2	0.738899844	4.984267417	30.14615939	9.46E-07	1.1E-05
**ENSG00000122965**	RBM19	-0.49653376	5.923758257	30.14360661	9.47E-07	1.1E-05
**ENSG00000185697**	MYBL1	0.937433048	6.900642375	34.51266458	9.48E-07	1.1E-05
**ENSG00000123983**	ACSL3	0.582554326	5.680916414	30.13573454	9.5E-07	1.1E-05
**ENSG00000116815**	CD58	0.912805312	4.579965545	30.46748322	9.5E-07	1.1E-05
**ENSG00000242086**	SDHAP2	-0.506135985	6.657978267	30.18182035	9.59E-07	1.11E-05
**ENSG00000160932**	LY6E	0.670523283	4.990306985	30.09843675	9.62E-07	1.11E-05
**ENSG00000123416**	TUBA1B	0.52257565	6.316492891	30.09187479	9.64E-07	1.11E-05
**ENSG00000162909**	CAPN2	0.332683495	8.010321908	30.06238655	9.73E-07	1.12E-05
**ENSG00000119408**	NEK6	0.743080573	4.63434608	30.0374341	9.82E-07	1.13E-05
**ENSG00000064995**	TAF11	-0.63495331	4.664504406	29.9952182	9.96E-07	1.14E-05
**ENSG00000101265**	RASSF2	0.40928984	6.825778398	29.98384781	1E-06	1.14E-05
**ENSG00000241489**		0.376980866	6.503432633	29.94321781	1.01E-06	1.16E-05
**ENSG00000170144**	MIR4444-1	-0.304533139	8.932805058	29.93685303	1.02E-06	1.16E-05
**ENSG00000198089**	SFI1	-0.5066908	7.109680016	30.78409858	1.02E-06	1.17E-05
**ENSG00000205581**	HMGN1	-0.431444356	7.284227165	29.90852044	1.03E-06	1.17E-05
**ENSG00000239653**	PSMD6-AS2	-0.762955523	5.301143796	30.45602233	1.03E-06	1.17E-05
**ENSG00000237298**	TTN-AS1	-0.721061869	7.224403895	33.58630932	1.03E-06	1.17E-05
**ENSG00000141337**	ARSG	-0.793865609	4.707268784	29.8881567	1.03E-06	1.17E-05
**ENSG00000108510**	MED13	0.304332393	8.076479723	29.87048787	1.04E-06	1.18E-05
**ENSG00000086300**	SNX10	0.892278413	4.187062615	29.86158939	1.04E-06	1.18E-05
**ENSG00000122958**	VPS26A	0.534950749	5.672494569	29.86101022	1.04E-06	1.18E-05

**ENSG00000078304**	PPP2R5C	0.270565224	8.729647943	29.84398772	1.05E-06	1.19E-05
**ENSG00000090975**	PITPNM2	-0.686919361	5.317198496	29.75261458	1.08E-06	1.22E-05
**ENSG00000166289**	PLEKHF1	0.950555277	3.644195541	29.74776199	1.08E-06	1.22E-05
**ENSG00000077585**	GPR137B	0.860885423	3.978524853	29.7414689	1.09E-06	1.22E-05
**ENSG00000270055**		-1.25686208	3.816469809	30.46312573	1.09E-06	1.23E-05
**ENSG00000169508**	GPR183	0.574600222	8.172527509	32.46117042	1.1E-06	1.24E-05
**ENSG00000012061**	ercc1	-0.622039111	4.990723524	29.68378946	1.11E-06	1.24E-05
**ENSG00000196154**	S100A4	1.027331794	6.62472006	33.93419743	1.12E-06	1.26E-05
**ENSG00000187210**	GCNT1	1.05582016	3.591898758	29.63492694	1.13E-06	1.26E-05
**ENSG00000166272**	WBP1L	0.486725183	5.626533635	29.61569463	1.13E-06	1.27E-05
**ENSG00000187742**	SECISBP2	-0.545361422	7.166260728	31.17406763	1.14E-06	1.27E-05
**ENSG00000137409**	MTCH1	-0.521483218	5.800551629	29.58932799	1.14E-06	1.28E-05
**ENSG00000176715**	ACSF3	-0.631849974	5.780766505	29.89899406	1.15E-06	1.29E-05
**ENSG00000056586**	RC3H2	0.332795405	7.527902458	29.53780914	1.16E-06	1.3E-05
**ENSG00000136108**	CKAP2	0.584746894	5.317080469	29.53515084	1.16E-06	1.3E-05
**ENSG00000116574**	RHOU	1.026465793	4.142426315	29.56767172	1.17E-06	1.3E-05
**ENSG00000241978**	AKAP2	0.727766302	5.003963947	29.47277716	1.19E-06	1.32E-05
**ENSG00000258017**		0.548930246	6.029743104	29.43378695	1.21E-06	1.34E-05
**ENSG00000104852**	SNRNP70	-0.996976835	6.979632349	33.68511131	1.21E-06	1.35E-05
**ENSG00000184613**	NELL2	-0.724767851	6.920189705	32.73417633	1.23E-06	1.36E-05
**ENSG00000164305**	CASP3	0.564443771	5.343494182	29.35012813	1.24E-06	1.37E-05
**ENSG00000127526**	SLC35E1	0.567136319	5.523443437	29.33672433	1.25E-06	1.38E-05
**ENSG00000122694**	GLIPR2	0.672365499	4.990767964	29.282399	1.27E-06	1.4E-05
**ENSG00000116747**	TROVE2	0.372673243	7.73276742	29.2380121	1.29E-06	1.42E-05
**ENSG00000100351**	GRAP2	-0.372968079	6.93248404	29.21040237	1.3E-06	1.43E-05
**ENSG00000114021**	NIT2	-0.627983407	5.547375263	29.16673947	1.32E-06	1.45E-05
**ENSG00000164754**	MIR3610	0.307230798	8.41105211	29.16553268	1.32E-06	1.45E-05
**ENSG00000004468**	CD38	-1.155433969	3.949439446	29.61609576	1.32E-06	1.45E-05
**ENSG00000137817**	PARP6	-0.684749967	5.859699471	30.2993091	1.33E-06	1.46E-05
**ENSG00000258875**		0.938242297	3.406811202	29.11325467	1.34E-06	1.47E-05
**ENSG00000173890**	gpr160	-0.975937255	4.19997329	29.11182449	1.35E-06	1.47E-05
**ENSG00000077150**	NFKB2	-0.6694675	5.139077335	29.09033568	1.36E-06	1.48E-05
**ENSG00000174738**	NR1D2	0.377027721	7.177038069	29.0210702	1.39E-06	1.52E-05
**ENSG00000173846**	PLK3	0.636941702	5.487529756	28.99425301	1.4E-06	1.53E-05
**ENSG00000165650**	PDZD8	0.468945641	6.157205929	28.95161072	1.42E-06	1.55E-05
**ENSG00000138376**	BARD1	0.898729356	4.56751226	29.25909801	1.43E-06	1.56E-05
**ENSG00000132635**	PCED1A	-1.160641446	3.967166569	29.46231821	1.44E-06	1.57E-05

**ENSG00000115548**	KDM3A	-0.396059258	7.520140189	28.90805165	1.44E-06	1.57E-05
**ENSG00000170989**	S1PR1	0.410710737	8.180889533	29.0718976	1.45E-06	1.57E-05
**ENSG00000153561**	RMND5A	0.403800036	6.462696374	28.87817928	1.46E-06	1.58E-05
**ENSG00000145391**	SETD7	0.649094071	5.033679601	28.81872585	1.49E-06	1.61E-05
**ENSG00000151414**	NEK7	0.408717014	6.991513233	28.7899351	1.5E-06	1.63E-05
**ENSG00000274810**	NPHP3-ACAD11	-0.732926095	6.488489957	31.59492001	1.51E-06	1.64E-05
**ENSG00000108518**	PFN1	0.490050857	8.286868904	30.31898403	1.52E-06	1.64E-05
**ENSG00000198951**	NAGA	0.695478425	4.288133349	28.69232314	1.55E-06	1.68E-05
**ENSG00000114978**	mob1a	0.356892881	8.141763598	28.64560621	1.58E-06	1.7E-05
**ENSG00000128951**	DUT	-0.583011783	5.43472372	28.64457579	1.58E-06	1.7E-05
**ENSG00000111669**	TPI1	0.569315335	5.641685703	28.64273978	1.58E-06	1.7E-05
**ENSG00000221823**	PPP3R1	0.43407193	6.562435522	28.6102838	1.6E-06	1.72E-05
**ENSG00000143761**	MIR3620	0.453286956	6.934912175	28.60825286	1.6E-06	1.72E-05
**ENSG00000118689**	FOXO3	0.524490095	5.740526966	28.60754264	1.6E-06	1.72E-05
**ENSG00000060971**	ACAA1	-0.749440973	4.958212087	28.60663624	1.6E-06	1.72E-05
**ENSG00000155096**	AZIN1	0.401698134	7.046671417	28.56717602	1.62E-06	1.74E-05
**ENSG00000135926**	MIR6513	0.490276006	6.047307074	28.56529572	1.62E-06	1.74E-05
**ENSG00000124767**	GLO1	0.581703601	5.49383227	28.56369565	1.62E-06	1.74E-05
**ENSG00000027075**	PRKCH	0.322337773	8.165428136	28.56186412	1.63E-06	1.74E-05
**ENSG00000279232**		-0.951761415	3.875514684	28.54677639	1.63E-06	1.74E-05
**ENSG00000270231**	NBPF8	-0.686344745	5.554164185	28.99619381	1.64E-06	1.75E-05
**ENSG00000262160**		0.388197163	6.314058045	28.51908557	1.65E-06	1.76E-05
**ENSG00000283199**		-0.923102469	5.068053325	30.05955042	1.66E-06	1.76E-05
**ENSG00000239306**	RBM14	-0.421417281	6.468004008	28.45821257	1.68E-06	1.79E-05
**ENSG00000152582**	SPEF2	-0.722492293	5.148279295	28.53713061	1.69E-06	1.79E-05
**ENSG00000144026**	ZNF514	-0.791715285	4.948628783	28.6435335	1.69E-06	1.8E-05
**ENSG00000108262**	GIT1	0.564281408	5.553970356	28.4291257	1.7E-06	1.8E-05
**ENSG00000163378**	EOGT	0.602631675	5.099111931	28.39210886	1.72E-06	1.82E-05
**ENSG00000126602**	TRAP1	-0.56340183	5.517168503	28.37484525	1.73E-06	1.83E-05
**ENSG00000246596**		-0.802825682	4.236664062	28.36127867	1.74E-06	1.84E-05
**ENSG00000108819**	PPP1R9B	0.457200931	5.956880537	28.33393344	1.76E-06	1.86E-05
**ENSG00000085491**	SLC25A24	0.60691121	5.584582817	28.29921297	1.78E-06	1.88E-05
**ENSG00000168175**	MAPK1IP1L	0.347130851	7.345867352	28.2659225	1.8E-06	1.9E-05
**ENSG00000079134**	THOC1	-0.435526166	6.361177822	28.25428852	1.81E-06	1.9E-05
**ENSG00000099875**	MKNK2	0.470191798	6.574329359	28.22902497	1.82E-06	1.92E-05
**ENSG00000210112**		-0.909625943	4.19114686	28.22597986	1.82E-06	1.92E-05
**ENSG00000081019**	RSBN1	0.33092976	7.377548842	28.17717656	1.86E-06	1.95E-05

**ENSG00000197150**	ABCB8	-0.846692912	4.67941035	28.30460763	1.88E-06	1.96E-05
**ENSG00000086598**	TMED2	0.506409334	6.567086734	28.421896	1.88E-06	1.96E-05
**ENSG00000107581**	EIF3A	0.348019987	8.861793408	28.10233338	1.9E-06	1.99E-05
**ENSG00000065665**	SEC61A2	-0.785862777	4.696042513	28.0914556	1.91E-06	2E-05
**ENSG00000132716**	dcaf8	-0.454002381	6.848701562	28.08074349	1.92E-06	2E-05
**ENSG00000055332**	eif2ak2	0.42229922	7.316595028	28.05817052	1.93E-06	2.02E-05
**ENSG00000251022**	THAP9-AS1	-0.648557286	5.411463904	28.05607316	1.94E-06	2.02E-05
**ENSG00000134109**	EDEM1	0.283483829	7.699345959	28.03112531	1.95E-06	2.03E-05
**ENSG00000116954**	RRAGC	0.615505333	4.950834804	28.01926618	1.96E-06	2.04E-05
**ENSG00000181827**	RFX7	0.349652288	7.168842679	28.00905798	1.97E-06	2.04E-05
**ENSG00000137070**	il11ra	-1.162425831	5.90077214	31.84016908	1.97E-06	2.05E-05
**ENSG00000100219**	XBP1	0.408639045	6.747595411	27.9691938	1.99E-06	2.07E-05
**ENSG00000073921**	PICALM	0.330751299	7.409710231	27.96153311	2E-06	2.07E-05
**ENSG00000278050**		-1.159952908	5.348295746	31.37751032	2E-06	2.07E-05
**ENSG00000153560**	UBP1	-0.365509268	6.88067505	27.92154855	2.03E-06	2.1E-05
**ENSG00000271964**		-1.04639356	4.528779219	29.14200635	2.04E-06	2.11E-05
**ENSG00000157306**		-0.713297382	5.186314349	27.98989084	2.04E-06	2.11E-05
**ENSG00000071054**	map4k4	-0.335316723	7.707821282	27.88031043	2.06E-06	2.12E-05
**ENSG00000156398**	SFXN2	-0.823997516	4.455978642	27.86503219	2.07E-06	2.13E-05
**ENSG00000102580**	DNAJC3	0.450756321	6.263641041	27.84908851	2.08E-06	2.14E-05
**ENSG00000141522**	ARHGDIA	0.438703336	6.601810829	27.82333464	2.1E-06	2.16E-05
**ENSG00000163564**	PYHIN1	0.515089283	6.79204184	28.5178704	2.1E-06	2.16E-05
**ENSG00000108799**	EZH1	-0.404242823	6.868897483	27.78926874	2.12E-06	2.18E-05
**ENSG00000266173**	STRADA	-0.515588364	6.21613476	27.77605847	2.13E-06	2.19E-05
**ENSG00000182952**	HMGN4	0.473105998	5.602336545	27.76234491	2.14E-06	2.19E-05
**ENSG00000280135**		-0.62803617	4.944086331	27.73965831	2.16E-06	2.21E-05
**ENSG00000198380**	GFPT1	0.471406881	6.304631947	27.71665794	2.18E-06	2.22E-05
**ENSG00000115762**	plekhb2	0.399294834	6.153558754	27.71436197	2.18E-06	2.22E-05
**ENSG00000105639**	JAK3	-0.39942356	7.999978067	27.72952923	2.21E-06	2.25E-05
**ENSG00000138594**	TMOD3	0.346016973	7.314972997	27.66733644	2.22E-06	2.26E-05
**ENSG00000026508**	CD44	0.333869798	9.792697511	27.65845533	2.22E-06	2.26E-05
**ENSG00000163219**	ARHGAP25	0.342735257	7.241451604	27.65580337	2.22E-06	2.26E-05
**ENSG00000026950**	BTN3A1	-0.509940629	7.583341971	29.19933428	2.26E-06	2.29E-05
**ENSG00000153814**	JAZF1	0.561386907	5.087032606	27.60849958	2.26E-06	2.29E-05
**ENSG00000276045**	ORAI1	0.603904723	5.360242106	27.60181811	2.27E-06	2.3E-05
**ENSG00000167004**	PDIA3	0.356838723	7.350289741	27.59918478	2.27E-06	2.3E-05
**ENSG00000252561**		1.033250058	4.887131243	29.49554063	2.32E-06	2.35E-05

**ENSG00000129595**	EPB41L4A	-1.183573726	3.892585966	28.19548626	2.33E-06	2.35E-05
**ENSG00000183943**	PRKX	0.354591183	7.152923249	27.46274089	2.38E-06	2.4E-05
**ENSG00000149925**	ALDOA	0.452329212	7.350445256	27.98812737	2.38E-06	2.4E-05
**ENSG00000188042**	ARL4C	0.332299115	8.395423396	27.45557182	2.39E-06	2.4E-05
**ENSG00000185862**	EVI2B	0.389894719	9.055471487	27.44190648	2.4E-06	2.41E-05
**ENSG00000064393**	HIPK2	-0.381520784	7.189373358	27.41487268	2.42E-06	2.43E-05
**ENSG00000224032**		-0.764429128	4.525698232	27.39115004	2.44E-06	2.45E-05
**ENSG00000106692**	fktn	-0.589504135	4.941882235	27.3887922	2.44E-06	2.45E-05
**ENSG00000097096**	SYDE2	-0.948810376	3.875760623	27.353948	2.47E-06	2.48E-05
**ENSG00000080189**	SLC35C2	-0.588761711	5.697347996	27.34426212	2.48E-06	2.48E-05
**ENSG00000170385**	SLC30A1	0.656542559	5.007840423	27.32711038	2.5E-06	2.5E-05
**ENSG00000276550**	HERC2P2	-0.876776911	4.951096129	28.18370264	2.58E-06	2.58E-05
**ENSG00000269693**		0.498217335	5.506939113	27.20816103	2.6E-06	2.6E-05
**ENSG00000177570**	SAMD12	-1.141100579	3.943823239	27.79271498	2.61E-06	2.6E-05
**ENSG00000132965**	ALOX5AP	0.615683859	5.242732121	27.18654197	2.62E-06	2.61E-05
**ENSG00000158805**	ZNF276	-0.435785372	7.020442459	27.15235439	2.65E-06	2.64E-05
**ENSG00000168876**	ANKRD49	-0.49690894	5.794831658	27.13574401	2.67E-06	2.66E-05
**ENSG00000197535**	MYO5A	0.392093305	7.359625561	27.08249633	2.72E-06	2.7E-05
**ENSG00000281005**	LINC00921	-0.798602351	4.427297069	27.08232708	2.72E-06	2.7E-05
**ENSG00000273015**		-0.571743527	5.318654024	27.08153011	2.72E-06	2.7E-05
**ENSG00000099622**	CIRBP	-0.47058924	7.521500652	28.01337733	2.74E-06	2.71E-05
**ENSG00000076770**	MBNL3	0.397246708	7.12949104	27.03106209	2.77E-06	2.74E-05
**ENSG00000064201**	TSPAN32	-0.877259274	4.570332388	27.30060465	2.78E-06	2.75E-05
**ENSG00000204525**	HLA-C	0.361353173	9.718267885	27.01747738	2.78E-06	2.75E-05
**ENSG00000166801**	FAM111A	-0.529472147	7.156661252	28.42980102	2.79E-06	2.76E-05
**ENSG00000122122**	SASH3	0.449394571	6.514618976	27.00139719	2.8E-06	2.76E-05
**ENSG00000284564**		-0.94158636	5.861030922	30.06976411	2.87E-06	2.83E-05
**ENSG00000143851**	PTPN7	0.500177129	5.764697021	26.90210689	2.9E-06	2.85E-05
**ENSG00000153187**	HNRNPU	-0.290795451	9.964953459	26.899058	2.9E-06	2.85E-05
**ENSG00000166582**	cenpv	-0.92066387	4.006452846	26.89456122	2.9E-06	2.86E-05
**ENSG00000167196**	FBXO22	-0.680703892	5.511397018	27.32098132	2.93E-06	2.88E-05
**ENSG00000261864**		0.445399995	5.83825624	26.82346783	2.98E-06	2.92E-05
**ENSG00000173011**	TADA2B	0.476806687	5.670036001	26.81401313	2.99E-06	2.93E-05
**ENSG00000120265**	PCMT1	0.55315955	5.214264653	26.81180721	2.99E-06	2.93E-05
**ENSG00000137845**	ADAM10	0.297910689	8.151147562	26.77764709	3.03E-06	2.96E-05
**ENSG00000127419**	tmem175	-0.876664881	4.270524702	26.74927363	3.06E-06	2.99E-05
**ENSG00000242588**		-0.527160908	5.626649836	26.74746057	3.06E-06	2.99E-05

**ENSG00000108061**	SHOC2	0.385326854	6.788223405	26.70075098	3.11E-06	3.03E-05
**ENSG00000163519**	trat1	-0.407871385	7.699702676	26.8974825	3.11E-06	3.04E-05
**ENSG00000116095**	PLEKHA3	0.379957773	6.326036581	26.65913154	3.16E-06	3.07E-05
**ENSG00000198538**	ZNF28	0.551467984	5.163821557	26.64366012	3.17E-06	3.09E-05
**ENSG00000050426**	LETMD1	-0.537991089	5.742887736	26.62568662	3.19E-06	3.1E-05
**ENSG00000234663**	LOC101927156	0.608447576	4.537786077	26.59612266	3.23E-06	3.13E-05
**ENSG00000124486**	USP9X	0.287143983	8.444878695	26.56626999	3.26E-06	3.16E-05
**ENSG00000170779**	CDCA4	0.863930155	3.55891265	26.55834077	3.27E-06	3.17E-05
**ENSG00000173442**	EHBP1L1	-0.510963947	6.076310986	26.54443081	3.29E-06	3.18E-05
**ENSG00000131171**	SH3BGRL	0.387401401	6.85726241	26.52096082	3.31E-06	3.21E-05
**ENSG00000167895**	tmc8	-0.60690047	8.103362949	29.34982232	3.32E-06	3.21E-05
**ENSG00000077420**	APBB1IP	0.305936833	7.486731198	26.46858798	3.38E-06	3.26E-05
**ENSG00000123739**	PLA2G12A	-0.624584002	4.975655224	26.45471987	3.39E-06	3.27E-05
**ENSG00000266338**	NBPF15	-0.575407049	6.193745258	27.16803822	3.4E-06	3.28E-05
**ENSG00000171223**	JUNB	0.912180134	6.051961678	29.58434371	3.41E-06	3.28E-05
**ENSG00000156802**	ATAD2	0.603477082	5.997641095	27.24434013	3.44E-06	3.31E-05
**ENSG00000186951**	PPARA	0.615087787	4.96074784	26.40790395	3.45E-06	3.32E-05
**ENSG00000271913**		-0.426250477	6.62789854	26.4051457	3.45E-06	3.32E-05
**ENSG00000115241**	PPM1G	0.459576098	5.768961136	26.36594133	3.5E-06	3.36E-05
**ENSG00000284292**		0.401220318	6.806083659	26.35942382	3.51E-06	3.36E-05
**ENSG00000275183**	LENG9	-1.004930484	4.454052098	27.12545457	3.54E-06	3.38E-05
**ENSG00000112297**	AIM1	0.26403871	7.858051009	26.33808055	3.54E-06	3.38E-05
**ENSG00000064607**	SUGP2	-0.882037865	6.618215163	29.62348041	3.58E-06	3.42E-05
**ENSG00000165516**	KLHDC2	-0.426879237	6.743519917	26.24569316	3.65E-06	3.49E-05
**ENSG00000163611**	SPICE1	-0.590387537	5.432347615	26.21861372	3.69E-06	3.52E-05
**ENSG00000185669**	SNAI3	0.910797049	3.594171863	26.21668686	3.69E-06	3.52E-05
**ENSG00000155657**	TTN	-0.746162693	9.438197626	29.49922576	3.76E-06	3.59E-05
**ENSG00000114861**	FOXP1	-0.320313226	8.919017189	26.15292409	3.78E-06	3.59E-05
**ENSG00000128872**	TMOD2	-0.499396572	5.695794397	26.10202807	3.85E-06	3.66E-05
**ENSG00000131389**	SLC6A6	0.450234339	5.969419	26.09261233	3.86E-06	3.66E-05
**ENSG00000138767**	CNOT6L	0.317309952	8.607460168	26.03950068	3.93E-06	3.73E-05
**ENSG00000102753**	KPNA3	0.440456481	6.153097121	26.0322375	3.94E-06	3.74E-05
**ENSG00000162434**	JAK1	0.292593326	9.068258942	26.02621384	3.95E-06	3.74E-05
**ENSG00000072071**	ADGRL1	-0.767421055	4.846126777	26.09783747	3.96E-06	3.75E-05
**ENSG00000226232**		-1.015902385	3.682238991	26.01595747	3.97E-06	3.75E-05
**ENSG00000204406**	mbd5	-0.439041802	6.606950787	25.99360145	4E-06	3.78E-05
**ENSG00000243305**		-1.000853372	3.751365034	25.98039049	4.02E-06	3.79E-05

**ENSG00000163950**	SLBP	0.526140005	5.334374597	25.9802668	4.02E-06	3.79E-05
**ENSG00000182179**	MIR5193	-0.676178765	6.298484237	27.96254771	4.02E-06	3.79E-05
**ENSG00000102910**	LOC100507577	-0.306578812	7.575265587	25.94832008	4.06E-06	3.82E-05
**ENSG00000150991**	UBC	0.34844796	8.723269846	25.90697393	4.12E-06	3.88E-05
**ENSG00000139163**	ETNK1	0.344552717	7.452187981	25.90284908	4.13E-06	3.88E-05
**ENSG00000197976**	AKAP17A	-0.687560526	5.99052996	27.46577017	4.18E-06	3.92E-05
**ENSG00000168646**	AXIN2	-0.571578134	5.355288568	25.84659267	4.21E-06	3.95E-05
**ENSG00000139641**	ESYT1	0.309878107	7.897700559	25.82909996	4.24E-06	3.97E-05
**ENSG00000112033**	PPARD	-0.550163129	5.306939893	25.71051514	4.42E-06	4.14E-05
**ENSG00000100629**	CEP128	0.668956518	5.241338059	25.819215	4.44E-06	4.16E-05
**ENSG00000204161**	C10orf128	0.842146645	4.400633209	25.69126611	4.45E-06	4.16E-05
**ENSG00000129422**	MTUS1	-0.986933157	4.06375663	25.68223428	4.47E-06	4.17E-05
**ENSG00000178188**	SH2B1	-0.83228916	4.761701958	26.0466663	4.48E-06	4.18E-05
**ENSG00000140853**	NLRC5	-0.567805202	8.312352496	28.15024413	4.51E-06	4.2E-05
**ENSG00000198556**	ZNF789	-0.768196809	5.117628106	26.25973377	4.51E-06	4.2E-05
**ENSG00000266412**	NCOA4	0.311107045	7.293645039	25.64155228	4.53E-06	4.22E-05
**ENSG00000169230**	PRELID1	0.552654193	4.870786959	25.62863227	4.55E-06	4.23E-05
**ENSG00000214894**	LINC00243	-0.796768967	4.188883308	25.60645944	4.59E-06	4.26E-05
**ENSG00000155111**	CDK19	0.551416778	5.234299404	25.60057536	4.6E-06	4.27E-05
**ENSG00000204946**	ZNF783	-0.858082157	5.005123024	26.72125324	4.64E-06	4.3E-05
**ENSG00000280194**		0.791663557	4.805708673	25.88024517	4.65E-06	4.3E-05
**ENSG00000196470**	SIAH1	-0.511167919	5.528529792	25.55986754	4.67E-06	4.32E-05
**ENSG00000196440**	ARMCX4	-1.059354455	3.957161751	25.76966635	4.7E-06	4.35E-05
**ENSG00000118308**	LRMP	-0.525584128	5.581475425	25.5229916	4.73E-06	4.37E-05
**ENSG00000215440**	NPEPL1	-0.99185104	4.627741695	26.7942025	4.75E-06	4.38E-05
**ENSG00000010610**	CD4	0.319591767	8.046490111	25.50842888	4.75E-06	4.38E-05
**ENSG00000126882**	FAM78A	0.311429138	7.031181953	25.48589338	4.79E-06	4.42E-05
**ENSG00000044574**	HSPA5	0.369825233	7.303062126	25.47156283	4.82E-06	4.43E-05
**ENSG00000182199**	SHMT2	0.698252014	4.371267388	25.46976906	4.82E-06	4.43E-05
**ENSG00000234745**	HLA-B	0.358716056	10.74339623	25.4222171	4.9E-06	4.51E-05
**ENSG00000107099**	DOCK8	0.264789359	9.176346336	25.38310554	4.97E-06	4.57E-05
**ENSG00000108963**	DPH1	-0.589292183	5.022539115	25.33090274	5.07E-06	4.64E-05
**ENSG00000118496**	FBXO30	0.609094954	5.34754884	25.32951204	5.07E-06	4.64E-05
**ENSG00000116001**	TIA1	-0.560355709	7.244973153	27.22751097	5.07E-06	4.64E-05
**ENSG00000178338**	ZNF354B	-0.568994725	4.835708083	25.32694298	5.07E-06	4.64E-05
**ENSG00000111912**	NCOA7	0.360390051	6.67760403	25.32506419	5.08E-06	4.64E-05
**ENSG00000131018**	SYNE1	0.350391976	8.823423403	25.29981082	5.12E-06	4.68E-05

**ENSG00000179715**	PCED1B	-0.37207463	6.712040915	25.29555123	5.13E-06	4.68E-05
**ENSG00000186088**	GSAP	-0.7236258	5.234752059	25.72563637	5.22E-06	4.76E-05
**ENSG00000100300**	TSPO	0.84733572	3.591242192	25.23749032	5.24E-06	4.77E-05
**ENSG00000234961**		0.406224289	8.264958058	25.67453769	5.25E-06	4.78E-05
**ENSG00000205758**	CRYZL1	-0.5707573	5.351848617	25.22682717	5.26E-06	4.78E-05
**ENSG00000254788**	CKLF-CMTM1	0.664241455	4.448120974	25.21672822	5.28E-06	4.79E-05
**ENSG00000109133**	TMEM33	0.403835715	6.930970655	25.21586049	5.28E-06	4.79E-05
**ENSG00000259834**		0.342405232	7.656492239	25.21557474	5.28E-06	4.79E-05
**ENSG00000089053**	ANAPC5	-0.453528546	7.20330558	25.73859449	5.32E-06	4.82E-05
**ENSG00000155368**	DBI	0.676348605	4.833246691	25.18387536	5.34E-06	4.84E-05
**ENSG00000127980**	PEX1	-0.520417454	5.99312705	25.14847771	5.41E-06	4.89E-05
**ENSG00000255302**	EID1	0.491926764	6.47416789	25.39614591	5.42E-06	4.89E-05
**ENSG00000241058**	NSUN6	-0.627672571	5.163681584	25.13631949	5.43E-06	4.91E-05
**ENSG00000176658**	MYO1D	0.679681744	4.340596441	25.11923324	5.47E-06	4.93E-05
**ENSG00000211794**		0.83153445	3.758598837	25.09520736	5.52E-06	4.97E-05
**ENSG00000152332**	UHMK1	0.292204086	8.295436058	25.09283862	5.52E-06	4.97E-05
**ENSG00000082701**	GSK3B	0.366164766	6.81581239	25.09240834	5.52E-06	4.97E-05
**ENSG00000174574**	AKIRIN1	0.350588401	6.678210113	25.08307192	5.54E-06	4.98E-05
**ENSG00000204650**	CRHR1-IT1	-0.529614648	5.220418233	25.08183263	5.54E-06	4.98E-05
**ENSG00000226945**		-0.913141638	3.707324106	25.07511768	5.56E-06	4.99E-05
**ENSG00000198431**	TXNRD1	0.446827336	5.45594534	25.05326494	5.6E-06	5.02E-05
**ENSG00000188404**	SELL	-0.415317897	8.734160851	25.67272555	5.6E-06	5.02E-05
**ENSG00000248124**		-0.667267482	6.02523209	26.60007715	5.61E-06	5.02E-05
**ENSG00000170113**	NIPA1	0.474152364	5.084915249	25.04888399	5.61E-06	5.02E-05
**ENSG00000100097**	LGALS1	0.957857697	4.331154123	25.32057399	5.65E-06	5.05E-05
**ENSG00000106609**	TMEM248	0.360211882	7.052289953	25.02469891	5.66E-06	5.05E-05
**ENSG00000163492**	CCDC141	-0.797625608	5.673808363	26.97386112	5.67E-06	5.06E-05
**ENSG00000182841**	RRP7BP	-0.676651854	4.740091103	24.99159318	5.73E-06	5.1E-05
**ENSG00000214765**	SEPT7P2	-0.642293011	5.134519833	24.98854678	5.73E-06	5.11E-05
**ENSG00000142303**	ADAMTS10	-0.982373044	4.072472546	25.10421124	5.77E-06	5.13E-05
**ENSG00000160856**	FCRL3	0.753024564	4.912920973	25.19362034	5.81E-06	5.17E-05
**ENSG00000111801**	BTN3A3	-0.368350531	7.143713447	24.92873381	5.86E-06	5.2E-05
**ENSG00000140199**	SLC12A6	-0.289421234	7.516824451	24.92699405	5.86E-06	5.2E-05
**ENSG00000125351**	UPF3B	-0.674385564	4.820230381	24.84883742	6.03E-06	5.35E-05
**ENSG00000133773**	CCDC59	-0.489856295	5.833217722	24.8195946	6.09E-06	5.4E-05
**ENSG00000180376**	CCDC66	-0.336325506	7.034213177	24.81083843	6.11E-06	5.41E-05
**ENSG00000133835**	HSD17B4	-0.473529388	5.52161046	24.80643549	6.12E-06	5.42E-05

**ENSG00000106415**	GLCCI1	0.433824417	6.346856143	24.78000897	6.18E-06	5.46E-05
**ENSG00000136738**	STAM	0.399648847	6.191299363	24.76128076	6.22E-06	5.5E-05
**ENSG00000171824**	EXOSC10	-0.391363265	6.570592726	24.75823992	6.23E-06	5.5E-05
**ENSG00000163376**	KBTBD8	0.759409662	3.941494396	24.73211789	6.29E-06	5.55E-05
**ENSG00000133816**	MICAL2	0.628630039	5.284966692	24.68631846	6.39E-06	5.63E-05
**ENSG00000116852**	KIF21B	0.342509251	7.406145169	24.65652462	6.46E-06	5.69E-05
**ENSG00000139697**	MIR8072	0.293698187	7.877532449	24.63178134	6.52E-06	5.74E-05
**ENSG00000131368**	MRPS25	-0.48772161	6.166690468	24.61984462	6.55E-06	5.75E-05
**ENSG00000206530**	CFAP44	-0.786840943	5.18749971	25.60962326	6.55E-06	5.75E-05
**ENSG00000100083**	GGA1	-0.527560988	5.329426889	24.61358301	6.57E-06	5.76E-05
**ENSG00000075240**	GRAMD4	0.942120714	3.501188247	24.57234885	6.66E-06	5.84E-05
**ENSG00000141506**	PIK3R5	0.370269466	6.818566554	24.57233065	6.66E-06	5.84E-05
**ENSG00000186908**	ZDHHC17	-0.490062661	6.198588195	24.52648815	6.78E-06	5.93E-05
**ENSG00000139116**	KIF21A	0.554150562	5.622946937	24.44372911	6.98E-06	6.11E-05
**ENSG00000142327**		0.633509782	6.591259435	26.35646869	7E-06	6.11E-05
**ENSG00000182095**	TNRC18	0.482268284	5.55237473	24.42881221	7.02E-06	6.13E-05
**ENSG00000178028**	DMAP1	-0.64886515	5.524391643	24.86167103	7.02E-06	6.13E-05
**ENSG00000243811**	APOBEC3D	0.533723905	5.245167467	24.38808391	7.13E-06	6.21E-05
**ENSG00000134146**	DPH6	-0.826474735	4.0261449	24.37647797	7.16E-06	6.23E-05
**ENSG00000162231**	NXF1	-0.469097722	6.793859145	24.73110831	7.24E-06	6.3E-05
**ENSG00000107290**	SETX	0.278609995	8.756017841	24.33688847	7.26E-06	6.31E-05
**ENSG00000080822**	CLDND1	0.414559336	7.294430665	24.56144632	7.28E-06	6.32E-05
**ENSG00000253861**		0.604167231	4.72574997	24.31287926	7.32E-06	6.36E-05
**ENSG00000084112**	SSH1	0.47743243	5.679716691	24.30001229	7.36E-06	6.38E-05
**ENSG00000101019**	UQCC1	-0.61664143	4.831643147	24.29441469	7.37E-06	6.38E-05
**ENSG00000187741**	FANCA	-0.428976774	6.2206526	24.29394825	7.38E-06	6.38E-05
**ENSG00000134046**	MBD2	0.37215451	7.02411145	24.28980708	7.39E-06	6.39E-05
**ENSG00000174004**	NRROS	0.767734858	3.790806468	24.28606653	7.4E-06	6.39E-05
**ENSG00000102901**	CENPT	-0.711326998	5.274092617	24.82766415	7.42E-06	6.4E-05
**ENSG00000125868**	DSTN	0.464454355	5.790867677	24.25814589	7.47E-06	6.45E-05
**ENSG00000018189**	RUFY3	-0.620017706	4.948422532	24.25558516	7.48E-06	6.45E-05
**ENSG00000120129**	DUSP1	-0.776624938	6.023452156	26.50829015	7.5E-06	6.46E-05
**ENSG00000177105**	RHOG	0.596898581	5.22458783	24.2354871	7.53E-06	6.48E-05
**ENSG00000080824**	HSP90AA1	0.363281111	9.09257743	24.23531954	7.53E-06	6.48E-05
**ENSG00000178974**	fbxo34	0.504470669	5.585085488	24.22054024	7.58E-06	6.51E-05
**ENSG00000170348**	TMED10	0.357524144	7.835938614	24.2130305	7.6E-06	6.52E-05
**ENSG00000258407**		0.816136802	4.309694388	24.20224208	7.63E-06	6.54E-05

**ENSG00000080345**	RIF1	0.296753354	8.043329475	24.1952415	7.65E-06	6.55E-05
**ENSG00000280064**		0.944783643	4.308670649	24.65169838	7.7E-06	6.59E-05
**ENSG00000170027**	YWHAG	0.410860145	6.101768874	24.13973397	7.8E-06	6.68E-05
**ENSG00000145029**	NICN1	-0.962156587	3.7297132	24.09427781	7.93E-06	6.78E-05
**ENSG00000143799**	PARP1	0.342606611	6.993356328	24.08670712	7.96E-06	6.8E-05
**ENSG00000111011**	RSRC2	-0.371860067	7.563884908	24.0759364	7.99E-06	6.82E-05
**ENSG00000196696**	PDXDC2P	-0.701139421	5.178634617	24.42620048	8E-06	6.82E-05
**ENSG00000197694**	SPTAN1	0.273660959	9.484248535	24.06496042	8.02E-06	6.83E-05
**ENSG00000155330**	c16orf87	0.591294862	4.337979592	24.06383696	8.02E-06	6.83E-05
**ENSG00000232295**		0.836608193	3.810524787	24.04284759	8.08E-06	6.88E-05
**ENSG00000189050**	RNFT1	-0.589012033	4.858034785	24.02285011	8.14E-06	6.92E-05
**ENSG00000133318**	RTN3	0.464117017	5.672130584	24.0192329	8.15E-06	6.93E-05
**ENSG00000211751**		0.34009964	7.903630123	23.97637357	8.28E-06	7.03E-05
**ENSG00000125910**	S1PR4	0.642764167	5.017446866	23.97275878	8.29E-06	7.03E-05
**ENSG00000280734**		-0.92956428	3.745042703	23.95904613	8.34E-06	7.06E-05
**ENSG00000212232**	Snord17	1.822755096	5.865594643	26.85638889	8.4E-06	7.11E-05
**ENSG00000102781**	KATNAL1	0.678204161	4.831307847	23.93326282	8.41E-06	7.12E-05
**ENSG00000163516**	ANKZF1	-0.896715089	6.336536644	26.72491057	8.43E-06	7.12E-05
**ENSG00000180644**	PRF1	1.062414225	5.776009157	26.72584833	8.48E-06	7.16E-05
**ENSG00000135387**	CAPRIN1	0.259677622	8.057242604	23.89503247	8.53E-06	7.2E-05
**ENSG00000122188**	LAX1	0.452753228	5.847830457	23.8778965	8.59E-06	7.24E-05
**ENSG00000164073**	MFSD8	-0.557609347	5.898810746	24.19921946	8.6E-06	7.25E-05
**ENSG00000162511**	LAPTM5	0.331198579	9.537797905	23.85145995	8.67E-06	7.3E-05
**ENSG00000158470**	B4GALT5	0.761905551	4.583619103	23.81822629	8.78E-06	7.38E-05
**ENSG00000040633**	PHF23	0.604967441	4.392195386	23.79433005	8.85E-06	7.44E-05
**ENSG00000180822**	PSMG4	-0.556598738	5.089091853	23.7914702	8.86E-06	7.44E-05
**ENSG00000176171**	BNIP3	-0.557291982	5.084793649	23.78752087	8.88E-06	7.45E-05
**ENSG00000061273**	HDAC7	-0.511628217	6.500353582	24.39090635	8.91E-06	7.47E-05
**ENSG00000135205**	CCDC146	-0.649527611	4.552474105	23.7628837	8.96E-06	7.5E-05
**ENSG00000280077**		-0.635645044	4.235152491	23.76136364	8.96E-06	7.5E-05
**ENSG00000143933**	CALM2	0.346887867	7.468573483	23.74402585	9.02E-06	7.55E-05
**ENSG00000170006**	TMEM154	0.410058503	6.252440464	23.72133838	9.1E-06	7.6E-05
**ENSG00000227671**		-0.653168651	6.200197883	25.32805888	9.15E-06	7.64E-05
**ENSG00000130844**	ZNF331	-0.547808636	5.178962159	23.69167112	9.19E-06	7.67E-05
**ENSG00000172775**	FAM192A	-0.391757226	6.185881574	23.64475436	9.35E-06	7.8E-05
**ENSG00000170275**	CRTAP	0.456605284	5.823718198	23.6298267	9.41E-06	7.84E-05
**ENSG00000198001**	IRAK4	-0.406221235	6.547873165	23.62243594	9.43E-06	7.85E-05

**ENSG00000197321**	svil	-0.362680506	6.46890466	23.61768913	9.45E-06	7.86E-05
**ENSG00000213965**	NUDT19	0.595800611	4.450090462	23.58482894	9.56E-06	7.95E-05
**ENSG00000130024**	PHF10	-0.32087375	7.260523091	23.57666219	9.59E-06	7.97E-05
**ENSG00000175455**	CCDC14	-0.694241455	6.300594945	25.57949741	9.67E-06	8.02E-05
**ENSG00000085982**	USP40	-0.700773018	4.638870873	23.53399664	9.74E-06	8.08E-05
**ENSG00000151422**	FER	-0.634928337	4.557016316	23.53303107	9.75E-06	8.08E-05
**ENSG00000153201**	RANBP2	0.273707117	8.515059377	23.52925444	9.76E-06	8.08E-05
**ENSG00000051108**	HERPUD1	0.415237145	6.007900591	23.50854809	9.84E-06	8.14E-05
**ENSG00000100567**	PSMA3	-0.375674471	6.688176807	23.49581566	9.88E-06	8.17E-05
**ENSG00000143224**	PPOX	-0.811310272	4.00920991	23.49281473	9.89E-06	8.17E-05
**ENSG00000230590**	FTX	-0.430232717	7.738617133	24.27590017	9.95E-06	8.21E-05
**ENSG00000116983**	HPCAL4	-0.732391479	4.505808918	23.463562	1E-05	8.25E-05
**ENSG00000124209**	rab22a	0.37666366	6.642999081	23.42737221	1.01E-05	8.35E-05
**ENSG00000162607**	USP1	0.429230102	6.659398737	23.4269446	1.01E-05	8.35E-05
**ENSG00000223705**	NSUN5P1	-1.027103178	5.085946316	25.59979322	1.01E-05	8.35E-05
**ENSG00000167785**	ZNF558	-0.684947153	4.778655947	23.40675631	1.02E-05	8.39E-05
**ENSG00000130787**	HIP1R	-0.674459748	4.955043137	23.40670016	1.02E-05	8.39E-05
**ENSG00000079974**	RABL2B	-0.655394897	4.744509228	23.4038021	1.02E-05	8.39E-05
**ENSG00000131873**	CHSY1	0.651543055	4.738144615	23.40080949	1.02E-05	8.39E-05
**ENSG00000023445**	BIRC3	-0.408131714	8.520627382	24.01911371	1.03E-05	8.44E-05
**ENSG00000178498**	DTX3	-0.915898801	3.489977647	23.37280261	1.03E-05	8.47E-05
**ENSG00000171943**	SRGAP2C	0.866964457	3.859229202	23.33084689	1.05E-05	8.59E-05
**ENSG00000217555**	cklf	0.592538508	4.650661189	23.30512114	1.06E-05	8.66E-05
**ENSG00000108669**	CYTH1	-0.266136867	8.374377303	23.30335326	1.06E-05	8.66E-05
**ENSG00000102125**	TAZ	-0.844734208	4.583480241	23.6551663	1.06E-05	8.66E-05
**ENSG00000244754**	N4BP2L2	-0.33038774	8.677170004	23.27627326	1.07E-05	8.74E-05
**ENSG00000116741**	RGS2	0.850712908	3.852115849	23.26175827	1.08E-05	8.78E-05
**ENSG00000230124**	LHX4-AS1	-0.550891889	5.253036082	23.25906713	1.08E-05	8.78E-05
**ENSG00000166341**	DCHS1	-0.775941184	4.627036875	23.33778515	1.08E-05	8.82E-05
**ENSG00000127511**	SIN3B	-0.598350136	5.681689284	23.63461328	1.09E-05	8.85E-05
**ENSG00000005893**	LAMP2	0.330895955	6.465905252	23.22768949	1.09E-05	8.86E-05
**ENSG00000127951**	FGL2	0.620031858	4.900618261	23.21923036	1.09E-05	8.89E-05
**ENSG00000174227**	PIGG	-0.522461799	5.790739019	23.20542562	1.1E-05	8.92E-05
**ENSG00000186130**	ZBTB6	0.527971734	5.519899598	23.14472281	1.13E-05	9.12E-05
**ENSG00000137767**	SQRDL	0.620217268	4.868531077	23.14196598	1.13E-05	9.12E-05
**ENSG00000171681**	ATF7IP	0.24783838	8.992066208	23.12013443	1.14E-05	9.19E-05
**ENSG00000179909**	ZNF154	-0.874443053	4.339142798	23.10291629	1.14E-05	9.24E-05

**ENSG00000066855**	MTFR1	-0.421297728	6.032232876	23.09709046	1.15E-05	9.25E-05
**ENSG00000185324**	CDK10	-0.712616582	5.017802182	23.32186444	1.15E-05	9.28E-05
**ENSG00000186432**	KPNA4	0.311179547	7.358239574	23.07637005	1.15E-05	9.31E-05
**ENSG00000101966**	XIAP	0.346571994	6.815128512	23.04724835	1.17E-05	9.4E-05
**ENSG00000131067**	GGT7	-0.947102728	4.067729642	23.19485502	1.17E-05	9.4E-05
**ENSG00000272053**		0.791225284	3.65623053	23.01853981	1.18E-05	9.49E-05
**ENSG00000135945**	REV1	-0.399526694	6.43285866	22.98664759	1.19E-05	9.6E-05
**ENSG00000204217**	BMPR2	0.437629634	6.356758024	22.9817426	1.2E-05	9.61E-05
**ENSG00000156171**	DRAM2	-0.430509796	5.569947169	22.97597678	1.2E-05	9.62E-05
**ENSG00000167615**	LENG8	-1.036570322	8.146518712	25.58365588	1.23E-05	9.87E-05
**ENSG00000168785**	TSPAN5	0.596562977	4.737015195	22.90261644	1.23E-05	9.87E-05
**ENSG00000104904**	OAZ1	0.356236462	6.902349769	22.90050714	1.23E-05	9.87E-05
**ENSG00000026036**	RTEL1-TNFRSF6	-0.671203227	4.713103831	22.83640323	1.26E-05	0.0001009
**ENSG00000139187**	KLRG1	0.704920698	5.567220841	23.94701988	1.26E-05	0.0001009
**ENSG00000271383**	LOC100996717	-0.421907465	5.901909433	22.83191008	1.26E-05	0.0001010
**ENSG00000166507**	NDST2	-0.423708976	6.072641892	22.82404664	1.27E-05	0.0001012
**ENSG00000124570**	SERPINB6	-0.655867386	4.425934946	22.74166133	1.31E-05	0.0001043
**ENSG00000176953**	MIR4517	-0.545762742	6.325219398	23.56753427	1.31E-05	0.0001044
**ENSG00000126790**	L3HYPDH	-0.79758456	4.187243442	22.71025381	1.32E-05	0.0001053
**ENSG00000149428**	HYOU1	0.461912686	5.766904531	22.70712215	1.32E-05	0.0001054
**ENSG00000178802**	MPI	-0.387097338	6.102074424	22.67272214	1.34E-05	0.0001067
**ENSG00000111361**	EIF2B1	-0.487536127	5.638306294	22.66452797	1.34E-05	0.0001069
**ENSG00000124181**	PLCG1	-0.347296233	8.052480549	22.65261144	1.35E-05	0.0001073
**ENSG00000106733**	NMRK1	-0.448692334	5.842275717	22.64836994	1.35E-05	0.0001074
**ENSG00000187514**	MIR1244-1	0.3840625	9.200961363	22.97372629	1.35E-05	0.0001074
**ENSG00000110665**	C11orf21	-0.576968049	5.334989712	22.64039365	1.36E-05	0.0001075
**ENSG00000204165**	CXorf65	-0.795836027	3.88486509	22.63638084	1.36E-05	0.0001076
**ENSG00000101474**	APMAP	0.587023398	5.125451964	22.63347703	1.36E-05	0.0001077
**ENSG00000051009**	fam160a2	-0.664662311	4.99230133	22.62468201	1.37E-05	0.0001079
**ENSG00000139974**	SLC38A6	-0.842942577	4.149380822	22.62354976	1.37E-05	0.0001079
**ENSG00000166794**	PPIB	0.444187102	6.412296944	22.60226068	1.38E-05	0.0001087
**ENSG00000010295**	IFFO1	-0.686188144	4.900890424	22.59403517	1.38E-05	0.0001089
**ENSG00000077147**	TM9SF3	0.312115153	7.593678041	22.59046333	1.38E-05	0.0001090
**ENSG00000162980**	ARL5A	0.407677078	6.477832874	22.53523225	1.41E-05	0.0001112
**ENSG00000281691**	RBM5-AS1	-0.519561592	5.639414179	22.47155605	1.45E-05	0.0001138
**ENSG00000003509**	NDUFAF7	-0.626447469	4.99855083	22.46231765	1.45E-05	0.0001141
**ENSG00000145390**	USP53	-0.364090458	6.925677825	22.45755561	1.45E-05	0.0001142

**ENSG00000102178**	UBL4A	0.737108954	4.141750521	22.42875254	1.47E-05	0.0001153
**ENSG00000013441**	CLK1	-0.512215742	8.579621806	24.2425047	1.48E-05	0.0001161
**ENSG00000198771**	RCSD1	0.300465126	7.397703138	22.40673926	1.48E-05	0.0001161
**ENSG00000143401**	ANP32E	0.396042316	7.344039303	22.58620893	1.49E-05	0.0001169
**ENSG00000184588**	PDE4B	0.359035697	6.74895844	22.37336717	1.5E-05	0.0001174
**ENSG00000170185**	USP38	0.390371791	6.32571183	22.36202662	1.51E-05	0.0001178
**ENSG00000198780**	FAM169A	-0.505079711	5.122170896	22.35299282	1.51E-05	0.0001181
**ENSG00000101138**	CSTF1	0.431167807	5.755880859	22.31198044	1.53E-05	0.0001199
**ENSG00000160179**	ABCG1	-0.909784439	3.994431858	22.30796003	1.54E-05	0.0001200
**ENSG00000120690**	ELF1	0.341291558	8.285587071	22.27324468	1.56E-05	0.0001214
**ENSG00000165280**	VCP	0.33581783	6.934066532	22.26994973	1.56E-05	0.0001215
**ENSG00000087589**	cass4	-0.881736035	4.111530118	22.24833939	1.57E-05	0.0001224
**ENSG00000259976**	Mir568	0.34549787	7.183332375	22.24378743	1.57E-05	0.0001225
**ENSG00000099949**	LZTR1	-0.633951648	5.154843082	22.2402167	1.58E-05	0.0001226
**ENSG00000146350**	TBC1D32	-0.722959995	4.69814027	22.23463759	1.58E-05	0.0001227
**ENSG00000215417**	MIR17	-0.733832922	4.505929373	22.22548108	1.58E-05	0.0001231
**ENSG00000184357**	HIST1H1B	0.972863316	3.80244754	22.31966023	1.59E-05	0.0001236
**ENSG00000183486**	MX2	-0.378560391	6.658751884	22.20837648	1.6E-05	0.0001237
**ENSG00000136908**	DPM2	-0.751049829	4.25894618	22.17040801	1.62E-05	0.0001254
**ENSG00000111684**	LPCAT3	-0.526119191	5.129772067	22.1470375	1.63E-05	0.0001264
**ENSG00000126822**	PLEKHG3	0.601259405	5.79298388	22.83777417	1.63E-05	0.0001264
**ENSG00000082458**	DLG3	0.830437537	4.308162567	22.13519537	1.64E-05	0.0001268
**ENSG00000126860**	EVI2A	0.405233681	7.20342077	22.36380508	1.64E-05	0.0001268
**ENSG00000163874**	MIR6732	0.752374206	4.070512324	22.12064821	1.65E-05	0.0001272
**ENSG00000167257**	RNF214	0.475351682	5.832264295	22.11911188	1.65E-05	0.0001272
**ENSG00000144579**	CTDSP1	0.389207079	6.115258072	22.11370252	1.65E-05	0.0001274
**ENSG00000133657**	ATP13A3	0.305017348	7.218533124	22.10895955	1.66E-05	0.0001275
**ENSG00000175548**	ALG10B	-0.589183638	5.021783551	22.07760449	1.68E-05	0.0001290
**ENSG00000168255**	POLR2J3	-0.503349711	5.672884035	22.07237982	1.68E-05	0.0001291
**ENSG00000110876**	SELPLG	0.383738774	8.084279281	22.43409483	1.69E-05	0.0001303
**ENSG00000156875**	MFSD14A	0.382373054	6.173159176	22.04035589	1.7E-05	0.0001305
**ENSG00000100612**	DHRS7	0.47900046	5.657282806	22.01340988	1.72E-05	0.0001317
**ENSG00000258959**		0.477474509	5.093332637	21.98785344	1.73E-05	0.0001329
**ENSG00000038274**	MAT2B	0.363185363	7.24517322	21.98668967	1.73E-05	0.0001329
**ENSG00000107331**	abca2	0.685810155	6.338810759	23.89070316	1.74E-05	0.0001333
**ENSG00000265148**	TSPOAP1-AS1	-0.396751438	6.117028027	21.96495709	1.75E-05	0.0001338
**ENSG00000173960**	UBXN2A	0.60376781	4.578166371	21.95472443	1.75E-05	0.0001342

**ENSG00000232354**		-0.706725931	4.32213092	21.95135374	1.76E-05	0.0001342
**ENSG00000076928**	LOC100505585	-0.678695161	8.296299704	24.29269121	1.79E-05	0.0001363
**ENSG00000076003**	MCM6	0.511928404	5.356153653	21.88958049	1.8E-05	0.0001372
**ENSG00000055208**	TAB2	0.259702216	8.818595912	21.8569354	1.82E-05	0.0001388
**ENSG00000006530**	AGK	-0.388296406	5.713433233	21.85210418	1.82E-05	0.0001390
**ENSG00000109062**	MIR3615	0.385090654	6.664493418	21.81383409	1.85E-05	0.0001409
**ENSG00000260170**		0.620968121	4.414530401	21.81058303	1.85E-05	0.0001410
**ENSG00000070718**	AP3M2	-0.49333236	5.880961159	21.78873016	1.87E-05	0.0001419
**ENSG00000221962**		-0.984454487	5.137042108	23.73155305	1.87E-05	0.0001419
**ENSG00000164828**	SUN1	-0.46202432	6.550249954	22.05944354	1.87E-05	0.0001421
**ENSG00000139083**	ETV6	0.402029658	5.955104563	21.77311089	1.88E-05	0.0001425
**ENSG00000125430**	HS3ST3B1	0.455507836	5.978179541	21.75140478	1.89E-05	0.0001436
**ENSG00000260304**		-0.464274878	5.494440327	21.7394293	1.9E-05	0.0001441
**ENSG00000109920**	FNBP4	-0.559485755	7.963235803	23.75729845	1.9E-05	0.0001441
**ENSG00000107890**	ANKRD26	-0.548248007	5.52900701	21.73094286	1.91E-05	0.0001444
**ENSG00000185591**	SP1	0.284889777	7.666933568	21.69157647	1.94E-05	0.0001465
**ENSG00000214135**	LOC220729	-0.741568764	5.288687996	22.62112697	1.94E-05	0.0001465
**ENSG00000120896**	SORBS3	-0.702469907	4.417353755	21.67863802	1.95E-05	0.0001470
**ENSG00000072818**	ACAP1	-0.544981411	7.443615544	23.44671837	1.96E-05	0.0001475
**ENSG00000049883**	PTCD2	-0.68052242	4.4994568	21.62902646	1.98E-05	0.0001495
**ENSG00000184465**	WDR27	-0.862282083	5.174300876	23.09105572	1.99E-05	0.0001498
**ENSG00000172081**	MOB3A	0.393159017	7.515277696	21.95686188	2E-05	0.0001504
**ENSG00000130254**	SAFB2	-0.329827574	6.796754496	21.58321313	2.02E-05	0.0001518
**ENSG00000184992**	BRI3BP	0.653819333	4.665428855	21.56983905	2.03E-05	0.0001525
**ENSG00000142864**	SERBP1	0.273101389	8.114360968	21.55395751	2.04E-05	0.0001533
**ENSG00000186812**	ZNF397	-0.480038646	5.93174424	21.53969459	2.05E-05	0.0001540
**ENSG00000155252**	PI4K2A	0.717010132	3.723761435	21.51340304	2.07E-05	0.0001555
**ENSG00000149292**	TTC12	-0.586340291	5.229461602	21.50897106	2.08E-05	0.0001556
**ENSG00000138698**	RAP1GDS1	0.376070141	6.073472391	21.50416881	2.08E-05	0.0001558
**ENSG00000161010**	MRNIP	-0.352773648	6.470306169	21.49659598	2.09E-05	0.0001561
**ENSG00000150961**	SEC24D	0.468163104	5.349885195	21.49482434	2.09E-05	0.0001561
**ENSG00000147202**	DIAPH2	0.456331192	5.48631203	21.46845288	2.11E-05	0.0001576
**ENSG00000157181**	C1orf27	-0.567981157	5.156695495	21.4643337	2.11E-05	0.0001577
**ENSG00000162368**	CMPK1	0.35589372	7.73485977	21.50492491	2.12E-05	0.0001579
**ENSG00000135801**	TAF5L	0.540084529	4.813822885	21.4451464	2.13E-05	0.0001586
**ENSG00000160584**	SIK3	0.287048471	6.860242771	21.44105011	2.13E-05	0.0001588
**ENSG00000112308**	c6orf62	0.252955311	8.083404356	21.39983815	2.16E-05	0.0001612

**ENSG00000138757**	G3BP2	0.322176795	7.640972742	21.39635011	2.17E-05	0.0001612
**ENSG00000167264**	dus2	-0.76794859	3.939282118	21.38908782	2.17E-05	0.0001616
**ENSG00000065613**	SLK	0.329063459	7.584126033	21.38535741	2.18E-05	0.0001617
**ENSG00000087460**	GNAS	0.265915697	8.947489574	21.3798621	2.18E-05	0.0001619
**ENSG00000100722**	ZC3H14	-0.358125092	6.451993968	21.36318008	2.19E-05	0.0001628
**ENSG00000110074**	FOXRED1	-0.823811304	3.671821659	21.36209509	2.2E-05	0.0001628
**ENSG00000100614**	PPM1A	0.330271159	6.775151874	21.33318295	2.22E-05	0.0001645
**ENSG00000108559**	NUP88	-0.337187018	6.431472976	21.29728307	2.25E-05	0.0001666
**ENSG00000172578**	KLHL6	-0.337978894	6.669640186	21.29581652	2.25E-05	0.0001666
**ENSG00000251474**	RPL32P3	-0.618255319	4.926517405	21.28935455	2.26E-05	0.0001669
**ENSG00000175348**	TMEM9B	0.512664054	5.310274794	21.28463779	2.26E-05	0.0001671
**ENSG00000136986**	DERL1	0.384664635	6.250941261	21.27898155	2.27E-05	0.0001673
**ENSG00000142173**	COL6A2	-0.874560873	3.84890024	21.27683365	2.27E-05	0.0001673
**ENSG00000160321**	znf208	-0.867957839	4.107791333	21.26673441	2.28E-05	0.0001678
**ENSG00000156136**	DCK	0.38491405	6.552159205	21.26594466	2.28E-05	0.0001678
**ENSG00000169490**	TM2D2	0.632590363	4.267839831	21.26263928	2.28E-05	0.0001679
**ENSG00000180198**	RCC1	-0.681110959	4.759795126	21.23948255	2.3E-05	0.0001691
**ENSG00000028310**	BRD9	-0.458001052	6.180780617	21.23704521	2.3E-05	0.0001691
**ENSG00000164938**	TP53INP1	0.458090634	6.633115784	21.60333202	2.3E-05	0.0001691
**ENSG00000144635**	DYNC1LI1	0.422842957	5.309029976	21.23360775	2.31E-05	0.0001691
**ENSG00000172725**	CORO1B	0.465543049	6.545861622	21.61495131	2.31E-05	0.0001691
**ENSG00000167113**	COQ4	-0.797754912	4.437503857	21.40615143	2.31E-05	0.0001691
**ENSG00000166340**	TPP1	0.320439284	7.376319577	21.22492935	2.31E-05	0.0001695
**ENSG00000008294**	SPAG9	0.332147721	6.827982798	21.19476669	2.34E-05	0.0001713
**ENSG00000102096**	PIM2	-0.408860398	7.195587709	21.50921782	2.37E-05	0.0001731
**ENSG00000163041**	H3F3A	-0.442821748	5.910912064	21.1448593	2.38E-05	0.0001743
**ENSG00000183718**	TRIM52	-0.387369342	6.483568055	21.1328676	2.4E-05	0.0001750
**ENSG00000102921**	N4BP1	0.364891616	6.619684947	21.12875053	2.4E-05	0.0001750
**ENSG00000166888**	STAT6	-0.37229501	7.061101392	21.12814194	2.4E-05	0.0001750
**ENSG00000107614**	TRDMT1	-0.59735375	5.493527264	21.37978296	2.4E-05	0.0001750
**ENSG00000203880**	PCMTD2	-0.344546088	6.921972753	21.08829604	2.44E-05	0.0001775
**ENSG00000115310**	RTN4	0.305733466	6.650959462	21.08591513	2.44E-05	0.0001776
**ENSG00000171302**	CANT1	0.465307608	4.89162861	21.07134872	2.45E-05	0.0001784
**ENSG00000225828**	FAM229A	-0.81228361	4.179721956	21.04362645	2.48E-05	0.0001802
**ENSG00000140943**	MBTPS1	-0.294613924	7.018408397	21.04101722	2.48E-05	0.0001802
**ENSG00000157734**	SNX22	0.446264507	5.98795199	21.02896515	2.49E-05	0.0001810
**ENSG00000172531**	PPP1CA	0.436037665	5.86666743	21.02146118	2.5E-05	0.0001813

**ENSG00000083168**	KAT6A	0.276027616	8.583509448	21.01714925	2.5E-05	0.0001815
**ENSG00000234420**	ZNF37BP	-0.584913384	6.303198118	22.21284564	2.51E-05	0.0001816
**ENSG00000125304**	TM9SF2	0.318813257	7.017775487	20.99986094	2.52E-05	0.0001825
**ENSG00000198740**	ZNF652	0.253146719	7.259875085	20.95171607	2.57E-05	0.0001857
**ENSG00000188732**	FAM221A	-0.789370583	3.711607315	20.95007451	2.57E-05	0.0001857
**ENSG00000119760**	SUPT7L	-0.489330404	6.03486189	21.11663367	2.57E-05	0.0001858
**ENSG00000142347**	MYO1F	0.683066667	6.935687357	22.97418659	2.62E-05	0.0001890
**ENSG00000102053**	zc3h12b	-0.866842054	3.840338121	20.89505693	2.62E-05	0.0001893
**ENSG00000198734**	F5	0.600161611	5.917867761	21.80043667	2.64E-05	0.0001900
**ENSG00000164086**	DUSP7	0.480100673	5.928555327	20.87763761	2.64E-05	0.0001903
**ENSG00000184083**	FAM120C	-0.791087708	4.178869668	20.85876469	2.66E-05	0.0001915
**ENSG00000165275**	TRMT10B	-0.558585332	5.240333898	20.85388985	2.66E-05	0.0001917
**ENSG00000196187**	TMEM63A	-0.502006394	8.393429892	22.49647278	2.67E-05	0.0001918
**ENSG00000228327**		-0.679882149	3.885504998	20.83740061	2.68E-05	0.0001927
**ENSG00000104447**	TRPS1	0.427861107	5.880983098	20.83244099	2.69E-05	0.0001929
**ENSG00000247315**	ZCCHC3	0.560502125	4.993884897	20.83159243	2.69E-05	0.0001929
**ENSG00000196843**	ARID5A	0.651039907	4.537615297	20.82706598	2.69E-05	0.0001931
**ENSG00000111786**	SRSF9	-0.338867018	6.240876596	20.79225563	2.73E-05	0.0001953
**ENSG00000132825**	PPP1R3D	0.58745841	4.265570657	20.79124724	2.73E-05	0.0001953
**ENSG00000132300**	PTCD3	-0.396088139	7.291843929	21.0764356	2.73E-05	0.0001953
**ENSG00000162924**	REL	0.378526518	6.868956592	20.78669478	2.73E-05	0.0001955
**ENSG00000054523**	KIF1B	0.477774715	5.760269898	20.78557402	2.74E-05	0.0001955
**ENSG00000128159**	TUBGCP6	-0.695789349	6.365018785	22.61417853	2.75E-05	0.0001961
**ENSG00000110013**	SIAE	-0.556829992	4.939773174	20.77274085	2.75E-05	0.0001962
**ENSG00000140941**	MAP1LC3B	0.399201496	6.167683705	20.76602499	2.76E-05	0.0001965
**ENSG00000072756**	TRNT1	-0.376386216	6.434491993	20.75024602	2.77E-05	0.0001976
**ENSG00000197471**	SPN	0.434312809	8.248633766	21.79460108	2.78E-05	0.0001979
**ENSG00000147533**	GOLGA7	0.359804554	6.375176762	20.7172013	2.81E-05	0.0001999
**ENSG00000182472**	CAPN12	1.058001741	4.92123923	22.53070042	2.86E-05	0.0002035
**ENSG00000281896**		-0.608473402	4.679880886	20.65404634	2.88E-05	0.0002044
**ENSG00000153113**	CAST	0.267912117	8.721797923	20.65362029	2.88E-05	0.0002044
**ENSG00000138166**	DUSP5	0.883624163	3.734140134	20.63336272	2.9E-05	0.0002058
**ENSG00000114841**	DNAH1	-0.738998794	7.035946038	22.75774477	2.9E-05	0.0002058
**ENSG00000140564**	FURIN	0.658174357	4.56731201	20.62585656	2.91E-05	0.0002060
**ENSG00000137266**	SLC22A23	-0.59222921	4.995807167	20.62451882	2.91E-05	0.0002060
**ENSG00000113407**	TARS	0.538654151	5.834962497	20.97932111	2.91E-05	0.0002060
**ENSG00000104228**	TRIM35	0.487432454	5.416765451	20.62219886	2.91E-05	0.0002060

**ENSG00000167613**	LAIR1	-0.496019143	5.674168257	20.6137143	2.92E-05	0.0002064
**ENSG00000119242**	CCDC92	0.407931616	5.553825584	20.61369239	2.92E-05	0.0002064
**ENSG00000197081**	IGF2R	0.294167897	7.93111389	20.58921865	2.95E-05	0.0002082
**ENSG00000272501**		-0.783051925	4.178724175	20.54772207	3E-05	0.0002114
**ENSG00000080371**	RAB21	0.338365119	7.230592093	20.5383805	3.01E-05	0.0002120
**ENSG00000110723**	EXPH5	-0.862938773	3.908691928	20.50794806	3.04E-05	0.0002144
**ENSG00000160551**	MIR4523	0.275625844	8.119962305	20.48483654	3.07E-05	0.0002161
**ENSG00000187650**	VMAC	-0.831192135	4.165247591	20.48365778	3.07E-05	0.0002161
**ENSG00000176444**	CLK2	-0.473057198	5.951402118	20.47905506	3.08E-05	0.0002163
**ENSG00000139921**	TMX1	0.405968649	6.034199631	20.47708176	3.08E-05	0.0002164
**ENSG00000113851**	CRBN	-0.311259411	7.723260329	20.45218077	3.11E-05	0.0002183
**ENSG00000234127**	TRIM26	0.492902422	5.294887585	20.44833245	3.11E-05	0.0002185
**ENSG00000144029**	MRPS5	-0.400367325	5.7837125	20.4462075	3.12E-05	0.0002185
**ENSG00000172890**	NADSYN1	-0.497862175	6.240005821	20.89716787	3.14E-05	0.0002198
**ENSG00000067167**	TRAM1	0.314678677	7.942457014	20.42098124	3.15E-05	0.0002204
**ENSG00000113441**	LNPEP	0.253779436	9.281568513	20.41647305	3.15E-05	0.0002206
**ENSG00000153898**	MCOLN2	0.924677273	4.436402983	21.19633703	3.18E-05	0.0002220
**ENSG00000077454**	lrch4	-0.569419418	6.475447138	21.60698715	3.18E-05	0.0002220
**ENSG00000093000**	NUP50	0.284191821	7.592052043	20.39522409	3.18E-05	0.0002220
**ENSG00000169018**	FEM1B	0.291141851	6.94347661	20.38972053	3.18E-05	0.0002222
**ENSG00000269821**	KCNQ1OT1	-0.903456145	6.708560527	22.51922394	3.19E-05	0.0002222
**ENSG00000177082**	WDR73	-0.563488534	6.273181223	21.42078801	3.2E-05	0.0002230
**ENSG00000138031**	ADCY3	0.542055456	4.895808943	20.37173668	3.21E-05	0.0002234
**ENSG00000117394**	SLC2A1	0.463084336	5.144085896	20.35537081	3.23E-05	0.0002246
**ENSG00000229474**	PATL2	-0.640338298	4.739300323	20.3349997	3.25E-05	0.0002263
**ENSG00000166710**	B2M	0.401036591	11.45498781	20.56384585	3.27E-05	0.0002272
**ENSG00000258757**		0.499551693	5.847706013	20.38505388	3.28E-05	0.0002276
**ENSG00000135469**	COQ10A	-0.849243049	3.661331082	20.29175494	3.31E-05	0.0002296
**ENSG00000128284**	apol3	-0.319061633	6.398631752	20.27954288	3.32E-05	0.0002305
**ENSG00000269243**		0.404233046	5.305553292	20.25235548	3.36E-05	0.0002328
**ENSG00000172757**	CFL1	0.425710233	8.272069183	21.22596678	3.36E-05	0.0002329
**ENSG00000167797**	CDK2AP2	0.731010896	4.806700212	20.59115882	3.36E-05	0.0002329
**ENSG00000188997**	KCTD21	0.758695169	3.596769718	20.23481247	3.38E-05	0.0002339
**ENSG00000211793**		0.620339463	4.411328745	20.22355373	3.4E-05	0.0002348
**ENSG00000221978**	CCNL2	-0.839606778	7.258955312	22.30580685	3.42E-05	0.0002362
**ENSG00000134294**	SLC38A2	0.262021263	8.132233267	20.19676908	3.43E-05	0.0002369
**ENSG00000153395**	LPCAT1	0.495600259	5.620496191	20.19163989	3.44E-05	0.0002372

**ENSG00000012822**	CALCOCO1	-0.471525237	6.059176374	20.291838	3.45E-05	0.0002378
**ENSG00000134970**	TMED7	0.461077972	6.30603665	20.41521307	3.45E-05	0.0002380
**ENSG00000272916**	NDST2	-0.392746013	6.40213927	20.17193999	3.46E-05	0.0002385
**ENSG00000167081**	PBX3	-0.6577714	4.421101753	20.16805185	3.47E-05	0.0002385
**ENSG00000245552**	LOC101929295	-0.663114303	4.090746882	20.16795798	3.47E-05	0.0002385
**ENSG00000196227**	FAM217B	0.433977219	5.75042578	20.16743311	3.47E-05	0.0002385
**ENSG00000188227**	ZNF793	-0.722002045	4.483329311	20.16293951	3.48E-05	0.0002388
**ENSG00000159921**	GNE	0.451508492	5.345091686	20.14244597	3.5E-05	0.0002404
**ENSG00000103489**	XYLT1	0.439000607	5.942528664	20.14207636	3.5E-05	0.0002404
**ENSG00000007392**	LUC7L	-0.674378417	6.399475096	21.85634844	3.51E-05	0.0002410
**ENSG00000111737**	RAB35	0.472067976	4.873549898	20.11781385	3.54E-05	0.0002424
**ENSG00000109618**	SEPSECS	-0.522511925	5.072035848	20.11104906	3.55E-05	0.0002428
**ENSG00000273275**		0.521211151	5.122213451	20.10551684	3.55E-05	0.0002432
**ENSG00000110888**	CAPRIN2	-0.567557211	5.952609769	20.80361222	3.58E-05	0.0002448
**ENSG00000196110**	ZNF699	0.571497224	4.451718082	20.07619218	3.59E-05	0.0002456
**ENSG00000204560**	DHX16	-0.494561824	5.337294398	20.06257545	3.61E-05	0.0002468
**ENSG00000112294**	ALDH5A1	-0.538713476	4.781437401	20.05957888	3.62E-05	0.0002469
**ENSG00000180304**	OAZ2	-0.532928602	4.937892252	20.05387712	3.63E-05	0.0002473
**ENSG00000114850**	SSR3	0.372407299	6.62400427	20.02998071	3.66E-05	0.0002494
**ENSG00000160285**	LSS	0.508973116	5.277797276	20.0188048	3.68E-05	0.0002503
**ENSG00000134900**	TPP2	-0.303535582	7.802592564	19.99021352	3.72E-05	0.0002530
**ENSG00000206341**		0.495227605	6.454577555	20.5795503	3.72E-05	0.0002532
**ENSG00000111647**	UHRF1BP1L	0.410987595	5.405722363	19.97681784	3.74E-05	0.0002540
**ENSG00000107938**	EDRF1	-0.430529769	6.31854255	19.97230009	3.74E-05	0.0002542
**ENSG00000072135**	PTPN18	0.385018381	6.168695966	19.96418284	3.75E-05	0.0002549
**ENSG00000130592**	MIR7847	0.315889641	6.662244426	19.95679574	3.77E-05	0.0002554
**ENSG00000164054**	SHISA5	0.423844056	6.751667202	20.19169278	3.77E-05	0.0002559
**ENSG00000105519**	CAPS	-0.710947123	4.413739013	19.94148886	3.79E-05	0.0002566
**ENSG00000171606**	ZNF274	-0.413101912	5.512030278	19.93591992	3.8E-05	0.0002570
**ENSG00000253276**	CCDC71L	0.641282286	4.150096645	19.92693879	3.81E-05	0.0002577
**ENSG00000167005**	NUDT21	0.297067562	6.800191421	19.91706031	3.82E-05	0.0002586
**ENSG00000173852**	DPY19L1	0.606989997	4.715429016	19.91250587	3.83E-05	0.0002589
**ENSG00000125107**	CNOT1	0.225289568	8.988067871	19.90392875	3.84E-05	0.0002596
**ENSG00000091317**	CMTM6	0.42091526	6.432892228	19.89575727	3.86E-05	0.0002603
**ENSG00000213983**	ap1g2	-0.708637536	7.03695576	21.83451762	3.89E-05	0.0002627
**ENSG00000166881**	NEMP1	-0.459648617	5.513446178	19.86122417	3.91E-05	0.0002634
**ENSG00000146409**	SLC18B1	-0.579435565	4.639291761	19.85643745	3.91E-05	0.0002637

**ENSG00000169442**	CD52	0.472754025	8.221975186	21.23141547	3.92E-05	0.0002637
**ENSG00000110172**	CHORDC1	-0.324563487	7.099891181	19.84945452	3.93E-05	0.0002641
**ENSG00000143353**	LYPLAL1	-0.580972144	4.181139894	19.82795567	3.96E-05	0.0002661
**ENSG00000164219**	PGGT1B	-0.42198834	6.055511067	19.81645175	3.98E-05	0.0002671
**ENSG00000115368**	WDR75	-0.35380769	6.521022564	19.8091466	3.99E-05	0.0002677
**ENSG00000204514**	ZNF814	-0.36862844	6.014911186	19.7550534	4.07E-05	0.0002732
**ENSG00000140406**	mesdc1	0.709647211	4.402270993	19.75158175	4.08E-05	0.0002734
**ENSG00000074706**	IPCEF1	-0.319466924	7.446892204	19.73287848	4.11E-05	0.0002751
**ENSG00000143337**	TOR1AIP1	0.309496123	7.120636499	19.73249189	4.11E-05	0.0002751
**ENSG00000258643**	BCL2L2-PABPN1	-0.37741132	5.616103839	19.70653622	4.15E-05	0.0002777
**ENSG00000114770**	ABCC5	-0.558856848	5.362702782	19.7187917	4.16E-05	0.0002780
**ENSG00000254413**	CHKB-CPT1B	-0.825785098	5.976022322	21.55122752	4.17E-05	0.0002787
**ENSG00000253645**		0.77088103	4.002500815	19.6887419	4.18E-05	0.0002791
**ENSG00000213799**	ZNF845	0.471312021	5.743991168	19.66979863	4.21E-05	0.0002810
**ENSG00000119669**	IRF2BPL	0.566383535	4.922857223	19.65348849	4.24E-05	0.0002825
**ENSG00000157514**	TSC22D3	-0.309767528	7.707805708	19.65348077	4.24E-05	0.0002825
**ENSG00000111269**	CREBL2	0.317494139	6.673322787	19.65157066	4.24E-05	0.0002825
**ENSG00000132405**	TBC1D14	0.312237096	6.489578564	19.64601221	4.25E-05	0.0002829
**ENSG00000109971**	HSPA8	0.331378235	9.957015422	19.63680698	4.26E-05	0.0002837
**ENSG00000107672**	NSMCE4A	-0.620513566	5.412752861	20.07979635	4.27E-05	0.0002837
**ENSG00000125249**	RAP2A	0.529371011	5.536336811	19.65888828	4.27E-05	0.0002839
**ENSG00000196199**	MPHOSPH8	-0.287323132	7.776490885	19.60680546	4.31E-05	0.0002865
**ENSG00000170222**	ADPRM	-0.589211898	4.535360695	19.59306266	4.34E-05	0.0002879
**ENSG00000170581**	STAT2	-0.382091007	6.647162365	19.58951583	4.34E-05	0.0002881
**ENSG00000109452**	INPP4B	0.30135727	9.072233705	19.57795399	4.36E-05	0.0002892
**ENSG00000169914**	OTUD3	-0.694323338	4.890049524	19.82214504	4.37E-05	0.0002896
**ENSG00000147454**	SLC25A37	-0.62271382	5.189389633	19.7559733	4.41E-05	0.0002916
**ENSG00000006831**	ADIPOR2	0.430782338	5.388000444	19.55246066	4.41E-05	0.0002916
**ENSG00000104205**	SGK3	0.420691687	5.554667454	19.54683037	4.42E-05	0.0002920
**ENSG00000147162**	OGT	-0.648511257	9.744077679	21.45604988	4.43E-05	0.0002925
**ENSG00000033170**	FUT8	0.402972769	5.914160794	19.5272334	4.45E-05	0.0002939
**ENSG00000258297**		-0.570280772	5.177294463	19.52261334	4.46E-05	0.0002942
**ENSG00000111325**	OGFOD2	-0.757529468	3.708335058	19.51438568	4.47E-05	0.0002950
**ENSG00000114796**	KLHL24	0.323958268	7.263270087	19.47462381	4.54E-05	0.0002994
**ENSG00000176463**	SLCO3A1	0.490950645	5.689766331	19.44206059	4.6E-05	0.0003031
**ENSG00000265206**	MIR142	-0.909240143	5.127683643	20.91446468	4.63E-05	0.0003052
**ENSG00000197622**	CDC42SE1	-0.263585916	8.583731935	19.40850025	4.66E-05	0.0003067

**ENSG00000280828**		-0.489910404	5.417430454	19.38896775	4.7E-05	0.0003088
**ENSG00000144118**	RALB	0.623344616	4.00864142	19.38370891	4.71E-05	0.0003093
**ENSG00000183513**	COA5	-0.562278291	5.002459202	19.36325017	4.74E-05	0.0003114
**ENSG00000272779**		-0.705484182	4.016661083	19.36315598	4.74E-05	0.0003114
**ENSG00000205808**	plpp6	-0.589272279	4.547166558	19.35141882	4.77E-05	0.0003126
**ENSG00000124203**	ZNF831	0.325473791	6.75493366	19.32810449	4.81E-05	0.0003153
**ENSG00000175826**	CTDNEP1	0.39369434	6.224315564	19.32053482	4.82E-05	0.0003160
**ENSG00000145715**	RASA1	-0.318531074	7.327381832	19.31083108	4.84E-05	0.0003170
**ENSG00000142396**	LOC105372481	-0.503433921	5.740045931	19.40637394	4.85E-05	0.0003172
**ENSG00000117640**	MTFR1L	-0.601757732	4.544738805	19.27943397	4.9E-05	0.0003206
**ENSG00000103479**	RBL2	-0.266748444	9.261185301	19.2671213	4.93E-05	0.0003219
**ENSG00000177272**	KCNA3	0.299765351	7.195770984	19.25474242	4.95E-05	0.0003233
**ENSG00000271856**	LINC01215	-0.551459685	4.925704142	19.25013602	4.96E-05	0.0003237
**ENSG00000158062**	UBXN11	0.456817161	7.95829355	20.41947743	5.03E-05	0.0003279
**ENSG00000011114**	BTBD7	0.364773489	6.110502689	19.21097335	5.04E-05	0.0003283
**ENSG00000164331**	ANKRA2	-0.554777424	5.198096267	19.17596082	5.11E-05	0.0003326
**ENSG00000074935**	TUBE1	-0.477380763	5.37120953	19.13326878	5.19E-05	0.0003380
**ENSG00000114857**	NKTR	-0.706662807	9.156192897	21.01095496	5.2E-05	0.0003383
**ENSG00000155380**	SLC16A1	0.578551224	4.561509189	19.12643259	5.21E-05	0.0003385
**ENSG00000196712**	NF1	0.282920918	7.318511884	19.12062062	5.22E-05	0.0003390
**ENSG00000156471**	PTDSS1	0.390640413	6.255295274	19.10737268	5.24E-05	0.0003406
**ENSG00000107738**	c10orf54	0.363408807	6.452788676	19.09100199	5.28E-05	0.0003426
**ENSG00000166704**	ZNF606	-0.744694523	4.266184868	19.08116208	5.3E-05	0.0003437
**ENSG00000266076**		-0.742325461	4.247418615	19.07940341	5.3E-05	0.0003437
**ENSG00000228315**	GUSBP11	-0.811277418	5.85102035	20.78155216	5.31E-05	0.0003441
**ENSG00000162714**	ZNF496	-0.849542543	3.633739874	19.06947442	5.32E-05	0.0003445
**ENSG00000137312**	FLOT1	-0.489633627	4.882945943	19.06866578	5.33E-05	0.0003445
**ENSG00000021300**	PLEKHB1	-0.547212344	4.639532956	19.06358526	5.34E-05	0.0003448
**ENSG00000115977**	AAK1	-0.262911651	9.029380982	19.06344284	5.34E-05	0.0003448
**ENSG00000134250**	NOTCH2	0.272382791	7.273645025	19.03860991	5.39E-05	0.0003480
**ENSG00000142867**	BCL10	0.399869892	5.473143445	19.03118533	5.4E-05	0.0003488
**ENSG00000136213**	LOC101927181	0.535384088	5.398541687	19.01885516	5.43E-05	0.0003503
**ENSG00000106479**	ZNF862	-0.489901059	5.489905569	18.99603525	5.48E-05	0.0003532
**ENSG00000211772**		0.289117156	9.021548029	18.98913909	5.49E-05	0.0003540
**ENSG00000111554**	MDM1	-0.512602208	4.752442686	18.96534347	5.55E-05	0.0003571
**ENSG00000189159**	HN1	0.520013263	5.510966747	18.95833062	5.56E-05	0.0003578
**ENSG00000267458**		0.717408143	3.6235731	18.95676174	5.56E-05	0.0003578

**ENSG00000198618**		0.657335448	4.526746978	18.92769361	5.63E-05	0.0003617
**ENSG00000270127**		-0.752965405	4.518686935	19.0634125	5.64E-05	0.0003622
**ENSG00000116128**	BCL9	-0.536282234	4.943142812	18.90537623	5.68E-05	0.0003645
**ENSG00000103495**	MAZ	0.385953148	6.226793436	18.90353476	5.68E-05	0.0003645
**ENSG00000175550**	DRAP1	0.543151618	4.468112972	18.89367804	5.7E-05	0.0003657
**ENSG00000279765**		-0.323794497	7.761072139	18.87574567	5.75E-05	0.0003681
**ENSG00000122223**	CD244	-0.70940099	3.624850769	18.87284121	5.75E-05	0.0003683
**ENSG00000089820**	ARHGAP4	-0.562926966	6.90742497	20.2828167	5.76E-05	0.0003683
**ENSG00000145416**	36951	-0.62696895	4.420756994	18.84818564	5.81E-05	0.0003714
**ENSG00000123091**	RNF11	0.436577762	5.577825932	18.83597151	5.84E-05	0.0003730
**ENSG00000267121**		-0.918012836	5.02053231	20.24166596	5.9E-05	0.0003770
**ENSG00000204439**	C6orf47	0.678803862	4.259902183	18.79290769	5.94E-05	0.0003789
**ENSG00000167325**	RRM1	0.425705683	5.748551508	18.77199205	5.99E-05	0.0003818
**ENSG00000011007**	TCEB3	0.358498409	5.79342699	18.76230034	6.01E-05	0.0003830
**ENSG00000161547**	SRSF2	-0.492948711	7.472654856	20.04404942	6.03E-05	0.0003841
**ENSG00000109171**	SLAIN2	0.351002946	7.017817432	18.75206239	6.03E-05	0.0003841
**ENSG00000268575**		-0.401829242	6.569048161	18.75381047	6.07E-05	0.0003862
**ENSG00000149483**	TMEM138	-0.489614651	4.939457547	18.72703931	6.09E-05	0.0003874
**ENSG00000265817**	FSBP	-0.876499777	3.658786241	18.72578916	6.1E-05	0.0003874
**ENSG00000182054**	IDH2	0.472073392	5.126059469	18.71607413	6.12E-05	0.0003885
**ENSG00000130023**	ERMARD	-0.608063961	4.833226413	18.71543294	6.12E-05	0.0003885
**ENSG00000169118**	CSNK1G1	0.379295152	5.951364604	18.69677927	6.17E-05	0.0003912
**ENSG00000158615**	PPP1R15B	0.274239804	7.291193117	18.66253264	6.25E-05	0.0003963
**ENSG00000134152**	KATNBL1	-0.461145986	5.201345542	18.62421623	6.35E-05	0.0004021
**ENSG00000100207**	tcf20	0.291180686	7.143285333	18.62149156	6.35E-05	0.0004021
**ENSG00000112245**	PTP4A1	0.349451184	6.84433656	18.62079021	6.35E-05	0.0004021
**ENSG00000160213**	CSTB	0.549520062	5.378714361	18.70850689	6.36E-05	0.0004022
**ENSG00000112486**	CCR6	0.659416413	5.667197773	19.5893472	6.38E-05	0.0004031
**ENSG00000155393**	HEATR3	-0.581995264	4.800683544	18.59362387	6.42E-05	0.0004058
**ENSG00000173575**	MIR3175	-0.251782106	9.059790053	18.57019598	6.48E-05	0.0004093
**ENSG00000277599**		-0.855529231	5.842183382	20.25296335	6.49E-05	0.0004093
**ENSG00000281887**	GIMAP1-GIMAP5	-0.28324083	7.651798082	18.5404894	6.56E-05	0.0004136
**ENSG00000134905**	CARS2	-0.477306472	5.564588846	18.5328609	6.58E-05	0.0004146
**ENSG00000138735**	PDE5A	-0.615833965	5.141655977	18.75116947	6.58E-05	0.0004148
**ENSG00000083223**	ZCCHC6	0.311342198	7.15793867	18.51531143	6.62E-05	0.0004170
**ENSG00000258366**	RTEL1	-0.628839416	4.473016739	18.50169363	6.66E-05	0.0004190
**ENSG00000181915**	ADO	0.518083866	4.570240326	18.48556495	6.7E-05	0.0004215

**ENSG00000171475**	WIPF2	0.351574711	5.868741337	18.47532014	6.73E-05	0.0004229
**ENSG00000166750**	SLFN5	0.255941581	9.783847177	18.47354691	6.74E-05	0.0004230
**ENSG00000111540**	RAB5B	0.37173186	6.421537346	18.47088708	6.74E-05	0.0004232
**ENSG00000159069**	FBXW5	0.534763379	5.27216973	18.45710297	6.78E-05	0.0004252
**ENSG00000279641**		0.543421203	5.03727343	18.4526967	6.79E-05	0.0004257
**ENSG00000150977**	rilpl2	0.619957537	4.549495446	18.43995801	6.83E-05	0.0004276
**ENSG00000168824**	NSG1	0.639771602	4.629124162	18.42657087	6.86E-05	0.0004296
**ENSG00000198961**	PJA2	0.281621642	7.712138036	18.41985477	6.88E-05	0.0004305
**ENSG00000119688**	ABCD4	-0.524153619	5.19059297	18.40986777	6.91E-05	0.0004318
**ENSG00000170759**	KIF5B	0.260448341	7.838990264	18.4096331	6.91E-05	0.0004318
**ENSG00000265808**	SEC22B	-0.331343478	6.154499188	18.40580796	6.92E-05	0.0004322
**ENSG00000233893**		0.763736292	3.54231087	18.39773298	6.94E-05	0.0004333
**ENSG00000129515**	SNX6	0.300112926	6.776256015	18.39090312	6.96E-05	0.0004342
**ENSG00000166398**	KIAA0355	-0.37787068	6.267735864	18.33276525	7.12E-05	0.0004438
**ENSG00000147324**	MFHAS1	0.384299414	6.430223766	18.33273628	7.12E-05	0.0004438
**ENSG00000140398**	NEIL1	-0.794683947	4.597746845	18.76498717	7.19E-05	0.0004477
**ENSG00000130779**	CLIP1	0.288468563	7.068555127	18.3055307	7.2E-05	0.0004481
**ENSG00000265118**		0.313567126	8.170014629	18.29785737	7.22E-05	0.0004492
**ENSG00000151065**	DCP1B	0.451410551	5.251905306	18.27843137	7.28E-05	0.0004524
**ENSG00000118058**	KMT2A	0.224323338	9.454377984	18.2562646	7.34E-05	0.0004562
**ENSG00000077549**	CAPZB	0.267745221	7.426022996	18.2546067	7.35E-05	0.0004562
**ENSG00000130589**	HELZ2	0.575870043	5.204179719	18.3566797	7.37E-05	0.0004571
**ENSG00000068724**	TTC7A	0.439077691	5.349259822	18.23389163	7.41E-05	0.0004594
**ENSG00000179454**	KLHL28	0.282653989	6.761910287	18.20832153	7.48E-05	0.0004639
**ENSG00000278259**	MYO19	-0.473598498	5.614687811	18.20459432	7.5E-05	0.0004643
**ENSG00000211790**		0.587728372	4.10483105	18.19961545	7.51E-05	0.0004649
**ENSG00000122678**	MIR6838	-0.607008768	4.753531487	18.19528194	7.52E-05	0.0004653
**ENSG00000101391**	CDK5RAP1	-0.534798773	5.354005954	18.19450388	7.53E-05	0.0004653
**ENSG00000165525**	NEMF	-0.296603421	7.56887607	18.18700339	7.55E-05	0.0004663
**ENSG00000198561**	CTNND1	-0.744399601	3.84080581	18.18623585	7.55E-05	0.0004663
**ENSG00000153179**	RASSF3	0.286629955	7.734857598	18.17298068	7.59E-05	0.0004685
**ENSG00000197774**	EME2	-0.642139687	5.421326873	18.77162902	7.7E-05	0.0004753
**ENSG00000143753**	DEGS1	0.446592114	5.775885242	18.09204839	7.84E-05	0.0004833
**ENSG00000163138**	PACRGL	-0.812359177	3.695233765	18.07205958	7.9E-05	0.0004869
**ENSG00000198040**	ZNF84	-0.439951798	5.910417182	18.04745282	7.98E-05	0.0004914
**ENSG00000111860**	CEP85L	-0.305996408	7.496741653	18.04426522	7.99E-05	0.0004917
**ENSG00000099326**	MZF1	-0.832299064	4.951007426	19.07710124	8.02E-05	0.0004931

**ENSG00000197971**	MBP	0.282097249	7.392609152	18.03423915	8.02E-05	0.0004931
**ENSG00000080546**	SESN1	-0.381392502	6.20783707	18.01137051	8.1E-05	0.0004974
**ENSG00000135535**	CD164	0.253774248	8.364294484	18.00671668	8.11E-05	0.0004980
**ENSG00000179918**	SEPHS2	0.505661509	5.149222577	18.00512559	8.12E-05	0.0004980
**ENSG00000272540**		0.515147808	5.844587572	18.36974908	8.13E-05	0.0004984
**ENSG00000084234**	APLP2	0.368926021	5.949497031	18.00021155	8.13E-05	0.0004984
**ENSG00000082146**	STRADB	-0.59888471	4.519753695	17.98625531	8.18E-05	0.0005009
**ENSG00000186020**	ZNF529	-0.395059102	5.82356939	17.97092122	8.23E-05	0.0005037
**ENSG00000113971**	NPHP3	-0.594876521	6.500374159	19.26010906	8.24E-05	0.0005041
**ENSG00000262580**		-1.008134063	4.212262976	18.7870149	8.25E-05	0.0005041
**ENSG00000168792**	ABHD15	0.647872536	4.177908544	17.96443221	8.25E-05	0.0005041
**ENSG00000212443**	SNORA53	1.499808268	5.152437887	19.62831904	8.3E-05	0.0005070
**ENSG00000131797**	CLUHP3	-0.51941459	5.602178476	18.08273189	8.31E-05	0.0005070
**ENSG00000279296**		-0.793647209	6.900010552	19.60742414	8.32E-05	0.0005075
**ENSG00000153283**	CD96	0.241529384	8.306179117	17.93295777	8.35E-05	0.0005093
**ENSG00000111897**	SERINC1	0.289774682	8.179289485	17.91690713	8.41E-05	0.0005119
**ENSG00000156931**	VPS8	-0.375859574	6.611367583	17.91621863	8.41E-05	0.0005119
**ENSG00000136147**	PHF11	-0.412694291	6.528970684	18.07186213	8.41E-05	0.0005119
**ENSG00000116473**	RAP1A	0.280418795	7.527243866	17.90583063	8.44E-05	0.0005137
**ENSG00000113649**	TCERG1	-0.329483914	7.144615492	17.88291346	8.52E-05	0.0005181
**ENSG00000100823**	APEX1	-0.335830797	6.131417358	17.87322701	8.56E-05	0.0005198
**ENSG00000166971**	AKTIP	-0.284996155	7.302347544	17.85534542	8.62E-05	0.0005232
**ENSG00000004700**	RECQL	0.274968449	7.060767969	17.83279822	8.7E-05	0.0005277
**ENSG00000100099**	HPS4	-0.464424076	5.615156643	17.82790755	8.71E-05	0.0005284
**ENSG00000057935**	mta3	-0.552274189	4.833741273	17.82612792	8.72E-05	0.0005285
**ENSG00000007168**	PAFAH1B1	0.221615223	7.719542721	17.82383269	8.73E-05	0.0005286
**ENSG00000085449**	WDFY1	0.361026389	5.793452416	17.8222963	8.73E-05	0.0005287
**ENSG00000245958**	LOC645513	-0.570186156	5.27402423	17.95247621	8.74E-05	0.0005291
**ENSG00000132530**	XAF1	-0.786183117	7.332237724	19.44642284	8.82E-05	0.0005331
**ENSG00000173762**	CD7	-0.551510901	5.385507483	17.89978135	8.85E-05	0.0005346
**ENSG00000224660**	SH3BP5-AS1	-0.319616796	7.016070965	17.78860976	8.85E-05	0.0005346
**ENSG00000186470**	BTN3A2	-0.287398927	7.543827809	17.77042492	8.92E-05	0.0005382
**ENSG00000138834**	MAPK8IP3	-0.698645701	6.090373864	19.18209827	8.96E-05	0.0005408
**ENSG00000065029**	ZNF76	-0.70877805	5.203964946	18.52962827	8.98E-05	0.0005415
**ENSG00000185624**	P4HB	0.348197684	6.424460384	17.74758327	9E-05	0.0005422
**ENSG00000146414**	SHPRH	-0.352957338	7.132334861	17.76855474	9.05E-05	0.0005454
**ENSG00000188785**	ZNF548	-0.457790287	5.487132071	17.72746043	9.07E-05	0.0005460

**ENSG00000104219**	ZDHHC2	0.305950771	6.835012317	17.72550978	9.08E-05	0.0005461
**ENSG00000006459**	KDM7A	0.327835871	7.174112705	17.72423552	9.08E-05	0.0005461
**ENSG00000185291**	IL3RA	-0.772609359	4.151742702	17.71983393	9.1E-05	0.0005467
**ENSG00000117500**	TMED5	0.289379734	7.346852782	17.69621641	9.18E-05	0.0005516
**ENSG00000198876**	DCAF12	0.46430649	4.99787831	17.68481397	9.23E-05	0.0005535
**ENSG00000162526**	TSSK3	-0.840093078	3.791410478	17.68473444	9.23E-05	0.0005535
**ENSG00000157978**	LDLRAP1	-0.323713015	7.464583297	17.68294532	9.23E-05	0.0005536
**ENSG00000196911**	KPNA5	-0.340153095	6.637866824	17.68075568	9.24E-05	0.0005538
**ENSG00000078269**	SYNJ2	-0.42094315	5.598256088	17.67327524	9.27E-05	0.0005551
**ENSG00000177479**	ARIH2	-0.406375415	6.310547669	17.68068429	9.3E-05	0.0005566
**ENSG00000279753**		0.477721713	5.891575185	17.85125598	9.32E-05	0.0005576
**ENSG00000129128**	SPCS3	0.230523512	8.162680019	17.65709666	9.33E-05	0.0005578
**ENSG00000049768**	FOXP3	0.579069104	5.098826846	17.70687383	9.4E-05	0.0005617
**ENSG00000183726**	TMEM50A	0.431486495	6.126095037	17.70818976	9.45E-05	0.0005641
**ENSG00000204516**	MICB	0.628739167	4.261238655	17.61817677	9.48E-05	0.0005656
**ENSG00000105136**	ZNF419	-0.679699791	4.069431075	17.6107925	9.51E-05	0.0005670
**ENSG00000108100**	CCNY	0.309736112	6.3066653	17.60241643	9.54E-05	0.0005686
**ENSG00000152270**	PDE3B	-0.339712496	8.146261768	17.80995158	9.62E-05	0.0005734
**ENSG00000261609**	MIR4720	0.433987103	5.470833624	17.56405658	9.69E-05	0.0005767
**ENSG00000173120**	KDM2A	-0.229010982	7.494795007	17.56210599	9.69E-05	0.0005769
**ENSG00000267100**	ILF3-AS1	-0.643208584	4.208058156	17.55502874	9.72E-05	0.0005781
**ENSG00000140497**	SCAMP2	0.357952991	6.320632207	17.55367031	9.73E-05	0.0005781
**ENSG00000257151**		-0.751125637	4.301315053	17.61331486	9.73E-05	0.0005781
**ENSG00000144283**	PKP4	0.468846932	5.196057203	17.52757842	9.83E-05	0.0005836
**ENSG00000143013**	LMO4	0.702283268	4.373048355	17.50685332	9.91E-05	0.0005882
**ENSG00000135951**	TSGA10	-0.62268213	4.31913025	17.50428603	9.92E-05	0.0005884
**ENSG00000139192**	TAPBPL	-0.387043819	5.779582843	17.49073173	9.98E-05	0.0005913
**ENSG00000071073**	MGAT4A	-0.220339038	8.774430905	17.48883282	9.98E-05	0.0005914
**ENSG00000197536**	C5orf56	-0.359872176	7.017159927	17.53184769	0.0001014	0.0006009
**ENSG00000163902**	RPN1	0.371303174	6.359193039	17.4203009	0.0001026	0.0006073
**ENSG00000058262**	SEC61A1	0.323889152	6.935861936	17.41898529	0.0001026	0.0006073
**ENSG00000013573**	DDX11	-0.586680914	4.708589002	17.4154876	0.0001028	0.0006078
**ENSG00000152601**	MBNL1	-0.223357778	10.20313279	17.40435129	0.0001032	0.0006102
**ENSG00000169446**	MMGT1	0.401139476	6.085149711	17.40170991	0.0001034	0.0006105
**ENSG00000126709**	IFI6	0.644631861	4.289069831	17.39718028	0.0001035	0.0006113
**ENSG00000091009**	RBM27	0.300173901	7.201238069	17.38864676	0.0001039	0.0006130
**ENSG00000163703**	creld1	-0.609134968	4.104687957	17.36409668	0.0001049	0.0006188

**ENSG00000167996**	FTH1	0.365351375	8.384925082	17.85895423	0.0001055	0.0006217
**ENSG00000167995**	BEST1	0.367066648	8.244210048	17.85742331	0.0001059	0.0006236
**ENSG00000132694**	ARHGEF11	-0.514603971	5.095348908	17.32120483	0.0001068	0.0006285
**ENSG00000124575**	Hist1h1d	0.646408331	7.850163319	18.84537774	0.0001076	0.0006329
**ENSG00000210196**		-0.525322458	6.798479386	18.39252172	0.0001076	0.0006329
**ENSG00000026297**	RNASET2	-0.289192142	7.296621022	17.2951326	0.0001079	0.0006341
**ENSG00000105821**	DNAJC2	-0.380502283	6.218987953	17.28050943	0.0001085	0.0006375
**ENSG00000114302**	PRKAR2A	0.393534411	6.023177322	17.27602637	0.0001087	0.0006383
**ENSG00000145020**	AMT	-0.73437203	4.060386106	17.27325417	0.0001089	0.0006385
**ENSG00000198586**	TLK1	0.263527349	7.92078654	17.27258538	0.0001089	0.0006385
**ENSG00000197747**	S100A10	0.565675443	6.363557912	18.31259361	0.0001093	0.0006406
**ENSG00000075826**	SEC31B	-0.950958084	5.690586136	18.77992005	0.0001096	0.0006420
**ENSG00000188895**	MSL1	-0.344345739	6.676188494	17.2472859	0.0001100	0.0006440
**ENSG00000115649**	CNPPD1	0.46184567	4.971159825	17.22550338	0.0001110	0.0006493
**ENSG00000169991**	IFFO2	0.40034198	6.009094716	17.21514599	0.0001114	0.0006517
**ENSG00000205903**	ZNF316	-0.70261457	4.440286065	17.21262164	0.0001116	0.0006520
**ENSG00000104957**	CCDC130	-0.589090312	4.814060374	17.2030012	0.0001120	0.0006541
**ENSG00000205560**	CPT1B	-0.876052985	4.707988303	18.11310241	0.0001122	0.0006550
**ENSG00000148700**	ADD3	-0.212508266	9.083341277	17.19360281	0.0001124	0.0006559
**ENSG00000140526**	ABHD2	0.306865614	6.723637465	17.17680681	0.0001132	0.0006600
**ENSG00000113273**	ARSB	0.594126995	3.958317412	17.16528825	0.0001137	0.0006627
**ENSG00000101751**	POLI	-0.570067739	5.683991967	17.70385925	0.0001138	0.0006630
**ENSG00000211801**		0.613469232	4.088994754	17.15905696	0.0001140	0.0006637
**ENSG00000084073**	ZMPSTE24	0.475840334	5.096071006	17.14830644	0.0001145	0.0006662
**ENSG00000040933**	INPP4A	-0.302832874	7.880206139	17.14149531	0.0001148	0.0006677
**ENSG00000277586**	NEFL	-0.780655516	4.183294337	17.12941174	0.0001154	0.0006706
**ENSG00000205707**	ETFRF1	-0.473665834	5.128349367	17.11271943	0.0001162	0.0006747
**ENSG00000156500**	FAM122C	-0.68238768	3.827053586	17.10653144	0.0001165	0.0006760
**ENSG00000210174**		-0.53903851	5.149612182	17.09686192	0.0001169	0.0006783
**ENSG00000270362**	HMGN3-AS1	-0.683944238	3.616098375	17.08822078	0.0001173	0.0006803
**ENSG00000168092**	PAFAH1B2	0.35321839	6.236212715	17.08544666	0.0001175	0.0006807
**ENSG00000103227**	LMF1	-0.77387431	4.340277125	17.26413632	0.0001175	0.0006808
**ENSG00000100288**	CHKB	-0.79658656	5.38779188	18.28970194	0.0001178	0.000682
**ENSG00000108515**	ENO3	0.477363155	6.248457615	17.5972964	0.0001181	0.0006835
**ENSG00000260853**		-0.765583506	4.537214617	17.38878586	0.0001182	0.0006837
**ENSG00000103168**	TAF1C	-0.845796881	4.749478454	17.92789921	0.0001189	0.0006869
**ENSG00000102034**	ELF4	0.319106873	6.088460865	17.04588729	0.0001194	0.0006894

**ENSG00000272578**		-0.801435585	5.553987474	18.35728992	0.0001194	0.0006894
**ENSG00000124214**	STAU1	0.320030892	6.442256659	17.03017855	0.0001201	0.0006931
**ENSG00000148110**	MFSD14B	0.357477407	5.767891596	17.02214742	0.0001205	0.0006950
**ENSG00000279838**		-0.7121447	4.035107974	17.00919734	0.0001211	0.0006982
**ENSG00000171700**	RGS19	0.560725334	4.956759274	17.00506969	0.0001214	0.0006990
**ENSG00000119943**	MIR1287	-0.85754907	3.63238963	16.99786614	0.0001217	0.0007006
**ENSG00000242294**	STAG3L5P	-0.798377627	5.566418162	18.30693566	0.0001218	0.0007006
**ENSG00000169249**	zrsr2	-0.477038352	5.477981374	16.99249008	0.0001220	0.0007014
**ENSG00000133226**	SRRM1	-0.227286977	8.055164378	16.98647668	0.0001223	0.0007028
**ENSG00000111450**	STX2	-0.475722863	5.493033371	16.96758611	0.0001232	0.0007078
**ENSG00000269609**	RPARP-AS1	-0.608137758	4.42532427	16.94960773	0.0001241	0.0007126
**ENSG00000139211**	AMIGO2	-0.546054094	4.477943591	16.9365421	0.0001248	0.0007160
**ENSG00000166454**	ATMIN	0.338324053	6.595821668	16.92928668	0.0001251	0.0007177
**ENSG00000037757**	mri1	-0.638900533	4.970937924	17.13231301	0.000127	0.0007276
**ENSG00000211788**		0.532129855	4.222317896	16.8874025	0.0001273	0.0007292
**ENSG00000166130**	IKBIP	0.750663139	3.616059897	16.88349571	0.0001275	0.0007300
**ENSG00000112640**	PPP2R5D	0.44224845	5.244229446	16.87906555	0.0001277	0.0007309
**ENSG00000172845**	SP3	0.232044914	7.882554853	16.87063161	0.0001282	0.0007330
**ENSG00000103423**	DNAJA3	-0.536023627	5.103014582	16.86654422	0.0001284	0.0007338
**ENSG00000123737**	EXOSC9	-0.376957404	5.886418157	16.8569369	0.0001289	0.0007363
**ENSG00000163617**	CCDC191	-0.753815669	3.906520613	16.83751852	0.0001299	0.0007417
**ENSG00000154001**	PPP2R5E	0.289063671	6.725970816	16.83629368	0.0001300	0.0007417
**ENSG00000119514**	GALNT12	-0.678430822	3.532552076	16.82127086	0.0001308	0.0007459
**ENSG00000077458**	FAM76B	-0.370622787	6.014078317	16.81778043	0.0001310	0.0007465
**ENSG00000254166**		-0.818227678	4.174441211	17.00180364	0.0001312	0.0007472
**ENSG00000079999**	KEAP1	0.724406877	3.677863395	16.80274459	0.0001318	0.0007502
**ENSG00000089012**	SIRPG	-0.414516013	5.480866587	16.80179855	0.0001318	0.0007502
**ENSG00000151136**	BTBD11	0.520176193	4.940665662	16.79965042	0.0001319	0.0007504
**ENSG00000134444**	KIAA1468	-0.346839326	6.959683279	16.79174947	0.0001324	0.0007524
**ENSG00000204304**	PBX2	-0.426102438	6.199050464	16.92186085	0.0001328	0.0007546
**ENSG00000073417**	PDE8A	-0.470693485	5.384995006	16.76890652	0.0001336	0.0007586
**ENSG00000159023**	EPB41	0.293574277	8.979420437	16.76693109	0.0001337	0.0007588
**ENSG00000066294**	CD84	0.311235535	7.525218337	16.75719104	0.0001342	0.0007614
**ENSG00000176700**		-0.497988743	6.394350478	17.44811627	0.0001354	0.0007677
**ENSG00000059378**	PARP12	-0.446753376	6.144889662	16.95043709	0.0001355	0.0007677
**ENSG00000100906**	NFKBIA	-0.392148351	6.185084818	16.72677678	0.0001359	0.0007697
**ENSG00000104960**	PTOV1	-0.578560698	5.360263429	17.05801358	0.0001362	0.0007709

**ENSG00000156313**	RPGR	-0.692862111	4.389558751	16.70931698	0.0001369	0.0007743
**ENSG00000213614**	HEXA	-0.416245041	5.58825739	16.70459456	0.0001371	0.0007754
**ENSG00000121774**	KHDRBS1	0.265955477	7.041232203	16.69816026	0.0001375	0.0007770
**ENSG00000155287**	SLC25A28	-0.591227384	4.632611451	16.69473913	0.0001377	0.0007777
**ENSG00000186481**		-0.771612528	4.257058476	16.84194045	0.0001379	0.0007784
**ENSG00000173726**	TOMM20	0.299160532	6.649032345	16.6804042	0.0001385	0.0007814
**ENSG00000144152**	fbln7	0.685295458	3.640283751	16.67552873	0.0001388	0.0007819
**ENSG00000213463**	SYNJ2BP	-0.446498573	5.925310369	16.78575737	0.0001388	0.0007819
**ENSG00000115282**	TTC31	-0.630683079	4.79667702	16.78487369	0.0001389	0.0007819
**ENSG00000278962**		0.726337488	3.773191364	16.6735315	0.0001389	0.0007819
**ENSG00000233937**		-0.418794618	5.603450873	16.66490552	0.0001394	0.0007841
**ENSG00000110367**	DDX6	0.238534387	9.183961752	16.66414538	0.0001394	0.0007841
**ENSG00000140400**	MAN2C1	-0.694111676	5.73482404	17.77111094	0.0001406	0.0007904
**ENSG00000167461**	RAB8A	0.344465761	5.819161152	16.63479627	0.0001411	0.0007925
**ENSG00000155363**	MOV10	-0.383148976	5.621665082	16.63401731	0.0001411	0.0007925
**ENSG00000104177**	MYEF2	-0.60028301	4.765691849	16.62745773	0.0001415	0.0007942
**ENSG00000167377**	ZNF23	-0.658576293	4.140383654	16.59324556	0.0001435	0.0008049
**ENSG00000196605**	ZNF846	-0.762200053	3.816189706	16.59038818	0.0001437	0.0008055
**ENSG00000188229**	TUBB4B	0.545126429	4.393842311	16.57996809	0.0001443	0.0008085
**ENSG00000273637**		0.481167382	4.493794834	16.57371839	0.0001447	0.0008101
**ENSG00000153933**	DGKE	-0.353110942	6.338670248	16.56985694	0.0001449	0.0008108
**ENSG00000198162**	MAN1A2	0.315203323	7.571489062	16.5689692	0.0001450	0.0008108
**ENSG00000102265**	TIMP1	0.598988693	4.657615122	16.56048377	0.0001455	0.0008132
**ENSG00000154358**	OBSCN	-0.390019963	7.229865959	17.01801374	0.0001456	0.0008133
**ENSG00000164048**	ZNF589	-0.583738877	5.046314142	16.66521512	0.0001460	0.0008151
**ENSG00000223959**	AFG3L1P	-0.628110307	4.669364192	16.53334594	0.0001471	0.0008209
**ENSG00000124789**	NUP153	0.245072359	7.430110353	16.52186547	0.0001478	0.0008244
**ENSG00000273559**	CWC25	-0.462711326	5.231824375	16.50416925	0.0001488	0.0008299
**ENSG00000160226**	c21orf2	-0.74433695	4.338444531	16.60507587	0.0001503	0.0008375
**ENSG00000178971**	CTC1	-0.408395843	7.533723176	17.18012901	0.0001513	0.0008425
**ENSG00000132274**	TRIM22	-0.289275111	8.119816514	16.45492534	0.0001519	0.0008455
**ENSG00000110330**	BIRC2	-0.293903668	6.706499589	16.45282139	0.0001520	0.0008457
**ENSG00000168802**	CHTF8	0.417561792	5.323087787	16.44428969	0.0001525	0.0008482
**ENSG00000116106**	EPHA4	0.440408742	6.059492496	16.56275792	0.0001529	0.0008491
**ENSG00000166822**	TMEM170A	0.348403824	6.245651581	16.43926108	0.0001529	0.0008491
**ENSG00000235016**	SEMA3F-AS1	-0.526454942	5.105559405	16.41590382	0.0001543	0.0008568
**ENSG00000203709**	Mir29b2	-0.514239327	6.264950366	17.14697912	0.0001545	0.0008573

**ENSG00000118507**	AKAP7	-0.526208452	4.794590834	16.41072035	0.0001547	0.0008577
**ENSG00000164180**	TMEM161B	-0.41492198	6.15481705	16.47352772	0.0001550	0.0008591
**ENSG00000221420**	Snora81	0.938073684	4.977844816	17.63501761	0.0001558	0.0008628
**ENSG00000137145**	DENND4C	0.232441634	7.407475943	16.39228336	0.0001558	0.0008628
**ENSG00000176248**	ANAPC2	-0.603641704	4.846817887	16.43855483	0.0001568	0.0008676
**ENSG00000086758**	HUWE1	0.227154099	9.017991614	16.3589219	0.0001580	0.0008738
**ENSG00000096063**	SRPK1	0.323792407	6.201217442	16.34400424	0.0001589	0.0008787
**ENSG00000163714**	U2SURP	-0.240160258	8.056059616	16.34069774	0.0001592	0.0008794
**ENSG00000211799**		0.680087958	3.535459349	16.33327393	0.0001596	0.0008817
**ENSG00000178252**	wdr6	-0.380969464	6.388082481	16.3132464	0.0001610	0.0008885
**ENSG00000163938**	GNL3	-0.336826117	6.205458698	16.30880143	0.0001613	0.0008896
**ENSG00000103769**	RAB11A	0.299876825	6.51616209	16.30419819	0.0001616	0.0008908
**ENSG00000172936**	MYD88	0.394618458	6.032339562	16.29843238	0.0001619	0.0008925
**ENSG00000105612**	dnase2	0.61058437	3.819622611	16.27632837	0.0001634	0.0009001
**ENSG00000179912**	R3HDM2	-0.282835267	6.985216843	16.27518543	0.0001635	0.0009001
**ENSG00000102189**	EEA1	-0.3619468	6.909325001	16.38150031	0.0001637	0.0009008
**ENSG00000163945**	UVSSA	-0.563082082	6.101795488	17.13070834	0.0001639	0.0009012
**ENSG00000106028**	SSBP1	-0.328570771	5.815981548	16.26590762	0.0001641	0.0009021
**ENSG00000114127**	XRN1	0.232885964	8.370784702	16.26049908	0.0001645	0.0009036
**ENSG00000135597**	REPS1	-0.349249646	6.655977403	16.25379112	0.0001649	0.0009056
**ENSG00000258727**	LOC102724814	-0.658392846	5.864831257	17.32442173	0.0001658	0.0009090
**ENSG00000135678**	CPM	0.606982274	3.930479228	16.24119443	0.0001658	0.0009090
**ENSG00000171603**	CLSTN1	0.248445303	7.302137097	16.2405937	0.0001658	0.0009090
**ENSG00000179335**	CLK3	-0.345842846	6.550967448	16.23965948	0.0001659	0.0009090
**ENSG00000138964**	PARVG	-0.511480725	6.225980232	16.93196461	0.0001662	0.0009102
**ENSG00000163608**	NEPRO	-0.312065133	6.611457757	16.23362914	0.0001663	0.0009103
**ENSG00000110911**	SLC11A2	-0.520178362	5.570976819	16.44895801	0.0001668	0.0009126
**ENSG00000177707**	NECTIN3	0.71732725	3.650862896	16.21946802	0.0001673	0.0009147
**ENSG00000175309**	PHYKPL	-0.441233117	6.701073914	16.75389448	0.0001676	0.0009159
**ENSG00000175105**	ZNF654	0.384014012	6.182367589	16.20341515	0.0001684	0.0009198
**ENSG00000187837**	HIST1H1C	0.740950308	7.378336793	17.5789687	0.0001685	0.0009201
**ENSG00000214021**	TTLL3	-0.770141895	5.68820485	17.43313195	0.0001687	0.0009206
**ENSG00000092529**	CAPN3	-0.753994058	4.090507951	16.20458128	0.0001692	0.0009228
**ENSG00000140299**	BNIP2	0.276203472	7.395954318	16.18803837	0.0001695	0.0009237
**ENSG00000174306**	ZHX3	-0.554598569	5.273490931	16.34600133	0.0001698	0.0009250
**ENSG00000111596**	CNOT2	-0.279154147	7.057521358	16.18206009	0.0001699	0.0009250
**ENSG00000162819**	BROX	0.293985531	6.81770688	16.17794059	0.0001702	0.0009261

**ENSG00000134333**	LDHA	0.32434416	7.10320659	16.17122834	0.0001706	0.0009281
**ENSG00000146476**	ARMT1	0.480492422	4.695264803	16.16676053	0.0001710	0.0009294
**ENSG00000196313**	pom121	-0.298596936	6.791677709	16.14396839	0.0001726	0.0009376
**ENSG00000104133**	SPG11	-0.281472882	7.65199605	16.12904256	0.0001736	0.0009429
**ENSG00000182378**	PLCXD1	-0.598459283	4.658047334	16.12380389	0.0001740	0.0009444
**ENSG00000135655**	MIR6125	-0.310403654	8.411532453	16.22940802	0.0001741	0.0009444
**ENSG00000100003**	SEC14L2	-0.752581509	3.994601606	16.11871479	0.0001744	0.0009455
**ENSG00000163785**	RYK	-0.363281908	5.598191507	16.11092878	0.0001749	0.0009480
**ENSG00000118705**	RPN2	0.285587825	6.876730611	16.06305934	0.0001784	0.0009664
**ENSG00000110711**	AIP	0.421341088	5.052352248	16.05836369	0.0001788	0.0009678
**ENSG00000132383**	RPA1	0.33862652	6.287804842	16.05482632	0.0001790	0.0009687
**ENSG00000100519**	PSMC6	-0.329701605	6.239283183	16.04573819	0.0001797	0.0009715
**ENSG00000139160**	ETFBKMT	-0.651511583	3.908840741	16.04518761	0.0001797	0.0009715
**ENSG00000283958**		1.066879943	4.872021173	17.32644275	0.0001802	0.0009729
**ENSG00000173200**	PARP15	-0.605210684	6.956956252	17.30631393	0.0001802	0.0009729
**ENSG00000126870**	WDR60	-0.42950865	5.215422645	16.03483581	0.0001805	0.0009742
**ENSG00000147155**	EBP	0.727961962	3.692695825	16.03198422	0.0001807	0.0009748
**ENSG00000185163**	DDX51	-0.527303028	4.593620396	16.02765588	0.0001810	0.0009761
**ENSG00000166484**	MAPK7	0.505816797	4.311266438	16.01950416	0.0001817	0.0009786
**ENSG00000155090**	KLF10	0.63956839	4.764042413	16.13595695	0.0001818	0.0009786
**ENSG00000204130**	RUFY2	-0.278215485	6.325468768	16.0169528	0.0001818	0.0009786
**ENSG00000100426**	zbed4	0.336815522	5.801007031	16.01649537	0.0001819	0.0009786
**ENSG00000007129**	CEACAM21	-0.413305447	5.298322433	16.01129998	0.0001823	0.0009801
**ENSG00000154451**	GBP5	0.415832593	8.523433635	16.87781507	0.0001829	0.0009830
**ENSG00000121454**	LHX4	-0.685768061	4.535974597	16.13609528	0.0001841	0.0009883
**ENSG00000169762**	TAPT1	-0.346333232	5.731326401	15.98739896	0.0001841	0.0009883
**ENSG00000186298**	PPP1CC	0.250871946	7.341771548	15.98037251	0.0001846	0.0009907
**ENSG00000271581**		0.503263827	8.200056326	17.18270444	0.0001860	0.0009977
**ENSG00000134899**	ERCC5	-0.233091741	7.57662658	15.9604395	0.0001861	0.0009979
**ENSG00000111229**	ARPC3	0.290197931	7.067014467	15.95283109	0.0001867	0.0010005
**ENSG00000144935**	TRPC1	-0.695313057	3.545540303	15.94960907	0.0001870	0.0010013
**ENSG00000247287**		-0.667933249	4.666821603	16.10834273	0.0001884	0.0010084
**ENSG00000119537**	KDSR	0.472224711	5.970019421	16.20816873	0.0001900	0.0010165
**ENSG00000137038**	TMEM261	-0.48727617	4.381728534	15.89977568	0.0001909	0.0010206
**ENSG00000157617**	C2CD2	-0.71075961	3.830992488	15.89713501	0.0001911	0.0010212
**ENSG00000261490**		-0.493672588	5.418988676	15.87511913	0.0001928	0.0010301
**ENSG00000230262**	MIRLET7DHG	-0.738229247	3.498818395	15.87281293	0.0001930	0.0010304

**ENSG00000274272**		-0.665137616	5.888517002	16.9139404	0.0001960	0.0010457
**ENSG00000104218**	CSPP1	-0.409590353	5.735302612	15.82377949	0.0001970	0.0010505
**ENSG00000164300**	SERINC5	-0.260895267	8.181217919	15.81336547	0.0001978	0.0010545
**ENSG00000115947**	ORC4	-0.308457505	6.29436328	15.79255047	0.0001995	0.0010631
**ENSG00000008128**	CDK11A	-0.432131737	5.846450401	15.79883817	0.0002003	0.0010667
**ENSG00000135837**	cep350	0.259301773	8.408544379	15.77999821	0.0002006	0.0010675
**ENSG00000100030**	MAPK1	0.255597802	7.491521371	15.75008298	0.0002031	0.0010800
**ENSG00000147274**	RBMX	-0.245111862	8.106355118	15.74954263	0.0002031	0.0010800
**ENSG00000115306**	SPTBN1	-0.258361004	8.914553741	15.7481967	0.0002032	0.0010800
**ENSG00000174231**	PRPF8	0.218506418	9.161098932	15.7397055	0.0002039	0.0010828
**ENSG00000136709**	WDR33	-0.232199502	7.48193996	15.73957021	0.0002039	0.0010828
**ENSG00000163754**	GYG1	0.522504763	4.734554071	15.73725135	0.0002041	0.0010833
**ENSG00000196497**	IPO4	-0.553256028	4.500104668	15.73165759	0.0002046	0.0010852
**ENSG00000144535**	DIS3L2	-0.421578491	5.610719012	15.72974438	0.0002048	0.0010855
**ENSG00000185650**	ZFP36L1	0.261271768	8.286561448	15.72816121	0.0002049	0.0010857
**ENSG00000136273**	HUS1	-0.31649077	5.853809597	15.72285736	0.0002054	0.0010875
**ENSG00000129292**	PHF20L1	-0.339308639	7.174029333	15.82371248	0.0002055	0.0010878
**ENSG00000244462**	rbm12	0.250563766	7.299336345	15.70168836	0.0002072	0.0010960
**ENSG00000078747**	ITCH	0.274290666	7.034540327	15.69855629	0.0002074	0.0010969
**ENSG00000218283**		0.568959294	4.516085546	15.69289867	0.0002079	0.0010985
**ENSG00000127585**	FBXL16	0.499264585	4.652946257	15.69267527	0.0002079	0.0010985
**ENSG00000269743**	SLC25A53	0.583546378	3.996240582	15.68386863	0.0002087	0.0011019
**ENSG00000260565**		-0.411051319	6.171412117	15.77533802	0.0002099	0.0011074
**ENSG00000105486**	LIG1	-0.419737226	5.595381005	15.65805204	0.0002110	0.0011127
**ENSG00000204308**	RNF5	0.753856149	3.427742347	15.65388873	0.0002113	0.0011140
**ENSG00000115204**	MPV17	-0.546666251	4.643309405	15.64556308	0.0002121	0.0011173
**ENSG00000181744**	c3orf58	0.461904109	5.421108439	15.61677674	0.0002146	0.0011302
**ENSG00000124596**	OARD1	-0.359083875	5.508267044	15.61168787	0.0002151	0.0011320
**ENSG00000122477**	LRRC39	-0.73844561	4.055213532	15.60775065	0.0002154	0.0011332
**ENSG00000143756**	FBXO28	0.356461623	5.966875936	15.60678206	0.0002155	0.0011332
**ENSG00000204267**	tap2	-0.34356773	6.613121922	15.59483475	0.0002166	0.0011382
**ENSG00000051620**	HEBP2	-0.375545465	6.033154224	15.59296777	0.0002167	0.0011386
**ENSG00000131876**	SNRPA1	-0.583992689	5.793447494	16.34008972	0.0002177	0.0011428
**ENSG00000146828**	SLC12A9	-0.627280878	5.000672689	15.87519492	0.0002192	0.0011502
**ENSG00000139679**	lpar6	-0.413197452	6.894855045	16.05013891	0.0002194	0.0011507
**ENSG00000256664**		0.529286132	4.201507185	15.5413428	0.0002214	0.0011609
**ENSG00000164543**	STK17A	0.281487877	7.360341991	15.53972764	0.0002216	0.0011611

**ENSG00000058600**	POLR3E	-0.365431321	6.07864618	15.53830011	0.0002217	0.0011612
**ENSG00000205045**	SLFN12L	0.369770667	7.482965268	15.97020282	0.0002231	0.0011679
**ENSG00000213918**	DNASE1	-0.504240345	5.977025059	16.02403388	0.0002232	0.0011679
**ENSG00000180233**	ZNRF2	0.400514131	5.476837735	15.51558583	0.0002238	0.0011705
**ENSG00000129347**	KRI1	-0.338553239	5.852602398	15.51137207	0.0002242	0.0011719
**ENSG00000141258**	SGSM2	-0.81305413	5.932700731	16.74025615	0.0002247	0.0011741
**ENSG00000283228**		0.452451663	5.107757979	15.4904172	0.0002262	0.0011808
**ENSG00000160679**	CHTOP	-0.302035449	6.755504275	15.48958814	0.0002263	0.0011808
**ENSG00000175322**	znf519	-0.602259206	4.420435326	15.48632767	0.0002266	0.0011814
**ENSG00000131069**	ACSS2	-0.764186726	3.495930455	15.4859888	0.0002266	0.0011814
**ENSG00000163577**	EIF5A2	0.534210073	4.287986822	15.4728968	0.0002278	0.0011873
**ENSG00000151718**	WWC2	-0.724263486	3.745055736	15.46701372	0.0002284	0.0011896
**ENSG00000009954**	BAZ1B	0.257329844	7.653246292	15.44785203	0.0002302	0.0011986
**ENSG00000001631**	KRIT1	-0.350478286	6.865069223	15.47077177	0.0002334	0.0012145
**ENSG00000078687**	TNRC6C	-0.238947075	7.784996469	15.40531387	0.0002343	0.0012187
**ENSG00000147475**	ERLIN2	-0.433324486	4.685306226	15.4043521	0.0002344	0.0012187
**ENSG00000185917**	SETD4	-0.533079614	4.697231918	15.39489993	0.0002354	0.0012229
**ENSG00000231721**	LINC-PINT	-0.451982297	6.716805092	15.99566701	0.0002371	0.0012306
**ENSG00000179456**	zbtb18	-0.433124752	6.172993179	15.62917876	0.0002371	0.0012306
**ENSG00000124201**	ZNFX1	0.236008958	7.640964104	15.37517006	0.0002373	0.0012311
**ENSG00000163412**	EIF4E3	0.264084737	7.487311861	15.36622739	0.0002382	0.0012348
**ENSG00000166387**	PPFIBP2	-0.553936099	4.320058811	15.36567739	0.0002382	0.0012348
**ENSG00000185379**	RAD51D	-0.520862676	4.82087353	15.34856219	0.0002400	0.0012430
**ENSG00000144744**	UBA3	-0.318113934	6.281921994	15.34628626	0.0002402	0.0012436
**ENSG00000166377**	ATP9B	-0.325080452	6.522190898	15.34429359	0.0002404	0.0012440
**ENSG00000163867**	ZMYM6	-0.428635143	6.32598391	15.62734751	0.0002421	0.0012523
**ENSG00000119723**	COQ6	-0.522774807	4.81352461	15.32577596	0.0002422	0.0012524
**ENSG00000148690**	FRA10AC1	-0.429966498	5.856537652	15.36339518	0.0002435	0.0012584
**ENSG00000276965**		-0.454631087	5.09121579	15.31090514	0.0002438	0.0012590
**ENSG00000184445**	KNTC1	-0.395916622	5.878870772	15.30114077	0.0002448	0.0012635
**ENSG00000272498**		-0.626596513	4.497975495	15.29934132	0.0002449	0.0012638
**ENSG00000232656**	IDI2-AS1	0.732669761	3.560290115	15.29732925	0.0002451	0.0012642
**ENSG00000136161**	RCBTB2	-0.572056443	4.587703144	15.2932559	0.0002456	0.0012652
**ENSG00000167491**	GATAD2A	0.318013506	6.742901495	15.29198755	0.0002457	0.0012652
**ENSG00000115170**	ACVR1	0.437342218	5.399504119	15.29189301	0.0002457	0.0012652
**ENSG00000134255**	CEPT1	-0.347287719	7.627143289	15.6397696	0.0002463	0.0012673
**ENSG00000169895**	SYAP1	0.415163147	5.62345703	15.28563998	0.0002463	0.0012673

**ENSG00000269929**		-0.702065395	3.649304865	15.27065389	0.0002479	0.0012746
**ENSG00000169660**	HEXDC	-0.71984985	4.804689705	15.75673064	0.0002496	0.0012823
**ENSG00000258989**		0.406585479	5.471738945	15.25361419	0.0002497	0.0012823
**ENSG00000009724**	MASP2	-0.714427131	4.507162778	15.44947008	0.0002498	0.0012823
**ENSG00000261716**		-0.574892175	4.448658637	15.24704525	0.0002503	0.0012847
**ENSG00000062650**	WAPL	0.24570884	7.714140592	15.23604556	0.0002515	0.0012900
**ENSG00000132323**	ILKAP	-0.432276036	5.298365176	15.23483927	0.0002516	0.0012900
**ENSG00000168672**	FAM84B	0.36758157	5.495054115	15.22970416	0.0002522	0.0012922
**ENSG00000231160**	KLF3-AS1	-0.653556085	4.22586348	15.22395741	0.0002528	0.0012947
**ENSG00000146802**	TMEM168	-0.36842718	6.012973242	15.22129145	0.0002531	0.0012955
**ENSG00000105656**	ELL	0.49605223	4.457031025	15.20589842	0.0002547	0.0013026
**ENSG00000168350**	DEGS2	-0.383698826	6.194852377	15.20588404	0.0002547	0.0013026
**ENSG00000188295**	ZNF669	0.579917497	3.845941029	15.20255468	0.0002550	0.0013037
**ENSG00000145623**	OSMR	-0.585947747	3.936694868	15.19747007	0.0002556	0.0013059
**ENSG00000204070**	SYS1	0.361511031	5.570643502	15.19534535	0.0002558	0.0013064
**ENSG00000197283**	MIR5004	-0.376047374	6.345060288	15.20563524	0.0002565	0.0013091
**ENSG00000178467**	P4HTM	-0.591571232	4.694675849	15.1753037	0.0002580	0.0013161
**ENSG00000272980**		0.351192274	6.082225467	15.16671683	0.0002589	0.0013199
**ENSG00000197746**	PSAP	0.29286799	7.045299072	15.16609971	0.0002590	0.0013199
**ENSG00000265784**		0.494763746	4.831124864	15.15903885	0.0002597	0.0013232
**ENSG00000115207**	GTF3C2	-0.345173219	5.73609589	15.15580794	0.0002601	0.0013243
**ENSG00000214941**	ZSWIM7	-0.733540358	4.006516298	15.14283799	0.0002615	0.0013309
**ENSG00000062716**	MIR21	0.44050119	7.009259691	15.84405925	0.0002629	0.0013372
**ENSG00000174197**	MGA	0.212986926	7.820762347	15.12005044	0.0002640	0.0013423
**ENSG00000104859**	CLASRP	-0.569151933	5.113469587	15.26940004	0.0002642	0.0013425
**ENSG00000111843**	TMEM14C	0.647408464	3.97556441	15.11642448	0.0002644	0.0013431
**ENSG00000196715**	VKORC1L1	0.44538612	4.993331535	15.11324785	0.0002648	0.0013442
**ENSG00000101342**	TLDC2	-0.302109364	6.275746854	15.08930223	0.0002674	0.0013571
**ENSG00000263934**	SNORD3A	1.4851776	6.924116162	16.28444738	0.0002687	0.0013627
**ENSG00000169375**	SIN3A	0.217656392	7.616200719	15.07018184	0.0002696	0.0013667
**ENSG00000184208**	C22orf46	-0.513438955	4.226735245	15.06641762	0.0002700	0.0013682
**ENSG00000244491**		0.296695702	6.210225783	15.06311254	0.0002704	0.0013694
**ENSG00000213204**	CFAP206	-0.468005067	4.787110927	15.05700597	0.0002711	0.0013722
**ENSG00000178761**	FAM219B	-0.466868401	5.474727073	15.04876178	0.0002720	0.0013763
**ENSG00000158985**	CDC42SE2	0.23689418	9.131367497	15.04474296	0.0002725	0.0013780
**ENSG00000274422**		-1.024250461	4.395946005	15.95747368	0.0002730	0.0013798
**ENSG00000206561**	COLQ	0.811218756	3.473605996	15.03662553	0.0002734	0.0013813

**ENSG00000160072**	atad3b	-0.717650007	4.351920859	15.14144119	0.0002742	0.0013845
**ENSG00000174749**	C4orf32	-0.705704048	4.374867105	15.12192338	0.0002745	0.0013853
**ENSG00000197457**	STMN3	-0.558145709	5.040538787	15.116742	0.0002747	0.0013853
**ENSG00000135956**	TMEM127	0.327163292	5.748317843	15.0249459	0.0002747	0.0013853
**ENSG00000103510**	kat8	-0.379673708	5.480292994	15.02368469	0.0002749	0.0013853
**ENSG00000030419**	IKZF2	0.691529711	6.33826446	16.17491884	0.0002750	0.0013853
**ENSG00000008324**	SS18L2	0.61966855	3.955761217	15.02049221	0.0002753	0.0013860
**ENSG00000273000**		-0.68909764	5.245574443	15.7482133	0.0002754	0.0013861
**ENSG00000129534**	MIS18BP1	0.363521478	6.206589548	15.01813529	0.0002755	0.0013861
**ENSG00000135334**	AKIRIN2	0.380553234	5.248066587	15.01147884	0.0002763	0.0013893
**ENSG00000120742**	SERP1	0.256567447	7.169181222	15.00510308	0.0002770	0.0013923
**ENSG00000257065**		0.596543638	3.867348883	14.98844957	0.0002790	0.0014010
**ENSG00000100359**	SGSM3	-0.614503455	5.045120864	15.31532334	0.0002790	0.0014010
**ENSG00000239697**	TNFSF12	0.589971962	4.148084231	14.97994558	0.0002800	0.0014051
**ENSG00000043143**	JADE2	0.245988779	7.495714468	14.97241472	0.0002809	0.0014089
**ENSG00000189241**	TSPYL1	0.297936781	7.149404046	14.96984536	0.0002812	0.0014097
**ENSG00000081665**	ZNF506	-0.261588599	7.228664644	14.94214101	0.0002845	0.0014255
**ENSG00000278311**	GGNBP2	-0.245440983	7.166740949	14.93191048	0.0002857	0.0014310
**ENSG00000108953**	YWHAE	0.311130164	6.650046991	14.92808115	0.0002861	0.0014326
**ENSG00000061987**	MON2	-0.30420193	7.590120965	14.96294209	0.0002866	0.0014342
**ENSG00000088986**	DYNLL1	0.550766492	5.125040502	15.05126643	0.0002876	0.0014387
**ENSG00000236778**	INTS6-AS1	-0.618749571	3.8607338	14.90779004	0.0002886	0.0014428
**ENSG00000133321**	RARRES3	0.473424042	5.610514909	15.03148535	0.0002896	0.0014472
**ENSG00000076555**	ACACB	-0.441117476	5.084221034	14.89485631	0.0002902	0.0014491
**ENSG00000069849**	ATP1B3	0.445746035	4.862422619	14.89338306	0.0002904	0.0014491
**ENSG00000010803**	SCMH1	0.41214725	5.042439134	14.89290207	0.0002904	0.0014491
**ENSG00000177830**	CHID1	-0.578772015	3.96273758	14.87629738	0.0002924	0.0014577
**ENSG00000081913**	PHLPP1	0.522794608	3.848393429	14.87533861	0.0002926	0.0014577
**ENSG00000254901**	BORCS8	-0.583715469	4.28274966	14.87533183	0.0002926	0.0014577
**ENSG00000185278**	ZBTB37	-0.463029371	6.858982612	15.62261755	0.0002933	0.0014604
**ENSG00000253729**	PRKDC	0.2200719	8.603362141	14.85672436	0.0002949	0.0014671
**ENSG00000153815**	CMIP	0.335475284	5.929199686	14.85671852	0.0002949	0.0014671
**ENSG00000126749**	EMG1	-0.349083249	5.521924238	14.83972758	0.0002970	0.0014768
**ENSG00000153487**	ING1	0.450670561	5.086517734	14.83874456	0.0002971	0.0014768
**ENSG00000119844**	AFTPH	0.255596174	6.594789576	14.8357948	0.0002975	0.0014778
**ENSG00000112282**	MED23	-0.305063223	6.88807528	14.83494463	0.0002976	0.0014778
**ENSG00000205413**	SAMD9	0.337223827	7.877304381	15.16474131	0.0002984	0.0014809

**ENSG00000266947**		-0.637393939	3.962089041	14.82155494	0.0002993	0.0014847
**ENSG00000132600**	PRMT7	-0.404220797	5.429512397	14.80744279	0.0003010	0.0014929
**ENSG00000100422**	CERK	0.240002932	7.334761235	14.79187367	0.0003030	0.0015020
**ENSG00000262413**		0.512133269	5.008286386	14.77059148	0.0003058	0.0015148
**ENSG00000100647**	SUSD6	0.327239647	6.099392875	14.76591559	0.0003064	0.0015170
**ENSG00000237914**	SIRPG-AS1	-0.714169496	3.930623534	14.75298162	0.0003080	0.0015246
**ENSG00000162976**	PQLC3	0.477084075	4.992236937	14.7462092	0.0003089	0.0015282
**ENSG00000163466**	ARPC2	0.22552036	8.172815037	14.7389374	0.0003099	0.0015322
**ENSG00000143882**	ATP6V1C2	0.502599722	4.960862746	14.72867417	0.0003112	0.0015381
**ENSG00000198898**	CAPZA2	0.321599282	6.258786588	14.70930154	0.0003138	0.0015500
**ENSG00000179119**	SPTY2D1	0.34763889	5.796102676	14.69523638	0.0003156	0.0015582
**ENSG00000134287**	ARF3	0.37034279	5.721884289	14.69462263	0.0003157	0.0015582
**ENSG00000170445**	HARS	-0.371037302	5.686348486	14.6915825	0.0003161	0.0015594
**ENSG00000261611**		-0.677090599	3.999465582	14.67620383	0.0003182	0.0015688
**ENSG00000257093**	KIAA1147	-0.269658264	7.1928962	14.6673757	0.0003194	0.0015740
**ENSG00000100605**	ITPK1	0.505119638	4.538379246	14.66089453	0.0003202	0.0015775
**ENSG00000223547**	ZNF844	-0.682542248	4.420096123	14.74986076	0.0003207	0.0015793
**ENSG00000166188**	ZNF319	0.580593866	4.444990255	14.64729432	0.0003221	0.0015851
**ENSG00000065675**	PRKCQ	0.231095784	6.978307924	14.64011557	0.0003231	0.0015892
**ENSG00000272316**		-0.766672149	3.677051029	14.63369569	0.0003239	0.0015927
**ENSG00000122068**	FYTTD1	0.310823125	6.606823679	14.62863132	0.0003246	0.0015954
**ENSG00000220205**	VAMP2	-0.334352376	6.735722856	14.61231528	0.0003269	0.0016054
**ENSG00000173875**	ZNF791	0.329315381	6.358542524	14.61164539	0.0003270	0.0016054
**ENSG00000148187**	MRRF	-0.446225322	5.259403239	14.60595203	0.0003278	0.0016081
**ENSG00000197302**	LOC107983990	-0.35846467	5.48413627	14.60546297	0.0003278	0.0016081
**ENSG00000197948**	FCHSD1	-0.534248733	5.423407541	14.87278783	0.0003287	0.0016118
**ENSG00000109911**	ELP4	-0.402747777	5.898720028	14.58545345	0.0003306	0.0016202
**ENSG00000151657**	KIN	-0.499820544	4.948599261	14.58207604	0.0003311	0.0016213
**ENSG00000226742**	Hsbp1l1	-0.686902551	3.767824373	14.58157135	0.0003311	0.0016213
**ENSG00000187097**	ENTPD5	-0.48494183	5.013853366	14.56336067	0.0003337	0.0016331
**ENSG00000198198**	SZT2	-0.3545384	7.22672206	14.8405237	0.0003358	0.0016426
**ENSG00000121741**	ZMYM2	-0.209586219	7.833700536	14.54472659	0.0003363	0.0016445
**ENSG00000127054**	MIR6727	-0.408519804	5.894297006	14.53338456	0.0003380	0.0016516
**ENSG00000197275**	RAD54B	-0.749166802	3.661687155	14.53163961	0.0003382	0.0016521
**ENSG00000111364**	DDX55	-0.402158166	5.266968306	14.52993783	0.0003385	0.0016525
**ENSG00000160124**	CCDC58	-0.771372857	3.595899916	14.52796806	0.0003387	0.0016531
**ENSG00000196597**	ZNF782	-0.573783726	4.382016841	14.52332055	0.0003394	0.0016556

**ENSG00000187189**	TSPYL4	-0.474665766	4.953778346	14.50682332	0.0003418	0.0016664
**ENSG00000258461**		-0.648473447	4.222099336	14.49862432	0.0003430	0.0016714
**ENSG00000273329**		-0.684549373	4.406197689	14.59356443	0.0003433	0.0016720
**ENSG00000075336**	TIMM21	-0.683961058	3.74393408	14.49557429	0.0003434	0.0016720
**ENSG00000124224**		-0.649780796	4.375971957	14.48337122	0.0003452	0.0016799
**ENSG00000185261**	KIAA0825	0.709472872	3.861013763	14.48232612	0.0003453	0.0016799
**ENSG00000198843**	SELENOT	0.346962657	6.103990136	14.47859599	0.0003459	0.0016817
**ENSG00000162777**	DENND2D	-0.250738133	7.813739486	14.46963268	0.0003472	0.0016873
**ENSG00000175066**	GK5	-0.518643989	5.304643987	14.58339446	0.0003487	0.0016940
**ENSG00000127314**	RAP1B	-0.255900898	7.603623199	14.45792905	0.0003489	0.0016940
**ENSG00000001497**	LAS1L	-0.372308027	5.30100121	14.45698334	0.0003491	0.0016940
**ENSG00000131446**	MGAT1	0.308411037	6.352413061	14.4451346	0.0003508	0.0017018
**ENSG00000153989**	NUS1	0.351336241	5.770296103	14.44305046	0.0003511	0.0017025
**ENSG00000171503**	ETFDH	-0.501943354	4.600693005	14.44147566	0.0003514	0.0017027
**ENSG00000167202**	TBC1D2B	0.251032592	6.64734948	14.44054618	0.0003515	0.0017027
**ENSG00000149577**	SIDT2	-0.42106369	5.339981319	14.43338866	0.0003526	0.0017071
**ENSG00000204622**	HLA-J	0.511030939	4.923815121	14.42856793	0.0003533	0.0017095
**ENSG00000087157**	PGS1	-0.452468148	5.087232425	14.42784793	0.0003534	0.0017095
**ENSG00000257242**	LINC01619	-0.392513808	6.23721157	14.53513168	0.0003540	0.0017112
**ENSG00000243696**		-0.634412203	4.623281275	14.53250211	0.0003541	0.0017112
**ENSG00000275835**	TUBGCP5	-0.397855266	5.393606104	14.42227609	0.0003542	0.0017112
**ENSG00000070010**	UFD1L	-0.344042639	5.5973334	14.413899	0.0003555	0.0017165
**ENSG00000164620**	RELL2	-0.659081644	3.786046709	14.40490673	0.0003569	0.0017218
**ENSG00000154114**	TBCEL	-0.506341483	4.714884842	14.40451482	0.0003569	0.0017218
**ENSG00000197714**	ZNF460	0.304218965	5.951200644	14.40341821	0.0003571	0.0017218
**ENSG00000165732**	DDX21	0.262269936	7.217740569	14.39935397	0.0003577	0.0017239
**ENSG00000112305**	SMAP1	0.336689631	6.032872864	14.38249304	0.0003603	0.0017355
**ENSG00000183562**		-0.67407835	4.654578282	14.60967022	0.0003661	0.0017628
**ENSG00000028528**	SNX1	-0.269960837	6.867461998	14.33279527	0.0003680	0.0017709
**ENSG00000198496**	NBR2	-0.703117528	3.658866249	14.32716614	0.0003688	0.0017743
**ENSG00000145365**	TIFA	0.450856085	5.009440979	14.32582815	0.0003690	0.0017745
**ENSG00000108854**	SMURF2	0.328769622	6.350276521	14.3127952	0.0003711	0.0017835
**ENSG00000188612**	SUMO2	0.333857841	6.74657211	14.31174686	0.0003713	0.0017835
**ENSG00000238083**	LRRC37A2	-0.434826754	5.328211613	14.29849675	0.0003734	0.0017927
**ENSG00000179406**	LINC00174	-0.536081017	4.495793471	14.29619175	0.0003737	0.0017936
**ENSG00000185880**	TRIM69	0.426317132	5.580553786	14.28634744	0.0003753	0.0018003
**ENSG00000070669**	ASNS	-0.407948567	4.998973292	14.27230093	0.0003775	0.0018103

**ENSG00000100418**	DESI1	-0.417411133	4.767271855	14.26996964	0.0003779	0.0018112
**ENSG00000112208**	BAG2	-0.517938593	4.316168423	14.26542523	0.0003786	0.0018139
**ENSG00000242372**	EIF6	0.601099656	3.66105401	14.26348605	0.0003789	0.0018146
**ENSG00000279649**		-0.464056556	5.146851493	14.2586255	0.0003797	0.0018175
**ENSG00000182827**	ACBD3	0.38222279	5.825272545	14.25743961	0.0003799	0.0018176
**ENSG00000173281**	PPP1R3B	0.664598039	4.366085199	14.26558869	0.0003819	0.0018264
**ENSG00000121966**	CXCR4	0.348855727	8.395838157	14.70381909	0.0003829	0.0018301
**ENSG00000106355**	LSM 5.00	-0.547566709	4.680813956	14.23798649	0.0003831	0.0018301
**ENSG00000068394**	GPKOW	0.554379747	4.199746795	14.2369462	0.0003832	0.0018301
**ENSG00000132646**	PCNA	0.478263733	4.864532464	14.21498256	0.0003868	0.0018465
**ENSG00000135974**	c2orf49	-0.429753551	5.353586987	14.20914843	0.0003878	0.0018502
**ENSG00000141378**	PTRH2	0.322305564	5.686084473	14.18763219	0.0003914	0.0018664
**ENSG00000131791**	PRKAB2	-0.575902393	5.021010559	14.35024875	0.0003933	0.0018749
**ENSG00000173611**	SCAI	-0.389612644	5.884776749	14.17321777	0.0003938	0.0018762
**ENSG00000163626**	cox18	-0.513630205	4.719914275	14.16757675	0.0003947	0.0018798
**ENSG00000136451**	VEZF1	0.272713344	6.907183105	14.1520761	0.0003973	0.0018914
**ENSG00000185619**	PCGF3	-0.609027178	6.447022772	15.1035742	0.0004017	0.0019107
**ENSG00000101152**	DNAJC5	0.310659513	5.732616154	14.12610348	0.0004018	0.0019107
**ENSG00000128590**	DNAJB9	0.446945189	5.501641393	14.11799836	0.0004032	0.0019164
**ENSG00000104365**	IKBKB	-0.363329003	6.658908677	14.2388928	0.0004041	0.0019199
**ENSG00000148334**	PTGES2	-0.704897311	3.90225068	14.10594974	0.0004052	0.0019246
**ENSG00000172315**	TP53RK	0.434609834	5.11644655	14.10437781	0.0004055	0.0019250
**ENSG00000151806**	GUF1	-0.346699796	5.838053812	14.09910409	0.0004064	0.0019284
**ENSG00000141452**	c18orf8	0.330691448	5.913137024	14.08197221	0.0004094	0.0019417
**ENSG00000166436**	TRIM66	-0.704212791	4.888407051	14.60259312	0.0004124	0.0019552
**ENSG00000065135**	gnai3	0.268400076	6.924511074	14.05810663	0.0004136	0.0019598
**ENSG00000052795**	FNIP2	-0.523466328	4.62310695	14.05661802	0.0004138	0.0019601
**ENSG00000234072**		-0.716868402	3.703215612	14.05338037	0.0004144	0.0019620
**ENSG00000137221**	TJAP1	-0.536738137	4.857908479	14.04951004	0.0004151	0.0019643
**ENSG00000144228**	SPOPL	0.431720097	5.430556787	14.04597225	0.0004157	0.0019664
**ENSG00000270181**	BIVM-ERCC5	-0.22385387	7.522499062	14.04259158	0.0004163	0.0019683
**ENSG00000178199**	ZC3H12D	-0.353156025	6.087138159	14.03805395	0.0004171	0.0019713
**ENSG00000105875**	WDR91	-0.531023068	4.540779804	14.02571067	0.0004193	0.0019808
**ENSG00000105701**	FKBP8	0.421599528	5.286906525	14.01818469	0.0004207	0.0019863
**ENSG00000266472**	MRPS21	-0.458698835	5.0141686	14.01329111	0.0004216	0.0019895
**ENSG00000125459**	MSTO1	-0.638582257	3.732589118	13.99651182	0.0004246	0.0020029
**ENSG00000205189**	ZBTB10	-0.371892555	5.553277646	13.98024234	0.0004276	0.0020160

**ENSG00000023041**	ZDHHC6	-0.349889274	5.933083252	13.97726239	0.0004281	0.0020174
**ENSG00000225032**	LOC102723566	-0.84842344	3.629348033	14.11140047	0.0004282	0.0020174
**ENSG00000165699**	TSC1	-0.321422537	6.307898662	13.97066547	0.0004293	0.0020215
**ENSG00000225636**		-0.669147206	3.540753068	13.95589514	0.0004320	0.0020334
**ENSG00000236472**		0.575054487	3.926390295	13.94948652	0.0004332	0.0020380
**ENSG00000143549**	TPM3	0.24471517	8.400349469	13.94109219	0.0004348	0.0020442
**ENSG00000198218**	QRICH1	-0.233229744	7.179876253	13.94034404	0.0004349	0.0020442
**ENSG00000198612**	COPS8	-0.35172954	5.558990042	13.93453912	0.0004360	0.0020482
**ENSG00000143493**	INTS7	0.44473975	4.974896391	13.93307506	0.0004363	0.0020482
**ENSG00000214176**		-0.549078506	5.89141597	14.5808672	0.0004363	0.0020482
**ENSG00000185728**	YTHDF3	0.232039766	6.96512231	13.92773589	0.0004373	0.0020515
**ENSG00000119414**	PPP6C	0.233391666	7.070198212	13.91914617	0.0004389	0.0020582
**ENSG00000113240**	CLK4	-0.397600138	6.833601293	14.33495001	0.0004392	0.0020588
**ENSG00000198900**	TOP 1.00	0.312310885	6.870184864	13.91309403	0.0004400	0.0020617
**ENSG00000188033**	ZNF490	0.519114927	4.614974328	13.89667233	0.0004431	0.0020753
**ENSG00000146776**	ATXN7L1	0.264898294	6.496581858	13.89219893	0.0004440	0.0020783
**ENSG00000014138**	POLA2	-0.511218255	4.400663334	13.88499997	0.0004453	0.0020838
**ENSG00000125817**	CENPB	0.476767121	4.658358697	13.86901272	0.0004484	0.0020971
**ENSG00000105323**	HNRNPUL1	0.237113573	7.914227398	13.86225076	0.0004497	0.0021023
**ENSG00000166224**	SGPL1	0.377620139	5.358126231	13.85543846	0.0004510	0.0021075
**ENSG00000182628**	SKA2	0.399977136	4.878875513	13.85353451	0.0004514	0.0021083
**ENSG00000269737**		-0.479835957	4.207697958	13.85137265	0.0004518	0.0021093
**ENSG00000114735**	HEMK1	-0.475232104	5.909746103	14.19485148	0.0004550	0.0021233
**ENSG00000225648**	SBDSP1	-0.39469275	5.284924183	13.83141299	0.0004557	0.0021255
**ENSG00000232940**	HCG25	-0.656311106	3.732409314	13.82165677	0.0004576	0.0021334
**ENSG00000124151**	NCOA3	0.203253476	7.920652516	13.81982387	0.0004579	0.0021342
**ENSG00000100811**	MIR6764	0.244097614	7.389167399	13.81579303	0.0004587	0.0021369
**ENSG00000115085**	ZAP70	-0.456327515	7.16471008	14.6026218	0.0004591	0.0021377
**ENSG00000174946**	GPR171	0.378089111	6.65401262	14.0056352	0.0004632	0.0021559
**ENSG00000151702**	FLI1	0.20258278	7.458388314	13.78940415	0.0004639	0.0021583
**ENSG00000207445**	SNORD15B	1.294077757	5.34805552	14.79231337	0.0004664	0.0021686
**ENSG00000167524**	SGK494	-0.72157488	4.15168613	13.87062859	0.0004668	0.0021695
**ENSG00000204632**	HLA-G	0.531153084	4.852317488	13.76788207	0.0004682	0.0021754
**ENSG00000143669**	LYST	0.306096748	7.072031272	13.75973267	0.0004699	0.0021821
**ENSG00000106591**	MRPL32	-0.479465089	4.886219257	13.74138534	0.0004736	0.0021983
**ENSG00000130305**	NSUN5	-0.54841944	4.534688897	13.72572215	0.0004768	0.0022120
**ENSG00000145780**	FEM1C	0.326492862	6.123838396	13.7248124	0.0004770	0.0022120

**ENSG00000172590**	mrpl52	-0.594780866	4.286226354	13.72144083	0.0004777	0.0022143
**ENSG00000198363**	ASPH	-0.473089828	4.940337426	13.71731747	0.0004785	0.0022172
**ENSG00000040275**	SPDL1	-0.645215656	3.875690858	13.71455377	0.0004791	0.0022189
**ENSG00000114331**	ACAP2	0.192363753	7.950085381	13.71283256	0.0004794	0.0022192
**ENSG00000234222**	LOC105371260	0.306794725	5.460314347	13.71211738	0.0004796	0.0022192
**ENSG00000206527**	HACD2	0.365184793	5.86544479	13.6944836	0.0004832	0.0022351
**ENSG00000148396**	SEC16A	0.231120522	7.462474049	13.69245031	0.0004837	0.0022360
**ENSG00000087470**	DNM1L	-0.264499291	6.5936943	13.69143014	0.0004839	0.0022360
**ENSG00000130584**	ZBTB46	-0.704160208	3.989865821	13.68481787	0.0004852	0.0022414
**ENSG00000239969**		-0.847105921	3.52816935	13.78528365	0.0004862	0.0022450
**ENSG00000243302**		-0.567746066	4.831289848	13.75361126	0.0004874	0.0022494
**ENSG00000278600**		-0.623227557	4.155999378	13.66295959	0.0004898	0.0022596
**ENSG00000067182**	TNFRSF1A	0.395838517	5.231842534	13.6595206	0.0004905	0.0022619
**ENSG00000127483**	HP1BP3	-0.23143067	8.188009992	13.64851849	0.0004929	0.0022716
**ENSG00000165689**	SDCCAG3	-0.522447525	5.016884543	13.68661416	0.0004938	0.0022748
**ENSG00000150787**	PTS	-0.678890858	4.010054823	13.64098706	0.0004945	0.0022770
**ENSG00000137200**	CMTR1	-0.389962823	5.67135952	13.63978278	0.0004947	0.0022771
**ENSG00000104613**	INTS10	-0.286652346	6.235346456	13.62470232	0.0004979	0.0022909
**ENSG00000283774**		-0.491947884	4.109571038	13.60925312	0.0005013	0.0023052
**ENSG00000228060**	LOC101929516	-0.447478159	4.605264092	13.60183836	0.0005029	0.0023115
**ENSG00000168010**	atg16l2	-0.560947852	6.29711069	14.42225033	0.0005041	0.0023161
**ENSG00000163029**	SMC6	0.366985487	6.006511991	13.58967739	0.0005055	0.0023216
**ENSG00000229124**	VIM-AS1	0.550117378	7.122005145	14.51935911	0.0005069	0.0023269
**ENSG00000111850**	SMIM8	-0.574914893	3.995412881	13.56158169	0.0005116	0.0023478
**ENSG00000122705**	CLTA	0.468351729	4.977579389	13.53794582	0.0005169	0.0023701
**ENSG00000085719**	CPNE3	0.296901665	6.674755798	13.53760647	0.0005169	0.0023701
**ENSG00000010017**	RANBP9	0.264681988	6.550314503	13.52915104	0.0005188	0.0023777
**ENSG00000085760**	MTIF2	-0.407463201	5.218979001	13.50542726	0.0005242	0.0024011
**ENSG00000006007**	GDE1	0.538979047	4.729198073	13.50288837	0.0005247	0.0024019
**ENSG00000115446**	UNC50	-0.426557315	4.973283949	13.50128737	0.0005251	0.0024019
**ENSG00000177646**	ACAD9	-0.473419728	4.768657183	13.50027322	0.0005253	0.0024019
**ENSG00000164975**	SNAPC3	-0.303881707	6.816907616	13.50026791	0.0005253	0.0024019
**ENSG00000164535**	DAGLB	-0.423029624	4.827055005	13.49950438	0.0005255	0.0024019
**ENSG00000239264**	TXNDC5	0.552262563	4.378453666	13.4926774	0.0005271	0.0024079
**ENSG00000158773**	USF1	-0.400033152	4.776919299	13.48904265	0.0005279	0.0024107
**ENSG00000163161**	ERCC3	-0.391508546	5.633760916	13.48139371	0.0005296	0.0024176
**ENSG00000134215**	VAV3	0.641188619	4.306071234	13.44952169	0.0005371	0.0024507

**ENSG00000214182**		0.719980121	4.689521577	13.91236915	0.0005387	0.0024570
**ENSG00000130713**	EXOSC2	-0.452891298	5.022279311	13.43193915	0.0005410	0.0024665
**ENSG00000066651**	TRMT11	-0.43149728	5.006993977	13.43065063	0.0005413	0.0024668
**ENSG00000214413**	BBIP1	-0.245082415	7.161531139	13.42413848	0.0005429	0.0024716
**ENSG00000031003**	FAM13B	0.270908574	6.893821708	13.42404781	0.0005429	0.0024716
**ENSG00000232869**		0.509983329	4.657132726	13.42244595	0.0005433	0.0024723
**ENSG00000272752**	STAG3L5P-PVRI	-0.744655892	6.300931278	14.37477525	0.0005439	0.0024741
**ENSG00000035141**	FAM136A	-0.550311565	4.329904787	13.418488	0.0005442	0.0024743
**ENSG00000280137**		-0.610488926	4.791128935	13.6278791	0.0005450	0.0024767
**ENSG00000139618**	brca2	0.693695603	4.241347137	13.49533543	0.0005459	0.0024798
**ENSG00000111790**	FGFR1OP2	0.243527727	7.156756963	13.40524157	0.0005473	0.0024853
**ENSG00000134452**	FBXO18	-0.341969789	5.681245605	13.38250878	0.0005527	0.0025087
**ENSG00000143819**	EPHX1	-0.577832211	4.572013301	13.37661504	0.0005541	0.0025140
**ENSG00000113504**	SLC12A7	-0.5186808	5.297209014	13.52805681	0.0005551	0.0025165
**ENSG00000158006**	PAFAH2	-0.534922573	4.288898015	13.37231507	0.0005551	0.0025165
**ENSG00000156232**	WHAMM	-0.344689565	5.966087319	13.36964484	0.0005558	0.0025183
**ENSG00000145817**	YIPF5	0.39040743	5.600769319	13.36645308	0.0005566	0.0025207
**ENSG00000271533**		-0.714612326	5.277211452	14.11934886	0.0005570	0.0025216
**ENSG00000182831**	C16orf72	0.21201928	8.606799912	13.35515069	0.0005593	0.0025308
**ENSG00000092140**	G2E3	-0.311582661	6.080943292	13.35162465	0.0005601	0.0025336
**ENSG00000116604**	MEF2D	0.341883627	6.012390285	13.34339493	0.0005621	0.0025415
**ENSG00000096968**	JAK2	0.369896884	5.932997689	13.33896604	0.0005632	0.0025432
**ENSG00000081307**	UBA5	-0.388921654	5.701025086	13.3384858	0.0005633	0.0025432
**ENSG00000111817**	DSE	0.360145308	5.519442049	13.33830072	0.0005634	0.0025432
**ENSG00000128694**	OSGEPL1	-0.501266976	4.500302591	13.33789227	0.0005635	0.0025432
**ENSG00000120963**	ZNF706	-0.324428941	6.095951695	13.3303642	0.0005653	0.0025504
**ENSG00000071626**	DAZAP1	-0.364114454	5.949134387	13.32514129	0.0005666	0.0025550
**ENSG00000108825**	PTGES3L-AARS	-0.648733565	3.443569796	13.32264123	0.0005672	0.0025567
**ENSG00000259522**		-0.501244015	4.578251968	13.31024211	0.0005702	0.0025693
**ENSG00000254469**		-0.574030125	3.984076838	13.29282917	0.0005745	0.0025876
**ENSG00000128923**	FAM63B	0.311925716	5.987105997	13.28633191	0.0005762	0.0025938
**ENSG00000122515**	ZMIZ2	-0.478612507	6.245649426	13.84679393	0.0005765	0.0025940
**ENSG00000136059**	VILL	-0.503325049	4.530082868	13.27858858	0.0005781	0.0026002
**ENSG00000151743**	AMN1	-0.562744336	4.054025759	13.27215041	0.0005797	0.0026064
**ENSG00000245532**	NEAT1	-0.612009424	10.03882095	14.20302855	0.0005815	0.0026132
**ENSG00000110660**	SLC35F2	0.68344015	3.44568263	13.25869893	0.0005831	0.0026189
**ENSG00000138641**	LOC101929134	-0.220920024	7.213469649	13.2581095	0.0005832	0.0026189

**ENSG00000140474**	ULK3	-0.58020821	4.983314817	13.45111085	0.0005846	0.0026240
**ENSG00000106351**	AGFG2	0.423767063	5.28575245	13.23388894	0.0005894	0.0026442
**ENSG00000101544**	ADNP2	0.331817292	5.572593677	13.2321403	0.0005898	0.0026448
**ENSG00000281593**		0.604818861	3.748004759	13.23063867	0.0005902	0.0026448
**ENSG00000273269**		0.425514422	5.222108498	13.23043785	0.0005903	0.0026448
**ENSG00000119596**	YLPM1	-0.255577483	7.531562233	13.22778389	0.0005910	0.0026467
**ENSG00000185813**	PCYT2	0.614941087	3.743329242	13.22422958	0.0005919	0.0026492
**ENSG00000071537**	SEL1L	0.254334032	7.086037022	13.22361267	0.0005920	0.0026492
**ENSG00000132824**	SERINC3	0.220877433	7.497858137	13.2168518	0.0005938	0.0026558
**ENSG00000140990**	NDUFB10	0.452139804	4.468971253	13.21173976	0.0005951	0.0026598
**ENSG00000136997**	MYC	-0.280459201	7.238283032	13.211435	0.0005952	0.0026598
**ENSG00000136490**	LIMD2	-0.272425639	6.878012057	13.20899931	0.0005958	0.0026615
**ENSG00000205784**	ARRDC5	-0.609030354	3.593870853	13.20795178	0.0005960	0.0026615
**ENSG00000169718**	DUS1L	-0.437153305	5.275691513	13.19255363	0.0006000	0.0026782
**ENSG00000267278**	MAP3K14-AS1	-0.529222179	4.141658376	13.18848364	0.0006011	0.0026818
**ENSG00000101365**	IDH3B	-0.447782248	4.592434726	13.18605109	0.0006017	0.0026835
**ENSG00000168298**	HIST1H1E	0.529504434	8.277892263	14.08636933	0.0006044	0.0026943
**ENSG00000103091**	WDR59	-0.467195215	5.618855419	13.33201061	0.0006091	0.0027141
**ENSG00000101109**	STK4	0.192456303	9.312459901	13.14362597	0.0006129	0.0027298
**ENSG00000160588**	MPZL3	0.430505225	5.137924724	13.13342902	0.0006156	0.0027407
**ENSG00000107362**	ABHD17B	0.503734934	4.488436636	13.11788505	0.0006198	0.0027573
**ENSG00000163281**	GNPDA2	-0.349111953	5.471136632	13.11756769	0.0006199	0.0027573
**ENSG00000069493**	CLEC2D	-0.219831141	8.986726632	13.10738851	0.0006226	0.0027682
**ENSG00000078043**	PIAS2	-0.341329083	6.152402392	13.1065367	0.0006228	0.0027682
**ENSG00000142687**	KIAA0319L	-0.341953652	6.056724882	13.10480253	0.0006233	0.0027691
**ENSG00000184154**	LRTOMT	-0.472985694	4.349610959	13.09915197	0.0006248	0.0027747
**ENSG00000197744**		0.70884716	3.867600053	13.09450339	0.0006261	0.0027791
**ENSG00000125375**	ATP5S	-0.362238621	5.786221837	13.079129	0.0006303	0.0027965
**ENSG00000173559**	NABP1	0.463327336	7.556427886	13.89741156	0.0006312	0.0027995
**ENSG00000184863**	RBM33	-0.202490441	7.91686978	13.07379465	0.0006318	0.0028007
**ENSG00000115808**	STRN	0.260848251	6.82989353	13.07023205	0.0006327	0.0028030
**ENSG00000131196**	NFATC1	0.297812329	6.128046974	13.06991102	0.0006328	0.0028030
**ENSG00000266967**	AARSD1	-0.624899841	3.830810791	13.06430912	0.0006344	0.0028086
**ENSG00000130684**	ZNF337	-0.383412972	5.201086782	13.05965241	0.0006357	0.0028131
**ENSG00000112983**	BRD8	-0.335650092	5.691129845	13.05757257	0.0006362	0.0028145
**ENSG00000147996**	CBWD5	-0.548859746	4.269639578	13.05571899	0.0006367	0.0028156
**ENSG00000124688**	MAD2L1BP	0.682045218	3.638586147	13.05467875	0.0006370	0.0028156

**ENSG00000270024**	C8orf44-SGK3	0.34303398	5.113504025	13.05328987	0.0006374	0.0028162
**ENSG00000244676**		0.586315604	3.685397045	13.04166785	0.0006406	0.0028292
**ENSG00000137413**	TAF8	-0.303126876	5.779604511	13.03318287	0.0006430	0.0028385
**ENSG00000149532**	CPSF7	-0.277404158	6.433145844	13.01555737	0.0006480	0.0028591
**ENSG00000112406**	HECA	0.183879948	8.486036744	13.01031558	0.0006494	0.0028644
**ENSG00000138279**	ANXA7	0.262534271	6.389931401	13.0066174	0.0006505	0.0028678
**ENSG00000115956**	PLEK	1.068885534	4.341933803	13.77271716	0.0006515	0.0028713
**ENSG00000111711**	GOLT1B	-0.328806317	5.517625082	12.98815608	0.0006557	0.0028885
**ENSG00000145439**	CBR4	-0.438346889	4.955392049	12.95543662	0.0006651	0.0029287
**ENSG00000101695**	RNF125	0.231521528	7.236557937	12.94305057	0.0006687	0.0029433
**ENSG00000164039**	BDH2	-0.586064848	4.055082857	12.94181278	0.0006691	0.0029436
**ENSG00000102316**	MAGED2	0.371164106	5.001546514	12.94025487	0.0006695	0.0029444
**ENSG00000130813**	c19orf66	-0.419995738	5.345152872	12.93767631	0.0006703	0.0029464
**ENSG00000178209**	PLEC	0.361424595	8.831103273	13.48405511	0.0006722	0.0029538
**ENSG00000138835**	RGS3	0.574684375	3.84367404	12.92758483	0.0006732	0.0029569
**ENSG00000163875**	MEAF6	0.29230112	5.849570585	12.92541758	0.0006739	0.0029585
**ENSG00000256966**	FBXO10	0.544394219	4.241504133	12.92319855	0.0006745	0.0029601
**ENSG00000184716**	SERINC4	-0.459671188	4.678158572	12.9195291	0.0006756	0.0029636
**ENSG00000262831**		0.456117959	4.753316469	12.9171621	0.0006763	0.0029654
**ENSG00000115875**	SRSF7	-0.3458395	7.801685883	13.35762751	0.0006766	0.0029656
**ENSG00000180104**	EXOC3	-0.293605048	6.128750711	12.91504266	0.0006769	0.0029656
**ENSG00000157353**	FUK	-0.549136622	3.986246912	12.89905834	0.0006817	0.0029851
**ENSG00000281195**		-0.575519331	4.74650951	12.99970846	0.0006822	0.0029862
**ENSG00000162971**	TYW5	-0.431842376	5.326484108	12.88834288	0.0006848	0.0029966
**ENSG00000225339**		0.271470856	6.537976708	12.87029022	0.0006903	0.0030190
**ENSG00000118985**	ELL2	0.501394736	4.188426916	12.85714082	0.0006942	0.0030331
**ENSG00000161551**	ZNF577	-0.508714377	4.468756017	12.8570192	0.0006943	0.0030331
**ENSG00000111335**	OAS2	0.27228226	7.295287029	12.8567014	0.0006944	0.0030331
**ENSG00000131374**	TBC1D5	0.266609842	6.574422673	12.85314428	0.0006954	0.0030365
**ENSG00000272529**		-0.670544892	4.13195834	12.84251784	0.0006987	0.0030494
**ENSG00000104047**	DTWD1	-0.412850993	4.994064385	12.838376	0.0006999	0.0030536
**ENSG00000162636**	FAM102B	0.364267578	5.368207235	12.83314267	0.0007015	0.0030593
**ENSG00000181754**	AMIGO1	-0.392400446	5.511341057	12.81039857	0.0007085	0.0030885
**ENSG00000164398**	ACSL6	0.313090698	5.822817163	12.80259877	0.0007109	0.0030967
**ENSG00000184575**	XPOT	0.240287749	6.850240087	12.80243246	0.0007110	0.0030967
**ENSG00000197857**	ZNF44	-0.301903235	5.749074725	12.7964382	0.0007128	0.0031035
**ENSG00000264364**	DYNLL2	0.284809461	6.922787996	12.77611149	0.0007192	0.0031299

**ENSG00000112309**	B3GAT2	0.365474159	5.445297084	12.77469492	0.0007196	0.0031305
**ENSG00000126217**	MCF2L	-0.680226026	3.955984876	12.76082363	0.0007240	0.0031482
**ENSG00000157873**	TNFRSF14	-0.354605488	5.945183509	12.74720227	0.0007283	0.0031657
**ENSG00000161267**	BDH1	-0.431586736	4.793519195	12.73845343	0.0007311	0.0031765
**ENSG00000164091**	WDR82	0.209935631	8.15302634	12.72815345	0.0007344	0.0031895
**ENSG00000110852**	CLEC2B	-0.292463552	6.338902527	12.72710266	0.0007348	0.0031897
**ENSG00000182253**	SYNM	0.778118248	3.840610285	12.87228427	0.0007360	0.0031921
**ENSG00000162775**	RBM15	0.300791188	6.071006824	12.7227399	0.0007362	0.0031921
**ENSG00000133134**	BEX2	-0.586178023	3.6306445	12.72253	0.0007362	0.0031921
**ENSG00000230084**		-0.608163307	4.719005611	12.91180716	0.0007374	0.0031946
**ENSG00000226752**		-0.327580968	5.971369344	12.71883437	0.0007374	0.0031946
**ENSG00000248487**	ABHD14A	0.565729036	4.113763741	12.71583844	0.0007384	0.0031974
**ENSG00000136271**	DDX56	-0.456661312	5.450667497	12.78612073	0.0007389	0.0031978
**ENSG00000069956**	MAPK6	0.362547087	5.564636212	12.71364734	0.0007391	0.0031978
**ENSG00000134262**	AP4B1	-0.532137897	5.353196191	13.01466845	0.0007410	0.0032048
**ENSG00000198105**	ZNF248	-0.447030466	4.752651637	12.70588291	0.0007416	0.0032061
**ENSG00000263276**		0.351476048	5.682086149	12.69441235	0.0007453	0.0032209
**ENSG00000165006**	UBAP1	0.353478344	5.365536067	12.6852009	0.0007484	0.0032325
**ENSG00000146701**	MDH2	0.331471769	5.709333729	12.68210879	0.0007494	0.0032344
**ENSG00000126247**	CAPNS1	0.384336044	6.209222664	12.83892064	0.0007496	0.0032344
**ENSG00000106560**	GIMAP2	-0.280433769	6.944079159	12.68107255	0.0007497	0.0032344
**ENSG00000274049**	INO80B-WBP1	-0.529845131	4.574740609	12.66743059	0.0007542	0.0032524
**ENSG00000197713**	RPE	-0.359482614	5.717628145	12.653918	0.0007587	0.0032703
**ENSG00000165417**	GTF2A1	0.28729169	6.843933702	12.62769754	0.0007674	0.0033067
**ENSG00000172059**	KLF11	0.570769185	4.206089737	12.62308982	0.0007690	0.0033120
**ENSG00000275023**	MLLT6	-0.254857679	8.289148527	12.62214701	0.0007693	0.0033120
**ENSG00000111252**	SH2B3	0.285579899	5.970167238	12.61746058	0.0007709	0.0033175
**ENSG00000087448**	KLHL42	-0.420240035	5.127876368	12.60960582	0.0007735	0.0033273
**ENSG00000115760**	BIRC6	0.195212758	9.19690408	12.60884156	0.0007738	0.0033273
**ENSG00000156599**	ZDHHC5	0.269846475	6.270726001	12.60755812	0.0007742	0.0033278
**ENSG00000140688**	c16orf58	-0.444685155	5.132806477	12.60488891	0.0007751	0.0033289
**ENSG00000268205**		0.332862911	5.872982416	12.60397905	0.0007754	0.0033289
**ENSG00000196700**	ZNF512B	-0.47369954	4.575720406	12.60395003	0.0007755	0.0033289
**ENSG00000258441**	LINC00641	-0.450221179	6.190049885	13.01909455	0.0007781	0.0033390
**ENSG00000092531**	SNAP23	-0.293222038	5.9811196	12.59236001	0.0007794	0.0033431
**ENSG00000136950**	ARPC5L	0.423124236	4.727176842	12.58881927	0.0007806	0.0033470
**ENSG00000165272**	AQP3	0.492038615	6.542240552	13.25628843	0.0007817	0.0033502

**ENSG00000100523**	DDHD1	-0.322239676	7.215763252	12.75749522	0.000784	0.003359
**ENSG00000188428**	BLOC1S5	0.455259031	4.924721205	12.57136575	0.000786	0.003368
**ENSG00000136444**	RSAD1	-0.446915199	4.594984792	12.56777216	0.000787	0.003372
**ENSG00000132781**	MUTYH	-0.640894729	3.900028468	12.55976469	0.000790	0.003382
**ENSG00000146067**	FAM193B	-0.594669284	5.747744384	13.21745447	0.000791	0.003387
**ENSG00000184068**		0.455371588	4.30268775	12.54820029	0.000794	0.003397
**ENSG00000267369**		0.528796646	4.932954045	12.61571032	0.000795	0.003399
**ENSG00000211747**		0.463398467	4.735516562	12.54413112	0.000796	0.003400
**ENSG00000255036**	LOC100499484-	-0.44503836	4.85929706	12.54337296	0.000796	0.003400
**ENSG00000110768**	GTF2H1	-0.306455132	5.728035711	12.5399658	0.000797	0.003404
**ENSG00000082641**	NFE2L1	0.33069304	5.696924139	12.5373421	0.000798	0.003406
**ENSG00000188021**	UBQLN2	-0.240813962	7.384795243	12.53632705	0.000798	0.003406
**ENSG00000112096**	LOC100129518	0.235019882	7.14783922	12.52948569	0.000801	0.003415
**ENSG00000125755**	SYMPK	-0.424830999	6.335986226	12.89681359	0.000810	0.003451
**ENSG00000108771**	DHX58	-0.669794385	3.93944043	12.50175019	0.000811	0.003454
**ENSG00000047578**	KIAA0556	-0.502535766	4.260054608	12.50043595	0.000811	0.003455
**ENSG00000151929**	BAG3	-0.475900561	4.777984222	12.49495723	0.000813	0.003462
**ENSG00000139636**	LMBR1L	-0.465856722	5.226958162	12.50470907	0.000815	0.003471
**ENSG00000118200**	CAMSAP2	-0.58301389	4.433060483	12.48117419	0.000818	0.003480
**ENSG00000168040**	FADD	0.548453218	3.943746772	12.46659241	0.000823	0.003501
**ENSG00000281344**		-1.169562969	4.477601899	13.23580085	0.000824	0.003501
**ENSG00000026652**	AGPAT4	-0.527100284	4.298699837	12.46354902	0.000824	0.003503
**ENSG00000096717**	SIRT1	0.231662686	6.490198353	12.45821853	0.000826	0.003509
**ENSG00000095139**	ARCN1	0.233046911	7.249537231	12.45225077	0.000828	0.003517
**ENSG00000140612**	SEC11A	0.360598622	5.627589617	12.43708879	0.000834	0.003539
**ENSG00000059145**	UNKL	-0.403567169	5.235579347	12.42549388	0.000838	0.003556
**ENSG00000258430**		0.523939911	3.818470529	12.42110305	0.000840	0.003560
**ENSG00000060749**	QSER1	0.298498377	6.345030177	12.42104914	0.000840	0.003560
**ENSG00000115415**	STAT1	-0.42211302	8.55788544	13.13417096	0.000842	0.003569
**ENSG00000140931**	CMTM3	0.372833545	5.252144161	12.40577112	0.000845	0.003581
**ENSG00000168758**	SEMA4C	0.419891714	4.972860089	12.40319913	0.000846	0.003584
**ENSG00000238018**		-0.42652669	4.703193226	12.3931687	0.000850	0.003598
**ENSG00000151498**	ACAD8	-0.453517373	4.945802565	12.38867162	0.000852	0.003602
**ENSG00000263753**	LINC00667	-0.411633354	5.611340434	12.38860476	0.000852	0.003602
**ENSG00000175087**	PDIK1L	0.416524757	5.168436229	12.3781422	0.000856	0.003617
**ENSG00000272325**	NUDT3	0.23276355	7.015298149	12.37740444	0.000856	0.003617
**ENSG00000239883**		-0.520339182	4.544195134	12.36983553	0.000859	0.003628

**ENSG00000107758**	PPP3CB	0.224924864	6.676004377	12.36842081	0.0008599	0.0036290
**ENSG00000269987**		-0.439956872	5.238106031	12.36089971	0.0008628	0.0036396
**ENSG00000155463**	OXA1L	-0.246368425	6.841870392	12.35759396	0.0008640	0.0036434
**ENSG00000187257**	RSBN1L	0.265915337	6.807741942	12.35129878	0.0008664	0.0036521
**ENSG00000273891**		-0.632979824	3.865236863	12.34855343	0.0008675	0.0036550
**ENSG00000119714**	GPR68	0.549624545	3.807975304	12.34225575	0.0008699	0.0036637
**ENSG00000227678**		0.443756624	5.015127358	12.33833676	0.0008714	0.0036685
**ENSG00000125450**	NUP85	-0.490986516	5.095063632	12.38859332	0.0008749	0.0036817
**ENSG00000167468**	GPX4	0.399186029	4.998090056	12.32177111	0.0008778	0.0036924
**ENSG00000232593**		-0.595516673	4.870315777	12.55233175	0.0008808	0.0037036
**ENSG00000263345**		-0.832650791	3.927161846	12.58882653	0.0008812	0.0037037
**ENSG00000099860**	GADD45B	0.529636164	3.892661171	12.29007012	0.0008901	0.0037397
**ENSG00000176593**	LOC100128398	-0.525888028	4.440447951	12.28924848	0.0008904	0.0037397
**ENSG00000197879**	MYO1C	0.530007143	4.486264697	12.28396125	0.0008925	0.0037469
**ENSG00000075785**	RAB7A	0.275415805	7.225631409	12.27397361	0.0008965	0.0037606
**ENSG00000134283**	PPHLN1	-0.242065112	6.875751291	12.27348194	0.0008967	0.0037606
**ENSG00000259040**	BLOC1S5-TXND	0.499867834	4.672020572	12.27296945	0.0008969	0.0037606
**ENSG00000198492**	YTHDF2	0.245139308	6.14553985	12.26835896	0.0008987	0.0037664
**ENSG00000120451**	SNX19	-0.230728613	6.518913397	12.26760861	0.0008990	0.0037664
**ENSG00000141424**	SLC39A6	0.337696014	5.199362979	12.26459365	0.0009002	0.0037690
**ENSG00000283761**		0.259315456	6.510502592	12.2634499	0.0009006	0.0037690
**ENSG00000259529**		-0.322308321	6.385512691	12.26328759	0.0009007	0.0037690
**ENSG00000105186**	ANKRD27	0.305717043	5.828614313	12.26241866	0.0009010	0.0037690
**ENSG00000158987**	RAPGEF6	-0.222389787	8.532399874	12.26057388	0.0009018	0.0037706
**ENSG00000056277**	ZNF280C	0.458456993	4.583594494	12.25841799	0.0009026	0.0037716
**ENSG00000258424**		0.261220466	6.358267955	12.25814716	0.0009027	0.0037716
**ENSG00000251369**	ZNF550	-0.56067497	5.178837737	12.52227167	0.0009089	0.0037958
**ENSG00000143612**	c1orf43	0.372274316	5.566377618	12.24100022	0.0009096	0.0037972
**ENSG00000196912**	ANKRD36B	-0.347554645	6.101098716	12.23892614	0.0009104	0.0037992
**ENSG00000225484**	NUTM2B-AS1	-0.411611475	5.583211088	12.23789293	0.0009109	0.0037997
**ENSG00000125630**	POLR1B	0.380854438	5.499744427	12.23529491	0.0009119	0.0038009
**ENSG00000181722**	ZBTB20	0.238459588	8.160063181	12.23518237	0.0009119	0.0038009
**ENSG00000114166**	KAT2B	0.272139437	7.56777825	12.21355158	0.0009207	0.0038358
**ENSG00000090989**	EXOC1	-0.289169656	6.069931545	12.20252683	0.0009252	0.0038530
**ENSG00000227191**		0.642715729	4.726601436	12.48282682	0.0009269	0.0038588
**ENSG00000240489**		0.677179887	3.650176073	12.19121296	0.0009298	0.0038692
**ENSG00000100075**	SLC25A1	0.681486571	3.696948996	12.18012532	0.0009344	0.0038866

**ENSG00000168970**	jmjd7-pla2g4b	-0.649959339	5.763173531	12.89724747	0.0009348	0.0038868
**ENSG00000169714**	CNBP	0.223964132	8.180659835	12.17521608	0.0009364	0.0038920
**ENSG00000171425**	ZNF581	-0.597190583	3.884965243	12.16313093	0.0009414	0.0039113
**ENSG00000134480**	CCNH	-0.286133086	6.597532879	12.15857193	0.0009433	0.0039176
**ENSG00000181396**	OGFOD3	-0.578593842	3.809266364	12.15668905	0.0009441	0.0039193
**ENSG00000154310**	TNIK	0.222498398	7.506447663	12.15288791	0.0009457	0.0039243
**ENSG00000267074**		0.436941337	5.27494636	12.15001212	0.0009469	0.0039277
**ENSG00000199631**	SNORD33	0.819685609	4.19731121	12.54696857	0.0009483	0.0039320
**ENSG00000086015**	mast2	0.54178848	3.423919861	12.14286128	0.0009499	0.0039371
**ENSG00000168393**	DTYMK	-0.537539487	3.773005398	12.1417115	0.0009504	0.0039372
**ENSG00000245748**	LOC100129931	0.391931823	5.321760686	12.14095979	0.0009507	0.0039372
**ENSG00000087338**	GMCL1	0.297957387	5.875830873	12.13934568	0.0009513	0.0039385
**ENSG00000079691**	CARMIL1	-0.540498914	4.764599805	12.14791812	0.0009544	0.0039495
**ENSG00000197776**	KLHDC1	-0.512729515	3.952486097	12.13102019	0.0009549	0.0039499
**ENSG00000067533**	RRP15	-0.343122437	5.56589192	12.12894437	0.0009557	0.0039519
**ENSG00000141524**	TMC6	-0.415002672	7.461094425	12.75781696	0.0009593	0.0039651
**ENSG00000104687**	GSR	0.335109676	5.708779725	12.11356515	0.0009623	0.0039758
**ENSG00000272367**		-0.443825417	4.561666881	12.11036287	0.0009636	0.0039798
**ENSG00000197771**	MCMBP	0.24882207	6.367523789	12.1072589	0.0009649	0.0039837
**ENSG00000108055**	SMC3	0.267389142	7.544457813	12.09943527	0.0009683	0.003996
**ENSG00000185513**	L3MBTL1	-0.677262483	3.872274852	12.09314004	0.0009710	0.0040055
**ENSG00000133706**	LARS	-0.221171718	7.281387351	12.09141104	0.0009717	0.0040070
**ENSG00000149970**	CNKSR2	-0.815010285	4.134131924	12.46564848	0.0009728	0.0040096
**ENSG00000168216**	LMBRD1	0.326423658	6.067213992	12.08713091	0.0009736	0.0040114
**ENSG00000177239**	MAN1B1	-0.459396597	5.777461215	12.36343405	0.0009753	0.0040172
**ENSG00000183283**	DAZAP2	0.202216021	8.316624963	12.06916627	0.0009813	0.0040403
**ENSG00000009694**	TENM1	-0.443512728	5.869625009	12.31107403	0.0009922	0.0040832
**ENSG00000166797**	FAM96A	0.533738538	4.3334404	12.04182465	0.0009933	0.0040862
**ENSG00000155229**	MMS19	-0.352241935	6.400294601	12.13792519	0.0009952	0.0040926
**ENSG00000170315**	UBB	0.288774915	7.559867112	12.13210734	0.0009967	0.0040971
**ENSG00000240344**	PPIL3	-0.430785816	4.775967091	12.03063109	0.0009982	0.0041004
**ENSG00000053900**	ANAPC4	-0.428949173	6.241934172	12.40839585	0.0009983	0.0041004
**ENSG00000120333**	MRPS14	-0.347171541	5.20626955	12.02017391	0.0010029	0.0041175
**ENSG00000082074**	FYB	-0.20091233	10.2556779	12.01782425	0.0010039	0.0041196
**ENSG00000263731**		0.621761724	3.423254151	12.01726369	0.0010042	0.0041196
**ENSG00000173473**	SMARCC1	0.249104575	7.386870426	12.00678948	0.0010088	0.0041371
**ENSG00000169062**	UPF3A	-0.371501847	5.764488585	11.99760721	0.0010129	0.0041524

**ENSG00000173933**	RBM4	-0.267476822	6.420021563	11.99066959	0.0010161	0.0041635
**ENSG00000239779**	WBP1	-0.59827511	4.361829934	11.9896673	0.0010165	0.0041637
**ENSG00000172469**	MANEA	0.397334519	5.394613975	11.98241795	0.0010198	0.0041748
**ENSG00000258659**	TRIM34	-0.538850269	3.84317189	11.981896	0.0010200	0.0041748
**ENSG00000089876**	DHX32	-0.545419632	3.974993518	11.97904313	0.0010213	0.0041785
**ENSG00000104907**	TRMT1	-0.666250596	4.587859887	12.24790742	0.0010241	0.0041885
**ENSG00000133250**	ZNF414	-0.598517757	3.527298424	11.9718526	0.0010246	0.0041885
**ENSG00000254721**		0.549429918	3.775832836	11.95356284	0.0010329	0.0042210
**ENSG00000234545**	FAM133B	-0.380433833	5.284708633	11.94217982	0.0010381	0.0042400
**ENSG00000105819**	PMPCB	-0.275851584	6.453711905	11.94167499	0.0010384	0.0042400
**ENSG00000168591**	TMUB2	-0.579675346	4.45204056	11.95064442	0.0010413	0.0042503
**ENSG00000274180**	natd1	0.50351337	3.617531775	11.92940347	0.0010440	0.0042598
**ENSG00000211689**	TARP	0.582090074	3.996061504	11.9221434	0.0010474	0.0042719
**ENSG00000268069**		-0.594678936	3.408222543	11.91431427	0.0010511	0.0042851
**ENSG00000185495**		-0.716073992	4.423186802	12.20179764	0.0010528	0.0042907
**ENSG00000205981**	DNAJC19	-0.422291676	4.860635538	11.88663242	0.0010641	0.0043347
**ENSG00000162836**	ACP6	-0.508506805	4.85438469	11.8724702	0.0010759	0.0043814
**ENSG00000271870**		-0.512167838	4.075569346	11.85323285	0.0010800	0.0043961
**ENSG00000125505**	MBOAT7	0.489453115	4.139223222	11.84850246	0.0010822	0.0044036
**ENSG00000172354**	GNB2	0.446273505	5.068333571	11.84554617	0.0010837	0.0044077
**ENSG00000100138**	SNU13	0.370375592	5.682957457	11.84399739	0.0010844	0.0044090
**ENSG00000165502**	RPL36AL	0.465757939	7.463508835	12.5412436	0.0010879	0.0044214
**ENSG00000183291**	SELENOF	0.330137749	6.637410781	11.91876292	0.0010883	0.0044214
**ENSG00000170776**	MIR7706	0.163904511	9.055526553	11.83094681	0.0010907	0.0044295
**ENSG00000136874**	STX17	-0.368785549	5.491462974	11.82314261	0.0010945	0.0044432
**ENSG00000213246**	SUPT4H1	-0.31928386	5.876396548	11.81578557	0.0010981	0.0044560
**ENSG00000151135**	TMEM263	-0.309131269	5.750994097	11.80494416	0.0011034	0.0044758
**ENSG00000152767**	FARP1	0.243861503	6.507206344	11.79529074	0.0011082	0.0044933
**ENSG00000112038**	OPRM1	-0.560365192	3.631897329	11.78631607	0.0011126	0.0045082
**ENSG00000118503**	TNFAIP3	-0.380993088	7.160507735	12.24314232	0.0011127	0.0045082
**ENSG00000145348**	TBCK	-0.320885457	6.287799422	11.78327255	0.0011141	0.0045122
**ENSG00000267520**		0.373865516	5.783555238	11.76668509	0.0011224	0.0045439
**ENSG00000070495**	JMJD6	0.473227837	4.670814734	11.76368861	0.0011238	0.0045482
**ENSG00000110321**	EIF4G2	0.181952556	9.507658841	11.76193219	0.0011247	0.0045500
**ENSG00000136731**	UGGT1	0.238047641	7.38515482	11.7508321	0.0011303	0.0045693
**ENSG00000283498**		0.517966403	4.202159869	11.75068986	0.0011304	0.0045693
**ENSG00000135821**	GLUL	-0.448680933	5.013862313	11.74621213	0.0011326	0.0045766

**ENSG00000142541**	RPL13A	0.259416478	10.71129364	11.73893007	0.0011363	0.0045897
**ENSG00000117899**	MESDC2	0.289547698	5.672604233	11.7368082	0.0011374	0.0045923
**ENSG00000178605**	GTPBP6	-0.492618178	4.999163204	11.76318061	0.0011418	0.0046074
**ENSG00000163655**	GMPS	0.274744663	6.557070893	11.72770612	0.0011420	0.0046074
**ENSG00000102409**	BEX4	-0.537548798	4.06972962	11.72314857	0.0011443	0.0046141
**ENSG00000157500**	APPL1	0.261223933	6.702647743	11.72270447	0.0011446	0.0046141
**ENSG00000198169**	ZNF251	-0.52212272	4.078074158	11.71949145	0.0011462	0.0046189
**ENSG00000164663**	USP49	-0.582796924	4.053014188	11.71225918	0.0011499	0.0046315
**ENSG00000277194**	Snord22	1.374411739	5.360051924	12.4574418	0.0011502	0.0046315
**ENSG00000273217**	RAPGEF6	-0.221998101	8.176075992	11.70877579	0.0011517	0.0046345
**ENSG00000134014**	ELP3	0.288363104	5.849430441	11.70849412	0.0011518	0.0046345
**ENSG00000151651**	ADAM8	0.40566759	5.478667245	11.70204161	0.0011551	0.0046460
**ENSG00000080815**	PSEN1	0.26847039	6.0257573	11.69738223	0.0011575	0.0046539
**ENSG00000135720**	DYNC1LI2	-0.26256846	6.413648755	11.69337317	0.0011596	0.0046604
**ENSG00000196072**	BLOC1S2	0.370378118	4.793507734	11.6908067	0.0011609	0.0046640
**ENSG00000211752**		0.580265397	3.461724054	11.68668464	0.0011631	0.0046708
**ENSG00000204604**	ZNF468	0.3762991	5.134521045	11.68082801	0.0011661	0.0046812
**ENSG00000155876**	RRAGA	0.404098	5.163263426	11.66976524	0.0011719	0.0047025
**ENSG00000266053**	NDUFV2-AS1	-0.578901866	3.765341155	11.66851737	0.0011725	0.0047029
**ENSG00000227124**	ZNF717	-0.587681738	3.89094363	11.66719651	0.0011732	0.0047029
**ENSG00000198805**	PNP	0.427575693	5.501686232	11.72762423	0.0011733	0.0047029
**ENSG00000050405**	LIMA1	0.412582655	5.222243713	11.66562234	0.0011741	0.0047038
**ENSG00000108423**	TUBD1	-0.521226635	4.751917366	11.67487494	0.0011744	0.0047038
**ENSG00000141568**	FOXK2	0.334327277	5.807371779	11.66099117	0.0011765	0.0047101
**ENSG00000118246**	FASTKD2	-0.335056238	5.51796687	11.65581361	0.0011792	0.0047179
**ENSG00000196950**	SLC39A10	0.235095939	6.796384897	11.65560024	0.0011793	0.0047179
**ENSG00000213928**	IRF9	-0.404232596	6.153084832	11.90586091	0.0011831	0.0047302
**ENSG00000275066**	SYNRG	0.18222527	8.364135547	11.64801745	0.0011833	0.0047302
**ENSG00000101608**	MYL12A	0.323058513	8.277993772	12.00644454	0.0011838	0.0047304
**ENSG00000136937**	NCBP1	-0.243472709	6.868808544	11.64541704	0.0011847	0.0047321
**ENSG00000029364**	SLC39A9	0.276656942	6.04819628	11.64219286	0.0011864	0.0047371
**ENSG00000139517**	LNX2	0.368112513	4.892794443	11.640329	0.0011874	0.0047392
**ENSG00000103994**	ZNF106	0.237557491	7.078538732	11.63508534	0.0011902	0.0047485
**ENSG00000076662**	ICAM3	0.29595339	6.18998394	11.63030337	0.0011927	0.0047569
**ENSG00000163932**	PRKCD	0.562430631	4.037003676	11.62697239	0.0011945	0.0047621
**ENSG00000210191**		-0.594892639	4.178423622	11.62555875	0.0011952	0.0047633
**ENSG00000171492**	LRRC8D	0.298620534	5.939613446	11.62339488	0.0011964	0.0047661

**ENSG00000103502**	CDIPT	0.430512834	4.907870776	11.62088776	0.0011977	0.0047696
**ENSG00000131263**	RLIM	0.240968126	7.145351984	11.60723945	0.0012050	0.0047969
**ENSG00000204305**	AGER	-0.63222963	3.935077921	11.59609923	0.0012111	0.0048190
**ENSG00000197157**	SND1	0.272552595	6.623498848	11.59454004	0.0012119	0.0048206
**ENSG00000197448**	GSTK1	-0.248814752	7.902321742	11.59297354	0.0012127	0.0048221
**ENSG00000106123**	EPHB6	-0.575792236	4.333934014	11.59014696	0.0012143	0.0048264
**ENSG00000264538**		-0.498634991	5.281064556	11.77567388	0.0012164	0.0048331
**ENSG00000230521**		0.482188383	4.721055796	11.58145799	0.0012190	0.0048414
**ENSG00000178035**	IMPDH2	-0.283119607	5.889753042	11.57067408	0.0012249	0.0048630
**ENSG00000187555**	USP7	0.176943328	8.049581908	11.56847471	0.0012261	0.0048659
**ENSG00000095787**	WAC	0.202898344	8.336116654	11.55757582	0.0012321	0.0048870
**ENSG00000014216**	CAPN1	0.343220323	5.499042253	11.55709932	0.0012323	0.0048870
**ENSG00000166788**	SAAL1	-0.519867702	3.921132664	11.55521343	0.0012334	0.0048887
**ENSG00000130177**	CDC16	-0.302198349	6.594419108	11.55462383	0.0012337	0.0048887
**ENSG00000008226**	DLEC1	-0.520882322	4.744507523	11.56735512	0.0012346	0.0048905
**ENSG00000270011**	ZNF559-ZNF177	-0.679300393	3.77826576	11.5503994	0.0012360	0.0048942
**ENSG00000111271**	ACAD10	-0.4578911	4.823288592	11.54505306	0.0012390	0.0049041
**ENSG00000130724**	CHMP2A	0.373636605	4.999807383	11.54306578	0.0012401	0.0049066
**ENSG00000166004**	CEP295	-0.299477865	6.391758451	11.53735547	0.0012433	0.0049173
**ENSG00000196323**	ZBTB44	0.181882029	7.715731497	11.53304099	0.0012457	0.0049249
**ENSG00000274266**	Snora73a	1.20291869	4.971099954	12.22894395	0.0012593	0.0049769
**ENSG00000105514**	RAB3D	0.61262542	3.484938701	11.49708477	0.0012659	0.0050010
**ENSG00000136810**	TXN	0.602651549	4.498642127	11.60095529	0.0012714	0.0050210
**ENSG00000215041**	neurl4	-0.527411268	4.341860453	11.48436794	0.0012731	0.0050258
**ENSG00000210195**		-0.417753405	5.181232741	11.4721593	0.0012801	0.0050514
**ENSG00000172785**	CBWD1	-0.569300097	4.020590536	11.46461302	0.0012844	0.0050666
**ENSG00000154743**	TSEN2	-0.522728099	4.133154867	11.46052693	0.0012868	0.0050739
**ENSG00000141582**	CBX4	0.367982245	5.162126524	11.45954213	0.0012873	0.0050743
**ENSG00000236830**	CBR3-AS1	-0.631007581	3.683649933	11.45732965	0.0012886	0.0050774
**ENSG00000105732**	ZNF574	0.479690891	4.062585089	11.44982349	0.001293	0.0050926
**ENSG00000165494**	PCF11	-0.191515472	8.114644862	11.4488499	0.0012935	0.0050929
**ENSG00000101247**	NDUFAF5	-0.578310388	4.230266698	11.44659464	0.0012948	0.0050961
**ENSG00000196867**	ZFP28	-0.52099063	3.987596561	11.43843035	0.0012996	0.0051129
**ENSG00000206573**	Thumpd3-as1	-0.303106303	6.154932427	11.42427515	0.0013078	0.0051435
**ENSG00000144746**	ARL6IP5	0.28326533	8.02210462	11.56746528	0.0013128	0.0051611
**ENSG00000129083**	COPB1	0.189022516	7.176312441	11.39634231	0.0013243	0.0052044
**ENSG00000119969**	HELLS	0.397512762	5.077312675	11.39307909	0.0013263	0.0052100

**ENSG00000129071**	MBD4	-0.296459139	5.949771811	11.38019474	0.0013339	0.0052382
**ENSG00000197114**	ZGPAT	-0.477185204	4.580919761	11.36959725	0.0013403	0.0052612
**ENSG00000283103**		-0.4682902	4.72503842	11.36836343	0.0013410	0.0052622
**ENSG00000172543**	CTSW	0.49702852	4.775206781	11.36121281	0.0013454	0.0052771
**ENSG00000177125**	zbtb34	0.447291207	4.735575396	11.3587754	0.0013468	0.0052809
**ENSG00000130935**	NOL11	-0.223047164	6.608907991	11.35419391	0.0013496	0.0052884
**ENSG00000156521**	TYSND1	-0.52853671	4.157386879	11.35393109	0.0013498	0.0052884
**ENSG00000182134**	TDRKH	0.445094132	4.442376625	11.3512107	0.0013514	0.0052917
**ENSG00000184271**	POU6F1	-0.48712761	4.823726536	11.35090298	0.0013516	0.0052917
**ENSG00000034693**	PEX3	-0.418891262	4.730970045	11.34586152	0.0013547	0.0053017
**ENSG00000066322**	MIR6734	0.508957485	4.568941179	11.34183134	0.0013571	0.0053093
**ENSG00000173757**	STAT5B	0.193067672	8.114544145	11.32749663	0.0013659	0.0053416
**ENSG00000229097**		-0.648049976	3.680556391	11.32540239	0.0013672	0.0053446
**ENSG00000275740**	RBM27	0.264082968	6.523062919	11.32398069	0.0013680	0.0053460
**ENSG00000111775**	Cox6a1	0.414675035	5.143954305	11.3202757	0.0013703	0.0053529
**ENSG00000268006**	PTOV1-AS1	-0.606787121	3.463995393	11.30892254	0.0013773	0.0053783
**ENSG00000123384**	LRP1	-0.525760439	4.153838656	11.30503301	0.0013797	0.0053856
**ENSG00000183741**	CBX6	0.301622615	5.625526591	11.30105698	0.0013822	0.0053933
**ENSG00000140157**	NIPA2	0.320305766	5.362655966	11.28886835	0.0013898	0.0054209
**ENSG00000156469**	MTERF3	-0.5436999	3.964045519	11.28442365	0.0013926	0.0054291
**ENSG00000117862**	TXNDC12	0.374087093	5.17993881	11.28383238	0.0013929	0.0054291
**ENSG00000100461**	RBM23	-0.262010916	6.357667648	11.28112749	0.0013946	0.0054337
**ENSG00000116514**	RNF19B	0.425228244	4.658673454	11.27924117	0.0013958	0.0054363
**ENSG00000114491**	UMPS	-0.390370236	5.006696579	11.27582945	0.001398	0.0054426
**ENSG00000152818**	UTRN	0.197555064	9.437276267	11.27027164	0.0014014	0.0054542
**ENSG00000205765**	C5orf51	0.258648593	6.139554561	11.26173148	0.0014068	0.0054731
**ENSG00000163960**	UBXN7	0.220974386	6.991178602	11.25535549	0.0014109	0.0054868
**ENSG00000258539**		0.318506319	5.48411385	11.2513699	0.0014134	0.0054929
**ENSG00000159593**	NAE1	-0.287663301	5.868286566	11.25120231	0.0014135	0.0054929
**ENSG00000160741**	CRTC2	-0.329531492	5.501550852	11.24488626	0.0014175	0.0055065
**ENSG00000271730**		-0.546686879	4.123054744	11.23850066	0.0014216	0.0055203
**ENSG00000132388**	UBE2G1	0.278686741	6.156227044	11.23521101	0.0014237	0.0055264
**ENSG00000165802**	MIR7114	-0.512955361	4.50410046	11.23240073	0.0014255	0.0055314
**ENSG00000066583**	ISOC1	0.536525146	3.9053699	11.23088698	0.0014265	0.0055331
**ENSG00000141384**	TAF4B	-0.52425332	4.535948738	11.22667233	0.0014292	0.0055415
**ENSG00000267349**		0.445668903	4.498070655	11.21783191	0.0014349	0.0055616
**ENSG00000263528**	IKBKE	-0.34745457	5.580327779	11.21180514	0.0014388	0.0055746

**ENSG00000185420**	SMYD3	-0.519624711	4.237815792	11.20944862	0.0014403	0.0055785
**ENSG00000104763**	ASAH1	-0.295605931	5.880860476	11.20783341	0.0014414	0.0055805
**ENSG00000107863**	ARHGAP21	0.344787102	5.611449967	11.19700278	0.0014484	0.0056057
**ENSG00000160877**	NACC1	0.435764649	4.292741883	11.19174597	0.0014518	0.0056169
**ENSG00000152102**	FAM168B	0.269521456	6.425178032	11.18836841	0.0014541	0.0056233
**ENSG00000164609**	SLU7	0.305462091	6.420937156	11.18216291	0.0014581	0.0056356
**ENSG00000154889**	MPPE1	-0.432735504	4.829756119	11.18189111	0.0014583	0.0056356
**ENSG00000141527**	CARD14	-0.763563728	3.6591184	11.28543204	0.0014609	0.0056435
**ENSG00000178977**	LINC00324	0.535244157	3.83467699	11.16755752	0.0014678	0.0056647
**ENSG00000140395**	WDR61	-0.451757762	4.683672923	11.16662633	0.0014684	0.0056647
**ENSG00000182400**	TRAPPC6B	-0.312750628	5.65725884	11.16645051	0.0014685	0.0056647
**ENSG00000124243**	BCAS4	-0.441957099	4.605107757	11.16633725	0.0014686	0.0056647
**ENSG00000165591**	FAAH2	-0.542756992	3.879418214	11.16365014	0.0014703	0.0056695
**ENSG00000022976**	ZNF839	-0.481083599	4.220515649	11.16207018	0.0014714	0.0056715
**ENSG00000172939**	OXSR1	0.277064308	5.918183829	11.15283516	0.0014775	0.0056930
**ENSG00000135404**	CD63	0.666592708	3.97461553	11.20553472	0.0014785	0.0056946
**ENSG00000185947**	ZNF267	0.320719987	5.86782394	11.14962051	0.0014797	0.0056952
**ENSG00000157014**	TATDN2	0.222588395	6.586848714	11.14952658	0.0014797	0.0056952
**ENSG00000147576**	ADHFE1	-0.467205486	4.68249299	11.14312705	0.0014840	0.0057096
**ENSG00000277072**		-0.520510776	4.111463918	11.14004985	0.0014861	0.0057154
**ENSG00000168411**	RFWD3	0.314341219	5.423468641	11.1357066	0.0014890	0.0057245
**ENSG00000242861**		-0.556736713	4.221431947	11.13260383	0.0014911	0.0057304
**ENSG00000270589**		-0.548613135	4.096885866	11.12966283	0.0014930	0.0057359
**ENSG00000092094**	OSGEP	-0.578373031	5.076250794	11.43150835	0.0015032	0.0057727
**ENSG00000197375**	slc22a5	-0.44971992	5.261590102	11.1617538	0.0015066	0.0057837
**ENSG00000179010**	MRFAP1	0.259152916	6.396747247	11.09867632	0.0015140	0.0058102
**ENSG00000109466**	KLHL2	-0.346071208	5.117559423	11.09770069	0.0015147	0.0058106
**ENSG00000102144**	PGK 1.00	0.223703502	7.274556199	11.09322053	0.0015178	0.0058202
**ENSG00000120675**	DNAJC15	-0.391747922	5.506424878	11.08604608	0.0015227	0.0058370
**ENSG00000135315**	CEP162	-0.42476551	4.712947583	11.08330793	0.0015246	0.0058420
**ENSG00000089327**	FXYD5	0.27321965	8.374567885	11.20861693	0.0015255	0.0058435
**ENSG00000188687**	slc4a5	-0.462953206	4.716865105	11.08021535	0.0015267	0.0058459
**ENSG00000273025**	CELF6	-0.642082118	3.574747305	11.07593393	0.0015297	0.0058551
**ENSG00000248333**	CDK11B	-0.318363224	5.567358977	11.07170856	0.0015326	0.0058641
**ENSG00000259865**		-0.483009458	4.657102119	11.06406647	0.0015379	0.0058798
**ENSG00000155304**	HSPA13	0.402588179	5.451304025	11.06364218	0.0015382	0.0058798
**ENSG00000149929**	hirip3	-0.435556976	4.531591136	11.06335438	0.0015384	0.0058798

**ENSG00000150712**	MTMR12	0.300448756	5.580957735	11.0465077	0.0015501	0.0059225
**ENSG00000267469**		0.259735895	7.164657865	11.04548815	0.0015508	0.0059231
**ENSG00000112685**	exoc2	0.263926803	6.702277904	11.04340456	0.0015523	0.0059265
**ENSG00000145191**	EIF2B5	-0.308017952	5.643580603	11.04072174	0.0015542	0.0059313
**ENSG00000185664**	PMEL	-0.511213468	4.929839572	11.13888213	0.0015555	0.0059313
**ENSG00000148339**	SLC25A25	0.377859996	4.719526055	11.03860734	0.0015557	0.0059313
**ENSG00000160570**	DEDD2	0.410485257	4.806849599	11.03837706	0.0015558	0.0059313
**ENSG00000237568**		0.487290801	5.068267021	11.12298061	0.0015574	0.0059350
**ENSG00000267009**		0.296389551	5.656711507	11.03339999	0.0015593	0.0059404
**ENSG00000126267**	Cox6b1	0.38865468	5.223375116	11.03218205	0.0015602	0.0059415
**ENSG00000248734**		-0.394382059	4.866400245	11.02863138	0.0015627	0.0059488
**ENSG00000171843**	MLLT3	-0.288209004	6.795973859	11.02518893	0.0015651	0.0059559
**ENSG00000284707**		-0.34241028	5.186685323	11.024336	0.0015657	0.0059561
**ENSG00000133256**	PDE6B	-0.635662542	3.537261677	11.01755453	0.0015705	0.0059722
**ENSG00000116199**	FAM20B	0.33920588	5.461251257	11.00627117	0.0015786	0.0060005
**ENSG00000115145**	STAM2	0.326905117	5.694436403	11.00180118	0.0015818	0.0060105
**ENSG00000225528**		-0.629549816	3.647929205	10.9956172	0.0015862	0.0060251
**ENSG00000279530**		-0.531228274	4.245446447	10.99181218	0.0015889	0.0060319
**ENSG00000113119**	TMCO6	-0.477895328	4.344419791	10.99150322	0.0015891	0.0060319
**ENSG00000215305**	VPS16	-0.403448324	4.809373795	10.97608039	0.0016003	0.0060719
**ENSG00000120254**	MTHFD1L	0.598058618	3.671324717	10.9746181	0.0016013	0.0060738
**ENSG00000115211**	EIF2B4	-0.423113982	4.916556508	10.97372707	0.0016020	0.0060740
**ENSG00000196975**	ANXA4	0.568882407	3.988041231	10.970221	0.0016045	0.0060814
**ENSG00000243708**	PLA2G4B	-0.70529783	4.998391565	11.48712217	0.0016059	0.0060847
**ENSG00000161800**	RACGAP1	0.609657747	3.576280149	10.96620118	0.0016074	0.0060881
**ENSG00000085998**	POMGNT1	-0.497445299	4.229175688	10.96431679	0.0016088	0.0060911
**ENSG00000108523**	RNF167	0.294666391	5.845352348	10.9626664	0.0016100	0.0060934
**ENSG00000108424**	KPNB1	0.15913007	7.959824776	10.95187758	0.0016179	0.0061210
**ENSG00000005884**	ITGA3	0.685976049	3.430640745	10.9452488	0.0016227	0.0061372
**ENSG00000119638**	NEK9	-0.229390742	6.64977287	10.93831772	0.0016278	0.0061542
**ENSG00000248483**	POU5F2	-0.620641862	4.259174031	10.98029445	0.0016362	0.0061837
**ENSG00000165572**	KBTBD6	0.53806247	4.564355146	10.93836388	0.0016428	0.0062064
**ENSG00000182923**	CEP63	-0.416448374	5.370423202	10.91199541	0.0016473	0.0062213
**ENSG00000197586**	ENTPD6	-0.399408868	4.738974369	10.90653243	0.0016514	0.0062344
**ENSG00000179271**	GADD45GIP1	0.439965762	4.720185827	10.90102539	0.0016556	0.0062477
**ENSG00000213190**	MLLT11	-0.38174512	5.363105464	10.89810441	0.0016577	0.0062538
**ENSG00000137275**	RIPK1	0.265074451	6.154340959	10.89653105	0.0016589	0.0062560

**ENSG00000184220**	CMSS1	-0.570723052	4.046591208	10.87147482	0.0016779	0.0063251
**ENSG00000181830**	SLC35C1	0.461755007	4.12280145	10.8572308	0.0016887	0.0063638
**ENSG00000198408**	MGEA5	-0.243844108	8.88147995	10.85351711	0.0016916	0.0063722
**ENSG00000159840**	ZYX	0.292558719	6.487114282	10.84185431	0.0017006	0.0064035
**ENSG00000115604**	IL18R1	0.52395566	4.869229955	10.95547893	0.0017011	0.0064035
**ENSG00000152495**	CAMK4	-0.264546538	8.805298195	10.9284706	0.0017082	0.0064279
**ENSG00000213588**	ZBTB9	0.49228995	3.561608608	10.82958496	0.0017100	0.0064325
**ENSG00000235703**	LINC00894	-0.673327175	4.252956465	10.98331033	0.0017121	0.0064364
**ENSG00000188321**	ZNF559	-0.347985976	5.228876899	10.82665497	0.0017123	0.0064364
**ENSG00000154978**	VOPP1	0.268726801	5.706963652	10.81681286	0.0017200	0.0064629
**ENSG00000162894**	FCMR	-0.244043459	7.80997734	10.8037998	0.0017302	0.0064988
**ENSG00000102879**	coro1a	0.268807886	8.028725436	10.90926266	0.0017340	0.0065111
**ENSG00000135766**	EGLN1	0.29186397	5.96235688	10.79479991	0.0017373	0.0065208
**ENSG00000135709**	KIAA0513	0.507716715	4.313036573	10.78995934	0.0017411	0.0065328
**ENSG00000143891**	GALM	0.479928565	4.88066863	10.79012567	0.0017473	0.0065538
**ENSG00000144741**	SLC25A26	0.291433653	5.531754252	10.77840537	0.0017502	0.0065624
**ENSG00000114738**	MAPKAPK3	0.395656202	5.373001051	10.77546015	0.0017526	0.0065688
**ENSG00000257027**		-0.390515585	6.476517485	11.08198385	0.0017535	0.0065699
**ENSG00000010818**	HIVEP2	0.201587298	8.570458042	10.76903134	0.0017577	0.0065833
**ENSG00000241127**	YAE1D1	-0.568931194	3.381146886	10.76668226	0.0017596	0.0065880
**ENSG00000175895**	PLEKHF2	0.427461287	5.060282715	10.76113144	0.0017640	0.0066023
**ENSG00000229180**		-0.399585	5.468043375	10.75897	0.0017658	0.0066064
**ENSG00000198189**	HSD17B11	0.264139649	6.147299947	10.75685465	0.0017674	0.0066104
**ENSG00000213865**	C8orf44	-0.519040862	3.892539086	10.73289543	0.0017868	0.0066801
**ENSG00000132768**	DPH2	-0.565243349	3.618136234	10.73198053	0.0017875	0.0066801
**ENSG00000243406**	MRPS31P5	-0.450099685	4.539821795	10.73139308	0.0017880	0.0066801
**ENSG00000149716**	ORAOV1	-0.512066837	4.628028697	10.72855811	0.0017903	0.0066864
**ENSG00000137841**	PLCB2	-0.414798867	6.889457657	11.20327021	0.0017943	0.0066987
**ENSG00000165434**	PGM2L1	0.36536753	5.588103388	10.71998664	0.0017973	0.0067077
**ENSG00000155329**	ZCCHC10	0.483921226	4.365734307	10.71540357	0.0018011	0.0067193
**ENSG00000226950**	DANCR	-0.407399591	4.603832757	10.71418128	0.0018021	0.0067206
**ENSG00000110429**	FBXO3	-0.265620447	6.93321666	10.70479344	0.0018098	0.0067469
**ENSG00000161533**	ACOX1	0.337015408	5.583079659	10.70180994	0.0018122	0.0067537
**ENSG00000149231**	CCDC82	-0.270852374	6.335151043	10.70049945	0.0018133	0.0067553
**ENSG00000188186**	LAMTOR4	-0.454506345	4.632077995	10.69899991	0.0018145	0.0067575
**ENSG00000128340**	RAC2	0.281718376	8.425429989	10.86756865	0.0018268	0.0068008
**ENSG00000153066**	TXNDC11	0.320714121	5.514015007	10.67894985	0.0018312	0.0068146

**ENSG00000266094**	RASSF5	0.199449178	8.101001101	10.67135562	0.0018375	0.0068358
**ENSG00000100376**	FAM118A	-0.536655831	5.716490151	11.10621199	0.0018397	0.0068414
**ENSG00000119720**	NRDE2	-0.269522201	5.841200919	10.66708228	0.0018411	0.0068442
**ENSG00000213281**	NRAS	0.290821823	6.066339719	10.66574397	0.0018422	0.0068459
**ENSG00000276291**		-0.393194601	4.856598319	10.65384858	0.0018522	0.0068807
**ENSG00000123595**	RAB9A	0.520810229	3.806603818	10.64883406	0.0018565	0.0068917
**ENSG00000218510**	LINC00339	-0.526429296	3.869925719	10.64877155	0.0018565	0.0068917
**ENSG00000258813**		-0.548963983	4.026275597	10.64449953	0.0018601	0.0069021
**ENSG00000166734**	CASC4	0.247149522	6.916240985	10.64389399	0.0018606	0.0069021
**ENSG00000167851**	CD300A	0.615669904	3.817339171	10.63934808	0.0018645	0.0069140
**ENSG00000198589**	LRBA	0.195464274	8.365965138	10.63060501	0.0018719	0.0069391
**ENSG00000111215**	PRR4	-0.489921932	4.209093585	10.62279673	0.0018786	0.0069613
**ENSG00000122882**	ecd	-0.284360304	5.707455338	10.62142687	0.0018798	0.0069632
**ENSG00000130520**	LSM 4.00	-0.405923822	4.754854678	10.6184507	0.0018823	0.0069699
**ENSG00000116044**	NFE2L2	0.207745638	6.929206142	10.61776866	0.0018829	0.0069699
**ENSG00000267645**	POLR2J2	-0.464253083	4.52911179	10.61697906	0.0018836	0.0069699
**ENSG00000257594**	GALNT4	0.397991167	4.759358232	10.61407512	0.0018861	0.0069767
**ENSG00000177971**	IMP3	0.429932142	4.814473865	10.60957296	0.0018900	0.0069885
**ENSG00000171163**	ZNF692	-0.696753639	4.791696861	11.04708249	0.0018910	0.0069899
**ENSG00000140367**	UBE2Q2	0.296150107	6.385135326	10.60720973	0.0018920	0.0069911
**ENSG00000083290**	ULK2	-0.410355548	4.891975223	10.6059365	0.0018931	0.0069927
**ENSG00000104885**	DOT1L	-0.381645428	5.111226293	10.59972986	0.0018985	0.0070101
**ENSG00000131844**	MCCC2	-0.307966834	5.55837589	10.59201443	0.0019051	0.0070323
**ENSG00000166313**	APBB1	-0.375304447	5.45888293	10.58485554	0.0019114	0.0070528
**ENSG00000114779**	ABHD14B	0.378711752	5.15868013	10.57934396	0.0019162	0.0070680
**ENSG00000149582**	TMEM25	-0.585301782	3.481988896	10.57630055	0.0019188	0.0070753
**ENSG00000261771**	DYX1C1-CCPG1	0.293681129	5.679948289	10.56605272	0.0019278	0.0071060
**ENSG00000198026**	ZNF335	-0.328941216	6.215198105	10.59643195	0.0019343	0.0071274
**ENSG00000198833**	UBE2J1	0.269310212	5.906099479	10.55272516	0.0019396	0.0071443
**ENSG00000213445**	SIPA1	0.330177816	6.375921469	10.62788127	0.0019426	0.0071530
**ENSG00000157800**	SLC37A3	-0.342148152	4.814704993	10.54045482	0.0019505	0.0071793
**ENSG00000124608**	AARS2	-0.468218032	4.578966925	10.52077656	0.0019681	0.0072416
**ENSG00000135677**	GNS	0.313752349	5.915993543	10.51492664	0.0019733	0.0072584
**ENSG00000158156**	XKR8	0.592196002	3.591862961	10.50959963	0.0019781	0.0072735
**ENSG00000188234**	AGAP4	-0.744042604	3.398284319	10.55219098	0.0019809	0.0072811
**ENSG00000251992**		-0.609756963	6.926005735	11.10806302	0.0019824	0.0072835
**ENSG00000107077**	KDM4C	-0.23559764	7.073511788	10.50428478	0.0019829	0.0072835

**ENSG00000120533**	ENY2	-0.316337656	5.399033196	10.49906042	0.0019877	0.0072983
**ENSG00000188878**	FBF1	-0.63710209	3.711015379	10.49599767	0.0019905	0.0073060
**ENSG00000167106**	FAM102A	-0.215984041	7.847097272	10.49063037	0.0019954	0.0073213
**ENSG00000166508**	MCM7	-0.431453447	5.417684481	10.56690654	0.0019983	0.0073295
**ENSG00000130775**	THEMIS2	0.306836078	5.270148018	10.48452041	0.0020009	0.0073367
**ENSG00000113916**	BCL6	0.655819888	3.61018505	10.48322995	0.0020021	0.0073384
**ENSG00000155189**	AGPAT5	0.380775691	4.898734473	10.48048422	0.0020046	0.0073450
**ENSG00000189195**	KIAA1107	-0.554668837	3.906878625	10.46937102	0.0020148	0.0073799
**ENSG00000218739**	CEBPZOS	-0.272359439	6.275185948	10.46745769	0.0020166	0.0073837
**ENSG00000124357**	NAGK	-0.383872598	5.460233691	10.46332319	0.0020204	0.0073944
**ENSG00000174353**	TRIM74	-0.504613496	4.466238289	10.4627695	0.0020209	0.0073944
**ENSG00000012232**	EXTL3	0.437916368	4.323821632	10.45101815	0.0020318	0.0074316
**ENSG00000213742**	ZNF337-AS1	-0.351573271	5.159578744	10.44585904	0.0020366	0.0074444
**ENSG00000013725**	CD6	-0.214116022	7.583169671	10.44509588	0.0020373	0.0074444
**ENSG00000140543**	DET1	-0.544069499	4.219787415	10.44495341	0.0020375	0.0074444
**ENSG00000117000**	RLF	0.284926757	6.195171046	10.43872724	0.0020433	0.0074631
**ENSG00000116497**	S100PBP	0.296898561	6.229664286	10.43631104	0.0020455	0.0074687
**ENSG00000108433**	GOSR2	-0.27475329	6.407592335	10.43114273	0.0020504	0.0074838
**ENSG00000133624**	ZNF767P	-0.501784267	4.996447559	10.55679161	0.0020517	0.0074862
**ENSG00000180096**	37135	-0.342253204	6.746968348	10.6432266	0.0020537	0.0074907
**ENSG00000186318**	BACE1	0.373438858	5.413145561	10.42242677	0.0020586	0.0075058
**ENSG00000067900**	ROCK1	0.241904699	8.296053195	10.42827208	0.0020599	0.0075081
**ENSG00000235944**		-0.462222798	4.526999166	10.41416922	0.0020663	0.0075290
**ENSG00000270157**		-0.671036607	3.897423932	10.46841867	0.0020686	0.0075345
**ENSG00000143569**	UBAP2L	-0.190199549	7.586303885	10.40812233	0.0020721	0.0075446
**ENSG00000157212**	PAXIP1	-0.303722553	5.561055226	10.39460802	0.0020849	0.0075887
**ENSG00000004897**	CDC27	0.213207442	6.614759186	10.3897914	0.0020895	0.0076029
**ENSG00000151116**	UEVLD	0.499966379	4.096495665	10.37585514	0.0021029	0.0076489
**ENSG00000264112**		-0.578236355	5.191651715	10.74401606	0.0021044	0.0076515
**ENSG00000138796**	HADH	-0.356462902	4.850400681	10.36457636	0.0021138	0.0076831
**ENSG00000099219**	ERMP1	0.280820879	5.847005565	10.36369416	0.0021147	0.0076836
**ENSG00000113194**	FAF2	0.280456953	5.741496874	10.36268311	0.0021156	0.0076845
**ENSG00000167476**	LOC105372240	0.404488578	5.451114012	10.39489845	0.0021244	0.0077135
**ENSG00000255987**		0.434615066	4.609233982	10.34746887	0.0021304	0.0077328
**ENSG00000270012**		-0.656075826	3.822971195	10.33993905	0.0021419	0.0077698
**ENSG00000102871**	TRADD	0.388449516	4.962022939	10.33554148	0.0021421	0.0077698
**ENSG00000124831**	LRRFIP1	0.198989903	8.613206768	10.33391458	0.0021437	0.0077729

**ENSG00000055955**	ITIH4	-0.65096431	4.360331894	10.50519026	0.0021447	0.0077737
**ENSG00000213903**	LTB4R	-0.568337277	4.527306578	10.41044461	0.0021521	0.0077942
**ENSG00000185246**	PRPF39	-0.392344506	6.282291269	10.60237348	0.0021524	0.0077942
**ENSG00000243107**		-0.637753663	3.782313468	10.3249351	0.0021525	0.0077942
**ENSG00000139508**	SLC46A3	-0.348072312	5.259734411	10.32368143	0.0021538	0.0077960
**ENSG00000124733**	MEA1	0.510659413	4.234091828	10.32107949	0.0021564	0.0078026
**ENSG00000221817**	PPP3CB-AS1	-0.387369377	5.04248682	10.31620558	0.0021612	0.0078162
**ENSG00000253980**		-0.811878348	3.809368659	10.55780451	0.0021616	0.0078162
**ENSG00000229127**		-0.618475948	3.981798131	10.30498299	0.0021723	0.0078522
**ENSG00000108788**	MLX	0.357893794	5.070504959	10.29770377	0.0021796	0.0078757
**ENSG00000110934**	BIN2	-0.173311448	8.038961331	10.28559659	0.0021917	0.0079136
**ENSG00000143951**	WDPCP	-0.587920597	4.027444756	10.28556562	0.0021918	0.0079136
**ENSG00000158863**	FAM160B2	-0.544126328	5.205591475	10.57289705	0.0021923	0.0079136
**ENSG00000176407**	KCMF1	0.285923564	5.746653531	10.27250136	0.0022049	0.0079564
**ENSG00000103264**	FBXO31	0.367590605	4.679471825	10.26940699	0.0022081	0.0079649
**ENSG00000145901**	TNIP1	0.217134014	6.653311123	10.26784508	0.0022097	0.0079679
**ENSG00000177432**	NAP1L5	-0.572818447	3.365859942	10.26091561	0.0022167	0.0079905
**ENSG00000117632**	MIR3917	-0.421878326	4.473662819	10.25352803	0.0022242	0.0080149
**ENSG00000197008**	ZNF138	-0.392105959	5.119270466	10.24601395	0.0022319	0.0080397
**ENSG00000225470**	Jpx	-0.362417621	6.27749533	10.41483532	0.0022327	0.0080397
**ENSG00000263482**		-0.446202186	4.312094643	10.24137177	0.0022367	0.0080514
**ENSG00000265794**		-0.23048647	6.454557659	10.23982656	0.0022383	0.0080527
**ENSG00000121716**	MIR6840	-0.641442104	5.651781844	10.77800493	0.0022386	0.0080527
**ENSG00000196670**	ZFP62	-0.344397926	5.38786955	10.23459635	0.0022437	0.0080666
**ENSG00000114107**	CEP70	-0.531069756	4.219937032	10.23428301	0.0022440	0.0080666
**ENSG00000074621**	SLC24A1	-0.377740033	5.216366369	10.23298055	0.0022453	0.0080686
**ENSG00000174943**	kctd13	-0.589453217	4.316371118	10.27816396	0.0022523	0.0080892
**ENSG00000249115**	HAUS5	-0.515405505	4.240487001	10.22593785	0.0022526	0.0080892
**ENSG00000170946**	DNAJC24	-0.363756577	4.986643174	10.22109603	0.0022576	0.0081045
**ENSG00000070476**	ZXDC	-0.282021447	5.846515141	10.19732932	0.0022824	0.0081906
**ENSG00000185963**	BICD2	0.264974953	5.691103919	10.19595186	0.0022839	0.0081930
**ENSG00000142634**	EFHD2	0.467895845	4.789922471	10.19345746	0.0022865	0.0081996
**ENSG00000144566**	RAB5A	0.26227219	6.003116843	10.18995376	0.0022902	0.0082100
**ENSG00000163629**	PTPN13	0.533030847	5.3788398	10.52207287	0.0022929	0.0082172
**ENSG00000171817**	ZNF540	-0.497052151	4.392670433	10.18403336	0.0022964	0.0082268
**ENSG00000180957**	PITPNB	0.231133431	6.243167779	10.17893945	0.0023018	0.0082432
**ENSG00000265401**		0.300101208	7.222547561	10.33222014	0.0023150	0.0082875

**ENSG00000184988**	TMEM106A	-0.532417374	3.844542321	10.16516069	0.0023164	0.0082899
**ENSG00000170542**	SERPINB9	0.223489771	7.326883783	10.1574013	0.0023247	0.0083144
**ENSG00000084093**	REST	0.214561076	7.811968339	10.15694125	0.0023252	0.0083144
**ENSG00000132763**	MMACHC	0.428110805	4.916488308	10.15651626	0.0023257	0.0083144
**ENSG00000176422**	spryd4	-0.398023741	4.586704242	10.1473805	0.0023355	0.0083466
**ENSG00000109685**	whsc1	0.303591198	6.306599269	10.14572019	0.0023372	0.0083501
**ENSG00000162302**	RPS6KA4	0.637695868	3.307181194	10.143261	0.0023399	0.0083567
**ENSG00000136891**	TEX10	-0.289400649	6.128889427	10.1390654	0.0023444	0.0083700
**ENSG00000173418**	NAA20	-0.372879797	4.573801762	10.13762272	0.0023460	0.0083727
**ENSG00000177225**	PDDC1	-0.465014937	4.442864421	10.13602144	0.0023477	0.0083760
**ENSG00000100911**	MIR7703	-0.346006129	5.525188421	10.13529334	0.0023485	0.0083760
**ENSG00000267368**	UPK3BL	-0.470500104	4.36569418	10.13347376	0.0023504	0.0083802
**ENSG00000181826**	RELL1	0.470809107	4.117488401	10.12604629	0.0023585	0.0084060
**ENSG00000088038**	CNOT3	-0.29971633	5.992188885	10.11904803	0.0023661	0.0084275
**ENSG00000274020**	LINC01138	-0.368774255	4.990424096	10.11901553	0.0023661	0.0084275
**ENSG00000125821**	DTD1	0.400639324	4.489305487	10.10421678	0.0023823	0.0084823
**ENSG00000123144**	c19orf43	0.325858663	5.843166244	10.10312508	0.0023835	0.0084836
**ENSG00000119965**	c10orf88	-0.436381958	3.96897923	10.08961707	0.0023984	0.0085337
**ENSG00000204564**	C6orf136	-0.488919838	3.868579014	10.08296822	0.0024058	0.0085570
**ENSG00000063046**	EIF4B	0.205837041	9.520732282	10.07779247	0.0024115	0.0085745
**ENSG00000213762**	ZNF134	0.371336376	5.071792774	10.07216323	0.0024178	0.0085938
**ENSG00000172803**	SNX32	0.322572598	6.328845009	10.1323213	0.0024222	0.0086068
**ENSG00000138002**	IFT172	-0.561030451	4.136963804	10.06442865	0.0024264	0.0086187
**ENSG00000111581**	NUP107	-0.282503269	6.013898054	10.06008306	0.0024313	0.0086330
**ENSG00000160058**	BSDC1	-0.288468152	5.689978144	10.05560945	0.0024363	0.0086479
**ENSG00000162032**	SPSB3	-0.564877672	4.972653857	10.30925765	0.0024380	0.0086510
**ENSG00000284431**		-0.268249034	6.311194618	10.0523566	0.0024400	0.0086550
**ENSG00000106682**	EIF4H	0.248283554	6.463901104	10.03825724	0.0024559	0.0087085
**ENSG00000197381**	ADARB1	0.24405285	5.974062424	10.03668278	0.0024577	0.0087119
**ENSG00000154265**	ABCA5	-0.282608511	6.481386265	10.03508717	0.0024595	0.0087154
**ENSG00000116288**	PARK7	0.326259351	5.588465711	10.02502476	0.0024709	0.0087529
**ENSG00000183049**	CAMK1D	0.256697879	6.202032041	10.02410735	0.0024720	0.0087537
**ENSG00000204256**	BRD2	0.172858092	8.124693841	10.0209163	0.0024756	0.0087636
**ENSG00000109323**	MANBA	-0.371074108	4.799123077	10.02018082	0.0024764	0.0087636
**ENSG00000122481**	RWDD3	-0.43965338	4.006361864	10.01658097	0.0024806	0.0087752
**ENSG00000260729**		-0.463218856	4.101136893	9.994231427	0.0025063	0.0088632
**ENSG00000197563**	PIGN	-0.328465342	5.519069557	9.984672653	0.0025174	0.0088967

**ENSG00000172345**	STARD5	-0.23186524	6.727349208	9.984060421	0.0025181	0.0088967
**ENSG00000142765**	SYTL1	-0.497096439	5.673068524	10.33738416	0.0025183	0.0088967
**ENSG00000284360**		-0.511262469	3.794635893	9.979079426	0.0025239	0.0089134
**ENSG00000104626**	ERI1	0.340964171	4.945470958	9.977201952	0.0025261	0.0089159
**ENSG00000170903**	MSANTD4	0.367118418	5.001825707	9.976997503	0.0025263	0.0089159
**ENSG00000215837**		-0.47399383	4.133002943	9.973770148	0.0025301	0.0089261
**ENSG00000168283**	BMI1	0.225587234	6.738328353	9.973070358	0.0025309	0.0089261
**ENSG00000136628**	EPRS	0.223960505	7.1912145	9.969194061	0.0025354	0.0089391
**ENSG00000112658**	SRF	0.417826679	4.683473901	9.967260035	0.0025377	0.0089441
**ENSG00000215788**	TNFRSF25	-0.539913489	6.518308659	10.490994	0.0025400	0.0089492
**ENSG00000281039**		-0.633100456	3.408811814	9.959599807	0.0025467	0.0089697
**ENSG00000266402**	SNHG25	-0.681073244	4.06988486	10.08986402	0.0025565	0.0090012
**ENSG00000203896**	lime1	0.483122981	5.196760626	10.10916715	0.0025596	0.0090091
**ENSG00000238105**	GOLGA2P5	-0.474351655	5.238870749	10.10669158	0.0025608	0.0090105
**ENSG00000187240**	DYNC2H1	-0.546340172	4.225336191	9.940640499	0.0025691	0.0090365
**ENSG00000155850**	SLC26A2	0.297531779	5.508624504	9.931835719	0.0025796	0.0090703
**ENSG00000085117**	CD82	0.4233335	4.660348565	9.929828348	0.0025820	0.0090757
**ENSG00000110218**	PANX1	0.527281102	3.746077485	9.927514808	0.0025847	0.0090823
**ENSG00000046651**	OFD1	-0.246529015	7.043395784	9.925911605	0.0025867	0.0090860
**ENSG00000128534**	LSM 8.00	-0.266794647	5.959901492	9.921926481	0.0025914	0.0090997
**ENSG00000139990**	DCAF5	-0.197635619	6.984942223	9.917423483	0.0025968	0.0091156
**ENSG00000262526**		0.356544193	6.113780899	10.04194429	0.0026008	0.0091263
**ENSG00000140326**	CDAN1	-0.435408036	4.241084628	9.910494651	0.0026052	0.0091387
**ENSG00000277778**	PGM5P2	-0.503779408	3.789848627	9.906725894	0.0026097	0.0091516
**ENSG00000105643**	ARRDC2	-0.325202281	5.29219423	9.904686982	0.0026122	0.0091572
**ENSG00000114503**	NCBP2	-0.252032243	6.112231648	9.895013759	0.0026239	0.0091952
**ENSG00000255443**		0.328229327	5.519262748	9.892362612	0.0026271	0.0092034
**ENSG00000088876**	ZNF343	-0.558772514	3.662604612	9.880623468	0.0026414	0.0092504
**ENSG00000235785**		-0.605300538	3.819853689	9.870354784	0.0026540	0.0092914
**ENSG00000115159**	GPD2	0.347964241	5.051502725	9.867478243	0.0026575	0.0093006
**ENSG00000119684**	MLH3	-0.289580968	5.8494012	9.865461004	0.0026600	0.0093062
**ENSG00000151413**	NUBPL	-0.572975318	3.424402914	9.857964054	0.0026693	0.0093336
**ENSG00000053702**	NRIP2	-0.53063097	3.955590106	9.8576629	0.0026697	0.0093336
**ENSG00000163806**	spdya	0.327122401	6.296439839	9.937559275	0.0026709	0.0093350
**ENSG00000101266**	CSNK2A1	0.229291865	6.226768743	9.853499308	0.0026748	0.0093454
**ENSG00000163877**	SNIP1	0.329471127	5.125609135	9.850804583	0.0026781	0.0093513
**ENSG00000048828**	FAM120A	0.167105517	7.763297773	9.850699461	0.0026783	0.0093513

**ENSG00000105926**	MPP6	-0.381439503	5.458930464	9.846148368	0.0026841	0.0093685
**ENSG00000133398**	MED10	-0.368321687	4.816676792	9.842748241	0.0026882	0.0093795
**ENSG00000136824**	SMC2	0.332426324	5.293864138	9.835392365	0.0026973	0.0094084
**ENSG00000158711**	ELK4	0.192802459	8.950072445	9.830122577	0.0027039	0.0094252
**ENSG00000172663**	TMEM134	-0.445985345	4.569726879	9.830113071	0.0027039	0.0094252
**ENSG00000224086**		-0.381719724	5.541784373	9.857209596	0.0027063	0.0094303
**ENSG00000279059**		-0.539538645	3.770775011	9.826437185	0.0027085	0.0094349
**ENSG00000272030**		-0.482568065	3.855353718	9.823336369	0.0027124	0.0094454
**ENSG00000176438**	syne3	0.214398981	7.320126821	9.816792443	0.0027207	0.0094709
**ENSG00000200087**		1.211416467	4.98583261	10.33730783	0.0027359	0.0095208
**ENSG00000177042**	TMEM80	-0.497217344	3.678597643	9.797400729	0.0027453	0.0095501
**ENSG00000144524**	COPS7B	-0.360861056	5.158090883	9.795863286	0.0027472	0.0095537
**ENSG00000240342**		0.455564621	5.652988902	10.0380743	0.0027616	0.0096006
**ENSG00000143319**	ISG20L2	0.321785232	5.11190021	9.765927046	0.0027856	0.0096808
**ENSG00000105339**	DENND3	0.535342742	4.476256667	9.80607163	0.0027868	0.0096817
**ENSG00000070831**	CDC42	0.230730671	7.951518057	9.762825807	0.0027896	0.0096852
**ENSG00000131845**	ZNF304	0.462159063	4.216325441	9.762800237	0.0027897	0.0096852
**ENSG00000008838**	MIR6884	-0.321907768	5.398465271	9.761742941	0.0027910	0.0096868
**ENSG00000243156**	MICAL3	0.37247986	5.337766024	9.760164167	0.0027931	0.0096907
**ENSG00000151665**	PIGF	-0.344391705	4.875129735	9.756886524	0.0027973	0.0097022
**ENSG00000166716**	ZNF592	0.231459578	6.527092567	9.7526326	0.0028029	0.0097181
**ENSG00000272742**		0.38359579	5.833237115	9.876203796	0.0028095	0.009738
**ENSG00000089682**	RBM41	-0.37577903	5.075635612	9.742983703	0.0028154	0.0097553
**ENSG00000152683**	SLC30A6	0.256870898	5.827866947	9.739176142	0.0028204	0.0097693
**ENSG00000204131**	NHSL2	0.453462203	6.408918558	10.15381069	0.0028304	0.0098005
**ENSG00000089094**	MIR7107	0.24577946	6.300675104	9.726886869	0.0028366	0.0098187
**ENSG00000105127**	AKAP8	-0.348850235	5.736340752	9.720234244	0.0028453	0.0098459
**ENSG00000050130**	JKAMP	-0.339062904	5.060474226	9.718822085	0.0028472	0.0098491
**ENSG00000123411**	IKZF4	0.479344137	4.148214755	9.716235542	0.0028506	0.0098576
**ENSG00000101191**	DIDO1	-0.200007938	7.540644057	9.710069442	0.0028588	0.0098816
**ENSG00000162086**	ZNF75A	-0.282925795	5.721140057	9.709577441	0.0028595	0.0098816
**ENSG00000259075**	POC1B-GALNT4	0.365069749	4.834729999	9.708385577	0.0028611	0.0098838
**ENSG00000167393**	PPP2R3B	-0.628162304	3.488658782	9.705628785	0.0028647	0.0098932
**ENSG00000139725**	RHOF	0.21610892	6.916771859	9.694643586	0.0028794	0.0099356
**ENSG00000089818**	NECAP1	-0.282282123	5.630114677	9.694111422	0.0028801	0.0099356
**ENSG00000117877**	CD3EAP	-0.508170333	3.845140947	9.694080948	0.0028801	0.0099356
**ENSG00000108219**	TSPAN14	-0.17309902	7.750891951	9.693498322	0.0028809	0.0099356

**ENSG00000119929**	CUTC	-0.368912978	4.404937027	9.692875091	0.0028817	0.0099356
**ENSG00000163154**	TNFAIP8L2	0.42624142	4.578702214	9.690595768	0.0028848	0.0099429
**ENSG00000135372**	NAT10	-0.237756556	5.980524907	9.68897219	0.0028870	0.0099471
**ENSG00000171806**	METTL18	0.627083628	3.8714111	9.724397923	0.0028953	0.0099725
**ENSG00000132436**	FIGNL1	-0.39827135	4.639270716	9.677765531	0.0029021	0.0099925
**ENSG00000242028**	HYPK	-0.384720322	5.004018712	9.676116479	0.0029043	0.0099969
**ENSG00000172671**	ZFAND4	-0.35691019	5.070593154	9.672295875	0.0029094	0.0100113
**ENSG00000135486**	HNRNPA1	-0.193063011	9.636650327	9.670264181	0.0029122	0.0100175
**ENSG00000148834**	GSTO1	0.444728011	4.03422426	9.667212463	0.0029163	0.0100284
**ENSG00000178691**	SUZ12	0.253876254	6.750320761	9.657025196	0.0029302	0.0100728
**ENSG00000185787**	MORF4L1	0.16516951	7.73516	9.65303989	0.0029356	0.0100881
**ENSG00000249592**	LOC100129917	-0.543593154	4.462835177	9.698663988	0.0029412	0.0101040
**ENSG00000198924**	DCLRE1A	0.593784248	3.874768633	9.647044992	0.0029438	0.0101081
**ENSG00000185033**	SEMA4B	0.538423727	3.465286092	9.646666456	0.0029443	0.0101081
**ENSG00000083123**	BCKDHB	-0.451928528	4.218251076	9.643754411	0.0029483	0.0101185
**ENSG00000096060**	FKBP5	-0.242005419	7.839507211	9.671080381	0.0029633	0.0101665
**ENSG00000068697**	laptm4a	0.374539672	5.324966381	9.631315304	0.0029654	0.0101706
**ENSG00000162664**	ZNF326	-0.363421307	6.086990463	9.778806952	0.0029730	0.0101932
**ENSG00000226438**		0.456217775	4.074444237	9.615674079	0.0029871	0.0102382
**ENSG00000162066**	AMDHD2	-0.615415306	3.692522672	9.612948812	0.0029909	0.0102478
**ENSG00000071889**	FAM3A	-0.579361704	3.926054236	9.599472384	0.0030097	0.0103080
**ENSG00000167207**	NOD2	0.539780666	3.495652535	9.598959811	0.0030105	0.0103080
**ENSG00000204469**	PRRC2A	0.195291101	7.553268593	9.59739708	0.0030126	0.0103122
**ENSG00000103494**	RPGRIP1L	-0.55380853	3.774300017	9.588666518	0.0030249	0.0103508
**ENSG00000172840**	PDP2	0.523861731	4.33567006	9.581842463	0.0030346	0.0103803
**ENSG00000211795**		0.564863833	3.941568978	9.577376921	0.0030409	0.0103958
**ENSG00000100897**	DCAF11	-0.27266601	5.772406356	9.577236043	0.0030411	0.0103958
**ENSG00000231205**		0.517456299	3.646976578	9.565927568	0.0030571	0.0104474
**ENSG00000071189**	SNX13	-0.278164811	6.496544416	9.563957839	0.0030600	0.0104535
**ENSG00000154640**	btg3	0.51035625	3.948547585	9.563011492	0.0030613	0.0104547
**ENSG00000236991**	EDRF1-AS1	-0.498161084	4.14064065	9.561417673	0.0030636	0.0104591
**ENSG00000106404**	CLDN15	-0.605879093	3.666028303	9.549479685	0.0030807	0.0105140
**ENSG00000255680**		0.582412205	3.363281348	9.534137864	0.0031028	0.0105861
**ENSG00000099341**	PSMD8	0.316980312	4.917893366	9.533422133	0.0031038	0.0105861
**ENSG00000115091**	ACTR3	0.174015509	8.703008657	9.52320719	0.0031187	0.0106332
**ENSG00000148120**	mir24-1	-0.5906778	3.53776168	9.522122968	0.0031202	0.0106349
**ENSG00000142657**	PGD	0.436565178	4.288059456	9.521468301	0.0031212	0.0106349

**ENSG00000279933**		-0.460775394	5.117540715	9.613255281	0.003126	0.010649
**ENSG00000165795**	MIR6717	-0.568395668	3.463329161	9.51523144	0.003130	0.010657
**ENSG00000182087**	TMEM259	-0.594993276	6.051478446	10.00160839	0.003130	0.010657
**ENSG00000109606**	DHX15	-0.183451911	7.629419381	9.513214456	0.003133	0.010662
**ENSG00000169925**	BRD3	0.287045005	5.675446488	9.508081109	0.003140	0.010684
**ENSG00000261684**		-0.406231831	4.744263418	9.504076801	0.003146	0.010700
**ENSG00000105438**	KDELR1	0.41833294	4.784449341	9.500991335	0.003151	0.010712
**ENSG00000184207**	PGP	-0.546638387	3.520504263	9.498789898	0.003154	0.010720
**ENSG00000168918**	INPP5D	0.18184364	7.529178798	9.485358844	0.003174	0.010784
**ENSG00000142208**	AKT1	0.293610098	5.561607401	9.479839481	0.003182	0.010808
**ENSG00000114416**	FXR1	-0.170785905	7.635477627	9.472985802	0.003192	0.010839
**ENSG00000131375**	CAPN7	-0.273189258	6.800577431	9.449968455	0.003242	0.011003
**ENSG00000170089**	LOC728554	-0.472372036	4.254145904	9.438515787	0.003244	0.011008
**ENSG00000125977**	EIF2S2	0.246164256	6.352227319	9.437161608	0.003246	0.011011
**ENSG00000258465**		-0.3070392	6.070703645	9.435589713	0.003248	0.011016
**ENSG00000138035**	PNPT1	-0.425834017	5.614417996	9.615945669	0.003253	0.011028
**ENSG00000103353**	UBFD1	-0.305102356	5.13565625	9.43056512	0.003256	0.011035
**ENSG00000008277**	ADAM22	-0.541745341	4.440267986	9.465146505	0.003265	0.011063
**ENSG00000167302**	TEPSIN	-0.564782249	4.091172348	9.421902364	0.003269	0.011072
**ENSG00000128000**	ZNF780B	-0.283460108	6.238117869	9.420924014	0.003271	0.011074
**ENSG00000010322**	NISCH	-0.400780997	6.351086162	9.726741735	0.003280	0.011102
**ENSG00000279069**		-0.445181751	4.261877582	9.398288313	0.003306	0.011184
**ENSG00000271949**		0.404180277	4.498957344	9.392754357	0.003314	0.011210
**ENSG00000173821**	RNF213	0.191974603	10.35774316	9.391722402	0.003316	0.011211
**ENSG00000185825**	BCAP31	0.319569209	5.639235876	9.389262709	0.003320	0.011221
**ENSG00000064726**	BTBD1	0.250449915	5.812645642	9.380323007	0.003334	0.011264
**ENSG00000131584**	acap3	-0.595980748	4.394133396	9.493901094	0.003347	0.011305
**ENSG00000154764**	WNT7A	-0.501151501	3.773955306	9.360487332	0.003365	0.011362
**ENSG00000109180**	OCIAD1	-0.246303729	6.41694235	9.356703533	0.003371	0.011378
**ENSG00000143443**	C1orf56	-0.206165035	6.679628425	9.353580973	0.003376	0.011391
**ENSG00000171444**	MCC	0.416369444	4.493976708	9.351638948	0.003379	0.011398
**ENSG00000279541**		0.307875732	5.202269482	9.35015418	0.003381	0.011402
**ENSG00000165813**	MIR2110	0.246477427	7.057673865	9.343623069	0.003391	0.011433
**ENSG00000136240**	KDELR2	0.295415957	5.643353589	9.3410998	0.003395	0.011443
**ENSG00000071205**	ARHGAP10	0.572765362	3.926793382	9.339456033	0.003398	0.011448
**ENSG00000151726**	ACSL1	0.448785388	4.011579126	9.338062752	0.003400	0.011451
**ENSG00000179583**	ciita	0.474065213	4.111624173	9.337657198	0.003401	0.011451

**ENSG00000134996**	OSTF1	0.257085865	6.06274756	9.334898008	0.0034057	0.0114623
**ENSG00000174456**	C12orf76	-0.353246858	4.953661045	9.32858977	0.0034157	0.0114925
**ENSG00000171204**	TMEM126B	-0.341634872	5.063069967	9.314183478	0.0034389	0.0115667
**ENSG00000279344**		-0.254599643	6.854407833	9.31188449	0.0034426	0.0115754
**ENSG00000164902**	PHAX	0.328109932	5.574092274	9.310048163	0.0034456	0.0115817
**ENSG00000080503**	SMARCA2	0.167647579	8.749693845	9.309279178	0.0034468	0.0115821
**ENSG00000163947**	ARHGEF3	0.18912457	7.745554928	9.305175554	0.0034534	0.0116007
**ENSG00000048707**	VPS13D	0.174201157	8.283071969	9.297864436	0.0034653	0.0116368
**ENSG00000168374**	ARF4	0.292691643	5.598012852	9.296277808	0.0034679	0.0116414
**ENSG00000166848**	LOC105371348	0.241471533	6.605390755	9.295652451	0.0034689	0.0116414
**ENSG00000196284**	SUPT3H	-0.414655065	4.487022876	9.294944916	0.0034700	0.0116415
**ENSG00000108091**	CCDC6	0.256863167	6.257929303	9.293154159	0.0034730	0.0116460
**ENSG00000187735**	TCEA1	0.26066496	6.348799759	9.292754507	0.0034736	0.0116460
**ENSG00000246922**	UBAP1L	-0.53272949	4.209212171	9.290502668	0.0034773	0.0116546
**ENSG00000159596**	TMEM69	-0.394264269	4.558913142	9.284482979	0.0034871	0.0116838
**ENSG00000130511**	SSBP4	0.548554741	4.265694877	9.300070533	0.0034995	0.0117216
**ENSG00000188554**	NBR1	0.189219305	7.03940372	9.273147386	0.0035057	0.0117385
**ENSG00000165512**	ZNF22	0.313159703	5.112256611	9.27028269	0.0035104	0.0117496
**ENSG00000234945**	GTF3C2-AS1	-0.452117709	4.016593245	9.269785313	0.0035112	0.0117496
**ENSG00000156239**	N6AMT1	-0.502066576	4.253061681	9.264195829	0.0035205	0.0117766
**ENSG00000267633**		-0.652076936	4.074139142	9.368558661	0.0035219	0.0117777
**ENSG00000113966**	ARL 6.00	-0.482304548	3.54704554	9.259816834	0.0035277	0.0117896
**ENSG00000178127**	NDUFV2	-0.299981553	5.689761158	9.259804429	0.0035277	0.0117896
**ENSG00000205542**	TMSB4X	0.282297254	10.53505713	9.414602246	0.0035289	0.0117899
**ENSG00000106052**	TAX1BP1	0.215794243	7.501144551	9.25430687	0.0035368	0.0118093
**ENSG00000160959**	LRRC14	-0.378831313	4.663769495	9.254210334	0.0035370	0.0118093
**ENSG00000055163**	cyfip2	0.171178584	9.342069292	9.252886953	0.0035392	0.0118119
**ENSG00000168014**	C2CD3	-0.254249596	6.100079945	9.252375314	0.0035400	0.0118119
**ENSG00000156639**	ZFAND3	0.269774749	5.480725333	9.24094971	0.0035591	0.0118717
**ENSG00000174579**	MSL2	0.187951429	7.505227544	9.234841981	0.0035693	0.0119020
**ENSG00000065911**	mthfd2	0.352867915	5.060046558	9.229364456	0.0035785	0.0119288
**ENSG00000165983**	PTER	0.298248279	5.378114728	9.22630066	0.0035836	0.0119422
**ENSG00000134759**	ELP2	-0.207042851	7.062344467	9.218451078	0.0035969	0.0119789
**ENSG00000205236**		-0.491691818	3.954371394	9.218212813	0.0035973	0.0119789
**ENSG00000177733**	HNRNPA0	0.24853244	6.322172049	9.217735931	0.0035981	0.0119789
**ENSG00000177189**	RPS6KA3	0.156205019	8.40717387	9.216828707	0.0035996	0.0119802
**ENSG00000164414**	slc35a1	-0.307313519	5.112896052	9.211531605	0.0036086	0.0120062

**ENSG00000237945**	LINC00649	0.189022678	7.482566422	9.209811587	0.0036115	0.0120121
**ENSG00000188092**	GPR89B	-0.562184246	3.751963695	9.207757313	0.0036150	0.0120199
**ENSG00000184203**	PPP1R2	-0.208450741	7.583929173	9.189904733	0.0036455	0.0121173
**ENSG00000170515**	PA2G4	0.24469011	6.523428216	9.188212153	0.0036484	0.0121231
**ENSG00000164190**	NIPBL	0.172965377	8.783346182	9.171933159	0.0036764	0.0122123
**ENSG00000197603**	c5orf42	-0.403223173	5.511681875	9.266708071	0.0036799	0.0122201
**ENSG00000158710**	TAGLN2	-0.347935534	6.648075561	9.39935944	0.0036835	0.0122283
**ENSG00000198718**	FAM179B	-0.381739953	4.822878268	9.166586472	0.0036856	0.0122314
**ENSG00000204149**	AGAP6	-0.703144537	4.345457526	9.440127782	0.0036905	0.0122436
**ENSG00000170266**	GLB1	0.352145444	4.64271577	9.156548779	0.0037031	0.0122815
**ENSG00000180336**	MEIOC	-0.516610859	3.683799901	9.150153944	0.0037142	0.0123146
**ENSG00000180257**	ZNF816	0.42692658	4.72880232	9.147889384	0.0037182	0.0123238
**ENSG00000140350**	ANP32A	0.235593209	6.572340665	9.145795953	0.0037219	0.0123321
**ENSG00000215769**	LOC146880	-0.664387541	4.819218277	9.48601293	0.0037447	0.0124024
**ENSG00000135775**	COG2	-0.257116264	5.902468002	9.132357786	0.0037455	0.0124024
**ENSG00000111780**		0.442866849	4.445042467	9.129570689	0.0037504	0.0124148
**ENSG00000255185**		-0.522256394	4.425999958	9.151916418	0.0037557	0.0124284
**ENSG00000065809**	FAM107B	0.193301268	8.117610037	9.123906505	0.0037604	0.0124401
**ENSG00000141404**	GNAL	-0.529121258	3.80892823	9.12204907	0.0037637	0.0124467
**ENSG00000209082**		-0.332271502	6.538044208	9.27939153	0.0037648	0.0124467
**ENSG00000269352**		-0.548824455	4.040566105	9.115519787	0.0037753	0.0124774
**ENSG00000169223**	LMAN2	0.3272334	5.580882492	9.113044934	0.0037797	0.0124877
**ENSG00000171033**	PKIA	-0.354172078	4.940392324	9.112435845	0.0037808	0.0124877
**ENSG00000083535**	PIBF1	-0.292155525	5.947194657	9.110651381	0.0037840	0.0124942
**ENSG00000083097**	DOPEY1	-0.215542463	6.761422627	9.108734709	0.0037874	0.0125016
**ENSG00000167548**	KMT2D	0.192593159	8.341899816	9.102266986	0.0037989	0.0125357
**ENSG00000113163**	COL4A3BP	0.243292531	6.459382192	9.09894932	0.0038049	0.0125514
**ENSG00000136854**	STXBP1	-0.533005865	3.75664847	9.094286218	0.0038132	0.0125750
**ENSG00000126698**	DNAJC8	0.292957839	6.144762369	9.09118595	0.0038188	0.0125894
**ENSG00000069329**	VPS35	0.189141789	7.040902897	9.080853935	0.0038374	0.0126469
**ENSG00000066084**	DIP2B	-0.262097923	6.827916353	9.078942292	0.0038409	0.0126529
**ENSG00000160746**	ANO10	-0.552769579	3.397556369	9.078515028	0.0038417	0.0126529
**ENSG00000099899**	TRMT2A	-0.441682313	4.458393267	9.071993043	0.0038535	0.0126878
**ENSG00000119523**	ALG2	0.334157828	4.66048045	9.070610051	0.0038560	0.0126921
**ENSG00000271853**		-0.483397061	3.774462367	9.06946872	0.0038581	0.0126949
**ENSG00000132676**	DAP3	-0.21544278	6.394594795	9.067412052	0.0038618	0.0127032
**ENSG00000144320**	LNPK	0.25858525	6.020012064	9.065469599	0.0038654	0.0127109

**ENSG00000166479**	TMX3	-0.245109006	6.733810053	9.062811592	0.0038702	0.0127192
**ENSG00000173914**	RBM4B	-0.341142126	4.974402079	9.062746005	0.0038703	0.0127192
**ENSG00000144021**	CIAO1	-0.289214983	5.981034863	9.057264526	0.0038804	0.0127481
**ENSG00000258588**	TRIM6-TRIM34	-0.468458084	3.825758585	9.036540894	0.0039185	0.0128693
**ENSG00000185101**	ANO9	-0.664259073	5.636524215	9.477614912	0.0039217	0.0128760
**ENSG00000117305**	HMGCL	-0.542618646	3.659111378	9.032359733	0.0039262	0.0128866
**ENSG00000120802**	TMPO	0.199207856	7.774953008	9.02295352	0.0039437	0.0129374
**ENSG00000180329**	CCDC43	-0.46376519	4.200935629	9.022696221	0.0039441	0.0129374
**ENSG00000178904**	DPY19L3	-0.43911007	4.566744231	9.008496536	0.0039707	0.0130177
**ENSG00000241878**	MIR7109	-0.461783924	4.602929067	9.008244956	0.0039711	0.0130177
**ENSG00000154874**	Ccdc144b	-0.427581467	4.859745977	9.006232937	0.0039749	0.0130260
**ENSG00000055609**	KMT2C	0.177330808	9.146727058	9.002126003	0.0039826	0.0130472
**ENSG00000111642**	CHD4	0.183622493	7.844246012	8.997073417	0.0039921	0.0130711
**ENSG00000181704**	YIPF6	-0.382156244	5.06434636	8.996931919	0.0039924	0.0130711
**ENSG00000242247**	ARFGAP3	0.297212256	5.479805926	8.992364811	0.0040010	0.0130920
**ENSG00000198879**	SFMBT2	-0.30077327	5.89870755	8.992219665	0.0040013	0.0130920
**ENSG00000100038**	TOP3B	-0.528224765	4.830360405	9.151903045	0.0040213	0.0131534
**ENSG00000121892**	PDS5A	0.170874739	8.388739559	8.977224529	0.0040297	0.0131768
**ENSG00000140319**	SRP14	0.244462574	6.844863754	8.976409477	0.0040313	0.0131777
**ENSG00000255339**	NDUFB8	-0.27616308	5.54519952	8.968745929	0.0040459	0.0132214
**ENSG00000260465**		-0.357305799	4.57330019	8.965317453	0.0040525	0.0132387
**ENSG00000231799**		-0.57122208	3.859239691	8.939867456	0.0041015	0.0133947
**ENSG00000134802**	SLC43A3	0.493932564	3.668062531	8.934995202	0.0041110	0.0134214
**ENSG00000137815**	rtf1	0.247309722	6.356594695	8.932508016	0.0041158	0.0134330
**ENSG00000172716**	SLFN11	0.341414689	5.102103151	8.930504916	0.0041197	0.0134415
**ENSG00000183576**	SETD3	0.270047809	5.792430887	8.929036151	0.0041226	0.0134467
**ENSG00000164338**	UTP15	-0.279814202	5.190979763	8.928223434	0.0041242	0.0134477
**ENSG00000179532**	DNHD1	-0.498697233	6.282055358	9.3375109	0.0041323	0.0134699
**ENSG00000260272**		0.401567205	5.368327699	8.983652869	0.0041357	0.0134769
**ENSG00000145241**	CENPC	-0.257152726	7.222498366	8.966315249	0.0041410	0.0134900
**ENSG00000111144**	LTA4H	-0.251338179	6.170035823	8.917099602	0.0041459	0.0135018
**ENSG00000152942**	RAD17	-0.304370348	5.396505242	8.915922982	0.0041482	0.0135051
**ENSG00000226121**		0.541837576	3.389094967	8.913053867	0.0041539	0.0135192
**ENSG00000282804**		-0.315695745	5.222117121	8.893881508	0.0041917	0.0136382
**ENSG00000170606**	HSPA4	0.237303062	6.637057827	8.885010258	0.0042093	0.0136913
**ENSG00000261884**		-0.370530392	5.519478307	8.919473874	0.0042113	0.0136935
**ENSG00000158092**	NCK1	0.262944272	5.764086851	8.882955184	0.0042134	0.0136961

**ENSG00000087087**	SRRT	-0.256486222	6.155135051	8.879846581	0.0042197	0.0137120
**ENSG00000196312**	MFSD14C	-0.496433479	4.173768149	8.878473033	0.0042224	0.0137167
**ENSG00000117155**	SSX2IP	-0.437998884	4.228901782	8.877366045	0.0042246	0.0137172
**ENSG00000249915**	PDCD6	-0.296763846	5.380853504	8.877081493	0.0042252	0.0137172
**ENSG00000243943**	ZNF512	-0.270393324	5.749216594	8.874831592	0.0042297	0.0137275
**ENSG00000072518**	MARK2	0.208264434	6.283804438	8.866806942	0.0042458	0.0137755
**ENSG00000168806**	lcmt2	0.528199259	3.573631344	8.853351893	0.0042729	0.0138593
**ENSG00000167658**	EEF2	0.2255879	10.32073636	8.852695509	0.0042743	0.0138593
**ENSG00000005700**	IBTK	0.21388401	6.61684723	8.843321758	0.0042933	0.0139167
**ENSG00000171262**	FAM98B	-0.274237292	6.61103835	8.848679669	0.0042958	0.0139205
**ENSG00000178980**	SELENOW	0.389108277	4.788094807	8.839433218	0.0043012	0.0139337
**ENSG00000172175**	MALT1	-0.206609051	7.869676317	8.838682113	0.0043027	0.0139344
**ENSG00000213347**	MXD3	0.311673794	4.884429974	8.829907982	0.0043207	0.0139881
**ENSG00000172687**		-0.535120748	3.376290655	8.829056693	0.0043224	0.0139886
**ENSG00000144711**	IQSEC1	0.196193778	7.121507893	8.828527096	0.0043235	0.0139886
**ENSG00000100084**	HIRA	0.275088587	5.349213677	8.826891773	0.0043269	0.0139951
**ENSG00000120686**	UFM1	-0.230756897	6.693900942	8.816884092	0.0043474	0.0140562
**ENSG00000225031**		0.542986577	3.603088156	8.816405225	0.0043484	0.0140562
**ENSG00000090924**	PLEKHG2	-0.394479089	5.421892523	8.872370326	0.0043620	0.0140958
**ENSG00000276057**		-0.559722304	3.731744851	8.806731396	0.0043684	0.0141121
**ENSG00000109670**	FBXW7	0.238650873	6.825599308	8.79850008	0.0043855	0.0141629
**ENSG00000081791**	KIAA0141	-0.324437245	5.743880899	8.796049971	0.0043906	0.0141725
**ENSG00000172954**	LCLAT1	-0.352637711	5.20782588	8.795766433	0.0043912	0.0141725
**ENSG00000197530**	MIB2	-0.553046387	4.194531896	8.814035836	0.0044048	0.0142077
**ENSG00000171791**	BCL2	0.251138097	8.840638647	8.902416233	0.0044048	0.0142077
**ENSG00000187951**	LOC100288637	0.631643884	3.809728704	8.83582929	0.0044135	0.0142314
**ENSG00000140386**	SCAPER	-0.276700301	6.196081311	8.777173411	0.0044301	0.0142805
**ENSG00000150637**	CD226	0.188524988	7.886882692	8.773422232	0.0044380	0.0143006
**ENSG00000181523**	SGSH	-0.354958085	5.577678792	8.786710347	0.0044400	0.0143006
**ENSG00000123143**	PKN1	0.286974992	5.813992027	8.772118588	0.0044407	0.0143006
**ENSG00000145868**	FBXO38	-0.244328168	6.357211151	8.771606725	0.0044418	0.0143006
**ENSG00000177932**	ZNF354C	-0.359028335	4.699755455	8.762713966	0.0044606	0.0143567
**ENSG00000103248**	MTHFSD	-0.41771948	4.087835097	8.760976492	0.0044643	0.0143642
**ENSG00000257181**		0.412600663	4.603879562	8.755057918	0.0044769	0.0143978
**ENSG00000180867**		0.489294453	3.754727834	8.754749487	0.0044775	0.0143978
**ENSG00000083937**	CHMP2B	0.32337712	5.473061028	8.753093136	0.0044810	0.0144047
**ENSG00000154127**	UBASH3B	0.291327866	5.928601822	8.750210996	0.0044872	0.0144200

**ENSG00000205531**	NAP1L4	-0.166205933	7.687254922	8.746119046	0.0044959	0.0144436
**ENSG00000160209**	LOC105372824	0.302836225	5.748890935	8.737228888	0.0045149	0.0145003
**ENSG00000254876**	LOC100499484	-0.518480394	3.949224804	8.729931261	0.0045306	0.0145424
**ENSG00000170322**	NFRKB	-0.28024281	6.100596239	8.729840368	0.0045308	0.0145424
**ENSG00000198931**	APRT	0.419088395	4.559161671	8.723751036	0.0045439	0.0145800
**ENSG00000135046**	ANXA1	-0.292728399	8.251851096	8.964849523	0.0045644	0.0146413
**ENSG00000163840**	DTX3L	0.221872823	7.353018058	8.71040402	0.0045729	0.0146607
**ENSG00000178764**	ZHX2	0.249813571	5.93281232	8.710208351	0.0045733	0.0146607
**ENSG00000269972**		-0.34019747	5.271195684	8.698781238	0.0045982	0.0147360
**ENSG00000167986**	DDB1	0.186657255	6.953875026	8.691955888	0.0046131	0.0147794
**ENSG00000175470**	PPP2R2D	-0.298520439	5.728717113	8.683941295	0.0046308	0.0148313
**ENSG00000204209**	DAXX	0.29270483	5.69267583	8.683004991	0.0046328	0.0148334
**ENSG00000158195**	WASF2	0.175545266	7.68948409	8.677817145	0.0046443	0.0148627
**ENSG00000188725**	SMIM15	0.368939509	4.987219955	8.677272293	0.0046455	0.0148627
**ENSG00000163513**	TGFBR2	-0.208268599	8.074365791	8.676927665	0.0046462	0.0148627
**ENSG00000119778**	ATAD2B	-0.237766347	6.714742008	8.674488437	0.0046516	0.0148754
**ENSG00000260404**		-0.464383126	5.053143782	8.777289948	0.0046648	0.0149129
**ENSG00000134318**	ROCK2	0.235869315	6.800415932	8.66504674	0.0046726	0.0149332
**ENSG00000140511**	HAPLN3	-0.493932429	5.096514912	8.834139527	0.0046833	0.0149628
**ENSG00000143167**	GPA33	-0.493675727	4.063382053	8.655734885	0.0046933	0.0149904
**ENSG00000196705**	ZNF431	-0.246344417	6.658633838	8.651535114	0.0047027	0.0150158
**ENSG00000152926**	ZNF117	-0.477527244	4.723859616	8.684833202	0.0047081	0.0150284
**ENSG00000188167**	TMPPE	0.449248871	3.890846712	8.645680882	0.0047158	0.0150485
**ENSG00000102390**	PBDC1	0.429075939	4.329708684	8.633126699	0.0047441	0.0151341
**ENSG00000113712**	CSNK1A1	0.161687289	7.924359452	8.632070601	0.0047465	0.0151371
**ENSG00000141446**	ESCO1	0.239872269	6.596318567	8.630920027	0.0047491	0.0151408
**ENSG00000277791**	PSMB3	0.455202365	4.768051465	8.661965941	0.0047567	0.0151602
**ENSG00000105401**	MIR1181	-0.249472965	5.941839061	8.626685089	0.0047587	0.0151621
**ENSG00000078319**		-0.445204615	4.20422073	8.623115279	0.0047668	0.0151813
**ENSG00000278274**	Snora61	1.163239987	5.012021054	9.040963245	0.0047676	0.0151813
**ENSG00000069345**	DNAJA2	0.223816545	6.433131911	8.617331187	0.0047799	0.0152159
**ENSG00000121895**	TMEM156	0.405789716	4.849615477	8.614179881	0.0047871	0.0152341
**ENSG00000120868**	APAF1	0.276458467	6.006491467	8.61133165	0.0047936	0.0152502
**ENSG00000157916**	RER1	-0.235643972	5.994520017	8.607035354	0.0048035	0.0152768
**ENSG00000147854**	UHRF2	-0.224566129	7.21328441	8.603798371	0.0048109	0.0152957
**ENSG00000258908**		0.536965972	3.922223739	8.602837515	0.0048131	0.0152981
**ENSG00000111348**	ARHGDIB	0.178975065	9.647542704	8.597863898	0.0048245	0.0153297

**ENSG00000154025**	SLC5A10	-0.323939878	4.880908501	8.593979228	0.0048334	0.0153535
**ENSG00000144559**	TAMM41	-0.363578495	5.174224714	8.592699768	0.0048364	0.0153582
**ENSG00000101350**	KIF3B	0.309365241	5.334072686	8.589765905	0.0048432	0.0153750
**ENSG00000066933**	MYO9A	0.235007418	6.896960696	8.588176047	0.0048468	0.0153820
**ENSG00000148411**	NACC2	0.43964169	4.151842846	8.583478779	0.0048577	0.0154118
**ENSG00000177885**	GRB2	0.21581665	6.519249032	8.577374149	0.0048719	0.0154506
**ENSG00000180628**	PCGF5	0.209545237	7.744294764	8.576931704	0.0048729	0.0154506
**ENSG00000037749**	MFAP3	0.358396218	4.692333365	8.576205166	0.0048746	0.0154513
**ENSG00000156050**	FAM161B	0.374516679	4.441583291	8.575366485	0.0048765	0.0154528
**ENSG00000070061**	IKBKAP	-0.260762881	6.183323523	8.573845603	0.0048801	0.0154593
**ENSG00000168488**	ATXN2L	-0.216916215	7.321304219	8.571419739	0.0048857	0.0154720
**ENSG00000113360**	DROSHA	-0.230489353	6.39389901	8.570846177	0.0048870	0.0154720
**ENSG00000170854**	MINA	-0.252606556	5.596760481	8.565252396	0.0049001	0.0155087
**ENSG00000189308**	LIN54	0.245136171	5.840911534	8.56438517	0.0049021	0.0155104
**ENSG00000142751**	GPN2	-0.358691254	4.586760458	8.562984834	0.0049054	0.0155161
**ENSG00000162929**	KIAA1841	0.59148507	3.607656588	8.559566845	0.0049134	0.0155367
**ENSG00000103044**	HAS3	0.52648024	3.778063925	8.558241327	0.0049165	0.0155418
**ENSG00000254999**	BRK1	0.358104528	5.038653655	8.548913647	0.0049384	0.0156064
**ENSG00000168724**	DNAJC21	0.223553579	6.533485856	8.546798621	0.0049434	0.0156175
**ENSG00000172183**	ISG20	0.232316282	6.443754276	8.545303543	0.0049470	0.0156239
**ENSG00000260114**		-0.565714859	3.949269514	8.550239063	0.0049565	0.0156494
**ENSG00000149016**	TUT1	-0.514135807	3.578361748	8.53711373	0.0049663	0.0156756
**ENSG00000267481**		-0.51406417	4.476418875	8.585614285	0.0049711	0.0156851
**ENSG00000182481**	KPNA2	0.478568271	4.178031602	8.534587985	0.0049723	0.0156851
**ENSG00000153015**	CWC27	-0.357650817	5.051209316	8.533106019	0.0049758	0.0156914
**ENSG00000108679**	LGALS3BP	-0.361882714	4.676308164	8.531233286	0.0049803	0.0157007
**ENSG00000123600**	METTL8	0.367330906	4.929296922	8.530462183	0.0049821	0.0157018
**ENSG00000126012**	KDM5C	-0.216803708	6.928156328	8.522766336	0.0050005	0.0157548
**ENSG00000198373**	WWP2	0.207197396	6.128585902	8.518807302	0.0050099	0.0157795
**ENSG00000168228**	ZCCHC4	-0.435106775	4.518361862	8.518221276	0.0050113	0.0157795
**ENSG00000143373**	ZNF687	0.329332207	4.967830116	8.517595791	0.0050128	0.0157795
**ENSG00000110917**	MLEC	0.284535122	5.869189683	8.516760195	0.0050148	0.0157811
**ENSG00000151748**	SAV1	0.405661931	4.320495264	8.512519544	0.0050250	0.0158083
**ENSG00000087086**	FTL	0.289294524	8.337422504	8.753585821	0.0050329	0.0158282
**ENSG00000005483**	KMT2E	0.159477387	9.121514525	8.505309312	0.0050423	0.0158533
**ENSG00000241553**	ARPC4	0.248684748	6.248235435	8.500084247	0.0050549	0.0158853
**ENSG00000075292**	ZNF638	-0.188847745	8.172839617	8.499831226	0.0050556	0.0158853

**ENSG00000125814**	NAPB	-0.399679323	4.563490455	8.498655464	0.0050584	0.0158867
**ENSG00000183484**	GPR132	0.355534888	5.241965643	8.498062202	0.0050598	0.0158867
**ENSG00000116903**	EXOC8	0.238615246	5.964386991	8.497762713	0.0050606	0.0158867
**ENSG00000135249**	RINT1	-0.253888977	5.459007497	8.493631927	0.0050705	0.0159133
**ENSG00000197183**	NOL4L	-0.228006768	7.020204368	8.492494683	0.0050733	0.0159172
**ENSG00000185883**	ATP6V0C	0.391577811	5.406322482	8.555718694	0.0050819	0.0159392
**ENSG00000107798**	lipA	0.301683079	5.652961122	8.486555635	0.0050877	0.0159528
**ENSG00000165282**	PIGO	-0.380081049	4.531702756	8.484536777	0.0050926	0.0159634
**ENSG00000054118**	THRAP3	0.17825314	7.587064413	8.482542452	0.0050975	0.0159739
**ENSG00000117335**	CD46	0.145838197	7.91367567	8.476000868	0.0051135	0.0160191
**ENSG00000111879**	FAM184A	-0.590872912	4.190572713	8.558839271	0.0051459	0.0161160
**ENSG00000280607**		0.437098881	4.34840721	8.46175889	0.0051484	0.0161189
**ENSG00000103051**	COG4	-0.328783315	5.270759743	8.459185415	0.0051547	0.0161339
**ENSG00000204231**	RXRB	-0.409099441	5.039529574	8.463829147	0.0051633	0.0161560
**ENSG00000215908**		-0.382593996	5.624146994	8.551284149	0.0051734	0.0161826
**ENSG00000135966**	TGFBRAP1	0.290899093	5.385334832	8.440849484	0.0052001	0.0162615
**ENSG00000141252**	VPS53	-0.217219057	6.351958813	8.439381217	0.0052038	0.0162680
**ENSG00000108588**	CCDC47	0.26465234	6.010216744	8.436500618	0.0052110	0.0162856
**ENSG00000277147**	LINC00869	-0.317412099	5.117080743	8.432514645	0.0052209	0.0163059
**ENSG00000136141**	LRCH1	0.262646917	5.380344455	8.432141123	0.0052219	0.0163059
**ENSG00000267523**		-0.58420088	3.648047195	8.432026925	0.0052221	0.0163059
**ENSG00000156162**	DPY19L4	-0.306586074	5.237811105	8.429576905	0.0052283	0.0163201
**ENSG00000057252**	SOAT1	0.200209254	6.730778355	8.424693427	0.0052405	0.0163534
**ENSG00000159461**	AMFR	0.279440336	5.757460369	8.418864275	0.0052552	0.0163942
**ENSG00000146826**	MIR4658	-0.457011702	4.639026173	8.422699848	0.0052592	0.0164021
**ENSG00000159459**	UBR1	0.196962103	6.929371935	8.416289605	0.0052616	0.0164047
**ENSG00000167173**	c15orf39	0.370579069	4.81298823	8.411714251	0.0052732	0.0164357
**ENSG00000260032**	NORAD	0.184592391	8.668676971	8.408587546	0.0052811	0.0164555
**ENSG00000088682**	COQ9	-0.515664339	3.997673182	8.407498209	0.0052838	0.0164591
**ENSG00000115459**	ELMOD3	-0.41278753	4.706734762	8.406675307	0.0052859	0.0164607
**ENSG00000218537**		0.586323347	5.328258044	8.746133813	0.0052883	0.0164631
**ENSG00000152784**	PRDM8	0.387909877	4.647153158	8.403884213	0.0052930	0.0164729
**ENSG00000197162**	ZNF785	-0.470843543	4.116810983	8.402972564	0.0052953	0.0164752
**ENSG00000203875**	SNHG5	-0.311040347	6.586033107	8.530698779	0.0052991	0.0164821
**ENSG00000244005**	NFS1	-0.434959344	4.307742371	8.397555136	0.0053090	0.0165082
**ENSG00000054282**	SDCCAG8	-0.286903945	5.42061146	8.391074623	0.0053256	0.0165522
**ENSG00000089006**	SNX5	0.213901481	6.641581794	8.390757209	0.0053264	0.0165522

**ENSG00000138303**	ASCC1	-0.364413491	4.547627294	8.388745494	0.0053315	0.0165626
**ENSG00000118518**	RNF146	-0.28944686	5.173530515	8.388202206	0.0053329	0.0165626
**ENSG00000266049**		0.347337926	4.68441223	8.374469399	0.0053681	0.0166670
**ENSG00000119655**	MIR4709	0.431630475	4.511400583	8.372277968	0.0053737	0.0166796
**ENSG00000077312**	SNRPA	-0.373669702	4.771901039	8.366747383	0.0053880	0.0167189
**ENSG00000116237**	ICMT	0.358857621	4.819166768	8.363909039	0.0053953	0.0167367
**ENSG00000135316**	SYNCRIP	0.169310282	7.898041002	8.361390254	0.0054018	0.0167519
**ENSG00000148019**	CEP78	0.287620558	6.090347106	8.360371854	0.0054045	0.0167551
**ENSG00000237798**		0.334081292	4.675670118	8.356196207	0.0054153	0.0167837
**ENSG00000176095**	IP6K1	0.346528472	4.871034307	8.355398847	0.0054174	0.0167852
**ENSG00000116793**	PHTF1	0.526287091	3.727002297	8.353276292	0.0054229	0.0167933
**ENSG00000138182**	KIF20B	0.323437638	5.660926707	8.353156698	0.0054232	0.0167933
**ENSG00000187653**		0.483133037	6.593025021	8.728057703	0.0054274	0.0168013
**ENSG00000172869**	DMXL1	-0.217174294	6.929554029	8.350348736	0.0054305	0.0168060
**ENSG00000063978**	RNF4	0.199414379	6.758752555	8.348310607	0.0054358	0.0168174
**ENSG00000240972**	MIF	0.622099037	5.288572953	8.696140544	0.0054536	0.0168673
**ENSG00000132879**	FBXO44	-0.504298985	4.28912443	8.334014587	0.0054732	0.0169231
**ENSG00000067057**	PFKP	0.31822752	5.511999807	8.330121068	0.0054835	0.0169497
**ENSG00000150967**	ABCB9	-0.446996284	4.272379337	8.324982556	0.0054970	0.0169865
**ENSG00000151240**	DIP2C	-0.427432431	4.21109213	8.324195084	0.0054991	0.0169879
**ENSG00000187764**	SEMA4D	-0.170973508	8.668859699	8.323549668	0.0055008	0.0169882
**ENSG00000237441**	RGL2	-0.521347848	4.684205487	8.439224948	0.0055084	0.0170066
**ENSG00000008282**	SYPL1	0.291953145	5.85098583	8.315716461	0.0055215	0.0170421
**ENSG00000132326**	PER2	0.363639398	4.90299949	8.308914906	0.0055396	0.0170886
**ENSG00000258704**		-0.548108953	3.636454717	8.308773806	0.0055399	0.0170886
**ENSG00000087365**	SF3B2	0.18237906	7.320241129	8.308189552	0.0055415	0.0170886
**ENSG00000128928**	IVD	-0.372786837	4.621985949	8.306419302	0.0055462	0.0170981
**ENSG00000196776**	CD47	-0.159131161	8.730761061	8.298279125	0.0055679	0.0171600
**ENSG00000104388**	RAB2A	0.24929771	6.211142245	8.293759315	0.0055800	0.0171922
**ENSG00000260007**		-0.26165425	5.502464127	8.289719547	0.0055908	0.0172205
**ENSG00000153879**	CEBPG	0.308936406	5.189827098	8.286560924	0.0055993	0.0172416
**ENSG00000181652**	ATG9B	-0.563551798	3.945993757	8.281540577	0.0056131	0.0172790
**ENSG00000168958**	MFF	-0.217682082	6.057793789	8.279480899	0.0056184	0.0172902
**ENSG00000065413**	ANKRD44	-0.171327024	8.855219392	8.278581631	0.0056208	0.0172909
**ENSG00000177963**	MIR6743	0.271511777	5.28676272	8.278166919	0.0056219	0.0172909
**ENSG00000187109**	NAP1L1	0.214212775	9.76327916	8.266582434	0.0056533	0.0173823
**ENSG00000273088**		0.400567235	4.859146248	8.261168138	0.0056681	0.0174121

**ENSG00000280320**		0.376828278	4.703226248	8.261139863	0.0056681	0.0174121
**ENSG00000211753**		0.559518665	3.751588677	8.260900261	0.0056688	0.0174121
**ENSG00000164010**	ERMAP	-0.577220803	3.498080342	8.260568653	0.0056697	0.0174121
**ENSG00000173530**	TNFRSF10D	-0.409511484	4.335273611	8.245434457	0.0057111	0.0175341
**ENSG00000280248**		0.350658281	4.790682238	8.239174763	0.0057283	0.0175815
**ENSG00000172340**	SUCLG2	-0.225358822	6.061000052	8.238596456	0.0057299	0.0175815
**ENSG00000213516**	RBMXL1	0.286172866	5.664998898	8.235266229	0.0057391	0.0176045
**ENSG00000174437**	ATP2A2	0.186860334	7.043692882	8.226530538	0.0057632	0.0176734
**ENSG00000174695**	TMEM167A	0.215999324	6.161194795	8.222265644	0.0057751	0.0176996
**ENSG00000036549**	ZZZ3	-0.208951308	6.8436158	8.222239291	0.0057751	0.0176996
**ENSG00000111752**	PHC1	-0.245073012	5.870950091	8.218351191	0.0057860	0.0177275
**ENSG00000245205**		0.486923103	3.391126276	8.205278562	0.0058225	0.0178342
**ENSG00000249709**	ZNF564	0.286214736	5.084727128	8.199946395	0.0058374	0.0178707
**ENSG00000009307**	CSDE1	0.182086959	9.291865515	8.199805368	0.0058378	0.0178707
**ENSG00000083099**	LYRM2	0.267838774	5.50169096	8.196074442	0.0058483	0.0178951
**ENSG00000164241**	c5orf63	-0.518835322	4.074823212	8.1954851	0.0058500	0.0178951
**ENSG00000069275**	NUCKS1	0.205195598	7.950460825	8.195153491	0.0058509	0.0178951
**ENSG00000034152**	MAP2K3	0.347833901	4.493703617	8.190980571	0.0058627	0.0179258
**ENSG00000282246**		-0.297294927	5.113973394	8.183767967	0.0058831	0.0179829
**ENSG00000101452**	DHX35	-0.37369872	4.337294555	8.178394223	0.0058983	0.0180242
**ENSG00000030110**	BAK1	0.526112927	3.443839383	8.169302372	0.0059242	0.0180980
**ENSG00000005812**	FBXL3	0.18925701	7.458960894	8.16760629	0.0059290	0.0181075
**ENSG00000172197**	MBOAT1	0.406660472	4.448812134	8.165335438	0.0059355	0.0181221
**ENSG00000029534**	ANK1	0.505176543	4.023815332	8.160166704	0.0059503	0.0181619
**ENSG00000125484**	GTF3C4	0.275598913	5.273587128	8.158696304	0.0059545	0.0181695
**ENSG00000268030**		-0.499817811	3.75396026	8.15786355	0.0059569	0.0181715
**ENSG00000267322**	SNHG22	-0.534647204	7.484637383	8.528945733	0.0059708	0.0182086
**ENSG00000100731**	PCNX1	0.161410828	8.896370062	8.149432436	0.0059812	0.0182306
**ENSG00000153936**	HS2ST1	0.33441461	5.443743616	8.149265565	0.0059817	0.0182306
**ENSG00000166260**	cox11	-0.282963363	5.475048832	8.148705555	0.0059833	0.0182306
**ENSG00000075239**	ACAT1	-0.309688611	5.640511748	8.144812278	0.0059945	0.0182595
**ENSG00000196787**	Hist1h2ag	0.671731075	4.629869765	8.437057402	0.0059998	0.0182705
**ENSG00000015475**	BID	0.343543195	4.446651415	8.141612395	0.0060038	0.0182770
**ENSG00000169180**	XPO6	-0.189919338	7.94878073	8.136902826	0.0060174	0.0183132
**ENSG00000165526**	RPUSD4	-0.382557189	4.517486921	8.131977937	0.0060317	0.0183514
**ENSG00000262302**		0.439092721	4.398194238	8.12877499	0.0060410	0.0183745
**ENSG00000149781**	FERMT3	0.290950725	5.758968075	8.126647953	0.0060472	0.0183880

**ENSG00000183977**	PP2D1	-0.485238392	3.595018108	8.125983083	0.0060492	0.0183885
**ENSG00000119231**	SENP5	0.200519593	6.305296748	8.115392553	0.0060801	0.0184773
**ENSG00000002919**	SNX11	-0.336069652	4.784287025	8.109328684	0.0060979	0.0185260
**ENSG00000186517**	ARHGAP30	0.169001402	7.881540521	8.100047094	0.0061253	0.0186038
**ENSG00000184860**	SDR42E1	-0.436476258	4.372901865	8.086934008	0.0061642	0.0187164
**ENSG00000160908**	ZNF394	-0.283829405	6.015643236	8.084566757	0.0061712	0.0187324
**ENSG00000244625**		-0.530549993	4.767276726	8.258469149	0.0061751	0.0187385
**ENSG00000140983**	RHOT2	-0.609520944	4.965207857	8.374277872	0.0061807	0.0187502
**ENSG00000156110**	ADK	0.356239034	4.949121182	8.076221549	0.0061962	0.0187916
**ENSG00000236088**	COX10-AS1	-0.371714347	4.641021567	8.067432798	0.0062225	0.0188661
**ENSG00000198824**	CHAMP1	0.305808025	5.577704739	8.066332907	0.0062258	0.0188706
**ENSG00000144136**	SLC20A1	0.235391366	6.703174234	8.050466847	0.0062737	0.0190103
**ENSG00000139266**	39873	-0.397964745	4.848165954	8.048365428	0.0062801	0.0190241
**ENSG00000010292**	NCAPD2	0.276102914	6.146353686	8.039484417	0.0063071	0.0191004
**ENSG00000134369**	NAV1	-0.438788474	4.082614065	8.035387109	0.0063196	0.0191327
**ENSG00000137075**	RNF38	0.167475485	7.366490916	8.02778456	0.0063429	0.0191976
**ENSG00000052841**	TTC17	-0.263786884	7.277946554	8.133709487	0.0063481	0.0192077
**ENSG00000136169**	SETDB2	-0.229742496	6.194546409	8.018342379	0.0063719	0.0192742
**ENSG00000272589**	ZSWIM8-AS1	-0.41999212	4.221922847	8.017058856	0.0063758	0.0192806
**ENSG00000068878**	PSME4	0.219506795	7.309011739	8.015826649	0.0063796	0.0192865
**ENSG00000280758**		0.543579286	3.515051763	8.01505467	0.0063820	0.0192882
**ENSG00000257303**		-0.459489325	3.712445142	8.011173784	0.0063940	0.0193188
**ENSG00000133106**	EPSTI1	0.322524151	5.639840386	8.005086848	0.0064128	0.0193702
**ENSG00000273047**		0.5286862	4.408640796	8.077211944	0.0064159	0.0193738
**ENSG00000182866**	LCK	0.197132531	7.567463069	7.993597298	0.0064486	0.0194669
**ENSG00000100281**	HMGXB4	0.351006754	4.816659873	7.99170001	0.0064545	0.0194791
**ENSG00000112855**	HARS2	-0.3682318	5.084087963	7.982576835	0.0064831	0.0195597
**ENSG00000162384**	c1orf123	-0.381601271	4.652136273	7.981812805	0.0064855	0.0195613
**ENSG00000184402**	SS18L1	-0.440533142	4.410749393	7.979812262	0.0064917	0.0195746
**ENSG00000196233**	C10orf12	0.185710943	7.497431542	7.97729502	0.0064997	0.0195928
**ENSG00000184381**	PLA2G6	-0.530711331	4.591817537	8.093854314	0.0065116	0.0196232
**ENSG00000274425**		0.271398398	6.008068656	7.961630188	0.0065491	0.0197233
**ENSG00000151366**	NDUFC2	0.435604381	4.490038171	7.961469066	0.0065496	0.0197233
**ENSG00000197969**	VPS13A	-0.210440467	7.873737087	7.961193856	0.0065505	0.0197233
**ENSG00000139793**	MBNL2	0.209416946	6.351166135	7.958431179	0.0065593	0.0197441
**ENSG00000238227**	c9orf69	0.452038398	4.087297163	7.95428322	0.0065725	0.0197781
**ENSG00000273151**		-0.626241904	3.870405949	8.045057715	0.0065826	0.0198029

**ENSG00000107779**	BMPR1A	0.338637177	4.69380558	7.9504664	0.0065846	0.0198033
**ENSG00000172428**	COPS9	0.599738233	3.686211516	7.97598417	0.0065926	0.0198215
**ENSG00000141298**	SSH2	0.169206841	7.711065688	7.945532705	0.0066004	0.0198392
**ENSG00000101745**	ANKRD12	0.185780872	9.210279836	7.940578036	0.0066163	0.0198812
**ENSG00000172273**	HINFP	-0.335366264	4.817258523	7.938025003	0.0066244	0.0199001
**ENSG00000101361**	MIR1292	-0.233803141	6.184714298	7.936539699	0.0066292	0.0199064
**ENSG00000171206**	TRIM8	0.357558319	4.9287087	7.936181763	0.0066304	0.0199064
**ENSG00000172932**	ANKRD13D	-0.28690655	5.925066489	7.931627391	0.0066450	0.0199399
**ENSG00000178927**	c17orf62	-0.264155266	6.050840661	7.931526987	0.0066453	0.0199399
**ENSG00000179295**	PTPN11	0.198764069	6.821825762	7.921587161	0.0066774	0.0200304
**ENSG00000273221**		-0.326065163	5.617074568	7.908311768	0.0067208	0.0201549
**ENSG00000143198**	MGST3	0.285565743	5.22247534	7.903536439	0.0067361	0.0201949
**ENSG00000196968**	FUT11	0.373044443	4.935144868	7.902497424	0.0067395	0.0201993
**ENSG00000160714**	UBE2Q1	0.257726239	6.076752969	7.901431633	0.0067430	0.0202040
**ENSG00000171827**	ZNF570	0.356309593	4.59158902	7.900157553	0.0067472	0.0202050
**ENSG00000136935**	GOLGA1	-0.292848758	5.746729096	7.900141039	0.0067472	0.0202050
**ENSG00000176105**	YES1	-0.445899853	3.734311761	7.897930067	0.0067544	0.0202209
**ENSG00000184574**	LPAR5	0.492229089	3.929585505	7.894186773	0.0067667	0.0202519
**ENSG00000173039**	RELA	0.242772247	6.018131997	7.885501979	0.0067953	0.0203315
**ENSG00000178921**	PFAS	-0.518705789	4.075629917	7.878359042	0.0068189	0.0203963
**ENSG00000166887**	VPS39	-0.235146028	6.336830895	7.871867393	0.0068404	0.0204548
**ENSG00000173588**	cep83	-0.343824533	4.969655236	7.870763571	0.0068441	0.0204599
**ENSG00000133985**	TTC9	-0.288858164	5.406874387	7.866865935	0.0068570	0.0204928
**ENSG00000105829**	BET1	-0.421441906	4.294423432	7.864236937	0.0068658	0.0205131
**ENSG00000113569**	NUP155	0.205931841	6.424066124	7.857006404	0.0068899	0.0205793
**ENSG00000183060**	LYSMD4	-0.405176809	4.383431594	7.848617998	0.0069180	0.0206574
**ENSG00000105865**	DUS4L	-0.53855862	3.738096893	7.847976641	0.0069202	0.0206579
**ENSG00000005844**	ITGAL	0.176348772	8.449384638	7.844259081	0.0069327	0.0206894
**ENSG00000161057**	PSMC2	0.264508709	5.649616768	7.842285046	0.0069393	0.0207033
**ENSG00000132467**	UTP3	0.307262339	5.162771445	7.838212895	0.0069531	0.0207384
**ENSG00000272410**	TATDN2	0.218622743	6.054504121	7.837228843	0.0069564	0.0207424
**ENSG00000107371**	EXOSC3	-0.289154871	5.274833882	7.836582894	0.0069586	0.0207430
**ENSG00000108666**	c17orf75	-0.455299496	3.933803928	7.835109955	0.0069636	0.0207519
**ENSG00000176623**	RMDN1	-0.231628269	6.338432581	7.831710372	0.0069751	0.0207803
**ENSG00000231999**	FLJ27354	-0.447844789	4.144230384	7.824137317	0.0070008	0.0208509
**ENSG00000268858**		-0.581990134	3.587989507	7.823527761	0.0070029	0.0208512
**ENSG00000128271**	ADORA2A	0.391093376	4.738803758	7.798654143	0.0070880	0.0210988

**ENSG00000073910**	FRY	-0.315642869	5.840041527	7.820629991	0.0070933	0.0211085
**ENSG00000261824**	LINC00662	-0.449014572	4.427439623	7.785180953	0.0071346	0.0212254
**ENSG00000214265**	SNURF	-0.204655985	6.303485483	7.778925969	0.0071564	0.0212840
**ENSG00000131626**	ppfia1	-0.231112312	6.445086054	7.777749508	0.0071605	0.0212868
**ENSG00000267046**		0.605906206	3.688197035	7.813869044	0.0071614	0.0212868
**ENSG00000135899**	SP110	-0.190538418	7.315254201	7.75905944	0.0072259	0.0214709
**ENSG00000136783**	NIPSNAP3A	-0.539968328	3.574154683	7.758624424	0.0072274	0.0214709
**ENSG00000079739**	PGM1	0.37159985	4.306537873	7.748395321	0.0072635	0.0215719
**ENSG00000111196**	MAGOHB	-0.453937186	4.485062784	7.745940854	0.0072722	0.0215886
**ENSG00000224186**		-0.349196845	5.398001917	7.750325267	0.0072732	0.0215886
**ENSG00000126945**	HNRNPH2	0.251656128	6.00658215	7.738837666	0.0072974	0.0216541
**ENSG00000148290**	SURF1	-0.381523397	3.814210202	7.737542109	0.0073020	0.0216616
**ENSG00000284634**		0.216874164	6.910169068	7.73665187	0.0073052	0.0216649
**ENSG00000119397**	CNTRL	-0.189628513	8.359068519	7.735973152	0.0073076	0.0216659
**ENSG00000123200**	ZC3H13	0.221143292	7.807801906	7.766827179	0.0073142	0.0216795
**ENSG00000160185**	UBASH3A	-0.223510696	6.673452487	7.732414465	0.0073202	0.0216912
**ENSG00000147123**	NDUFB11	0.390408258	4.532801062	7.727588336	0.0073375	0.0217361
**ENSG00000180879**	SSR4	0.384914202	5.499443677	7.82110715	0.0073612	0.0218003
**ENSG00000092470**	WDR76	0.350862262	4.756180377	7.718537322	0.0073699	0.0218170
**ENSG00000164308**	ERAP2	-0.261337133	7.817366201	7.869827937	0.0073711	0.0218170
**ENSG00000214022**	REPIN1	-0.306830142	4.732117366	7.717625454	0.0073732	0.0218171
**ENSG00000250264**		-0.307468075	5.275046337	7.71662188	0.0073768	0.0218216
**ENSG00000164211**	STARD4	0.306501053	6.137839259	7.776684969	0.0073921	0.0218608
**ENSG00000115274**	INO80B	-0.47189988	3.458290185	7.711625063	0.0073948	0.0218624
**ENSG00000165678**	GHITM	0.239806558	6.55303829	7.710990424	0.0073971	0.0218630
**ENSG00000252010**	SCARNA5	0.933660039	6.972975554	8.046676807	0.0074092	0.0218928
**ENSG00000189362**	NEMP2	-0.290278514	5.762026425	7.704862932	0.0074192	0.0219160
**ENSG00000076043**	REXO2	-0.31428772	5.169308752	7.702836159	0.0074265	0.0219315
**ENSG00000198689**	SLC9A6	0.280030881	5.591068573	7.700473014	0.0074351	0.0219506
**ENSG00000178567**	EPM2AIP1	-0.222097892	6.678755602	7.697218041	0.0074469	0.0219792
**ENSG00000171960**	PPIH	-0.451811005	3.968248027	7.687082488	0.0074838	0.0220819
**ENSG00000118363**	SPCS2	0.31625776	5.086294193	7.683219785	0.0074979	0.0221173
**ENSG00000154305**	MIA3	-0.179575464	7.590058913	7.682150709	0.0075018	0.0221225
**ENSG00000093100**		0.521888077	3.730724386	7.679950596	0.0075098	0.0221401
**ENSG00000105655**	ISYNA1	0.53517884	3.851906644	7.677580132	0.0075185	0.0221591
**ENSG00000172167**	MTBP	-0.405900301	4.070202072	7.677027786	0.0075206	0.0221591
**ENSG00000270277**		-0.558328278	3.507651349	7.674880413	0.0075284	0.0221761

**ENSG00000177156**	TALDO1	0.325528023	5.214148432	7.673222975	0.0075345	0.0221878
**ENSG00000137055**	PLAA	0.272808975	5.298958365	7.670613189	0.0075441	0.0222098
**ENSG00000111875**	ASF1A	0.239402645	6.221361308	7.66582324	0.0075618	0.0222555
**ENSG00000132471**	WBP2	0.263286781	5.979074798	7.664256595	0.0075676	0.0222663
**ENSG00000073849**	st6gal1	0.173250284	7.886077853	7.663477527	0.0075704	0.0222685
**ENSG00000101158**	NELFCD	-0.251360593	5.844108415	7.657840422	0.0075913	0.0223235
**ENSG00000122257**	RBBP6	-0.207971041	7.590790284	7.650113201	0.0076200	0.0223965
**ENSG00000061938**	TNK2	-0.404475137	5.497973545	7.787115428	0.0076204	0.0223965
**ENSG00000136877**	FPGS	-0.439208895	4.186412483	7.641261145	0.0076530	0.0224825
**ENSG00000175662**	TOM1L2	-0.346164227	5.2187705	7.641000475	0.0076539	0.0224825
**ENSG00000049239**	H6PD	0.275701864	5.851678583	7.637670418	0.0076664	0.0225127
**ENSG00000174799**	CEP135	-0.261931083	6.033633746	7.633541315	0.0076819	0.0225518
**ENSG00000172780**	RAB43	-0.479958645	3.719233917	7.629826603	0.0076958	0.0225864
**ENSG00000131931**	THAP1	0.291891628	4.873149098	7.623565833	0.0077194	0.0226493
**ENSG00000235453**	TOPORS-AS1	-0.50944797	3.431094028	7.618596938	0.0077381	0.0226979
**ENSG00000163605**	PPP4R2	0.178892235	6.856197104	7.617958599	0.0077406	0.0226986
**ENSG00000004766**	VPS50	-0.25636345	5.983661289	7.61456842	0.0077534	0.0227299
**ENSG00000162413**	KLHL21	0.259551622	5.191448856	7.613406741	0.0077578	0.0227364
**ENSG00000113575**	PPP2CA	0.177802046	6.839707239	7.605569811	0.0077876	0.0228173
**ENSG00000136682**	CBWD2	-0.345879646	4.55579332	7.602507602	0.0077992	0.0228401
**ENSG00000048028**	USP28	0.303348468	4.830913281	7.602138905	0.0078006	0.0228401
**ENSG00000101940**	WDR13	-0.47583282	3.75857207	7.601803587	0.0078019	0.0228401
**ENSG00000140992**	PDPK1	0.212861831	5.839501641	7.60077964	0.0078058	0.0228451
**ENSG00000113269**	RNF130	-0.51795203	3.467895739	7.591765961	0.0078403	0.0229387
**ENSG00000204569**	PPP1R10	0.213827427	6.602000156	7.590986297	0.0078433	0.0229387
**ENSG00000170296**	GABARAP	0.33766063	5.812427609	7.667944252	0.0078443	0.0229387
**ENSG00000106524**	ANKMY2	0.378188596	4.192331443	7.58968002	0.0078483	0.0229437
**ENSG00000127831**	VIL1	0.421184812	4.37494412	7.585618834	0.0078639	0.0229829
**ENSG00000164961**	KIAA0196	0.196395873	6.141359124	7.583645482	0.0078715	0.0229987
**ENSG00000142188**	TMEM50B	-0.212025684	6.14045923	7.582723428	0.0078750	0.0230026
**ENSG00000163913**	IFT122	-0.49659635	3.851169684	7.573113314	0.0079121	0.0231045
**ENSG00000136026**	CKAP4	-0.525495836	3.681876402	7.563810965	0.0079482	0.0232034
**ENSG00000043093**	DCUN1D1	0.228255062	6.03008744	7.561979939	0.0079553	0.0232076
**ENSG00000145916**	RMND5B	-0.292715104	5.373436928	7.561872197	0.0079557	0.0232076
**ENSG00000164896**	FASTK	-0.493811537	4.597300471	7.642017084	0.0079563	0.0232076
**ENSG00000251357**		0.462971051	4.764790022	7.626982944	0.0079659	0.0232291
**ENSG00000259205**		-0.50788406	4.098998718	7.554915988	0.0079829	0.0232721

**ENSG00000110315**	RNF141	-0.280492644	5.513489859	7.552895932	0.0079908	0.0232841
**ENSG00000132478**	UNK	-0.276323653	5.324559607	7.552728219	0.0079914	0.0232841
**ENSG00000143862**	ARL8A	0.465251064	4.094808038	7.551715655	0.0079954	0.0232891
**ENSG00000068305**	MEF2A	-0.217053542	6.099190405	7.540111124	0.0080409	0.0234152
**ENSG00000188994**	ZNF292	0.19241656	8.145347693	7.538945836	0.0080455	0.0234221
**ENSG00000170043**	TRAPPC1	0.33210228	4.590027289	7.537703006	0.0080504	0.0234298
**ENSG00000169032**	MAP2K1	0.248437896	5.992014325	7.535017147	0.0080610	0.0234541
**ENSG00000176049**	JAKMIP2	0.523388366	4.253041283	7.568512835	0.0080743	0.0234826
**ENSG00000183401**	CCDC159	-0.538018976	3.507117281	7.531398461	0.0080753	0.0234826
**ENSG00000182117**	NOP10	0.469328064	4.702336882	7.608721084	0.0080851	0.0235008
**ENSG00000136816**	TOR1B	0.321977257	4.661063674	7.528683546	0.0080860	0.0235008
**ENSG00000100241**	SBF1	0.265035043	7.070663967	7.629409433	0.0080907	0.0235078
**ENSG00000101084**	c20orf24	0.329352496	4.649640926	7.523389082	0.0081070	0.0235487
**ENSG00000123353**	ORMDL2	0.347402245	4.596680629	7.52258015	0.0081102	0.0235498
**ENSG00000100239**	PPP6R2	-0.30314571	6.050091278	7.572106409	0.0081119	0.0235498
**ENSG00000165704**	HPRT1	0.46917919	4.172710096	7.517601228	0.0081300	0.0235959
**ENSG00000126351**	THRA	-0.317118339	5.389998105	7.516471829	0.0081345	0.0235976
**ENSG00000198663**	C6orf89	0.201347702	6.298014623	7.51620776	0.0081356	0.0235976
**ENSG00000072609**	CHFR	-0.265569863	5.916364639	7.515754488	0.0081374	0.0235976
**ENSG00000167977**	KCTD5	0.442407261	3.776237439	7.513458308	0.0081465	0.0236176
**ENSG00000198677**	TTC37	-0.163677365	7.634519836	7.497295573	0.0082113	0.0237988
**ENSG00000123130**	ACOT9	0.373790293	4.446941958	7.490718062	0.0082378	0.0238690
**ENSG00000122507**	BBS9	-0.294496934	5.102203825	7.488860333	0.0082453	0.0238784
**ENSG00000100242**	SUN2	0.184607425	8.341329135	7.48878869	0.0082456	0.0238784
**ENSG00000134686**	MIR3605	0.399182132	4.238858056	7.487853906	0.0082494	0.0238827
**ENSG00000112592**	TBP	-0.310873014	5.127704308	7.485765867	0.0082579	0.0239006
**ENSG00000204592**	HLA-E	0.16524561	9.917961915	7.484101755	0.0082646	0.0239134
**ENSG00000130714**	POMT1	-0.486314668	4.887651043	7.621351501	0.0082683	0.0239175
**ENSG00000120860**	CCDC53	-0.293134082	5.170631842	7.481923678	0.0082734	0.0239246
**ENSG00000131378**	RFTN1	-0.221938677	6.822484287	7.481456898	0.0082753	0.0239246
**ENSG00000215695**	RSC1A1	0.294481265	4.76307426	7.471048729	0.0083177	0.0240404
**ENSG00000138185**	ENTPD1	0.561191874	3.67025067	7.470319155	0.0083206	0.0240423
**ENSG00000105221**	AKT2	-0.220458367	6.123011037	7.468981686	0.0083261	0.0240515
**ENSG00000170915**	PAQR8	-0.250152868	6.048305576	7.466892054	0.0083346	0.0240695
**ENSG00000110713**	NUP98	0.151746087	7.602822565	7.464623581	0.0083439	0.0240896
**ENSG00000137700**	SLC37A4	-0.617613346	3.559599073	7.502669052	0.0083540	0.0241121
**ENSG00000196684**	HSH2D	-0.533854458	3.872391113	7.447147056	0.0084158	0.0242837

**ENSG00000117625**	RCOR3	-0.213496327	6.710398149	7.443578926	0.0084305	0.0243195
**ENSG00000246451**		-0.426955744	4.301421193	7.440459123	0.0084434	0.0243501
**ENSG00000260916**	MIR628	0.221312687	6.066992166	7.435189124	0.0084653	0.0244064
**ENSG00000102967**	DHODH	-0.483247416	3.936773787	7.433224988	0.0084735	0.0244202
**ENSG00000128016**	ZFP36	0.293513669	6.919088663	7.585367404	0.0084763	0.0244202
**ENSG00000102317**	RBM3	-0.1723392	7.176123661	7.432350371	0.0084771	0.0244202
**ENSG00000165138**	ANKS6	-0.511415672	3.704479144	7.42997316	0.0084870	0.0244420
**ENSG00000130475**	FCHO1	-0.287053933	5.40757251	7.427657001	0.0084967	0.0244631
**ENSG00000083896**	YTHDC1	-0.15657469	7.906092965	7.426734399	0.0085005	0.0244674
**ENSG00000140463**	BBS4	-0.478469702	3.985764396	7.421869727	0.0085208	0.0245192
**ENSG00000059122**	FLYWCH1	-0.456166674	4.960957052	7.530066421	0.0085317	0.0245438
**ENSG00000110048**	OSBP	0.217744244	6.215899831	7.413565738	0.0085557	0.0246059
**ENSG00000139055**	erp27	-0.379568863	5.570628961	7.522480537	0.0085637	0.0246221
**ENSG00000013563**	DNASE1L1	-0.464170287	4.124048898	7.406746465	0.0085844	0.0246748
**ENSG00000100139**	MICALL1	0.415106161	4.121548648	7.405170985	0.0085910	0.0246872
**ENSG00000160216**	AGPAT3	0.196802072	6.104248849	7.401384076	0.0086070	0.0247263
**ENSG00000011258**	MBTD1	-0.265972266	5.906734362	7.398925834	0.0086174	0.0247494
**ENSG00000246334**		-0.398039788	4.467062784	7.398156694	0.0086207	0.0247516
**ENSG00000100413**	POLR3H	-0.25856576	5.315056562	7.397594508	0.0086231	0.0247516
**ENSG00000211786**		0.420119779	4.274899382	7.396705608	0.0086268	0.0247516
**ENSG00000185716**	c16orf52	-0.363751909	4.819369631	7.396515989	0.0086276	0.0247516
**ENSG00000204348**	DXO	-0.505740826	3.464461836	7.39403052	0.0086382	0.0247750
**ENSG00000136938**	ANP32B	0.249115288	7.786719035	7.515274683	0.0086495	0.0248006
**ENSG00000253719**	ATXN7L3B	-0.183665082	6.815196405	7.3898466	0.0086560	0.0248124
**ENSG00000166783**	MIR6506	0.139099523	8.057388589	7.388717686	0.0086608	0.0248194
**ENSG00000100889**	PCK2	0.532038196	3.599293112	7.38651019	0.0086702	0.0248395
**ENSG00000267697**	LUZP6	0.348747993	4.304758425	7.385301643	0.0086753	0.0248475
**ENSG00000204152**	TIMM23B	-0.461849738	3.933513612	7.375559678	0.0087170	0.0249599
**ENSG00000116337**	AMPD2	-0.356771559	5.006385526	7.372085135	0.0087319	0.0249932
**ENSG00000267680**	ZNF224	-0.256229138	6.132882659	7.371736205	0.0087334	0.0249932
**ENSG00000175354**	PTPN2	0.200647738	6.401078407	7.370740747	0.0087376	0.0249986
**ENSG00000122729**	ACO1	-0.335722068	5.089180244	7.365930725	0.0087583	0.0250505
**ENSG00000206149**	HERC2P9	-0.470054546	4.074052617	7.365406392	0.0087606	0.0250505
**ENSG00000105443**	CYTH2	-0.334079983	5.000966747	7.363102698	0.0087705	0.0250721
**ENSG00000155508**	CNOT8	0.17975129	6.757485094	7.359232359	0.0087872	0.0251130
**ENSG00000083312**	TNPO1	0.154295949	7.824153579	7.351870258	0.0088191	0.0251972
**ENSG00000261460**		-0.396251231	4.034839477	7.35089768	0.0088233	0.0252024

**ENSG00000159176**	CSRP1	0.317851231	4.704853557	7.349366675	0.0088300	0.0252145
**ENSG00000196419**	XRCC6	0.193783616	6.867007584	7.34675875	0.0088413	0.0252400
**ENSG00000115109**	EPB41L5	-0.473117502	3.81715715	7.344038611	0.0088532	0.0252669
**ENSG00000243749**	Tmem35b	0.332894753	4.40972757	7.341836891	0.0088628	0.0252874
**ENSG00000101079**	NDRG3	0.275699841	5.546830732	7.339429415	0.0088733	0.0253105
**ENSG00000105993**	DNAJB6	-0.210851509	6.519139155	7.3383989	0.0088778	0.0253164
**ENSG00000140675**	SLC5A2	-0.395136085	4.373072057	7.336783887	0.0088848	0.0253296
**ENSG00000078177**	N4BP2	-0.221468884	6.885539172	7.335330735	0.0088912	0.0253408
**ENSG00000112584**	FAM120B	-0.215016816	6.299719515	7.318273313	0.0089662	0.0255476
**ENSG00000146909**	NOM1	-0.279970948	5.484527465	7.307564065	0.0090136	0.0256757
**ENSG00000130175**	PRKCSH	0.20162552	6.678090661	7.304968957	0.0090251	0.0257015
**ENSG00000143321**	HDGF	0.287368374	5.573969778	7.303437615	0.0090319	0.0257139
**ENSG00000060069**	CTDP1	0.293868876	4.967089218	7.298361842	0.0090545	0.0257644
**ENSG00000239002**	Scarna10	0.855721646	6.614003127	7.604576349	0.0090546	0.0257644
**ENSG00000276232**		0.85619364	6.613945174	7.60142064	0.0090676	0.0257945
**ENSG00000138802**	SEC24B	0.174307564	7.2298959	7.293935511	0.0090743	0.0258065
**ENSG00000204237**	oxld1	-0.504214729	3.592923717	7.291414519	0.0090856	0.0258315
**ENSG00000197903**	Hist1h2bk	0.590280595	5.357666736	7.57688367	0.0090908	0.0258394
**ENSG00000136111**	TBC1D4	-0.35155391	7.606896042	7.56728958	0.0091061	0.0258757
**ENSG00000163848**	ZNF148	0.196223449	7.306226363	7.284245987	0.0091177	0.0259018
**ENSG00000103126**	AXIN1	0.267422385	5.119704848	7.283198484	0.0091225	0.0259082
**ENSG00000104343**	UBE2W	0.258728929	5.780596172	7.275929697	0.0091552	0.0259941
**ENSG00000279332**		0.409429411	4.236782127	7.270857595	0.0091781	0.0260521
**ENSG00000140750**	ARHGAP17	-0.215063416	5.987190815	7.256592481	0.0092429	0.0262289
**ENSG00000113749**	HRH2	-0.437760186	4.258339581	7.24981324	0.0092739	0.0263096
**ENSG00000214753**	HNRNPUL2	0.193353949	7.503749944	7.24658688	0.0092887	0.0263444
**ENSG00000177337**		0.41785497	3.81196884	7.240691815	0.0093157	0.0264140
**ENSG00000168118**	RAB4A	0.338734509	4.822410772	7.238470886	0.0093259	0.0264293
**ENSG00000198783**	ZNF830	-0.302036315	4.936058543	7.238420949	0.0093262	0.0264293
**ENSG00000213672**	nckipsd	-0.453244983	3.673367196	7.229075948	0.0093693	0.0265443
**ENSG00000182287**	AP1S2	0.289867476	5.657500184	7.225577786	0.0093855	0.0265829
**ENSG00000116120**	FARSB	-0.246327622	5.50091293	7.222776673	0.0093985	0.0266125
**ENSG00000148154**	UGCG	0.256658619	6.013775943	7.217969142	0.0094208	0.0266593
**ENSG00000107897**	ACBD5	0.251612006	5.338378064	7.217942689	0.0094209	0.0266593
**ENSG00000150477**	KIAA1328	-0.39062491	4.579773967	7.2175784	0.0094226	0.0266593
**ENSG00000139324**	TMTC3	0.301211537	5.205608809	7.212120235	0.0094480	0.0267240
**ENSG00000159733**	ZFYVE28	0.372505693	4.656900101	7.210949875	0.0094535	0.0267314

**ENSG00000174652**	ZNF266	-0.300415518	6.195346988	7.289063907	0.0094558	0.0267314
**ENSG00000132254**	ARFIP2	-0.418011854	4.138394007	7.205966154	0.0094768	0.0267836
**ENSG00000197258**		0.436689972	4.364072072	7.205030633	0.0094812	0.0267887
**ENSG00000133997**	MED6	-0.261332788	5.425470115	7.199004713	0.0095095	0.0268614
**ENSG00000259952**		0.35301398	4.531852086	7.196503994	0.0095212	0.0268802
**ENSG00000175305**	CCNE2	-0.402062555	4.579778707	7.19648986	0.0095213	0.0268802
**ENSG00000174606**	ANGEL2	-0.224939952	6.426309238	7.193816086	0.0095339	0.0269085
**ENSG00000284461**		0.245345197	5.319369405	7.193055594	0.0095374	0.0269105
**ENSG00000114316**	USP4	-0.200999167	6.515475741	7.19256979	0.0095397	0.0269105
**ENSG00000163807**	KIAA1143	0.243112894	5.590089445	7.191501935	0.0095448	0.0269129
**ENSG00000104154**	SLC30A4	-0.445403951	3.916054552	7.1913022	0.0095457	0.0269129
**ENSG00000135002**	RFK	0.362594226	4.361868949	7.189301775	0.0095552	0.0269322
**ENSG00000279662**		-0.529839963	3.595631031	7.186878898	0.0095666	0.0269572
**ENSG00000131437**	KIF3A	0.252269161	5.522844491	7.184666602	0.0095771	0.0269795
**ENSG00000113615**	sec24a	0.228511277	6.186435086	7.182780299	0.0095860	0.0269974
**ENSG00000169764**	UGP2	0.182369064	6.9381529	7.182219961	0.0095887	0.0269976
**ENSG00000107959**	PITRM1	-0.218498606	6.53890851	7.18105978	0.0095942	0.0270058
**ENSG00000131507**	NDFIP1	-0.194283314	7.211214645	7.179416645	0.0096020	0.0270204
**ENSG00000010810**	FYN	0.159708163	8.484327743	7.177760593	0.0096098	0.0270349
**ENSG00000089351**	GRAMD1A	-0.2647465	6.405604139	7.201153204	0.0096122	0.0270349
**ENSG00000101849**	TBL1X	0.267385843	5.808112273	7.175390259	0.0096211	0.0270506
**ENSG00000234857**	HNRNPUL2-BSC	0.203815549	7.156547405	7.174714945	0.0096243	0.0270506
**ENSG00000101193**	GID8	0.224611748	5.883616569	7.174439456	0.0096256	0.0270506
**ENSG00000100196**	KDELR3	-0.356833156	4.892014721	7.163847742	0.0096762	0.0271853
**ENSG00000112763**	btn2a1	-0.290273477	5.67408289	7.155412143	0.0097166	0.0272917
**ENSG00000115484**	CCT4	0.203444631	6.90921089	7.154302547	0.0097219	0.0272993
**ENSG00000008283**	CYB561	0.409596559	4.449980573	7.15002337	0.0097425	0.0273498
**ENSG00000170584**	NUDCD2	-0.245973844	5.28960384	7.146855588	0.0097578	0.0273854
**ENSG00000186704**		-0.503553812	4.014799345	7.143087872	0.0097760	0.0274241
**ENSG00000115904**	SOS 1	0.171456648	8.010546222	7.142914698	0.0097769	0.0274241
**ENSG00000139505**	MTMR6	0.221603892	6.204908417	7.138625868	0.0097976	0.0274750
**ENSG00000013561**	RNF14	0.290066597	5.115939489	7.133734775	0.0098214	0.0275270
**ENSG00000154813**	DPH3	0.350677324	4.554974019	7.133425204	0.0098229	0.0275270
**ENSG00000120458**	MSANTD2	-0.397006956	4.443919184	7.133178752	0.0098241	0.0275270
**ENSG00000165121**		-0.416338209	4.115362482	7.131382161	0.0098328	0.0275441
**ENSG00000271741**		-0.21767437	6.03983069	7.12851311	0.0098468	0.0275759
**ENSG00000109519**	GRPEL1	0.304636168	4.885454112	7.127802741	0.0098503	0.0275782

**ENSG00000275307**		-0.36024887	4.435230433	7.124720796	0.0098653	0.0276129
**ENSG00000204387**	C6orf48	-0.353096164	5.29135366	7.147951499	0.0098688	0.0276152
**ENSG00000254452**		0.395097702	4.010237262	7.119313885	0.0098917	0.0276721
**ENSG00000170836**	PPM1D	0.354219391	4.26668046	7.118385347	0.0098963	0.0276774
**ENSG00000136827**	TOR1A	0.276859487	4.784318548	7.11599584	0.0099080	0.0277028
**ENSG00000187713**	TMEM203	0.401137162	4.124799659	7.114150947	0.0099171	0.0277207
**ENSG00000284691**		-0.416753838	4.455808659	7.108792594	0.0099434	0.0277869
**ENSG00000133111**	rfxap	-0.414569758	3.810402262	7.108251166	0.0099461	0.0277870
**ENSG00000156304**	SCAF4	-0.177512863	7.012580055	7.105583339	0.0099592	0.0278163
**ENSG00000102158**	MAGT1	0.223244339	6.124038477	7.102722364	0.0099734	0.0278483
**ENSG00000196074**	SYCP2	-0.436125067	4.041557534	7.099844413	0.0099876	0.0278806
**ENSG00000206760**	SNORA6	0.525218624	3.785551018	7.097720939	0.0099981	0.0279025
**ENSG00000168872**	DDX19A	-0.242334052	5.561140473	7.089611397	0.0100384	0.0280073
**ENSG00000253607**		0.431472335	3.40724408	7.088482678	0.0100440	0.0280125
**ENSG00000083799**	CYLD	-0.15048071	9.25444567	7.088160712	0.0100456	0.0280125
**ENSG00000100580**	TMED8	0.274147349	5.10946516	7.083802469	0.0100673	0.0280656
**ENSG00000108639**	SYNGR2	0.331953188	4.400603019	7.077292979	0.0100998	0.0281488
**ENSG00000008405**	CRY1	-0.294115747	4.887475637	7.074384502	0.0101144	0.0281819
**ENSG00000128513**	POT1	-0.30799886	5.052002149	7.072857925	0.0101221	0.0281957
**ENSG00000035403**	VCL	0.235901101	6.642610289	7.069633379	0.0101383	0.0282333
**ENSG00000213793**		0.444649085	4.006986982	7.067888753	0.0101470	0.0282502
**ENSG00000219481**	NBPF1	-0.371827028	4.247444204	7.065157713	0.0101608	0.0282756
**ENSG00000126067**	PSMB2	0.223220462	5.756556553	7.065007768	0.0101615	0.0282756
**ENSG00000027847**	B4GALT7	-0.440817454	4.027302927	7.062986845	0.0101717	0.0282904
**ENSG00000263020**	LY6G5B	-0.219306453	5.990831752	7.062637645	0.0101735	0.0282904
**ENSG00000198286**	CARD11	-0.160316173	7.032440926	7.062343854	0.0101750	0.0282904
**ENSG00000087301**	TXNDC16	-0.338148338	4.556465945	7.059329064	0.0101902	0.0283252
**ENSG00000071127**	WDR1	0.150011542	7.626984044	7.054629988	0.0102140	0.0283837
**ENSG00000110906**	KCTD10	0.283923598	5.334617324	7.049063792	0.0102422	0.0284546
**ENSG00000118420**	UBE3D	-0.537917887	3.77643671	7.045483704	0.0102604	0.0284945
**ENSG00000168067**	MAP4K2	-0.246385775	6.130791586	7.045171596	0.0102620	0.0284945
**ENSG00000258830**		-0.246165548	5.739988525	7.042626096	0.0102750	0.0285229
**ENSG00000162642**	C1orf52	-0.300745192	4.941985471	7.039172774	0.0102926	0.0285643
**ENSG00000276664**		-0.39299707	4.012683218	7.038016214	0.0102985	0.0285731
**ENSG00000249249**		0.377429795	3.830571632	7.034182544	0.0103181	0.0286083
**ENSG00000228409**		-0.426084121	4.43246149	7.034146598	0.0103183	0.0286083
**ENSG00000087303**	NID2	-0.629010263	3.519282513	7.091416398	0.0103194	0.0286083

**ENSG00000021574**	SPAST	0.257686524	5.698778156	7.031887695	0.0103299	0.0286297
**ENSG00000067596**	DHX8	0.214215914	6.246729056	7.030855565	0.0103352	0.0286368
**ENSG00000256594**	LOC374443	-0.227336397	6.674320036	7.025140453	0.0103645	0.0287106
**ENSG00000101546**	RBFA	-0.484297719	3.618599397	7.022764522	0.0103768	0.0287368
**ENSG00000137776**	SLTM	-0.168718449	7.871609581	7.021801014	0.0103817	0.0287411
**ENSG00000125656**	CLPP	0.344716837	4.615588928	7.02139679	0.0103838	0.0287411
**ENSG00000135457**	tfcp2	0.234251292	5.462003658	7.019249082	0.0103949	0.0287642
**ENSG00000250565**	ATP6V1E2	-0.445404189	3.725775913	7.016674941	0.0104082	0.0287934
**ENSG00000044090**	CUL7	-0.383815253	4.279513921	7.015435694	0.0104146	0.0288035
**ENSG00000113300**	CNOT6	0.205210979	6.526852123	7.009110343	0.0104474	0.0288864
**ENSG00000179262**	RAD23A	0.241206184	5.50857949	7.005564572	0.0104658	0.0289260
**ENSG00000214922**	HLA-F-AS1	-0.342694287	4.959434064	7.005289198	0.0104672	0.0289260
**ENSG00000110844**	PRPF40B	-0.327519212	4.889651655	7.002659888	0.0104809	0.0289508
**ENSG00000262560**		-0.401021442	3.870211184	7.002503638	0.0104817	0.0289508
**ENSG00000259185**		0.347811941	4.456000148	7.001559913	0.0104866	0.0289566
**ENSG00000196465**	MYL6B	0.293514877	5.238670079	7.001035641	0.0104894	0.0289566
**ENSG00000142186**	SCYL1	-0.262093435	5.072194542	6.995977743	0.0105158	0.0290218
**ENSG00000242338**		-0.520742592	3.8913215	6.994873081	0.0105215	0.0290301
**ENSG00000100528**	CNIH1	0.249720899	5.688965838	6.982990582	0.0105838	0.0291943
**ENSG00000160991**	ORAI2	0.346080334	4.812724215	6.98212536	0.0105884	0.0291991
**ENSG00000236552**	RPL13AP5	0.425188187	7.885073117	7.25274828	0.0106338	0.0293167
**ENSG00000183323**	CCDC125	-0.449215465	3.963000809	6.971690575	0.0106435	0.0293355
**ENSG00000281376**	ABALON	0.434095478	3.669002949	6.960074267	0.0107051	0.0294977
**ENSG00000048392**	RRM2B	0.333828673	4.63832564	6.958108274	0.0107156	0.0295188
**ENSG00000179029**	TMEM107	0.580080629	4.309192678	7.099176261	0.0107255	0.0295382
**ENSG00000206344**	HCG27	-0.459173984	4.190217141	6.955662902	0.0107286	0.0295391
**ENSG00000133943**	c14orf159	-0.282312638	5.461554149	6.954455697	0.0107351	0.0295417
**ENSG00000114209**	PDCD10	0.23718477	6.03493542	6.954431221	0.0107352	0.0295417
**ENSG00000124532**	MRS2	-0.296666425	5.129821272	6.952895016	0.0107434	0.0295565
**ENSG00000246263**	UBR5-AS1	0.442761158	3.495201791	6.950855174	0.0107543	0.0295787
**ENSG00000242288**		-0.453608141	4.280187457	6.948171463	0.0107687	0.0296104
**ENSG00000109083**	IFT20	-0.448577315	3.778235893	6.939884669	0.0108132	0.0297250
**ENSG00000091136**	LAMB1	-0.522440471	3.744721318	6.937819706	0.0108243	0.0297451
**ENSG00000005339**	CREBBP	0.145374052	8.395867298	6.937465008	0.0108262	0.0297451
**ENSG00000162623**	TYW3	-0.324070964	5.063497302	6.935696928	0.0108357	0.0297635
**ENSG00000167635**	ZNF146	0.19463576	6.845544917	6.927877658	0.0108780	0.0298717
**ENSG00000166987**	mbd6	-0.384776407	5.258790164	6.995654463	0.0108844	0.0298814

**ENSG00000176102**	CSTF3	-0.218846523	5.461336898	6.924623319	0.0108956	0.0299044
**ENSG00000198901**	PRC1	0.460924301	3.547732354	6.919476977	0.0109236	0.0299733
**ENSG00000197324**	LRP10	0.196517087	6.874462249	6.91889094	0.0109268	0.0299742
**ENSG00000100058**	CRYBB2P1	-0.423503204	4.522986472	6.909014798	0.0109806	0.0301141
**ENSG00000281383**		0.674682918	6.517711396	7.181819727	0.0109990	0.0301565
**ENSG00000116209**	TMEM59	0.203116673	6.745937479	6.904479453	0.0110055	0.0301651
**ENSG00000137171**	KLC4	-0.487729059	3.837964306	6.904035699	0.0110079	0.0301651
**ENSG00000086200**	IPO11-LRRC70	-0.292501119	5.122368257	6.903331021	0.0110118	0.0301678
**ENSG00000143252**	SDHC	-0.246510662	5.474856292	6.899370402	0.0110335	0.0302195
**ENSG00000171132**	PRKCE	0.397619148	4.219995009	6.895229995	0.0110563	0.0302739
**ENSG00000198000**	NOL8	-0.211305251	6.874033859	6.892340724	0.0110722	0.0303096
**ENSG00000072042**	RDH11	0.255728557	5.633868581	6.890295693	0.0110835	0.0303326
**ENSG00000165609**	NUDT5	-0.224676524	5.773888196	6.885835836	0.0111082	0.0303885
**ENSG00000101182**	PSMA7	0.230451653	6.021305082	6.885552415	0.0111098	0.0303885
**ENSG00000106615**	RHEB	0.323184239	4.324086401	6.883919784	0.0111188	0.0304052
**ENSG00000102241**	HTATSF1	0.240742956	6.408923502	6.879835601	0.0111415	0.0304592
**ENSG00000147099**	HDAC8	-0.424312229	3.761654338	6.873021719	0.0111794	0.0305485
**ENSG00000124422**	USP22	0.180103023	6.887275126	6.872915904	0.0111800	0.0305485
**ENSG00000113328**	CCNG1	0.218957094	6.815300593	6.870186961	0.0111952	0.0305821
**ENSG00000134375**	TIMM17A	-0.363169468	4.486087724	6.867051454	0.0112127	0.0306220
**ENSG00000107854**	TNKS2	0.139152847	7.499019441	6.864204215	0.0112286	0.0306575
**ENSG00000217128**	FNIP1	0.195516768	6.149814018	6.861108689	0.0112460	0.0306968
**ENSG00000082898**	XPO1	-0.151151866	8.202802187	6.855055754	0.0112800	0.0307785
**ENSG00000104946**	MIR4750	-0.338293497	4.972218834	6.854733655	0.0112818	0.0307785
**ENSG00000143702**	CEP170	-0.253281282	5.621611324	6.851329501	0.0113010	0.0308228
**ENSG00000275700**	AATF	0.244982426	5.647029242	6.849448817	0.0113116	0.0308319
**ENSG00000261644**		-0.410749786	3.957768123	6.849181058	0.0113131	0.0308319
**ENSG00000164118**	CEP44	-0.331212827	5.285957474	6.849168269	0.0113132	0.0308319
**ENSG00000257337**	LOC283335	0.181540323	7.08260338	6.847132347	0.0113247	0.0308552
**ENSG00000131979**	GCH1	0.283569808	5.167410869	6.844122182	0.0113417	0.0308880
**ENSG00000263986**		0.387654041	3.995562347	6.843600579	0.0113446	0.0308880
**ENSG00000198804**	COX1	0.239458683	13.34409756	6.843439276	0.0113456	0.0308880
**ENSG00000114626**	ABTB1	-0.342558249	5.333532957	6.863900572	0.0113501	0.0308922
**ENSG00000230149**		0.211002861	6.466307258	6.837297357	0.0113804	0.0309667
**ENSG00000164715**	LMTK2	0.19727687	5.968790513	6.836587781	0.0113844	0.0309696
**ENSG00000196562**	SULF2	-0.492355199	3.632934581	6.831455192	0.0114136	0.0310410
**ENSG00000117385**	P3H1	-0.371821984	4.520163179	6.827964846	0.0114335	0.0310870

**ENSG00000131943**	c19orf12	0.297756321	5.075475284	6.821769104	0.0114689	0.0311752
**ENSG00000105810**	CDK6	0.268181477	7.375252265	6.958539115	0.0114837	0.0312073
**ENSG00000279722**		-0.369389711	4.808143541	6.816819352	0.0114973	0.0312361
**ENSG00000231925**	TAPBP	0.183282126	8.557256832	6.809410586	0.0115399	0.0313438
**ENSG00000214013**	GANC	-0.325549361	5.531909467	6.835002582	0.0115474	0.0313559
**ENSG00000204390**	HSPA1L	0.523977773	3.411386901	6.807015914	0.0115538	0.0313608
**ENSG00000116266**	STXBP3	0.188846164	6.716698969	6.80676144	0.0115552	0.0313608
**ENSG00000057663**	ATG5	0.246809865	5.327660849	6.800443673	0.0115918	0.0314518
**ENSG00000102796**	DHRS12	-0.379463911	3.958327639	6.798876971	0.0116008	0.0314683
**ENSG00000225067**		0.724399357	4.74041249	7.043096482	0.0116594	0.0316190
**ENSG00000126903**	SLC10A3	0.507773198	3.728377782	6.785399506	0.0116792	0.0316620
**ENSG00000171988**	JMJD1C	-0.165614164	8.381963536	6.785037146	0.0116813	0.0316620
**ENSG00000172007**	RAB33B	0.310768838	5.372070998	6.783455963	0.0116906	0.0316789
**ENSG00000264522**	OTUD7B	0.344934129	4.624877294	6.779539437	0.0117135	0.0317327
**ENSG00000263244**		0.17329707	7.886958706	6.777642094	0.0117246	0.0317546
**ENSG00000150593**	MIR4680	0.16247219	8.375385433	6.77278063	0.0117531	0.0318236
**ENSG00000115993**	TRAK2	-0.164296488	6.802534125	6.77226326	0.0117562	0.0318236
**ENSG00000105726**	ATP13A1	-0.272283142	5.87873622	6.769160604	0.0117744	0.0318648
**ENSG00000134265**	NAPG	-0.237169373	5.460240061	6.764741929	0.0118005	0.0319270
**ENSG00000263264**		-0.235193617	5.88464103	6.75840586	0.0118379	0.0320201
**ENSG00000257315**	ZBED6	-0.145740906	8.436192728	6.752385252	0.0118736	0.0321084
**ENSG00000101246**	ARFRP1	-0.332138392	4.488956918	6.750722432	0.0118835	0.0321268
**ENSG00000118640**	VAMP8	0.446330231	4.552002896	6.782215038	0.0118911	0.0321389
**ENSG00000117748**	RPA2	0.278068599	5.215501171	6.748874584	0.0118945	0.0321399
**ENSG00000144306**	SCRN3	-0.321409562	4.479350267	6.738673522	0.0119554	0.0322960
**ENSG00000114026**	OGG1	-0.319551076	5.220808587	6.731951638	0.0119957	0.0323965
**ENSG00000244045**	MIR4723	-0.403122699	4.488179626	6.730036893	0.0120072	0.0324192
**ENSG00000058673**	ZC3H11A	-0.145454216	8.438260099	6.727523499	0.0120223	0.0324473
**ENSG00000119509**	INVS	-0.345456725	5.151514519	6.727273409	0.0120238	0.0324473
**ENSG00000141644**	MBD1	-0.254004703	6.166311762	6.725886669	0.0120322	0.0324573
**ENSG00000147118**	ZNF182	-0.31921542	4.784637335	6.725632253	0.0120337	0.0324573
**ENSG00000084652**	TXLNA	0.218664087	5.866970231	6.724785984	0.0120388	0.0324626
**ENSG00000074695**	LMAN1	0.196330019	6.780959555	6.721744285	0.0120572	0.0325037
**ENSG00000163389**	POGLUT1	-0.314596062	5.336492225	6.717223503	0.0120845	0.0325690
**ENSG00000175130**	MARCKSL1	0.444379837	4.02327919	6.712971767	0.0121102	0.0326300
**ENSG00000100410**	PHF5A	0.431119604	3.88143708	6.711579999	0.0121187	0.0326444
**ENSG00000189339**	slc35e2b	-0.189594585	6.741455933	6.707064537	0.0121461	0.0327047

**ENSG00000279767**		-0.456562533	4.136063669	6.706399239	0.0121502	0.0327047
**ENSG00000256100**		0.421294186	3.704878968	6.705885544	0.0121533	0.0327047
**ENSG00000159322**	ADPGK	-0.258943946	6.000666268	6.705839256	0.0121536	0.0327047
**ENSG00000121274**	PAPD5	-0.206797748	6.121221763	6.698050615	0.0122011	0.0328241
**ENSG00000198270**	TMEM116	-0.309376895	5.154328782	6.697247871	0.0122060	0.0328289
**ENSG00000116299**	KIAA1324	0.263550634	4.955492411	6.695002877	0.0122198	0.0328506
**ENSG00000095059**	DHPS	-0.34026229	4.807481688	6.694901219	0.0122204	0.0328506
**ENSG00000100335**	MIEF1	0.271289541	5.499790438	6.693144437	0.0122311	0.0328711
**ENSG00000273373**		-0.622771414	3.573466181	6.751880232	0.0122344	0.0328714
**ENSG00000166170**	BAG5	0.202362304	6.12508194	6.691270575	0.0122426	0.0328851
**ENSG00000149480**	MTA2	0.167496977	6.535019159	6.687061188	0.0122685	0.0329461
**ENSG00000143643**	TTC13	-0.314145926	5.988020619	6.761629148	0.0123274	0.0330950
**ENSG00000129696**	TTI2	0.340316646	5.258285485	6.691711235	0.0123303	0.0330950
**ENSG00000084623**	EIF3I	0.284481558	5.628064089	6.676014093	0.0123366	0.0331035
**ENSG00000157823**	AP3S2	0.323805896	4.962313068	6.671281346	0.0123659	0.0331737
**ENSG00000237399**	PITRM1-AS1	-0.363591384	4.651678401	6.667911877	0.0123869	0.0332213
**ENSG00000204642**	HLA-F	-0.166702765	7.340898249	6.662609567	0.0124198	0.0333012
**ENSG00000153147**	SMARCA5	0.184012156	8.060759252	6.661724286	0.0124254	0.0333075
**ENSG00000115042**	FAHD2A	-0.581866847	4.070435556	6.747829386	0.0124635	0.0334012
**ENSG00000104472**	CHRAC1	0.292584997	5.33024064	6.654537372	0.0124702	0.0334106
**ENSG00000245937**	LINC01184	-0.309361528	4.899841647	6.651711656	0.0124879	0.0334495
**ENSG00000159111**	MRPL10	0.375070089	4.410237351	6.650207154	0.0124974	0.0334662
**ENSG00000280987**	MATR3	0.154569271	8.897468552	6.649508926	0.0125018	0.0334693
**ENSG00000176542**	USF3	0.180797604	7.464510066	6.64791309	0.0125118	0.0334871
**ENSG00000243964**		0.614667065	4.006137519	6.765638424	0.0125148	0.0334871
**ENSG00000105576**	TNPO2	-0.274354506	5.171510119	6.646370582	0.0125215	0.0334963
**ENSG00000113319**	RASGRF2	-0.197510354	6.854288609	6.639136103	0.0125670	0.0336034
**ENSG00000116030**	SUMO1	0.291304959	5.105342761	6.63849154	0.0125711	0.0336034
**ENSG00000166169**	POLL	-0.377374353	4.436525422	6.638481368	0.0125711	0.0336034
**ENSG00000071051**	NCK2	0.200197008	6.735979151	6.637055976	0.0125801	0.0336189
**ENSG00000140577**	CRTC3	-0.174499853	6.883486673	6.636207079	0.0125855	0.0336246
**ENSG00000165060**	FXN	-0.402823446	3.964817623	6.635284793	0.0125913	0.0336316
**ENSG00000115839**	RAB3GAP1	-0.198814803	6.592837473	6.634093061	0.0125989	0.0336432
**ENSG00000141086**	CTRL	-0.449288032	3.896476715	6.620986284	0.0126821	0.0338567
**ENSG00000198920**	KIAA0753	-0.354064009	4.558679866	6.614925677	0.0127207	0.0339385
**ENSG00000251201**	TMED7-TICAM2	0.375188731	4.404433588	6.614698126	0.0127222	0.0339385
**ENSG00000254741**		0.279122934	6.475760366	6.696833339	0.0127226	0.0339385

**ENSG00000178685**	PARP10	-0.443549987	4.678488272	6.668603907	0.0127268	0.0339385
**ENSG00000214655**	ZSWIM8	-0.25109131	5.840831554	6.613640923	0.0127289	0.0339385
**ENSG00000147251**	DOCK11	0.142631559	8.82538374	6.611389972	0.0127433	0.0339682
**ENSG00000127804**	METTL16	-0.224813521	5.794667372	6.603807171	0.0127920	0.0340892
**ENSG00000258466**		0.402320335	3.984500279	6.602950936	0.0127975	0.0340952
**ENSG00000226849**		-0.351239331	4.509731098	6.600320421	0.0128144	0.0341316
**ENSG00000083750**	RRAGB	-0.536092527	3.867641075	6.62758397	0.0128228	0.0341452
**ENSG00000101911**	PRPS2	0.33077469	4.79301331	6.595774425	0.0128437	0.0341922
**ENSG00000039650**	PNKP	-0.43618318	5.215437786	6.730068885	0.0129006	0.0343350
**ENSG00000070367**	EXOC5	0.19373302	6.765466495	6.582753199	0.0129281	0.0343950
**ENSG00000118960**	HS1BP3	0.426583195	3.868559855	6.582142918	0.0129320	0.0343950
**ENSG00000103381**	CPPED1	0.532699534	3.470569878	6.581569851	0.0129358	0.0343950
**ENSG00000086619**	ERO1B	-0.334842295	5.133031525	6.581305546	0.0129375	0.0343950
**ENSG00000129055**	ANAPC13	0.332055166	4.678670881	6.580978552	0.0129396	0.0343950
**ENSG00000105835**	NAMPT	0.287550323	5.024761512	6.57664449	0.0129678	0.0344612
**ENSG00000146433**	TMEM181	0.240701716	6.488800678	6.578161126	0.0129780	0.0344715
**ENSG00000159640**	ACE	0.47850053	3.601028523	6.57504487	0.0129783	0.0344715
**ENSG00000011243**	AKAP8L	-0.288703029	5.785902241	6.583625225	0.0129937	0.0345037
**ENSG00000165240**	ATP7A	0.23424252	5.64265981	6.564292403	0.0130487	0.0346408
**ENSG00000159363**	ATP13A2	0.335001087	4.678590738	6.563746953	0.0130522	0.0346415
**ENSG00000100483**	VCPKMT	-0.35328494	4.612213339	6.563181281	0.0130560	0.0346426
**ENSG00000196247**	ZNF107	-0.20437696	6.981582659	6.561252998	0.0130686	0.0346674
**ENSG00000101160**	CTSZ	0.458064727	3.764675964	6.560642147	0.0130726	0.0346693
**ENSG00000007923**	DNAJC11	-0.316423574	5.051245719	6.559448568	0.0130805	0.0346813
**ENSG00000067248**	DHX29	-0.241025324	5.716265137	6.556083349	0.0131027	0.0347313
**ENSG00000165410**	CFL2	0.301122041	4.696647711	6.554400973	0.0131138	0.0347519
**ENSG00000145675**	PIK3R1	0.153360981	8.773393577	6.552535328	0.0131261	0.0347757
**ENSG00000170638**	TRABD	-0.444063563	5.402651932	6.721506065	0.0131305	0.0347787
**ENSG00000111641**	NOP2	-0.284931568	5.128252092	6.545897226	0.0131700	0.0348744
**ENSG00000152291**	TGOLN2	-0.13739454	8.509519118	6.542054533	0.0131955	0.0349331
**ENSG00000272949**		-0.418179351	3.901239188	6.528038444	0.0132890	0.0351716
**ENSG00000264235**	LOC104968399	0.256188843	6.050831418	6.525477705	0.0133061	0.0352081
**ENSG00000136560**	TANK	0.198774406	6.514058014	6.523321466	0.0133206	0.0352374
**ENSG00000006652**	IFRD1	-0.258090628	5.61656438	6.520863298	0.0133371	0.0352722
**ENSG00000163312**	HELQ	-0.229017743	5.49996871	6.519536605	0.0133460	0.0352811
**ENSG00000102738**	MRPS31	-0.290473571	5.141230347	6.519358158	0.0133472	0.0352811
**ENSG00000172086**	KRCC1	0.263810596	5.301960837	6.516846957	0.0133641	0.0353143

**ENSG00000163131**	CTSS	0.216905596	6.867562433	6.516486799	0.0133665	0.0353143
**ENSG00000176410**	DNAJC30	-0.47411415	3.491423946	6.51546263	0.0133734	0.0353189
**ENSG00000130638**	ATXN10	0.234855878	6.095417153	6.515222579	0.0133750	0.0353189
**ENSG00000137496**	IL18BP	-0.338263594	5.257956121	6.524918554	0.0133955	0.0353616
**ENSG00000231551**		-0.384958611	5.091131096	6.564463476	0.0133979	0.0353616
**ENSG00000155324**	GRAMD3	-0.423420353	3.743709767	6.508143331	0.0134228	0.0354183
**ENSG00000273749**	CYFIP1	0.297879459	5.048097076	6.503225185	0.0134561	0.0354902
**ENSG00000198015**	MRPL42	-0.188442723	6.678502911	6.502864659	0.0134586	0.0354902
**ENSG00000234912**	Mir6516	-0.453381881	3.937574405	6.502617965	0.0134602	0.0354902
**ENSG00000143149**	ALDH9A1	0.21630685	5.642729565	6.501472322	0.0134680	0.0355017
**ENSG00000161091**	MFSD12	-0.319136955	4.9613264	6.500251444	0.0134763	0.0355108
**ENSG00000183688**	RFLNB	0.211998438	6.468248811	6.499964246	0.0134783	0.0355108
**ENSG00000240230**	cox19	-0.258462931	5.390324611	6.496239339	0.0135036	0.0355686
**ENSG00000279605**		0.233188464	7.357523246	6.565322635	0.0135243	0.0356143
**ENSG00000198841**	KTI12	0.422366071	3.423221254	6.492236557	0.0135309	0.0356225
**ENSG00000147231**	cxorf57	-0.427151777	4.149815609	6.49165757	0.0135348	0.0356239
**ENSG00000254893**		0.50855363	3.516514412	6.489988413	0.0135462	0.0356450
**ENSG00000246203**		-0.298462587	4.452223611	6.488653575	0.0135553	0.0356600
**ENSG00000231310**		0.341024113	4.347556501	6.487290423	0.0135647	0.0356755
**ENSG00000267207**		0.490853239	3.515072292	6.475851879	0.0136431	0.0358729
**ENSG00000103351**	CLUAP1	-0.225983971	6.034600224	6.472049564	0.0136693	0.0359204
**ENSG00000105705**	SUGP1	-0.317403048	4.657082122	6.472004338	0.0136696	0.0359204
**ENSG00000175104**	TRAF6	0.269175862	5.539427727	6.471735981	0.0136715	0.0359204
**ENSG00000258268**		-0.436206088	3.564717867	6.461775065	0.0137404	0.0360923
**ENSG00000065883**	CDK13	0.147344076	7.454273956	6.459694027	0.0137548	0.0361211
**ENSG00000115268**	RPS15	0.327682605	7.972972515	6.686468202	0.0137651	0.0361391
**ENSG00000116871**	MAP7D1	0.204992106	6.167387461	6.456774367	0.0137751	0.0361562
**ENSG00000134001**	EIF2S1	0.218460224	6.149523728	6.453949652	0.0137947	0.0361987
**ENSG00000211584**	SLC48A1	-0.426593768	4.142296953	6.45194083	0.0138087	0.0362264
**ENSG00000189114**	BLOC1S3	0.456570923	3.918660442	6.451145336	0.0138143	0.0362318
**ENSG00000143507**	DUSP10	0.459398246	3.820049164	6.449890343	0.0138230	0.0362457
**ENSG00000198160**	MIER1	0.15716956	7.769162715	6.439114017	0.0138984	0.0364343
**ENSG00000058272**	PPP1R12A	0.162585207	8.25325455	6.437309276	0.0139111	0.0364584
**ENSG00000140988**	RPS2	0.254398102	9.088026376	6.585411395	0.0139240	0.0364830
**ENSG00000173542**	MOB1B	0.220614722	5.966213371	6.428739725	0.0139715	0.0365941
**ENSG00000139218**	SCAF11	0.155014028	8.650891945	6.428462952	0.0139734	0.0365941
**ENSG00000130518**	KIAA1683	-0.497496899	4.014760583	6.432127345	0.0139821	0.0366077

**ENSG00000197043**	ANXA6	0.165036782	7.965860774	6.426659763	0.013986	0.036609
**ENSG00000122557**	HERPUD2	0.187495559	6.637852177	6.424052318	0.014004	0.036648
**ENSG00000093072**	CECR1	-0.1690204	7.065874052	6.421917168	0.014019	0.036675
**ENSG00000163428**	LRRC58	0.192183251	6.862172369	6.421588069	0.014022	0.036675
**ENSG00000279407**		-0.467453807	3.533187767	6.420061782	0.014032	0.036694
**ENSG00000168159**	RNF187	0.32602359	4.657000924	6.418144347	0.014046	0.036721
**ENSG00000164169**	PRMT9	-0.357587925	4.493298418	6.401475731	0.014165	0.037022
**ENSG00000115806**	GORASP2	0.190199774	6.090949265	6.396621374	0.014200	0.037095
**ENSG00000135018**	UBQLN1	0.147307215	7.699089725	6.396610082	0.014200	0.037095
**ENSG00000197928**	ZNF677	-0.460724233	3.767438732	6.394362784	0.014216	0.037128
**ENSG00000070190**	DAPP1	-0.265793283	5.063328599	6.390596204	0.014243	0.037189
**ENSG00000100445**	SDR39U1	-0.300615541	5.625887406	6.407510445	0.014256	0.037195
**ENSG00000168310**	IRF2	0.213573712	6.218985652	6.388716691	0.014257	0.037195
**ENSG00000003987**	MTMR7	-0.549673882	3.728463552	6.416766789	0.014258	0.037195
**ENSG00000109084**	TMEM97	0.483942572	3.468953707	6.388174378	0.014260	0.037195
**ENSG00000261573**		-0.424948837	5.046301987	6.483498096	0.014263	0.037195
**ENSG00000229036**		-0.519691345	3.443014019	6.376328046	0.014346	0.037395
**ENSG00000123329**	ARHGAP9	-0.292780383	6.690779024	6.502160547	0.014347	0.037395
**ENSG00000259112**	NDUFC2-KCTD1	0.495399686	3.687443517	6.373519211	0.014367	0.037437
**ENSG00000133392**	MYH11	-0.270944817	5.316348096	6.371617448	0.014380	0.037464
**ENSG00000184470**	TXNRD2	-0.441524139	3.946435882	6.370631829	0.014388	0.037473
**ENSG00000102524**	TNFSF13B	0.484083793	3.710583733	6.368613385	0.014402	0.037500
**ENSG00000164040**	PGRMC2	0.251655589	5.540347382	6.36821901	0.014405	0.037500
**ENSG00000024862**	CCDC28A	0.415592269	4.39170131	6.364066614	0.014435	0.037570
**ENSG00000185753**	cxorf38	-0.257213622	5.658333049	6.35651107	0.014491	0.037704
**ENSG00000110200**	ANAPC15	-0.382377444	3.990416121	6.352276678	0.014522	0.037776
**ENSG00000167085**	PHB	0.332078399	4.728266375	6.348146201	0.014552	0.037845
**ENSG00000101367**	MAPRE1	0.211226831	6.462726551	6.347650137	0.014556	0.037845
**ENSG00000182919**	C11orf54	-0.298915758	5.049922577	6.343650749	0.014585	0.037913
**ENSG00000132153**	DHX30	-0.209179797	5.782796713	6.343019616	0.014590	0.037915
**ENSG00000146676**	MIR4657	0.180855337	6.493933467	6.341557074	0.014601	0.037934
**ENSG00000255275**		0.341451804	4.314820363	6.336390624	0.014639	0.038024
**ENSG00000137497**	NUMA1	-0.150633849	8.540539492	6.333447203	0.014661	0.038071
**ENSG00000231752**	EMBP1	0.304275268	4.546580892	6.332492261	0.014668	0.038080
**ENSG00000103018**	CYB5B	0.238364823	5.801565629	6.330853312	0.014680	0.038095
**ENSG00000247596**	TWF2	0.289291765	5.042310755	6.33077407	0.014681	0.038095
**ENSG00000137193**	PIM1	0.321815028	7.266986551	6.532972021	0.014694	0.038119

**ENSG00000196458**	znf605	-0.342208371	4.888672224	6.327317178	0.0147070	0.0381428
**ENSG00000173214**	MFSD4B	-0.42709167	4.511587493	6.343254049	0.0147295	0.0381915
**ENSG00000213066**	FGFR1OP	-0.304318931	5.347081142	6.323329586	0.0147368	0.0382010
**ENSG00000092036**	MIR4707	-0.41417092	3.99892814	6.321356498	0.0147515	0.0382298
**ENSG00000261416**		0.293203618	5.023120837	6.317490842	0.0147805	0.0382952
**ENSG00000251791**	Scarna6	0.86951006	5.254085829	6.550818167	0.0147856	0.0382991
**ENSG00000121988**	ZRANB3	-0.379337118	4.140656244	6.315296764	0.0147969	0.0383189
**ENSG00000124574**	ABCC10	-0.30415994	5.297167219	6.313361463	0.0148114	0.0383470
**ENSG00000144655**	CSRNP1	0.400945896	3.740888366	6.301451027	0.0149011	0.0385697
**ENSG00000140545**	MFGE8	-0.405956584	4.606054973	6.306326095	0.0149480	0.0386813
**ENSG00000278963**		-0.480851148	4.004383946	6.297922563	0.0149633	0.0387115
**ENSG00000044446**	PHKA2	-0.292623087	5.37229955	6.288552518	0.0149989	0.0387872
**ENSG00000196150**	ZNF250	0.384438181	4.139511317	6.288410193	0.0150000	0.0387872
**ENSG00000067840**	PDZD4	-0.477212521	3.912267462	6.287003673	0.0150107	0.0387991
**ENSG00000197892**	KIF13B	0.240730454	5.54911746	6.286668288	0.0150133	0.0387991
**ENSG00000152117**	LOC150776	-0.34403677	4.424362104	6.286343588	0.0150158	0.0387991
**ENSG00000105982**	RNF32	-0.499106294	3.745528614	6.285045135	0.0150256	0.0388078
**ENSG00000141576**	RNF157	-0.247751967	6.330345034	6.295232852	0.0150282	0.0388078
**ENSG00000103335**	MIR4722	0.299492486	7.411149576	6.472121762	0.0150302	0.0388078
**ENSG00000130826**	MIR664B	-0.23415904	5.646548016	6.281958974	0.0150492	0.0388471
**ENSG00000177853**	ZNF518A	-0.223768082	6.495853436	6.280151593	0.0150630	0.0388732
**ENSG00000182621**	PLCB1	0.482968946	4.441985577	6.340559949	0.0150833	0.0389159
**ENSG00000197816**	CCDC180	-0.359601184	4.677102772	6.274569558	0.0151057	0.0389642
**ENSG00000123131**	PRDX4	0.443638711	3.374040494	6.271474902	0.0151295	0.0390099
**ENSG00000149499**	EML3	-0.339723364	5.219019455	6.288967481	0.0151341	0.0390099
**ENSG00000112697**	TMEM30A	0.174068031	7.35039389	6.270804464	0.0151346	0.0390099
**ENSG00000135090**	TAOK3	0.163910358	7.509802118	6.257200062	0.0152395	0.0392706
**ENSG00000159346**	ADIPOR1	0.222147718	5.527961429	6.254637488	0.0152594	0.0393120
**ENSG00000267128**	RNF157-AS1	-0.423999229	3.975701565	6.251929521	0.0152804	0.0393473
**ENSG00000114446**	IFT57	-0.296404576	4.738509744	6.251902479	0.0152806	0.0393473
**ENSG00000110583**	NAA40	-0.44428746	4.042550672	6.250412929	0.0152921	0.0393673
**ENSG00000267135**		0.673326317	4.163650271	6.413169467	0.0153206	0.0394308
**ENSG00000272047**	GTF2H5	-0.457167487	3.960868762	6.242523709	0.0153535	0.0395060
**ENSG00000104231**	ZFAND1	-0.259104694	5.28620428	6.239822876	0.0153746	0.0395505
**ENSG00000123636**	BAZ2B	0.22928177	6.406075877	6.234511338	0.0154162	0.0396476
**ENSG00000159884**	CCDC107	0.387849682	3.566160396	6.233774313	0.0154219	0.0396498
**ENSG00000156052**	GNAQ	0.167913469	6.882253981	6.233204192	0.0154264	0.0396498

**ENSG00000103160**	HSDL1	-0.34777744	4.57151577	6.232948183	0.0154284	0.0396498
**ENSG00000204410**	MSH5	-0.497231231	3.836310101	6.231824262	0.0154372	0.0396627
**ENSG00000147121**	KRBOX4	-0.393330693	4.037771638	6.22650218	0.0154790	0.0397604
**ENSG00000108883**	EFTUD2	-0.199734846	5.967934847	6.223802368	0.0155003	0.0398052
**ENSG00000196466**	ZNF799	0.41290927	3.818296474	6.216510222	0.0155579	0.0399432
**ENSG00000109534**	GAR1	-0.443379539	3.843245012	6.213223356	0.0155839	0.0399996
**ENSG00000272822**		0.39882642	3.82871099	6.212773081	0.0155874	0.0399996
**ENSG00000197134**	ZNF257	0.448346837	3.909252521	6.211631045	0.0155965	0.0400130
**ENSG00000092978**	GPATCH2	-0.304377147	5.035430658	6.208237608	0.0156234	0.0400723
**ENSG00000114126**	TFDP2	-0.235446741	5.626951678	6.204573935	0.0156526	0.0401372
**ENSG00000005889**	ZFX	0.15503389	7.470085931	6.199700699	0.0156914	0.0402269
**ENSG00000119661**	DNAL1	-0.406454951	3.58180195	6.197873936	0.0157060	0.0402543
**ENSG00000112851**	ERBIN	-0.15383825	8.64089288	6.197398765	0.0157098	0.0402543
**ENSG00000239665**		-0.319533418	5.839045494	6.271845158	0.0157403	0.0403224
**ENSG00000166986**	MIR6758	-0.23216594	6.224776194	6.193115185	0.0157441	0.0403224
**ENSG00000078140**	UBE2K	0.216837712	6.528302844	6.189886442	0.0157700	0.0403788
**ENSG00000085185**	BCORL1	0.348216644	4.568795045	6.183510135	0.0158212	0.0405001
**ENSG00000138468**	SENP7	-0.196227949	7.195612549	6.179400621	0.0158543	0.040575
**ENSG00000135749**	PCNX2	0.208770164	6.738503933	6.177269659	0.0158716	0.0405973
**ENSG00000156515**	HK1	0.21243855	6.444838235	6.177045868	0.0158734	0.0405973
**ENSG00000165632**	TAF3	0.226092607	6.025185406	6.176878338	0.0158747	0.0405973
**ENSG00000135596**	MICAL1	-0.278681952	6.521176693	6.262670126	0.0158999	0.0406517
**ENSG00000184178**	SCFD2	-0.404210039	4.206144087	6.172791734	0.0159078	0.0406619
**ENSG00000229780**	UBE2Q1-AS1	0.397454191	3.554697424	6.171341619	0.0159195	0.0406820
**ENSG00000143379**	SETDB1	-0.221713841	5.83993145	6.162176821	0.0159940	0.0408623
**ENSG00000156256**	USP16	-0.259691584	6.80185214	6.246777546	0.0159997	0.0408669
**ENSG00000101146**	RAE1	-0.318451643	4.700031077	6.159726785	0.0160140	0.0408934
**ENSG00000122545**	39326	0.154577248	8.099772654	6.159158619	0.0160186	0.0408952
**ENSG00000225871**		-0.375959443	4.236437183	6.152815232	0.0160704	0.0410120
**ENSG00000185721**	DRG1	-0.24895398	5.363341566	6.152601269	0.0160722	0.0410120
**ENSG00000112081**	SRSF3	-0.122227869	8.332068384	6.149603212	0.0160968	0.0410630
**ENSG00000108774**	RAB5C	0.282988782	5.131967883	6.149203284	0.0161000	0.0410630
**ENSG00000138750**	NUP54	-0.227353006	5.688800066	6.146800666	0.0161198	0.0411033
**ENSG00000151332**	MBIP	-0.280649377	4.845241141	6.144278732	0.0161405	0.0411461
**ENSG00000241015**	TPM3P9	-0.35358516	4.234091819	6.140288117	0.0161733	0.0412198
**ENSG00000166575**	TMEM135	-0.378579491	4.438241007	6.135626329	0.0162118	0.0413078
**ENSG00000119927**	GPAM	-0.360880575	4.299932536	6.134096672	0.0162245	0.0413300

**ENSG00000161011**	SQSTM1	0.167135155	6.457031697	6.129457002	0.0162629	0.0414177
**ENSG00000266993**	LOC105378721	-0.402188854	4.102490945	6.128089206	0.0162742	0.0414365
**ENSG00000249180**		0.301908723	4.710358755	6.107550546	0.0164456	0.0418573
**ENSG00000084207**	GSTP1	0.400284307	3.996316062	6.107325622	0.0164475	0.0418573
**ENSG00000151292**	csnk1g3	0.280341614	6.513058342	6.196761598	0.0164876	0.0419492
**ENSG00000112514**	CUTA	0.317324054	4.972398406	6.101484225	0.0164966	0.0419604
**ENSG00000262664**	OVCA2	-0.411312068	3.710376816	6.101070275	0.0165000	0.0419604
**ENSG00000269900**	RMRP	0.767731591	9.1508622	6.318008441	0.0165210	0.0419933
**ENSG00000277027**	RMRP	0.767731591	9.1508622	6.318008441	0.0165210	0.0419933
**ENSG00000055483**	USP36	-0.220370495	6.509617035	6.09759049	0.0165294	0.0420044
**ENSG00000227775**		-0.270211103	5.548288763	6.095836233	0.0165442	0.0420318
**ENSG00000160803**	UBQLN4	0.2907458	4.533988461	6.095336148	0.0165484	0.0420323
**ENSG00000217130**		0.455277131	3.96721277	6.094437945	0.0165560	0.0420414
**ENSG00000258790**	KIAA0391	0.187457114	6.236114402	6.092386575	0.0165733	0.0420722
**ENSG00000122565**	CBX3	0.185757558	7.141407566	6.092047116	0.0165762	0.0420722
**ENSG00000229419**	RALGAPA1P1	0.363585379	3.708512758	6.091002967	0.0165850	0.0420845
**ENSG00000135124**	P2RX4	-0.308812107	5.031859944	6.088999316	0.0166020	0.0421173
**ENSG00000172057**	ORMDL3	-0.256484161	5.390498775	6.088092038	0.0166097	0.0421266
**ENSG00000164442**	CITED2	0.222076978	6.086455854	6.08630397	0.0166249	0.0421548
**ENSG00000048649**	RSF1	0.169374024	7.672412807	6.081684853	0.0166641	0.0422441
**ENSG00000280138**		-0.402406467	5.772277668	6.249777279	0.0166843	0.0422765
**ENSG00000100744**	GSKIP	0.335410272	4.531704161	6.079025944	0.0166868	0.0422765
**ENSG00000104714**	erich1	-0.245436847	6.172823343	6.078761492	0.0166890	0.0422765
**ENSG00000141965**	FEM1A	0.330004411	5.083175963	6.073846663	0.016731	0.0423725
**ENSG00000121022**	COPS5	-0.237455621	5.828542674	6.07216383	0.0167453	0.0423986
**ENSG00000119640**	ACYP1	-0.456738749	3.814048404	6.07042717	0.0167602	0.0424260
**ENSG00000162604**	TM2D1	-0.262708738	5.432951318	6.06406214	0.0168148	0.0425538
**ENSG00000232119**	MCTS1	-0.327151495	4.266142563	6.059789806	0.0168515	0.0426365
**ENSG00000269604**		0.34315385	4.845486729	6.048724289	0.0169471	0.0428679
**ENSG00000151690**	MFSD6	0.186203296	6.454666099	6.044548049	0.0169833	0.0429491
**ENSG00000165671**	NSD1	0.156404515	7.89517869	6.040763299	0.0170162	0.0430219
**ENSG00000213390**	ARHGAP19	0.29902407	4.819856459	6.038246736	0.0170381	0.0430669
**ENSG00000272004**		-0.361987067	3.620465052	6.035685692	0.0170604	0.0431129
**ENSG00000102908**	NFAT5	0.178187348	8.001762789	6.035036029	0.0170661	0.0431168
**ENSG00000123338**	NCKAP1L	0.132919385	7.978460355	6.03190957	0.0170934	0.0431753
**ENSG00000261971**		-0.460365469	6.179930043	6.235347232	0.0171346	0.0432689
**ENSG00000113719**	ERGIC1	0.239070391	5.765891463	6.026543436	0.0171404	0.0432731

**ENSG00000213995**	NAXD	-0.344169106	4.568236211	6.019395687	0.0172031	0.0434211
**ENSG00000013810**	TACC3	-0.224325416	6.074569733	6.018854826	0.0172079	0.0434226
**ENSG00000228606**		-0.394844298	3.802784396	6.018252305	0.0172132	0.0434255
**ENSG00000171853**	TRAPPC12	-0.228371373	5.751259694	6.01747324	0.0172201	0.0434324
**ENSG00000161203**	AP2M1	0.20804252	6.269933411	6.016791433	0.0172261	0.0434339
**ENSG00000146830**	gigyf1	-0.257754921	6.06348041	6.031583511	0.0172290	0.0434339
**ENSG00000212464**	SNORA12	0.918123974	4.192145205	6.225751818	0.0172426	0.0434579
**ENSG00000214517**	PPME1	-0.330352496	4.426251506	6.01433051	0.0172478	0.0434603
**ENSG00000105393**	BABAM1	-0.317155628	4.733292235	6.012365512	0.0172651	0.0434936
**ENSG00000128191**	MIR1306	-0.234140092	5.515075882	6.008133605	0.0173026	0.0435774
**ENSG00000040199**	PHLPP2	0.319201841	5.085288251	6.007410643	0.0173090	0.0435830
**ENSG00000177494**	ZBED2	-0.431857766	4.251945125	6.006337262	0.0173185	0.0435965
**ENSG00000015133**	CCDC88C	-0.155748801	8.268661874	6.004245646	0.0173370	0.0436326
**ENSG00000109762**	SNX25	0.296815492	4.870656302	5.997332882	0.0173985	0.0437768
**ENSG00000020426**	MNAT1	-0.421660615	3.675870357	5.992968767	0.0174374	0.0438642
**ENSG00000106459**	NRF1	0.220496092	5.18293752	5.987941128	0.0174824	0.0439666
**ENSG00000164104**	HMGB2	0.233414001	6.242287683	5.984764145	0.0175108	0.0440276
**ENSG00000196290**	NIF3L1	-0.367367132	4.273351869	5.984086053	0.0175169	0.0440324
**ENSG00000182183**	FAM159A	-0.293360067	5.329994614	5.981827323	0.0175372	0.0440727
**ENSG00000175611**	LINC00476	-0.345019376	4.010905154	5.98077413	0.0175466	0.0440859
**ENSG00000141068**	KSR1	0.329009335	4.522021031	5.978120266	0.0175705	0.0441353
**ENSG00000089022**	MAPKAPK5	-0.23156017	6.614482736	5.990065802	0.0176094	0.0442193
**ENSG00000173258**	ZNF483	-0.331346513	4.462822836	5.973218954	0.0176147	0.0442193
**ENSG00000196812**	ZSCAN16	0.405083103	3.636992119	5.97300064	0.0176167	0.0442193
**ENSG00000089289**	IGBP1	0.233636773	5.999369662	5.967977296	0.0176621	0.0443227
**ENSG00000151445**	VIPAS39	-0.337192192	4.317987557	5.962918543	0.0177079	0.0444270
**ENSG00000157110**	RBPMS	-0.470624332	4.166812555	5.976172834	0.0178086	0.0446689
**ENSG00000265257**		0.201955617	6.443095318	5.950266385	0.0178231	0.0446947
**ENSG00000279759**		-0.481581518	3.753520473	5.948311374	0.0178410	0.0447288
**ENSG00000181090**	EHMT1	-0.177593394	6.463664599	5.944662381	0.0178744	0.0448018
**ENSG00000117543**	DPH5	-0.283181174	4.974663422	5.942884066	0.0178907	0.0448303
**ENSG00000257411**		0.28004363	5.025471591	5.942486179	0.0178944	0.0448303
**ENSG00000155729**	KCTD18	-0.344197857	4.655910653	5.930917484	0.0180009	0.0450806
**ENSG00000103404**	USP31	0.408926614	4.215161463	5.930493881	0.0180048	0.0450806
**ENSG00000100105**	PATZ1	-0.261666275	5.169770748	5.929934001	0.0180100	0.0450806
**ENSG00000196693**	ZNF33B	-0.217470233	6.337851264	5.929766306	0.0180115	0.0450806
**ENSG00000131370**	SH3BP5	-0.166068677	6.836197814	5.923600077	0.0180686	0.0452126

**ENSG00000086189**	DIMT1	0.154822457	6.884974973	5.921801974	0.0180853	0.0452435
**ENSG00000228655**		-0.566296347	4.443046705	6.060163158	0.0181013	0.0452729
**ENSG00000120370**	GORAB	-0.382782177	4.313733447	5.914707569	0.0181512	0.0453869
**ENSG00000198393**	ZNF26	-0.224788086	6.004377527	5.914010014	0.0181577	0.0453923
**ENSG00000261150**	EPPK1	-0.392810509	4.473447191	5.908827006	0.0182061	0.0455023
**ENSG00000260966**		-0.498078429	3.583011312	5.907910375	0.0182147	0.0455035
**ENSG00000181852**	RNF41	0.235788999	5.243198561	5.907847837	0.0182153	0.0455035
**ENSG00000130479**	MAP1S	0.346567245	4.089452263	5.903486003	0.0182561	0.0455946
**ENSG00000135250**	SRPK2	0.167050031	7.0080192	5.894794369	0.0183378	0.0457876
**ENSG00000103274**	NUBP1	0.428154211	3.458435826	5.888960599	0.0183928	0.0459141
**ENSG00000237039**		0.61304219	3.553734754	5.957853945	0.0184771	0.0461134
**ENSG00000130985**	UBA1	0.181992697	7.243773484	5.876667257	0.0185094	0.0461830
**ENSG00000131100**	ATP6V1E1	0.208549668	5.660615281	5.873367589	0.0185408	0.0462504
**ENSG00000271092**	TMEM56-RWDD	-0.361329135	3.48541361	5.867992007	0.0185921	0.0463673
**ENSG00000160818**	GPATCH4	-0.300719287	5.193374512	5.864049567	0.0186298	0.0464503
**ENSG00000204856**	FAM216A	-0.429870005	4.155535478	5.860960168	0.0186594	0.0465073
**ENSG00000270316**	BORCS7	-0.45953158	3.416635464	5.860736021	0.0186616	0.0465073
**ENSG00000196428**	TSC22D2	0.193320428	6.188837156	5.859084906	0.0186775	0.0465357
**ENSG00000182979**	MTA1	-0.281381561	5.496896856	5.856918267	0.0186983	0.0465765
**ENSG00000013364**	MVP	-0.200309755	6.489587611	5.851779236	0.0187478	0.0466886
**ENSG00000145495**	38777	-0.120193657	8.414463499	5.848363387	0.0187807	0.0467583
**ENSG00000213442**		0.452972216	5.986989323	6.041662539	0.0187847	0.0467583
**ENSG00000074054**	CLASP1	0.142171417	7.058209375	5.844825328	0.0188149	0.0468225
**ENSG00000185829**	ARL17A	-0.377292232	4.335068635	5.84431794	0.0188199	0.0468236
**ENSG00000123609**	NMI	0.259467431	5.327393794	5.842862556	0.0188340	0.0468475
**ENSG00000257103**	LSM14A	0.149993794	7.575655647	5.839923418	0.0188625	0.0469073
**ENSG00000198894**	CIPC	0.289526034	4.945629651	5.837465829	0.0188863	0.0469555
**ENSG00000141380**	SS18	0.153557304	6.825194897	5.836485106	0.0188959	0.0469576
**ENSG00000196810**		-0.285539795	4.627585769	5.836067293	0.0188999	0.0469576
**ENSG00000137337**	MDC 1.00	-0.196000109	5.827318525	5.835991982	0.0189007	0.0469576
**ENSG00000211771**		0.372451299	4.196153134	5.832380761	0.0189358	0.0470244
**ENSG00000138593**	SECISBP2L	0.178797515	6.978037992	5.832306087	0.0189365	0.0470244
**ENSG00000185811**	IKZF1	0.126858126	9.562905155	5.828808541	0.0189707	0.0470980
**ENSG00000180773**	SLC36A4	-0.279337403	4.98340881	5.82801839	0.0189784	0.0471059
**ENSG00000129562**	DAD1	0.318963668	4.637839068	5.827308471	0.0189853	0.0471120
**ENSG00000167384**	ZNF180	0.329636676	4.506215558	5.826103574	0.0189971	0.0471300
**ENSG00000125352**	RNF113A	0.334471572	4.332757812	5.823125833	0.0190262	0.0471835

**ENSG00000197885**	nkiras1	-0.426013976	3.578830673	5.822977303	0.0190277	0.0471835
**ENSG00000092098**	RNF31	-0.27311865	5.401595351	5.818947801	0.0190672	0.0472703
**ENSG00000178741**	Cox5a	0.463020211	4.620095119	5.902210089	0.0191055	0.0473541
**ENSG00000133895**	MEN1	-0.315230025	4.671036373	5.799140522	0.0192628	0.0477324
**ENSG00000110697**	PITPNM1	0.33986475	5.643901748	5.889957541	0.0192739	0.0477489
**ENSG00000176619**	MIR7108	0.41930055	3.699055147	5.796994901	0.0192841	0.0477626
**ENSG00000168710**	AHCYL1	0.222365054	5.848602703	5.795727666	0.0192967	0.0477825
**ENSG00000160688**	FLAD1	-0.409936394	3.929888405	5.794736269	0.0193065	0.0477956
**ENSG00000171448**	ZBTB26	-0.407039553	4.130198942	5.792174724	0.0193320	0.0478474
**ENSG00000279598**		-0.547699209	4.139442264	5.880011214	0.0193375	0.0478495
**ENSG00000282851**	BISPR	-0.357825362	4.377733126	5.791171331	0.0193420	0.0478495
**ENSG00000143322**	ABL2	-0.219800123	5.701087844	5.788491374	0.0193688	0.0479043
**ENSG00000167721**	TSR1	-0.235438756	5.550509609	5.786243874	0.0193912	0.0479485
**ENSG00000180787**	ZFP3	-0.324671562	4.649842288	5.78394752	0.0194142	0.0479939
**ENSG00000069869**	NEDD4	0.442990405	4.082090534	5.780397199	0.0194497	0.0480705
**ENSG00000214029**	ZNF891	-0.276618414	5.800067777	5.792213915	0.0194947	0.0481703
**ENSG00000115520**	COQ10B	0.281861041	5.141910676	5.77169501	0.0195372	0.0482639
**ENSG00000270580**	PKD1P6-NPIPP1	-0.495982617	3.9085239	5.792241384	0.0195615	0.0483125
**ENSG00000198700**	IPO9	-0.19951056	6.789809174	5.763927089	0.0196157	0.0484348
**ENSG00000076351**	SLC46A1	-0.296165342	4.889361578	5.762885696	0.0196262	0.0484493
**ENSG00000173402**	DAG1	0.298447933	4.57509748	5.760210621	0.0196533	0.0485048
**ENSG00000072310**	SREBF1	-0.259621451	5.285238789	5.750056042	0.0197565	0.0487449
**ENSG00000228863**		0.224171603	7.496284852	5.824101447	0.0197599	0.0487449
**ENSG00000187531**	SIRT7	-0.401427297	4.865138587	5.799907658	0.0197909	0.0488098
**ENSG00000268471**	MIR4453	-0.452305728	3.87182443	5.740079719	0.0198586	0.0489651
**ENSG00000203965**	EFCAB7	-0.396969297	4.049662856	5.739545265	0.0198640	0.0489671
**ENSG00000162419**	GMEB1	0.30442268	4.871722304	5.737590894	0.0198841	0.0490050
**ENSG00000108651**	UTP6	-0.211677004	5.483342887	5.736103241	0.0198994	0.0490311
**ENSG00000103657**	HERC1	-0.145681932	8.728377394	5.734143977	0.0199195	0.0490692
**ENSG00000005436**	GCFC2	-0.245584009	5.547172893	5.732448849	0.0199369	0.0491006
**ENSG00000116685**	KIAA2013	0.327953078	4.321260744	5.726477343	0.0199985	0.0492353
**ENSG00000138378**	STAT4	-0.171987316	7.305779677	5.726229023	0.0200011	0.0492353
**ENSG00000178307**	TMEM11	0.416899171	3.578418769	5.718632588	0.0200797	0.0494173
**ENSG00000284195**		0.425708096	3.73154244	5.711376632	0.0201552	0.0495913
**ENSG00000163946**	FAM208A	0.138329272	8.169406184	5.709960703	0.0201699	0.0496159
**ENSG00000131725**	WDR44	0.195239382	5.835465988	5.708917029	0.0201808	0.0496310
**ENSG00000182185**	RAD51B	-0.465924562	4.05824239	5.723942359	0.0201918	0.0496463

**ENSG00000114745**	GORASP1	-0.393909144	3.888650124	5.705205266	0.0202195	0.0497029
**ENSG00000134882**	UBAC2	-0.21712944	5.89525754	5.701895371	0.0202542	0.0497677
**ENSG00000145337**	PYURF	0.270106671	5.248258096	5.701774785	0.0202554	0.0497677
**ENSG00000067225**	PKM	0.152455979	7.491403394	5.701232514	0.0202611	0.0497700
**ENSG00000184840**	TMED9	0.300964267	4.886115766	5.700760826	0.0202661	0.0497704
**ENSG00000202538**	RNU4-2	0.786896228	8.822308234	5.893289048	0.0202783	0.0497888
**ENSG00000263756**		0.313468716	4.645142669	5.698938657	0.0202852	0.0497939
**ENSG00000140403**	DNAJA4	-0.306601672	5.002075634	5.693274305	0.0203446	0.0499283
**ENSG00000090861**	AARS	0.261304755	5.143347691	5.69194439	0.0203586	0.0499509
**ENSG00000106993**	CDC37L1	-0.223306852	5.340394865	5.690466946	0.0203742	0.0499773
