# Supplementary material for: CSF-resident CD4+ T-cells display a distinct gene expression profile with relevance to immune surveillance and multiple sclerosis
Source: Brain Commun. 2021 Jul 13;3(3):fcab155. doi: 10.1093/braincomms/fcab155 (PMC8574295; doi:10.1093/braincomms/fcab155)
Supplement: fcab155_Supplementary_Data [file fcab155_Supplementary_Data.zip › Supplementary table 15_GO analyses of top 1000-500-100 genes.pdf]

Supplementary table 15_GO analyses of top 1000-500-100 genes

**All**				**Top 1000**				**Top 500**				**Top 100**		
**NIC**				NIC				NIC				NIC		
**GO term**	Fold enrichment	FDR		GO term	Fold enrichment	FDR		GO term	Fold enrichment	FDR		GO term	Fold enrichment	FDR
**Movement of cell or subcellular component**	1.23	2.5 x 10-6		Movement of cell or subcellular component	1.9	3.3 x 10-12		Biological adhesion	2.3	1.1 x 10-11		Cell activation	4.9	1.4 x 10-7
**Biological adhesion**	1.23	4.6 x 10-5		Biological adhesion	1.9	6.7 x 10-11		Cell adhesion	2.3	1.5 x 10-11		Cell adhesion	3.8	1.4 x 10-7
**Locomotion**	1.2	4.6 x 10-5		Locomotion	2	6.9 x 10-11		Cell activation	2.7	3.5 x 10-10		Biological adhesion	3.8	1.4 x 10-7
**Cell adhesion**	1.2	4.6 x 10-5		Cell adhesion	1.9	6.9 x 10-11		Movement of cell or subcellular component	2.3	3.5 x 10-10		Single organism cell adhesion	5.2	8.7 x 10-7
**Cell motility**	1.23	2.6 x 10-4		Cell motility	2	8.8 x 10-11		Cell-cell adhesion	2.4	1.9 x 10-9		Lymphocyte activation	5.4	1.1 x 10-6
**Localization of cell**	1.23	2.6 x 10-4		Localization of cell	2	8.8 x 10-11		Single organism cell adhesion	2.8	3.1 x 10-9		Single organismal cell-cell adhesion	5.3	1.1 x 10-6
**Cell migration**	1.23	2.6 x 10-4		Cell migration	2.1	2.2 x 10-10		Single organismal cell-cell adhesion	2.8	5.3 x 10-9		Leukocyte activation	4.9	3.9 x 10-6
**Actin filament-based process**	1.3	2.6 x 10-4		Cell-cell adhesion	1.9	1.4 x 10-9		Locomotion	2.3	4.3 x 10-8		Cholesterol biosynthetic process	25.6	5.2 x 10-6
**Cell-cell adhesion**	1.2	7.7 x 10-4		Cellular response to chemical stimulus	1.6	2.4 x 10-9		Lymphocyte activation	2.7	1.2 x 10-7		Secondary alcohol biosynthetic process	24.8	6.0 x 10-6
**Actin filament organisation**	1.38	9.3 x 10-4		Cell activation	2	3.4 x 10-9		Localization of cell	2.3	2.3 x 10-7		Sterol biosynthetic process	22.5	1.1 x 10-5
**MS**				MS				MS				MS		
**GO term**	Fold enrichment	FDR		GO term	Fold enrichment	FDR		GO term	Fold enrichment	FDR		GO term	Fold enrichment	FDR
**Movement of cell or subcellular component**	1.32	2.7 x 10-10		Biological adhesion	1.9	2.8 x 10-12		Biological adhesion	2.6	7.4 x 10-16		Single organism cell adhesion	6.1	4.9 x 10-9
**Single organismal cell-cell adhesion**	1.43	4.4 x 10-8		Cell adhesion	1.9	2.8 x 10-12		Cell adhesion	2.5	1.1 x 10-15		Single organismal cell-cell adhesion	6	3.8 x 10-8
**Cell migration**	1.38	1.9 x 10-7		Single organism cell adhesion	2.4	2.8 x 10-12		Single organism cell adhesion	3.3	3.1 x 10-15		Cell adhesion	3.9	5.5 x 10-8
**Cell activation**	1.36	2.0 x 10-7		Cell activation	2.2	5.4 x 10-12		Single organismal cell-cell adhesion	3.3	4.4 x 10-13		Biological adhesion	3.9	5.5 x 10-8
**Locomotion**	1.31	2.1 x 10-7		Single organismal cell-cell adhesion	2.4	3.0 x 10-11		Cell activation	2.8	9.0 x 10-12		Cell activation	4.9	2.9 x 10-7
**Single organism cell adhesion**	1.39	4.4 x 10-7		Movement of cell or subcellular component	1.9	3.0 x 10-11		Cell-cell adhesion	2.5	2.3 x 10-11		Lymphocyte activation	5.5	6.7 x 10-7
**Immune response**	1.3	6.7 x 10-7		Cell-cell adhesion	2	7.6 x 10-11		Leukocyte cell-cell adhesion	3.3	2.0 x 10-10		Leukocyte cell-cell adhesion	6.3	6.7 x 10-7
**Localization of cell**	1.31	1.4 x 10-6		Locomotion	1.9	6.3 x 10-10		Lymphocyte activation	3	5.4 x 10-10		Regulation of cell differentiation	3.9	6.7 x 10-7
**Cell motility**	1.31	1.4 x 10-6		Leukocyte cell-cell adhesion	2.4	4.7 x 10-9		Movement of cell or subcellular component	2.2	1.2 x 10-9		Leukocyte activation	5	2.0 x 10-6
**Biological adhesion**	1.25	4.5 x 10-6		Lymphocyte activation	2.2	5.8 x 10-9		Leukocyte activation	2.7	4.9 x 10-9		Positive regulation of developmental process	4.6	2.6 x 10-6
