# Supplementary material for: CSF-resident CD4+ T-cells display a distinct gene expression profile with relevance to immune surveillance and multiple sclerosis
Source: Brain Commun. 2021 Jul 13;3(3):fcab155. doi: 10.1093/braincomms/fcab155 (PMC8574295; doi:10.1093/braincomms/fcab155)
Supplement: fcab155_Supplementary_Data [file fcab155_Supplementary_Data.zip › Supplementary table 16_MS_CSFvNID_CSF_FDR0.05.pdf]

Supplementary table 16_MS_CSFvNID_CSF_FDR0.05

ensembl_gene_id	hgnc_symbol	logFC	logCPM	F	PValue	FDR
**ENSG00000026025**	VIM	0.412073097	10.15734889	29.67913984	4.33E-07	0.001169638
**ENSG00000198804**	MT-CO1	0.510895672	13.57186275	29.9799697	4.37E-07	0.001169638
**ENSG00000230021**		0.799109308	6.332498243	31.48796123	3.77E-07	0.001169638
**ENSG00000100219**	XBP1	0.51977117	7.180548117	27.80096392	9.1E-07	0.001217184
**ENSG00000226221**	RPL26P19	0.758422344	5.759946577	28.38480307	7.18E-07	0.001217184
**ENSG00000278791**		0.962960257	7.40636822	30.7730756	7.68E-07	0.001217184
**ENSG00000198888**	MT-ND1	0.604609305	12.10260883	28.86863843	1.26E-06	0.001442631
**ENSG00000067064**	IDI1	0.434827019	6.675343183	25.17706655	2.58E-06	0.002155953
**ENSG00000218227**		0.726748234	6.242761616	26.20892892	2.34E-06	0.002155953
**ENSG00000237973**	MTCO1P12	0.747139851	8.717317938	27.12088468	2.69E-06	0.002155953
**ENSG00000229344**	MTCO2P12	1.035214038	6.049883942	26.22489753	3.54E-06	0.002583364
**ENSG00000134107**	BHLHE40	0.607318416	7.046116958	24.14946487	5.81E-06	0.003887807
**ENSG00000163191**	S100A11	0.547238608	7.164587985	23.21768029	7.37E-06	0.004548776
**ENSG00000210082**	MT-RNR2	0.605753449	15.70122417	22.40654806	1.28E-05	0.00736351
**ENSG00000102245**	CD40LG	0.39011917	7.545789602	20.95629962	1.49E-05	0.007954057
**ENSG00000026297**	RNASET2	-0.382813734	7.064779489	20.34988735	1.92E-05	0.008876125
**ENSG00000197635**	DPP4	0.564912457	7.492169691	21.31429621	2.01E-05	0.008876125
**ENSG00000198668**	CALM1	0.256580812	9.901916448	20.14332269	2.1E-05	0.008876125
**ENSG00000198712**	MT-CO2	0.459748029	12.08485683	21.19356026	1.85E-05	0.008876125
**ENSG00000198744**	MTCO3P12	0.840057118	6.913807296	21.20283865	2.33E-05	0.008915768
**ENSG00000198938**	MT-CO3	0.460841228	12.12387692	20.78661152	2.25E-05	0.008915768
**ENSG00000171867**	PRNP	0.464864044	6.78993601	19.66738799	2.58E-05	0.009400809
**ENSG00000225630**	MTND2P28	0.866277271	8.563075805	20.41881422	3.16E-05	0.011020845
**ENSG00000248923**	MTND5P11	0.826040856	4.644308514	18.96191719	3.49E-05	0.011683973
**ENSG00000210140**	MT-TC	0.493554931	9.053470818	19.78748538	3.82E-05	0.012269973
**ENSG00000134308**	YWHAQ	0.386913693	7.14926391	18.18577042	4.9E-05	0.01399029
**ENSG00000150093**	ITGB1	0.29320232	9.59140899	17.70446081	6.05E-05	0.01399029
**ENSG00000154358**	OBSCN	-0.529648752	6.948944091	18.80018522	4.65E-05	0.01399029
**ENSG00000169871**	TRIM56	-0.36488509	6.973330629	17.6270762	6.26E-05	0.01399029
**ENSG00000198899**	MT-ATP6	0.539422604	12.26472913	18.83283441	5.68E-05	0.01399029
**ENSG00000214485**	RPL7P1	0.680457708	5.181722559	17.85794976	5.65E-05	0.01399029
**ENSG00000225972**	MTND1P23	1.123851344	5.460941646	18.94185786	5.54E-05	0.01399029
**ENSG00000228253**	MT-ATP8	0.545611438	10.81035543	18.97470052	5.46E-05	0.01399029
**ENSG00000238121**	LINC00426	-0.5834871	5.64760041	17.62060001	6.28E-05	0.01399029
**ENSG00000244716**		0.650876081	4.946738792	17.78781355	5.83E-05	0.01399029
**ENSG00000284135**	MIR3654	0.591162528	5.255874827	17.92361362	5.49E-05	0.01399029
**ENSG00000170515**	PA2G4	0.397430379	6.712001026	17.55436731	6.46E-05	0.01401486

**ENSG00000225067**	RPL23AP2	0.833743553	5.454522828	18.13685409	6.65E-05	0.014040525
**ENSG00000099331**	MYO9B	-0.409181303	7.331856261	17.25660161	7.57E-05	0.014470812
**ENSG00000129625**	REEP5	0.383865667	7.203402579	17.3030965	7.22E-05	0.014470812
**ENSG00000142669**	SH3BGRL3	0.474853335	7.84961798	17.97088498	7.39E-05	0.014470812
**ENSG00000167522**	ANKRD11	-0.303961336	7.762627069	17.20348366	7.54E-05	0.014470812
**ENSG00000198727**	MT-CYB	0.534935916	12.18947643	17.74297416	8.85E-05	0.016172442
**ENSG00000198840**	MT-ND3	0.444056172	10.49486954	17.63195789	8.87E-05	0.016172442
**ENSG00000135272**	MDFIC	0.333223873	8.629910201	16.78397863	9.19E-05	0.016396667
**ENSG00000102265**	TIMP1	0.718775273	5.108538714	16.63362274	9.71E-05	0.016946521
**ENSG00000230897**	RPS18P12	0.755520273	4.94701699	16.50341673	0.000104553	0.017851941
**ENSG00000210144**	MT-TY	0.446073949	9.358574533	17.11679052	0.000110556	0.018483588
**ENSG00000142937**	RPS8	0.330262905	9.660951209	16.35613505	0.000115995	0.018690394
**ENSG00000198089**	SFI1	-0.553656471	6.78933553	16.79549475	0.000116451	0.018690394
**ENSG00000075624**	ACTB	0.324188396	12.16013961	16.1287981	0.000121703	0.019150381
**ENSG00000177479**	ARIH2	-0.482701685	6.069775631	15.91857357	0.000133738	0.019740298
**ENSG00000181524**	RPL24P4	0.732861444	4.503605956	15.93272346	0.000132891	0.019740298
**ENSG00000212907**	MT-ND4L	0.493645208	11.42489396	16.68756998	0.000135292	0.019740298
**ENSG00000242071**	RPL7AP6	0.531136398	5.509544732	15.97856913	0.000130184	0.019740298
**ENSG00000241973**	PI4KA	-0.348599981	7.291821792	15.79814946	0.000141176	0.020231028
**ENSG00000151846**	PABPC3	0.533477728	5.663753415	15.56685367	0.000156678	0.020751248
**ENSG00000152558**	TMEM123	0.255081543	9.414874471	15.49827115	0.000161603	0.020751248
**ENSG00000196531**	NACA	0.297861868	9.369467766	15.52295641	0.000159813	0.020751248
**ENSG00000198763**	MT-ND2	0.537756046	12.46642708	16.27765997	0.00016179	0.020751248
**ENSG00000218175**		0.61763898	4.701545326	15.67697833	0.00014909	0.020751248
**ENSG00000225783**	MIAT	-0.85820698	6.781376848	16.36587148	0.000157831	0.020751248
**ENSG00000243964**	RPL23AP65	0.714670419	4.812765122	15.48047941	0.000162907	0.020751248
**ENSG00000089009**	RPL6	0.271832757	9.465951674	15.38462139	0.000170118	0.021331247
**ENSG00000198695**	MT-ND6	0.504935683	11.55562634	16.02554341	0.000179009	0.02210069
**ENSG00000209082**	MT-TL1	0.433713577	6.463220176	15.2072791	0.000184337	0.022413759
**ENSG00000115687**	PASK	-0.570623914	6.125516161	15.21035478	0.00020468	0.024515833
**ENSG00000123636**	BAZ2B	-0.428834439	6.529546187	14.85743975	0.000216081	0.025131217
**ENSG00000217027**	TPT1P4	0.822260563	4.58094799	14.97591888	0.000213348	0.025131217
**ENSG00000283795**	MIR4426	0.634865394	5.258558293	14.76372427	0.000225505	0.025852481
**ENSG00000250182**	EEF1A1P13	0.617252979	6.945528755	15.27730449	0.0002436	0.027533635
**ENSG00000236439**		0.681117643	5.638609027	14.90821831	0.000249285	0.027784836
**ENSG00000102007**	PLP2	0.499920371	6.947914509	14.95693291	0.000253539	0.027871967
**ENSG00000233476**	EEF1A1P6	0.544848154	7.03912042	14.85774192	0.000284142	0.03081407
**ENSG00000234287**		0.655902186	5.025662716	14.20582959	0.000291059	0.031143296
**ENSG00000134333**	LDHA	0.304850903	7.318096984	14.00801143	0.000318767	0.032359813

**ENSG00000136156**	ITM2B	0.243031582	9.370450112	13.98211577	0.00032259	0.032359813
**ENSG00000210049**	MT-TF	0.586578188	6.874373699	14.60520178	0.000320018	0.032359813
**ENSG00000223551**	TMSB4XP4	0.714546294	4.903718256	14.05971397	0.000318117	0.032359813
**ENSG00000234420**	ZNF37BP	-0.659951055	5.942624056	14.47477101	0.000314767	0.032359813
**ENSG00000001630**	CYP51A1	0.431509453	6.304443178	13.76731487	0.000356178	0.03282819
**ENSG00000147065**	MSN	0.271917384	9.480784442	13.74931259	0.000359152	0.03282819
**ENSG00000186469**	GNG2	0.287902763	7.908919154	13.84081468	0.000344298	0.03282819
**ENSG00000197747**	S100A10	0.380218997	6.707657994	13.80151824	0.000350598	0.03282819
**ENSG00000198886**	MT-ND4	0.483089342	13.53177515	14.42525713	0.000343188	0.03282819
**ENSG00000228502**	EEF1A1P11	0.614651145	6.076960997	14.21748103	0.000354577	0.03282819
**ENSG00000233559**	LINC00513	-0.791772297	4.580228021	13.80775811	0.000359985	0.03282819
**ENSG00000234851**	RPL23AP42	0.545919698	8.137313986	14.40922584	0.000356076	0.03282819
**ENSG00000067167**	TRAM1	0.288751946	8.197792976	13.69205969	0.00036878	0.032882898
**ENSG00000112335**	SNX3	0.455621116	5.836683065	13.70002781	0.000367424	0.032882898
**ENSG00000227081**		0.560983602	6.850213144	14.18999557	0.000377262	0.032907951
**ENSG00000227694**	RPL23AP74	0.761183631	5.185729665	14.00782014	0.000375195	0.032907951
**ENSG00000225484**	NUTM2B-AS1	-0.563120901	5.468407781	13.56133806	0.000391772	0.033806173
**ENSG00000005302**	MSL3	-0.359220643	6.559180771	13.52486463	0.000398447	0.034016387
**ENSG00000150991**	UBC	0.230165687	8.917668452	13.44625538	0.000413235	0.034543827
**ENSG00000258757**		0.405656609	6.231207538	13.4475351	0.000412989	0.034543827
**ENSG00000196205**	EEF1A1P5	0.411475194	8.52512273	13.92850356	0.000419759	0.034727518
**ENSG00000266412**	NCOA4	0.347710274	7.479669432	13.41887056	0.000426988	0.034965051
**ENSG00000148175**	STOM	0.314472315	7.245958435	13.29927379	0.000442418	0.035862676
**ENSG00000136167**	LCP1	0.241684285	9.946778315	13.27616643	0.000447196	0.035887444
**ENSG00000139644**	TMBIM6	0.245598635	9.013113475	13.11816298	0.000481319	0.037868511
**ENSG00000269888**		0.536177759	8.060514339	13.70756722	0.000480297	0.037868511
**ENSG00000125735**	TNFSF14	0.520353627	6.077528188	13.10731347	0.000516009	0.037990599
**ENSG00000144746**	ARL6IP5	0.28209044	8.223864771	13.05993123	0.000494562	0.037990599
**ENSG00000169508**	GPR183	0.243194616	8.487627381	12.96938081	0.000515905	0.037990599
**ENSG00000198786**	MT-ND5	0.44943844	13.10055858	13.47276882	0.000514369	0.037990599
**ENSG00000212664**		0.613812968	5.068359443	13.06940527	0.000492382	0.037990599
**ENSG00000226084**		0.540695895	5.209213193	13.01101005	0.000505977	0.037990599
**ENSG00000249264**	EEF1A1P9	0.692953753	4.685355623	13.04871066	0.000497157	0.037990599
**ENSG00000274272**		-0.620230338	5.488618102	13.03666866	0.000523785	0.038212475
**ENSG00000055208**	TAB2	0.232943046	8.996384765	12.9084045	0.00053081	0.038364313
**ENSG00000236552**	RPL13AP5	0.461146268	8.355515228	13.43659019	0.000535427	0.038364313
**ENSG00000140575**	IQGAP1	0.246352425	9.4290931	12.86479951	0.00054174	0.038473094
**ENSG00000227155**		-0.704195982	4.744834177	12.79991366	0.000559123	0.039017055
**ENSG00000240720**	LRRD1	0.423604488	6.252725543	12.81141602	0.000555437	0.039017055

**ENSG00000108518**	PFN1	0.38129081	8.627373345	13.16460963	0.000570324	0.039455593
**ENSG00000134444**	RELCH	-0.382413491	6.761630451	12.71804347	0.000581424	0.039879731
**ENSG00000114737**	CISH	0.705686855	7.181666146	13.22164124	0.000593261	0.0403468
**ENSG00000106546**	AHR	0.266612909	7.486434318	12.64681948	0.00059996	0.040459483
**ENSG00000214199**	EEF1A1P12	0.641888348	4.810704156	12.58940532	0.000616342	0.041217867
**ENSG00000197536**	IRF1-AS1	-0.362932597	6.79343908	12.56486085	0.000623485	0.041350982
**ENSG00000267135**		0.711759059	4.791549774	12.55290074	0.000646147	0.042502733
**ENSG00000116754**	SRSF11	-0.365613196	8.129361421	12.7574813	0.000663023	0.042607641
**ENSG00000131238**	PPT1	0.267682093	7.975286367	12.43196868	0.00066367	0.042607641
**ENSG00000225178**	RPSAP58	0.435114632	6.176508684	12.4370057	0.0006621	0.042607641
**ENSG00000213066**	CEP43	-0.624747893	5.105793766	12.40019726	0.000673667	0.042906186
**ENSG00000126264**	HCST	0.568381967	5.510710839	12.38884593	0.000693949	0.04350738
**ENSG00000180448**	ARHGAP45	-0.324844147	8.174547067	12.48622667	0.000692746	0.04350738
**ENSG00000211459**	MT-RNR1	0.463282284	14.52368865	12.76496573	0.000707456	0.044010336
**ENSG00000240376**		0.653494656	4.289261963	12.27145636	0.000715791	0.044186308
**ENSG00000113448**	PDE4D	0.348308046	7.350124199	12.26343332	0.000742131	0.045118218
**ENSG00000135945**	REV1	-0.421818975	6.199490851	12.1986084	0.00074082	0.045118218
**ENSG00000081320**	STK17B	0.213533294	9.731832173	12.15927049	0.000754709	0.045537904
**ENSG00000146677**		0.562587946	6.708947311	12.58286784	0.00076606	0.045872709
**ENSG00000269028**	MTRNR2L12	0.81649326	7.735232041	12.61829933	0.00077169	0.045872709
**ENSG00000114857**	NKTR	-0.603881506	8.680714713	12.52798452	0.00080281	0.047025917
**ENSG00000231767**	RPS27AP5	0.580158924	5.698840219	12.22215573	0.000799288	0.047025917
**ENSG00000267449**		0.712271827	4.945289454	12.14158643	0.000828856	0.048199765
**ENSG00000113742**	CPEB4	0.374826893	5.949277083	11.90641048	0.000850638	0.049110564
**ENSG00000131469**	RPL27	0.415430771	9.43770846	12.33023475	0.000866171	0.049650164
