# Supplementary material for: CSF-resident CD4+ T-cells display a distinct gene expression profile with relevance to immune surveillance and multiple sclerosis
Source: Brain Commun. 2021 Jul 13;3(3):fcab155. doi: 10.1093/braincomms/fcab155 (PMC8574295; doi:10.1093/braincomms/fcab155)
Supplement: fcab155_Supplementary_Data [file fcab155_Supplementary_Data.zip › Supplementary table 9_CSFvBLOODinNIC.pdf]

Supplementary table 9_CSFvBLOODinNIC_FDR0.05

ID	Gene	logFC	logCPM	F	PValue	FDR
**ENSG00000130164**	LDLR	3.89934476	6.692584337	899.3560761	3.52E-33	3.68E-29
**ENSG00000240720**	LRRD1	2.720303376	5.482373821	532.2835823	9.49E-31	4.96E-27
**ENSG00000001630**	CYP51A1	2.745364275	5.520396658	516.9796004	2.02E-30	7.05E-27
**ENSG00000104549**	SQLE	2.892346261	5.034519901	437.1354356	1.51E-28	3.94E-25
**ENSG00000186480**	INSIG1	2.135977042	6.39668639	398.0129399	1.62E-27	3.39E-24
**ENSG00000189283**	FHIT	-3.949497707	5.817150685	452.4563409	1.99E-26	3.46E-23
**ENSG00000198911**	SREBF2	1.716487326	6.835835724	335.4669199	1.14E-25	1.71E-22
**ENSG00000072110**	ACTN1	-2.327469027	6.576191379	355.1681383	6.45E-25	8.43E-22
**ENSG00000067064**	IDI1	1.663794834	6.140645338	281.2873908	8.24E-24	9.57E-21
**ENSG00000079459**	FDFT1	1.221593558	6.588039196	250.3413769	1.31E-22	1.37E-19
**ENSG00000184014**	DENND5A	-1.705143783	6.121900821	244.3930898	2.29E-22	2.18E-19
**ENSG00000136603**	SKIL	1.866527055	6.283337272	260.2644394	2.6E-22	2.27E-19
**ENSG00000058668**	ATP2B4	1.160013086	8.312857767	238.7914613	3.94E-22	3.17E-19
**ENSG00000113088**	GZMK	2.888181966	6.170775125	329.6516173	1.63E-21	1.22E-18
**ENSG00000120915**	EPHX2	-2.157884837	5.03751136	222.9498371	1.92E-21	1.33E-18
**ENSG00000186854**	LOC105374836	-1.362971438	7.683379591	233.3712936	2.06E-21	1.33E-18
**ENSG00000052802**	MSMO1	2.057126693	5.482285791	222.6423858	2.17E-21	1.33E-18
**ENSG00000113161**	HMGCR	1.422212348	5.974734733	218.0786088	3.19E-21	1.85E-18
**ENSG00000124788**	ATXN1	1.193287085	7.230087061	213.9465368	4.94E-21	2.72E-18
**ENSG00000113532**	ST8SIA4	1.157081887	6.665145311	207.831026	9.53E-21	4.98E-18
**ENSG00000245164**	LINC00861	-1.429370596	9.348106282	234.3953108	1.97E-20	9.79E-18
**ENSG00000182568**	SATB1	-0.989354962	8.789586776	200.6643135	2.1E-20	9.79E-18
**ENSG00000135842**	FAM129A	1.513106808	7.055032485	223.1445896	2.15E-20	9.79E-18
**ENSG00000160791**	CCR5	2.849903052	5.3340748	246.9176279	2.76E-20	1.2E-17
**ENSG00000113448**	PDE4D	1.249162425	6.903204209	191.0605155	6.28E-20	2.63E-17
**ENSG00000140443**	IGF1R	-2.15958088	6.362304826	236.9117502	7.62E-20	3.04E-17
**ENSG00000240535**		2.783947871	5.70691013	255.7038082	7.85E-20	3.04E-17
**ENSG00000152518**	ZFP36L2	0.945063753	9.594675682	184.1865318	1.41E-19	5.28E-17
**ENSG00000138814**	PPP3CA	1.311307056	6.253301484	182.9107544	1.65E-19	5.94E-17
**ENSG00000069974**	RAB27A	1.232108346	6.643567108	180.2235345	2.28E-19	7.94E-17
**ENSG00000112972**	HMGCS1	1.741594908	5.925185172	190.6482671	2.6E-19	8.78E-17
**ENSG00000140575**	iqgap1	0.934579324	9.068556814	176.1428284	3.76E-19	1.23E-16
**ENSG00000160007**	arhgap35	1.166961452	6.75098677	168.2354715	1.02E-18	3.22E-16
**ENSG00000044115**	CTNNA1	1.942421365	4.753366731	166.93197	1.2E-18	3.7E-16
**ENSG00000081059**	TCF7	-0.869217155	9.449192462	165.0711248	1.53E-18	4.57E-16

**ENSG00000101096**	NFATC2	0.841123681	7.937864262	164.2654315	1.7E-18	4.93E-16
**ENSG00000262211**		-1.742523871	5.871197041	168.7632433	2.02E-18	5.71E-16
**ENSG00000129625**	REEP5	1.141281667	6.754039994	162.1478894	2.24E-18	6.17E-16
**ENSG00000088179**	PTPN4	0.863275009	8.24899438	161.3589265	2.49E-18	6.67E-16
**ENSG00000137504**	CREBZF	-1.189585425	7.151374657	161.2055455	2.77E-18	7.24E-16
**ENSG00000148175**	STOM	1.5926731	6.72342457	189.4943995	2.87E-18	7.32E-16
**ENSG00000135272**	MDFIC	1.081973517	8.16868833	164.8852255	3.71E-18	9.24E-16
**ENSG00000100941**	PNN	-1.152972038	7.869423442	166.8124125	4.07E-18	9.9E-16
**ENSG00000186469**	GNG2	0.946457272	7.606660561	157.0275917	4.43E-18	1.05E-15
**ENSG00000138795**	LEF1	-1.027993116	9.210650256	155.9685594	6.27E-18	1.46E-15
**ENSG00000134352**	il6st	-1.676030954	9.002741151	214.8119065	6.47E-18	1.47E-15
**ENSG00000161405**	IKZF3	0.82100729	8.933283	151.1955306	9.84E-18	2.19E-15
**ENSG00000085832**	EPS15	0.748690906	7.916854409	150.6295127	1.06E-17	2.32E-15
**ENSG00000232021**	LEF1-AS1	-1.85727645	5.112578717	150.2123637	1.13E-17	2.41E-15
**ENSG00000165527**	ARF6	0.955740441	7.873421368	148.5789165	1.42E-17	2.96E-15
**ENSG00000257621**	PSMA3-AS1	-1.08211385	7.733134693	152.4081435	1.59E-17	3.23E-15
**ENSG00000034053**	APBA2	-1.609132819	5.509694163	147.6020132	1.63E-17	3.23E-15
**ENSG00000125827**	TMX4	0.966478916	7.235736175	147.5463008	1.64E-17	3.23E-15
**ENSG00000170074**	FAM153A	-2.960787484	5.296242863	180.5867899	1.92E-17	3.71E-15
**ENSG00000151692**	RNF144A	-1.324221874	6.051622986	145.6379149	2.15E-17	4.08E-15
**ENSG00000145703**	IQGAP2	0.734759299	8.3447009	144.4037675	2.56E-17	4.79E-15
**ENSG00000124942**	AHNAK	1.067571462	11.69858562	142.6751158	3.29E-17	6.03E-15
**ENSG00000124126**	PREX1	1.048524433	7.689225117	145.407142	4.71E-17	8.49E-15
**ENSG00000116824**	CD2	0.839393603	8.082335181	139.9217016	4.91E-17	8.7E-15
**ENSG00000233355**	CHRM3-AS2	-2.576931883	6.914246084	204.0488807	1.16E-16	2.03E-14
**ENSG00000163297**	ANTXR2	1.018123088	6.947828917	133.8289107	1.22E-16	2.05E-14
**ENSG00000143110**	C1orf162	-1.481867991	5.377753024	133.7316945	1.23E-16	2.05E-14
**ENSG00000186810**	CXCR3	2.564144742	5.301991824	172.3421024	1.23E-16	2.05E-14
**ENSG00000130402**	ACTN4	1.312408659	7.271872003	156.3450424	1.36E-16	2.22E-14
**ENSG00000119314**	PTBP3	0.800118155	8.590667651	132.9942302	1.38E-16	2.22E-14
**ENSG00000101665**	SMAD7	1.880213179	4.188309881	132.620446	1.46E-16	2.31E-14
**ENSG00000188452**	CERKL	0.916206955	7.285477859	132.1075004	1.58E-16	2.47E-14
**ENSG00000109861**	CTSC	1.085474227	6.624906021	130.7199751	1.95E-16	2.94E-14
**ENSG00000136167**	LCP1	0.825293986	9.651482141	130.7086656	1.96E-16	2.94E-14
**ENSG00000106546**	AHR	0.979928426	7.006653171	130.6724205	1.97E-16	2.94E-14
**ENSG00000125735**	TNFSF14	1.850480898	5.272016142	140.166227	2.26E-16	3.32E-14
**ENSG00000171867**	PRNP	1.279205957	6.413318793	134.8588918	2.38E-16	3.46E-14

**ENSG00000198932**	GPRASP1	-1.312966597	6.042104792	129.2178966	2.46E-16	3.53E-14
**ENSG00000131504**	DIAPH1	0.72840398	8.492946829	128.5066114	2.75E-16	3.89E-14
**ENSG00000213719**	CLIC1	1.361143095	5.798772878	127.9838861	2.99E-16	4.16E-14
**ENSG00000092820**	EZR	0.865671511	8.03567466	127.6678814	3.14E-16	4.32E-14
**ENSG00000197930**	ERO1A	0.787451149	7.887240892	124.6138364	5.08E-16	6.9E-14
**ENSG00000149311**	ATM	-0.739723665	10.2696002	124.2622954	5.37E-16	7.2E-14
**ENSG00000106799**	TGFBR1	0.847104338	6.888934444	123.6489446	5.92E-16	7.77E-14
**ENSG00000119900**	OGFRL1	1.20407281	5.772654152	123.6290647	5.94E-16	7.77E-14
**ENSG00000150867**	PIP4K2A	0.695209426	8.251778143	123.2056928	6.36E-16	8.21E-14
**ENSG00000246223**	LINC01550	-1.309961201	5.75349394	123.0426386	6.53E-16	8.32E-14
**ENSG00000115232**	ITGA4	0.730713638	9.411335899	122.9172526	6.66E-16	8.39E-14
**ENSG00000196405**	EVL	-1.087412677	8.817948657	137.7307642	6.95E-16	8.65E-14
**ENSG00000100385**	IL2RB	1.58934027	6.544775659	150.262974	7.07E-16	8.7E-14
**ENSG00000183508**	FAM46C	1.966007496	5.540051039	142.3727763	8.25E-16	1E-13
**ENSG00000047365**	ARAP2	0.887455091	7.868589409	121.1403727	8.87E-16	1.07E-13
**ENSG00000067955**	CBFB	0.921964428	7.103611495	120.8614982	9.28E-16	1.1E-13
**ENSG00000108654**	Mir3064	-0.93203283	10.50672841	120.5767207	9.72E-16	1.14E-13
**ENSG00000237943**		-0.972875802	7.26620926	119.6381187	1.13E-15	1.32E-13
**ENSG00000121807**	CCR2	2.394578741	6.31666687	172.5119752	1.43E-15	1.64E-13
**ENSG00000012660**	ELOVL5	0.816232408	7.247336613	114.4131436	2.7E-15	3.07E-13
**ENSG00000213064**	SFT2D2	0.776742811	7.809401804	114.0480995	2.87E-15	3.23E-13
**ENSG00000086062**	B4GALT1	0.917071743	6.587309902	113.4008344	3.2E-15	3.56E-13
**ENSG00000244509**	APOBEC3C	1.410605974	6.030089886	122.8154327	3.52E-15	3.87E-13
**ENSG00000169499**	PLEKHA2	0.952000378	6.877867879	112.3109449	3.86E-15	4.2E-13
**ENSG00000197217**	ENTPD4	-0.920147671	7.427137277	111.599902	4.35E-15	4.69E-13
**ENSG00000008517**	IL32	1.320920711	8.096432528	142.9273685	4.79E-15	5.11E-13
**ENSG00000118971**	CCND2	0.733305787	9.073366824	110.3885489	5.36E-15	5.66E-13
**ENSG00000180370**	PAK2	0.683125098	8.029485298	109.8814232	5.85E-15	6.07E-13
**ENSG00000154027**	ak5	-1.843952342	4.874366803	109.8697102	5.87E-15	6.07E-13
**ENSG00000078596**	ITM2A	0.905254426	6.969864282	109.6676673	6.08E-15	6.23E-13
**ENSG00000164111**	ANXA5	1.174426076	5.654622615	109.515542	6.24E-15	6.33E-13
**ENSG00000075624**	ACTB	0.873418661	11.77562199	108.4834722	7.47E-15	7.5E-13
**ENSG00000160310**	PRMT2	-0.740960524	7.466705739	108.4324994	7.53E-15	7.5E-13
**ENSG00000006125**	AP2B1	0.660962608	7.820807613	108.1892449	7.86E-15	7.75E-13
**ENSG00000100219**	XBP1	0.870223421	6.747595411	107.9279813	8.23E-15	8.04E-13
**ENSG00000009790**	TRAF3IP3	-1.001195624	8.641075485	118.5597267	8.89E-15	8.6E-13
**ENSG00000147065**	MSN	0.721199885	9.244651726	107.0667759	9.57E-15	9.18E-13

**ENSG00000108946**	PRKAR1A	0.717416995	7.687835881	106.1472348	1.13E-14	1.07E-12
**ENSG00000198668**	CALM1	0.659872783	9.646588237	106.0819451	1.14E-14	1.07E-12
**ENSG00000185946**	RNPC3	-1.26450374	6.849878416	119.0772988	1.46E-14	1.36E-12
**ENSG00000147457**	CHMP7	-0.799418919	7.478611162	104.2444691	1.58E-14	1.46E-12
**ENSG00000111728**	ST8SIA1	1.19295825	5.473482147	103.6140926	1.77E-14	1.62E-12
**ENSG00000163508**	EOMES	2.246483673	4.597149605	112.14812	1.8E-14	1.63E-12
**ENSG00000102007**	PLP2	1.364596621	6.521759769	120.8206669	1.82E-14	1.64E-12
**ENSG00000267534**	S1PR2	1.730997097	3.84281062	103.3533842	1.86E-14	1.66E-12
**ENSG00000077044**	DGKD	-0.924768991	6.762888051	102.4668532	2.18E-14	1.93E-12
**ENSG00000182158**	CREB3L2	0.963165422	6.383046394	102.2322724	2.27E-14	2E-12
**ENSG00000146376**	arhgap18	1.623609735	4.276575994	102.1308256	2.32E-14	2.02E-12
**ENSG00000150093**	ITGB1	1.171074458	9.128056157	125.2540481	2.6E-14	2.25E-12
**ENSG00000148730**	EIF4EBP2	0.72074346	7.737117398	101.3467777	2.67E-14	2.29E-12
**ENSG00000060237**	WNK1	0.555337312	9.381213736	101.2133213	2.74E-14	2.33E-12
**ENSG00000111863**	ADTRP	-2.494351847	4.793221524	117.3481874	2.87E-14	2.42E-12
**ENSG00000057657**	PRDM1	1.393267548	7.57518954	132.6968012	2.99E-14	2.49E-12
**ENSG00000116489**	CAPZA1	0.630943732	8.197167286	100.7176109	3E-14	2.49E-12
**ENSG00000235437**	LINC01278	-1.316454739	5.500284646	100.5192755	3.11E-14	2.55E-12
**ENSG00000139644**	TMBIM6	0.765842445	8.707798634	100.4931307	3.12E-14	2.55E-12
**ENSG00000005302**	MSL3	-0.918641937	7.068760988	100.4633635	3.14E-14	2.55E-12
**ENSG00000028137**	MIR7846	0.956764187	6.53180697	100.0887025	3.37E-14	2.7E-12
**ENSG00000123684**	LPGAT1	1.026265592	6.310881362	100.067106	3.38E-14	2.7E-12
**ENSG00000281649**	EBLN3P	-0.683169074	7.224755656	99.28254461	3.91E-14	3.07E-12
**ENSG00000171310**	CHST11	0.899266146	6.232834756	99.2823111	3.91E-14	3.07E-12
**ENSG00000173848**	NET1	-1.599718561	4.527210419	99.00876555	4.11E-14	3.21E-12
**ENSG00000145012**	LPP	0.841304278	7.19859348	98.82800196	4.25E-14	3.29E-12
**ENSG00000117643**	MAN1C1	-1.405152439	5.448720283	98.24986017	4.73E-14	3.64E-12
**ENSG00000182718**	ANXA2	1.387002156	6.527986528	117.0620425	4.86E-14	3.69E-12
**ENSG00000138758**	40787	0.94320955	5.994478146	98.08789137	4.88E-14	3.69E-12
**ENSG00000278217**		-0.930233359	11.00855619	101.2997849	5.12E-14	3.86E-12
**ENSG00000214262**	ANKRD36BP1	0.987094147	5.778363412	97.01556047	5.96E-14	4.45E-12
**ENSG00000054654**	SYNE2	0.754274571	10.56060203	96.52159324	6.54E-14	4.85E-12
**ENSG00000131051**	RBM39	-0.714569525	9.269927964	96.48594448	6.58E-14	4.85E-12
**ENSG00000144802**	nfkbiz	-1.236328243	7.195295618	113.898338	7.37E-14	5.39E-12
**ENSG00000026025**	VIM	0.82383271	9.839688013	97.13611706	7.66E-14	5.57E-12
**ENSG00000002586**	CD99	0.955176425	6.47857606	95.51600107	7.91E-14	5.7E-12
**ENSG00000163171**	CDC42EP3	1.078401514	7.243359239	107.5068311	8.53E-14	6.11E-12

**ENSG00000134107**	BHLHE40	1.795346816	6.403102352	127.2542831	8.59E-14	6.11E-12
**ENSG00000263798**		-1.714658048	5.939325489	112.2239654	1.01E-13	7.13E-12
**ENSG00000124193**	SRSF6	-0.792458032	7.815342502	94.15600482	1.03E-13	7.15E-12
**ENSG00000075426**	FOSL2	2.004391508	4.941783101	108.2152931	1.03E-13	7.15E-12
**ENSG00000120798**	NR2C1	-1.167393032	5.820139622	93.69082395	1.12E-13	7.76E-12
**ENSG00000181467**	RAP2B	0.857652356	7.160339978	93.37708279	1.19E-13	8.19E-12
**ENSG00000134308**	YWHAQ	0.883413576	6.812047773	93.18597918	1.24E-13	8.44E-12
**ENSG00000126353**	CCR7	-1.327058765	7.772400492	119.3983512	1.51E-13	1.02E-11
**ENSG00000111640**	GAPDH	0.828821668	8.063696697	94.94958763	1.53E-13	1.03E-11
**ENSG00000173597**	SULT1B1	-1.38083169	5.614003254	94.24859495	1.64E-13	1.1E-11
**ENSG00000099139**	PCSK5	-2.436243096	5.137272195	112.8616602	1.65E-13	1.1E-11
**ENSG00000213047**	DENND1B	0.797229554	6.448787152	91.58465195	1.68E-13	1.11E-11
**ENSG00000140471**	LINS1	-0.723509221	7.190203515	91.46439226	1.72E-13	1.13E-11
**ENSG00000137076**	MIR6852	0.7505275	8.916250268	91.24881526	1.8E-13	1.18E-11
**ENSG00000277726**		-0.764079718	6.888444669	91.15970972	1.83E-13	1.18E-11
**ENSG00000117090**	SLAMF1	0.834143316	6.510870762	91.15792437	1.83E-13	1.18E-11
**ENSG00000145220**	LYAR	1.098947919	5.335990585	90.95661055	1.9E-13	1.22E-11
**ENSG00000283321**		0.931209479	6.223325793	90.84693086	1.95E-13	1.24E-11
**ENSG00000261604**		1.76416234	4.298385823	90.56501909	2.06E-13	1.29E-11
**ENSG00000281106**	LINC00282	-2.532935022	4.448562801	100.1899247	2.06E-13	1.29E-11
**ENSG00000150938**	CRIM1	1.55157142	4.710556763	90.54450805	2.06E-13	1.29E-11
**ENSG00000089060**	SLC8B1	-1.181630447	5.749853835	90.3768461	2.13E-13	1.32E-11
**ENSG00000115524**	SF3B1	-1.074619548	9.651355229	107.1686084	2.14E-13	1.32E-11
**ENSG00000103257**	SLC7A5	1.612906079	3.725042436	89.99665873	2.3E-13	1.41E-11
**ENSG00000141232**	TOB1	0.801788198	7.274889049	89.81107779	2.38E-13	1.46E-11
**ENSG00000105953**	OGDH	0.82562815	6.767230978	89.37362732	2.6E-13	1.58E-11
**ENSG00000095564**	BTAF1	-1.108219455	7.735655835	106.2787148	2.61E-13	1.58E-11
**ENSG00000172292**	CERS6	-1.396643225	5.526830823	91.34256606	2.79E-13	1.68E-11
**ENSG00000071575**	TRIB2	-0.733175784	7.349892644	88.96074405	2.82E-13	1.69E-11
**ENSG00000132424**	PNISR	-1.396518435	8.869846838	117.8866114	3.14E-13	1.87E-11
**ENSG00000091490**	SEL1L3	0.685994743	6.824878685	88.34389185	3.19E-13	1.88E-11
**ENSG00000152061**	RABGAP1L	0.680125778	7.657668082	88.2201105	3.27E-13	1.92E-11
**ENSG00000120063**	GNA13	0.64519168	8.108627617	88.19619071	3.28E-13	1.92E-11
**ENSG00000105329**	TGFB1	0.726002698	7.655920772	88.12442318	3.33E-13	1.94E-11
**ENSG00000177200**	CHD9	0.63847717	7.27926879	88.09504909	3.35E-13	1.94E-11
**ENSG00000139641**	ESYT1	0.618940419	7.897700559	87.9664179	3.44E-13	1.98E-11
**ENSG00000135426**	TESPA1	-0.618252757	7.741376136	87.3742746	3.87E-13	2.21E-11

**ENSG00000162757**	C1orf74	-0.814462156	7.035490402	87.15826077	4.04E-13	2.3E-11
**ENSG00000157796**	WDR19	-1.437153936	4.613928165	86.98602344	4.19E-13	2.37E-11
**ENSG00000124813**	RUNX2	0.951404963	6.70571195	88.85590066	4.22E-13	2.37E-11
**ENSG00000172215**	Cxcr6	1.973055189	4.579913587	92.82028606	4.48E-13	2.51E-11
**ENSG00000010404**	IDS	0.53561749	8.083405263	86.49155231	4.62E-13	2.57E-11
**ENSG00000197442**	MAP3K5	0.80115703	6.683770745	86.27474767	4.83E-13	2.67E-11
**ENSG00000168056**	LTBP3	-1.37293637	6.29274441	98.88777098	4.96E-13	2.72E-11
**ENSG00000100650**	SRSF5	-1.002583236	8.973218872	99.94307726	4.97E-13	2.72E-11
**ENSG00000184743**	ATL3	0.690596488	7.142413201	85.96708562	5.14E-13	2.8E-11
**ENSG00000198846**	TOX	1.25709875	5.309171796	85.78565894	5.33E-13	2.89E-11
**ENSG00000124222**	STX16	-1.273462734	7.4502298	107.5584504	5.38E-13	2.9E-11
**ENSG00000136153**	LMO7	-1.153635865	5.628080161	85.43270122	5.73E-13	3.07E-11
**ENSG00000268027**		-0.833801642	6.443447988	85.35481075	5.82E-13	3.11E-11
**ENSG00000111859**	NEDD9	0.908969645	6.072356393	85.08776641	6.15E-13	3.26E-11
**ENSG00000269968**		0.794077677	7.160337989	84.98693734	6.28E-13	3.31E-11
**ENSG00000131149**	GSE1	1.061670653	5.608278616	84.65749236	6.71E-13	3.53E-11
**ENSG00000110090**	CPT1A	-1.845855774	5.253371193	94.74101959	7.22E-13	3.77E-11
**ENSG00000100097**	LGALS1	1.953540172	4.331154123	86.82119919	7.34E-13	3.78E-11
**ENSG00000074966**	TXK	-1.16975126	6.825965263	95.85808556	7.34E-13	3.78E-11
**ENSG00000205356**	TECPR1	-1.157983303	5.860630474	84.47683143	7.34E-13	3.78E-11
**ENSG00000131238**	PPT1	0.606445202	7.736571098	84.14988851	7.45E-13	3.82E-11
**ENSG00000239713**	APOBEC3G	1.227954963	5.515252023	84.04516117	7.64E-13	3.9E-11
**ENSG00000120137**	PANK3	0.63653127	7.479778677	83.78123364	8.03E-13	4.08E-11
**ENSG00000146285**	SCML4	-0.797389329	6.768903667	83.50028638	8.51E-13	4.3E-11
**ENSG00000065357**	DGKA	-0.907813445	9.209420664	92.33414256	9.01E-13	4.53E-11
**ENSG00000151553**	FAM160B1	0.81446758	6.416103428	83.1129368	9.22E-13	4.61E-11
**ENSG00000101445**	PPP1R16B	0.799222371	6.024623718	83.03999598	9.36E-13	4.66E-11
**ENSG00000213366**	GSTM2	-2.289259436	4.388066749	88.13040516	1.05E-12	5.21E-11
**ENSG00000181690**	PLAG1	-1.679880254	4.482750629	82.45456532	1.06E-12	5.21E-11
**ENSG00000120948**	TARDBP	-0.60552094	7.591322118	82.25501356	1.1E-12	5.41E-11
**ENSG00000143970**	ASXL2	0.651016637	7.821963928	82.22782936	1.11E-12	5.41E-11
**ENSG00000152795**	HNRNPDL	-0.67688332	8.520703452	82.06573842	1.15E-12	5.57E-11
**ENSG00000164924**	YWHAZ	0.591376951	9.598698121	81.93954991	1.18E-12	5.7E-11
**ENSG00000271503**	CCL5	2.689061951	7.814701142	111.5437138	1.26E-12	6.09E-11
**ENSG00000228956**		-1.118262582	5.614502628	81.52448193	1.28E-12	6.15E-11
**ENSG00000077984**	CST7	2.320380807	5.333146335	104.9550826	1.41E-12	6.72E-11
**ENSG00000020633**	RUNX3	0.82637184	6.715729365	81.02430289	1.42E-12	6.77E-11

**ENSG00000168807**	SNTB2	0.947270017	6.171927443	80.07475017	1.74E-12	8.24E-11
**ENSG00000007944**	MYLIP	-1.00501616	5.844724913	79.6980221	1.88E-12	8.86E-11
**ENSG00000099622**	CIRBP	-0.918156646	7.521500652	86.50073146	1.89E-12	8.86E-11
**ENSG00000164483**	SAMD3	1.010672593	6.626448886	84.41500235	1.9E-12	8.89E-11
**ENSG00000114541**	FRMD4B	1.213377176	5.140224948	79.56880353	1.94E-12	9E-11
**ENSG00000254870**	ATP6V1G2-DDX3	-0.989775555	7.657772058	90.59225008	2.03E-12	9.39E-11
**ENSG00000145075**	LOC101928882	-1.230082439	6.169621994	86.74442789	2.04E-12	9.4E-11
**ENSG00000128989**	ARPP19	0.642946996	7.803198113	79.29263686	2.05E-12	9.42E-11
**ENSG00000235532**		-1.438033996	5.823611347	87.64046398	2.17E-12	9.91E-11
**ENSG00000183918**	SH2D1A	0.705786714	6.561413618	78.99499062	2.19E-12	9.91E-11
**ENSG00000101752**	MIB1	0.644616367	6.955664878	78.99209941	2.19E-12	9.91E-11
**ENSG00000124486**	USP9X	0.533755161	8.444878695	78.90282065	2.23E-12	1.01E-10
**ENSG00000138821**	slc39a8	1.082874961	5.921816162	80.72533536	2.26E-12	1.01E-10
**ENSG00000092964**	DPYSL2	1.153560176	5.507106828	78.76866874	2.3E-12	1.03E-10
**ENSG00000169045**	HNRNPH1	-0.844799924	10.20731317	83.44310523	2.34E-12	1.04E-10
**ENSG00000147044**	CASK	0.723231356	6.541355163	78.55852351	2.4E-12	1.06E-10
**ENSG00000205744**	DENND1C	-0.909574886	6.402771489	78.51197381	2.43E-12	1.07E-10
**ENSG00000156299**	TIAM1	-0.796755053	6.759475428	78.43769318	2.47E-12	1.08E-10
**ENSG00000166128**	RAB8B	0.696573486	7.414860827	78.1061148	2.65E-12	1.16E-10
**ENSG00000198740**	ZNF652	0.532856044	7.259875085	77.72967152	2.87E-12	1.25E-10
**ENSG00000251562**	MALAT1	-0.761621503	15.3114953	77.72854056	2.87E-12	1.25E-10
**ENSG00000154144**	TBRG1	-1.134532011	6.097272507	80.97618273	2.98E-12	1.29E-10
**ENSG00000104490**	NCALD	1.31365306	4.688230051	77.47886737	3.03E-12	1.3E-10
**ENSG00000167280**	ENGASE	-1.82395703	4.929531553	83.76026768	3.12E-12	1.34E-10
**ENSG00000181104**	F2R	2.330369002	4.633074556	92.13653101	3.15E-12	1.34E-10
**ENSG00000068885**	IFT80	-1.240643905	5.755973272	80.24561235	3.21E-12	1.37E-10
**ENSG00000047634**	SCML1	-2.405904739	4.708387466	90.33662427	3.38E-12	1.43E-10
**ENSG00000205581**	HMGN1	-0.76207432	7.284227165	76.87700335	3.45E-12	1.45E-10
**ENSG00000100599**	rin3	-0.775021298	6.190415215	76.83565825	3.48E-12	1.46E-10
**ENSG00000234771**		-1.385865484	5.127428644	76.73252117	3.56E-12	1.49E-10
**ENSG00000163931**	TKT	0.914653817	6.090371222	76.72174093	3.57E-12	1.49E-10
**ENSG00000138071**	ACTR2	0.565652762	8.905768459	76.39168363	3.83E-12	1.59E-10
**ENSG00000103187**	COTL1	0.821991357	7.502816101	79.76270304	3.93E-12	1.63E-10
**ENSG00000125304**	TM9SF2	0.662539277	7.017775487	76.23480857	3.97E-12	1.63E-10
**ENSG00000184677**	ZBTB40	-0.98959735	6.812287679	81.09495065	4.09E-12	1.68E-10
**ENSG00000203880**	PCMTD2	-0.730530145	6.921972753	75.95866503	4.21E-12	1.72E-10
**ENSG00000108848**	LUC7L3	-1.454450294	8.677139474	100.6722955	4.25E-12	1.73E-10

**ENSG00000212694**	LINC01089	-1.449896021	5.584733919	82.20202538	4.48E-12	1.81E-10
**ENSG00000074800**	ENO1	0.75297734	7.263641643	75.65546602	4.5E-12	1.82E-10
**ENSG00000105639**	JAK3	-0.722071363	7.999978067	75.9415274	4.52E-12	1.82E-10
**ENSG00000145649**	GZMA	2.559439892	5.521090081	100.6066295	4.56E-12	1.83E-10
**ENSG00000164951**	PDP1	0.83217122	6.171289012	75.56321683	4.59E-12	1.83E-10
**ENSG00000170004**	CHD3	-0.558881389	8.650846373	75.54188037	4.61E-12	1.83E-10
**ENSG00000108819**	PPP1R9B	0.822715679	5.956880537	75.49186933	4.66E-12	1.85E-10
**ENSG00000130338**	TULP4	0.647836463	7.110964942	75.33657991	4.83E-12	1.9E-10
**ENSG00000011275**	RNF216	-0.612036875	6.936436688	75.33514113	4.83E-12	1.9E-10
**ENSG00000116679**	IVNS1ABP	-0.597451076	7.911379321	75.21131235	4.96E-12	1.94E-10
**ENSG00000196295**		-0.92400208	6.975547607	78.87854978	4.99E-12	1.95E-10
**ENSG00000117360**	PRPF3	-0.984623135	6.384642305	76.79864032	5.11E-12	1.98E-10
**ENSG00000164430**	MB21D1	1.118932782	4.963244314	75.0742967	5.11E-12	1.98E-10
**ENSG00000135679**	MDM2	0.578096613	7.335221321	74.57260843	5.71E-12	2.2E-10
**ENSG00000114423**	CBLB	0.552488204	7.502500005	74.29525472	6.07E-12	2.33E-10
**ENSG00000187824**	TMEM220	-1.38859977	4.880941123	74.12694487	6.3E-12	2.41E-10
**ENSG00000151835**	SACS	0.614212179	7.672746542	74.11125769	6.32E-12	2.41E-10
**ENSG00000252561**		1.930189424	4.887131243	85.99904447	6.77E-12	2.58E-10
**ENSG00000182463**	TSHZ2	-1.521719096	6.043722709	88.23119111	6.89E-12	2.61E-10
**ENSG00000182230**	LOC100507387	-2.610342533	4.598290625	84.99809252	7.21E-12	2.72E-10
**ENSG00000165819**	mettl3	-0.817058527	6.428209252	73.50605962	7.23E-12	2.72E-10
**ENSG00000158411**	MITD1	-0.992919388	5.656656984	73.47030548	7.29E-12	2.73E-10
**ENSG00000051825**	MPHOSPH9	-0.731525687	6.869218254	73.3804031	7.43E-12	2.78E-10
**ENSG00000177311**	ZBTB38	0.695751857	7.624430946	73.29161467	7.58E-12	2.82E-10
**ENSG00000276550**	HERC2P2	-1.757345468	4.951096129	78.67900661	8.2E-12	3.04E-10
**ENSG00000184384**	MAML2	-0.705065673	7.785636436	72.82629725	8.41E-12	3.11E-10
**ENSG00000122862**	SRGN	1.10036917	7.125803418	85.76738277	9.29E-12	3.41E-10
**ENSG00000067560**	RHOA	0.698888033	8.17447356	72.93301918	9.3E-12	3.41E-10
**ENSG00000185324**	CDK10	-1.524508295	5.017802182	74.19721892	9.39E-12	3.43E-10
**ENSG00000112659**	CUL9	-1.12121969	5.885324585	73.91714539	9.45E-12	3.44E-10
**ENSG00000103061**	SLC7A6OS	-0.709001992	7.31345319	71.97054014	1.02E-11	3.7E-10
**ENSG00000163728**	TTC14	-1.042148341	7.768018525	85.80062065	1.03E-11	3.71E-10
**ENSG00000065613**	SLK	0.654580297	7.584126033	71.78825287	1.06E-11	3.83E-10
**ENSG00000128245**	YWHAH	1.051382481	5.388903833	71.75477538	1.07E-11	3.84E-10
**ENSG00000138172**	CALHM2	1.297026763	4.81547331	71.64913332	1.1E-11	3.92E-10
**ENSG00000166432**	ZMAT1	-1.726271905	6.198550103	90.90806273	1.1E-11	3.93E-10
**ENSG00000059804**	SLC2A3	0.762563265	7.258760277	72.07980755	1.11E-11	3.94E-10

**ENSG00000169710**	FASN	1.229632805	5.826378486	77.80625357	1.15E-11	4.07E-10
**ENSG00000072736**	NFATC3	0.526225605	8.283304142	71.3457234	1.17E-11	4.14E-10
**ENSG00000130024**	PHF10	-0.615133124	7.260523091	71.3337115	1.18E-11	4.14E-10
**ENSG00000129351**	ILF3	-0.628309205	7.881396916	71.25939438	1.2E-11	4.2E-10
**ENSG00000147168**	IL2RG	0.578775406	8.505762623	71.22417771	1.21E-11	4.22E-10
**ENSG00000247556**		0.524363047	8.245000123	71.11308869	1.24E-11	4.31E-10
**ENSG00000182162**	P2RY8	0.701285472	7.376889357	71.099895	1.24E-11	4.31E-10
**ENSG00000127951**	FGL2	1.171560617	4.900618261	71.08400543	1.24E-11	4.31E-10
**ENSG00000110651**	CD81	0.984379305	5.639711881	71.05602261	1.25E-11	4.32E-10
**ENSG00000113368**	LMNB1	1.029814988	5.293204571	70.72550832	1.35E-11	4.64E-10
**ENSG00000163659**	TIPARP	0.797370652	5.897393683	70.69149568	1.36E-11	4.65E-10
**ENSG00000147202**	DIAPH2	0.91056463	5.48631203	70.68691714	1.36E-11	4.65E-10
**ENSG00000160593**	JAML	-0.976065389	6.35314142	72.67020979	1.37E-11	4.68E-10
**ENSG00000132485**	ZRANB2	-0.90014437	7.853385337	79.14557671	1.41E-11	4.8E-10
**ENSG00000100813**	ACIN1	-0.538046641	7.849633103	70.46758534	1.43E-11	4.84E-10
**ENSG00000197622**	CDC42SE1	-0.543336982	8.583731935	70.39161542	1.46E-11	4.91E-10
**ENSG00000196329**	GIMAP5	-0.566457022	8.198779243	70.27836794	1.49E-11	5.03E-10
**ENSG00000132716**	dcaf8	-0.806845318	6.848701562	70.20576814	1.52E-11	5.09E-10
**ENSG00000132680**	KIAA0907	-1.072858611	6.427350201	75.80655963	1.62E-11	5.4E-10
**ENSG00000078369**	GNB1	0.546282101	7.960886625	69.91158013	1.63E-11	5.4E-10
**ENSG00000131236**	CAP1	0.543638544	8.187783657	69.9075219	1.63E-11	5.4E-10
**ENSG00000266086**		-0.877055557	5.602058257	69.86649811	1.64E-11	5.43E-10
**ENSG00000144824**	PHLDB2	1.571375621	4.472953256	70.04172269	1.68E-11	5.53E-10
**ENSG00000169641**	LUZP1	0.794873185	5.78076795	69.7431604	1.69E-11	5.55E-10
**ENSG00000133302**	SLF1	0.94300188	5.65965101	69.48007941	1.79E-11	5.88E-10
**ENSG00000103064**	SLC7A6	-0.685268995	7.850471111	69.45830065	1.8E-11	5.89E-10
**ENSG00000162783**	IER5	1.562958919	4.001729412	69.32856067	1.86E-11	6.05E-10
**ENSG00000089280**	FUS	-0.709540297	8.696098389	70.81095479	1.9E-11	6.16E-10
**ENSG00000166405**	RIC3	-1.966424996	4.746262702	76.25341556	1.96E-11	6.35E-10
**ENSG00000111801**	BTN3A3	-0.678740079	7.143713447	69.05587897	1.98E-11	6.38E-10
**ENSG00000184009**	ACTG1	0.766633835	9.796667662	72.80820357	2E-11	6.43E-10
**ENSG00000168234**	TTC39C	0.544186355	7.884764953	68.99710065	2E-11	6.43E-10
**ENSG00000162889**	MAPKAPK2	0.818200152	6.004148805	68.97703168	2.01E-11	6.44E-10
**ENSG00000113580**	NR3C1	0.552793815	7.532716182	68.87940087	2.06E-11	6.57E-10
**ENSG00000100100**	PIK3IP1	-0.622907216	7.594423885	68.75642175	2.12E-11	6.74E-10
**ENSG00000027869**	SH2D2A	1.138296892	4.938758074	68.5184214	2.24E-11	7.09E-10
**ENSG00000116754**	SRSF11	-1.093221459	8.586279116	84.4993444	2.36E-11	7.45E-10

**ENSG00000068831**	RASGRP2	-1.353838645	7.178985956	86.37088631	2.39E-11	7.54E-10
**ENSG00000184787**	UBE2G2	-1.11078939	7.122642644	80.59037119	2.49E-11	7.83E-10
**ENSG00000084733**	RAB10	0.667851337	6.740730775	67.98511903	2.53E-11	7.93E-10
**ENSG00000076770**	MBNL3	0.687870917	7.12949104	67.93482262	2.56E-11	8E-10
**ENSG00000170571**	EMB	0.484724094	8.619786732	67.85497784	2.61E-11	8.13E-10
**ENSG00000155307**	SAMSN1	0.890670969	6.07139531	67.74125863	2.68E-11	8.32E-10
**ENSG00000111913**	FAM65B	-0.47246917	9.780101482	67.72211933	2.69E-11	8.33E-10
**ENSG00000167978**	SRRM2	-0.681914996	10.3359862	67.69193565	2.71E-11	8.37E-10
**ENSG00000156675**	RAB11FIP1	1.083744991	6.047981072	72.13307493	2.8E-11	8.6E-10
**ENSG00000110108**	TMEM109	1.062466394	5.080816517	67.51085241	2.83E-11	8.68E-10
**ENSG00000141367**	CLTC	0.538111821	8.215046171	67.4259507	2.89E-11	8.82E-10
**ENSG00000107104**	KANK1	-1.389607412	4.565475531	67.36181718	2.93E-11	8.93E-10
**ENSG00000188529**	SRSF10	-0.630904558	7.741390773	67.25589506	3E-11	9.13E-10
**ENSG00000117616**	RSRP1	-1.092963579	7.981744912	82.57415666	3.03E-11	9.18E-10
**ENSG00000167004**	PDIA3	0.603869647	7.350289741	67.1185286	3.1E-11	9.37E-10
**ENSG00000110422**	HIPK3	0.547265482	8.034200643	66.9180601	3.25E-11	9.79E-10
**ENSG00000100836**	PABPN1	-0.684562855	6.182544633	66.87607148	3.28E-11	9.86E-10
**ENSG00000108465**	CDK5RAP3	-1.860950861	6.05913247	84.95593541	3.37E-11	1.01E-09
**ENSG00000069667**	RORA	0.569302599	9.20596249	66.34980047	3.71E-11	1.11E-09
**ENSG00000136279**	MIR6837	-0.677761152	7.004828843	66.3302539	3.73E-11	1.11E-09
**ENSG00000133858**	ZFC3H1	-0.675445766	8.19685507	66.68207085	3.81E-11	1.13E-09
**ENSG00000099204**	ABLIM1	-0.649421844	8.693859481	66.16959543	3.87E-11	1.15E-09
**ENSG00000170017**	ALCAM	1.241090833	4.64270152	66.11363186	3.93E-11	1.16E-09
**ENSG00000149177**	PTPRJ	0.683484183	7.168129043	65.98160558	4.05E-11	1.19E-09
**ENSG00000261371**	PECAM1	-2.47621969	4.807057363	80.50426095	4.19E-11	1.23E-09
**ENSG00000167460**	TPM4	0.595002833	7.122902497	65.71361242	4.32E-11	1.26E-09
**ENSG00000111796**	Klrb1	1.672920547	7.094117504	85.06318458	4.47E-11	1.31E-09
**ENSG00000112624**	GLTSCR1L	0.542365616	7.342026491	65.53129604	4.51E-11	1.31E-09
**ENSG00000271601**	LIX1L	0.794076498	7.208037191	68.41182981	4.52E-11	1.31E-09
**ENSG00000244879**	MIR4712	-1.2772569	7.009540605	80.59173276	4.69E-11	1.36E-09
**ENSG00000198089**	SFI1	-0.851392805	7.109680016	68.83084524	4.82E-11	1.39E-09
**ENSG00000100346**	CACNA1I	-1.368090662	5.596910621	70.7865019	4.92E-11	1.42E-09
**ENSG00000113593**	ppwd1	-0.716329647	6.890812234	65.11160625	4.98E-11	1.43E-09
**ENSG00000101901**	ALG13	-0.82323539	6.684821281	65.1448707	5E-11	1.43E-09
**ENSG00000173114**	LRRN3	-2.214128941	5.75969735	83.40583887	5.1E-11	1.46E-09
**ENSG00000153914**	SREK1	-0.744605783	7.438462546	66.33211047	5.22E-11	1.49E-09
**ENSG00000083844**	ZNF264	0.760570039	7.01839641	65.38792956	5.55E-11	1.58E-09

**ENSG00000197081**	IGF2R	0.565653238	7.93111389	64.53716017	5.71E-11	1.62E-09
**ENSG00000138380**	CARF	-1.144504154	5.493031802	64.50946007	5.75E-11	1.63E-09
**ENSG00000170581**	STAT2	-0.784734974	6.647162365	64.49481829	5.77E-11	1.63E-09
**ENSG00000092841**	MYL6	0.655407218	7.087890861	64.46754424	5.8E-11	1.63E-09
**ENSG00000134884**	ARGLU1	-1.385162291	7.723811485	82.67787022	5.9E-11	1.65E-09
**ENSG00000258890**	CEP95	-0.934161328	6.465235768	66.52641633	6.07E-11	1.7E-09
**ENSG00000130414**	NDUFA10	-0.782715947	6.136673677	64.21597887	6.16E-11	1.72E-09
**ENSG00000135269**	TES	0.577967465	7.361756165	64.1798052	6.22E-11	1.73E-09
**ENSG00000240303**	ACAD11	-1.148727777	5.093237403	64.08318325	6.36E-11	1.77E-09
**ENSG00000162613**	FUBP1	-0.489248365	8.12553972	64.03631613	6.44E-11	1.78E-09
**ENSG00000109107**	ALDOC	1.228549196	4.23864556	64.00525652	6.48E-11	1.79E-09
**ENSG00000168876**	ANKRD49	-0.879306558	5.794831658	63.97969155	6.52E-11	1.8E-09
**ENSG00000005893**	LAMP2	0.601168241	6.465905252	63.93299953	6.6E-11	1.81E-09
**ENSG00000140526**	ABHD2	0.646804869	6.723637465	63.89876378	6.65E-11	1.81E-09
**ENSG00000150991**	UBC	0.587529328	8.723269846	63.89751025	6.65E-11	1.81E-09
**ENSG00000204389**	HSPA1A	1.215677479	4.752213384	63.89692499	6.65E-11	1.81E-09
**ENSG00000253352**		-0.592110097	8.367710068	63.88597136	6.67E-11	1.81E-09
**ENSG00000182541**	LIMK2	-0.777391849	6.674811446	63.8835336	6.68E-11	1.81E-09
**ENSG00000185404**	SP140L	-0.68242014	6.556816715	63.80623861	6.8E-11	1.84E-09
**ENSG00000166323**	c11orf65	-0.915364387	5.296592676	63.78280422	6.84E-11	1.84E-09
**ENSG00000137710**	RDX	0.892786514	5.419405819	63.71848513	6.95E-11	1.87E-09
**ENSG00000178573**	MAF	1.093912936	7.987688721	78.46656494	7.66E-11	2.05E-09
**ENSG00000178607**	ERN 1.00	0.956196978	7.450456877	73.33935156	7.83E-11	2.09E-09
**ENSG00000142546**	NOSIP	-0.805308589	6.488180065	63.04485548	8.17E-11	2.18E-09
**ENSG00000119760**	SUPT7L	-0.987668476	6.03486189	64.26028981	8.27E-11	2.2E-09
**ENSG00000118308**	LRMP	-0.943180749	5.581475425	62.98529985	8.29E-11	2.2E-09
**ENSG00000213639**	ppp1cb	0.619159256	8.55580686	62.90461525	8.46E-11	2.24E-09
**ENSG00000120662**	MTRF1	-1.253825996	5.052907617	62.79361014	8.69E-11	2.29E-09
**ENSG00000170348**	TMED10	0.620338714	7.835938614	62.70548042	8.87E-11	2.34E-09
**ENSG00000121310**	ECHDC2	-1.386006467	5.832953205	71.34583754	8.95E-11	2.35E-09
**ENSG00000157933**	SKI	0.671064123	7.070815457	62.53824114	9.24E-11	2.42E-09
**ENSG00000135945**	REV1	-0.748033538	6.43285866	62.5262931	9.27E-11	2.42E-09
**ENSG00000175582**	RAB6A	0.632230741	6.949061598	62.50313734	9.32E-11	2.43E-09
**ENSG00000198563**	DDX39B	-1.164203772	8.438602708	78.46058986	9.41E-11	2.44E-09
**ENSG00000089335**	ZNF302	-0.888780598	5.612914362	62.45875134	9.42E-11	2.44E-09
**ENSG00000108799**	EZH1	-0.672361776	6.868897483	62.29117971	9.81E-11	2.54E-09
**ENSG00000146112**	PPP1R18	0.839018837	6.91405471	65.71109529	9.92E-11	2.56E-09

**ENSG00000162804**	sned1	-0.990610712	5.667925803	62.22173157	9.98E-11	2.57E-09
**ENSG00000184007**	PTP4A2	0.544285628	8.590064398	61.85349676	1.09E-10	2.81E-09
**ENSG00000177565**	TBL1XR1	0.555951018	8.282648444	61.64262055	1.15E-10	2.95E-09
**ENSG00000227617**	CERS6-AS1	-1.617621797	4.373583553	61.53285025	1.18E-10	3.02E-09
**ENSG00000142867**	BCL10	0.798409339	5.473143445	61.45341779	1.2E-10	3.07E-09
**ENSG00000159086**	PAXBP1	-0.774308296	6.762810648	61.36024948	1.23E-10	3.14E-09
**ENSG00000164548**	TRA2A	-0.699972916	6.963660839	61.34181115	1.24E-10	3.14E-09
**ENSG00000091592**	NLRP1	-1.206164647	7.904539108	77.03622996	1.27E-10	3.2E-09
**ENSG00000119707**	RBM25	-0.688655588	8.593363599	63.20972324	1.3E-10	3.26E-09
**ENSG00000163961**	RNF168	0.642910427	6.477136079	61.15020815	1.3E-10	3.26E-09
**ENSG00000159128**	IFNGR2	-1.345899406	4.190222254	61.1467708	1.3E-10	3.26E-09
**ENSG00000235750**	KIAA0040	0.535846259	6.941192587	61.14001453	1.3E-10	3.26E-09
**ENSG00000137078**	SIT 1.00	1.352635935	4.628494055	61.0770438	1.32E-10	3.31E-09
**ENSG00000188811**	NHLRC3	-1.040128275	5.314118498	61.05181941	1.33E-10	3.32E-09
**ENSG00000143815**	LBR	0.536819344	7.588922821	61.00772932	1.34E-10	3.34E-09
**ENSG00000143889**	HNRNPLL	0.869605162	6.68054926	63.60176518	1.35E-10	3.34E-09
**ENSG00000127022**	CANX	0.590776992	8.433112658	60.93086108	1.37E-10	3.39E-09
**ENSG00000162739**	SLAMF6	0.663424222	6.999994816	60.90803306	1.38E-10	3.41E-09
**ENSG00000135074**	ADAM19	0.958663778	6.455743262	65.40144527	1.42E-10	3.51E-09
**ENSG00000162892**	IL24	-0.88212414	6.198682925	60.55080264	1.51E-10	3.71E-09
**ENSG00000125384**	PTGER2	1.115263585	6.666199273	70.82745012	1.52E-10	3.73E-09
**ENSG00000162630**	B3GALT2	1.018204034	4.818928434	60.40300923	1.56E-10	3.81E-09
**ENSG00000111885**	MAN1A1	1.064059473	5.311855423	60.40287558	1.56E-10	3.81E-09
**ENSG00000078589**	p2ry10	0.768181232	6.186376035	60.39109084	1.57E-10	3.81E-09
**ENSG00000139182**	clstn3	0.986466777	5.47653842	60.29917225	1.6E-10	3.89E-09
**ENSG00000113441**	LNPEP	0.466901098	9.281568513	60.23975153	1.62E-10	3.94E-09
**ENSG00000076641**	PAG1	0.446550544	8.964754049	60.20989911	1.64E-10	3.96E-09
**ENSG00000171522**	PTGER4	0.683595174	6.609956625	59.94983901	1.75E-10	4.22E-09
**ENSG00000138600**	SPPL2A	0.721199546	6.309508516	59.91267362	1.76E-10	4.25E-09
**ENSG00000136238**	RAC1	0.646608676	6.405570132	59.88742946	1.77E-10	4.26E-09
**ENSG00000124767**	GLO1	0.934563187	5.49383227	59.80059781	1.81E-10	4.35E-09
**ENSG00000123908**	AGO2	0.477976099	7.765613124	59.69673296	1.86E-10	4.45E-09
**ENSG00000178338**	ZNF354B	-1.000756935	4.835708083	59.58651573	1.91E-10	4.56E-09
**ENSG00000126264**	HCST	1.391115091	5.034629581	63.63946297	1.96E-10	4.67E-09
**ENSG00000146021**	KLHL3	-1.392096991	4.9490354	60.66348847	1.98E-10	4.7E-09
**ENSG00000091039**	OSBPL8	0.574216465	8.536736276	59.3776313	2.01E-10	4.78E-09
**ENSG00000163577**	EIF5A2	1.155952177	4.287986822	59.28784098	2.06E-10	4.87E-09

**ENSG00000139793**	MBNL2	0.626710064	6.351166135	59.16737377	2.12E-10	4.99E-09
**ENSG00000137312**	FLOT1	-1.001055623	4.882945943	59.16612902	2.12E-10	4.99E-09
**ENSG00000060339**	CCAR1	-0.499040116	7.589961347	59.16214316	2.13E-10	4.99E-09
**ENSG00000284368**		-1.687720275	5.6976834	71.50970363	2.16E-10	5.07E-09
**ENSG00000234608**	MAPKAPK5-AS1	-1.183565416	4.694123021	58.95175892	2.24E-10	5.24E-09
**ENSG00000140396**	NCOA2	0.523483612	7.370760078	58.82391668	2.31E-10	5.4E-09
**ENSG00000270127**		-1.622624388	4.518686935	59.86761581	2.36E-10	5.48E-09
**ENSG00000134250**	NOTCH2	0.520538473	7.273645025	58.74488436	2.36E-10	5.48E-09
**ENSG00000100201**	DDX17	-1.000798689	10.53317721	69.74244413	2.39E-10	5.54E-09
**ENSG00000139746**	RBM26	-0.619269729	8.060257651	58.59143471	2.45E-10	5.67E-09
**ENSG00000010244**	Mir632	-0.514507799	8.857938931	58.58477087	2.46E-10	5.67E-09
**ENSG00000279088**		-1.846174176	3.817956136	58.48013269	2.52E-10	5.81E-09
**ENSG00000077238**	IL4R	-0.765240787	6.297015802	58.23164755	2.69E-10	6.17E-09
**ENSG00000112303**	VNN 2.00	-1.855169547	4.130434555	59.33061455	2.69E-10	6.17E-09
**ENSG00000066027**	PPP2R5A	0.540509669	6.692650398	58.2131769	2.7E-10	6.17E-09
**ENSG00000158321**	AUTS2	1.459324474	6.077360438	71.21315092	2.75E-10	6.29E-09
**ENSG00000198648**	STK39	0.802771159	5.931734287	58.07915277	2.79E-10	6.36E-09
**ENSG00000274810**	NPHP3-ACAD11	-1.233045624	6.488489957	68.551601	2.82E-10	6.41E-09
**ENSG00000103342**	GSPT1	0.519649528	7.730531075	57.967929	2.87E-10	6.51E-09
**ENSG00000111266**	DUSP16	0.597601213	7.169869234	57.86890855	2.94E-10	6.65E-09
**ENSG00000182944**	EWSR1	-0.582580366	7.930043775	57.86604009	2.95E-10	6.65E-09
**ENSG00000204219**	TCEA3	-1.23479705	4.629713181	57.80225108	2.99E-10	6.75E-09
**ENSG00000067167**	TRAM1	0.567864819	7.942457014	57.74257669	3.04E-10	6.83E-09
**ENSG00000112297**	AIM1	0.423072083	7.858051009	57.72665355	3.05E-10	6.85E-09
**ENSG00000105519**	CAPS	-1.474751204	4.413739013	57.6626293	3.1E-10	6.94E-09
**ENSG00000117450**	PRDX1	1.058068142	5.443938762	58.16132266	3.16E-10	7.06E-09
**ENSG00000044574**	HSPA5	0.604149482	7.303062126	57.50858253	3.22E-10	7.19E-09
**ENSG00000106780**	MEGF9	0.719242559	5.801787763	57.47304566	3.25E-10	7.23E-09
**ENSG00000133935**	C14orf1	1.146351334	4.654059069	57.47149994	3.26E-10	7.23E-09
**ENSG00000266714**	MYO15B	-2.662590599	5.112036088	71.88020322	3.35E-10	7.43E-09
**ENSG00000258757**		0.909378361	5.847706013	57.77209616	3.39E-10	7.49E-09
**ENSG00000138594**	TMOD3	0.542274789	7.314972997	57.30217635	3.4E-10	7.5E-09
**ENSG00000112739**	PRPF4B	-0.553156507	8.123003991	57.25515163	3.44E-10	7.57E-09
**ENSG00000105851**	PIK3CG	0.655949065	6.502439601	57.20198015	3.49E-10	7.66E-09
**ENSG00000069702**	TGFBR3	1.192841212	6.094125089	65.52347126	3.5E-10	7.67E-09
**ENSG00000254995**	STX16-NPEPL1	-1.178581729	6.502532526	66.67536773	3.51E-10	7.68E-09
**ENSG00000197912**	SPG7	-0.824489966	5.907167756	57.12644081	3.55E-10	7.76E-09

**ENSG00000204977**	TRIM13	0.672839258	6.175185195	57.08190206	3.59E-10	7.83E-09
**ENSG00000147649**	MTDH	0.529063293	7.534303036	56.9826394	3.69E-10	8.02E-09
**ENSG00000047849**	MAP4	0.579751053	6.938193031	56.7521581	3.91E-10	8.48E-09
**ENSG00000169508**	GPR183	0.883939117	8.172527509	65.88836167	4.01E-10	8.69E-09
**ENSG00000142227**	EMP3	0.960082701	6.788484364	63.53364676	4.06E-10	8.77E-09
**ENSG00000080824**	HSP90AA1	0.594498465	9.09257743	56.5843976	4.08E-10	8.8E-09
**ENSG00000091409**	ITGA6	-0.542051323	8.139382912	56.53698858	4.13E-10	8.89E-09
**ENSG00000223501**	VPS52	-0.993340203	5.592304462	56.30359209	4.39E-10	9.42E-09
**ENSG00000003756**	RBM5	-0.750247603	8.148577657	60.96998041	4.56E-10	9.78E-09
**ENSG00000121210**	KIAA0922	-0.563364775	7.663443044	56.10963061	4.61E-10	9.86E-09
**ENSG00000152127**	MGAT5	0.662587501	6.986153834	56.08610571	4.64E-10	9.9E-09
**ENSG00000115935**	WIPF1	0.366765666	9.399506427	55.97182031	4.78E-10	1.02E-08
**ENSG00000166801**	FAM111A	-0.863979625	7.156661252	61.19938441	5.04E-10	1.07E-08
**ENSG00000152270**	PDE3B	-0.667487454	8.146261768	57.65722644	5.08E-10	1.08E-08
**ENSG00000169592**	INO80E	-1.21748659	5.16778477	56.57342865	5.28E-10	1.12E-08
**ENSG00000172757**	CFL1	0.783949276	8.272069183	61.84333455	5.46E-10	1.15E-08
**ENSG00000159445**	THEM4	-0.675806057	6.722490658	55.44984451	5.47E-10	1.15E-08
**ENSG00000121578**	B4GALT4	-1.002491014	4.801720775	55.44743588	5.47E-10	1.15E-08
**ENSG00000129197**	RPAIN	-0.889957012	5.4848664	55.3506623	5.61E-10	1.18E-08
**ENSG00000284554**		0.840812726	5.358435683	55.30087734	5.68E-10	1.19E-08
**ENSG00000107771**	CCSER2	0.479992574	7.977963575	55.26502365	5.74E-10	1.2E-08
**ENSG00000127980**	PEX1	-0.875884743	5.99312705	55.24579314	5.76E-10	1.2E-08
**ENSG00000169813**	HNRNPF	0.593541145	7.267691275	55.2406387	5.77E-10	1.2E-08
**ENSG00000143401**	ANP32E	0.674045823	7.344039303	56.09107329	6.05E-10	1.26E-08
**ENSG00000240038**	AMY2B	-1.619597152	5.567021943	65.38319951	6.06E-10	1.26E-08
**ENSG00000204789**		-1.500815234	3.953087241	55.04114978	6.08E-10	1.26E-08
**ENSG00000140398**	NEIL1	-1.709254212	4.597746845	58.50233691	6.12E-10	1.26E-08
**ENSG00000196914**	ARHGEF12	1.027142602	5.242590719	54.97602786	6.18E-10	1.28E-08
**ENSG00000148158**	SNX30	0.874721878	5.583567688	54.93605355	6.25E-10	1.29E-08
**ENSG00000196696**	PDXDC2P	-1.278589694	5.178634617	56.52827923	6.27E-10	1.29E-08
**ENSG00000277734**		0.587897744	9.291363113	54.90302837	6.3E-10	1.29E-08
**ENSG00000179218**	CALR	0.545738154	7.347666707	54.86981339	6.36E-10	1.3E-08
**ENSG00000026508**	CD44	0.504303346	9.792697511	54.83763871	6.41E-10	1.31E-08
**ENSG00000167470**	MIDN	1.037387348	4.989970505	54.75273578	6.55E-10	1.34E-08
**ENSG00000166949**	SMAD3	0.617788483	7.016052229	54.64397596	6.74E-10	1.37E-08
**ENSG00000118680**	MYL12B	0.775328394	7.603490452	59.73327803	6.8E-10	1.38E-08
**ENSG00000026950**	BTN3A1	-0.812443297	7.583341971	60.17988462	6.88E-10	1.39E-08

**ENSG00000167792**	NDUFV1	-1.137673525	5.386715818	55.56692329	7.11E-10	1.44E-08
**ENSG00000126602**	TRAP1	-0.890238074	5.517168503	54.00323606	7.97E-10	1.61E-08
**ENSG00000113263**	ITK	-0.458069215	9.128637372	53.95903755	8.06E-10	1.62E-08
**ENSG00000196126**	HLA-DRB1	1.75959708	3.990995904	54.19183554	8.09E-10	1.63E-08
**ENSG00000185163**	DDX51	-1.14436968	4.593620396	53.8728765	8.25E-10	1.66E-08
**ENSG00000164733**	CTSB	0.635176492	6.694409617	53.82741457	8.35E-10	1.67E-08
**ENSG00000152256**	PDK1	-1.328234543	6.983910057	66.44428775	8.39E-10	1.68E-08
**ENSG00000119408**	NEK6	1.084833269	4.63434608	53.74795087	8.52E-10	1.7E-08
**ENSG00000073605**	GSDMB	-1.458168059	5.117288664	58.25170684	8.56E-10	1.71E-08
**ENSG00000155368**	DBI	1.101030183	4.833246691	53.70123305	8.63E-10	1.71E-08
**ENSG00000198589**	LRBA	0.474933712	8.365965138	53.69890877	8.63E-10	1.71E-08
**ENSG00000124795**	DEK	0.645407697	7.915677872	55.25299551	8.69E-10	1.72E-08
**ENSG00000173209**	AHSA2	-1.086805385	7.802763869	65.54516494	8.8E-10	1.74E-08
**ENSG00000175073**	VCPIP1	0.564013399	7.556471892	53.54749876	8.98E-10	1.77E-08
**ENSG00000186814**	ZSCAN30	-1.057731296	5.04969899	53.47220322	9.16E-10	1.8E-08
**ENSG00000107263**	RAPGEF1	0.505548982	7.308506338	53.46664823	9.18E-10	1.8E-08
**ENSG00000118689**	FOXO3	0.797534407	5.740526966	53.22069181	9.79E-10	1.92E-08
**ENSG00000059588**	TARBP1	-1.111837664	6.011611701	58.37472291	9.82E-10	1.92E-08
**ENSG00000120699**	EXOSC8	-0.864706364	5.835120354	53.15064059	9.98E-10	1.95E-08
**ENSG00000100596**	SPTLC2	0.693725833	6.267043579	53.11099556	1.01E-09	1.97E-08
**ENSG00000129128**	SPCS3	0.429344509	8.162680019	53.09090699	1.01E-09	1.97E-08
**ENSG00000085491**	SLC25A24	0.912628202	5.584582817	53.06228477	1.02E-09	1.98E-08
**ENSG00000140030**	GPR65	1.008552284	6.27396962	58.69016648	1.05E-09	2.03E-08
**ENSG00000183486**	MX2	-0.654567164	6.658751884	52.91920096	1.06E-09	2.05E-08
**ENSG00000197976**	AKAP17A	-1.158006812	5.99052996	58.96677433	1.06E-09	2.05E-08
**ENSG00000130254**	SAFB2	-0.577510781	6.796754496	52.90995286	1.06E-09	2.05E-08
**ENSG00000182796**	TMEM198B	-2.0137109	4.445732249	58.66020675	1.06E-09	2.05E-08
**ENSG00000154358**	OBSCN	-0.79831544	7.229865959	56.84030059	1.07E-09	2.06E-08
**ENSG00000214765**	SEPT7P2	-1.070172356	5.134519833	52.84638407	1.08E-09	2.07E-08
**ENSG00000114850**	SSR3	0.660741622	6.62400427	52.83548912	1.08E-09	2.08E-08
**ENSG00000168071**	CCDC88B	-0.99552739	5.726066691	53.76291712	1.09E-09	2.09E-08
**ENSG00000236213**		1.042414976	4.953598082	52.79320394	1.1E-09	2.09E-08
**ENSG00000132274**	TRIM22	-0.564028863	8.119816514	52.77100856	1.1E-09	2.1E-08
**ENSG00000171488**	LRRC8C	0.509909156	8.133097104	52.71396737	1.12E-09	2.13E-08
**ENSG00000256525**	POLG2	-1.123183034	5.022374988	52.67731256	1.13E-09	2.15E-08
**ENSG00000153814**	JAZF1	0.852565803	5.087032606	52.64603439	1.14E-09	2.16E-08
**ENSG00000123983**	ACSL3	0.839077441	5.680916414	52.47921773	1.19E-09	2.25E-08

**ENSG00000122958**	VPS26A	0.784034314	5.672494569	52.47495744	1.19E-09	2.25E-08
**ENSG00000168172**	HOOK3	0.565310866	7.229188579	52.44851525	1.2E-09	2.26E-08
**ENSG00000052126**	PLEKHA5	1.20185098	4.630063483	52.4430137	1.2E-09	2.26E-08
**ENSG00000112419**	PHACTR2	0.760673655	7.153009391	55.61587361	1.2E-09	2.26E-08
**ENSG00000139514**	SLC7A1	0.816910584	5.619040215	52.38657786	1.22E-09	2.29E-08
**ENSG00000204054**		1.181632353	4.401881098	52.28738424	1.25E-09	2.35E-08
**ENSG00000171552**	BCL2L1	1.007261664	4.469105566	52.2244654	1.28E-09	2.38E-08
**ENSG00000184205**	TSPYL2	-0.769896964	6.248130549	52.16273318	1.3E-09	2.42E-08
**ENSG00000147408**	CSGALNACT1	-0.762140489	6.049468005	52.15022655	1.3E-09	2.42E-08
**ENSG00000186812**	ZNF397	-0.849386548	5.93174424	52.1464206	1.3E-09	2.42E-08
**ENSG00000163191**	S100A11	1.385596708	6.588814941	63.96132181	1.36E-09	2.52E-08
**ENSG00000122042**	UBL3	0.64945813	6.833420239	51.83967111	1.41E-09	2.61E-08
**ENSG00000247774**	PCED1B-AS1	-0.530681299	7.420240556	51.83879308	1.41E-09	2.61E-08
**ENSG00000248019**	FAM13A-AS1	-0.977436222	5.556385232	51.80277773	1.43E-09	2.63E-08
**ENSG00000228506**		-1.114740453	4.794390514	51.73251961	1.46E-09	2.68E-08
**ENSG00000204271**	SPIN3	-1.061965081	4.963567791	51.66830559	1.48E-09	2.72E-08
**ENSG00000176155**	CCDC57	-1.321980203	5.097533619	54.30633715	1.49E-09	2.73E-08
**ENSG00000143409**	FAM63A	-1.171302878	4.752167287	51.6338919	1.49E-09	2.74E-08
**ENSG00000102572**	STK24	0.454894984	7.435160336	51.60363797	1.51E-09	2.75E-08
**ENSG00000135164**	DMTF1	-0.667614627	7.750322382	53.59765668	1.52E-09	2.78E-08
**ENSG00000213930**	GALT	-1.143725011	6.239469772	58.5312817	1.56E-09	2.84E-08
**ENSG00000150637**	CD226	0.493868758	7.886882692	51.3945084	1.59E-09	2.9E-08
**ENSG00000109929**	SC5D	0.869210589	5.366446477	51.39079997	1.59E-09	2.9E-08
**ENSG00000004468**	CD38	-1.930593573	3.949439446	52.57591061	1.63E-09	2.96E-08
**ENSG00000144597**	EAF1	0.736415494	5.659247965	51.26929371	1.65E-09	2.98E-08
**ENSG00000135362**	PRR5L	1.49366055	4.951089538	56.84332025	1.72E-09	3.1E-08
**ENSG00000125124**	BBS2	-0.67765707	6.101241022	51.09168102	1.73E-09	3.12E-08
**ENSG00000178951**	ZBTB7A	0.737926237	6.529945486	51.24384399	1.8E-09	3.24E-08
**ENSG00000035115**	SH3YL1	-0.786629082	6.121822344	50.8814651	1.83E-09	3.28E-08
**ENSG00000070081**	LOC105376575	-0.904111502	6.418639629	54.10765632	1.83E-09	3.28E-08
**ENSG00000154229**	PRKCA	-0.670594301	7.246256682	51.46219125	1.99E-09	3.56E-08
**ENSG00000091527**	CDV3	0.507826165	8.061415053	50.53055186	2.01E-09	3.59E-08
**ENSG00000139631**	CSAD	-1.870747495	4.772992259	57.37637066	2.01E-09	3.59E-08
**ENSG00000122085**	MTERF4	-0.604357903	6.642533106	50.51084732	2.02E-09	3.6E-08
**ENSG00000134046**	MBD2	0.58353898	7.02411145	50.43243983	2.07E-09	3.67E-08
**ENSG00000184371**	CSF1	1.360507786	4.219094517	50.41228945	2.08E-09	3.69E-08
**ENSG00000172766**	NAA16	-0.766348871	6.591926579	51.2144546	2.13E-09	3.78E-08

**ENSG00000196352**	CD55	-0.602643461	6.780832768	50.29647823	2.14E-09	3.79E-08
**ENSG00000102265**	TIMP1	1.137721315	4.657615122	50.2565564	2.17E-09	3.83E-08
**ENSG00000238197**	PAXBP1-AS1	-1.37373688	4.16575692	50.17705389	2.21E-09	3.9E-08
**ENSG00000198690**	FAN1	-0.791906088	5.895110093	50.11213655	2.25E-09	3.97E-08
**ENSG00000272849**		-1.675122119	4.44687814	52.54880957	2.29E-09	4.02E-08
**ENSG00000186815**	TPCN1	-0.925845688	5.859447876	50.96491244	2.32E-09	4.07E-08
**ENSG00000153214**	TMEM87B	0.568170969	6.405747388	49.87515408	2.4E-09	4.21E-08
**ENSG00000231160**	KLF3-AS1	-1.410252129	4.22586348	49.86817734	2.41E-09	4.21E-08
**ENSG00000108582**	CPD	0.574790139	6.381043573	49.84620621	2.42E-09	4.23E-08
**ENSG00000186470**	BTN3A2	-0.524514387	7.543827809	49.82172072	2.44E-09	4.25E-08
**ENSG00000177034**	MTX3	-0.769716199	6.158955191	49.77134758	2.47E-09	4.3E-08
**ENSG00000204217**	BMPR2	0.711407912	6.356758024	49.70870695	2.52E-09	4.37E-08
**ENSG00000147324**	MFHAS1	0.697481587	6.430223766	49.69352036	2.53E-09	4.38E-08
**ENSG00000175727**	MLXIP	-0.615544046	6.912429229	49.67762283	2.54E-09	4.39E-08
**ENSG00000156875**	MFSD14A	0.636944532	6.173159176	49.65417236	2.55E-09	4.41E-08
**ENSG00000162241**	SLC25A45	-1.381480724	4.775618681	50.99626908	2.59E-09	4.47E-08
**ENSG00000132694**	ARHGEF11	-1.009607659	5.095348908	49.56720475	2.62E-09	4.51E-08
**ENSG00000129422**	MTUS1	-1.761507998	4.06375663	49.54380398	2.63E-09	4.53E-08
**ENSG00000166046**	TCP11L2	-0.65163942	6.582514416	49.53527564	2.64E-09	4.53E-08
**ENSG00000180376**	CCDC66	-0.527365668	7.034213177	49.48883422	2.67E-09	4.58E-08
**ENSG00000183718**	TRIM52	-0.654570895	6.483568055	49.47085075	2.68E-09	4.6E-08
**ENSG00000165699**	TSC1	-0.681007244	6.307898662	49.46000045	2.69E-09	4.6E-08
**ENSG00000027697**	IFNGR1	0.808750948	5.531877558	49.39609911	2.74E-09	4.67E-08
**ENSG00000204161**	C10orf128	1.232550632	4.400633209	49.38525667	2.75E-09	4.68E-08
**ENSG00000198000**	NOL8	-0.629786449	6.874033859	49.33966616	2.78E-09	4.73E-08
**ENSG00000234961**		0.615994539	8.264958058	50.8119294	2.81E-09	4.77E-08
**ENSG00000142669**	SH3BGRL3	1.074151716	7.447366248	59.52549344	2.86E-09	4.85E-08
**ENSG00000161912**	ADCY10P1	-1.470540503	4.780662084	51.9795319	2.9E-09	4.91E-08
**ENSG00000111203**	LOC100507424	-0.780949372	5.834302104	49.14092847	2.94E-09	4.97E-08
**ENSG00000127483**	HP1BP3	-0.476281283	8.188009992	49.02470095	3.03E-09	5.12E-08
**ENSG00000258377**		0.797871581	5.301600432	49.01303607	3.04E-09	5.13E-08
**ENSG00000280194**		1.222311686	4.805708673	50.00997679	3.06E-09	5.14E-08
**ENSG00000085719**	CPNE3	0.618722968	6.674755798	48.82456654	3.21E-09	5.38E-08
**ENSG00000123416**	TUBA1B	0.734388879	6.316492891	48.82170541	3.21E-09	5.38E-08
**ENSG00000158470**	B4GALT5	1.218357597	4.583619103	48.72862269	3.29E-09	5.51E-08
**ENSG00000204576**	PRR3	-1.041308282	4.598647641	48.65224661	3.36E-09	5.62E-08
**ENSG00000133460**	SLC2A11	-1.479175283	4.200970384	48.63424876	3.38E-09	5.64E-08

**ENSG00000162980**	ARL5A	0.658842323	6.477832874	48.46057535	3.55E-09	5.9E-08
**ENSG00000064607**	SUGP2	-1.429545169	6.618215163	59.07268749	3.55E-09	5.9E-08
**ENSG00000101966**	XIAP	0.554459389	6.815128512	48.37731083	3.63E-09	6.02E-08
**ENSG00000199631**	SNORD33	1.939126305	4.19731121	53.71951099	3.63E-09	6.02E-08
**ENSG00000172543**	CTSW	1.098159763	4.775206781	48.32798392	3.68E-09	6.09E-08
**ENSG00000108175**	ZMIZ1	0.725487343	5.675924848	48.25345745	3.75E-09	6.2E-08
**ENSG00000142102**	PGGHG	-2.445415431	6.487518709	59.25313766	3.84E-09	6.33E-08
**ENSG00000145819**	ARHGAP26	0.707875744	5.962693534	48.15628208	3.86E-09	6.35E-08
**ENSG00000168214**	RBPJ	0.510459644	7.00938428	48.13453103	3.88E-09	6.38E-08
**ENSG00000255026**		-2.19442932	4.394988043	54.90758141	3.98E-09	6.52E-08
**ENSG00000163660**	CCNL1	-0.997504697	8.496071028	57.81743173	3.98E-09	6.52E-08
**ENSG00000163596**	ICA1L	-1.351394049	4.453724175	47.98293974	4.05E-09	6.62E-08
**ENSG00000114812**	VIPR1	-0.878446352	5.361871096	47.95162085	4.08E-09	6.67E-08
**ENSG00000168282**	mgat2	0.78848449	5.306828135	47.92852294	4.11E-09	6.7E-08
**ENSG00000177119**	ANO6	0.574757275	6.323445785	47.8798981	4.16E-09	6.78E-08
**ENSG00000274292**		-1.291695231	4.759714295	48.4379624	4.18E-09	6.8E-08
**ENSG00000108518**	PFN1	0.69374654	8.286868904	51.83408853	4.2E-09	6.83E-08
**ENSG00000112394**	SLC16A10	-1.661529269	4.684273034	52.22905125	4.23E-09	6.86E-08
**ENSG00000105866**	SP4	0.511865132	7.094876113	47.80018218	4.26E-09	6.88E-08
**ENSG00000135473**	PAN2	-1.507388025	5.681065806	56.40800337	4.26E-09	6.88E-08
**ENSG00000197077**	KIAA1671	1.405955091	4.050456302	47.78110666	4.28E-09	6.91E-08
**ENSG00000235162**	C12orf75	1.166924671	4.652508469	47.73431808	4.34E-09	6.99E-08
**ENSG00000107742**	SPOCK2	0.486222731	9.010286122	47.6464489	4.44E-09	7.15E-08
**ENSG00000101265**	RASSF2	0.561094315	6.825778398	47.56945568	4.54E-09	7.29E-08
**ENSG00000181788**	SIAH2	0.988571391	4.845550519	47.41385926	4.74E-09	7.61E-08
**ENSG00000258728**		-1.373695242	5.007752229	50.45073044	4.83E-09	7.74E-08
**ENSG00000069849**	ATP1B3	0.881396192	4.862422619	47.28440349	4.92E-09	7.86E-08
**ENSG00000196821**	c6orf106	0.657685141	6.034394386	47.2311937	4.99E-09	7.97E-08
**ENSG00000128394**	APOBEC3F	0.920575453	4.580669301	47.22655481	5E-09	7.97E-08
**ENSG00000108510**	MED13	0.413840653	8.076479723	47.20865819	5.02E-09	7.99E-08
**ENSG00000140511**	HAPLN3	-1.394422694	5.096514912	51.33327904	5.05E-09	8.02E-08
**ENSG00000227372**	TP73-AS1	-1.176330956	4.432151126	47.18423376	5.06E-09	8.02E-08
**ENSG00000223745**		-1.513541536	6.035221924	56.7362945	5.11E-09	8.1E-08
**ENSG00000105887**	MTPN	0.548471816	7.413967952	47.05089528	5.25E-09	8.31E-08
**ENSG00000104419**	NDRG1	0.663468768	6.685876547	47.03903117	5.27E-09	8.32E-08
**ENSG00000188785**	ZNF548	-0.862141617	5.487132071	47.01263659	5.31E-09	8.37E-08
**ENSG00000196230**	TUBB	0.611693109	7.512598508	47.75546796	5.38E-09	8.48E-08

**ENSG00000113108**	MIR6831	-1.701998647	3.971991061	47.37541374	5.4E-09	8.49E-08
**ENSG00000115896**	PLCL1	-0.937980801	5.557969127	46.89519984	5.48E-09	8.61E-08
**ENSG00000118263**	KLF7	-0.750460676	6.042508858	46.81464886	5.61E-09	8.79E-08
**ENSG00000188735**	TMEM120B	0.57080441	6.711053745	46.80560988	5.62E-09	8.8E-08
**ENSG00000232656**	IDI2-AS1	1.463996559	3.560290115	46.75619586	5.7E-09	8.91E-08
**ENSG00000125817**	CENPB	0.982358688	4.658358697	46.70988422	5.78E-09	9.02E-08
**ENSG00000104852**	SNRNP70	-1.471802347	6.979632349	56.95698682	5.84E-09	9.1E-08
**ENSG00000196924**	FLNA	0.61337771	9.878377069	48.05691842	5.86E-09	9.11E-08
**ENSG00000189319**	FAM53B	0.661760441	6.073756931	46.6334791	5.9E-09	9.17E-08
**ENSG00000013441**	CLK1	-0.840430386	8.579621806	53.9431168	5.91E-09	9.17E-08
**ENSG00000174500**	GCSAM	-0.892350818	5.355740542	46.61897191	5.93E-09	9.18E-08
**ENSG00000168350**	DEGS2	-0.746939869	6.194852377	46.59168794	5.97E-09	9.24E-08
**ENSG00000165650**	PDZD8	0.658756495	6.157205929	46.55129644	6.04E-09	9.33E-08
**ENSG00000241839**	PLEKHO2	1.049067688	4.309549653	46.54738879	6.05E-09	9.33E-08
**ENSG00000110934**	BIN2	-0.400203896	8.038961331	46.49164947	6.14E-09	9.46E-08
**ENSG00000132965**	ALOX5AP	0.896363907	5.242732121	46.44657397	6.22E-09	9.57E-08
**ENSG00000004534**	RBM6	-0.770002417	7.483088805	51.09871648	6.28E-09	9.64E-08
**ENSG00000234420**	ZNF37BP	-1.020864198	6.303198118	51.68872617	6.3E-09	9.66E-08
**ENSG00000096746**	HNRNPH3	-0.413251246	7.88214844	46.39920499	6.31E-09	9.66E-08
**ENSG00000113971**	NPHP3	-1.143953223	6.500374159	54.12461922	6.39E-09	9.77E-08
**ENSG00000144579**	CTDSP1	0.622651398	6.115258072	46.3354394	6.42E-09	9.8E-08
**ENSG00000095794**	CREM	1.138077461	4.216029151	46.32211481	6.44E-09	9.82E-08
**ENSG00000170802**	FOXN2	0.546305662	7.305037083	46.27250798	6.54E-09	9.94E-08
**ENSG00000113384**	GOLPH3	0.681975485	6.311340897	46.27043786	6.54E-09	9.94E-08
**ENSG00000135926**	MIR6513	0.687228284	6.047307074	46.21140322	6.65E-09	1.01E-07
**ENSG00000006530**	AGK	-0.654982826	5.713433233	46.16220616	6.74E-09	1.02E-07
**ENSG00000227671**		-1.059683692	6.200197883	51.72517384	6.9E-09	1.04E-07
**ENSG00000125354**	38961	-0.441092666	8.565418415	46.06413379	6.93E-09	1.05E-07
**ENSG00000110047**	EHD1	0.516934247	6.906221677	46.05621529	6.95E-09	1.05E-07
**ENSG00000090975**	PITPNM2	-0.978493197	5.317198496	46.02561966	7.01E-09	1.06E-07
**ENSG00000170113**	NIPA1	0.716883902	5.084915249	45.95556469	7.15E-09	1.08E-07
**ENSG00000166582**	cenpv	-1.433142826	4.006452846	45.93296368	7.2E-09	1.08E-07
**ENSG00000109920**	FNBP4	-0.928258904	7.963235803	54.21183865	7.29E-09	1.09E-07
**ENSG00000119772**	DNMT3A	-0.580032173	6.469873974	45.8729506	7.32E-09	1.1E-07
**ENSG00000089775**	ZBTB25	-0.483605477	7.740760897	45.86218734	7.34E-09	1.1E-07
**ENSG00000013810**	TACC3	-0.707702498	6.074569733	45.80869579	7.45E-09	1.11E-07
**ENSG00000105576**	TNPO2	-0.844571511	5.171510119	45.79337298	7.49E-09	1.12E-07

**ENSG00000012822**	CALCOCO1	-0.828805608	6.059176374	46.27422718	7.5E-09	1.12E-07
**ENSG00000087074**	PPP1R15A	-1.378293398	4.914858178	48.08995643	7.52E-09	1.12E-07
**ENSG00000139190**	VAMP1	-1.065455928	5.78276166	49.24161726	7.61E-09	1.13E-07
**ENSG00000234745**	HLA-B	0.51458268	10.74339623	45.72958713	7.62E-09	1.13E-07
**ENSG00000112782**	CLIC5	1.273754012	4.463097766	45.71561592	7.65E-09	1.13E-07
**ENSG00000140400**	MAN2C1	-1.431291112	5.73482404	53.32520672	7.71E-09	1.14E-07
**ENSG00000077458**	FAM76B	-0.692754921	6.014078317	45.66645341	7.76E-09	1.15E-07
**ENSG00000117505**	DR1	0.450025371	7.647828799	45.66503043	7.76E-09	1.15E-07
**ENSG00000108773**	KAT2A	-1.359105779	4.652512577	46.58748568	7.78E-09	1.15E-07
**ENSG00000163492**	CCDC141	-1.331139042	5.673808363	51.6857089	7.83E-09	1.15E-07
**ENSG00000147010**	SH3KBP1	0.408453055	7.712345022	45.61621125	7.87E-09	1.16E-07
**ENSG00000135932**	CAB39	0.473377581	7.805593899	45.59523909	7.92E-09	1.16E-07
**ENSG00000259007**		0.951001645	5.094982801	45.51408112	8.1E-09	1.19E-07
**ENSG00000182179**	MIR5193	-1.042655304	6.298484237	51.14216959	8.22E-09	1.2E-07
**ENSG00000122435**	TRMT13	-0.972908716	5.947626826	48.16737403	8.27E-09	1.21E-07
**ENSG00000284564**		-1.452310535	5.861030922	53.90324819	8.28E-09	1.21E-07
**ENSG00000116001**	TIA1	-0.84973966	7.244973153	51.10550924	8.32E-09	1.21E-07
**ENSG00000126453**	BCL2L12	-1.162742784	4.323013181	45.39157735	8.39E-09	1.22E-07
**ENSG00000157570**	TSPAN18	0.56234628	6.170093433	45.37068819	8.44E-09	1.23E-07
**ENSG00000155096**	AZIN1	0.551295466	7.046671417	45.36402591	8.46E-09	1.23E-07
**ENSG00000143870**	PDIA6	0.71820967	6.215023382	45.33058819	8.54E-09	1.24E-07
**ENSG00000118418**	HMGN3	-1.090522402	4.937106581	45.21878178	8.82E-09	1.28E-07
**ENSG00000197343**	ZNF655	-0.615118995	7.558433203	46.20338579	8.99E-09	1.3E-07
**ENSG00000161381**	PLXDC1	-1.808631708	4.532600244	50.16984158	9.12E-09	1.32E-07
**ENSG00000175265**	GOLGA8A	-2.001596636	6.998243282	54.86686489	9.13E-09	1.32E-07
**ENSG00000167766**	ZNF83	-1.210789136	6.760516378	53.84037461	9.19E-09	1.32E-07
**ENSG00000235194**	PPP1R3E	-1.050290279	5.541973631	46.98318075	9.21E-09	1.32E-07
**ENSG00000126456**	IRF3	-0.984141758	5.718255947	46.92031086	9.25E-09	1.33E-07
**ENSG00000128699**	ORMDL1	-0.900330833	6.682958563	49.6382442	9.38E-09	1.34E-07
**ENSG00000153283**	CD96	0.411522798	8.306179117	44.95506886	9.51E-09	1.36E-07
**ENSG00000167005**	NUDT21	0.48843857	6.800191421	44.89133669	9.68E-09	1.38E-07
**ENSG00000215252**	GOLGA8B	-1.988122092	7.235499508	54.56700187	9.71E-09	1.38E-07
**ENSG00000206503**	hla-a	0.497311339	9.376646075	44.87869569	9.72E-09	1.38E-07
**ENSG00000205268**	PDE7A	-0.463728674	8.455319969	44.86578351	9.75E-09	1.39E-07
**ENSG00000153113**	CAST	0.423945662	8.721797923	44.86338364	9.76E-09	1.39E-07
**ENSG00000170989**	S1PR1	0.553661765	8.180889533	45.23666751	9.78E-09	1.39E-07
**ENSG00000280064**		1.470750329	4.308670649	46.21434268	1.01E-08	1.43E-07

**ENSG00000165792**	METTL17	-1.089666225	5.187220189	45.41201584	1.01E-08	1.44E-07
**ENSG00000050426**	LETMD1	-0.789705688	5.742887736	44.70025487	1.02E-08	1.45E-07
**ENSG00000259431**	THTPA	-1.103021622	4.864723678	44.69171156	1.03E-08	1.45E-07
**ENSG00000229619**	MBNL1-AS1	-0.705203745	6.809924699	45.68101045	1.03E-08	1.46E-07
**ENSG00000189050**	RNFT1	-0.954618135	4.858034785	44.65918577	1.03E-08	1.46E-07
**ENSG00000132294**	EFR3A	0.444634003	7.036601075	44.61161556	1.05E-08	1.47E-07
**ENSG00000271680**		-1.440921533	3.672120367	44.57505051	1.06E-08	1.49E-07
**ENSG00000204282**	TNRC6C-AS1	-0.756660977	6.410407868	45.25807714	1.08E-08	1.51E-07
**ENSG00000110876**	SELPLG	0.593646627	8.084279281	45.93472507	1.08E-08	1.51E-07
**ENSG00000167261**	DPEP2	-1.314443009	5.197580853	48.09259482	1.11E-08	1.55E-07
**ENSG00000114353**	GNAI2	0.528973192	7.672168697	44.34953783	1.13E-08	1.58E-07
**ENSG00000114857**	NKTR	-1.223705711	9.156192897	53.64791039	1.15E-08	1.6E-07
**ENSG00000174738**	NR1D2	0.510142099	7.177038069	44.24676474	1.17E-08	1.62E-07
**ENSG00000118816**	CCNI	0.522923459	9.034760619	44.24554667	1.17E-08	1.62E-07
**ENSG00000073331**	alpk1	-1.235978423	4.648617462	44.23374014	1.17E-08	1.62E-07
**ENSG00000170445**	HARS	-0.722513581	5.686348486	44.20953796	1.18E-08	1.63E-07
**ENSG00000178038**	ALS2CL	-2.053313428	4.704082675	51.41824191	1.19E-08	1.65E-07
**ENSG00000196154**	S100A4	1.406604725	6.62472006	53.33234869	1.19E-08	1.65E-07
**ENSG00000167615**	LENG8	-1.675606536	8.146518712	53.53256387	1.2E-08	1.66E-07
**ENSG00000115808**	STRN	0.525978294	6.82989353	44.13805193	1.2E-08	1.66E-07
**ENSG00000152332**	UHMK1	0.416418393	8.295436058	44.12357984	1.21E-08	1.66E-07
**ENSG00000148248**	SURF4	0.693270267	6.092597946	44.07877965	1.22E-08	1.68E-07
**ENSG00000154814**	OXNAD1	-0.546390035	7.85455766	44.06492374	1.23E-08	1.69E-07
**ENSG00000259976**	Mir568	0.535785654	7.183332375	44.01026949	1.25E-08	1.71E-07
**ENSG00000168209**	DDIT4	1.306193617	4.789477078	45.95576572	1.31E-08	1.8E-07
**ENSG00000061936**	SFSWAP	-0.702428663	6.710676823	44.55716195	1.34E-08	1.83E-07
**ENSG00000183891**	ttc32	-1.257901144	4.069518773	43.71702712	1.36E-08	1.85E-07
**ENSG00000163565**	IFI16	0.465824762	7.63741245	43.71678772	1.36E-08	1.85E-07
**ENSG00000111554**	MDM1	-0.915578224	4.752442686	43.7165856	1.36E-08	1.85E-07
**ENSG00000184208**	C22orf46	-1.029412709	4.226735245	43.666626	1.38E-08	1.88E-07
**ENSG00000169926**	KLF13	0.444176374	7.704646814	43.637013	1.39E-08	1.89E-07
**ENSG00000206190**	ATP10A	-0.768395254	5.827221607	43.63362854	1.39E-08	1.89E-07
**ENSG00000163516**	ANKZF1	-1.466305258	6.336536644	52.4147721	1.39E-08	1.89E-07
**ENSG00000007392**	LUC7L	-1.179658642	6.399475096	51.02999959	1.41E-08	1.92E-07
**ENSG00000076043**	REXO2	-0.873852327	5.169308752	43.56813754	1.42E-08	1.92E-07
**ENSG00000187742**	SECISBP2	-0.74623298	7.166260728	46.74237942	1.43E-08	1.93E-07
**ENSG00000135387**	CAPRIN1	0.377827515	8.057242604	43.53478403	1.43E-08	1.93E-07

**ENSG00000230084**		-1.404716264	4.719005611	45.3801867	1.45E-08	1.96E-07
**ENSG00000246790**		-1.056957622	4.664858306	43.48194321	1.45E-08	1.96E-07
**ENSG00000143443**	C1orf56	-0.496963039	6.679628425	43.46074364	1.46E-08	1.97E-07
**ENSG00000261864**		0.627516513	5.83825624	43.4436855	1.47E-08	1.97E-07
**ENSG00000236287**	ZBED5	-0.527544798	7.065199789	43.41491109	1.48E-08	1.99E-07
**ENSG00000122224**	LY9	-0.492711973	6.701443846	43.4086981	1.49E-08	1.99E-07
**ENSG00000168067**	MAP4K2	-0.702536062	6.130791586	43.28763532	1.54E-08	2.06E-07
**ENSG00000183864**	tob2	0.694655391	5.571615506	43.27626597	1.54E-08	2.06E-07
**ENSG00000170340**	B3GNT2	0.732650813	5.04735941	43.27192999	1.55E-08	2.06E-07
**ENSG00000204650**	CRHR1-IT1	-0.80338199	5.220418233	43.19141129	1.58E-08	2.11E-07
**ENSG00000173442**	EHBP1L1	-0.741900248	6.076310986	43.16317253	1.6E-08	2.12E-07
**ENSG00000273559**	CWC25	-0.867724648	5.231824375	43.08979418	1.63E-08	2.17E-07
**ENSG00000140199**	SLC12A6	-0.416970777	7.516824451	43.04019126	1.65E-08	2.2E-07
**ENSG00000170006**	TMEM154	0.61353969	6.252440464	42.96730729	1.69E-08	2.24E-07
**ENSG00000244754**	N4BP2L2	-0.487050995	8.677170004	42.84767515	1.75E-08	2.32E-07
**ENSG00000109452**	INPP4B	0.480524623	9.072233705	42.82125925	1.76E-08	2.33E-07
**ENSG00000102710**	SUPT20H	-0.448509171	7.297709365	42.76723039	1.79E-08	2.37E-07
**ENSG00000026297**	RNASET2	-0.506597543	7.296621022	42.65746667	1.85E-08	2.44E-07
**ENSG00000111775**	Cox6a1	0.889795983	5.143954305	42.5093229	1.93E-08	2.54E-07
**ENSG00000278050**		-1.691341201	5.348295746	50.07671261	1.93E-08	2.54E-07
**ENSG00000170385**	SLC30A1	0.906633179	5.007840423	42.49669512	1.94E-08	2.55E-07
**ENSG00000139289**	PHLDA1	1.127903645	4.266137903	42.46676023	1.96E-08	2.57E-07
**ENSG00000124181**	PLCG1	-0.516814373	8.052480549	42.44795485	1.97E-08	2.58E-07
**ENSG00000105486**	LIG1	-0.802335221	5.595381005	42.42305244	1.98E-08	2.59E-07
**ENSG00000114529**	c3orf52	-1.463698712	4.018699133	42.42297675	1.98E-08	2.59E-07
**ENSG00000147454**	SLC25A37	-1.055077198	5.189389633	43.21337725	2.01E-08	2.63E-07
**ENSG00000212443**	SNORA53	2.796562574	5.152437887	50.97246501	2.04E-08	2.66E-07
**ENSG00000110888**	CAPRIN2	-0.96033819	5.952609769	45.16550051	2.06E-08	2.68E-07
**ENSG00000064666**	CNN2	0.518340413	8.02143245	42.2882307	2.06E-08	2.68E-07
**ENSG00000185697**	MYBL1	1.209532926	6.900642375	50.62015282	2.06E-08	2.68E-07
**ENSG00000129347**	KRI1	-0.629326395	5.852602398	42.22009386	2.1E-08	2.73E-07
**ENSG00000137449**	CPEB2	0.776055244	5.461884083	42.18102778	2.13E-08	2.76E-07
**ENSG00000183813**	CCR4	0.929772142	6.599340738	47.24223848	2.18E-08	2.82E-07
**ENSG00000155363**	MOV10	-0.69234936	5.621665082	42.09511843	2.18E-08	2.82E-07
**ENSG00000134186**	PRPF38B	-0.474620904	8.010777935	42.0756343	2.2E-08	2.83E-07
**ENSG00000171316**	CHD7	-0.605794163	6.773598878	42.06593427	2.2E-08	2.84E-07
**ENSG00000107938**	EDRF1	-0.700203732	6.31854255	41.94597389	2.28E-08	2.94E-07

**ENSG00000110367**	DDX6	0.406380501	9.183961752	41.93260248	2.29E-08	2.94E-07
**ENSG00000111669**	TPI1	0.761288185	5.641685703	41.93034618	2.29E-08	2.94E-07
**ENSG00000166888**	STAT6	-0.583716554	7.061101392	41.926841	2.29E-08	2.94E-07
**ENSG00000154153**	fam134b	-0.689277977	5.460180541	41.89788547	2.31E-08	2.97E-07
**ENSG00000137845**	ADAM10	0.400459418	8.151147562	41.7863023	2.39E-08	3.06E-07
**ENSG00000070010**	UFD1L	-0.66470473	5.5973334	41.72957907	2.43E-08	3.11E-07
**ENSG00000159658**	EFCAB14	0.463044068	7.942575632	41.72233991	2.44E-08	3.11E-07
**ENSG00000111364**	DDX55	-0.788888596	5.266968306	41.7154313	2.44E-08	3.11E-07
**ENSG00000197956**	S100A6	0.807683014	5.898144066	42.49472812	2.44E-08	3.11E-07
**ENSG00000126267**	Cox6b1	0.833735699	5.223375116	41.70551156	2.45E-08	3.12E-07
**ENSG00000102910**	LOC100507577	-0.424129547	7.575265587	41.63016099	2.51E-08	3.18E-07
**ENSG00000101104**	PABPC1L	-1.720934417	4.84838561	47.31621182	2.53E-08	3.21E-07
**ENSG00000169018**	FEM1B	0.454183538	6.94347661	41.58358613	2.54E-08	3.22E-07
**ENSG00000181472**	ZBTB2	0.618375769	5.45690172	41.56710166	2.55E-08	3.23E-07
**ENSG00000134371**	CDC73	0.42928574	6.970113998	41.54509222	2.57E-08	3.25E-07
**ENSG00000115548**	KDM3A	-0.521727593	7.520140189	41.49306987	2.61E-08	3.3E-07
**ENSG00000203667**	cox20	-0.81248473	7.170138825	46.11050714	2.67E-08	3.36E-07
**ENSG00000107738**	c10orf54	0.588113163	6.452788676	41.41666791	2.67E-08	3.36E-07
**ENSG00000104957**	CCDC130	-1.096215527	4.814060374	41.35298462	2.72E-08	3.42E-07
**ENSG00000176463**	SLCO3A1	0.791732176	5.689766331	41.35120186	2.72E-08	3.42E-07
**ENSG00000147138**	GPR174	0.666627092	6.005346953	41.34207933	2.73E-08	3.43E-07
**ENSG00000212232**	Snord17	2.784027706	5.865594643	49.58606041	2.75E-08	3.45E-07
**ENSG00000108474**	PIGL	-1.169881642	5.210165451	43.59736012	2.76E-08	3.45E-07
**ENSG00000111271**	ACAD10	-1.014345368	4.823288592	41.22207206	2.83E-08	3.54E-07
**ENSG00000081019**	RSBN1	0.43595134	7.377548842	41.12105018	2.91E-08	3.64E-07
**ENSG00000121579**	NAA50	0.510808035	6.437359592	41.08492054	2.95E-08	3.67E-07
**ENSG00000112305**	SMAP1	0.622701154	6.032872864	41.0821751	2.95E-08	3.67E-07
**ENSG00000115762**	plekhb2	0.537729938	6.153558754	41.02166762	3E-08	3.74E-07
**ENSG00000162231**	NXF1	-0.69012712	6.793859145	42.01979344	3.01E-08	3.74E-07
**ENSG00000168175**	MAPK1IP1L	0.455019792	7.345867352	40.96017852	3.06E-08	3.79E-07
**ENSG00000198625**	MDM4	-0.464187561	8.467394683	40.95627257	3.06E-08	3.79E-07
**ENSG00000134909**	ARHGAP32	-1.490234903	4.068619827	40.95579007	3.06E-08	3.79E-07
**ENSG00000166340**	TPP1	0.483191912	7.376319577	40.94623269	3.07E-08	3.8E-07
**ENSG00000117500**	TMED5	0.478531579	7.346852782	40.94460351	3.07E-08	3.8E-07
**ENSG00000105662**	CRTC1	-1.030317104	4.597611123	40.84933694	3.16E-08	3.9E-07
**ENSG00000013573**	DDX11	-1.109793065	4.708589002	40.83393251	3.18E-08	3.92E-07
**ENSG00000086598**	TMED2	0.669560513	6.567086734	41.35258082	3.2E-08	3.94E-07

**ENSG00000139187**	KLRG1	1.071777727	5.567220841	44.09344783	3.2E-08	3.94E-07
**ENSG00000232810**	TNF	1.745962694	4.111093442	44.10178955	3.24E-08	3.98E-07
**ENSG00000119723**	COQ6	-0.985129869	4.81352461	40.75156167	3.25E-08	3.99E-07
**ENSG00000128159**	TUBGCP6	-1.142466771	6.365018785	47.33313323	3.27E-08	4.01E-07
**ENSG00000185684**	EP400NL	-0.95766013	5.266503908	40.76691699	3.3E-08	4.04E-07
**ENSG00000177479**	ARIH2	-0.701566699	6.310547669	40.72462971	3.35E-08	4.1E-07
**ENSG00000122694**	GLIPR2	0.868414511	4.990767964	40.62220746	3.38E-08	4.13E-07
**ENSG00000283228**		0.817857884	5.107757979	40.57950038	3.43E-08	4.18E-07
**ENSG00000143493**	INTS7	0.849257783	4.974896391	40.57702032	3.43E-08	4.18E-07
**ENSG00000126790**	L3HYPDH	-1.260780332	4.187243442	40.56429233	3.44E-08	4.19E-07
**ENSG00000139116**	KIF21A	0.794494951	5.622946937	40.50072222	3.51E-08	4.27E-07
**ENSG00000157306**		-1.004497253	5.186314349	40.61061561	3.58E-08	4.35E-07
**ENSG00000136986**	DERL1	0.587240101	6.250941261	40.28471986	3.74E-08	4.54E-07
**ENSG00000138439**	FAM117B	-0.845193433	6.462433518	43.46746982	3.77E-08	4.57E-07
**ENSG00000170027**	YWHAG	0.582954129	6.101768874	40.25812264	3.77E-08	4.57E-07
**ENSG00000132825**	PPP1R3D	0.913301731	4.265570657	40.24574637	3.79E-08	4.58E-07
**ENSG00000143198**	MGST3	0.728284535	5.22247534	40.15594072	3.89E-08	4.7E-07
**ENSG00000228606**		-1.237161007	3.802784396	40.1501128	3.9E-08	4.7E-07
**ENSG00000258017**		0.709151853	6.029743104	40.1349714	3.92E-08	4.72E-07
**ENSG00000104365**	IKBKB	-0.695744148	6.658908677	40.89847459	4.05E-08	4.87E-07
**ENSG00000127511**	SIN3B	-0.911843928	5.681689284	41.10224653	4.06E-08	4.88E-07
**ENSG00000125772**	GPCPD1	-0.637504041	7.121384786	41.06271798	4.11E-08	4.93E-07
**ENSG00000235079**		-1.03259706	4.842433766	39.9500002	4.14E-08	4.97E-07
**ENSG00000113240**	CLK4	-0.772557693	6.833601293	42.95365468	4.15E-08	4.97E-07
**ENSG00000157800**	SLC37A3	-0.755368558	4.814704993	39.87917293	4.23E-08	5.06E-07
**ENSG00000134453**	RBM17	-0.588040683	6.63462865	39.87776479	4.23E-08	5.06E-07
**ENSG00000170540**	ARL6IP1	0.571850683	6.905229308	39.83703212	4.28E-08	5.11E-07
**ENSG00000213015**	ZNF580	-1.215218723	4.445051255	39.79961223	4.33E-08	5.17E-07
**ENSG00000221420**	Snora81	1.743790564	4.977844816	46.50884393	4.34E-08	5.17E-07
**ENSG00000168785**	TSPAN5	0.889654863	4.737015195	39.78019402	4.36E-08	5.19E-07
**ENSG00000197635**	DPP4	0.621078291	7.180622773	41.13784023	4.36E-08	5.19E-07
**ENSG00000170296**	GABARAP	0.870019102	5.812427609	41.45613005	4.38E-08	5.19E-07
**ENSG00000166326**	TRIM44	-0.508045078	6.91333891	39.76032959	4.39E-08	5.19E-07
**ENSG00000240053**	LY6G5B	-1.424422428	4.448465231	40.6403104	4.39E-08	5.19E-07
**ENSG00000270362**	HMGN3-AS1	-1.281545685	3.616098375	39.75321575	4.39E-08	5.2E-07
**ENSG00000116574**	RHOU	1.30884283	4.142426315	39.81706108	4.42E-08	5.22E-07
**ENSG00000135720**	DYNC1LI2	-0.540239483	6.413648755	39.71487619	4.45E-08	5.25E-07

**ENSG00000164331**	ANKRA2	-0.911360376	5.198096267	39.70294565	4.46E-08	5.26E-07
**ENSG00000266338**	NBPF15	-0.797805433	6.193745258	41.22095129	4.46E-08	5.26E-07
**ENSG00000198730**	CTR9	0.48287174	7.058229999	39.64359483	4.54E-08	5.34E-07
**ENSG00000144746**	ARL6IP5	0.574639145	8.02210462	41.16028689	4.57E-08	5.37E-07
**ENSG00000272980**		0.624556197	6.082225467	39.60701261	4.59E-08	5.39E-07
**ENSG00000140299**	BNIP2	0.468987412	7.395954318	39.55707768	4.66E-08	5.47E-07
**ENSG00000172795**	DCP2	0.406383535	7.5349972	39.490058	4.76E-08	5.57E-07
**ENSG00000153560**	UBP1	-0.482592649	6.88067505	39.41706438	4.87E-08	5.69E-07
**ENSG00000221978**	CCNL2	-1.380458519	7.258955312	46.92187606	4.89E-08	5.71E-07
**ENSG00000154642**	c21orf91	0.589318188	6.938724865	39.47903475	4.9E-08	5.72E-07
**ENSG00000196199**	MPHOSPH8	-0.443525326	7.776490885	39.35811719	4.95E-08	5.77E-07
**ENSG00000167264**	dus2	-1.288766694	3.939282118	39.34395191	4.98E-08	5.79E-07
**ENSG00000275183**	LENG9	-1.453286865	4.454052098	40.90792416	5.1E-08	5.93E-07
**ENSG00000109756**	RAPGEF2	0.678043232	5.648290809	39.22033675	5.17E-08	6E-07
**ENSG00000162511**	LAPTM5	0.456050793	9.537797905	39.17910221	5.23E-08	6.07E-07
**ENSG00000112486**	CCR6	1.065959062	5.667197773	43.12799069	5.25E-08	6.08E-07
**ENSG00000077147**	TM9SF3	0.444461308	7.593678041	39.14298904	5.29E-08	6.12E-07
**ENSG00000106733**	NMRK1	-0.673406283	5.842275717	39.09606926	5.36E-08	6.21E-07
**ENSG00000164574**	GALNT10	0.67663413	5.723394644	39.08665238	5.38E-08	6.21E-07
**ENSG00000147162**	OGT	-1.030392424	9.744077679	46.27949069	5.38E-08	6.21E-07
**ENSG00000198380**	GFPT1	0.619488522	6.304631947	39.03005958	5.47E-08	6.31E-07
**ENSG00000197471**	SPN	0.652574822	8.248633766	42.40872255	5.55E-08	6.39E-07
**ENSG00000136450**	SRSF1	-0.388670992	7.728080726	38.97520895	5.57E-08	6.4E-07
**ENSG00000033867**	SLC4A7	0.400513017	7.801927394	38.96390219	5.59E-08	6.42E-07
**ENSG00000265206**	MIR142	-1.564761322	5.127683643	44.50551323	5.64E-08	6.47E-07
**ENSG00000141458**	NPC1	0.587121127	6.529241643	38.92864117	5.65E-08	6.47E-07
**ENSG00000171681**	ATF7IP	0.345784255	8.992066208	38.90550568	5.69E-08	6.51E-07
**ENSG00000102781**	KATNAL1	0.981774388	4.831307847	38.87656062	5.74E-08	6.56E-07
**ENSG00000131368**	MRPS25	-0.688879376	6.166690468	38.87312625	5.74E-08	6.56E-07
**ENSG00000124783**	SSR1	0.458025882	8.044340731	38.83690876	5.81E-08	6.63E-07
**ENSG00000108469**	RECQL5	-1.084883677	4.14957163	38.7290187	6E-08	6.84E-07
**ENSG00000142687**	KIAA0319L	-0.6721777	6.056724882	38.67025025	6.11E-08	6.96E-07
**ENSG00000167895**	tmc8	-0.814020772	8.103362949	44.39977671	6.21E-08	7.07E-07
**ENSG00000034677**	RNF19A	0.438804614	7.200329187	38.60281415	6.24E-08	7.09E-07
**ENSG00000073921**	PICALM	0.423045453	7.409710231	38.58367373	6.27E-08	7.12E-07
**ENSG00000277599**		-1.474434323	5.842183382	45.37309673	6.28E-08	7.13E-07
**ENSG00000283958**		1.976852009	4.872021173	45.40649722	6.34E-08	7.18E-07

**ENSG00000002834**	LASP1	0.48192122	6.896848179	38.54736001	6.34E-08	7.18E-07
**ENSG00000132199**	ENOSF1	-1.100255271	5.942327648	42.97800493	6.51E-08	7.36E-07
**ENSG00000167785**	ZNF558	-1.0296254	4.778655947	38.43161733	6.57E-08	7.42E-07
**ENSG00000102901**	CENPT	-1.043005683	5.274092617	39.65127929	6.69E-08	7.55E-07
**ENSG00000250264**		-0.784484362	5.275046337	38.35666892	6.73E-08	7.58E-07
**ENSG00000166913**	YWHAB	0.400028344	8.694845629	38.34789476	6.74E-08	7.59E-07
**ENSG00000171791**	BCL2	0.56970621	8.840638647	40.11019012	6.76E-08	7.6E-07
**ENSG00000160679**	CHTOP	-0.524816786	6.755504275	38.3073609	6.83E-08	7.67E-07
**ENSG00000100612**	DHRS7	0.695938353	5.657282806	38.29587118	6.85E-08	7.69E-07
**ENSG00000172932**	ANKRD13D	-0.732100298	5.925066489	38.22149683	7.01E-08	7.86E-07
**ENSG00000100099**	HPS4	-0.79081259	5.615156643	38.20976889	7.04E-08	7.88E-07
**ENSG00000143933**	CALM2	0.477733823	7.468573483	38.10912362	7.26E-08	8.12E-07
**ENSG00000226232**		-1.507346973	3.682238991	38.10125314	7.28E-08	8.13E-07
**ENSG00000070061**	IKBKAP	-0.627695177	6.183323523	38.08813421	7.31E-08	8.15E-07
**ENSG00000100519**	PSMC6	-0.571975723	6.239283183	38.0839689	7.32E-08	8.15E-07
**ENSG00000121774**	KHDRBS1	0.436749687	7.041232203	38.03215946	7.43E-08	8.28E-07
**ENSG00000119714**	GPR68	1.089205517	3.807975304	38.02512388	7.45E-08	8.29E-07
**ENSG00000126822**	PLEKHG3	0.881726343	5.79298388	39.85834229	7.58E-08	8.42E-07
**ENSG00000277194**	Snord22	2.985427546	5.360051924	44.90008353	7.78E-08	8.63E-07
**ENSG00000056097**	ZFR	0.426372746	7.487198597	37.88282474	7.78E-08	8.63E-07
**ENSG00000196670**	ZFP62	-0.75262435	5.38786955	37.85993629	7.84E-08	8.68E-07
**ENSG00000082701**	GSK3B	0.495666977	6.81581239	37.84297971	7.88E-08	8.72E-07
**ENSG00000243811**	APOBEC3D	0.726449031	5.245167467	37.83916478	7.89E-08	8.72E-07
**ENSG00000172775**	FAM192A	-0.555508222	6.185881574	37.78774837	8.02E-08	8.85E-07
**ENSG00000170881**	RNF139	0.573675008	5.825174716	37.75655892	8.09E-08	8.93E-07
**ENSG00000174013**	FBXO45	0.863904156	4.522567578	37.75061512	8.11E-08	8.93E-07
**ENSG00000189007**	ADAT2	-1.409456197	4.830814159	41.06184863	8.12E-08	8.93E-07
**ENSG00000214894**	LINC00243	-1.141800412	4.188883308	37.74601834	8.12E-08	8.93E-07
**ENSG00000124831**	LRRFIP1	0.407996604	8.613206768	37.73563287	8.15E-08	8.95E-07
**ENSG00000198431**	TXNRD1	0.606287478	5.45594534	37.68272547	8.28E-08	9.09E-07
**ENSG00000145348**	TBCK	-0.651487731	6.287799422	37.67472668	8.3E-08	9.1E-07
**ENSG00000086015**	mast2	1.079620866	3.423919861	37.64838658	8.37E-08	9.16E-07
**ENSG00000004399**	PLXND1	1.658016722	4.726098262	42.68639515	8.44E-08	9.23E-07
**ENSG00000169660**	HEXDC	-1.369518767	4.804689705	40.24308564	8.68E-08	9.49E-07
**ENSG00000211751**		0.459244069	7.903630123	37.52381151	8.7E-08	9.49E-07
**ENSG00000198618**		1.022796366	4.526746978	37.51601394	8.72E-08	9.51E-07
**ENSG00000166483**	WEE1	1.275035528	4.186163382	37.55727597	8.95E-08	9.75E-07

**ENSG00000119688**	ABCD4	-0.889350435	5.19059297	37.41555211	8.99E-08	9.79E-07
**ENSG00000255185**		-1.312605226	4.425999958	37.75431194	9.02E-08	9.8E-07
**ENSG00000166532**	RIMKLB	-1.025246074	4.769060746	37.39869629	9.04E-08	9.82E-07
**ENSG00000262526**		0.777312395	6.113780899	38.90484032	9.08E-08	9.85E-07
**ENSG00000107890**	ANKRD26	-0.821481213	5.52900701	37.3763261	9.1E-08	9.87E-07
**ENSG00000138166**	DUSP5	1.33117297	3.734140134	37.36429807	9.14E-08	9.89E-07
**ENSG00000073861**	TBX21	1.693830074	4.068930932	40.5911266	9.2E-08	9.95E-07
**ENSG00000260729**		-1.105612809	4.101136893	37.32429543	9.25E-08	1E-06
**ENSG00000138757**	G3BP2	0.462378429	7.640972742	37.27439211	9.4E-08	1.01E-06
**ENSG00000033170**	FUT8	0.616927698	5.914160794	37.21871453	9.56E-08	1.03E-06
**ENSG00000155657**	TTN	-0.989240174	9.438197626	43.73124633	9.62E-08	1.04E-06
**ENSG00000178971**	CTC1	-0.693241777	7.533723176	40.49498909	9.65E-08	1.04E-06
**ENSG00000121413**	ZSCAN18	-1.149598523	4.518797827	37.1517843	9.76E-08	1.05E-06
**ENSG00000111011**	RSRC2	-0.508847337	7.563884908	37.12241656	9.85E-08	1.06E-06
**ENSG00000198087**	CD2AP	0.474860402	6.440734466	36.94112736	1.04E-07	1.12E-06
**ENSG00000188895**	MSL1	-0.564890551	6.676188494	36.90997795	1.05E-07	1.13E-06
**ENSG00000115091**	ACTR3	0.368392421	8.703008657	36.89046329	1.06E-07	1.13E-06
**ENSG00000076928**	LOC100505585	-0.995775324	8.296299704	43.30668726	1.07E-07	1.14E-06
**ENSG00000116747**	TROVE2	0.451633565	7.73276742	36.8441665	1.07E-07	1.15E-06
**ENSG00000242294**	STAG3L5P	-1.44222221	5.566418162	42.5510527	1.08E-07	1.15E-06
**ENSG00000160058**	BSDC1	-0.639445144	5.689978144	36.83266187	1.08E-07	1.15E-06
**ENSG00000187239**	FNBP1	0.344629182	8.652477188	36.7506532	1.11E-07	1.18E-06
**ENSG00000143337**	TOR1AIP1	0.46023159	7.120636499	36.74608996	1.11E-07	1.18E-06
**ENSG00000269926**		1.365070574	4.373519375	37.91067436	1.15E-07	1.22E-06
**ENSG00000134014**	ELP3	0.564857665	5.849430441	36.5817433	1.17E-07	1.24E-06
**ENSG00000165359**	INTS6L	-0.913295368	6.255953207	40.16452039	1.17E-07	1.24E-06
**ENSG00000090621**	PABPC4	-0.434505171	6.999686648	36.5669866	1.17E-07	1.24E-06
**ENSG00000072071**	ADGRL1	-1.048539808	4.846126777	36.71493672	1.17E-07	1.24E-06
**ENSG00000211801**		1.00805989	4.088994754	36.54581682	1.18E-07	1.25E-06
**ENSG00000138413**	idh1	1.110615975	4.425269896	36.51910494	1.19E-07	1.26E-06
**ENSG00000010610**	CD4	0.412835941	8.046490111	36.41942568	1.23E-07	1.3E-06
**ENSG00000068366**	ACSL4	0.666828749	5.984520006	36.40395473	1.23E-07	1.3E-06
**ENSG00000185862**	EVI2B	0.480937159	9.055471487	36.39869517	1.24E-07	1.3E-06
**ENSG00000145730**	PAM	0.797447371	5.221217806	36.35717336	1.25E-07	1.32E-06
**ENSG00000005020**	SKAP2	1.22147006	4.203484561	36.26002083	1.29E-07	1.36E-06
**ENSG00000100359**	SGSM3	-1.157315153	5.045120864	37.91873266	1.31E-07	1.37E-06
**ENSG00000101290**	CDS2	0.397230276	6.876496285	36.21550151	1.31E-07	1.37E-06

**ENSG00000023909**	GCLM	0.727760069	5.220667285	36.20897156	1.31E-07	1.37E-06
**ENSG00000134452**	FBXO18	-0.64547138	5.681245605	36.18908513	1.32E-07	1.38E-06
**ENSG00000166734**	CASC4	0.496172127	6.916240985	36.12456565	1.35E-07	1.41E-06
**ENSG00000166710**	B2M	0.57164591	11.45498781	36.81792699	1.35E-07	1.41E-06
**ENSG00000089053**	ANAPC5	-0.599958296	7.20330558	37.15359984	1.36E-07	1.42E-06
**ENSG00000162601**	MYSM1	-0.472677743	7.251798584	35.98250135	1.41E-07	1.47E-06
**ENSG00000012061**	ercc1	-0.79089349	4.990723524	35.92640108	1.43E-07	1.49E-06
**ENSG00000182841**	RRP7BP	-0.939435625	4.740091103	35.92061413	1.44E-07	1.49E-06
**ENSG00000011243**	AKAP8L	-0.780730252	5.785902241	36.16928893	1.44E-07	1.5E-06
**ENSG00000164086**	DUSP7	0.690075605	5.928555327	35.90278359	1.44E-07	1.5E-06
**ENSG00000223705**	NSUN5P1	-1.558634572	5.085946316	40.73403954	1.47E-07	1.52E-06
**ENSG00000171700**	RGS19	0.911597475	4.956759274	35.83207382	1.48E-07	1.53E-06
**ENSG00000106133**	NSUN5P2	-0.96770491	4.951080104	35.82305708	1.48E-07	1.53E-06
**ENSG00000108588**	CCDC47	0.599563196	6.010216744	35.81131325	1.49E-07	1.54E-06
**ENSG00000137070**	il11ra	-1.577650048	5.90077214	41.90549064	1.5E-07	1.55E-06
**ENSG00000172890**	NADSYN1	-0.752210619	6.240005821	37.06586803	1.52E-07	1.57E-06
**ENSG00000158805**	ZNF276	-0.55444747	7.020442459	35.73494219	1.52E-07	1.57E-06
**ENSG00000186432**	KPNA4	0.420983713	7.358239574	35.73357767	1.52E-07	1.57E-06
**ENSG00000179335**	CLK3	-0.575106816	6.550967448	35.72093338	1.53E-07	1.57E-06
**ENSG00000177707**	NECTIN3	1.215012732	3.650862896	35.69951944	1.54E-07	1.58E-06
**ENSG00000145476**	CYP4V2	-0.503490671	6.70911025	35.68148907	1.55E-07	1.59E-06
**ENSG00000152969**	JAKMIP1	1.099990732	3.732746592	35.67427966	1.55E-07	1.59E-06
**ENSG00000157259**	GATAD1	-0.991253866	4.989063497	35.76739721	1.56E-07	1.6E-06
**ENSG00000178028**	DMAP1	-0.892239011	5.524391643	36.51352312	1.57E-07	1.61E-06
**ENSG00000047188**	YTHDC2	-0.579004649	6.829231372	35.62694179	1.58E-07	1.61E-06
**ENSG00000232940**	HCG25	-1.278304205	3.732409314	35.62595129	1.58E-07	1.61E-06
**ENSG00000274272**		-1.198994059	5.888517002	40.63213395	1.59E-07	1.62E-06
**ENSG00000163820**	FYCO1	0.523720999	6.645061229	35.57215535	1.6E-07	1.64E-06
**ENSG00000108061**	SHOC2	0.486118425	6.788223405	35.55659373	1.61E-07	1.64E-06
**ENSG00000111605**	CPSF6	-0.479960086	7.69516193	35.55417893	1.61E-07	1.64E-06
**ENSG00000227678**		0.825742756	5.015127358	35.54186994	1.62E-07	1.65E-06
**ENSG00000154265**	ABCA5	-0.603843907	6.481386265	35.51330671	1.63E-07	1.66E-06
**ENSG00000183735**	TBK1	0.71541104	5.395692187	35.4990583	1.64E-07	1.66E-06
**ENSG00000089820**	ARHGAP4	-0.881857969	6.90742497	40.17792775	1.64E-07	1.66E-06
**ENSG00000084090**	STARD7	0.436216937	6.425758715	35.48489503	1.65E-07	1.67E-06
**ENSG00000112146**	FBXO9	-0.603779509	6.12098904	35.44010353	1.67E-07	1.69E-06
**ENSG00000176444**	CLK2	-0.700317983	5.951402118	35.40848774	1.69E-07	1.71E-06

**ENSG00000175455**	CCDC14	-0.980695197	6.300594945	39.77076591	1.69E-07	1.71E-06
**ENSG00000223509**		-1.042507749	4.70793672	35.3709645	1.71E-07	1.73E-06
**ENSG00000100345**	MYH9	0.395589871	10.53747687	35.32766303	1.73E-07	1.75E-06
**ENSG00000143624**	INTS3	-0.622646291	6.185020979	35.28605581	1.76E-07	1.77E-06
**ENSG00000147894**	C9orf72	-0.716412518	5.45311812	35.28224323	1.76E-07	1.77E-06
**ENSG00000102241**	HTATSF1	0.58790163	6.408923502	35.23905216	1.78E-07	1.79E-06
**ENSG00000131067**	GGT7	-1.417830292	4.067729642	35.53538292	1.8E-07	1.81E-06
**ENSG00000120437**	ACAT2	0.776815498	5.035379164	35.1751423	1.82E-07	1.82E-06
**ENSG00000046651**	OFD1	-0.512956609	7.043395784	35.17444239	1.82E-07	1.82E-06
**ENSG00000180096**	37135	-0.714761782	6.746968348	37.26758753	1.82E-07	1.83E-06
**ENSG00000237298**	TTN-AS1	-0.887761507	7.224403895	40.1850659	1.83E-07	1.83E-06
**ENSG00000169446**	MMGT1	0.622613187	6.085149711	35.10403977	1.86E-07	1.86E-06
**ENSG00000172345**	STARD5	-0.483746547	6.727349208	35.08651383	1.87E-07	1.87E-06
**ENSG00000173200**	PARP15	-1.027123637	6.956956252	40.70558352	1.88E-07	1.88E-06
**ENSG00000213918**	DNASE1	-0.880620516	5.977025059	37.37551662	1.88E-07	1.88E-06
**ENSG00000023445**	BIRC3	-0.542819482	8.520627382	36.40807544	1.88E-07	1.88E-06
**ENSG00000221963**	APOL6	0.461744049	8.373980522	35.0356112	1.9E-07	1.89E-06
**ENSG00000130787**	HIP1R	-0.974122814	4.955043137	35.0332872	1.9E-07	1.89E-06
**ENSG00000123091**	RNF11	0.661966465	5.577825932	35.02692592	1.91E-07	1.89E-06
**ENSG00000136111**	TBC1D4	-0.901839037	7.606896042	40.51622849	1.92E-07	1.9E-06
**ENSG00000133318**	RTN3	0.620267606	5.672130584	34.97254689	1.94E-07	1.92E-06
**ENSG00000064995**	TAF11	-0.811175752	4.664504406	34.95533588	1.95E-07	1.93E-06
**ENSG00000270231**	NBPF8	-0.86248671	5.554164185	35.60932342	1.96E-07	1.94E-06
**ENSG00000132718**	SYT11	1.040559524	4.778018269	35.21408443	1.97E-07	1.95E-06
**ENSG00000273749**	CYFIP1	0.761319505	5.048097076	34.82649624	2.03E-07	2.01E-06
**ENSG00000198265**	HELZ	0.326661669	8.381334024	34.7868092	2.06E-07	2.03E-06
**ENSG00000148110**	MFSD14B	0.564157575	5.767891596	34.7077063	2.11E-07	2.08E-06
**ENSG00000047662**	FAM184B	-0.81446746	5.650555386	35.1348751	2.11E-07	2.08E-06
**ENSG00000279296**		-1.289136152	6.900010552	40.51999187	2.14E-07	2.11E-06
**ENSG00000072818**	ACAP1	-0.777221111	7.443615544	38.97133258	2.16E-07	2.12E-06
**ENSG00000155876**	RRAGA	0.763374707	5.163263426	34.63773357	2.16E-07	2.12E-06
**ENSG00000131473**	ACLY	0.475336005	6.389929819	34.59748591	2.19E-07	2.15E-06
**ENSG00000198169**	ZNF251	-1.057675621	4.078074158	34.58118024	2.2E-07	2.15E-06
**ENSG00000180448**	ARHGAP45	-0.557104863	8.246505099	36.1127601	2.2E-07	2.15E-06
**ENSG00000010295**	IFFO1	-0.970672267	4.900890424	34.56129536	2.21E-07	2.16E-06
**ENSG00000115875**	SRSF7	-0.641719903	7.801685883	37.36140458	2.22E-07	2.17E-06
**ENSG00000112245**	PTP4A1	0.518764106	6.84433656	34.53880567	2.23E-07	2.18E-06

**ENSG00000229474**	PATL2	-1.012170392	4.739300323	34.52837737	2.24E-07	2.18E-06
**ENSG00000258875**		1.145884192	3.406811202	34.43779923	2.3E-07	2.24E-06
**ENSG00000070882**	OSBPL3	0.609293902	6.43836564	34.41249199	2.32E-07	2.26E-06
**ENSG00000107736**	CDH23	-1.401286215	4.441462992	35.84303971	2.34E-07	2.27E-06
**ENSG00000163605**	PPP4R2	0.414901024	6.856197104	34.38021136	2.35E-07	2.28E-06
**ENSG00000166398**	KIAA0355	-0.592802491	6.267735864	34.3660558	2.36E-07	2.29E-06
**ENSG00000272501**		-1.213123292	4.178724175	34.36204298	2.36E-07	2.29E-06
**ENSG00000164180**	TMEM161B	-0.684814204	6.15481705	34.62418858	2.36E-07	2.29E-06
**ENSG00000124209**	rab22a	0.499402145	6.642999081	34.35368795	2.37E-07	2.29E-06
**ENSG00000178188**	SH2B1	-1.118176082	4.761701958	34.98592559	2.37E-07	2.29E-06
**ENSG00000100288**	CHKB	-1.37896902	5.38779188	38.90606543	2.37E-07	2.29E-06
**ENSG00000188404**	SELL	-0.526236335	8.734160851	35.4336094	2.39E-07	2.31E-06
**ENSG00000125430**	HS3ST3B1	0.635001121	5.978179541	34.31542582	2.39E-07	2.31E-06
**ENSG00000137955**	RABGGTB	-0.68372617	6.549565217	35.39381203	2.41E-07	2.32E-06
**ENSG00000215908**		-0.896301012	5.624146994	35.62739283	2.43E-07	2.34E-06
**ENSG00000266173**	STRADA	-0.650754449	6.21613476	34.25475114	2.44E-07	2.35E-06
**ENSG00000075420**	FNDC3B	0.538667221	6.141042742	34.25109426	2.44E-07	2.35E-06
**ENSG00000132530**	XAF1	-1.269508243	7.332237724	39.97101889	2.47E-07	2.37E-06
**ENSG00000151702**	FLI1	0.345261116	7.458388314	34.16132166	2.52E-07	2.41E-06
**ENSG00000206149**	HERC2P9	-1.256328218	4.074052617	34.11510682	2.55E-07	2.45E-06
**ENSG00000168216**	LMBRD1	0.605431784	6.067213992	34.10625821	2.56E-07	2.45E-06
**ENSG00000221817**	PPP3CB-AS1	-0.8052287	5.04248682	34.0299083	2.63E-07	2.51E-06
**ENSG00000114978**	mob1a	0.419581019	8.141763598	33.95506675	2.69E-07	2.57E-06
**ENSG00000025156**	HSF2	-0.701226619	5.274986295	33.93760733	2.7E-07	2.58E-06
**ENSG00000207445**	SNORD15B	2.382388638	5.34805552	39.59901325	2.72E-07	2.59E-06
**ENSG00000263753**	LINC00667	-0.771263848	5.611340434	33.89562912	2.74E-07	2.61E-06
**ENSG00000204267**	tap2	-0.56721921	6.613121922	33.89554288	2.74E-07	2.61E-06
**ENSG00000132635**	PCED1A	-1.539361651	3.967166569	34.6178052	2.75E-07	2.62E-06
**ENSG00000204130**	RUFY2	-0.452861644	6.325468768	33.86011067	2.77E-07	2.64E-06
**ENSG00000283761**		0.474929435	6.510502592	33.82329904	2.81E-07	2.67E-06
**ENSG00000100567**	PSMA3	-0.503455298	6.688176807	33.81391646	2.81E-07	2.67E-06
**ENSG00000186908**	ZDHHC17	-0.650120425	6.198588195	33.81226599	2.82E-07	2.67E-06
**ENSG00000170322**	NFRKB	-0.625781907	6.100596239	33.80562061	2.82E-07	2.67E-06
**ENSG00000101082**	SLA2	0.502703595	5.840665815	33.80531648	2.82E-07	2.67E-06
**ENSG00000077454**	lrch4	-0.834591019	6.475447138	36.92150186	2.84E-07	2.68E-06
**ENSG00000145675**	PIK3R1	0.373855361	8.773393577	33.72310976	2.9E-07	2.74E-06
**ENSG00000158062**	UBXN11	0.667267016	7.95829355	37.20387415	2.9E-07	2.74E-06

**ENSG00000272053**		1.105265054	3.65623053	33.70613972	2.91E-07	2.75E-06
**ENSG00000163611**	SPICE1	-0.773397924	5.432347615	33.70283811	2.92E-07	2.75E-06
**ENSG00000221823**	PPP3R1	0.520864878	6.562435522	33.67939295	2.94E-07	2.77E-06
**ENSG00000198218**	QRICH1	-0.397467311	7.179876253	33.63589781	2.98E-07	2.8E-06
**ENSG00000213463**	SYNJ2BP	-0.733926281	5.925310369	34.01651248	3.01E-07	2.83E-06
**ENSG00000271856**	LINC01215	-0.832090273	4.925704142	33.59353002	3.02E-07	2.84E-06
**ENSG00000154845**	PPP4R1	0.623213709	5.567857907	33.55250351	3.06E-07	2.87E-06
**ENSG00000068308**	OTUD5	-0.654090721	5.66686355	33.52857958	3.09E-07	2.89E-06
**ENSG00000179119**	SPTY2D1	0.579444999	5.796102676	33.52141534	3.09E-07	2.9E-06
**ENSG00000183513**	COA5	-0.868593817	5.002459202	33.48269367	3.13E-07	2.93E-06
**ENSG00000180644**	PRF1	1.421435637	5.776009157	38.81762847	3.14E-07	2.94E-06
**ENSG00000023516**	AKAP11	0.385338336	8.128145324	33.36270637	3.26E-07	3.04E-06
**ENSG00000138767**	CNOT6L	0.388550392	8.607460168	33.34488478	3.28E-07	3.06E-06
**ENSG00000267002**		-1.140194702	4.438348059	33.34228692	3.28E-07	3.06E-06
**ENSG00000121966**	CXCR4	0.585364777	8.395838157	35.62041945	3.3E-07	3.07E-06
**ENSG00000204304**	PBX2	-0.685439693	6.199050464	33.81793549	3.3E-07	3.07E-06
**ENSG00000106609**	TMEM248	0.452834762	7.052289953	33.32076052	3.3E-07	3.07E-06
**ENSG00000037757**	mri1	-1.047267867	4.970937924	34.14564435	3.32E-07	3.08E-06
**ENSG00000155158**	TTC39B	-0.493177313	6.867610436	33.29756201	3.33E-07	3.09E-06
**ENSG00000198804**	COX1	0.566624517	13.34409756	33.29374429	3.33E-07	3.09E-06
**ENSG00000072778**	ACADVL	-1.179000921	5.246566979	36.13170342	3.34E-07	3.1E-06
**ENSG00000128563**	PRKRIP1	-0.873528099	4.643695038	33.26039672	3.37E-07	3.12E-06
**ENSG00000109062**	MIR3615	0.521581731	6.664493418	33.25275662	3.38E-07	3.12E-06
**ENSG00000258461**		-1.220131069	4.222099336	33.24240442	3.39E-07	3.13E-06
**ENSG00000254413**	CHKB-CPT1B	-1.270332673	5.976022322	38.26886262	3.4E-07	3.14E-06
**ENSG00000251474**	RPL32P3	-0.913145234	4.926517405	33.22579541	3.41E-07	3.14E-06
**ENSG00000136485**	DCAF7	0.464197926	6.87090019	33.22123888	3.41E-07	3.14E-06
**ENSG00000263934**	SNORD3A	2.485153481	6.924116162	38.62870576	3.46E-07	3.18E-06
**ENSG00000248124**		-0.903375267	6.02523209	35.79761359	3.47E-07	3.19E-06
**ENSG00000137038**	TMEM261	-0.811072832	4.381728534	33.16501653	3.48E-07	3.19E-06
**ENSG00000163754**	GYG1	0.851677361	4.734554071	33.15693158	3.48E-07	3.2E-06
**ENSG00000125753**	VASP	0.640784251	5.249263493	33.15621896	3.49E-07	3.2E-06
**ENSG00000127084**	FGD3	-0.403695584	7.717272086	33.15026609	3.49E-07	3.2E-06
**ENSG00000115306**	SPTBN1	-0.403599755	8.914553741	33.13728803	3.51E-07	3.21E-06
**ENSG00000101040**	ZMYND8	-0.486275431	6.637313766	33.12480391	3.52E-07	3.22E-06
**ENSG00000251022**	THAP9-AS1	-0.799302365	5.411463904	33.10840398	3.54E-07	3.24E-06
**ENSG00000116473**	RAP1A	0.415106411	7.527243866	33.10433467	3.54E-07	3.24E-06

**ENSG00000051009**	fam160a2	-0.933554864	4.99230133	33.09285258	3.56E-07	3.25E-06
**ENSG00000162909**	CAPN2	0.374640528	8.010321908	33.0452926	3.61E-07	3.29E-06
**ENSG00000112182**	BACH2	-0.648722897	6.856225606	34.61015055	3.61E-07	3.29E-06
**ENSG00000163950**	SLBP	0.660256396	5.334374597	32.98317128	3.69E-07	3.36E-06
**ENSG00000197102**	DYNC1H1	0.305674966	9.124287261	32.9323524	3.75E-07	3.41E-06
**ENSG00000185278**	ZBTB37	-0.798133553	6.858982612	36.31473257	3.75E-07	3.41E-06
**ENSG00000133773**	CCDC59	-0.63859371	5.833217722	32.91051605	3.78E-07	3.43E-06
**ENSG00000055917**	PUM2	0.330427102	8.601826238	32.90380546	3.78E-07	3.43E-06
**ENSG00000114302**	PRKAR2A	0.596678614	6.023177322	32.89288766	3.8E-07	3.44E-06
**ENSG00000156504**	FAM122B	-0.613676216	6.017254609	32.88014699	3.81E-07	3.45E-06
**ENSG00000205758**	CRYZL1	-0.748872179	5.351848617	32.85770667	3.84E-07	3.47E-06
**ENSG00000168824**	NSG1	0.916240178	4.629124162	32.8556969	3.84E-07	3.47E-06
**ENSG00000204525**	HLA-C	0.425341773	9.718267885	32.78138256	3.94E-07	3.56E-06
**ENSG00000142303**	ADAMTS10	-1.342925671	4.072472546	32.99362567	3.95E-07	3.56E-06
**ENSG00000182472**	CAPN12	1.515082568	4.92123923	37.18593265	4.02E-07	3.62E-06
**ENSG00000197747**	S100A10	0.860519412	6.363557912	36.2336006	4.02E-07	3.62E-06
**ENSG00000269713**	NBPF9	-0.697230038	5.116331933	32.70340441	4.04E-07	3.64E-06
**ENSG00000065135**	gnai3	0.447964768	6.924511074	32.68066123	4.07E-07	3.66E-06
**ENSG00000173611**	SCAI	-0.67692207	5.884776749	32.67349096	4.08E-07	3.67E-06
**ENSG00000168675**	LDLRAD4	0.501486932	6.416460456	32.67047037	4.08E-07	3.67E-06
**ENSG00000083312**	TNPO1	0.351544059	7.824153579	32.66270088	4.1E-07	3.67E-06
**ENSG00000166446**	CDYL2	0.8929394	4.500012143	32.65914865	4.1E-07	3.67E-06
**ENSG00000100426**	zbed4	0.540921939	5.801007031	32.64327023	4.12E-07	3.69E-06
**ENSG00000227039**	ITGB2-AS1	-1.630730424	5.217230005	37.44238085	4.15E-07	3.71E-06
**ENSG00000147526**	TACC1	0.40133923	7.419806366	32.61807774	4.16E-07	3.71E-06
**ENSG00000253861**		0.792256471	4.72574997	32.59314413	4.19E-07	3.74E-06
**ENSG00000197043**	ANXA6	0.402299592	7.965860774	32.57483539	4.21E-07	3.76E-06
**ENSG00000138834**	MAPK8IP3	-1.133839918	6.090373864	37.05569894	4.26E-07	3.8E-06
**ENSG00000157873**	TNFRSF14	-0.647388442	5.945183509	32.53655638	4.27E-07	3.8E-06
**ENSG00000102144**	PGK 1.00	0.415940866	7.274556199	32.47631622	4.35E-07	3.87E-06
**ENSG00000140853**	NLRC5	-0.706315367	8.312352496	36.25624485	4.5E-07	4E-06
**ENSG00000272752**	STAG3L5P-PVRI	-1.403685407	6.300931278	37.4778914	4.54E-07	4.03E-06
**ENSG00000165476**	REEP3	0.678934608	5.974256614	32.67564695	4.58E-07	4.07E-06
**ENSG00000091317**	CMTM6	0.586715127	6.432892228	32.31606102	4.59E-07	4.07E-06
**ENSG00000165275**	TRMT10B	-0.795002094	5.240333898	32.2898624	4.63E-07	4.1E-06
**ENSG00000057252**	SOAT1	0.428399183	6.730778355	32.26045969	4.67E-07	4.14E-06
**ENSG00000267009**		0.565339156	5.656711507	32.20770311	4.76E-07	4.21E-06

**ENSG00000134109**	EDEM1	0.330447326	7.699345959	32.19363146	4.78E-07	4.22E-06
**ENSG00000107099**	DOCK8	0.320088316	9.176346336	32.1837008	4.79E-07	4.23E-06
**ENSG00000151743**	AMN1	-1.06155933	4.054025759	32.16359031	4.83E-07	4.26E-06
**ENSG00000069275**	NUCKS1	0.439961007	7.950460825	32.15122164	4.85E-07	4.27E-06
**ENSG00000116983**	HPCAL4	-0.995195787	4.505808918	32.1220818	4.89E-07	4.31E-06
**ENSG00000155090**	KLF10	0.995085325	4.764042413	32.51171744	4.96E-07	4.37E-06
**ENSG00000125375**	ATP5S	-0.64819299	5.786221837	32.05610522	5E-07	4.4E-06
**ENSG00000162368**	CMPK1	0.471298462	7.73485977	32.12188802	5.05E-07	4.44E-06
**ENSG00000152642**	GPD1L	0.594066028	5.285159786	32.02095081	5.06E-07	4.44E-06
**ENSG00000111237**	VPS29	-0.591801065	6.068082842	32.01923269	5.06E-07	4.44E-06
**ENSG00000106479**	ZNF862	-0.744634064	5.489905569	32.00058561	5.09E-07	4.46E-06
**ENSG00000135899**	SP110	-0.426083648	7.315254201	31.99387336	5.1E-07	4.47E-06
**ENSG00000221944**	MIR5001	-0.98211556	4.476158351	31.97414953	5.14E-07	4.49E-06
**ENSG00000114331**	ACAP2	0.317767998	7.950085381	31.97414423	5.14E-07	4.49E-06
**ENSG00000027075**	PRKCH	0.370296718	8.165428136	31.9559578	5.17E-07	4.51E-06
**ENSG00000174946**	GPR171	0.626303616	6.65401262	32.97003169	5.18E-07	4.52E-06
**ENSG00000137817**	PARP6	-0.809779383	5.859699471	33.28392255	5.21E-07	4.55E-06
**ENSG00000165417**	GTF2A1	0.499758017	6.843933702	31.85247198	5.35E-07	4.66E-06
**ENSG00000182287**	AP1S2	0.670343686	5.657500184	31.84883995	5.35E-07	4.66E-06
**ENSG00000109171**	SLAIN2	0.496185554	7.017817432	31.84859923	5.35E-07	4.66E-06
**ENSG00000136770**	DNAJC1	0.859566478	4.517827253	31.81787246	5.41E-07	4.7E-06
**ENSG00000138698**	RAP1GDS1	0.509889518	6.073472391	31.81657443	5.41E-07	4.7E-06
**ENSG00000090238**	YPEL3	-0.797181013	5.462152447	31.72409543	5.58E-07	4.84E-06
**ENSG00000214106**	PAXIP1-AS2	-0.726269348	4.958166817	31.68977761	5.64E-07	4.89E-06
**ENSG00000101544**	ADNP2	0.574540683	5.572593677	31.66080942	5.7E-07	4.94E-06
**ENSG00000141258**	SGSM2	-1.397255398	5.932700731	36.45370757	5.75E-07	4.98E-06
**ENSG00000168488**	ATXN2L	-0.459794601	7.321304219	31.59389626	5.83E-07	5.04E-06
**ENSG00000060749**	QSER1	0.528260372	6.345030177	31.58360147	5.85E-07	5.05E-06
**ENSG00000134709**	HOOK1	-0.74528047	5.376149327	31.55326685	5.91E-07	5.1E-06
**ENSG00000206530**	CFAP44	-1.047014518	5.18749971	33.12209441	5.91E-07	5.1E-06
**ENSG00000159840**	ZYX	0.549038483	6.487114282	31.52904027	5.95E-07	5.13E-06
**ENSG00000274627**		-0.948812004	5.118495074	31.79486067	6.07E-07	5.23E-06
**ENSG00000157978**	LDLRAP1	-0.475442016	7.464583297	31.4350001	6.14E-07	5.29E-06
**ENSG00000040633**	PHF23	0.773953553	4.392195386	31.39685746	6.22E-07	5.35E-06
**ENSG00000263482**		-0.934788127	4.312094643	31.37828969	6.26E-07	5.38E-06
**ENSG00000132300**	PTCD3	-0.539388223	7.291843929	31.97229966	6.3E-07	5.41E-06
**ENSG00000238105**	GOLGA2P5	-0.999792329	5.238870749	32.71086259	6.32E-07	5.42E-06

**ENSG00000164073**	MFSD8	-0.728146487	5.898810746	31.8626582	6.37E-07	5.46E-06
**ENSG00000110958**	PTGES3	0.401859141	7.437602104	31.30666589	6.41E-07	5.49E-06
**ENSG00000211772**		0.398281116	9.021548029	31.30580707	6.41E-07	5.49E-06
**ENSG00000170315**	UBB	0.506217891	7.559867112	31.869261	6.45E-07	5.51E-06
**ENSG00000245205**		1.046129078	3.391126276	31.26441618	6.5E-07	5.55E-06
**ENSG00000055208**	TAB2	0.333098739	8.818595912	31.23364831	6.57E-07	5.61E-06
**ENSG00000258643**	BCL2L2-PABPN1	-0.539640376	5.616103839	31.22949145	6.58E-07	5.61E-06
**ENSG00000066855**	MTFR1	-0.560210898	6.032232876	31.22078086	6.6E-07	5.62E-06
**ENSG00000279641**		0.789227829	5.03727343	31.2146011	6.61E-07	5.63E-06
**ENSG00000104219**	ZDHHC2	0.439777928	6.835012317	31.15908976	6.73E-07	5.73E-06
**ENSG00000161010**	MRNIP	-0.47184551	6.470306169	31.13149163	6.8E-07	5.78E-06
**ENSG00000166839**	ANKDD1A	-1.386530438	3.843094381	31.11403018	6.84E-07	5.81E-06
**ENSG00000131374**	TBC1D5	0.45399348	6.574422673	31.07064728	6.94E-07	5.89E-06
**ENSG00000107643**	MAPK8	-0.473754732	5.836461796	31.03232429	7.03E-07	5.96E-06
**ENSG00000174353**	TRIM74	-1.024637354	4.466238289	31.00280134	7.09E-07	6.01E-06
**ENSG00000181722**	ZBTB20	0.413528397	8.160063181	30.99969787	7.1E-07	6.01E-06
**ENSG00000188997**	KCTD21	1.040067841	3.596769718	30.9897682	7.13E-07	6.03E-06
**ENSG00000185291**	IL3RA	-1.228433362	4.151742702	30.98573641	7.14E-07	6.03E-06
**ENSG00000068400**	GRIPAP1	-0.702313554	5.86167541	31.22713965	7.16E-07	6.05E-06
**ENSG00000184271**	POU6F1	-0.950458054	4.823726536	30.97328171	7.17E-07	6.05E-06
**ENSG00000108963**	DPH1	-0.756886533	5.022539115	30.96529276	7.18E-07	6.06E-06
**ENSG00000104228**	TRIM35	0.667022887	5.416765451	30.96164406	7.19E-07	6.06E-06
**ENSG00000144029**	MRPS5	-0.557301235	5.7837125	30.95540715	7.21E-07	6.07E-06
**ENSG00000136213**	LOC101927181	0.760324731	5.398541687	30.92330213	7.29E-07	6.13E-06
**ENSG00000115446**	UNC50	-0.761295374	4.973283949	30.91826811	7.3E-07	6.14E-06
**ENSG00000160888**	IER2	0.891342889	6.140443532	34.05592153	7.34E-07	6.16E-06
**ENSG00000181754**	AMIGO1	-0.691291763	5.511341057	30.90101695	7.34E-07	6.16E-06
**ENSG00000115368**	WDR75	-0.491687685	6.521022564	30.87619913	7.4E-07	6.21E-06
**ENSG00000162894**	FCMR	-0.450447324	7.80997734	30.85655592	7.45E-07	6.24E-06
**ENSG00000204388**	hspa1b	1.057158017	3.939178687	30.85406554	7.46E-07	6.24E-06
**ENSG00000198830**	HMGN2	-0.408652049	6.684502219	30.84986246	7.47E-07	6.25E-06
**ENSG00000149084**	HSD17B12	0.684961498	5.058767199	30.84071679	7.49E-07	6.26E-06
**ENSG00000123104**	ITPR2	0.446747707	7.345155802	30.83937316	7.49E-07	6.26E-06
**ENSG00000175274**	TP53I11	0.856191318	3.983428006	30.81365522	7.56E-07	6.31E-06
**ENSG00000225205**		-0.789463249	4.887469286	30.80766249	7.57E-07	6.32E-06
**ENSG00000104613**	INTS10	-0.480358944	6.235346456	30.78959269	7.62E-07	6.35E-06
**ENSG00000163636**	PSMD6	-0.553827788	7.066282425	31.35352679	7.66E-07	6.38E-06

**ENSG00000204514**	ZNF814	-0.518293569	6.014911186	30.74173475	7.74E-07	6.44E-06
**ENSG00000213402**	PTPRCAP	0.68791154	6.015948487	31.20847674	7.8E-07	6.48E-06
**ENSG00000101608**	MYL12A	0.576340382	8.277993772	32.94805514	7.8E-07	6.48E-06
**ENSG00000160584**	SIK3	0.379421938	6.860242771	30.64013113	8.01E-07	6.65E-06
**ENSG00000134900**	TPP2	-0.409776765	7.802592564	30.63618251	8.02E-07	6.65E-06
**ENSG00000188042**	ARL4C	0.37711747	8.395423396	30.58504899	8.16E-07	6.76E-06
**ENSG00000123612**	ACVR1C	-0.99997393	4.401129124	30.56638377	8.21E-07	6.8E-06
**ENSG00000197746**	PSAP	0.454043842	7.045299072	30.56161609	8.23E-07	6.81E-06
**ENSG00000169062**	UPF3A	-0.668421401	5.764488585	30.49897746	8.4E-07	6.94E-06
**ENSG00000267598**		0.891408008	5.471442898	31.7166182	8.54E-07	7.05E-06
**ENSG00000085365**	SCAMP1	0.61316678	5.453535314	30.42495929	8.61E-07	7.11E-06
**ENSG00000148700**	ADD3	-0.303954701	9.083341277	30.42394121	8.62E-07	7.11E-06
**ENSG00000198961**	PJA2	0.394188915	7.712138036	30.40334556	8.68E-07	7.15E-06
**ENSG00000113851**	CRBN	-0.414673576	7.723260329	30.37229332	8.77E-07	7.22E-06
**ENSG00000120458**	MSANTD2	-0.972548015	4.443919184	30.35979922	8.8E-07	7.24E-06
**ENSG00000151414**	NEK7	0.461217288	6.991513233	30.3330868	8.88E-07	7.3E-06
**ENSG00000128951**	DUT	-0.691290986	5.43472372	30.30523546	8.97E-07	7.37E-06
**ENSG00000055070**	SZRD1	0.493352	5.872667823	30.30113705	8.98E-07	7.37E-06
**ENSG00000113569**	NUP155	0.440818653	6.424066124	30.25656249	9.12E-07	7.48E-06
**ENSG00000102753**	KPNA3	0.520311213	6.153097121	30.25266786	9.13E-07	7.48E-06
**ENSG00000166289**	PLEKHF1	1.091655077	3.644195541	30.21605586	9.24E-07	7.57E-06
**ENSG00000145391**	SETD7	0.74221226	5.033679601	30.21101889	9.26E-07	7.57E-06
**ENSG00000023902**	PLEKHO1	0.597604819	5.591045258	30.20097868	9.29E-07	7.59E-06
**ENSG00000228784**	LINC00954	-1.135965773	4.693815989	31.00782103	9.6E-07	7.84E-06
**ENSG00000111897**	SERINC1	0.406129749	8.179289485	30.09884811	9.62E-07	7.85E-06
**ENSG00000147155**	EBP	1.08886965	3.692695825	30.08821991	9.65E-07	7.87E-06
**ENSG00000102580**	DNAJC3	0.514042248	6.263641041	30.05716077	9.75E-07	7.95E-06
**ENSG00000144134**	RABL2A	-1.366235305	3.851248731	30.16806052	9.77E-07	7.96E-06
**ENSG00000057608**	GDI2	0.395968559	7.588334963	30.04790956	9.78E-07	7.96E-06
**ENSG00000180694**	TMEM64	0.807632373	4.739137484	30.02046608	9.87E-07	8.03E-06
**ENSG00000187953**	PMS2CL	-0.992367472	3.83598807	29.98497003	9.99E-07	8.12E-06
**ENSG00000135404**	CD63	1.233941958	3.97461553	30.31397226	1E-06	8.14E-06
**ENSG00000204569**	PPP1R10	0.470103379	6.602000156	29.97316945	1E-06	8.14E-06
**ENSG00000258813**		-1.101468049	4.026275597	29.96187214	1.01E-06	8.16E-06
**ENSG00000133226**	SRRM1	-0.326480899	8.055164378	29.95607035	1.01E-06	8.17E-06
**ENSG00000152492**	CCDC50	0.940368122	4.949806984	30.33665599	1.02E-06	8.24E-06
**ENSG00000269609**	RPARP-AS1	-0.919676107	4.42532427	29.90528707	1.03E-06	8.3E-06

**ENSG00000187446**	CHP1	0.729322671	5.073226725	29.89801823	1.03E-06	8.32E-06
**ENSG00000114770**	ABCC5	-0.809108323	5.362702782	29.89992345	1.04E-06	8.41E-06
**ENSG00000167995**	BEST1	0.524735236	8.244210048	31.2444645	1.06E-06	8.52E-06
**ENSG00000204131**	NHSL2	0.881391755	6.408918558	33.35201643	1.06E-06	8.52E-06
**ENSG00000147274**	RBMX	-0.365995795	8.106355118	29.80772777	1.06E-06	8.55E-06
**ENSG00000168066**	SF1	-0.407812434	8.561245687	29.74093661	1.09E-06	8.74E-06
**ENSG00000006831**	ADIPOR2	0.593611901	5.388000444	29.71799086	1.09E-06	8.8E-06
**ENSG00000175826**	CTDNEP1	0.537021666	6.224315564	29.69108969	1.1E-06	8.87E-06
**ENSG00000197283**	MIR5004	-0.592662348	6.345060288	29.73740154	1.11E-06	8.9E-06
**ENSG00000241489**		0.412081564	6.503432633	29.65685633	1.12E-06	8.96E-06
**ENSG00000070961**	ATP2B1	0.414092126	7.64448218	29.6480665	1.12E-06	8.98E-06
**ENSG00000168970**	jmjd7-pla2g4b	-1.215550999	5.763173531	33.55232544	1.12E-06	8.98E-06
**ENSG00000204681**	GABBR1	-1.493193385	6.035545174	33.96878407	1.13E-06	9.05E-06
**ENSG00000155287**	SLC25A28	-0.925158015	4.632611451	29.6191696	1.13E-06	9.05E-06
**ENSG00000179406**	LINC00174	-0.936963939	4.495793471	29.60471005	1.14E-06	9.09E-06
**ENSG00000225828**	FAM229A	-1.153238688	4.179721956	29.58944145	1.14E-06	9.13E-06
**ENSG00000228315**	GUSBP11	-1.193963328	5.85102035	33.52040101	1.15E-06	9.15E-06
**ENSG00000196187**	TMEM63A	-0.659135244	8.393429892	32.73826295	1.16E-06	9.23E-06
**ENSG00000184956**	MUC6	-1.400147254	4.036783132	30.15050616	1.16E-06	9.25E-06
**ENSG00000261490**		-0.772849924	5.418988676	29.5115852	1.17E-06	9.35E-06
**ENSG00000165175**	MID1IP1	0.808548906	4.667219227	29.50528577	1.18E-06	9.36E-06
**ENSG00000149428**	HYOU1	0.579529448	5.766904531	29.47408023	1.19E-06	9.45E-06
**ENSG00000101391**	CDK5RAP1	-0.777140155	5.354005954	29.46305381	1.19E-06	9.48E-06
**ENSG00000156171**	DRAM2	-0.545055733	5.569947169	29.46133091	1.19E-06	9.48E-06
**ENSG00000173852**	DPY19L1	0.831195241	4.715429016	29.38579597	1.23E-06	9.72E-06
**ENSG00000213585**	VDAC1	0.595094763	5.586068007	29.37847009	1.23E-06	9.74E-06
**ENSG00000205784**	ARRDC5	-1.107867446	3.593870853	29.35703256	1.24E-06	9.8E-06
**ENSG00000211797**		1.056609156	3.525913224	29.34967205	1.24E-06	9.82E-06
**ENSG00000163412**	EIF4E3	0.397946307	7.487311861	29.32481529	1.25E-06	9.89E-06
**ENSG00000235016**	SEMA3F-AS1	-0.814987862	5.105559405	29.29933909	1.26E-06	9.97E-06
**ENSG00000167196**	FBXO22	-0.798398084	5.511397018	29.80464757	1.27E-06	1E-05
**ENSG00000154832**	CXXC1	-0.69999482	5.405352025	29.27523924	1.27E-06	1E-05
**ENSG00000187741**	FANCA	-0.531535698	6.2206526	29.26956771	1.27E-06	1.01E-05
**ENSG00000149292**	TTC12	-0.802350069	5.229461602	29.23816217	1.29E-06	1.02E-05
**ENSG00000092529**	CAPN3	-1.258152526	4.090507951	29.26270719	1.29E-06	1.02E-05
**ENSG00000112983**	BRD8	-0.565325915	5.691129845	29.21222045	1.3E-06	1.02E-05
**ENSG00000141524**	TMC6	-0.74226397	7.461094425	32.59556064	1.3E-06	1.02E-05

**ENSG00000214941**	ZSWIM7	-1.235369766	4.006516298	29.19058246	1.31E-06	1.03E-05
**ENSG00000097033**	SH3GLB1	0.395389868	6.957505038	29.15702317	1.32E-06	1.04E-05
**ENSG00000175548**	ALG10B	-0.788227876	5.021783551	29.14206883	1.33E-06	1.04E-05
**ENSG00000162222**	TTC9C	-0.839197739	4.509873899	29.14182898	1.33E-06	1.04E-05
**ENSG00000281691**	RBM5-AS1	-0.66915421	5.639414179	29.12351428	1.34E-06	1.05E-05
**ENSG00000128340**	RAC2	0.504923726	8.425429989	30.25565084	1.37E-06	1.07E-05
**ENSG00000258407**		1.002325658	4.309694388	29.0550701	1.37E-06	1.07E-05
**ENSG00000078304**	PPP2R5C	0.2869817	8.729647943	29.03811202	1.38E-06	1.08E-05
**ENSG00000167377**	ZNF23	-0.99847879	4.140383654	29.02356675	1.39E-06	1.08E-05
**ENSG00000136141**	LRCH1	0.545515215	5.380344455	29.01535416	1.39E-06	1.09E-05
**ENSG00000110324**	IL10RA	0.44454657	8.419764943	29.206005	1.4E-06	1.09E-05
**ENSG00000107581**	EIF3A	0.379338083	8.861793408	28.98291553	1.41E-06	1.1E-05
**ENSG00000170759**	KIF5B	0.353444229	7.838990264	28.94673939	1.42E-06	1.11E-05
**ENSG00000163626**	cox18	-0.848780378	4.719914275	28.94031337	1.43E-06	1.11E-05
**ENSG00000171824**	EXOSC10	-0.467680427	6.570592726	28.93990698	1.43E-06	1.11E-05
**ENSG00000115687**	PASK	-0.515588069	6.20714751	28.89885265	1.45E-06	1.12E-05
**ENSG00000083168**	KAT6A	0.348750542	8.583509448	28.89884604	1.45E-06	1.12E-05
**ENSG00000134444**	KIAA1468	-0.506756551	6.959683279	28.8748396	1.46E-06	1.13E-05
**ENSG00000176624**	MEX3C	0.453480716	6.626033883	28.86689746	1.46E-06	1.13E-05
**ENSG00000081791**	KIAA0141	-0.672080846	5.743880899	28.85791184	1.47E-06	1.14E-05
**ENSG00000143761**	MIR3620	0.501232406	6.934912175	28.85049472	1.47E-06	1.14E-05
**ENSG00000150593**	MIR4680	0.360433016	8.375385433	28.82163697	1.49E-06	1.15E-05
**ENSG00000169756**	LIMS1	0.501331558	7.342678526	29.22961901	1.52E-06	1.17E-05
**ENSG00000172349**	IL16	-0.306852141	8.43368782	28.75349265	1.52E-06	1.17E-05
**ENSG00000114166**	KAT2B	0.449543491	7.56777825	28.75327943	1.52E-06	1.17E-05
**ENSG00000113742**	CPEB4	0.506041004	5.826658645	28.74160053	1.53E-06	1.18E-05
**ENSG00000258465**		-0.612143481	6.070703645	28.71984359	1.54E-06	1.19E-05
**ENSG00000211817**		1.284334688	3.482612264	28.71482172	1.54E-06	1.19E-05
**ENSG00000137497**	NUMA1	-0.347349335	8.540539492	28.70865229	1.54E-06	1.19E-05
**ENSG00000142173**	COL6A2	-1.218911635	3.84890024	28.69325021	1.55E-06	1.19E-05
**ENSG00000267680**	ZNF224	-0.568192507	6.132882659	28.6923035	1.55E-06	1.19E-05
**ENSG00000175309**	PHYKPL	-0.666503675	6.701073914	30.2545261	1.56E-06	1.2E-05
**ENSG00000122678**	MIR6838	-0.871336089	4.753531487	28.66659335	1.57E-06	1.2E-05
**ENSG00000117280**	RAB29	0.481873789	6.160224767	28.65908255	1.57E-06	1.2E-05
**ENSG00000281005**	LINC00921	-0.970034205	4.427297069	28.64228614	1.58E-06	1.21E-05
**ENSG00000139990**	DCAF5	-0.371644536	6.984942223	28.623985	1.59E-06	1.22E-05
**ENSG00000178974**	fbxo34	0.61203069	5.585085488	28.58175501	1.61E-06	1.23E-05

**ENSG00000118705**	RPN2	0.416880759	6.876730611	28.52625465	1.65E-06	1.26E-05
**ENSG00000061273**	HDAC7	-0.632512863	6.500353582	29.37900301	1.65E-06	1.26E-05
**ENSG00000051108**	HERPUD1	0.511290177	6.007900591	28.4952678	1.66E-06	1.27E-05
**ENSG00000169718**	DUS1L	-0.732570389	5.275691513	28.49032987	1.67E-06	1.27E-05
**ENSG00000180198**	RCC1	-0.911854169	4.759795126	28.48292537	1.67E-06	1.27E-05
**ENSG00000149532**	CPSF7	-0.45985872	6.433145844	28.48272543	1.67E-06	1.27E-05
**ENSG00000086758**	HUWE1	0.322549199	9.017991614	28.47094773	1.68E-06	1.28E-05
**ENSG00000259205**		-1.189039705	4.098998718	28.45775343	1.68E-06	1.28E-05
**ENSG00000183337**	BCOR	0.38658777	6.623961126	28.43233574	1.7E-06	1.29E-05
**ENSG00000174718**	KIAA1551	0.299197799	10.15760417	28.41690845	1.71E-06	1.3E-05
**ENSG00000137266**	SLC22A23	-0.802142014	4.995807167	28.41648384	1.71E-06	1.3E-05
**ENSG00000026103**	FAS	0.581187185	6.027705728	28.39853935	1.72E-06	1.3E-05
**ENSG00000130813**	c19orf66	-0.702969215	5.345152872	28.39472225	1.72E-06	1.3E-05
**ENSG00000237499**	LOC100130476	-0.953371915	4.668756394	28.37735181	1.73E-06	1.31E-05
**ENSG00000107672**	NSMCE4A	-0.865600779	5.412752861	29.25641608	1.73E-06	1.31E-05
**ENSG00000055332**	eif2ak2	0.462842529	7.316595028	28.36076769	1.74E-06	1.32E-05
**ENSG00000140474**	ULK3	-0.974467851	4.983314817	29.17561398	1.75E-06	1.32E-05
**ENSG00000112208**	BAG2	-0.858734906	4.316168423	28.34618078	1.75E-06	1.32E-05
**ENSG00000168014**	C2CD3	-0.509959512	6.100079945	28.33984236	1.75E-06	1.32E-05
**ENSG00000183943**	PRKX	0.390079832	7.152923249	28.3366707	1.76E-06	1.32E-05
**ENSG00000120896**	SORBS3	-0.933049053	4.417353755	28.33523818	1.76E-06	1.32E-05
**ENSG00000103479**	RBL2	-0.347060403	9.261185301	28.30435494	1.78E-06	1.34E-05
**ENSG00000054523**	KIF1B	0.625991096	5.760269898	28.29661261	1.78E-06	1.34E-05
**ENSG00000214135**	LOC220729	-0.971901038	5.288687996	29.79576802	1.8E-06	1.35E-05
**ENSG00000197971**	MBP	0.385334103	7.392609152	28.25471927	1.81E-06	1.36E-05
**ENSG00000178252**	wdr6	-0.558883303	6.388082481	28.24168293	1.82E-06	1.36E-05
**ENSG00000172270**	BSG	0.629304727	5.226701537	28.23668005	1.82E-06	1.36E-05
**ENSG00000138640**	FAM13A	-0.679970835	6.461380522	29.65908226	1.83E-06	1.37E-05
**ENSG00000182378**	PLCXD1	-0.922254703	4.658047334	28.20321181	1.84E-06	1.38E-05
**ENSG00000154114**	TBCEL	-0.829135908	4.714884842	28.19633821	1.84E-06	1.38E-05
**ENSG00000179456**	zbtb18	-0.665665391	6.172993179	28.97418959	1.85E-06	1.38E-05
**ENSG00000116095**	PLEKHA3	0.426386231	6.326036581	28.19155785	1.85E-06	1.38E-05
**ENSG00000273000**		-1.127697223	5.245574443	30.56710699	1.86E-06	1.39E-05
**ENSG00000259865**		-0.902593892	4.657102119	28.15941255	1.87E-06	1.39E-05
**ENSG00000197448**	GSTK1	-0.423585394	7.902321742	28.14869575	1.87E-06	1.4E-05
**ENSG00000156475**	PPP2R2B	1.035320881	4.192842421	28.14145203	1.88E-06	1.4E-05
**ENSG00000110665**	C11orf21	-0.734578868	5.334989712	28.13842045	1.88E-06	1.4E-05

**ENSG00000134242**	PTPN22	0.396899707	6.611479556	28.13817746	1.88E-06	1.4E-05
**ENSG00000266412**	NCOA4	0.353982969	7.293645039	28.12189497	1.89E-06	1.41E-05
**ENSG00000161547**	SRSF2	-0.673699535	7.472654856	30.89666204	1.89E-06	1.41E-05
**ENSG00000176871**	WSB2	0.632774117	5.201979992	28.09728029	1.91E-06	1.42E-05
**ENSG00000162676**	GFI1	1.001617819	4.106819293	28.08193809	1.92E-06	1.42E-05
**ENSG00000136243**	NUPL2	-0.67401508	4.871760245	28.0596536	1.93E-06	1.43E-05
**ENSG00000245958**	LOC645513	-0.826822946	5.27402423	28.33393864	1.96E-06	1.45E-05
**ENSG00000126247**	CAPNS1	0.636234289	6.209222664	28.71051852	1.96E-06	1.45E-05
**ENSG00000129071**	MBD4	-0.526111506	5.949771811	27.97814407	1.99E-06	1.47E-05
**ENSG00000106123**	EPHB6	-1.040252601	4.333934014	27.97427393	1.99E-06	1.47E-05
**ENSG00000163848**	ZNF148	0.419973891	7.306226363	27.94314547	2.01E-06	1.49E-05
**ENSG00000118579**	MED28	-0.377501094	7.232982043	27.91710945	2.03E-06	1.5E-05
**ENSG00000205903**	ZNF316	-1.040116051	4.440286065	27.88977448	2.05E-06	1.51E-05
**ENSG00000144026**	ZNF514	-0.895721417	4.948628783	28.05380978	2.07E-06	1.53E-05
**ENSG00000171115**	GIMAP8	-0.472701587	6.221781616	27.86107812	2.07E-06	1.53E-05
**ENSG00000174136**	RGMB	-0.919013285	4.613913253	27.85411612	2.08E-06	1.53E-05
**ENSG00000148399**	DPH7	-1.186067456	3.993457179	27.83063314	2.09E-06	1.54E-05
**ENSG00000197774**	EME2	-0.916043558	5.421326873	29.22387571	2.11E-06	1.55E-05
**ENSG00000204632**	HLA-G	0.827213753	4.852317488	27.80405808	2.11E-06	1.55E-05
**ENSG00000104960**	PTOV1	-0.875548001	5.360263429	28.65308367	2.13E-06	1.56E-05
**ENSG00000128923**	FAM63B	0.495488217	5.987105997	27.73945955	2.16E-06	1.59E-05
**ENSG00000109046**	WSB1	-0.478645461	7.060933775	27.72491441	2.17E-06	1.59E-05
**ENSG00000272916**	NDST2	-0.516348193	6.40213927	27.70343782	2.19E-06	1.6E-05
**ENSG00000120253**	NUP43	-0.546452051	5.973441531	27.68921179	2.2E-06	1.61E-05
**ENSG00000258297**		-0.768626587	5.177294463	27.68461208	2.2E-06	1.61E-05
**ENSG00000117899**	MESDC2	0.494506987	5.672604233	27.68152181	2.2E-06	1.61E-05
**ENSG00000128694**	OSGEPL1	-0.840298286	4.500302591	27.66590291	2.22E-06	1.62E-05
**ENSG00000095139**	ARCN1	0.377508685	7.249537231	27.65455166	2.23E-06	1.63E-05
**ENSG00000278791**	MIR6723	1.236359184	6.543175428	31.47726272	2.23E-06	1.63E-05
**ENSG00000279933**		-0.930593529	5.117540715	28.33862301	2.24E-06	1.63E-05
**ENSG00000111912**	NCOA7	0.412783711	6.67760403	27.62092793	2.25E-06	1.64E-05
**ENSG00000283189**		-0.969726602	4.723597472	27.75800456	2.25E-06	1.64E-05
**ENSG00000109971**	HSPA8	0.4189402	9.957015422	27.60446784	2.26E-06	1.65E-05
**ENSG00000196605**	ZNF846	-1.207522181	3.816189706	27.58839025	2.28E-06	1.66E-05
**ENSG00000080189**	SLC35C2	-0.679587955	5.697347996	27.58635794	2.28E-06	1.66E-05
**ENSG00000107611**	CUBN	-0.873623872	5.360698263	28.47494418	2.32E-06	1.69E-05
**ENSG00000176953**	MIR4517	-0.674144706	6.325219398	28.69355479	2.34E-06	1.7E-05

**ENSG00000102699**	PARP4	0.36793246	7.758745764	27.48750501	2.36E-06	1.71E-05
**ENSG00000102096**	PIM2	-0.515280556	7.195587709	28.04355842	2.36E-06	1.71E-05
**ENSG00000139636**	LMBR1L	-0.817299463	5.226958162	27.55527373	2.36E-06	1.71E-05
**ENSG00000215788**	TNFRSF25	-1.046662857	6.518308659	31.10697517	2.37E-06	1.71E-05
**ENSG00000206341**		0.635160935	6.454577555	28.53695551	2.38E-06	1.72E-05
**ENSG00000114738**	MAPKAPK3	0.682313421	5.373001051	27.45984209	2.38E-06	1.72E-05
**ENSG00000238018**		-0.727802236	4.703193226	27.45678387	2.38E-06	1.72E-05
**ENSG00000159496**	RGL4	-0.813840782	4.74008453	27.44087096	2.4E-06	1.73E-05
**ENSG00000111780**		0.858504669	4.445042467	27.42770063	2.41E-06	1.74E-05
**ENSG00000165434**	PGM2L1	0.651966398	5.588103388	27.35923005	2.47E-06	1.78E-05
**ENSG00000128284**	apol3	-0.415271532	6.398631752	27.34621308	2.48E-06	1.79E-05
**ENSG00000257093**	KIAA1147	-0.405993099	7.1928962	27.31797874	2.5E-06	1.8E-05
**ENSG00000176248**	ANAPC2	-0.927352987	4.846817887	27.47306403	2.51E-06	1.8E-05
**ENSG00000174227**	PIGG	-0.655063487	5.790739019	27.28948652	2.53E-06	1.82E-05
**ENSG00000118496**	FBXO30	0.701418907	5.34754884	27.28503695	2.53E-06	1.82E-05
**ENSG00000121716**	MIR6840	-1.271321251	5.651781844	30.72533635	2.56E-06	1.84E-05
**ENSG00000103653**	CSK	0.429477693	6.539665528	27.23245343	2.58E-06	1.85E-05
**ENSG00000163617**	CCDC191	-1.132088451	3.906520613	27.21241383	2.6E-06	1.86E-05
**ENSG00000143819**	EPHX1	-0.975146217	4.572013301	27.20755898	2.6E-06	1.86E-05
**ENSG00000235333**		-1.282022034	3.8614374	27.29793763	2.61E-06	1.87E-05
**ENSG00000276045**	ORAI1	0.658453644	5.360242106	27.18811637	2.62E-06	1.87E-05
**ENSG00000163376**	KBTBD8	0.910279101	3.941494396	27.17955598	2.63E-06	1.88E-05
**ENSG00000072786**	STK10	0.295062203	8.504141371	27.17737279	2.63E-06	1.88E-05
**ENSG00000068796**	KIF2A	0.346150813	7.966477606	27.16410862	2.64E-06	1.89E-05
**ENSG00000265401**		0.537794907	7.222547561	28.1937711	2.65E-06	1.89E-05
**ENSG00000135677**	GNS	0.562354364	5.915993543	27.14763617	2.66E-06	1.89E-05
**ENSG00000281344**		-2.038591607	4.477601899	30.52756152	2.67E-06	1.9E-05
**ENSG00000167996**	FTH1	0.496657867	8.384925082	28.30119291	2.68E-06	1.9E-05
**ENSG00000183726**	TMEM50A	0.589598565	6.126095037	27.30990288	2.68E-06	1.9E-05
**ENSG00000145390**	USP53	-0.444907301	6.925677825	27.12692368	2.68E-06	1.9E-05
**ENSG00000213614**	HEXA	-0.602716426	5.58825739	27.02216866	2.78E-06	1.97E-05
**ENSG00000280135**		-0.70906874	4.944086331	26.99754935	2.8E-06	1.99E-05
**ENSG00000132388**	UBE2G1	0.477582175	6.156227044	26.97666387	2.82E-06	2E-05
**ENSG00000116299**	KIAA1324	0.602870364	4.955492411	26.97396246	2.82E-06	2E-05
**ENSG00000154025**	SLC5A10	-0.653854469	4.880908501	26.91245127	2.89E-06	2.04E-05
**ENSG00000281376**	ABALON	0.981237318	3.669002949	26.91230486	2.89E-06	2.04E-05
**ENSG00000176715**	ACSF3	-0.691789545	5.780766505	27.1953646	2.89E-06	2.04E-05

**ENSG00000095951**	hivep1	-0.435319958	6.751310438	26.8852499	2.91E-06	2.06E-05
**ENSG00000142396**	LOC105372481	-0.683528993	5.740045931	27.01809482	2.97E-06	2.1E-05
**ENSG00000163219**	ARHGAP25	0.368602748	7.241451604	26.83053889	2.97E-06	2.1E-05
**ENSG00000137841**	PLCB2	-0.754212689	6.889457657	29.53977811	2.99E-06	2.11E-05
**ENSG00000079974**	RABL2B	-0.829837178	4.744509228	26.81364673	2.99E-06	2.11E-05
**ENSG00000117676**	RPS6KA1	0.508366687	5.97248923	26.7837888	3.02E-06	2.13E-05
**ENSG00000122477**	LRRC39	-1.150226975	4.055213532	26.77642795	3.03E-06	2.13E-05
**ENSG00000137200**	CMTR1	-0.625489127	5.67135952	26.7391107	3.07E-06	2.16E-05
**ENSG00000085063**	CD59	0.677369317	5.120934794	26.72432218	3.08E-06	2.17E-05
**ENSG00000108669**	CYTH1	-0.308820137	8.374377303	26.71708596	3.09E-06	2.17E-05
**ENSG00000083642**	PDS5B	0.380565613	7.242061452	26.71003418	3.1E-06	2.18E-05
**ENSG00000182979**	MTA1	-0.692779698	5.496896856	26.70736712	3.1E-06	2.18E-05
**ENSG00000058600**	POLR3E	-0.539779933	6.07864618	26.70520753	3.11E-06	2.18E-05
**ENSG00000111252**	SH2B3	0.455322073	5.970167238	26.69586559	3.12E-06	2.18E-05
**ENSG00000134970**	TMED7	0.583946382	6.30603665	27.08436699	3.12E-06	2.19E-05
**ENSG00000105865**	DUS4L	-1.196716467	3.738096893	26.64327193	3.17E-06	2.22E-05
**ENSG00000106560**	GIMAP2	-0.450540653	6.944079159	26.63267197	3.19E-06	2.23E-05
**ENSG00000167302**	TEPSIN	-1.137409409	4.091172348	26.61447486	3.21E-06	2.24E-05
**ENSG00000118007**	STAG1	0.413562832	6.950979734	26.58826362	3.24E-06	2.26E-05
**ENSG00000136444**	RSAD1	-0.746403268	4.594984792	26.5753536	3.25E-06	2.27E-05
**ENSG00000004897**	CDC27	0.374963367	6.614759186	26.56077906	3.27E-06	2.28E-05
**ENSG00000104343**	UBE2W	0.549419026	5.780596172	26.54906449	3.28E-06	2.29E-05
**ENSG00000128590**	DNAJB9	0.677202669	5.501641393	26.5200246	3.32E-06	2.31E-05
**ENSG00000144749**	LRIG1	0.443169316	6.42787115	26.47252203	3.37E-06	2.35E-05
**ENSG00000142197**	DOPEY2	0.558476622	5.646583383	26.44678494	3.4E-06	2.37E-05
**ENSG00000267697**	LUZP6	0.745598613	4.304758425	26.43137943	3.42E-06	2.38E-05
**ENSG00000085998**	POMGNT1	-0.905723554	4.229175688	26.40649572	3.45E-06	2.4E-05
**ENSG00000140367**	UBE2Q2	0.51238854	6.385135326	26.3432673	3.53E-06	2.45E-05
**ENSG00000186019**	LOC100379224	-0.679949436	5.088478858	26.3012254	3.58E-06	2.49E-05
**ENSG00000110013**	SIAE	-0.720229408	4.939773174	26.28915756	3.6E-06	2.5E-05
**ENSG00000162086**	ZNF75A	-0.527267348	5.721140057	26.2826338	3.61E-06	2.5E-05
**ENSG00000251369**	ZNF550	-0.932811598	5.178837737	27.41399697	3.65E-06	2.53E-05
**ENSG00000167797**	CDK2AP2	0.926456416	4.806700212	26.80154945	3.66E-06	2.53E-05
**ENSG00000009724**	MASP2	-1.086534929	4.507162778	26.7797257	3.66E-06	2.53E-05
**ENSG00000146826**	MIR4658	-0.95619564	4.639026173	26.24520477	3.71E-06	2.57E-05
**ENSG00000198040**	ZNF84	-0.600164555	5.910417182	26.18081781	3.74E-06	2.58E-05
**ENSG00000166794**	PPIB	0.524639257	6.412296944	26.15831168	3.77E-06	2.6E-05

**ENSG00000105221**	AKT2	-0.46685315	6.123011037	26.11793353	3.82E-06	2.64E-05
**ENSG00000231389**	HLA-DPA1	1.004768744	4.909509106	27.27359896	3.83E-06	2.64E-05
**ENSG00000182628**	SKA2	0.606646894	4.878875513	26.11156119	3.83E-06	2.64E-05
**ENSG00000237973**		0.967393092	8.031687411	29.53964408	3.83E-06	2.64E-05
**ENSG00000184465**	WDR27	-1.115438566	5.174300876	28.18867016	3.86E-06	2.65E-05
**ENSG00000165280**	VCP	0.40003081	6.934066532	26.08857767	3.86E-06	2.66E-05
**ENSG00000104093**	DMXL2	-1.371590611	4.054261563	26.86242059	3.87E-06	2.66E-05
**ENSG00000111641**	NOP2	-0.65367769	5.128252092	26.07101194	3.89E-06	2.67E-05
**ENSG00000176438**	syne3	0.380434791	7.320126821	26.06525284	3.9E-06	2.67E-05
**ENSG00000169764**	UGP2	0.376646266	6.9381529	26.06397289	3.9E-06	2.67E-05
**ENSG00000197879**	MYO1C	0.864176036	4.486264697	26.05816513	3.91E-06	2.68E-05
**ENSG00000213983**	ap1g2	-0.922450141	7.03695576	29.35362456	3.91E-06	2.68E-05
**ENSG00000198793**	MTOR	-0.430437494	6.811475728	26.04998929	3.92E-06	2.68E-05
**ENSG00000225648**	SBDSP1	-0.620085424	5.284924183	26.04694891	3.92E-06	2.68E-05
**ENSG00000185477**	GPRIN3	0.398999584	8.688959591	26.04189496	3.93E-06	2.69E-05
**ENSG00000198408**	MGEA5	-0.407204989	8.88147995	26.03807037	3.93E-06	2.69E-05
**ENSG00000166454**	ATMIN	0.463093916	6.595821668	26.01762349	3.96E-06	2.71E-05
**ENSG00000127419**	tmem175	-0.992852447	4.270524702	25.9976718	3.99E-06	2.72E-05
**ENSG00000163902**	RPN1	0.497083759	6.359193039	25.96706573	4.04E-06	2.75E-05
**ENSG00000136810**	TXN	1.012724546	4.498642127	26.47828941	4.05E-06	2.76E-05
**ENSG00000181523**	SGSH	-0.706391379	5.577678792	26.04777398	4.07E-06	2.77E-05
**ENSG00000178199**	ZC3H12D	-0.541763482	6.087138159	25.93994301	4.07E-06	2.77E-05
**ENSG00000122417**	ODF2L	-0.642286407	7.086474021	27.89424135	4.12E-06	2.8E-05
**ENSG00000115085**	ZAP70	-0.707239792	7.16471008	28.45765039	4.19E-06	2.85E-05
**ENSG00000134318**	ROCK2	0.442955159	6.800415932	25.84858604	4.21E-06	2.86E-05
**ENSG00000140157**	NIPA2	0.539509806	5.362655966	25.84845703	4.21E-06	2.86E-05
**ENSG00000239306**	RBM14	-0.452328134	6.468004008	25.84478002	4.22E-06	2.86E-05
**ENSG00000148187**	MRRF	-0.675499579	5.259403239	25.83584194	4.23E-06	2.87E-05
**ENSG00000065029**	ZNF76	-0.999902281	5.203964946	27.38007676	4.27E-06	2.89E-05
**ENSG00000135535**	CD164	0.327147944	8.364294484	25.79931768	4.28E-06	2.9E-05
**ENSG00000280077**		-0.794735275	4.235152491	25.76988847	4.33E-06	2.93E-05
**ENSG00000090104**	RGS1	1.358253382	5.354542275	28.91237465	4.33E-06	2.93E-05
**ENSG00000130479**	MAP1S	0.808032799	4.089452263	25.76328913	4.34E-06	2.93E-05
**ENSG00000157734**	SNX22	0.544243094	5.98795199	25.7489769	4.36E-06	2.94E-05
**ENSG00000140988**	RPS2	0.559271479	9.088026376	27.76272476	4.39E-06	2.96E-05
**ENSG00000187650**	VMAC	-1.099643788	4.165247591	25.71026068	4.42E-06	2.98E-05
**ENSG00000143549**	TPM3	0.357536523	8.400349469	25.6879418	4.46E-06	3E-05

**ENSG00000257524**		-1.141563615	4.131991311	25.7036173	4.47E-06	3.01E-05
**ENSG00000157500**	APPL1	0.422458901	6.702647743	25.67802856	4.47E-06	3.01E-05
**ENSG00000134333**	LDHA	0.443556733	7.10320659	25.65490957	4.51E-06	3.03E-05
**ENSG00000265118**		0.399649244	8.170014629	25.63894966	4.54E-06	3.05E-05
**ENSG00000163655**	GMPS	0.447094537	6.557070893	25.62147413	4.57E-06	3.07E-05
**ENSG00000136108**	CKAP2	0.611945303	5.317080469	25.61964722	4.57E-06	3.07E-05
**ENSG00000169991**	IFFO2	0.542429694	6.009094716	25.61206454	4.58E-06	3.07E-05
**ENSG00000173846**	PLK3	0.67726988	5.487529756	25.59376044	4.61E-06	3.09E-05
**ENSG00000160551**	MIR4523	0.333589341	8.119962305	25.57756665	4.64E-06	3.11E-05
**ENSG00000140941**	MAP1LC3B	0.485788756	6.167683705	25.57730808	4.64E-06	3.11E-05
**ENSG00000075826**	SEC31B	-1.388045679	5.690586136	28.74891844	4.72E-06	3.16E-05
**ENSG00000186951**	PPARA	0.672978255	4.96074784	25.52539268	4.73E-06	3.16E-05
**ENSG00000169118**	CSNK1G1	0.495864295	5.951364604	25.52231479	4.73E-06	3.16E-05
**ENSG00000145020**	AMT	-1.079151504	4.060386106	25.50448042	4.76E-06	3.18E-05
**ENSG00000179088**	C12orf42	-1.107637502	3.670353191	25.49962058	4.77E-06	3.18E-05
**ENSG00000147443**	dok2	0.680105261	5.019881978	25.49600567	4.78E-06	3.18E-05
**ENSG00000181915**	ADO	0.68807052	4.570240326	25.47234662	4.82E-06	3.21E-05
**ENSG00000005206**	SPPL2B	-1.323961898	4.467477733	27.05633183	4.83E-06	3.22E-05
**ENSG00000134287**	ARF3	0.540539934	5.721884289	25.44147876	4.87E-06	3.24E-05
**ENSG00000139971**	C14orf37	-0.895510294	4.076728699	25.41693353	4.91E-06	3.27E-05
**ENSG00000011258**	MBTD1	-0.564626074	5.906734362	25.408721	4.93E-06	3.28E-05
**ENSG00000100083**	GGA1	-0.610499116	5.329426889	25.40513007	4.93E-06	3.28E-05
**ENSG00000010322**	NISCH	-0.765838097	6.351086162	27.4210511	4.96E-06	3.29E-05
**ENSG00000198689**	SLC9A6	0.562634889	5.591068573	25.3761347	4.99E-06	3.31E-05
**ENSG00000166881**	NEMP1	-0.59458279	5.513446178	25.36983757	5E-06	3.31E-05
**ENSG00000184068**		0.731967952	4.30268775	25.35538104	5.02E-06	3.33E-05
**ENSG00000140403**	DNAJA4	-0.738578987	5.002075634	25.33296541	5.06E-06	3.35E-05
**ENSG00000280800**		1.596745529	4.919524009	28.43653886	5.14E-06	3.4E-05
**ENSG00000243708**	PLA2G4B	-1.29646242	4.998391565	27.81652285	5.15E-06	3.41E-05
**ENSG00000204628**	RACK1	0.431616923	9.844227954	25.53912519	5.19E-06	3.43E-05
**ENSG00000104205**	SGK3	0.533649959	5.554667454	25.25987511	5.2E-06	3.43E-05
**ENSG00000091542**	ALKBH5	0.463849601	6.372945744	25.24864949	5.22E-06	3.45E-05
**ENSG00000139508**	SLC46A3	-0.611257785	5.259734411	25.24352703	5.23E-06	3.45E-05
**ENSG00000198466**	ZNF587	-0.505551449	5.944109143	25.23787661	5.24E-06	3.45E-05
**ENSG00000104218**	CSPP1	-0.58426048	5.735302612	25.228324	5.26E-06	3.46E-05
**ENSG00000281383**		1.436832926	6.517711396	28.45270112	5.26E-06	3.46E-05
**ENSG00000172081**	MOB3A	0.463186803	7.515277696	25.68190872	5.27E-06	3.47E-05

**ENSG00000169230**	PRELID1	0.609181268	4.870786959	25.20748083	5.3E-06	3.48E-05
**ENSG00000143851**	PTPN7	0.537680979	5.764697021	25.19736399	5.32E-06	3.49E-05
**ENSG00000040275**	SPDL1	-1.027058349	3.875690858	25.16912663	5.37E-06	3.53E-05
**ENSG00000119397**	CNTRL	-0.37232816	8.359068519	25.16804355	5.37E-06	3.53E-05
**ENSG00000139192**	TAPBPL	-0.527272908	5.779582843	25.16317708	5.38E-06	3.53E-05
**ENSG00000242086**	SDHAP2	-0.522058832	6.657978267	25.19855308	5.42E-06	3.55E-05
**ENSG00000007129**	CEACAM21	-0.592606077	5.298322433	25.12576589	5.46E-06	3.57E-05
**ENSG00000267368**	UPK3BL	-0.911470627	4.36569418	25.11260729	5.48E-06	3.59E-05
**ENSG00000114021**	NIT2	-0.66251339	5.547375263	25.10946816	5.49E-06	3.59E-05
**ENSG00000130713**	EXOSC2	-0.711333286	5.022279311	25.06862385	5.57E-06	3.64E-05
**ENSG00000166501**	PRKCB	0.404988988	7.876442104	25.03799679	5.63E-06	3.68E-05
**ENSG00000102390**	PBDC1	0.812775942	4.329708684	25.033748	5.64E-06	3.68E-05
**ENSG00000180776**	ZDHHC20	0.410445762	7.251243292	25.03343003	5.64E-06	3.68E-05
**ENSG00000166507**	NDST2	-0.499958401	6.072641892	25.03245131	5.64E-06	3.68E-05
**ENSG00000100445**	SDR39U1	-0.695198325	5.625887406	25.24892177	5.68E-06	3.7E-05
**ENSG00000127054**	MIR6727	-0.601583438	5.894297006	25.00467091	5.7E-06	3.71E-05
**ENSG00000180867**		0.906016098	3.754727834	25.0036672	5.7E-06	3.71E-05
**ENSG00000155893**	PXYLP1	-0.784254178	4.752901119	24.96175699	5.79E-06	3.77E-05
**ENSG00000121964**	GTDC1	0.844839589	4.471281278	24.94766548	5.82E-06	3.78E-05
**ENSG00000119318**	RAD23B	0.346991883	6.808116814	24.93871763	5.84E-06	3.79E-05
**ENSG00000104859**	CLASRP	-0.829233096	5.113469587	25.31378515	5.85E-06	3.8E-05
**ENSG00000138964**	PARVG	-0.731828179	6.225980232	26.48344005	5.87E-06	3.81E-05
**ENSG00000183283**	DAZAP2	0.313391773	8.316624963	24.9087505	5.9E-06	3.83E-05
**ENSG00000278274**	Snora61	2.335923467	5.012021054	28.05237783	5.9E-06	3.83E-05
**ENSG00000175463**	TBC1D10C	-0.824720324	6.659304409	27.51850168	5.95E-06	3.86E-05
**ENSG00000206760**	SNORA6	1.13146805	3.785551018	24.88024494	5.96E-06	3.86E-05
**ENSG00000107669**	ATE1	0.591118947	5.631748941	24.87804686	5.97E-06	3.86E-05
**ENSG00000245532**	NEAT1	-0.924062076	10.03882095	27.99120032	5.97E-06	3.86E-05
**ENSG00000131873**	CHSY1	0.748415978	4.738144615	24.85281532	6.02E-06	3.89E-05
**ENSG00000171603**	CLSTN1	0.335531918	7.302137097	24.84950225	6.03E-06	3.89E-05
**ENSG00000124570**	SERPINB6	-0.803892159	4.425934946	24.82814479	6.07E-06	3.92E-05
**ENSG00000139163**	ETNK1	0.366529192	7.452187981	24.82699358	6.08E-06	3.92E-05
**ENSG00000233893**		0.986970187	3.54231087	24.82652148	6.08E-06	3.92E-05
**ENSG00000182831**	C16orf72	0.311145923	8.606799912	24.81670747	6.1E-06	3.93E-05
**ENSG00000126860**	EVI2A	0.466597925	7.20342077	25.08619888	6.14E-06	3.95E-05
**ENSG00000265808**	SEC22B	-0.428973374	6.154499188	24.78249525	6.18E-06	3.97E-05
**ENSG00000174483**	BBS1	-0.856353013	4.492658631	24.75945689	6.23E-06	4.01E-05

**ENSG00000253729**	PRKDC	0.306834158	8.603362141	24.75535701	6.24E-06	4.01E-05
**ENSG00000148834**	GSTO1	0.803725082	4.03422426	24.75410102	6.24E-06	4.01E-05
**ENSG00000267121**		-1.28158936	5.02053231	27.14081907	6.3E-06	4.04E-05
**ENSG00000134899**	ERCC5	-0.316579626	7.57662658	24.67019604	6.43E-06	4.13E-05
**ENSG00000169442**	CD52	0.574300537	8.221975186	26.72649337	6.47E-06	4.15E-05
**ENSG00000120832**	MTERF2	-0.860621984	4.396888447	24.63852782	6.51E-06	4.17E-05
**ENSG00000152767**	FARP1	0.387337899	6.507206344	24.61937653	6.55E-06	4.2E-05
**ENSG00000239665**		-0.740412164	5.839045494	25.64532675	6.56E-06	4.2E-05
**ENSG00000182621**	PLCB1	1.086676209	4.441985577	25.40790066	6.59E-06	4.22E-05
**ENSG00000171302**	CANT1	0.564195959	4.89162861	24.59721733	6.61E-06	4.22E-05
**ENSG00000092094**	OSGEP	-1.024532187	5.076250794	26.00389326	6.62E-06	4.23E-05
**ENSG00000249115**	HAUS5	-0.928701812	4.240487001	24.55707043	6.7E-06	4.28E-05
**ENSG00000103168**	TAF1C	-1.219283352	4.749478454	26.28637868	6.71E-06	4.28E-05
**ENSG00000135390**	ATP5G2	0.633583781	6.416538861	25.75423697	6.78E-06	4.32E-05
**ENSG00000121481**	RNF2	0.515104361	5.534568039	24.49521478	6.85E-06	4.37E-05
**ENSG00000198843**	SELENOT	0.503989926	6.103990136	24.49032614	6.87E-06	4.37E-05
**ENSG00000102125**	TAZ	-1.014061821	4.583480241	24.86320463	6.9E-06	4.39E-05
**ENSG00000103489**	XYLT1	0.528605503	5.942528664	24.46452572	6.93E-06	4.41E-05
**ENSG00000185728**	YTHDF3	0.336744975	6.96512231	24.44678615	6.98E-06	4.43E-05
**ENSG00000071537**	SEL1L	0.380751877	7.086037022	24.41527845	7.06E-06	4.48E-05
**ENSG00000140406**	mesdc1	0.884086792	4.402270993	24.41227169	7.06E-06	4.49E-05
**ENSG00000128872**	TMOD2	-0.549030518	5.695794397	24.39300063	7.11E-06	4.51E-05
**ENSG00000158985**	CDC42SE2	0.324013352	9.131367497	24.39226875	7.12E-06	4.51E-05
**ENSG00000119537**	KDSR	0.634452702	5.970019421	25.04556805	7.14E-06	4.52E-05
**ENSG00000168092**	PAFAH1B2	0.463836436	6.236212715	24.37424658	7.16E-06	4.54E-05
**ENSG00000080371**	RAB21	0.402259492	7.230592093	24.37260159	7.17E-06	4.54E-05
**ENSG00000115415**	STAT1	-0.654588392	8.55788544	26.95518729	7.2E-06	4.55E-05
**ENSG00000119335**	SET	0.38726412	8.306454725	24.35930272	7.2E-06	4.55E-05
**ENSG00000206680**	Snord21	1.582776209	4.104241919	26.68952576	7.25E-06	4.58E-05
**ENSG00000280137**		-0.965340658	4.791128935	24.93326751	7.39E-06	4.67E-05
**ENSG00000122223**	CD244	-0.991704197	3.624850769	24.26898532	7.44E-06	4.7E-05
**ENSG00000102780**	DGKH	0.480169617	6.754687294	24.2659721	7.45E-06	4.7E-05
**ENSG00000166971**	AKTIP	-0.363911565	7.302347544	24.25803186	7.47E-06	4.71E-05
**ENSG00000122545**	39326	0.330776267	8.099772654	24.24765597	7.5E-06	4.73E-05
**ENSG00000166130**	IKBIP	1.02535448	3.616059897	24.22489239	7.56E-06	4.76E-05
**ENSG00000243207**	PPAN-P2RY11	-0.739477527	4.901616098	24.21829195	7.58E-06	4.77E-05
**ENSG00000081870**	HSPB11	-0.778602487	4.498749007	24.21359469	7.59E-06	4.78E-05

**ENSG00000134283**	PPHLN1	-0.379997228	6.875751291	24.17493307	7.7E-06	4.84E-05
**ENSG00000006576**	PHTF2	0.404430574	6.851860488	24.16935372	7.72E-06	4.85E-05
**ENSG00000114867**	EIF4G1	0.323489084	7.47201409	24.16393058	7.73E-06	4.85E-05
**ENSG00000064201**	TSPAN32	-0.986555955	4.570332388	24.37689803	7.78E-06	4.88E-05
**ENSG00000104331**	IMPAD1	0.410185695	6.233477179	24.13487222	7.82E-06	4.9E-05
**ENSG00000134684**	YARS	0.520966677	5.511385132	24.12280725	7.85E-06	4.92E-05
**ENSG00000213186**	trim59	0.478498736	6.304850131	24.10740653	7.9E-06	4.94E-05
**ENSG00000165516**	KLHDC2	-0.452710088	6.743519917	24.08154477	7.97E-06	4.99E-05
**ENSG00000279753**		0.62587622	5.891575185	24.40491312	8.02E-06	5.02E-05
**ENSG00000158773**	USF1	-0.621254664	4.776919299	24.05484804	8.05E-06	5.03E-05
**ENSG00000122965**	RBM19	-0.499571235	5.923758257	24.03655831	8.1E-06	5.06E-05
**ENSG00000172590**	mrpl52	-0.946771342	4.286226354	23.99403082	8.23E-06	5.14E-05
**ENSG00000074706**	IPCEF1	-0.387969553	7.446892204	23.9758205	8.28E-06	5.17E-05
**ENSG00000273419**		-1.036434508	3.967527397	23.97097251	8.3E-06	5.18E-05
**ENSG00000232295**		0.930562074	3.810524787	23.95899966	8.34E-06	5.19E-05
**ENSG00000125347**	IRF1	-0.427985598	7.702300668	24.18121707	8.34E-06	5.2E-05
**ENSG00000178104**	PDE4DIP	0.434482963	6.325980323	23.93801366	8.4E-06	5.23E-05
**ENSG00000019582**	CD74	0.470475244	7.130045725	24.21780769	8.47E-06	5.27E-05
**ENSG00000127824**	tuba4a	0.485264346	6.229804607	23.89521061	8.53E-06	5.3E-05
**ENSG00000204070**	SYS1	0.513094009	5.570643502	23.89329477	8.54E-06	5.3E-05
**ENSG00000279838**		-1.015739773	4.035107974	23.89326112	8.54E-06	5.3E-05
**ENSG00000162642**	C1orf52	-0.634887951	4.941985471	23.88728587	8.56E-06	5.31E-05
**ENSG00000187514**	MIR1244-1	0.419720284	9.200961363	24.19778153	8.71E-06	5.4E-05
**ENSG00000135956**	TMEM127	0.460486715	5.748317843	23.8177479	8.78E-06	5.44E-05
**ENSG00000177082**	WDR73	-0.692177811	6.273181223	25.21208929	8.79E-06	5.45E-05
**ENSG00000210174**		-0.708277601	5.149612182	23.80327785	8.83E-06	5.46E-05
**ENSG00000104133**	SPG11	-0.373412405	7.65199605	23.7917185	8.86E-06	5.48E-05
**ENSG00000262160**		0.394014286	6.314058045	23.78376509	8.89E-06	5.5E-05
**ENSG00000153147**	SMARCA5	0.375862971	8.060759252	23.77997447	8.9E-06	5.5E-05
**ENSG00000100299**	ARSA	-1.219099522	4.051204515	24.09389114	8.92E-06	5.51E-05
**ENSG00000084093**	REST	0.356144284	7.811968339	23.77072389	8.93E-06	5.51E-05
**ENSG00000134996**	OSTF1	0.450283119	6.06274756	23.73477238	9.05E-06	5.58E-05
**ENSG00000275023**	MLLT6	-0.378960575	8.289148527	23.6915472	9.2E-06	5.67E-05
**ENSG00000137413**	TAF8	-0.460838498	5.779604511	23.65737745	9.31E-06	5.74E-05
**ENSG00000272578**		-1.109832802	5.553987474	26.09767532	9.34E-06	5.75E-05
**ENSG00000267369**		0.805141527	4.932954045	23.86788464	9.37E-06	5.77E-05
**ENSG00000068697**	laptm4a	0.653230244	5.324966381	23.63620071	9.38E-06	5.77E-05

**ENSG00000131778**	CHD1L	-0.665789859	5.076201345	23.63466654	9.39E-06	5.77E-05
**ENSG00000086300**	SNX10	0.879481357	4.187062615	23.63430207	9.39E-06	5.77E-05
**ENSG00000155111**	CDK19	0.591512472	5.234299404	23.63127687	9.4E-06	5.77E-05
**ENSG00000117984**	CTSD	0.577647827	5.526351985	23.63117291	9.4E-06	5.77E-05
**ENSG00000133895**	MEN1	-0.732149733	4.671036373	23.6248119	9.42E-06	5.78E-05
**ENSG00000170222**	ADPRM	-0.739571732	4.535360695	23.61265103	9.47E-06	5.8E-05
**ENSG00000075240**	GRAMD4	1.050800388	3.501188247	23.60627588	9.49E-06	5.81E-05
**ENSG00000155189**	AGPAT5	0.626314911	4.898734473	23.59675288	9.52E-06	5.83E-05
**ENSG00000115904**	SOS 1	0.337369474	8.010546222	23.59536744	9.53E-06	5.83E-05
**ENSG00000165934**	CPSF2	0.33649553	6.900180596	23.57832768	9.59E-06	5.86E-05
**ENSG00000176595**	KBTBD11	-1.044767894	3.926504165	23.57243667	9.61E-06	5.87E-05
**ENSG00000198001**	IRAK4	-0.451988868	6.547873165	23.55900367	9.65E-06	5.9E-05
**ENSG00000197157**	SND1	0.428853487	6.623498848	23.54334611	9.71E-06	5.93E-05
**ENSG00000111348**	ARHGDIB	0.31652243	9.647542704	23.53866938	9.73E-06	5.93E-05
**ENSG00000107331**	abca2	0.787528512	6.338810759	25.7117489	9.76E-06	5.95E-05
**ENSG00000119242**	CCDC92	0.48590632	5.553825584	23.51658656	9.81E-06	5.98E-05
**ENSG00000111879**	FAM184A	-1.217962009	4.190572713	24.15842937	9.83E-06	5.99E-05
**ENSG00000145780**	FEM1C	0.469155115	6.123838396	23.49208759	9.9E-06	6.02E-05
**ENSG00000247828**	TMEM161B-AS1	-0.795440763	5.037490678	23.59151018	9.91E-06	6.03E-05
**ENSG00000257181**		0.769735679	4.603879562	23.48455999	9.92E-06	6.03E-05
**ENSG00000134262**	AP4B1	-0.856639565	5.353196191	24.42623872	1E-05	6.09E-05
**ENSG00000090097**	PCBP4	0.875200597	3.580111923	23.45276543	1E-05	6.1E-05
**ENSG00000151914**	DST	-1.068097082	4.955039246	24.89874549	1.01E-05	6.12E-05
**ENSG00000077616**	NAALAD2	-0.979555563	3.493868225	23.42676685	1.01E-05	6.15E-05
**ENSG00000184613**	NELL2	-0.713617343	6.920189705	25.59063793	1.02E-05	6.18E-05
**ENSG00000077549**	CAPZB	0.329568337	7.426022996	23.38441547	1.03E-05	6.24E-05
**ENSG00000284707**		-0.570209864	5.186685323	23.33172108	1.05E-05	6.36E-05
**ENSG00000255339**	NDUFB8	-0.507835104	5.54519952	23.32506484	1.05E-05	6.37E-05
**ENSG00000166848**	LOC105371348	0.41938135	6.605390755	23.31065867	1.06E-05	6.4E-05
**ENSG00000124574**	ABCC10	-0.68034711	5.297167219	23.30102206	1.06E-05	6.42E-05
**ENSG00000139921**	TMX1	0.475718285	6.034199631	23.29436382	1.06E-05	6.43E-05
**ENSG00000132334**	PTPRE	0.474458403	5.838418303	23.29223973	1.07E-05	6.43E-05
**ENSG00000162607**	USP1	0.468592105	6.659398737	23.29167173	1.07E-05	6.43E-05
**ENSG00000198556**	ZNF789	-0.827928149	5.117628106	23.7946188	1.07E-05	6.43E-05
**ENSG00000270055**		-1.296293628	3.816469809	23.73031553	1.08E-05	6.48E-05
**ENSG00000105136**	ZNF419	-0.912458188	4.069431075	23.26029504	1.08E-05	6.49E-05
**ENSG00000279541**		0.535902785	5.202269482	23.25323752	1.08E-05	6.51E-05

**ENSG00000187210**	GCNT1	1.043717863	3.591898758	23.25111857	1.08E-05	6.51E-05
**ENSG00000176700**		-0.660596557	6.394350478	24.56654814	1.08E-05	6.52E-05
**ENSG00000269972**		-0.63709051	5.271195684	23.2292729	1.09E-05	6.55E-05
**ENSG00000112697**	TMEM30A	0.364563075	7.35039389	23.22815648	1.09E-05	6.55E-05
**ENSG00000279765**		-0.393760367	7.761072139	23.20531312	1.1E-05	6.6E-05
**ENSG00000169914**	OTUD3	-0.854925628	4.890049524	23.54302031	1.1E-05	6.61E-05
**ENSG00000174799**	CEP135	-0.51544497	6.033633746	23.19595264	1.1E-05	6.62E-05
**ENSG00000183291**	SELENOF	0.511064158	6.637410781	23.47517684	1.11E-05	6.64E-05
**ENSG00000171448**	ZBTB26	-0.956621305	4.130198942	23.1664287	1.12E-05	6.68E-05
**ENSG00000165688**	PMPCA	-0.647136915	4.776117969	23.14616068	1.12E-05	6.73E-05
**ENSG00000130177**	CDC16	-0.469180501	6.594419108	23.12167129	1.13E-05	6.79E-05
**ENSG00000163479**	SSR2	0.503937967	6.591137098	23.3066797	1.15E-05	6.85E-05
**ENSG00000197948**	FCHSD1	-0.780918461	5.423407541	23.74124803	1.15E-05	6.85E-05
**ENSG00000178467**	P4HTM	-0.835894914	4.694675849	23.09384703	1.15E-05	6.85E-05
**ENSG00000101751**	POLI	-0.75539084	5.683991967	24.03388337	1.15E-05	6.85E-05
**ENSG00000165071**	TMEM71	-0.451218541	6.410677817	23.07491338	1.15E-05	6.89E-05
**ENSG00000144535**	DIS3L2	-0.578865666	5.610719012	23.06160682	1.16E-05	6.91E-05
**ENSG00000077150**	NFKB2	-0.674951748	5.139077335	23.06131177	1.16E-05	6.91E-05
**ENSG00000013364**	MVP	-0.439105172	6.489587611	23.05514106	1.16E-05	6.92E-05
**ENSG00000173011**	TADA2B	0.487603659	5.670036001	23.05492836	1.16E-05	6.92E-05
**ENSG00000148672**	GLUD1	0.391258262	6.462044079	23.05004677	1.17E-05	6.93E-05
**ENSG00000263345**		-1.349141321	3.927161846	23.9233571	1.17E-05	6.98E-05
**ENSG00000137409**	MTCH1	-0.521798262	5.800551629	23.01575943	1.18E-05	7.01E-05
**ENSG00000259834**		0.355624326	7.656492239	23.01037079	1.18E-05	7.02E-05
**ENSG00000185513**	L3MBTL1	-1.121887332	3.872274852	23.00066285	1.19E-05	7.04E-05
**ENSG00000160685**	ZBTB7B	0.546397456	5.295225119	22.99098657	1.19E-05	7.06E-05
**ENSG00000134294**	SLC38A2	0.301138748	8.132233267	22.98309057	1.19E-05	7.08E-05
**ENSG00000162777**	DENND2D	-0.344019538	7.813739486	22.91617202	1.22E-05	7.26E-05
**ENSG00000059122**	FLYWCH1	-0.980035907	4.960957052	23.82878773	1.23E-05	7.26E-05
**ENSG00000144021**	CIAO1	-0.525341825	5.981034863	22.89931958	1.23E-05	7.29E-05
**ENSG00000173575**	MIR3175	-0.301397153	9.059790053	22.88887385	1.24E-05	7.31E-05
**ENSG00000256594**	LOC374443	-0.459015251	6.674320036	22.88830303	1.24E-05	7.31E-05
**ENSG00000100243**	cyb5r3	0.565368852	5.032468455	22.88379089	1.24E-05	7.32E-05
**ENSG00000205560**	CPT1B	-1.179427384	4.707988303	24.44515289	1.24E-05	7.34E-05
**ENSG00000230021**	LOC101928626	1.128791912	5.581673437	25.40911316	1.25E-05	7.36E-05
**ENSG00000117091**	CD48	0.565361024	8.272145759	24.79096856	1.25E-05	7.38E-05
**ENSG00000087460**	GNAS	0.295441882	8.947489574	22.84976588	1.26E-05	7.4E-05

**ENSG00000136731**	UGGT1	0.362268737	7.38515482	22.82300598	1.27E-05	7.47E-05
**ENSG00000087087**	SRRT	-0.460768296	6.155135051	22.80566175	1.28E-05	7.51E-05
**ENSG00000131797**	CLUHP3	-0.680758858	5.602178476	23.00575949	1.28E-05	7.54E-05
**ENSG00000066651**	TRMT11	-0.654261454	5.006993977	22.7873881	1.28E-05	7.56E-05
**ENSG00000156639**	ZFAND3	0.474793173	5.480725333	22.78437939	1.29E-05	7.56E-05
**ENSG00000112242**	E2F3	0.673844999	4.826904703	22.75838239	1.3E-05	7.63E-05
**ENSG00000266028**	SRGAP2	0.740191141	4.685856056	22.74871224	1.3E-05	7.65E-05
**ENSG00000173120**	KDM2A	-0.286875237	7.494795007	22.74271461	1.31E-05	7.67E-05
**ENSG00000170776**	MIR7706	0.244092792	9.055526553	22.71607317	1.32E-05	7.74E-05
**ENSG00000181830**	SLC35C1	0.745893434	4.12280145	22.70909265	1.32E-05	7.75E-05
**ENSG00000003400**	CASP10	-0.581107228	6.002197366	22.91693759	1.33E-05	7.78E-05
**ENSG00000174652**	ZNF266	-0.615469824	6.195346988	23.36045719	1.33E-05	7.8E-05
**ENSG00000153201**	RANBP2	0.289642033	8.515059377	22.66362692	1.35E-05	7.87E-05
**ENSG00000139266**	39873	-0.766030605	4.848165954	22.64132215	1.36E-05	7.93E-05
**ENSG00000272888**		-0.652880503	7.56906358	24.83900495	1.36E-05	7.94E-05
**ENSG00000059145**	UNKL	-0.627447263	5.235579347	22.62579446	1.36E-05	7.97E-05
**ENSG00000148660**	CAMK2G	-0.531086767	5.959322162	22.62508571	1.36E-05	7.97E-05
**ENSG00000108797**	CNTNAP1	-1.223475853	4.084551276	23.24554697	1.37E-05	7.97E-05
**ENSG00000163743**	RCHY1	0.458738334	5.767792036	22.61222305	1.37E-05	8E-05
**ENSG00000177225**	PDDC1	-0.819454991	4.442864421	22.60159303	1.38E-05	8.03E-05
**ENSG00000175567**	UCP2	-0.368042078	7.878764873	22.59856815	1.38E-05	8.03E-05
**ENSG00000174197**	MGA	0.282499639	7.820762347	22.57365419	1.39E-05	8.1E-05
**ENSG00000164620**	RELL2	-0.997559522	3.786046709	22.56850959	1.39E-05	8.11E-05
**ENSG00000236991**	EDRF1-AS1	-0.891748226	4.14064065	22.56469268	1.4E-05	8.12E-05
**ENSG00000105401**	MIR1181	-0.454168475	5.941839061	22.56187653	1.4E-05	8.12E-05
**ENSG00000133030**	MPRIP	-0.297973215	8.125611067	22.54225747	1.41E-05	8.18E-05
**ENSG00000139679**	lpar6	-0.553031061	6.894855045	23.51149231	1.41E-05	8.19E-05
**ENSG00000137075**	RNF38	0.305274187	7.366490916	22.5313073	1.41E-05	8.2E-05
**ENSG00000105879**	CBLL1	0.395986633	7.101314593	22.52416314	1.42E-05	8.22E-05
**ENSG00000119655**	MIR4709	0.797429175	4.511400583	22.51452121	1.42E-05	8.24E-05
**ENSG00000172059**	KLF11	0.865331397	4.206089737	22.50110387	1.43E-05	8.28E-05
**ENSG00000183484**	GPR132	0.639655786	5.241965643	22.49549524	1.43E-05	8.29E-05
**ENSG00000154001**	PPP2R5E	0.36421573	6.725970816	22.49444077	1.43E-05	8.29E-05
**ENSG00000160932**	LY6E	0.646680018	4.990306985	22.47529473	1.44E-05	8.35E-05
**ENSG00000184588**	PDE4B	0.39198456	6.74895844	22.45981269	1.45E-05	8.39E-05
**ENSG00000185246**	PRPF39	-0.657984966	6.282291269	23.63503385	1.46E-05	8.45E-05
**ENSG00000166900**	STX3	0.822907097	4.298727617	22.43345781	1.47E-05	8.47E-05

**ENSG00000172922**	rnaseh2c	-0.614218501	4.930166751	22.42199067	1.47E-05	8.5E-05
**ENSG00000274266**	Snora73a	1.960454807	4.971099954	24.97834309	1.48E-05	8.53E-05
**ENSG00000089006**	SNX5	0.38315166	6.641581794	22.40337083	1.48E-05	8.55E-05
**ENSG00000200087**		2.147851751	4.98583261	24.97040681	1.48E-05	8.55E-05
**ENSG00000213928**	IRF9	-0.641507175	6.153084832	23.2679883	1.49E-05	8.58E-05
**ENSG00000108100**	CCNY	0.383196421	6.3066653	22.37221847	1.5E-05	8.63E-05
**ENSG00000164048**	ZNF589	-0.800534548	5.046314142	22.56874872	1.5E-05	8.63E-05
**ENSG00000141522**	ARHGDIA	0.433077816	6.601810829	22.34899777	1.51E-05	8.7E-05
**ENSG00000136273**	HUS1	-0.425977742	5.853809597	22.34405512	1.52E-05	8.71E-05
**ENSG00000100003**	SEC14L2	-1.033797482	3.994601606	22.3412445	1.52E-05	8.71E-05
**ENSG00000115211**	EIF2B4	-0.714568513	4.916556508	22.33635374	1.52E-05	8.72E-05
**ENSG00000092201**	SUPT16H	0.300966197	7.260712188	22.33609776	1.52E-05	8.72E-05
**ENSG00000151151**	IPMK	0.60288875	4.774779396	22.3281907	1.53E-05	8.74E-05
**ENSG00000144567**	FAM134A	0.392463961	6.054759797	22.32174966	1.53E-05	8.76E-05
**ENSG00000166224**	SGPL1	0.532720775	5.358126231	22.30599459	1.54E-05	8.81E-05
**ENSG00000171503**	ETFDH	-0.725849231	4.600693005	22.29363049	1.54E-05	8.84E-05
**ENSG00000103051**	COG4	-0.608778341	5.270759743	22.28460506	1.55E-05	8.87E-05
**ENSG00000262580**		-1.367691044	4.212262976	23.47217766	1.58E-05	9E-05
**ENSG00000106615**	RHEB	0.655042965	4.324086401	22.23757654	1.58E-05	9.02E-05
**ENSG00000184716**	SERINC4	-0.694312801	4.678158572	22.20798803	1.6E-05	9.11E-05
**ENSG00000020922**	MRE11	-0.463634937	6.0176923	22.16623664	1.62E-05	9.25E-05
**ENSG00000112033**	PPARD	-0.576626945	5.306939893	22.15982167	1.62E-05	9.27E-05
**ENSG00000175348**	TMEM9B	0.583635251	5.310274794	22.12247512	1.65E-05	9.39E-05
**ENSG00000139211**	AMIGO2	-0.6954933	4.477943591	22.10445359	1.66E-05	9.45E-05
**ENSG00000144320**	LNPK	0.450375733	6.020012064	22.09887628	1.66E-05	9.47E-05
**ENSG00000136240**	KDELR2	0.498007498	5.643353589	22.09421752	1.66E-05	9.48E-05
**ENSG00000070669**	ASNS	-0.579993578	4.998973292	22.08084356	1.67E-05	9.52E-05
**ENSG00000162032**	SPSB3	-0.968888224	4.972653857	23.1894611	1.68E-05	9.55E-05
**ENSG00000242071**		0.793472587	5.081985419	22.65822866	1.68E-05	9.57E-05
**ENSG00000140688**	c16orf58	-0.673568002	5.132806477	22.0612333	1.69E-05	9.58E-05
**ENSG00000141580**	WDR45B	-0.552136164	5.186565187	22.04959521	1.69E-05	9.61E-05
**ENSG00000249786**		0.626371034	4.681027883	22.03947332	1.7E-05	9.64E-05
**ENSG00000142188**	TMEM50B	-0.407967386	6.14045923	22.02788351	1.71E-05	9.68E-05
**ENSG00000269987**		-0.664945206	5.238106031	22.02671094	1.71E-05	9.68E-05
**ENSG00000134905**	CARS2	-0.594339159	5.564588846	22.01114873	1.72E-05	9.73E-05
**ENSG00000141424**	SLC39A6	0.501084705	5.199362979	22.01107193	1.72E-05	9.73E-05
**ENSG00000163378**	EOGT	0.600988304	5.099111931	22.00448792	1.72E-05	9.74E-05

**ENSG00000113575**	PPP2CA	0.330565085	6.839707239	22.00417659	1.72E-05	9.74E-05
**ENSG00000070718**	AP3M2	-0.558817022	5.880961159	22.0004948	1.72E-05	9.75E-05
**ENSG00000172578**	KLHL6	-0.379349558	6.669640186	21.96365067	1.75E-05	9.88E-05
**ENSG00000277157**	HIST1H4D	1.577689159	3.983055901	23.89941152	1.75E-05	9.9E-05
**ENSG00000165526**	RPUSD4	-0.724481232	4.517486921	21.94621045	1.76E-05	9.93E-05
**ENSG00000159322**	ADPGK	-0.530231821	6.000666268	21.94242576	1.76E-05	9.94E-05
**ENSG00000148218**	ALAD	-0.60426261	4.768129691	21.93798305	1.77E-05	9.95E-05
**ENSG00000185104**	FAF1	0.421623059	5.80309929	21.93504026	1.77E-05	9.96E-05
**ENSG00000125505**	MBOAT7	0.759778085	4.139223222	21.91827374	1.78E-05	0.0001001
**ENSG00000087338**	GMCL1	0.442704928	5.875830873	21.89931307	1.79E-05	0.0001008
**ENSG00000106591**	MRPL32	-0.694991416	4.886219257	21.88853089	1.8E-05	0.0001011
**ENSG00000243302**		-0.845879249	4.831289848	22.07126273	1.8E-05	0.0001014
**ENSG00000008294**	SPAG9	0.373773711	6.827982798	21.87343694	1.81E-05	0.0001016
**ENSG00000263072**		-0.784911906	4.300306164	21.86800478	1.81E-05	0.0001018
**ENSG00000123219**	CENPK	-0.88226251	5.47526739	22.95380409	1.82E-05	0.0001020
**ENSG00000254999**	BRK1	0.642033691	5.038653655	21.8373377	1.83E-05	0.0001028
**ENSG00000071054**	map4k4	-0.324874685	7.707821282	21.82740578	1.84E-05	0.0001032
**ENSG00000188636**	LDOC1L	0.733322109	4.545706092	21.79634267	1.86E-05	0.0001043
**ENSG00000163082**	SGPP2	1.064620341	3.819325057	21.77646867	1.88E-05	0.0001051
**ENSG00000198826**	ARHGAP11A	0.885395434	3.989678141	21.74043002	1.9E-05	0.0001064
**ENSG00000185591**	SP1	0.30908959	7.666933568	21.73350866	1.91E-05	0.0001067
**ENSG00000281887**	GIMAP1-GIMAP5	-0.333404152	7.651798082	21.72743598	1.91E-05	0.0001069
**ENSG00000111276**	CDKN1B	0.341886878	7.694061224	21.70937506	1.92E-05	0.0001075
**ENSG00000271964**		-1.062239218	4.528779219	22.48382388	1.92E-05	0.0001075
**ENSG00000267645**	POLR2J2	-0.808857359	4.52911179	21.6971477	1.93E-05	0.0001079
**ENSG00000082458**	DLG3	0.918085153	4.308162567	21.69021765	1.94E-05	0.0001082
**ENSG00000099821**	POLRMT	-0.776327561	4.535293705	21.67848621	1.95E-05	0.0001086
**ENSG00000160072**	atad3b	-1.005816999	4.351920859	21.89738078	1.95E-05	0.0001086
**ENSG00000137171**	KLC4	-1.053021871	3.837964306	21.66774304	1.96E-05	0.0001089
**ENSG00000250644**		0.593538359	5.183765584	21.66017979	1.96E-05	0.0001091
**ENSG00000145868**	FBXO38	-0.432092024	6.357211151	21.64652068	1.97E-05	0.0001096
**ENSG00000059378**	PARP12	-0.574536881	6.144889662	21.99437434	1.97E-05	0.0001096
**ENSG00000134480**	CCNH	-0.429048895	6.597532879	21.64474403	1.97E-05	0.0001096
**ENSG00000105726**	ATP13A1	-0.560109884	5.87873622	21.63486458	1.98E-05	0.0001100
**ENSG00000284482**		0.800411194	4.210743177	21.62580662	1.99E-05	0.0001103
**ENSG00000135018**	UBQLN1	0.294477183	7.699089725	21.61197876	2E-05	0.0001108
**ENSG00000198677**	TTC37	-0.304224049	7.634519836	21.59081085	2.01E-05	0.0001116

**ENSG00000158863**	FAM160B2	-0.923822478	5.205591475	22.74861143	2.02E-05	0.0001118
**ENSG00000132879**	FBXO44	-0.937526181	4.28912443	21.57573681	2.02E-05	0.0001122
**ENSG00000035403**	VCL	0.450149451	6.642610289	21.57230319	2.03E-05	0.0001122
**ENSG00000211689**	TARP	0.904995541	3.996061504	21.56069683	2.04E-05	0.0001127
**ENSG00000181744**	c3orf58	0.594744512	5.421108439	21.55711532	2.04E-05	0.0001128
**ENSG00000188878**	FBF1	-1.125765989	3.711015379	21.55322373	2.04E-05	0.0001129
**ENSG00000164039**	BDH2	-0.901324741	4.055082857	21.55144954	2.04E-05	0.0001129
**ENSG00000026036**	RTEL1-TNFRSF6	-0.764205002	4.713103831	21.54032218	2.05E-05	0.0001133
**ENSG00000120129**	DUSP1	-0.834316209	6.023452156	23.33452803	2.06E-05	0.0001136
**ENSG00000102053**	zc3h12b	-1.053585847	3.840338121	21.52933656	2.06E-05	0.0001136
**ENSG00000160226**	c21orf2	-1.01235091	4.338444531	21.73332781	2.06E-05	0.0001136
**ENSG00000187837**	HIST1H1C	0.943312099	7.378336793	23.88797124	2.07E-05	0.0001140
**ENSG00000100614**	PPM1A	0.361159123	6.775151874	21.5062888	2.08E-05	0.0001145
**ENSG00000109083**	IFT20	-0.962246376	3.778235893	21.47388655	2.1E-05	0.0001158
**ENSG00000244716**		0.985547968	4.35844795	22.00985422	2.11E-05	0.0001161
**ENSG00000085982**	USP40	-0.805712812	4.638870873	21.46098151	2.11E-05	0.0001163
**ENSG00000177192**	PUS1	-0.832128029	4.100213653	21.446229	2.13E-05	0.0001169
**ENSG00000100030**	MAPK1	0.324556168	7.491521371	21.44500812	2.13E-05	0.0001169
**ENSG00000149231**	CCDC82	-0.429554411	6.335151043	21.43501666	2.14E-05	0.0001172
**ENSG00000201098**	rny1	1.901966001	6.049482238	23.77944945	2.15E-05	0.0001178
**ENSG00000112335**	SNX3	0.557022512	5.615202501	21.41949854	2.15E-05	0.0001178
**ENSG00000133624**	ZNF767P	-0.851900725	4.996447559	21.90059373	2.15E-05	0.0001181
**ENSG00000079134**	THOC1	-0.426994194	6.361177822	21.40991935	2.16E-05	0.0001181
**ENSG00000121644**	DESI2	0.554625222	5.111949342	21.40637365	2.16E-05	0.0001182
**ENSG00000104626**	ERI1	0.568920263	4.945470958	21.39190028	2.17E-05	0.0001188
**ENSG00000156471**	PTDSS1	0.457564126	6.255295274	21.38680009	2.18E-05	0.0001190
**ENSG00000178685**	PARP10	-0.937496177	4.678488272	21.86618626	2.18E-05	0.0001192
**ENSG00000105397**	TYK2	-0.689739982	6.039322309	22.48171561	2.18E-05	0.0001192
**ENSG00000179918**	SEPHS2	0.601759273	5.149222577	21.37731191	2.18E-05	0.0001192
**ENSG00000188229**	TUBB4B	0.707198737	4.393842311	21.37682474	2.18E-05	0.0001192
**ENSG00000100058**	CRYBB2P1	-0.866602123	4.522986472	21.35824665	2.2E-05	0.0001200
**ENSG00000237441**	RGL2	-0.983000886	4.684205487	22.04027136	2.21E-05	0.0001203
**ENSG00000102034**	ELF4	0.393791453	6.088460865	21.33075234	2.22E-05	0.0001211
**ENSG00000137767**	SQRDL	0.664334882	4.868531077	21.30150545	2.25E-05	0.0001224
**ENSG00000267458**		0.867697174	3.6235731	21.29584867	2.25E-05	0.0001226
**ENSG00000204946**	ZNF783	-0.900335145	5.005123024	22.09972882	2.26E-05	0.0001229
**ENSG00000075336**	TIMM21	-0.971705384	3.74393408	21.28017788	2.27E-05	0.0001232

**ENSG00000185164**	LOC102723728	0.701670179	4.098336895	21.27436602	2.27E-05	0.0001234
**ENSG00000257315**	ZBED6	-0.279381814	8.436192728	21.26384541	2.28E-05	0.0001238
**ENSG00000107290**	SETX	0.279988211	8.756017841	21.25080429	2.29E-05	0.0001244
**ENSG00000185261**	KIAA0825	0.976843994	3.861013763	21.24972425	2.29E-05	0.0001244
**ENSG00000278259**	MYO19	-0.589726762	5.614687811	21.24794625	2.29E-05	0.0001244
**ENSG00000180357**	ZNF609	-0.320162181	7.229176263	21.24675638	2.29E-05	0.0001244
**ENSG00000073169**	SELENOO	-1.017445098	3.979310712	21.23397087	2.31E-05	0.0001249
**ENSG00000166436**	TRIM66	-1.036759108	4.888407051	22.39303644	2.31E-05	0.0001253
**ENSG00000233913**		0.7842177	5.369623937	22.14188864	2.33E-05	0.0001260
**ENSG00000184445**	KNTC1	-0.530369342	5.878870772	21.20266019	2.33E-05	0.0001262
**ENSG00000121454**	LHX4	-0.953653667	4.535974597	21.45205166	2.33E-05	0.0001262
**ENSG00000084234**	APLP2	0.447549397	5.949497031	21.18667081	2.35E-05	0.0001269
**ENSG00000169410**	PTPN9	0.588625235	5.13292031	21.17794511	2.35E-05	0.0001272
**ENSG00000254806**	SYS1-DBNDD2	0.956275844	3.728655423	21.16559364	2.37E-05	0.0001277
**ENSG00000150712**	MTMR12	0.465291084	5.580957735	21.16404908	2.37E-05	0.0001278
**ENSG00000103423**	DNAJA3	-0.701274257	5.103014582	21.15569875	2.37E-05	0.0001281
**ENSG00000110723**	EXPH5	-1.051270491	3.908691928	21.14538574	2.38E-05	0.0001285
**ENSG00000111450**	STX2	-0.610963257	5.493033371	21.13575051	2.39E-05	0.0001289
**ENSG00000238741**	SCARNA7	1.168226621	7.291964	23.42040716	2.4E-05	0.0001294
**ENSG00000117394**	SLC2A1	0.525991033	5.144085896	21.11746752	2.41E-05	0.0001297
**ENSG00000174943**	kctd13	-1.025073331	4.316371118	21.30446338	2.42E-05	0.0001303
**ENSG00000122515**	ZMIZ2	-0.690036478	6.245649426	22.43827399	2.43E-05	0.0001308
**ENSG00000113597**	TRAPPC13	-0.472264858	5.918405033	21.09095244	2.43E-05	0.0001308
**ENSG00000258727**	LOC102724814	-0.864458583	5.864831257	22.84066639	2.46E-05	0.0001320
**ENSG00000152117**	LOC150776	-0.740805566	4.424362104	21.0641678	2.46E-05	0.0001320
**ENSG00000174231**	PRPF8	0.271369946	9.161098932	21.06058286	2.46E-05	0.0001321
**ENSG00000225178**		0.705258799	5.95446025	22.30625312	2.48E-05	0.0001328
**ENSG00000139718**	SETD1B	-0.428319722	6.969890338	21.03994904	2.48E-05	0.0001330
**ENSG00000196470**	SIAH1	-0.516928751	5.528529792	21.03869594	2.48E-05	0.0001330
**ENSG00000126870**	WDR60	-0.553360806	5.215422645	21.01625781	2.5E-05	0.0001341
**ENSG00000226221**		1.036523199	5.282417013	22.91377382	2.52E-05	0.0001346
**ENSG00000266402**	SNHG25	-1.214541729	4.06988486	21.55168163	2.53E-05	0.0001353
**ENSG00000197694**	SPTAN1	0.273889417	9.484248535	20.98249333	2.54E-05	0.0001357
**ENSG00000264112**		-0.997050141	5.191651715	22.37482143	2.54E-05	0.0001358
**ENSG00000263272**		-0.910567234	4.000145486	20.97431867	2.55E-05	0.0001359
**ENSG00000135837**	cep350	0.323261912	8.408544379	20.94043896	2.58E-05	0.0001376
**ENSG00000150347**	ARID5B	0.313144558	7.792482655	20.93921795	2.58E-05	0.0001376

**ENSG00000102172**	SMS	0.607972903	4.769654159	20.93054955	2.59E-05	0.0001380
**ENSG00000198898**	CAPZA2	0.426650247	6.258786588	20.92573435	2.59E-05	0.0001382
**ENSG00000284691**		-0.833130543	4.455808659	20.91921452	2.6E-05	0.0001385
**ENSG00000134852**	CLOCK	0.43914731	6.477437159	20.87304378	2.65E-05	0.0001409
**ENSG00000089022**	MAPKAPK5	-0.483034659	6.614482736	21.03385944	2.65E-05	0.0001411
**ENSG00000105643**	ARRDC2	-0.550902836	5.29219423	20.86273951	2.66E-05	0.0001413
**ENSG00000145882**	PCYOX1L	-0.600842785	5.317311864	20.85910552	2.66E-05	0.0001414
**ENSG00000116191**	RALGPS2	-0.919547758	4.601837123	21.15829187	2.66E-05	0.0001414
**ENSG00000058673**	ZC3H11A	-0.276479403	8.438260099	20.84693745	2.67E-05	0.0001419
**ENSG00000162591**	MEGF6	-1.078164879	4.725743099	21.92539128	2.69E-05	0.0001428
**ENSG00000104907**	TRMT1	-1.026745581	4.587859887	21.60470963	2.69E-05	0.0001429
**ENSG00000181904**	C5orf24	-0.476715555	5.857669959	20.80332924	2.72E-05	0.0001441
**ENSG00000253645**		0.907292392	4.002500815	20.79441131	2.73E-05	0.0001445
**ENSG00000163932**	PRKCD	0.850401177	4.037003676	20.79186304	2.73E-05	0.0001446
**ENSG00000111269**	CREBL2	0.357094648	6.673322787	20.78866935	2.73E-05	0.0001447
**ENSG00000132600**	PRMT7	-0.556029852	5.429512397	20.78697892	2.73E-05	0.0001447
**ENSG00000116106**	EPHA4	0.54387586	6.059492496	20.97029885	2.74E-05	0.0001451
**ENSG00000185651**	UBE2L3	0.477397102	5.844855824	20.77526599	2.75E-05	0.0001452
**ENSG00000144744**	UBA3	-0.41505312	6.281921994	20.77423502	2.75E-05	0.0001452
**ENSG00000108515**	ENO3	0.585179068	6.248457615	21.5247871	2.76E-05	0.0001456
**ENSG00000178996**	SNX18	0.530608331	5.564085113	20.74775437	2.78E-05	0.0001465
**ENSG00000269737**		-0.677349158	4.207697958	20.74664052	2.78E-05	0.0001465
**ENSG00000165502**	RPL36AL	0.681084044	7.463508835	22.76195615	2.81E-05	0.0001481
**ENSG00000172469**	MANEA	0.57492729	5.394613975	20.71128214	2.81E-05	0.0001483
**ENSG00000125450**	NUP85	-0.749906616	5.095063632	20.86667934	2.82E-05	0.0001483
**ENSG00000187017**	ESPN	-0.937252807	4.318578344	20.68786933	2.84E-05	0.0001496
**ENSG00000197150**	ABCB8	-0.845102052	4.67941035	20.77265784	2.85E-05	0.0001498
**ENSG00000147576**	ADHFE1	-0.734337473	4.68249299	20.67748039	2.85E-05	0.0001500
**ENSG00000143167**	GPA33	-0.889503222	4.063382053	20.65255771	2.88E-05	0.0001514
**ENSG00000167553**	TUBA1C	0.735077919	4.466329818	20.64436765	2.89E-05	0.0001518
**ENSG00000278962**		0.90217869	3.773191364	20.63145642	2.9E-05	0.0001525
**ENSG00000174574**	AKIRIN1	0.348555255	6.678210113	20.62872784	2.91E-05	0.0001525
**ENSG00000136997**	MYC	-0.381817203	7.238283032	20.61883969	2.92E-05	0.0001530
**ENSG00000109133**	TMEM33	0.399676467	6.930970655	20.61394527	2.92E-05	0.0001533
**ENSG00000218175**		0.941836965	4.253302583	20.80248323	2.94E-05	0.0001543
**ENSG00000196715**	VKORC1L1	0.585626089	4.993331535	20.58790123	2.95E-05	0.0001546
**ENSG00000198189**	HSD17B11	0.399831634	6.147299947	20.58661029	2.95E-05	0.0001546

**ENSG00000166387**	PPFIBP2	-0.780367744	4.320058811	20.56446483	2.98E-05	0.0001559
**ENSG00000172531**	PPP1CA	0.480432592	5.86666743	20.55766632	2.99E-05	0.0001562
**ENSG00000214013**	GANC	-0.66234281	5.531909467	20.76510675	2.99E-05	0.0001563
**ENSG00000172667**	ZMAT3	0.425078682	6.473255968	20.55026266	2.99E-05	0.0001565
**ENSG00000164880**	INTS1	-0.528567685	6.123211509	20.56246657	3.03E-05	0.0001582
**ENSG00000135205**	CCDC146	-0.720590442	4.552474105	20.51394541	3.04E-05	0.0001585
**ENSG00000064687**	ABCA7	-1.018918341	6.108044331	22.60978684	3.04E-05	0.0001586
**ENSG00000123374**	CDK2	-0.974700163	3.731204748	20.50628978	3.05E-05	0.0001588
**ENSG00000056586**	RC3H2	0.303084534	7.527902458	20.5024627	3.05E-05	0.0001590
**ENSG00000115310**	RTN4	0.330020226	6.650959462	20.4889109	3.07E-05	0.0001597
**ENSG00000132436**	FIGNL1	-0.664032291	4.639270716	20.48784178	3.07E-05	0.0001597
**ENSG00000132824**	SERINC3	0.297550451	7.497858137	20.46073013	3.1E-05	0.0001613
**ENSG00000130684**	ZNF337	-0.544291188	5.201086782	20.45133759	3.11E-05	0.0001618
**ENSG00000204186**	ZDBF2	-0.604035822	5.289564146	20.44963106	3.11E-05	0.0001619
**ENSG00000178605**	GTPBP6	-0.73795898	4.999163204	20.54218447	3.12E-05	0.0001622
**ENSG00000100075**	SLC25A1	0.988901419	3.696948996	20.4401071	3.12E-05	0.0001623
**ENSG00000228863**		0.465965889	7.496284852	21.21663885	3.14E-05	0.0001632
**ENSG00000103091**	WDR59	-0.672868731	5.618855419	20.81907405	3.14E-05	0.0001632
**ENSG00000116560**	SFPQ	-0.283861359	8.789248719	20.41872502	3.15E-05	0.0001634
**ENSG00000179912**	R3HDM2	-0.346845114	6.985216843	20.40956777	3.16E-05	0.0001639
**ENSG00000156500**	FAM122C	-0.901409196	3.827053586	20.39088897	3.18E-05	0.0001650
**ENSG00000222041**	CYTOR	0.79984615	4.071217186	20.38898738	3.19E-05	0.0001650
**ENSG00000137770**	CTDSPL2	0.3457293	7.077270183	20.38120931	3.2E-05	0.0001654
**ENSG00000146802**	TMEM168	-0.480905975	6.012973242	20.37849476	3.2E-05	0.0001655
**ENSG00000261460**		-0.782934486	4.034839477	20.37298301	3.21E-05	0.0001658
**ENSG00000165494**	PCF11	-0.277724246	8.114644862	20.36737134	3.21E-05	0.0001661
**ENSG00000082146**	STRADB	-0.715393614	4.519753695	20.36121408	3.22E-05	0.0001664
**ENSG00000171163**	ZNF692	-1.159768383	4.791696861	21.86103752	3.23E-05	0.0001667
**ENSG00000197457**	STMN3	-0.747578255	5.040538787	20.50482651	3.24E-05	0.0001673
**ENSG00000128294**	TPST2	0.5863856	5.001277212	20.33743686	3.25E-05	0.0001677
**ENSG00000206527**	HACD2	0.493111083	5.86544479	20.33504893	3.25E-05	0.0001678
**ENSG00000101246**	ARFRP1	-0.670298172	4.488956918	20.31482183	3.28E-05	0.0001690
**ENSG00000106477**	CEP41	-0.948936511	3.770121741	20.30017236	3.3E-05	0.0001699
**ENSG00000148429**	USP6NL	-1.024645804	3.70763644	20.28512992	3.32E-05	0.0001708
**ENSG00000163945**	UVSSA	-0.712178125	6.101795488	21.58984423	3.32E-05	0.0001708
**ENSG00000071626**	DAZAP1	-0.511113173	5.949134387	20.28022296	3.32E-05	0.0001709
**ENSG00000240409**		1.111198494	4.171366405	20.90722245	3.33E-05	0.0001711

**ENSG00000077420**	APBB1IP	0.290951861	7.486731198	20.26292335	3.34E-05	0.0001719
**ENSG00000143252**	SDHC	-0.485798135	5.474856292	20.26214684	3.35E-05	0.0001719
**ENSG00000178498**	DTX3	-1.043793868	3.489977647	20.25826696	3.35E-05	0.0001720
**ENSG00000167112**	TRUB2	-0.768494164	4.320582794	20.25734534	3.35E-05	0.0001720
**ENSG00000204622**	HLA-J	0.672662178	4.923815121	20.2510454	3.36E-05	0.0001723
**ENSG00000151498**	ACAD8	-0.659209061	4.945802565	20.24677518	3.37E-05	0.0001725
**ENSG00000169375**	SIN3A	0.273745543	7.616200719	20.23643552	3.38E-05	0.0001731
**ENSG00000144909**	OSBPL11	0.641844899	4.91254287	20.23623358	3.38E-05	0.0001731
**ENSG00000282826**	FRG1CP	-0.804193033	4.333253244	20.21403307	3.41E-05	0.0001745
**ENSG00000119638**	NEK9	-0.342652186	6.64977287	20.19733121	3.43E-05	0.0001755
**ENSG00000134698**	AGO4	0.49235319	6.113092867	20.18179723	3.45E-05	0.0001765
**ENSG00000172673**	THEMIS	0.320298735	7.415242808	20.17718195	3.46E-05	0.0001767
**ENSG00000270024**	C8orf44-SGK3	0.474939442	5.113504025	20.16429174	3.47E-05	0.0001775
**ENSG00000130810**	PPAN	-0.637844432	4.808362094	20.1430963	3.5E-05	0.0001788
**ENSG00000204305**	AGER	-0.996197034	3.935077921	20.14272488	3.5E-05	0.0001788
**ENSG00000214900**	LINC01588	-0.822911364	4.826107033	20.37287713	3.51E-05	0.0001790
**ENSG00000217130**		0.905810763	3.96721277	20.13577161	3.51E-05	0.0001791
**ENSG00000205352**	PRR13	0.41138072	5.944561672	20.13026908	3.52E-05	0.0001794
**ENSG00000058453**	CROCC	-0.710448795	4.689194319	20.12416972	3.53E-05	0.0001798
**ENSG00000100731**	PCNX1	0.273200893	8.896370062	20.12219713	3.53E-05	0.0001798
**ENSG00000100101**	NOL12	-0.845249487	4.262681778	20.12057267	3.53E-05	0.0001798
**ENSG00000204165**	CXorf65	-0.907667655	3.88486509	20.10314612	3.56E-05	0.0001810
**ENSG00000213066**	FGFR1OP	-0.632754745	5.347081142	20.08515141	3.58E-05	0.0001822
**ENSG00000023041**	ZDHHC6	-0.468219716	5.933083252	20.07339401	3.6E-05	0.0001829
**ENSG00000197114**	ZGPAT	-0.717706363	4.580919761	20.05446218	3.63E-05	0.0001842
**ENSG00000116815**	CD58	0.846424489	4.579965545	20.20111283	3.64E-05	0.0001848
**ENSG00000136147**	PHF11	-0.492431081	6.528970684	20.2293224	3.65E-05	0.0001852
**ENSG00000101474**	APMAP	0.624157599	5.125451964	20.03043141	3.66E-05	0.0001856
**ENSG00000178852**	Efcab13	-0.731109393	4.742667926	20.01402334	3.68E-05	0.0001867
**ENSG00000115307**	AUP1	-0.611920164	5.7382375	20.22507497	3.73E-05	0.0001888
**ENSG00000181826**	RELL1	0.756051993	4.117488401	19.93416449	3.8E-05	0.0001924
**ENSG00000181847**	Tigit	0.651403692	5.651208654	20.28409112	3.81E-05	0.0001927
**ENSG00000265241**	RBM8A	0.367857132	6.483647143	19.90435205	3.84E-05	0.0001944
**ENSG00000116954**	RRAGC	0.576196611	4.950834804	19.88390453	3.87E-05	0.0001959
**ENSG00000204406**	mbd5	-0.432104871	6.606950787	19.87765233	3.88E-05	0.0001963
**ENSG00000177272**	KCNA3	0.333899386	7.195770984	19.87537563	3.89E-05	0.0001963
**ENSG00000110330**	BIRC2	-0.359561612	6.706499589	19.87431361	3.89E-05	0.0001963

**ENSG00000252010**	SCARNA5	1.660731372	6.972975554	21.91509888	3.89E-05	0.0001964
**ENSG00000166272**	WBP1L	0.442582411	5.626533635	19.87149415	3.89E-05	0.0001964
**ENSG00000110906**	KCTD10	0.536652147	5.334617324	19.86181579	3.91E-05	0.0001969
**ENSG00000110172**	CHORDC1	-0.358194564	7.099891181	19.86128058	3.91E-05	0.0001969
**ENSG00000120008**	WDR11	-0.495924543	6.182912042	19.8610626	3.91E-05	0.0001969
**ENSG00000105483**	CARD8	-0.332448558	7.99688565	19.85237394	3.92E-05	0.0001974
**ENSG00000178904**	DPY19L3	-0.761944574	4.566744231	19.85008354	3.92E-05	0.0001974
**ENSG00000137337**	MDC 1.00	-0.408913054	5.827318525	19.84992925	3.92E-05	0.0001974
**ENSG00000214182**		0.970756482	4.689521577	20.83000578	3.93E-05	0.0001974
**ENSG00000138658**	ZGRF1	-0.802368086	4.20159771	19.84824802	3.93E-05	0.0001974
**ENSG00000198951**	NAGA	0.658317289	4.288133349	19.81534863	3.98E-05	0.0001998
**ENSG00000204560**	DHX16	-0.558870872	5.337294398	19.8148768	3.98E-05	0.0001998
**ENSG00000124532**	MRS2	-0.567657778	5.129821272	19.81284988	3.98E-05	0.0001998
**ENSG00000095059**	DHPS	-0.689641326	4.807481688	19.80418687	3.99E-05	0.0002004
**ENSG00000021574**	SPAST	0.475563412	5.698778156	19.76973172	4.05E-05	0.0002030
**ENSG00000275052**	PPP4R3B	0.253637795	8.158363216	19.75854801	4.07E-05	0.0002038
**ENSG00000074695**	LMAN1	0.368759868	6.780959555	19.75692688	4.07E-05	0.0002038
**ENSG00000136527**	TRA2B	-0.323907082	8.420564501	19.72830261	4.11E-05	0.0002060
**ENSG00000188687**	slc4a5	-0.708245963	4.716865105	19.72055146	4.13E-05	0.0002065
**ENSG00000214021**	TTLL3	-0.988183023	5.68820485	21.51315188	4.13E-05	0.0002066
**ENSG00000047410**	TPR	-0.313148359	8.97641822	19.71774628	4.13E-05	0.0002066
**ENSG00000162139**	NEU3	-0.780092938	4.305239961	19.69652908	4.17E-05	0.0002082
**ENSG00000180098**	TRNAU1AP	-0.734042124	4.515052917	19.69403503	4.17E-05	0.0002083
**ENSG00000270181**	BIVM-ERCC5	-0.289838013	7.522499062	19.68764391	4.18E-05	0.0002087
**ENSG00000256349**	BBS1	-0.879397011	4.244603516	19.67604708	4.2E-05	0.0002095
**ENSG00000165121**		-0.80655017	4.115362482	19.67579267	4.2E-05	0.0002095
**ENSG00000116127**	ALMS1	0.327836783	7.482964645	19.65101404	4.24E-05	0.0002114
**ENSG00000116199**	FAM20B	0.506360693	5.461251257	19.62925692	4.28E-05	0.0002131
**ENSG00000212283**	Snord89	1.45013595	6.581843437	21.57150023	4.35E-05	0.0002167
**ENSG00000119414**	PPP6C	0.301835939	7.070198212	19.5716369	4.37E-05	0.0002176
**ENSG00000266947**		-0.858333508	3.962089041	19.57152111	4.37E-05	0.0002176
**ENSG00000204152**	TIMM23B	-0.920417602	3.933513612	19.56268344	4.39E-05	0.0002183
**ENSG00000257151**		-0.959517257	4.301315053	19.61453689	4.43E-05	0.0002201
**ENSG00000070367**	EXOC5	0.365442589	6.765466495	19.53036388	4.44E-05	0.0002208
**ENSG00000119514**	GALNT12	-0.884503582	3.532552076	19.53015268	4.44E-05	0.0002208
**ENSG00000156017**	CARNMT1	0.45401382	5.564716289	19.52617286	4.45E-05	0.0002210
**ENSG00000117020**	AKT3	0.331576632	7.339987913	19.51720719	4.47E-05	0.0002217

**ENSG00000133816**	MICAL2	0.61826425	5.284966692	19.51504677	4.47E-05	0.0002217
**ENSG00000116690**	PRG4	-0.609816426	4.995978142	19.49288975	4.51E-05	0.0002235
**ENSG00000134516**	DOCK2	0.253187113	8.171844608	19.49240678	4.51E-05	0.0002235
**ENSG00000080503**	SMARCA2	0.260654552	8.749693845	19.48570931	4.52E-05	0.0002240
**ENSG00000160588**	MPZL3	0.578114183	5.137924724	19.47587612	4.54E-05	0.0002247
**ENSG00000144554**	FANCD2	-0.685860421	5.183051929	19.58417036	4.54E-05	0.0002247
**ENSG00000236088**	COX10-AS1	-0.676686789	4.641021567	19.44318129	4.6E-05	0.0002274
**ENSG00000160124**	CCDC58	-1.051558284	3.595899916	19.42303011	4.63E-05	0.0002291
**ENSG00000108854**	SMURF2	0.418922626	6.350276521	19.39929894	4.68E-05	0.0002311
**ENSG00000181827**	RFX7	0.317257803	7.168842679	19.37544416	4.72E-05	0.0002332
**ENSG00000001497**	LAS1L	-0.495273362	5.30100121	19.37225396	4.73E-05	0.0002334
**ENSG00000148343**	MIGA2	-1.102631987	4.317658587	20.00481031	4.74E-05	0.0002339
**ENSG00000279069**		-0.745998225	4.261877582	19.33523401	4.8E-05	0.0002365
**ENSG00000227694**		1.188357378	4.235588991	20.53310247	4.8E-05	0.0002368
**ENSG00000120963**	ZNF706	-0.443188999	6.095951695	19.3304627	4.81E-05	0.0002368
**ENSG00000099942**	CRKL	0.34811014	6.99516584	19.31961269	4.83E-05	0.0002377
**ENSG00000267449**		1.184287775	4.045653493	20.29027319	4.84E-05	0.0002382
**ENSG00000172845**	SP3	0.268554779	7.882554853	19.30674702	4.85E-05	0.0002386
**ENSG00000250479**	CHCHD10	0.878915759	3.571985068	19.29321134	4.88E-05	0.0002398
**ENSG00000123240**	OPTN	0.308238677	7.324560891	19.2922142	4.88E-05	0.0002398
**ENSG00000175662**	TOM1L2	-0.630597767	5.2187705	19.28389484	4.89E-05	0.0002404
**ENSG00000187726**	DNAJB13	-0.908273758	3.837113717	19.28185056	4.9E-05	0.0002405
**ENSG00000111412**	c12orf49	0.449559356	5.45113341	19.26636778	4.93E-05	0.0002419
**ENSG00000146828**	SLC12A9	-0.82276545	5.000672689	19.7111943	4.96E-05	0.0002432
**ENSG00000173402**	DAG1	0.608774645	4.57509748	19.23781332	4.98E-05	0.0002444
**ENSG00000185236**	RAB11B	-0.457072601	5.341282532	19.23408918	4.99E-05	0.0002446
**ENSG00000108091**	CCDC6	0.409465924	6.257929303	19.21079314	5.04E-05	0.0002466
**ENSG00000119541**	VPS4B	0.334183614	6.589740615	19.21012401	5.04E-05	0.0002466
**ENSG00000204149**	AGAP6	-1.247689971	4.345457526	20.32414062	5.04E-05	0.0002466
**ENSG00000041802**	LSG1	-0.521910587	5.815995958	19.19521774	5.07E-05	0.0002479
**ENSG00000055163**	cyfip2	0.264347861	9.342069292	19.18348044	5.09E-05	0.0002489
**ENSG00000162526**	TSSK3	-1.047375261	3.791410478	19.17809108	5.1E-05	0.0002493
**ENSG00000084073**	ZMPSTE24	0.555235455	5.096071006	19.17517392	5.11E-05	0.0002494
**ENSG00000099899**	TRMT2A	-0.737562329	4.458393267	19.17509397	5.11E-05	0.0002494
**ENSG00000135090**	TAOK3	0.31075915	7.509802118	19.16014082	5.14E-05	0.0002507
**ENSG00000202538**	RNU4-2	1.604859802	8.822308234	21.05961234	5.15E-05	0.0002511
**ENSG00000130023**	ERMARD	-0.72755537	4.833226413	19.13697576	5.18E-05	0.0002528

**ENSG00000117632**	MIR3917	-0.660470243	4.473662819	19.13524286	5.19E-05	0.0002529
**ENSG00000183426**	NPIPA1	-0.961219774	3.816911496	19.11933234	5.22E-05	0.0002543
**ENSG00000211753**		0.956906753	3.751588677	19.1092507	5.24E-05	0.0002552
**ENSG00000105053**	VRK3	-0.477306474	5.426235335	19.08344226	5.29E-05	0.0002577
**ENSG00000131828**	PDHA1	-0.460307743	5.442474965	19.07845651	5.3E-05	0.0002581
**ENSG00000196440**	ARMCX4	-1.052818049	3.957161751	19.203806	5.32E-05	0.0002589
**ENSG00000150961**	SEC24D	0.492017055	5.349885195	19.0562076	5.35E-05	0.0002601
**ENSG00000258790**	KIAA0391	0.367892466	6.236114402	19.03839328	5.39E-05	0.0002618
**ENSG00000284431**		-0.415831962	6.311194618	19.0371445	5.39E-05	0.0002618
**ENSG00000149925**	ALDOA	0.412250494	7.350445256	19.30148039	5.4E-05	0.0002619
**ENSG00000053900**	ANAPC4	-0.617426482	6.241934172	19.92570796	5.41E-05	0.0002624
**ENSG00000125868**	DSTN	0.453311492	5.790867677	19.00659079	5.46E-05	0.0002646
**ENSG00000078319**		-0.769572142	4.20422073	18.99217915	5.49E-05	0.0002660
**ENSG00000114735**	HEMK1	-0.627698383	5.909746103	19.62272773	5.53E-05	0.0002681
**ENSG00000213965**	NUDT19	0.602411699	4.450090462	18.96963745	5.54E-05	0.0002681
**ENSG00000140577**	CRTC3	-0.325601183	6.883486673	18.96125579	5.55E-05	0.0002688
**ENSG00000170791**	CHCHD7	-0.439015563	5.593598598	18.96034926	5.56E-05	0.0002688
**ENSG00000168010**	atg16l2	-0.754047123	6.29711069	20.50028374	5.58E-05	0.0002700
**ENSG00000234287**		1.093154141	4.465629376	19.99310641	5.75E-05	0.0002778
**ENSG00000237914**	SIRPG-AS1	-0.974264273	3.930623534	18.86700708	5.77E-05	0.0002785
**ENSG00000102158**	MAGT1	0.40390582	6.124038477	18.85968601	5.78E-05	0.0002792
**ENSG00000122566**	HNRNPA2B1	-0.301187798	9.907385933	18.84257615	5.82E-05	0.0002810
**ENSG00000196547**	MAN2A2	-0.782444604	6.127625766	20.34648751	5.83E-05	0.0002812
**ENSG00000239002**	Scarna10	1.540844362	6.614003127	20.6630963	5.87E-05	0.0002831
**ENSG00000109606**	DHX15	-0.28120643	7.629419381	18.81247555	5.89E-05	0.0002839
**ENSG00000198586**	TLK1	0.298684312	7.92078654	18.80974864	5.9E-05	0.0002841
**ENSG00000180628**	PCGF5	0.335346802	7.744294764	18.80735953	5.9E-05	0.0002842
**ENSG00000276232**		1.540865627	6.613945174	20.63636181	5.92E-05	0.0002851
**ENSG00000239264**	TXNDC5	0.742081115	4.378453666	18.7969608	5.93E-05	0.0002851
**ENSG00000071894**	MIR939	-0.654789427	5.773037798	19.43748664	5.93E-05	0.0002851
**ENSG00000182473**	EXOC7	-0.414499928	6.426201996	18.78345705	5.96E-05	0.0002864
**ENSG00000132432**	SEC61G	0.763057891	4.483571603	18.77837064	5.97E-05	0.0002869
**ENSG00000247287**		-0.852733132	4.666821603	19.01141834	5.99E-05	0.0002876
**ENSG00000142657**	PGD	0.687572944	4.288059456	18.76790248	5.99E-05	0.0002878
**ENSG00000215417**	MIR17	-0.790529662	4.505929373	18.76574938	6E-05	0.0002879
**ENSG00000185619**	PCGF3	-0.79592028	6.447022772	20.43625263	6.02E-05	0.0002885
**ENSG00000164902**	PHAX	0.517147257	5.574092274	18.74600976	6.05E-05	0.0002899

**ENSG00000111670**	GNPTAB	0.350463694	6.943244366	18.69789489	6.16E-05	0.0002953
**ENSG00000225963**		-0.706857838	5.000802027	18.72660905	6.18E-05	0.0002960
**ENSG00000130827**	PLXNA3	-0.74386782	5.946827029	19.89905048	6.19E-05	0.0002962
**ENSG00000264235**	LOC104968399	0.476643895	6.050831418	18.68428262	6.2E-05	0.0002963
**ENSG00000077684**	JADE1	0.362181108	6.759444419	18.68324435	6.2E-05	0.0002963
**ENSG00000142864**	SERBP1	0.273625879	8.114360968	18.68319875	6.2E-05	0.0002963
**ENSG00000112309**	B3GAT2	0.485850807	5.445297084	18.67186965	6.23E-05	0.0002975
**ENSG00000135316**	SYNCRIP	0.273174305	7.898041002	18.65982119	6.26E-05	0.0002988
**ENSG00000089351**	GRAMD1A	-0.476100756	6.405604139	18.78534524	6.3E-05	0.0003006
**ENSG00000111300**	NAA25	-0.384051297	6.339142714	18.64004915	6.31E-05	0.0003009
**ENSG00000125107**	CNOT1	0.234549584	8.988067871	18.637252	6.31E-05	0.0003011
**ENSG00000008282**	SYPL1	0.483372364	5.85098583	18.63348229	6.32E-05	0.0003014
**ENSG00000223865**	HLA-DPB1	0.799462073	4.629013958	18.63202	6.33E-05	0.0003015
**ENSG00000119844**	AFTPH	0.316418286	6.594789576	18.6182296	6.36E-05	0.0003030
**ENSG00000185359**	HGS	-0.732724522	5.501633746	19.29971123	6.4E-05	0.0003048
**ENSG00000142541**	RPL13A	0.347621309	10.71129364	18.58479338	6.45E-05	0.0003067
**ENSG00000061938**	TNK2	-0.737624059	5.497973545	19.3063451	6.45E-05	0.0003068
**ENSG00000143799**	PARP1	0.329950611	6.993356328	18.56708443	6.49E-05	0.0003086
**ENSG00000148334**	PTGES2	-0.95157201	3.90225068	18.5560292	6.52E-05	0.0003098
**ENSG00000100038**	TOP3B	-0.916758575	4.830360405	19.21216038	6.54E-05	0.0003106
**ENSG00000135655**	MIR6125	-0.362278996	8.411532453	18.68449586	6.55E-05	0.0003108
**ENSG00000113558**	SKP1	-0.307603901	8.40097501	18.53848911	6.56E-05	0.0003115
**ENSG00000154016**	GRAP	-0.581661944	4.663201521	18.53351723	6.58E-05	0.0003118
**ENSG00000279332**		0.745214218	4.236782127	18.53322431	6.58E-05	0.0003118
**ENSG00000139160**	ETFBKMT	-0.823555644	3.908840741	18.53258962	6.58E-05	0.0003118
**ENSG00000204642**	HLA-F	-0.306072956	7.340898249	18.52151347	6.61E-05	0.0003131
**ENSG00000092531**	SNAP23	-0.395858989	5.9811196	18.51466658	6.63E-05	0.0003138
**ENSG00000251143**	LOC100128494	-0.329816135	7.401854041	18.50581128	6.65E-05	0.0003147
**ENSG00000108651**	UTP6	-0.430436841	5.483342887	18.50191884	6.66E-05	0.0003150
**ENSG00000281039**		-1.04189153	3.408811814	18.50178447	6.66E-05	0.0003150
**ENSG00000118246**	FASTKD2	-0.480781295	5.51796687	18.47904009	6.72E-05	0.0003177
**ENSG00000234782**		1.023721945	4.470926148	19.4133528	6.74E-05	0.0003183
**ENSG00000185187**	SIGIRR	-0.692726254	5.780119094	19.31360319	6.75E-05	0.0003186
**ENSG00000082898**	XPO1	-0.269087314	8.202802187	18.46248516	6.77E-05	0.0003192
**ENSG00000186088**	GSAP	-0.694047362	5.234752059	18.72722233	6.77E-05	0.0003192
**ENSG00000075785**	RAB7A	0.369198411	7.225631409	18.46102003	6.77E-05	0.0003192
**ENSG00000160185**	UBASH3A	-0.38506494	6.673452487	18.45967312	6.77E-05	0.0003193

**ENSG00000211799**		0.820798247	3.535459349	18.45497254	6.79E-05	0.0003197
**ENSG00000114796**	KLHL24	0.345159064	7.263270087	18.43914741	6.83E-05	0.0003216
**ENSG00000175550**	DRAP1	0.605331516	4.468112972	18.43033359	6.85E-05	0.0003226
**ENSG00000198105**	ZNF248	-0.616499913	4.752651637	18.41848825	6.88E-05	0.0003239
**ENSG00000136490**	LIMD2	-0.358415854	6.878012057	18.40607691	6.92E-05	0.0003254
**ENSG00000147854**	UHRF2	-0.362658567	7.21328441	18.40303704	6.93E-05	0.0003256
**ENSG00000120686**	UFM1	-0.371461703	6.693900942	18.39254106	6.96E-05	0.0003268
**ENSG00000124575**	Hist1h1d	0.724341952	7.850163319	20.11387635	6.99E-05	0.0003282
**ENSG00000143486**	EIF2D	-0.422512384	5.747861546	18.37194325	7.01E-05	0.0003292
**ENSG00000120690**	ELF1	0.334730942	8.285587071	18.3652596	7.03E-05	0.0003300
**ENSG00000186481**		-0.970135894	4.257058476	18.54449791	7.03E-05	0.0003300
**ENSG00000233937**		-0.493302475	5.603450873	18.35827812	7.05E-05	0.0003306
**ENSG00000120265**	PCMT1	0.513205753	5.214264653	18.34371275	7.09E-05	0.0003324
**ENSG00000204604**	ZNF468	0.518337602	5.134521045	18.33057789	7.13E-05	0.0003339
**ENSG00000177042**	TMEM80	-0.798412761	3.678597643	18.31824282	7.16E-05	0.0003354
**ENSG00000143458**	GABPB2	-0.413748993	6.14146066	18.31632084	7.17E-05	0.0003355
**ENSG00000200795**	RNU4-1	1.757270559	7.984365089	20.04548628	7.22E-05	0.0003376
**ENSG00000130714**	POMT1	-0.894938107	4.887651043	19.03447542	7.23E-05	0.0003379
**ENSG00000102409**	BEX4	-0.776644111	4.06972962	18.29518541	7.23E-05	0.0003379
**ENSG00000085185**	BCORL1	0.674028663	4.568795045	18.29110623	7.24E-05	0.0003383
**ENSG00000242288**		-0.861170557	4.280187457	18.28509523	7.26E-05	0.0003390
**ENSG00000108953**	YWHAE	0.377329264	6.650046991	18.27857325	7.28E-05	0.0003397
**ENSG00000111786**	SRSF9	-0.353002551	6.240876596	18.27540266	7.29E-05	0.0003400
**ENSG00000136935**	GOLGA1	-0.501838107	5.746729096	18.27322603	7.29E-05	0.0003401
**ENSG00000130592**	MIR7847	0.331896272	6.662244426	18.25176347	7.36E-05	0.0003429
**ENSG00000110768**	GTF2H1	-0.412730107	5.728035711	18.23882384	7.39E-05	0.0003445
**ENSG00000245849**	RAD51-AS1	-0.788119462	4.37445231	18.22715977	7.43E-05	0.0003460
**ENSG00000114841**	DNAH1	-0.779694809	7.035946038	19.90068201	7.44E-05	0.0003463
**ENSG00000246596**		-0.714813154	4.236664062	18.21847453	7.45E-05	0.0003468
**ENSG00000089682**	RBM41	-0.595063738	5.075635612	18.20445824	7.5E-05	0.0003486
**ENSG00000196323**	ZBTB44	0.248486916	7.715731497	18.19113416	7.54E-05	0.0003503
**ENSG00000143224**	PPOX	-0.839049405	4.00920991	18.18839895	7.54E-05	0.0003506
**ENSG00000218510**	LINC00339	-0.789809951	3.869925719	18.17715043	7.58E-05	0.0003520
**ENSG00000267633**		-1.059925783	4.074139142	18.54709182	7.58E-05	0.0003520
**ENSG00000067900**	ROCK1	0.345566291	8.296053195	18.19331346	7.59E-05	0.0003523
**ENSG00000277072**		-0.793221089	4.111463918	18.16806713	7.61E-05	0.0003527
**ENSG00000186130**	ZBTB6	0.510061974	5.519899598	18.16783687	7.61E-05	0.0003527

**ENSG00000168374**	ARF4	0.453775851	5.598012852	18.16660731	7.61E-05	0.0003527
**ENSG00000121022**	COPS5	-0.467537855	5.828542674	18.16550619	7.61E-05	0.0003527
**ENSG00000228409**		-0.808864624	4.43246149	18.15781232	7.64E-05	0.0003536
**ENSG00000130584**	ZBTB46	-0.944511722	3.989865821	18.15313773	7.65E-05	0.0003538
**ENSG00000242588**		-0.491917533	5.626649836	18.1529176	7.65E-05	0.0003538
**ENSG00000166233**	ARIH1	0.269126984	7.575131979	18.15271438	7.65E-05	0.0003538
**ENSG00000153933**	DGKE	-0.420694452	6.338670248	18.13916309	7.69E-05	0.0003556
**ENSG00000138279**	ANXA7	0.342750827	6.389931401	18.12573482	7.73E-05	0.0003574
**ENSG00000240489**		0.882172733	3.650176073	18.10777137	7.79E-05	0.0003598
**ENSG00000156030**	ELMSAN1	0.318154496	6.874093769	18.10205512	7.81E-05	0.0003604
**ENSG00000117877**	CD3EAP	-0.806187491	3.845140947	18.10145427	7.81E-05	0.0003604
**ENSG00000173875**	ZNF791	0.404444623	6.358542524	18.09935566	7.82E-05	0.0003605
**ENSG00000128000**	ZNF780B	-0.445279302	6.238117869	18.0940263	7.83E-05	0.0003611
**ENSG00000149499**	EML3	-0.673184438	5.219019455	18.20583623	7.89E-05	0.0003635
**ENSG00000141337**	ARSG	-0.716017329	4.707268784	18.07135017	7.9E-05	0.0003641
**ENSG00000198026**	ZNF335	-0.486041021	6.215198105	18.16848945	7.92E-05	0.0003647
**ENSG00000153094**	BCL2L11	0.458997508	5.950410098	18.06410567	7.93E-05	0.0003648
**ENSG00000111860**	CEP85L	-0.335774116	7.496741653	18.06141034	7.94E-05	0.0003650
**ENSG00000074935**	TUBE1	-0.525234139	5.37120953	18.05561951	7.95E-05	0.0003657
**ENSG00000244313**		0.966818284	4.808195614	19.12053353	8.01E-05	0.0003681
**ENSG00000157181**	C1orf27	-0.578246674	5.156695495	18.02831819	8.04E-05	0.0003693
**ENSG00000226752**		-0.444472852	5.971369344	18.02810634	8.04E-05	0.0003693
**ENSG00000138376**	BARD1	0.799712014	4.56751226	18.15742091	8.06E-05	0.0003700
**ENSG00000155304**	HSPA13	0.568971277	5.451304025	17.99301328	8.16E-05	0.0003742
**ENSG00000254893**		0.93983649	3.516514412	17.97485824	8.21E-05	0.0003767
**ENSG00000224660**	SH3BP5-AS1	-0.354810602	7.016070965	17.9740741	8.22E-05	0.0003767
**ENSG00000161551**	ZNF577	-0.698793207	4.468756017	17.97216476	8.22E-05	0.0003768
**ENSG00000167325**	RRM1	0.459299012	5.748551508	17.94880002	8.3E-05	0.0003802
**ENSG00000172780**	RAB43	-0.876528451	3.719233917	17.92951662	8.37E-05	0.0003829
**ENSG00000118620**	ZNF430	0.359274087	6.506407892	17.9195845	8.4E-05	0.0003843
**ENSG00000145287**	PLAC8	-0.371254694	7.803865618	18.1104088	8.4E-05	0.0003843
**ENSG00000153395**	LPCAT1	0.525185159	5.620496191	17.91730926	8.41E-05	0.0003843
**ENSG00000069493**	CLEC2D	-0.276555699	8.986726632	17.91587441	8.41E-05	0.0003844
**ENSG00000114626**	ABTB1	-0.640522567	5.333532957	18.02758496	8.46E-05	0.0003866
**ENSG00000166986**	MIR6758	-0.444019401	6.224776194	17.88445058	8.52E-05	0.0003889
**ENSG00000171291**	ZNF439	-0.753639198	4.397079364	17.87560037	8.55E-05	0.0003901
**ENSG00000173890**	gpr160	-0.891364354	4.19997329	17.87018516	8.57E-05	0.0003908

**ENSG00000184860**	SDR42E1	-0.779045525	4.372901865	17.85995461	8.6E-05	0.0003921
**ENSG00000272742**		0.570091478	5.833237115	18.25815953	8.61E-05	0.0003921
**ENSG00000021300**	PLEKHB1	-0.608016494	4.639532956	17.85718859	8.61E-05	0.0003921
**ENSG00000117036**	ETV3	0.471443215	5.754651013	17.85659976	8.61E-05	0.0003921
**ENSG00000188647**	PTAR1	-0.305318157	7.589554638	17.85629955	8.61E-05	0.0003921
**ENSG00000146414**	SHPRH	-0.390294297	7.132334861	17.8812157	8.66E-05	0.0003938
**ENSG00000143013**	LMO4	0.764519116	4.373048355	17.83568754	8.69E-05	0.0003950
**ENSG00000087086**	FTL	0.460494068	8.337422504	18.81887408	8.69E-05	0.0003950
**ENSG00000124203**	ZNF831	0.347572864	6.75493366	17.82888014	8.71E-05	0.0003957
**ENSG00000133111**	rfxap	-0.772766682	3.810402262	17.81559187	8.76E-05	0.0003977
**ENSG00000063854**	HAGH	-0.752271954	4.214244597	17.79987787	8.81E-05	0.0004000
**ENSG00000184992**	BRI3BP	0.659679453	4.665428855	17.79109827	8.84E-05	0.0004012
**ENSG00000217027**		1.084997073	3.779782544	18.24942959	8.89E-05	0.0004033
**ENSG00000228474**	OST4	0.531733717	5.820537163	17.90496553	8.91E-05	0.0004039
**ENSG00000115604**	IL18R1	0.75904809	4.869229955	18.04525029	8.96E-05	0.0004062
**ENSG00000136560**	TANK	0.36359457	6.514058014	17.75186786	8.98E-05	0.0004069
**ENSG00000133112**	TPT1	0.499829165	11.55278596	18.64712879	9.1E-05	0.0004122
**ENSG00000149016**	TUT1	-0.880797446	3.578361748	17.71512773	9.12E-05	0.0004126
**ENSG00000213625**	LEPROT	0.440431383	5.617377421	17.71263967	9.12E-05	0.0004128
**ENSG00000105810**	CDK6	0.468009589	7.375252265	18.537677	9.15E-05	0.0004140
**ENSG00000113649**	TCERG1	-0.359793219	7.144615492	17.69555622	9.19E-05	0.0004153
**ENSG00000163138**	PACRGL	-0.978613899	3.695233765	17.69449981	9.19E-05	0.0004153
**ENSG00000184207**	PGP	-0.889927074	3.520504263	17.69319398	9.2E-05	0.0004153
**ENSG00000174282**	ZBTB4	0.352955661	6.47379827	17.68070528	9.24E-05	0.0004172
**ENSG00000127415**	IDUA	-1.070389395	3.607154971	17.67511552	9.26E-05	0.0004180
**ENSG00000158615**	PPP1R15B	0.29088964	7.291193117	17.66625986	9.3E-05	0.0004193
**ENSG00000234222**	LOC105371260	0.380571565	5.460314347	17.65544726	9.34E-05	0.0004210
**ENSG00000112282**	MED23	-0.367376099	6.88807528	17.65255912	9.35E-05	0.0004213
**ENSG00000017797**	RALBP1	0.399942773	5.923457759	17.64233463	9.39E-05	0.0004226
**ENSG00000082153**	BZW1	0.275501694	6.949898538	17.64225085	9.39E-05	0.0004226
**ENSG00000261611**		-0.840521325	3.999465582	17.63425726	9.42E-05	0.0004238
**ENSG00000224546**		0.809833054	3.49090756	17.62981563	9.43E-05	0.0004244
**ENSG00000230124**	LHX4-AS1	-0.544425565	5.253036082	17.61209869	9.5E-05	0.0004272
**ENSG00000138735**	PDE5A	-0.706945452	5.141655977	17.79661337	9.56E-05	0.0004298
**ENSG00000147996**	CBWD5	-0.744282396	4.269639578	17.59026021	9.58E-05	0.0004306
**ENSG00000133657**	ATP13A3	0.293992454	7.218533124	17.57110886	9.66E-05	0.0004338
**ENSG00000156256**	USP16	-0.498447183	6.80185214	18.17173095	9.67E-05	0.0004340

**ENSG00000224086**		-0.591180228	5.541784373	17.64008367	9.72E-05	0.0004363
**ENSG00000279759**		-1.038177357	3.753520473	17.55084328	9.74E-05	0.0004368
**ENSG00000140983**	RHOT2	-1.066010681	4.965207857	18.80536972	9.78E-05	0.0004385
**ENSG00000141002**	TCF25	-0.413642592	6.459810691	17.5345255	9.8E-05	0.0004393
**ENSG00000144118**	RALB	0.662691887	4.00864142	17.51941286	9.86E-05	0.0004418
**ENSG00000100485**	SOS 2	0.317061194	6.84063217	17.51784245	9.87E-05	0.0004419
**ENSG00000164241**	c5orf63	-0.9192993	4.074823212	17.50907269	9.9E-05	0.0004432
**ENSG00000141378**	PTRH2	0.39732604	5.686084473	17.50514099	9.92E-05	0.0004437
**ENSG00000143882**	ATP6V1C2	0.606869652	4.960862746	17.49749868	9.95E-05	0.0004449
**ENSG00000131381**	RBSN	-0.418106132	5.652185642	17.49419167	9.96E-05	0.0004453
**ENSG00000242861**		-0.825947898	4.221431947	17.49334483	9.97E-05	0.0004453
**ENSG00000008324**	SS18L2	0.766857867	3.955761217	17.49226223	9.97E-05	0.0004453
**ENSG00000211790**		0.646479961	4.10483105	17.49024855	9.98E-05	0.0004455
**ENSG00000143753**	DEGS1	0.487035446	5.775885242	17.47283467	0.0001004	0.0004484
**ENSG00000128016**	ZFP36	0.498699186	6.919088663	18.22715914	0.0001006	0.0004490
**ENSG00000126945**	HNRNPH2	0.415161636	6.00658215	17.43418967	0.0001020	0.0004551
**ENSG00000106952**	TNFSF8	0.362682643	6.277936039	17.41624219	0.0001027	0.0004582
**ENSG00000243964**		1.106513838	4.006137519	18.11794853	0.0001033	0.0004606
**ENSG00000234009**		0.902443133	3.639074523	17.39986361	0.0001034	0.0004608
**ENSG00000183049**	CAMK1D	0.374185677	6.202032041	17.38728882	0.0001040	0.0004630
**ENSG00000226742**	Hsbp1l1	-0.931006996	3.767824373	17.37830184	0.0001043	0.0004644
**ENSG00000096968**	JAK2	0.46550401	5.932997689	17.37007274	0.0001047	0.0004658
**ENSG00000085449**	WDFY1	0.39703697	5.793452416	17.36188546	0.0001050	0.0004671
**ENSG00000198663**	C6orf89	0.337987931	6.298014623	17.36109223	0.0001051	0.0004671
**ENSG00000211793**		0.666323075	4.411328745	17.34121609	0.0001059	0.0004706
**ENSG00000197969**	VPS13A	-0.338112907	7.873737087	17.3327411	0.0001063	0.0004721
**ENSG00000100934**	SEC23A	0.341518273	6.397098232	17.30780756	0.0001073	0.0004766
**ENSG00000142347**	MYO1F	0.673352763	6.935687357	18.74646167	0.0001077	0.0004777
**ENSG00000241058**	NSUN6	-0.591774033	5.163681584	17.29991151	0.0001077	0.0004777
**ENSG00000014138**	POLA2	-0.67037691	4.400663334	17.29365736	0.0001080	0.0004788
**ENSG00000187555**	USP7	0.234088154	8.049581908	17.28069296	0.0001085	0.0004811
**ENSG00000134255**	CEPT1	-0.407571695	7.627143289	17.72280407	0.0001087	0.0004815
**ENSG00000132382**	MYBBP1A	-0.55568216	5.375953691	17.26810236	0.0001091	0.0004830
**ENSG00000280734**		-0.924429315	3.745042703	17.26765598	0.0001091	0.0004830
**ENSG00000139714**	MORN3	-0.811006295	4.048374938	17.25713692	0.0001096	0.0004848
**ENSG00000168016**	TRANK1	0.285569797	7.902347801	17.24401323	0.0001102	0.0004872
**ENSG00000143643**	TTC13	-0.578212577	5.988020619	17.73438095	0.0001103	0.0004878

**ENSG00000131732**	ZCCHC9	-0.578702464	4.790338671	17.22619481	0.0001109	0.0004903
**ENSG00000146409**	SLC18B1	-0.621365947	4.639291761	17.22507488	0.0001110	0.0004903
**ENSG00000171456**	ASXL1	-0.327187866	7.047749305	17.22422464	0.0001110	0.0004903
**ENSG00000102226**	USP11	-0.443804224	5.402647925	17.21541015	0.0001114	0.0004918
**ENSG00000154874**	Ccdc144b	-0.678720541	4.859745977	17.21247785	0.0001116	0.0004922
**ENSG00000007923**	DNAJC11	-0.588499235	5.051245719	17.20616176	0.0001119	0.0004933
**ENSG00000197943**	PLCG2	1.139221076	4.445561508	18.37447567	0.0001123	0.0004949
**ENSG00000143612**	c1orf43	0.490108363	5.566377618	17.17158533	0.0001134	0.0004998
**ENSG00000157617**	C2CD2	-0.884810649	3.830992488	17.17060545	0.0001135	0.0004998
**ENSG00000145687**	SSBP2	-0.497288665	5.972260284	17.16412103	0.0001138	0.0005010
**ENSG00000168646**	AXIN2	-0.525933619	5.355288568	17.15299474	0.0001143	0.0005029
**ENSG00000211788**		0.605929269	4.222317896	17.14999393	0.0001144	0.0005033
**ENSG00000276664**		-0.721662702	4.012683218	17.14733624	0.0001145	0.0005037
**ENSG00000164308**	ERAP2	-0.433061628	7.817366201	17.82040395	0.0001147	0.0005042
**ENSG00000214029**	ZNF891	-0.535092636	5.800067777	17.26179204	0.0001149	0.0005049
**ENSG00000073849**	st6gal1	0.281378254	7.886077853	17.13844052	0.0001150	0.0005049
**ENSG00000182087**	TMEM259	-0.93574439	6.051478446	18.6075213	0.0001157	0.0005079
**ENSG00000171132**	PRKCE	0.701790339	4.219995009	17.11985446	0.0001158	0.0005082
**ENSG00000147118**	ZNF182	-0.591385439	4.784637335	17.11707578	0.0001160	0.0005086
**ENSG00000001631**	KRIT1	-0.406435859	6.865069223	17.18352959	0.0001160	0.0005087
**ENSG00000146830**	gigyf1	-0.492665956	6.06348041	17.21343791	0.0001164	0.0005100
**ENSG00000160179**	ABCG1	-0.946696721	3.994431858	17.10610417	0.0001165	0.0005102
**ENSG00000182054**	IDH2	0.506922294	5.126059469	17.10523889	0.0001165	0.0005102
**ENSG00000164828**	SUN1	-0.45555712	6.550249954	17.28020054	0.0001166	0.0005102
**ENSG00000089818**	NECAP1	-0.423126765	5.630114677	17.1012826	0.0001167	0.0005106
**ENSG00000123144**	c19orf43	0.470892069	5.843166244	17.08959007	0.0001173	0.0005128
**ENSG00000169762**	TAPT1	-0.406383016	5.731326401	17.07332537	0.0001180	0.0005160
**ENSG00000189159**	HN1	0.551505378	5.510966747	17.06844985	0.0001183	0.0005169
**ENSG00000048392**	RRM2B	0.577992451	4.63832564	17.06150372	0.0001186	0.0005180
**ENSG00000268575**		-0.427328109	6.569048161	17.06753339	0.0001190	0.0005195
**ENSG00000109814**	UGDH	0.771139551	4.410254572	17.04937688	0.0001192	0.0005201
**ENSG00000189241**	TSPYL1	0.348131457	7.149404046	17.0385518	0.0001197	0.0005222
**ENSG00000120451**	SNX19	-0.302635553	6.518913397	17.03113024	0.0001201	0.0005235
**ENSG00000145741**	BTF3	0.44543491	8.458198845	17.9295346	0.0001201	0.0005235
**ENSG00000166716**	ZNF592	0.339248418	6.527092567	17.02959935	0.0001202	0.0005235
**ENSG00000279716**		-0.827741315	3.908663213	17.00906099	0.0001212	0.0005276
**ENSG00000207039**		2.299559386	5.459854527	18.52507494	0.0001212	0.0005277

**ENSG00000131725**	WDR44	0.378154124	5.835465988	17.00293485	0.0001215	0.0005285
**ENSG00000043093**	DCUN1D1	0.377755683	6.03008744	17.00095652	0.0001216	0.0005287
**ENSG00000170779**	CDCA4	0.784997493	3.55891265	16.99536107	0.0001218	0.0005297
**ENSG00000067182**	TNFRSF1A	0.492703428	5.231842534	16.98080853	0.0001226	0.0005326
**ENSG00000164211**	STARD4	0.503596007	6.137839259	17.26028292	0.0001226	0.0005326
**ENSG00000094631**	HDAC6	-0.625414074	5.093486907	16.97197113	0.0001230	0.0005341
**ENSG00000136271**	DDX56	-0.602482221	5.450667497	17.08912188	0.0001233	0.0005352
**ENSG00000166887**	VPS39	-0.385617303	6.336830895	16.96374778	0.0001234	0.0005354
**ENSG00000129933**	MAU2	-0.386275871	6.220124211	16.95126364	0.0001240	0.0005379
**ENSG00000171055**	FEZ2	0.602883141	4.673255031	16.94998561	0.0001241	0.0005380
**ENSG00000199565**		2.292398936	5.459001888	18.43639169	0.0001250	0.0005418
**ENSG00000143258**	USP21	-0.75798523	4.486169806	16.92356115	0.0001254	0.0005433
**ENSG00000131791**	PRKAB2	-0.724873631	5.021010559	17.16219889	0.0001257	0.0005440
**ENSG00000258366**	RTEL1	-0.71053593	4.473016739	16.91250457	0.0001260	0.0005453
**ENSG00000258830**		-0.425292823	5.739988525	16.91157615	0.0001261	0.0005453
**ENSG00000197586**	ENTPD6	-0.561816806	4.738974369	16.90808331	0.0001262	0.0005458
**ENSG00000100325**	ASCC2	-0.417825618	5.686768476	16.90351286	0.0001265	0.0005466
**ENSG00000180822**	PSMG4	-0.53263004	5.089091853	16.89091033	0.0001271	0.0005492
**ENSG00000239653**	PSMD6-AS2	-0.64136762	5.301143796	17.07757237	0.0001273	0.0005496
**ENSG00000242299**		0.92370643	5.892874835	18.32759967	0.0001277	0.0005514
**ENSG00000102471**	NDFIP2	0.785292477	3.334832801	16.87774705	0.0001278	0.0005515
**ENSG00000156398**	SFXN2	-0.714741152	4.455978642	16.85825922	0.0001288	0.0005556
**ENSG00000176623**	RMDN1	-0.380246959	6.338432581	16.83542504	0.0001300	0.0005606
**ENSG00000104177**	MYEF2	-0.70708897	4.765691849	16.8310292	0.0001303	0.0005614
**ENSG00000060971**	ACAA1	-0.646836394	4.958212087	16.82800919	0.0001304	0.0005617
**ENSG00000006015**	c19orf60	-0.758660269	4.221396231	16.82741287	0.0001304	0.0005617
**ENSG00000218283**		0.654741902	4.516085546	16.82146559	0.0001308	0.0005629
**ENSG00000251357**		0.794510082	4.764790022	17.1071236	0.0001316	0.0005661
**ENSG00000100629**	CEP128	0.612559912	5.241338059	16.85768142	0.0001318	0.0005668
**ENSG00000025770**	NCAPH2	-0.663505385	4.269376981	16.80131182	0.0001318	0.0005668
**ENSG00000071051**	NCK2	0.350039593	6.735979151	16.79551027	0.0001322	0.0005679
**ENSG00000179833**	SERTAD2	-0.45402443	6.321854615	16.89088266	0.0001325	0.0005691
**ENSG00000146067**	FAM193B	-0.79217753	5.747744384	17.92647651	0.0001330	0.0005709
**ENSG00000134146**	DPH6	-0.809659169	4.0261449	16.77944042	0.0001330	0.0005709
**ENSG00000235954**	TTC28-AS1	-0.699616188	4.401323721	16.76463054	0.0001338	0.0005742
**ENSG00000197535**	MYO5A	0.336670877	7.359625561	16.76269105	0.0001339	0.0005744
**ENSG00000283795**		0.925337076	4.782559755	17.69077915	0.0001343	0.0005759

**ENSG00000156521**	TYSND1	-0.767368862	4.157386879	16.74243805	0.0001350	0.0005787
**ENSG00000156858**	prr14	-0.66537437	4.666361979	16.73997107	0.0001352	0.0005790
**ENSG00000104047**	DTWD1	-0.531074294	4.994064385	16.72889292	0.0001358	0.0005814
**ENSG00000100647**	SUSD6	0.388607486	6.099392875	16.72649751	0.0001359	0.0005817
**ENSG00000083223**	ZCCHC6	0.324922114	7.15793867	16.71890624	0.0001363	0.0005833
**ENSG00000279232**		-0.835466328	3.875514684	16.71112167	0.0001368	0.0005849
**ENSG00000189079**	ARID2	0.2781481	7.606507197	16.70899467	0.0001369	0.0005852
**ENSG00000077585**	GPR137B	0.75203834	3.978524853	16.69851652	0.0001375	0.0005874
**ENSG00000106404**	CLDN15	-0.955190513	3.666028303	16.69413019	0.0001377	0.0005882
**ENSG00000284195**		0.834053004	3.73154244	16.69042813	0.0001379	0.0005889
**ENSG00000140350**	ANP32A	0.348133847	6.572340665	16.68699546	0.0001381	0.0005895
**ENSG00000167447**	SMG8	0.699304691	4.309786567	16.67560821	0.0001388	0.0005920
**ENSG00000267349**		0.603887125	4.498070655	16.67392309	0.0001389	0.0005921
**ENSG00000254788**	CKLF-CMTM1	0.609206847	4.448120974	16.67219335	0.0001390	0.0005923
**ENSG00000142327**		0.573045122	6.591259435	17.5875746	0.0001397	0.0005953
**ENSG00000264577**		0.483339334	8.739626731	17.75987954	0.0001400	0.0005963
**ENSG00000149806**	FAU	0.655202768	7.983953352	18.0724729	0.0001403	0.0005973
**ENSG00000188612**	SUMO2	0.395092013	6.74657211	16.64280148	0.0001406	0.0005985
**ENSG00000131171**	SH3BGRL	0.336730818	6.85726241	16.64089262	0.0001408	0.0005987
**ENSG00000168298**	HIST1H1E	0.646338413	8.277892263	18.01626289	0.0001430	0.0006079
**ENSG00000101160**	CTSZ	0.821069981	3.764675964	16.60151024	0.0001430	0.0006079
**ENSG00000279649**		-0.575428358	5.146851493	16.58448809	0.0001440	0.0006118
**ENSG00000117862**	TXNDC12	0.503304725	5.17993881	16.58412065	0.0001441	0.0006118
**ENSG00000125249**	RAP2A	0.54329528	5.536336811	16.59522004	0.0001445	0.0006136
**ENSG00000132155**	RAF1	-0.317450561	6.885103058	16.56978288	0.0001449	0.0006149
**ENSG00000150054**	MPP7	-0.410136116	6.173457528	16.55365125	0.0001459	0.0006187
**ENSG00000249592**	LOC100129917	-0.845405656	4.462835177	16.68840796	0.0001459	0.0006188
**ENSG00000151366**	NDUFC2	0.706943606	4.490038171	16.54952058	0.0001461	0.0006192
**ENSG00000230590**	FTX	-0.393088527	7.738617133	16.95862192	0.0001464	0.0006201
**ENSG00000217555**	cklf	0.567139558	4.650661189	16.54427046	0.0001464	0.0006201
**ENSG00000169609**	C15orf40	-0.418004476	5.456194857	16.53464044	0.0001470	0.0006222
**ENSG00000101310**	SEC23B	0.446112978	5.681898929	16.53383858	0.0001470	0.0006222
**ENSG00000175066**	GK5	-0.633531871	5.304643987	16.68266057	0.0001477	0.0006246
**ENSG00000054118**	THRAP3	0.270609238	7.587064413	16.51536918	0.0001482	0.0006264
**ENSG00000197728**	RPS26	0.488453183	7.486368205	17.30441252	0.0001487	0.0006285
**ENSG00000167468**	GPX4	0.519398475	4.998090056	16.50098476	0.0001490	0.0006294
**ENSG00000152495**	CAMK4	-0.353681262	8.805298195	16.71256598	0.0001491	0.0006294

**ENSG00000131844**	MCCC2	-0.438039885	5.55837589	16.49663828	0.0001493	0.0006301
**ENSG00000176422**	spryd4	-0.600678711	4.586704242	16.49615437	0.0001493	0.0006301
**ENSG00000120742**	SERP1	0.291861508	7.169181222	16.4901386	0.0001497	0.0006314
**ENSG00000029363**	BCLAF1	-0.249066851	8.426681303	16.4876858	0.0001499	0.0006318
**ENSG00000062650**	WAPL	0.27746134	7.714140592	16.48479051	0.0001500	0.0006323
**ENSG00000196684**	HSH2D	-0.947029146	3.872391113	16.47189544	0.0001508	0.0006354
**ENSG00000215440**	NPEPL1	-0.927624756	4.627741695	17.03468682	0.0001509	0.0006355
**ENSG00000100918**	REC8	-0.974454675	4.252589706	16.74863492	0.0001511	0.0006360
**ENSG00000273891**		-0.860559502	3.865236863	16.46091154	0.0001515	0.0006374
**ENSG00000196911**	KPNA5	-0.364927731	6.637866824	16.4597393	0.0001516	0.0006375
**ENSG00000179295**	PTPN11	0.312329534	6.821825762	16.45730307	0.0001517	0.0006379
**ENSG00000151923**	TIAL1	-0.297720941	7.616867523	16.43489233	0.0001531	0.0006435
**ENSG00000160305**	DIP2A	-0.549484117	7.152676274	17.49734842	0.0001539	0.0006465
**ENSG00000140992**	PDPK1	0.349038005	5.839501641	16.39330726	0.0001558	0.0006540
**ENSG00000234545**	FAM133B	-0.507157599	5.284708633	16.38456963	0.0001563	0.0006560
**ENSG00000158552**	ZFAND2B	-0.617396382	4.468677704	16.38394467	0.0001564	0.0006560
**ENSG00000185947**	ZNF267	0.434558918	5.86782394	16.35932704	0.0001579	0.0006624
**ENSG00000105514**	RAB3D	0.866522609	3.484938701	16.34687959	0.0001588	0.0006655
**ENSG00000237350**		0.91318443	3.507704021	16.32702871	0.0001601	0.0006707
**ENSG00000227155**		-0.697881871	4.817952665	16.32300945	0.0001603	0.0006716
**ENSG00000279059**		-0.788938964	3.770775011	16.29873296	0.0001619	0.0006779
**ENSG00000105926**	MPP6	-0.563517928	5.458930464	16.2981668	0.0001620	0.0006779
**ENSG00000164244**	PRRC1	0.357223792	6.431989839	16.29718607	0.0001620	0.0006779
**ENSG00000176658**	MYO1D	0.608952358	4.340596441	16.29330025	0.0001623	0.0006787
**ENSG00000127947**	PTPN12	0.45179688	5.146830737	16.27835243	0.0001633	0.0006826
**ENSG00000157379**	DHRS1	-0.682033102	3.893249808	16.27484066	0.0001635	0.0006833
**ENSG00000124299**	PEPD	0.671261498	4.383628465	16.27094065	0.0001638	0.0006841
**ENSG00000083896**	YTHDC1	-0.25239548	7.906092965	16.26340759	0.0001643	0.0006858
**ENSG00000214753**	HNRNPUL2	0.314137658	7.503749944	16.26309287	0.0001643	0.0006858
**ENSG00000196839**	ADA	-0.820520002	3.957911829	16.24818351	0.0001653	0.0006897
**ENSG00000234663**	LOC101927156	0.564331935	4.537786077	16.23627538	0.0001661	0.0006929
**ENSG00000138448**	ITGAV	0.580618245	4.800726734	16.21915201	0.0001673	0.0006975
**ENSG00000028310**	BRD9	-0.446400052	6.180780617	16.2152094	0.0001676	0.0006983
**ENSG00000168283**	BMI1	0.313223148	6.738328353	16.21420785	0.0001677	0.0006983
**ENSG00000037474**	NSUN2	-0.329861905	6.34158026	16.21110153	0.0001679	0.0006989
**ENSG00000148444**	COMMD3	-0.550044422	4.794819887	16.20306813	0.0001684	0.0007009
**ENSG00000089876**	DHX32	-0.713628911	3.974993518	16.20254781	0.0001685	0.0007009

**ENSG00000119596**	YLPM1	-0.30985692	7.531562233	16.18384996	0.0001698	0.0007060
**ENSG00000103227**	LMF1	-0.890438275	4.340277125	16.34295178	0.0001700	0.0007068
**ENSG00000278600**		-0.769355308	4.155999378	16.16687842	0.0001709	0.0007104
**ENSG00000260682**		1.184983126	4.277235021	17.23565739	0.0001712	0.0007111
**ENSG00000047578**	KIAA0556	-0.66338423	4.260054608	16.15276633	0.0001719	0.0007139
**ENSG00000184402**	SS18L1	-0.734194893	4.410749393	16.14566091	0.0001724	0.0007157
**ENSG00000114209**	PDCD10	0.400149206	6.03493542	16.14255554	0.0001727	0.0007164
**ENSG00000181061**	HIGD1A	0.686780844	4.555907585	16.13103055	0.0001735	0.0007195
**ENSG00000029364**	SLC39A9	0.363780247	6.04819628	16.12662494	0.0001738	0.0007205
**ENSG00000189343**		0.855175583	5.453247383	17.29409792	0.0001740	0.0007212
**ENSG00000172354**	GNB2	0.572906957	5.068333571	16.12030184	0.0001743	0.0007218
**ENSG00000165272**	AQP3	0.616195899	6.542240552	17.17438593	0.0001754	0.0007263
**ENSG00000198286**	CARD11	-0.267632082	7.032440926	16.09999148	0.0001757	0.0007273
**ENSG00000108262**	GIT1	0.473512502	5.553970356	16.08868055	0.0001765	0.0007304
**ENSG00000130958**	SLC35D2	0.713544326	4.21763103	16.07825519	0.0001773	0.0007333
**ENSG00000003509**	NDUFAF7	-0.601888579	4.99855083	16.07488734	0.0001775	0.0007340
**ENSG00000101557**	USP14	0.313055018	6.634943859	16.07238994	0.0001777	0.0007345
**ENSG00000269352**		-0.865130265	4.040566105	16.05393562	0.0001791	0.0007398
**ENSG00000213553**		0.618924593	5.605290696	16.55804371	0.0001794	0.0007409
**ENSG00000269900**	RMRP	1.373936188	9.1508622	17.39606833	0.0001802	0.0007434
**ENSG00000277027**	RMRP	1.373936188	9.1508622	17.39606833	0.0001802	0.0007434
**ENSG00000276965**		-0.52878519	5.09121579	16.03811837	0.0001803	0.0007434
**ENSG00000100416**	TRMU	-0.826414041	4.424517732	16.11101087	0.0001815	0.0007482
**ENSG00000278771**		1.585007252	8.634931533	17.37348725	0.0001816	0.0007482
**ENSG00000109184**	DCUN1D4	-0.422344374	5.737281334	16.01887092	0.0001817	0.0007482
**ENSG00000186318**	BACE1	0.508867181	5.413145561	16.01885588	0.0001817	0.0007482
**ENSG00000010818**	HIVEP2	0.265922276	8.570458042	16.00627791	0.0001826	0.0007518
**ENSG00000124789**	NUP153	0.262208181	7.430110353	15.98816148	0.0001840	0.0007571
**ENSG00000101158**	NELFCD	-0.413209895	5.844108415	15.97871946	0.0001847	0.0007598
**ENSG00000108559**	NUP88	-0.325811997	6.431472976	15.97197017	0.0001853	0.0007615
**ENSG00000139977**	NAA30	0.38558168	5.932642183	15.97127124	0.0001853	0.0007615
**ENSG00000214176**		-0.668072516	5.89141597	16.79635793	0.0001863	0.0007653
**ENSG00000078747**	ITCH	0.303648149	7.034540327	15.91637617	0.0001896	0.0007784
**ENSG00000079999**	KEAP1	0.811310582	3.677863395	15.9124523	0.0001899	0.0007793
**ENSG00000146350**	TBC1D32	-0.699240803	4.69814027	15.88766979	0.0001918	0.0007871
**ENSG00000130429**	ARPC1B	0.368093191	6.784452677	15.88424535	0.0001921	0.0007879
**ENSG00000229124**	VIM-AS1	0.663211692	7.122005145	17.14162754	0.0001923	0.0007885

**ENSG00000185825**	BCAP31	0.464804121	5.639235876	15.87714379	0.0001927	0.0007896
**ENSG00000197006**	METTL9	0.401770593	6.009072635	15.87519823	0.0001928	0.0007899
**ENSG00000131375**	CAPN7	-0.391558481	6.800577431	15.89443765	0.0001934	0.0007918
**ENSG00000281896**		-0.611655289	4.679880886	15.85184527	0.0001947	0.0007969
**ENSG00000255302**	EID1	0.428163494	6.47416789	15.95596824	0.0001948	0.0007972
**ENSG00000112406**	HECA	0.218033335	8.486036744	15.84756031	0.0001950	0.0007977
**ENSG00000068724**	TTC7A	0.458397741	5.349259822	15.83819361	0.0001958	0.0008005
**ENSG00000171453**	POLR1C	-0.714726061	3.895592438	15.82985451	0.0001965	0.0008030
**ENSG00000271383**	LOC100996717	-0.397141518	5.901909433	15.82771869	0.0001966	0.0008034
**ENSG00000213742**	ZNF337-AS1	-0.491529691	5.159578744	15.82362571	0.0001970	0.0008044
**ENSG00000225948**		-0.639084463	4.303363355	15.82121398	0.0001972	0.0008049
**ENSG00000230325**		-0.859590962	3.49833821	15.81800774	0.0001974	0.0008056
**ENSG00000259529**		-0.410739838	6.385512691	15.80656953	0.0001984	0.0008092
**ENSG00000240342**		0.629292033	5.652988902	16.4201794	0.0001992	0.0008122
**ENSG00000213281**	NRAS	0.390472492	6.066339719	15.79157273	0.0001996	0.0008136
**ENSG00000251791**	Scarna6	1.565557616	5.254085829	17.1036382	0.0001999	0.0008144
**ENSG00000271816**	BMS1P4	-0.894993089	3.919761298	15.78713911	0.0002000	0.0008144
**ENSG00000087589**	cass4	-0.855255354	4.111530118	15.77652973	0.0002008	0.0008177
**ENSG00000132405**	TBC1D14	0.310161248	6.489578564	15.74325554	0.0002036	0.0008287
**ENSG00000114861**	FOXP1	-0.267457029	8.919017189	15.73617198	0.0002042	0.0008308
**ENSG00000167272**	POP5	-0.820353273	4.154930987	15.73505799	0.0002043	0.0008309
**ENSG00000172661**	FAM21C	-0.432780455	5.791327368	15.72869973	0.0002049	0.0008328
**ENSG00000153487**	ING1	0.52177784	5.086517734	15.72009954	0.0002056	0.0008354
**ENSG00000103496**	STX4	-0.534835753	4.605114467	15.70725787	0.0002067	0.0008395
**ENSG00000164323**	CFAP97	0.309474632	6.872702245	15.7034758	0.0002070	0.0008405
**ENSG00000126217**	MCF2L	-0.879687251	3.955984876	15.69926048	0.0002074	0.0008417
**ENSG00000135823**	STX6	0.47042552	5.487543054	15.69499875	0.0002077	0.0008428
**ENSG00000078142**	PIK3C3	-0.295119356	6.858254598	15.69370721	0.0002079	0.0008430
**ENSG00000125870**	SNRPB2	0.468563163	5.296123294	15.68231035	0.0002088	0.0008466
**ENSG00000251247**	ZNF345	-0.66629886	4.390540968	15.66744532	0.0002101	0.0008515
**ENSG00000167397**	vkorc1	0.785606918	3.846526963	15.65526737	0.0002112	0.0008555
**ENSG00000156313**	RPGR	-0.793918522	4.389558751	15.64157762	0.0002124	0.0008601
**ENSG00000121741**	ZMYM2	-0.236854535	7.833700536	15.62360262	0.0002140	0.0008662
**ENSG00000172803**	SNX32	0.44675573	6.328845009	15.76671867	0.0002142	0.0008666
**ENSG00000084207**	GSTP1	0.745591743	3.996316062	15.62048809	0.0002143	0.0008666
**ENSG00000067992**	PDK3	0.464276817	4.912832185	15.60359436	0.0002158	0.0008724
**ENSG00000280987**	MATR3	0.254640978	8.897468552	15.594402	0.0002166	0.0008752

**ENSG00000106682**	EIF4H	0.339020057	6.463901104	15.59387628	0.0002167	0.0008752
**ENSG00000084072**	PPIE	-0.66100554	5.351905087	15.9547154	0.0002170	0.0008764
**ENSG00000169871**	TRIM56	-0.34073404	7.070585395	15.58364446	0.0002176	0.0008780
**ENSG00000085117**	CD82	0.595452407	4.660348565	15.58351334	0.0002176	0.0008780
**ENSG00000229097**		-0.928337301	3.680556391	15.57612279	0.0002183	0.0008800
**ENSG00000167081**	PBX3	-0.665641422	4.421101753	15.57605839	0.0002183	0.0008800
**ENSG00000142330**	CAPN10	-0.866666741	4.254754951	15.68003816	0.0002188	0.0008819
**ENSG00000196116**	TDRD7	0.51289254	4.732269521	15.56790561	0.0002190	0.0008823
**ENSG00000116459**	ATP5F1	0.372001612	6.456521231	15.56333472	0.0002194	0.0008837
**ENSG00000144524**	COPS7B	-0.520906154	5.158090883	15.56149814	0.0002196	0.0008840
**ENSG00000110911**	SLC11A2	-0.577793649	5.570976819	15.76527258	0.0002198	0.0008845
**ENSG00000222328**		1.272437482	7.685557173	16.82789015	0.0002207	0.0008879
**ENSG00000142765**	SYTL1	-0.720750854	5.673068524	16.36235229	0.0002209	0.0008882
**ENSG00000116977**	LGALS8	-0.315852114	7.701624524	15.53901522	0.0002217	0.0008909
**ENSG00000239697**	TNFSF12	0.663111811	4.148084231	15.53330377	0.0002222	0.0008927
**ENSG00000171103**	trmt61b	-0.735813192	4.126247845	15.52692586	0.0002228	0.0008947
**ENSG00000164896**	FASTK	-0.827275597	4.597300471	15.83201524	0.0002230	0.0008950
**ENSG00000177337**		0.702797907	3.81196884	15.52404413	0.0002230	0.0008950
**ENSG00000053702**	NRIP2	-0.788950529	3.955590106	15.52349393	0.0002231	0.0008950
**ENSG00000080546**	SESN1	-0.397723032	6.20783707	15.51958433	0.0002235	0.0008961
**ENSG00000268858**		-0.964589995	3.587989507	15.50561815	0.0002248	0.0009010
**ENSG00000112658**	SRF	0.587368824	4.683473901	15.49382108	0.0002259	0.0009051
**ENSG00000162734**	PEA15	0.570532136	4.37118764	15.48227236	0.000227	0.0009091
**ENSG00000165525**	NEMF	-0.299851589	7.56887607	15.47367507	0.0002278	0.0009120
**ENSG00000163608**	NEPRO	-0.338760966	6.611457757	15.47048315	0.0002281	0.0009129
**ENSG00000147403**	RPL10	0.381534001	10.13148006	15.8586526	0.0002286	0.0009144
**ENSG00000243156**	MICAL3	0.53711487	5.337766024	15.45049306	0.0002300	0.0009198
**ENSG00000132781**	MUTYH	-0.841014426	3.900028468	15.44526439	0.0002305	0.0009214
**ENSG00000120727**	PAIP2	0.400356092	6.640538743	15.51039492	0.0002307	0.0009220
**ENSG00000196465**	MYL6B	0.48843364	5.238670079	15.44080411	0.0002309	0.0009224
**ENSG00000142634**	EFHD2	0.640352472	4.789922471	15.43363563	0.0002316	0.0009246
**ENSG00000106771**	tmem245	0.282092382	7.18866077	15.43330029	0.0002316	0.0009246
**ENSG00000114316**	USP4	-0.328973894	6.515475741	15.4321868	0.0002317	0.0009247
**ENSG00000153815**	CMIP	0.378126487	5.929199686	15.42165275	0.0002328	0.0009284
**ENSG00000136068**	FLNB	-0.430725256	6.135529727	15.41386983	0.0002335	0.0009311
**ENSG00000176542**	USF3	0.301393581	7.464510066	15.41059613	0.0002338	0.0009320
**ENSG00000119943**	MIR1287	-0.952333661	3.63238963	15.40347124	0.0002345	0.0009340

**ENSG00000055609**	KMT2C	0.249422793	9.146727058	15.40345683	0.0002345	0.0009340
**ENSG00000101079**	NDRG3	0.442178939	5.546830732	15.40060162	0.0002348	0.0009348
**ENSG00000070476**	ZXDC	-0.395139519	5.846515141	15.39755425	0.0002351	0.0009356
**ENSG00000207008**	SNORA54	1.548915004	5.07844752	16.64847176	0.0002353	0.0009360
**ENSG00000072501**	SMC1A	0.284063808	7.582184818	15.39483846	0.0002354	0.0009360
**ENSG00000166313**	APBB1	-0.507880488	5.45888293	15.39350081	0.0002355	0.0009362
**ENSG00000272030**		-0.720667947	3.855353718	15.3775015	0.0002371	0.0009421
**ENSG00000213079**	scaf8	0.272861091	7.387381646	15.37528875	0.0002373	0.0009426
**ENSG00000184209**	SNRNP35	-0.529656068	4.801035561	15.36981305	0.0002378	0.0009444
**ENSG00000249264**		0.88150412	3.971891683	15.51829621	0.0002388	0.0009480
**ENSG00000178057**	NDUFAF3	0.721755738	4.424286757	15.35822578	0.0002390	0.0009482
**ENSG00000185787**	MORF4L1	0.226269208	7.73516	15.34968576	0.0002398	0.0009512
**ENSG00000104763**	ASAH1	-0.387468417	5.880860476	15.34857718	0.0002400	0.0009513
**ENSG00000124226**	RNF114	-0.364422581	6.028876449	15.34318141	0.0002405	0.0009531
**ENSG00000165915**	SLC39A13	-0.737617428	4.162170901	15.33262059	0.0002416	0.0009570
**ENSG00000269890**		-0.670303161	4.023649094	15.32652293	0.0002422	0.0009590
**ENSG00000154582**	TCEB1	0.615377117	4.680822082	15.32400633	0.0002424	0.0009597
**ENSG00000118922**	KLF12	0.218424951	8.312606273	15.31046274	0.0002438	0.0009647
**ENSG00000154743**	TSEN2	-0.688739885	4.133154867	15.30972116	0.0002439	0.0009647
**ENSG00000272498**		-0.726025204	4.497975495	15.29787916	0.0002451	0.0009691
**ENSG00000176105**	YES1	0.694274358	3.734311761	15.28867185	0.0002460	0.0009725
**ENSG00000131876**	SNRPA1	-0.664317802	5.793447494	16.00589892	0.0002473	0.0009770
**ENSG00000183741**	CBX6	0.389103667	5.625526591	15.27345861	0.0002476	0.0009779
**ENSG00000211810**		0.81222599	3.604736236	15.26037072	0.0002490	0.0009829
**ENSG00000074855**	ANO8	-1.173408475	4.216605615	15.98706569	0.0002492	0.0009837
**ENSG00000242338**		-0.92240297	3.8913215	15.23669691	0.0002514	0.0009918
**ENSG00000065883**	CDK13	0.247281892	7.454273956	15.23610272	0.0002515	0.0009918
**ENSG00000072518**	MARK2	0.30371998	6.283804438	15.23319782	0.0002518	0.0009926
**ENSG00000113300**	CNOT6	0.331738103	6.526852123	15.23215598	0.0002519	0.0009927
**ENSG00000103363**	TCEB2	0.677887959	4.244854789	15.22428144	0.0002527	0.0009956
**ENSG00000137494**	ANKRD42	-0.737891131	4.286796628	15.22297433	0.0002529	0.0009958
**ENSG00000198160**	MIER1	0.262184057	7.769162715	15.21913729	0.0002533	0.0009970
**ENSG00000273899**	NOL12	-0.909702928	3.759110271	15.21177418	0.0002541	0.0009997
**ENSG00000270012**		-0.927125809	3.822971195	15.21691869	0.0002544	0.0010008
**ENSG00000089327**	FXYD5	0.345964747	8.374567885	15.43420426	0.0002548	0.0010016
**ENSG00000258539**		0.417703454	5.48411385	15.19498805	0.0002559	0.0010056
**ENSG00000168872**	DDX19A	-0.404346221	5.561140473	15.18280601	0.0002572	0.0010103

**ENSG00000273149**		0.439232017	10.12971978	15.91821897	0.0002589	0.0010166
**ENSG00000181638**	ZFP41	-0.830572911	3.870018641	15.15432349	0.0002602	0.0010217
**ENSG00000146909**	NOM1	-0.45645827	5.484527465	15.15196714	0.0002605	0.0010222
**ENSG00000165521**	EML5	-0.494442628	5.328791855	15.15137048	0.0002606	0.0010222
**ENSG00000116668**	SWT1	-0.524589717	4.752137921	15.1492055	0.0002608	0.0010227
**ENSG00000258908**		0.806196168	3.922223739	15.13364764	0.0002625	0.0010290
**ENSG00000196284**	SUPT3H	-0.622444398	4.487022876	15.12906545	0.0002630	0.0010306
**ENSG00000018189**	RUFY3	-0.560630941	4.948422532	15.12726231	0.0002632	0.0010310
**ENSG00000267387**		-1.014427131	3.525472195	15.11152526	0.0002650	0.0010374
**ENSG00000142794**	NBPF3	-0.794384886	3.877903445	15.10782631	0.0002654	0.0010387
**ENSG00000200488**		1.414162557	4.491086581	16.28061111	0.0002662	0.0010414
**ENSG00000276291**		-0.545571645	4.856598319	15.09894577	0.0002664	0.0010417
**ENSG00000123329**	ARHGAP9	-0.506800873	6.690779024	15.71674686	0.0002678	0.0010469
**ENSG00000196912**	ANKRD36B	-0.432795369	6.101098716	15.07682464	0.0002688	0.0010507
**ENSG00000231752**	EMBP1	0.528326761	4.546580892	15.06843068	0.0002698	0.0010540
**ENSG00000165689**	SDCCAG3	-0.637708262	5.016884543	15.09913199	0.0002721	0.0010625
**ENSG00000182827**	ACBD3	0.436120803	5.825272545	15.04646075	0.0002723	0.0010629
**ENSG00000115760**	BIRC6	0.228961666	9.19690408	15.04393397	0.0002726	0.0010634
**ENSG00000255526**	NEDD8-MDP1	0.448833359	5.586558424	15.04357659	0.0002726	0.0010634
**ENSG00000115053**	NCL	0.295673894	9.152377753	15.0378808	0.0002733	0.0010656
**ENSG00000198496**	NBR2	-0.907050777	3.658866249	15.03556927	0.0002735	0.0010662
**ENSG00000151846**	PABPC3	0.650721775	5.11070203	15.34792405	0.0002738	0.0010669
**ENSG00000198771**	RCSD1	0.268399288	7.397703138	15.02475965	0.0002748	0.0010703
**ENSG00000171853**	TRAPPC12	-0.409746334	5.751259694	15.01947279	0.0002754	0.0010722
**ENSG00000129595**	EPB41L4A	-0.983667404	3.892585966	15.23105698	0.0002759	0.0010739
**ENSG00000081665**	ZNF506	-0.28774077	7.228664644	15.00876426	0.0002766	0.0010760
**ENSG00000227097**		0.952396558	4.876627187	15.91052627	0.0002767	0.0010760
**ENSG00000247596**	TWF2	0.489033299	5.042310755	14.9991397	0.0002777	0.0010798
**ENSG00000111817**	DSE	0.428326938	5.519442049	14.99217256	0.0002786	0.0010826
**ENSG00000145214**	DGKQ	-0.732571759	4.136811498	14.99016063	0.0002788	0.0010831
**ENSG00000178802**	MPI	-0.349187476	6.102074424	14.97980165	0.0002800	0.0010870
**ENSG00000165678**	GHITM	0.364824999	6.55303829	14.97967931	0.0002800	0.0010870
**ENSG00000178464**		0.672396956	6.74610316	16.09923386	0.0002811	0.0010907
**ENSG00000168159**	RNF187	0.55781443	4.657000924	14.96534933	0.0002817	0.0010928
**ENSG00000108771**	DHX58	-0.831715956	3.93944043	14.96414406	0.0002818	0.0010929
**ENSG00000136040**	PLXNC1	0.437165014	6.056434086	14.96156385	0.0002822	0.0010939
**ENSG00000107679**	PLEKHA1	-0.329227447	7.110198653	14.95769534	0.0002826	0.0010951

**ENSG00000150977**	rilpl2	0.63396351	4.549495446	14.94944216	0.0002836	0.0010985
**ENSG00000182544**	MFSD5	0.831430764	3.529218488	14.94770657	0.0002838	0.0010989
**ENSG00000048707**	VPS13D	0.238895303	8.283071969	14.93917167	0.0002848	0.0011024
**ENSG00000159753**	CARMIL2	-0.456933633	6.137345601	15.02394024	0.0002860	0.0011066
**ENSG00000079691**	CARMIL1	-0.682351404	4.764599805	14.94765827	0.0002866	0.0011085
**ENSG00000196975**	ANXA4	0.735440137	3.988041231	14.920042	0.0002871	0.0011101
**ENSG00000275307**		-0.608389051	4.435230433	14.91888026	0.0002873	0.0011102
**ENSG00000197162**	ZNF785	-0.757167232	4.116810983	14.91636135	0.0002876	0.0011110
**ENSG00000165983**	PTER	0.424477072	5.378114728	14.90311638	0.0002892	0.0011168
**ENSG00000159720**	ATP6V0D1	0.552259495	4.726618501	14.90134412	0.0002894	0.0011172
**ENSG00000135951**	TSGA10	-0.674573971	4.31913025	14.90006565	0.0002895	0.0011174
**ENSG00000277301**	LOC101929698	-1.051211834	3.618037473	15.00865479	0.0002916	0.0011249
**ENSG00000118363**	SPCS2	0.492139116	5.086294193	14.87760872	0.0002923	0.0011271
**ENSG00000164104**	HMGB2	0.40609621	6.242287683	14.87639358	0.0002924	0.0011273
**ENSG00000119669**	IRF2BPL	0.560806334	4.922857223	14.8645558	0.0002939	0.0011325
**ENSG00000106052**	TAX1BP1	0.297280897	7.501144551	14.86097512	0.0002943	0.0011338
**ENSG00000133316**	WDR74	1.10018534	7.827126098	16.01442779	0.0002965	0.0011416
**ENSG00000143119**	CD53	0.254181151	8.294268651	14.83988637	0.0002970	0.0011431
**ENSG00000170144**	MIR4444-1	-0.230426029	8.932805058	14.8379256	0.0002972	0.0011436
**ENSG00000125731**	SH2D3A	-0.577324065	4.753991319	14.83664562	0.0002974	0.0011438
**ENSG00000146476**	ARMT1	0.521244725	4.695264803	14.82226896	0.0002992	0.0011499
**ENSG00000228502**		0.900061108	5.495956561	15.92339881	0.0002992	0.0011499
**ENSG00000213865**	C8orf44	-0.737806409	3.892539086	14.80602732	0.0003012	0.0011571
**ENSG00000267855**	NDUFA7	-0.781966881	3.434053359	14.80572319	0.0003013	0.0011571
**ENSG00000236472**		0.669120612	3.926390295	14.80426344	0.0003014	0.0011574
**ENSG00000169976**	SF3B5	0.576452485	4.383596249	14.794925	0.0003026	0.0011615
**ENSG00000205707**	ETFRF1	-0.492635593	5.128349367	14.79079795	0.0003032	0.0011631
**ENSG00000103510**	kat8	-0.422072652	5.480292994	14.78837622	0.0003035	0.0011638
**ENSG00000046647**	gemin8	-0.839641283	3.518098191	14.78362983	0.0003041	0.0011657
**ENSG00000176225**	RTTN	-0.426468722	5.946057306	14.77323692	0.0003054	0.0011704
**ENSG00000228327**		-0.684654203	3.885504998	14.76574755	0.0003064	0.0011737
**ENSG00000164691**	TAGAP	-0.277355551	8.486190331	14.7436018	0.0003093	0.0011843
**ENSG00000084676**	NCOA1	0.274197494	7.170283945	14.73662943	0.0003102	0.0011873
**ENSG00000254721**		0.701532743	3.775832836	14.72679554	0.0003115	0.0011918
**ENSG00000197989**	Snhg12	1.123520438	6.14910129	15.8667563	0.0003129	0.0011968
**ENSG00000166822**	TMEM170A	0.364629354	6.245651581	14.71049591	0.0003136	0.0011992
**ENSG00000103769**	RAB11A	0.313722516	6.51616209	14.69508476	0.0003156	0.0012065

**ENSG00000163013**	FBXO41	-0.631475101	4.071706142	14.6791638	0.0003178	0.0012142
**ENSG00000106605**	BLVRA	0.695164852	3.529123313	14.6714081	0.0003188	0.0012178
**ENSG00000273015**		-0.472171854	5.318654024	14.66963139	0.0003191	0.0012182
**ENSG00000168778**	TCTN2	-0.863817285	3.584838129	14.66432456	0.0003198	0.0012205
**ENSG00000163131**	CTSS	0.357775661	6.867562433	14.65712893	0.0003207	0.0012238
**ENSG00000044446**	PHKA2	-0.501278687	5.37229955	14.65415103	0.0003211	0.0012249
**ENSG00000131263**	RLIM	0.294365979	7.145351984	14.63241764	0.0003241	0.0012357
**ENSG00000272917**		-0.558187997	4.994850025	14.62168575	0.0003256	0.0012409
**ENSG00000198718**	FAM179B	-0.545076675	4.822878268	14.61846383	0.0003260	0.0012418
**ENSG00000178209**	PLEC	0.418153052	8.831103273	15.31534819	0.0003261	0.0012418
**ENSG00000198612**	COPS8	-0.402261147	5.558990042	14.61568482	0.0003264	0.0012427
**ENSG00000172936**	MYD88	0.414164528	6.032339562	14.61023611	0.0003272	0.0012451
**ENSG00000272909**		-0.862156987	3.473170349	14.60427053	0.0003280	0.0012478
**ENSG00000198700**	IPO9	-0.354429189	6.789809174	14.59824522	0.0003288	0.0012505
**ENSG00000214199**		0.79392746	4.092358132	14.63450541	0.0003317	0.0012608
**ENSG00000221792**	MIR1282	0.577871407	4.962115127	14.57716901	0.0003318	0.0012608
**ENSG00000215769**	LOC146880	-0.965033081	4.819218277	15.42593708	0.0003324	0.0012626
**ENSG00000168310**	IRF2	0.358938561	6.218985652	14.57190161	0.0003325	0.0012627
**ENSG00000135185**	TMEM243	-0.309060285	6.105698248	14.57104348	0.0003326	0.0012627
**ENSG00000165169**	DYNLT3	0.476449057	5.263352811	14.56206714	0.0003339	0.0012670
**ENSG00000101972**	STAG2	0.282290658	8.405924484	14.56021194	0.0003341	0.0012675
**ENSG00000263528**	IKBKE	-0.450233784	5.580327779	14.5431661	0.0003366	0.0012762
**ENSG00000173726**	TOMM20	0.308155575	6.649032345	14.5343215	0.0003378	0.0012806
**ENSG00000281195**		-0.732106076	4.74650951	14.6536379	0.0003391	0.0012848
**ENSG00000005700**	IBTK	0.304359799	6.61684723	14.51644379	0.0003404	0.0012893
**ENSG00000280202**		0.565457587	4.538729571	14.50871323	0.0003415	0.0012931
**ENSG00000157353**	FUK	-0.68500983	3.986246912	14.50169338	0.0003425	0.0012965
**ENSG00000227191**		0.778464114	4.726601436	14.89396885	0.0003427	0.0012968
**ENSG00000236552**	RPL13AP5	0.668640575	7.885073117	15.60730168	0.0003432	0.0012982
**ENSG00000109572**	CLCN3	0.34671367	6.34276392	14.48359042	0.0003452	0.0013050
**ENSG00000115977**	AAK1	-0.247078374	9.029380982	14.47967091	0.0003457	0.0013067
**ENSG00000167613**	LAIR1	-0.462418657	5.674168257	14.47719561	0.0003461	0.0013076
**ENSG00000114107**	CEP70	-0.737451309	4.219937032	14.47442908	0.0003465	0.0013087
**ENSG00000168887**	c2orf68	-0.413265584	5.849263915	14.46925445	0.0003473	0.0013111
**ENSG00000186866**	POFUT2	-0.757783132	4.26807137	14.45654931	0.0003491	0.0013177
**ENSG00000179715**	PCED1B	-0.307419535	6.712040915	14.42816686	0.0003534	0.0013331
**ENSG00000136156**	ITM2B	0.280332538	9.221872931	14.4136283	0.0003555	0.0013409

**ENSG00000132589**	FLOT2	0.329985975	6.054750628	14.41155139	0.0003559	0.0013416
**ENSG00000266019**	MIR3609	0.850792454	6.921350934	15.51081524	0.0003565	0.0013434
**ENSG00000117335**	CD46	0.205942096	7.91367567	14.40667445	0.0003566	0.0013434
**ENSG00000167578**	RAB4B	-0.790806953	4.091577465	14.40290558	0.0003572	0.0013447
**ENSG00000271725**		-0.442476938	5.272901128	14.4027515	0.0003572	0.0013447
**ENSG00000124357**	NAGK	-0.512527527	5.460233691	14.38937503	0.0003592	0.0013518
**ENSG00000232354**		-0.646901299	4.32213092	14.37758049	0.0003610	0.0013581
**ENSG00000147164**	SNX12	0.593822953	4.215192843	14.36885145	0.0003624	0.0013627
**ENSG00000111229**	ARPC3	0.301583037	7.067014467	14.36003083	0.0003637	0.0013673
**ENSG00000269888**		0.771457833	7.585023715	15.44974219	0.0003647	0.0013705
**ENSG00000108375**	RNF43	-0.418802517	5.360080968	14.3527909	0.0003648	0.0013705
**ENSG00000234851**		0.774391349	7.665266634	15.44562268	0.0003653	0.0013718
**ENSG00000187688**	TRPV2	0.383997639	5.790636575	14.34682049	0.0003658	0.0013726
**ENSG00000198553**	KCNRG	0.687601427	3.924113494	14.34663333	0.0003658	0.0013726
**ENSG00000110344**	UBE4A	-0.343646642	7.388305033	14.43124601	0.0003661	0.0013733
**ENSG00000100983**	GSS	-0.798854553	3.69004115	14.3411539	0.0003666	0.0013748
**ENSG00000155229**	MMS19	-0.434269121	6.400294601	14.46289324	0.0003694	0.0013845
**ENSG00000203485**	INF2	-0.519738223	5.575710592	14.35342129	0.0003706	0.0013888
**ENSG00000111145**	ELK3	0.311039761	7.055155446	14.31122597	0.0003713	0.0013909
**ENSG00000181090**	EHMT1	-0.30879805	6.463664599	14.30835954	0.0003718	0.0013921
**ENSG00000127445**	PIN1	-0.65237908	4.338932017	14.29502679	0.0003739	0.0013995
**ENSG00000175322**	znf519	-0.683646516	4.420435326	14.28590008	0.0003754	0.0014045
**ENSG00000115806**	GORASP2	0.314201267	6.090949265	14.28149459	0.0003761	0.0014059
**ENSG00000124224**		-0.743447822	4.375971957	14.28105383	0.0003761	0.0014059
**ENSG00000116213**	WRAP73	-0.663162482	4.424323281	14.28092682	0.0003761	0.0014059
**ENSG00000283375**		0.730614086	3.364092742	14.27710002	0.0003768	0.0014077
**ENSG00000111647**	UHRF1BP1L	0.389391625	5.405722363	14.27231649	0.0003775	0.0014101
**ENSG00000167461**	RAB8A	0.353782095	5.819161152	14.26738702	0.0003783	0.0014125
**ENSG00000145029**	NICN1	-0.885599491	3.7297132	14.26292316	0.0003790	0.0014147
**ENSG00000079616**	KIF22	-0.494556353	5.40107278	14.25954403	0.0003796	0.0014162
**ENSG00000144445**	KANSL1L	-0.461631874	5.833729209	14.25720408	0.0003800	0.0014170
**ENSG00000078668**	VDAC3	-0.375223307	5.583952843	14.25661262	0.0003801	0.0014170
**ENSG00000070831**	CDC42	0.301142493	7.951518057	14.25461618	0.0003804	0.0014177
**ENSG00000161265**	U2AF1L4	-0.804513228	3.79881359	14.24759512	0.0003815	0.0014214
**ENSG00000008128**	CDK11A	-0.467918837	5.846450401	14.25508225	0.0003824	0.0014242
**ENSG00000197776**	KLHDC1	-0.654822722	3.952486097	14.22760476	0.0003848	0.0014325
**ENSG00000185650**	ZFP36L1	0.268222333	8.286561448	14.22373189	0.0003854	0.0014344

**ENSG00000050405**	LIMA1	0.511286118	5.222243713	14.22189662	0.0003857	0.0014350
**ENSG00000189067**	LITAF	0.383900797	7.287502348	14.53167667	0.0003863	0.0014366
**ENSG00000157212**	PAXIP1	-0.404343349	5.561055226	14.20855529	0.0003879	0.0014416
**ENSG00000187257**	RSBN1L	0.313258381	6.807741942	14.20849235	0.0003879	0.0014416
**ENSG00000179532**	DNHD1	-0.718938014	6.282055358	15.19604448	0.0003892	0.0014460
**ENSG00000198242**	RPL23A	0.473802243	9.550722349	15.0864361	0.0003901	0.0014486
**ENSG00000113407**	TARS	0.494530366	5.834962497	14.3703549	0.0003905	0.0014496
**ENSG00000162434**	JAK1	0.231856463	9.068258942	14.1842975	0.0003919	0.0014545
**ENSG00000110321**	EIF4G2	0.213956453	9.507658841	14.18255644	0.0003922	0.0014551
**ENSG00000101342**	TLDC2	-0.32648507	6.275746854	14.1814531	0.0003924	0.0014552
**ENSG00000141644**	MBD1	-0.410321658	6.166311762	14.16570135	0.0003950	0.0014645
**ENSG00000130770**	ATPIF1	-0.425744271	5.404956587	14.15822871	0.0003963	0.0014686
**ENSG00000262413**		0.566336199	5.008286386	14.1437122	0.0003988	0.0014772
**ENSG00000224861**		0.902414398	3.331756931	14.12601859	0.0004018	0.0014879
**ENSG00000182183**	FAM159A	-0.513641767	5.329994614	14.10624748	0.0004052	0.0014999
**ENSG00000168028**	RPSA	0.349957848	8.77889459	14.42178949	0.0004068	0.0015055
**ENSG00000081307**	UBA5	-0.452607762	5.701025086	14.0885211	0.0004083	0.0015102
**ENSG00000132763**	MMACHC	0.56231165	4.916488308	14.08269305	0.0004093	0.0015135
**ENSG00000106415**	GLCCI1	0.363621901	6.346856143	14.06278618	0.0004128	0.0015258
**ENSG00000202354**	rny3	1.661999773	5.238466885	15.11473622	0.0004134	0.0015275
**ENSG00000133256**	PDE6B	-0.829492052	3.537261677	14.05267964	0.0004145	0.0015313
**ENSG00000185917**	SETD4	-0.58772081	4.697231918	14.04500023	0.0004159	0.0015358
**ENSG00000214331**	LOC283922	-0.601600856	4.480517329	14.04024874	0.0004167	0.0015384
**ENSG00000275835**	TUBGCP5	-0.440790886	5.393606104	14.03175476	0.0004183	0.0015434
**ENSG00000184863**	RBM33	-0.227959919	7.91686978	14.02541774	0.0004194	0.0015471
**ENSG00000168924**	LETM1	0.461092429	4.941932925	14.01948054	0.0004205	0.0015505
**ENSG00000143106**	PSMA5	0.381346807	5.744832451	14.00805773	0.0004225	0.0015573
**ENSG00000113732**	ATP6V0E1	0.535635525	5.403692941	14.0927117	0.0004226	0.0015573
**ENSG00000279530**		-0.716819268	4.245446447	14.00471824	0.0004231	0.0015586
**ENSG00000090989**	EXOC1	-0.34711809	6.069931545	14.00250235	0.0004235	0.0015595
**ENSG00000172315**	TP53RK	0.485808368	5.11644655	14.00063456	0.0004239	0.0015602
**ENSG00000101191**	DIDO1	-0.263881223	7.540644057	13.99271128	0.0004253	0.0015650
**ENSG00000112855**	HARS2	-0.552797026	5.084087963	13.98436505	0.0004268	0.0015700
**ENSG00000197381**	ADARB1	0.322303029	5.974062424	13.9690331	0.0004296	0.0015798
**ENSG00000138449**	SLC40A1	-0.453462144	6.130472252	14.13494971	0.0004298	0.0015798
**ENSG00000182220**	ATP6AP2	0.328302001	6.109364887	13.96671995	0.0004300	0.0015798
**ENSG00000102921**	N4BP1	0.326860787	6.619684947	13.96651269	0.0004301	0.0015798

**ENSG00000183172**	SMDT1	0.582655834	5.032694204	13.9628897	0.0004307	0.0015817
**ENSG00000039650**	PNKP	-0.756756792	5.215437786	14.54449279	0.0004311	0.0015823
**ENSG00000126012**	KDM5C	-0.303036698	6.928156328	13.95893338	0.0004315	0.0015831
**ENSG00000159884**	CCDC107	0.674923213	3.566160396	13.95838745	0.0004316	0.0015831
**ENSG00000007541**	PIGQ	-0.753175923	3.634157017	13.95616084	0.0004320	0.0015840
**ENSG00000168591**	TMUB2	-0.750699998	4.45204056	13.97591876	0.0004321	0.0015840
**ENSG00000244005**	NFS1	-0.644169806	4.307742371	13.94845829	0.0004334	0.0015881
**ENSG00000164961**	KIAA0196	0.297512972	6.141359124	13.90241504	0.0004420	0.0016191
**ENSG00000069329**	VPS35	0.25609208	7.040902897	13.89269206	0.0004439	0.0016253
**ENSG00000261716**		-0.619292398	4.448658637	13.87394258	0.0004474	0.0016378
**ENSG00000122257**	RBBP6	-0.306132208	7.590790284	13.87092858	0.0004480	0.0016394
**ENSG00000185624**	P4HB	0.337559333	6.424460384	13.86784168	0.0004486	0.0016410
**ENSG00000125814**	NAPB	-0.582093823	4.563490455	13.85242348	0.0004516	0.0016508
**ENSG00000114933**	INO80D	0.259928513	7.679201256	13.85223669	0.0004516	0.0016508
**ENSG00000198805**	PNP	0.519009967	5.501686232	13.9331757	0.0004521	0.0016520
**ENSG00000140931**	CMTM3	0.440457016	5.252144161	13.84339676	0.0004533	0.0016554
**ENSG00000009694**	TENM1	-0.542108672	5.869625009	14.1896473	0.0004533	0.0016554
**ENSG00000187790**	fancm	-0.562598079	4.649468071	13.8366475	0.0004546	0.0016596
**ENSG00000166169**	POLL	-0.632215512	4.436525422	13.82905853	0.0004561	0.0016644
**ENSG00000175054**	ATR	-0.299026827	7.066443819	13.82128541	0.0004576	0.0016693
**ENSG00000271913**		-0.342889033	6.62789854	13.81682854	0.0004585	0.0016720
**ENSG00000273637**		0.489831047	4.493794834	13.81395376	0.0004591	0.0016734
**ENSG00000138496**	PARP9	-0.476747445	6.418321476	14.20386686	0.0004595	0.0016743
**ENSG00000113504**	SLC12A7	-0.615300738	5.297209014	13.97182943	0.0004606	0.0016771
**ENSG00000181524**		0.848182509	4.050180422	13.97477801	0.0004606	0.0016771
**ENSG00000113387**	SUB1	0.251214107	7.667305771	13.79797683	0.0004622	0.0016826
**ENSG00000188690**	UROS	-0.639665972	4.119778183	13.78891993	0.0004640	0.0016885
**ENSG00000197744**		0.786608036	3.867600053	13.78275417	0.0004653	0.0016924
**ENSG00000154822**	MIR3714	0.249651328	6.843760332	13.7738818	0.0004670	0.0016983
**ENSG00000022267**	FHL1	0.534665256	4.785930067	13.77197725	0.0004674	0.0016991
**ENSG00000134369**	NAV1	-0.706351604	4.082614065	13.77014054	0.0004678	0.0016998
**ENSG00000067141**	NEO1	0.518458704	4.660931987	13.76725082	0.0004684	0.0017013
**ENSG00000267207**		0.804854436	3.515072292	13.76279094	0.0004693	0.0017040
**ENSG00000267278**	MAP3K14-AS1	-0.651833893	4.141658376	13.74504199	0.0004729	0.0017164
**ENSG00000131469**	RPL27	0.628530507	9.207398539	14.74246272	0.0004735	0.0017181
**ENSG00000200312**		1.546610299	4.418909105	14.74213337	0.0004738	0.0017188
**ENSG00000204435**	CSNK2B	0.320706565	5.795542786	13.73908652	0.0004741	0.0017190

**ENSG00000168040**	FADD	0.656236318	3.943746772	13.73718806	0.0004745	0.0017198
**ENSG00000122068**	FYTTD1	0.329298235	6.606823679	13.73351815	0.0004752	0.0017219
**ENSG00000231767**		0.916507302	5.214113252	14.66088818	0.0004764	0.0017258
**ENSG00000205531**	NAP1L4	-0.227662691	7.687254922	13.71207126	0.0004796	0.0017367
**ENSG00000205189**	ZBTB10	-0.409174131	5.553277646	13.70641891	0.0004808	0.0017403
**ENSG00000177485**	ZBTB33	0.396272494	6.538167168	13.7867872	0.0004823	0.0017453
**ENSG00000125257**	ABCC4	0.546048493	4.589854571	13.69277341	0.0004836	0.0017486
**ENSG00000271853**		-0.700208499	3.774462367	13.6926299	0.0004836	0.0017486
**ENSG00000106351**	AGFG2	0.487067435	5.28575245	13.69211125	0.0004837	0.0017486
**ENSG00000241973**	PI4KA	-0.282377933	7.4255179	13.68721036	0.0004847	0.0017517
**ENSG00000185896**	LAMP1	0.311629936	6.634519191	13.67865435	0.0004865	0.0017575
**ENSG00000243696**		-0.722027288	4.623281275	13.77315483	0.0004874	0.0017602
**ENSG00000198205**	ZXDA	0.516436373	4.369348015	13.66807515	0.0004887	0.0017643
**ENSG00000163389**	POGLUT1	-0.515767804	5.336492225	13.66674862	0.0004890	0.0017647
**ENSG00000203814**	HIST2H2BF	0.9109664	4.569106341	14.28250983	0.0004892	0.0017647
**ENSG00000133835**	HSD17B4	-0.391414153	5.52161046	13.66312439	0.0004898	0.0017654
**ENSG00000207357**	RNU6-2	1.579644823	6.936635559	14.66237035	0.0004899	0.0017654
**ENSG00000168395**	ING5	-0.391943631	5.578138704	13.66258999	0.0004899	0.0017654
**ENSG00000184983**	NDUFA6	0.531169453	4.582852387	13.65971489	0.0004905	0.0017670
**ENSG00000164754**	MIR3610	0.226594288	8.41105211	13.65568987	0.0004913	0.0017694
**ENSG00000224032**		-0.600652953	4.525698232	13.65386256	0.0004917	0.0017702
**ENSG00000187653**		0.674851145	6.593025021	14.5696482	0.0004988	0.0017950
**ENSG00000204227**	ring1	-0.551602951	4.163810468	13.61132911	0.0005008	0.0018016
**ENSG00000138613**	APH1B	-0.502610613	4.630912134	13.60988069	0.0005011	0.0018021
**ENSG00000270580**	PKD1P6-NPIPP1	-0.933922291	3.9085239	13.72114039	0.0005014	0.0018025
**ENSG00000149357**	LAMTOR1	0.461419269	4.629677232	13.59993227	0.0005033	0.0018086
**ENSG00000139725**	RHOF	0.281472409	6.916771859	13.59795649	0.0005037	0.0018092
**ENSG00000139697**	MIR8072	0.237006682	7.877532449	13.59753958	0.0005038	0.0018092
**ENSG00000265972**	TXNIP	0.256618996	11.18975487	13.59526359	0.0005043	0.0018104
**ENSG00000100605**	ITPK1	0.540632524	4.538379246	13.57771744	0.0005081	0.0018235
**ENSG00000169598**	DFFB	-0.792952045	3.856642078	13.56972802	0.0005099	0.0018291
**ENSG00000160961**	ZNF333	-0.668068486	4.552221862	13.55986228	0.0005120	0.0018363
**ENSG00000243554**		-0.80556728	4.014675631	13.556251	0.0005128	0.0018385
**ENSG00000270800**	RPS10-NUDT3	0.418714822	8.798195334	14.23825012	0.0005149	0.0018453
**ENSG00000113658**	SMAD5	0.4708899	5.382005869	13.54149386	0.0005161	0.0018489
**ENSG00000099904**	ZDHHC8	-0.861763096	4.229610425	13.7211743	0.0005169	0.0018514
**ENSG00000173915**	MIR1307	0.619381884	4.919643971	13.62352766	0.0005172	0.0018518

**ENSG00000105443**	CYTH2	-0.521902307	5.000966747	13.53385163	0.0005178	0.0018531
**ENSG00000107362**	ABHD17B	0.570041103	4.488436636	13.5303122	0.0005186	0.0018553
**ENSG00000211747**		0.548561937	4.735516562	13.52727618	0.0005193	0.0018571
**ENSG00000081087**	OSTM1	0.362899871	5.766364984	13.51617385	0.0005217	0.0018654
**ENSG00000138002**	IFT172	-0.743978593	4.136963804	13.51408667	0.0005222	0.0018664
**ENSG00000163564**	PYHIN1	0.393085106	6.79204184	13.69303043	0.0005228	0.0018680
**ENSG00000204310**	MIR6721	0.481117229	4.57204779	13.49336543	0.0005269	0.0018819
**ENSG00000105875**	WDR91	-0.613629405	4.540779804	13.49244296	0.0005271	0.0018820
**ENSG00000163877**	SNIP1	0.425244501	5.125609135	13.49152102	0.0005273	0.0018821
**ENSG00000185201**	IFITM2	0.427884033	7.874303588	14.17420573	0.0005283	0.0018846
**ENSG00000197296**	FITM2	-0.643781619	3.819805526	13.48684992	0.0005284	0.0018846
**ENSG00000023171**	GRAMD1B	0.678197669	4.21783278	13.48270416	0.0005293	0.0018873
**ENSG00000164081**	TEX264	0.531460333	4.43331303	13.48108662	0.0005297	0.0018880
**ENSG00000174456**	C12orf76	-0.495978769	4.953661045	13.47570394	0.0005309	0.0018917
**ENSG00000189195**	KIAA1107	-0.732930469	3.906878625	13.46834053	0.0005326	0.0018971
**ENSG00000112996**	MRPS30	0.284773071	6.173533845	13.46760454	0.0005328	0.0018971
**ENSG00000158195**	WASF2	0.237083383	7.68948409	13.43982512	0.0005392	0.0019193
**ENSG00000049883**	PTCD2	-0.616746125	4.4994568	13.43881363	0.0005394	0.0019194
**ENSG00000230897**		0.944120045	4.420468597	14.05361637	0.0005467	0.0019445
**ENSG00000115137**	DNAJC27	-0.727015269	4.037124705	13.40539202	0.0005473	0.0019460
**ENSG00000155926**	SLA	0.273539904	7.394016638	13.40270576	0.0005479	0.0019476
**ENSG00000149577**	SIDT2	-0.468975106	5.339981319	13.39971956	0.0005486	0.0019495
**ENSG00000207507**	RNU6-9	1.560656748	6.930539799	14.35271335	0.0005508	0.0019564
**ENSG00000243789**	JMJD7	-0.816919934	4.342169027	13.53281018	0.0005511	0.0019568
**ENSG00000163867**	ZMYM6	-0.445783541	6.32598391	13.62080365	0.0005514	0.0019575
**ENSG00000188322**	SBK1	0.603856028	4.993274814	13.49793764	0.0005517	0.0019578
**ENSG00000233762**		0.6971438	6.137417826	14.27018373	0.0005531	0.0019620
**ENSG00000105127**	AKAP8	-0.458407833	5.736340752	13.37958637	0.0005534	0.0019625
**ENSG00000013275**	PSMC4	0.422481999	4.950514634	13.3719034	0.0005552	0.0019683
**ENSG00000076555**	ACACB	-0.484273688	5.084221034	13.34689805	0.0005613	0.0019890
**ENSG00000223959**	AFG3L1P	-0.655378818	4.669364192	13.33612255	0.0005639	0.0019976
**ENSG00000213658**	LAT	-0.36481476	6.441519233	13.33064776	0.0005652	0.0020017
**ENSG00000268030**		-0.759587994	3.75396026	13.32752556	0.0005660	0.0020037
**ENSG00000185305**	ARL 15.00	0.552296919	4.447007534	13.32230965	0.0005673	0.0020075
**ENSG00000088986**	DYNLL1	0.581603648	5.125040502	13.43134346	0.0005674	0.0020075
**ENSG00000148303**	RPL7A	0.288124941	9.326603844	13.31268677	0.0005696	0.0020146
**ENSG00000134987**	WDR36	0.324422422	6.587193568	13.30426858	0.0005717	0.0020212

**ENSG00000183401**	CCDC159	-0.87402029	3.507117281	13.30188535	0.0005723	0.0020226
**ENSG00000165406**	39508	0.369075535	5.958243186	13.30085993	0.0005726	0.0020228
**ENSG00000165704**	HPRT1	0.686069686	4.172710096	13.28676055	0.0005761	0.0020345
**ENSG00000013375**	PGM3	-0.3841358	5.620075228	13.27186921	0.0005798	0.0020470
**ENSG00000124596**	OARD1	-0.372123744	5.508267044	13.26897273	0.0005805	0.0020485
**ENSG00000141012**	GALNS	-0.736379144	4.408188625	13.26859923	0.0005806	0.0020485
**ENSG00000267080**	ASB16-AS1	-0.709058551	4.051962326	13.25313377	0.0005845	0.0020616
**ENSG00000105939**	ZC3HAV1	0.209376529	9.074864226	13.25040914	0.0005852	0.0020633
**ENSG00000103550**	KNOP1	-0.554779117	4.964463473	13.24100079	0.0005876	0.0020710
**ENSG00000225031**		0.721405888	3.603088156	13.22547451	0.0005915	0.0020843
**ENSG00000178741**	Cox5a	0.78570959	4.620095119	13.62297084	0.0005927	0.0020877
**ENSG00000109320**	NFKB1	0.248662965	7.275537461	13.20547017	0.0005967	0.0021010
**ENSG00000080345**	RIF1	0.237895842	8.043329475	13.19613385	0.0005991	0.0021088
**ENSG00000263244**		0.262422083	7.886958706	13.194479	0.0005995	0.0021096
**ENSG00000104885**	DOT1L	-0.493178885	5.111226293	13.17999527	0.0006033	0.0021222
**ENSG00000177963**	MIR6743	0.379915318	5.28676272	13.15973053	0.0006086	0.0021402
**ENSG00000160654**	CD3G	0.307604963	8.365049144	13.24699949	0.0006111	0.0021482
**ENSG00000126882**	FAM78A	0.244933929	7.031181953	13.14699268	0.0006120	0.0021506
**ENSG00000253980**		-1.100785622	3.809368659	13.51265348	0.0006160	0.0021632
**ENSG00000123607**	TTC21B	-0.355371805	6.098888587	13.13193807	0.0006160	0.0021632
**ENSG00000185485**	SDHAP1	-0.610469936	4.392077704	13.12697262	0.0006173	0.0021672
**ENSG00000089159**	PXN	0.309261125	7.073361599	13.122533	0.0006185	0.0021706
**ENSG00000140382**	HMG20A	-0.33919385	5.86526853	13.12124953	0.0006189	0.0021711
**ENSG00000171155**	C1GALT1C1	0.616609318	4.091837531	13.11305158	0.0006211	0.0021781
**ENSG00000168255**	POLR2J3	-0.458619954	5.672884035	13.10858191	0.0006223	0.0021816
**ENSG00000139517**	LNX2	0.435817507	4.892794443	13.09153611	0.0006269	0.0021971
**ENSG00000283199**		-0.693753016	5.068053325	13.44651149	0.0006285	0.0022014
**ENSG00000232176**		0.706630257	6.288739477	13.96845853	0.0006286	0.0022014
**ENSG00000278311**	GGNBP2	-0.252471962	7.166740949	13.08241492	0.0006294	0.0022029
**ENSG00000198162**	MAN1A2	0.303057267	7.571489062	13.08229437	0.0006294	0.0022029
**ENSG00000114439**	bbx	0.275534999	7.650341387	13.06077758	0.0006353	0.0022229
**ENSG00000226029**	LOC107984921	-0.611358685	4.558343437	13.05099498	0.0006380	0.0022316
**ENSG00000145604**	SKP2	-0.529003068	4.605892533	13.04159011	0.0006407	0.0022400
**ENSG00000274422**		-1.123957146	4.395946005	13.72933296	0.0006437	0.0022499
**ENSG00000163703**	creld1	-0.625517096	4.104687957	13.02651403	0.0006449	0.0022532
**ENSG00000130038**	CRACR2A	0.408762935	5.702786789	13.02506574	0.0006453	0.0022539
**ENSG00000283103**		-0.582838006	4.72503842	13.01140273	0.0006491	0.0022665

**ENSG00000173226**	IQCB1	-0.509297331	5.159895286	12.98803326	0.0006558	0.0022889
**ENSG00000140545**	MFGE8	-0.692151555	4.606054973	13.02562817	0.0006571	0.0022922
**ENSG00000123268**	ATF1	0.440695365	5.6023731	12.98319476	0.0006571	0.0022922
**ENSG00000198373**	WWP2	0.285634134	6.128585902	12.98096908	0.0006578	0.0022937
**ENSG00000202252**	Snord14c	1.233565943	4.483735739	13.83080754	0.0006602	0.0023006
**ENSG00000140563**	MCTP2	0.500775081	5.271723443	12.97251425	0.0006602	0.0023006
**ENSG00000249249**		0.582324346	3.830571632	12.96995722	0.0006609	0.0023024
**ENSG00000108825**	PTGES3L-AARS	-0.778853444	3.443569796	12.96393742	0.0006627	0.0023076
**ENSG00000246451**		-0.684304034	4.301421193	12.9627981	0.0006630	0.0023080
**ENSG00000257390**		0.379144256	5.552330297	12.96148156	0.0006634	0.0023084
**ENSG00000204469**	PRRC2A	0.247862711	7.553268593	12.96087617	0.0006635	0.0023084
**ENSG00000024048**	UBR2	0.229337195	7.649001332	12.95797166	0.0006644	0.0023106
**ENSG00000261553**		-0.487875091	4.700827353	12.95194025	0.0006661	0.0023159
**ENSG00000262831**		0.507894001	4.753316469	12.94978125	0.0006668	0.0023173
**ENSG00000168566**	SNRNP48	-0.431605876	5.52865798	12.94553884	0.0006680	0.0023208
**ENSG00000113712**	CSNK1A1	0.214493334	7.924359452	12.94288681	0.0006688	0.0023227
**ENSG00000122705**	CLTA	0.513685021	4.977579389	12.9406681	0.0006694	0.0023240
**ENSG00000111325**	OGFOD2	-0.722847507	3.708335058	12.93950132	0.0006698	0.0023240
**ENSG00000184730**	APOBR	0.581310814	4.516842064	12.9393296	0.0006698	0.0023240
**ENSG00000254170**		1.118457204	3.900288847	13.51776067	0.0006702	0.0023247
**ENSG00000123575**	FAM199X	0.313249725	6.25490269	12.93029832	0.0006724	0.0023316
**ENSG00000202198**	rn7sk	0.885667255	9.918761262	13.82839964	0.0006729	0.0023318
**ENSG00000283293**		0.885667255	9.918761262	13.82839964	0.0006729	0.0023318
**ENSG00000232593**		-0.703584032	4.870315777	13.18138178	0.0006753	0.0023392
**ENSG00000196110**	ZNF699	0.527660328	4.451718082	12.90534904	0.0006798	0.0023539
**ENSG00000116741**	RGS2	0.731370387	3.852115849	12.89786691	0.0006820	0.0023608
**ENSG00000011007**	TCEB3	0.328580677	5.79342699	12.89382946	0.0006832	0.0023642
**ENSG00000154803**	FLCN	-0.491217432	5.217916897	12.88316232	0.0006864	0.0023744
**ENSG00000008226**	DLEC1	-0.629656688	4.744507523	12.89876129	0.0006870	0.0023758
**ENSG00000272540**		0.484307704	5.844587572	13.07179345	0.0006890	0.0023818
**ENSG00000130844**	ZNF331	-0.45099784	5.178962159	12.87102532	0.0006900	0.0023847
**ENSG00000123739**	PLA2G12A	-0.492195173	4.975655224	12.87021467	0.0006903	0.0023847
**ENSG00000183255**	PTTG1IP	0.33376829	5.929310058	12.85015678	0.0006963	0.0024049
**ENSG00000138078**	PREPL	-0.247645927	6.599163264	12.84062333	0.0006992	0.0024141
**ENSG00000273275**		0.467922985	5.122213451	12.83574685	0.0007007	0.0024184
**ENSG00000099326**	MZF1	-0.803790744	4.951007426	13.37805687	0.0007023	0.0024230
**ENSG00000133561**	GIMAP6	0.287356211	7.301170515	12.82926416	0.0007027	0.0024237

**ENSG00000049860**	HEXB	0.434037423	5.081923391	12.82097473	0.0007053	0.0024317
**ENSG00000067082**	KLF6	0.357829653	8.584661703	13.22065223	0.0007061	0.0024339
**ENSG00000261799**		0.377394111	5.443987161	12.81346521	0.0007076	0.0024380
**ENSG00000226084**		0.624019874	4.900113315	12.9683136	0.0007079	0.0024384
**ENSG00000128915**	ice2	-0.305886456	6.774343818	12.81132003	0.0007082	0.0024387
**ENSG00000205339**	IPO7	0.274073083	7.331309557	12.80937743	0.0007088	0.0024400
**ENSG00000204231**	RXRB	-0.568508618	5.039529574	12.82193756	0.0007104	0.0024445
**ENSG00000102967**	DHODH	-0.763417032	3.936773787	12.77629929	0.0007191	0.0024738
**ENSG00000187097**	ENTPD5	-0.512076439	5.013853366	12.76380396	0.0007231	0.0024866
**ENSG00000102974**	CTCF	0.297302345	6.725335464	12.76105108	0.0007239	0.0024887
**ENSG00000143756**	FBXO28	0.356745253	5.966875936	12.75936293	0.0007245	0.0024897
**ENSG00000072135**	PTPN18	0.341567001	6.168695966	12.75739709	0.0007251	0.0024908
**ENSG00000149582**	TMEM25	-0.761229178	3.481988896	12.75687318	0.0007253	0.0024908
**ENSG00000213190**	MLLT11	-0.46629033	5.363105464	12.75351738	0.0007263	0.0024937
**ENSG00000158156**	XKR8	0.729256762	3.591862961	12.74731493	0.0007283	0.0024996
**ENSG00000185669**	SNAI3	0.707360938	3.594171863	12.74381705	0.0007294	0.0025026
**ENSG00000119977**	TCTN3	0.496817244	4.603122433	12.73779614	0.0007313	0.0025084
**ENSG00000105705**	SUGP1	-0.509788992	4.657082122	12.7336493	0.0007327	0.0025118
**ENSG00000182670**	TTC3	-0.256376778	8.704526075	12.73315759	0.0007328	0.0025118
**ENSG00000161091**	MFSD12	-0.515660951	4.9613264	12.73156826	0.0007333	0.0025127
**ENSG00000254901**	BORCS8	-0.648322685	4.28274966	12.73066541	0.0007336	0.0025129
**ENSG00000131389**	SLC6A6	0.352251526	5.969419	12.71762422	0.0007378	0.0025264
**ENSG00000102871**	TRADD	0.476348515	4.962022939	12.7076595	0.0007410	0.0025366
**ENSG00000213741**	LOC100288910	0.714050432	8.148088518	13.57268146	0.0007424	0.0025400
**ENSG00000063046**	EIF4B	0.24752836	9.520732282	12.70313071	0.0007425	0.0025400
**ENSG00000155666**	KDM8	-0.739528665	3.694979928	12.684363	0.0007486	0.0025601
**ENSG00000259661**		-0.898801789	3.613174754	12.66962168	0.0007535	0.0025758
**ENSG00000125351**	UPF3B	-0.551776153	4.820230381	12.66037416	0.0007565	0.0025854
**ENSG00000135678**	CPM	0.615954199	3.930479228	12.65326578	0.0007589	0.0025926
**ENSG00000176049**	JAKMIP2	0.765670875	4.253041283	12.73525837	0.0007637	0.0026083
**ENSG00000180957**	PITPNB	0.282504061	6.243167779	12.63385874	0.0007654	0.0026130
**ENSG00000100442**	FKBP3	-0.379415283	5.370667764	12.61949606	0.0007702	0.0026279
**ENSG00000280828**		-0.449673456	5.417430454	12.61941648	0.0007702	0.0026279
**ENSG00000198492**	YTHDF2	0.274119582	6.14553985	12.60752468	0.0007742	0.0026408
**ENSG00000230076**		0.751237778	5.50957924	13.33948698	0.0007759	0.0026454
**ENSG00000160216**	AGPAT3	0.287495181	6.104248849	12.59964567	0.0007769	0.0026482
**ENSG00000145416**	36951	-0.607245171	4.420756994	12.59873718	0.0007772	0.0026484

**ENSG00000055955**	ITIH4	-0.838368653	4.360331894	12.84747173	0.0007775	0.0026485
**ENSG00000108055**	SMC3	0.296464477	7.544457813	12.59386304	0.0007789	0.0026523
**ENSG00000175395**	ZNF25	-0.545776701	4.71503642	12.59114024	0.0007798	0.0026546
**ENSG00000157540**	DYRK1A	0.192858669	7.939246503	12.58626703	0.0007815	0.0026594
**ENSG00000111880**	RNGTT	0.373917933	5.781525508	12.57974722	0.0007837	0.0026661
**ENSG00000116017**	ARID3A	0.846844292	3.503159854	12.57092291	0.0007868	0.0026756
**ENSG00000162976**	PQLC3	0.487222627	4.992236937	12.56599659	0.0007885	0.0026805
**ENSG00000181381**	DDX60L	-0.358061477	7.322439469	12.77635485	0.0007919	0.0026910
**ENSG00000164022**	AIMP1	-0.407641413	5.783149734	12.55544899	0.0007921	0.0026910
**ENSG00000164091**	WDR82	0.225175692	8.15302634	12.55445305	0.0007925	0.0026910
**ENSG00000121892**	PDS5A	0.218113517	8.388739559	12.55414617	0.0007926	0.0026910
**ENSG00000132323**	ILKAP	-0.449137511	5.298365176	12.54936043	0.0007942	0.0026958
**ENSG00000084112**	SSH1	0.383951204	5.679716691	12.53789633	0.0007982	0.0027085
**ENSG00000261067**		-0.361089999	6.447614021	12.53690085	0.0007986	0.0027088
**ENSG00000141429**	GALNT1	0.435845028	5.542703246	12.53470191	0.0007994	0.0027105
**ENSG00000178567**	EPM2AIP1	-0.315299245	6.678755602	12.52592334	0.0008024	0.0027201
**ENSG00000090863**	GLG1	0.204898958	8.813555679	12.52291609	0.0008035	0.0027228
**ENSG00000267100**	ILF3-AS1	-0.61564943	4.208058156	12.52202206	0.0008038	0.0027229
**ENSG00000204308**	RNF5	0.76826844	3.427742347	12.52137154	0.0008040	0.0027229
**ENSG00000273729**		-0.802994526	3.593950556	12.51750688	0.0008054	0.0027266
**ENSG00000270589**		-0.693725913	4.096885866	12.50734244	0.0008090	0.0027379
**ENSG00000112763**	btn2a1	-0.434292741	5.67408289	12.49860917	0.0008121	0.0027475
**ENSG00000005483**	KMT2E	0.207710151	9.121514525	12.49503572	0.0008134	0.0027510
**ENSG00000008130**	NADK	-0.609205774	4.349531845	12.49091333	0.0008149	0.0027550
**ENSG00000171843**	MLLT3	-0.336181376	6.795973859	12.47771482	0.0008196	0.0027700
**ENSG00000162298**	MIR6751	0.368408918	7.058967829	12.69912137	0.0008198	0.0027700
**ENSG00000237818**		1.066120562	4.328876579	13.13756449	0.0008207	0.0027719
**ENSG00000129559**	NEDD8	0.417587405	5.865150474	12.4657139	0.0008239	0.0027821
**ENSG00000136682**	CBWD2	-0.507253576	4.55579332	12.46053643	0.0008258	0.0027875
**ENSG00000089157**	RPLP0	0.341109287	9.02828921	12.7727383	0.0008275	0.0027910
**ENSG00000260853**		-0.744557801	4.537214617	12.6328689	0.0008275	0.0027910
**ENSG00000135968**	GCC2	-0.3213656	8.345597021	12.64583051	0.0008276	0.0027910
**ENSG00000107758**	PPP3CB	0.249987829	6.676004377	12.44744587	0.0008306	0.0028000
**ENSG00000148337**	CIZ1	-0.332947637	6.165036691	12.439979	0.0008333	0.0028083
**ENSG00000115159**	GPD2	0.442581461	5.051502725	12.43791867	0.0008341	0.0028099
**ENSG00000115207**	GTF3C2	-0.355489654	5.73609589	12.41075582	0.0008441	0.0028428
**ENSG00000176915**	ANKLE2	-0.281266208	6.908041267	12.39664946	0.0008493	0.0028595

**ENSG00000229344**		0.971729102	5.185917312	13.18938058	0.0008540	0.0028743
**ENSG00000167113**	COQ4	-0.729736617	4.437503857	12.43650908	0.0008582	0.0028876
**ENSG00000176018**	LYSMD3	0.400529312	6.099906001	12.40977599	0.0008617	0.0028984
**ENSG00000198728**	LDB1	-0.377747708	6.211731294	12.35279802	0.0008659	0.0029115
**ENSG00000109519**	GRPEL1	0.452277129	4.885454112	12.3513171	0.0008664	0.0029125
**ENSG00000140675**	SLC5A2	-0.600519623	4.373072057	12.34977231	0.0008670	0.0029135
**ENSG00000163939**	PBRM1	0.23161112	7.423788464	12.33780281	0.0008716	0.0029272
**ENSG00000104687**	GSR	0.374449168	5.708779725	12.33764342	0.0008717	0.0029272
**ENSG00000123728**	RAP2C	0.392906285	5.701354958	12.33617112	0.0008722	0.0029282
**ENSG00000144566**	RAB5A	0.319931061	6.003116843	12.33517462	0.0008726	0.0029285
**ENSG00000164715**	LMTK2	0.296160258	5.968790513	12.32218842	0.0008776	0.0029444
**ENSG00000036257**	CUL3	0.265256624	7.347239265	12.31478822	0.0008805	0.0029530
**ENSG00000100429**	HDAC10	-0.833304764	4.386976798	12.56425552	0.0008817	0.0029563
**ENSG00000111361**	EIF2B1	-0.404204091	5.638306294	12.30610442	0.0008839	0.0029625
**ENSG00000126561**	STAT5A	-0.302962361	6.23157019	12.29731983	0.0008873	0.0029726
**ENSG00000110218**	PANX1	0.660501093	3.746077485	12.29647675	0.0008876	0.0029726
**ENSG00000185504**	FAAP100	-0.719666649	4.286214911	12.30630295	0.0008878	0.0029726
**ENSG00000151176**	PLBD2	0.464725455	5.719161587	12.40239177	0.0008880	0.0029726
**ENSG00000205981**	DNAJC19	-0.487944512	4.860635538	12.28861434	0.0008907	0.0029806
**ENSG00000149527**	PLCH2	-0.877410081	3.982959221	12.34373365	0.0008913	0.0029817
**ENSG00000167965**	MLST8	-0.69192316	3.796317862	12.28523405	0.0008920	0.0029831
**ENSG00000166704**	ZNF606	-0.670035685	4.266184868	12.28001308	0.0008941	0.0029890
**ENSG00000125695**		-0.380038112	5.284495082	12.27873875	0.0008946	0.0029898
**ENSG00000197771**	MCMBP	0.27615712	6.367523789	12.27793579	0.0008949	0.0029899
**ENSG00000149716**	ORAOV1	-0.622397284	4.628028697	12.26930535	0.0008983	0.0029999
**ENSG00000156802**	ATAD2	0.456728911	5.997641095	12.46641506	0.0008985	0.0029999
**ENSG00000157193**	LRP8	0.507867157	4.561249126	12.26535053	0.0008999	0.0030036
**ENSG00000101574**	METTL4	-0.455946269	5.221280158	12.26402825	0.0009004	0.0030044
**ENSG00000119689**	DLST	-0.349217302	6.107561515	12.26117607	0.0009015	0.0030072
**ENSG00000111481**	COPZ1	0.400669289	5.382010619	12.25789916	0.0009028	0.0030106
**ENSG00000140497**	SCAMP2	0.329816477	6.320632207	12.24812836	0.0009067	0.0030227
**ENSG00000169490**	TM2D2	0.537072484	4.267839831	12.24022428	0.0009099	0.0030323
**ENSG00000099219**	ERMP1	0.339877477	5.847005565	12.23854311	0.0009106	0.0030335
**ENSG00000125734**	MIR6791	0.589788955	4.642949864	12.23614386	0.0009115	0.0030358
**ENSG00000101365**	IDH3B	-0.493584518	4.592434726	12.2308673	0.0009137	0.0030419
**ENSG00000162819**	BROX	0.27893211	6.81770688	12.22156105	0.0009174	0.0030534
**ENSG00000136003**	ISCU	-0.342187715	5.937083428	12.21986072	0.0009181	0.0030547

**ENSG00000275764**		-0.848636956	3.394581171	12.21856331	0.000918	0.003055
**ENSG00000100911**	MIR7703	-0.430410671	5.525188421	12.20319027	0.000924	0.003075
**ENSG00000188130**	MAPK12	-0.823821239	4.303039928	12.40900561	0.000925	0.003075
**ENSG00000148396**	SEC16A	0.238088196	7.462474049	12.19722409	0.000927	0.003081
**ENSG00000107614**	TRDMT1	-0.506652431	5.493527264	12.28599003	0.000928	0.003082
**ENSG00000143633**	C1orf131	-0.469054038	4.571387415	12.19443873	0.000928	0.003083
**ENSG00000164054**	SHISA5	0.361823752	6.751667202	12.28440567	0.000930	0.003090
**ENSG00000087470**	DNM1L	-0.275653137	6.5936943	12.17599539	0.000936	0.003105
**ENSG00000197045**	GMFB	0.350442327	5.98147927	12.17569504	0.000936	0.003105
**ENSG00000100823**	APEX1	-0.306710269	6.131417358	12.17566593	0.000936	0.003105
**ENSG00000166188**	ZNF319	0.600195997	4.444990255	12.17246164	0.000937	0.003108
**ENSG00000169895**	SYAP1	0.40602661	5.62345703	12.15808446	0.000943	0.003127
**ENSG00000134815**	DHX34	-0.658147234	4.155272477	12.15542608	0.000944	0.003130
**ENSG00000155729**	KCTD18	-0.568818972	4.655910653	12.15303271	0.000945	0.003131
**ENSG00000179909**	ZNF154	-0.703707843	4.339142798	12.15298384	0.000945	0.003131
**ENSG00000146757**	ZNF92	0.386947391	5.916010532	12.15155884	0.000946	0.003132
**ENSG00000243406**	MRPS31P5	-0.54710283	4.539821795	12.14938201	0.000947	0.003134
**ENSG00000213024**	NUP62	0.356646835	6.056905666	12.14093308	0.000950	0.003145
**ENSG00000140943**	MBTPS1	-0.246738971	7.018408397	12.13889383	0.000951	0.003146
**ENSG00000162604**	TM2D1	-0.425989989	5.432951318	12.13745195	0.000952	0.003147
**ENSG00000165416**	SUGT1	0.263296447	6.889879173	12.13366592	0.000953	0.003152
**ENSG00000035141**	FAM136A	-0.584187067	4.329904787	12.12943891	0.000955	0.003157
**ENSG00000204713**	TRIM27	-0.301293568	5.873314382	12.12569311	0.000957	0.003161
**ENSG00000137492**	THAP12	0.268426223	6.483115391	12.11840573	0.000960	0.003170
**ENSG00000265148**	TSPOAP1-AS1	-0.331406265	6.117028027	12.11374624	0.000962	0.003176
**ENSG00000141527**	CARD14	-0.942657066	3.6591184	12.22226926	0.000969	0.003197
**ENSG00000090924**	PLEKHG2	-0.530223749	5.421892523	12.20184095	0.000972	0.003208
**ENSG00000172785**	CBWD1	-0.692868216	4.020590536	12.08591584	0.000974	0.003212
**ENSG00000182903**	znf721	-0.354156252	6.985229043	12.20988578	0.000976	0.003220
**ENSG00000087157**	PGS1	-0.473531908	5.087232425	12.0756448	0.000978	0.003225
**ENSG00000156232**	WHAMM	-0.368887857	5.966087319	12.06304105	0.000984	0.003242
**ENSG00000275740**	RBM27	0.298873057	6.523062919	12.05443458	0.000987	0.003253
**ENSG00000134152**	KATNBL1	-0.423634558	5.201345542	12.0518791	0.000988	0.003255
**ENSG00000167286**	CD3D	0.358980217	6.983902413	12.22329623	0.000989	0.003255
**ENSG00000171475**	WIPF2	0.317297652	5.868741337	12.04920134	0.000990	0.003256
**ENSG00000179271**	GADD45GIP1	0.521975517	4.720185827	12.04914647	0.000990	0.003256
**ENSG00000114573**	ATP6V1A	0.427153534	5.313527944	12.04851369	0.000990	0.003256

**ENSG00000176124**	DLEU1	0.668644307	4.900243928	12.27939852	0.0009917	0.0032602
**ENSG00000185875**	THNSL1	-0.686616122	3.685031758	12.04163086	0.0009934	0.0032646
**ENSG00000153936**	HS2ST1	0.453762303	5.443743616	12.03474725	0.0009964	0.0032736
**ENSG00000088448**	ANKRD10	-0.338286064	6.57586101	12.02528271	0.0010006	0.0032856
**ENSG00000163328**	GPR155	-0.374892041	6.666062921	12.1160655	0.0010007	0.0032856
**ENSG00000157881**	PANK4	-0.494154128	4.861497284	12.00375635	0.0010102	0.0033157
**ENSG00000148690**	FRA10AC1	-0.430851946	5.856537652	12.03109959	0.0010122	0.0033213
**ENSG00000144559**	TAMM41	-0.495679716	5.174224714	11.99661443	0.0010134	0.0033241
**ENSG00000103995**	CEP152	-0.566339306	4.693602448	11.99054539	0.0010161	0.0033320
**ENSG00000136628**	EPRS	0.266675394	7.1912145	11.98683145	0.0010178	0.0033365
**ENSG00000259112**	NDUFC2-KCTD1	0.763408782	3.687443517	11.98542871	0.0010184	0.0033375
**ENSG00000101247**	NDUFAF5	-0.681012249	4.230266698	11.98411169	0.0010190	0.0033377
**ENSG00000260170**		0.520511381	4.414530401	11.98390994	0.0010191	0.0033377
**ENSG00000185298**	CCDC137	-0.622720067	4.019206542	11.98028719	0.0010207	0.0033420
**ENSG00000185905**	C16orf54	0.309266628	7.416864655	12.02892044	0.0010271	0.0033618
**ENSG00000159921**	GNE	0.385228102	5.345091686	11.96213703	0.0010290	0.0033669
**ENSG00000255733**	IFNG-AS1	0.735756515	3.9536274	11.96055663	0.0010297	0.0033682
**ENSG00000165661**	QSOX2	-0.593674786	4.191417298	11.95846351	0.0010307	0.0033703
**ENSG00000189308**	LIN54	0.32480056	5.840911534	11.95487941	0.0010323	0.0033746
**ENSG00000155393**	HEATR3	-0.520926954	4.800683544	11.94792625	0.0010355	0.0033839
**ENSG00000177917**	ARL6IP6	0.480901371	4.79745116	11.94405649	0.0010373	0.0033881
**ENSG00000092871**	RFFL	0.251882794	7.005225483	11.94375152	0.0010374	0.0033881
**ENSG00000165802**	MIR7114	-0.626707542	4.50410046	11.93160623	0.0010430	0.0034053
**ENSG00000159461**	AMFR	0.369719459	5.757460369	11.93031416	0.0010436	0.0034062
**ENSG00000103199**	ZNF500	-0.691131679	3.932170109	11.92567767	0.0010458	0.0034122
**ENSG00000131584**	acap3	-0.779237441	4.394133396	12.10863273	0.0010495	0.0034232
**ENSG00000180233**	ZNRF2	0.393045464	5.476837735	11.91697994	0.0010498	0.0034232
**ENSG00000185101**	ANO9	-0.89940082	5.636524215	12.66333988	0.0010502	0.0034233
**ENSG00000113068**	PFDN1	0.474252426	4.494411391	11.91285764	0.0010517	0.0034274
**ENSG00000163602**	RYBP	0.363803943	6.009452828	11.89724099	0.0010591	0.0034501
**ENSG00000079819**	EPB41L2	-0.593138428	3.991980864	11.89002335	0.0010625	0.0034601
**ENSG00000157837**	SPPL3	0.348153076	5.489029786	11.88398939	0.0010653	0.0034683
**ENSG00000105339**	DENND3	0.674908322	4.476256667	11.94159545	0.0010660	0.0034696
**ENSG00000102218**	RP2	0.608657519	3.913620503	11.87999605	0.0010672	0.0034723
**ENSG00000204371**	EHMT2	-0.40769198	5.384000388	11.86448647	0.0010746	0.0034952
**ENSG00000090861**	AARS	0.423382825	5.143347691	11.84932911	0.0010818	0.0035178
**ENSG00000173933**	RBM4	-0.297401513	6.420021563	11.8475674	0.0010827	0.0035194

**ENSG00000223551**		0.944888851	4.24884528	12.30621435	0.0010883	0.0035366
**ENSG00000112592**	TBP	-0.438071008	5.127704308	11.83401657	0.0010892	0.0035385
**ENSG00000135686**	KLHL36	-0.283901862	6.722091122	11.82892376	0.0010917	0.0035454
**ENSG00000153187**	HNRNPU	-0.206619145	9.964953459	11.82667827	0.0010928	0.0035470
**ENSG00000104872**	PIH1D1	-0.662180146	4.544455682	11.88488361	0.0010929	0.0035470
**ENSG00000164219**	PGGT1B	-0.366304564	6.055511067	11.82214641	0.0010950	0.0035528
**ENSG00000172175**	MALT1	-0.260084046	7.869676317	11.82073092	0.0010957	0.0035539
**ENSG00000106692**	fktn	-0.439907999	4.941882235	11.81855285	0.0010967	0.0035557
**ENSG00000063978**	RNF4	0.260978443	6.758752555	11.81821998	0.0010969	0.0035557
**ENSG00000170275**	CRTAP	0.359271618	5.823718198	11.81700187	0.0010975	0.0035565
**ENSG00000171817**	ZNF540	-0.618537562	4.392670433	11.81612464	0.0010979	0.0035568
**ENSG00000284360**		-0.65673093	3.794635893	11.81236117	0.0010998	0.0035617
**ENSG00000149313**	AASDHPPT	0.351710605	5.760420889	11.8048047	0.0011035	0.0035726
**ENSG00000257027**		-0.458158453	6.476517485	12.16146561	0.0011075	0.0035844
**ENSG00000090581**	GNPTG	-0.457765235	4.770839457	11.79423219	0.0011087	0.0035872
**ENSG00000169682**	SPNS1	-0.696266574	4.008359208	11.79224183	0.0011097	0.0035892
**ENSG00000125484**	GTF3C4	0.371383543	5.273587128	11.78999765	0.0011108	0.0035908
**ENSG00000162971**	TYW5	-0.470388693	5.326484108	11.78988328	0.0011108	0.0035908
**ENSG00000149970**	CNKSR2	-0.94102592	4.134131924	12.13846374	0.0011158	0.0036057
**ENSG00000187713**	TMEM203	0.586805262	4.124799659	11.77920508	0.0011161	0.0036057
**ENSG00000153774**	CFDP1	0.508929267	4.886962196	11.77783623	0.0011168	0.0036068
**ENSG00000167085**	PHB	0.504408768	4.728266375	11.7746262	0.0011184	0.0036108
**ENSG00000127603**	MACF1	0.245991287	10.26875501	11.77326746	0.0011191	0.0036119
**ENSG00000261556**	SMG1P7	-0.548413769	4.586004458	11.77225802	0.0011196	0.0036124
**ENSG00000283498**		0.57475721	4.202159869	11.76900923	0.0011212	0.0036165
**ENSG00000111371**	SLC38A1	0.18552359	9.173092205	11.75408487	0.0011287	0.0036395
**ENSG00000147251**	DOCK11	0.204335145	8.82538374	11.74202003	0.0011347	0.0036579
**ENSG00000011052**	NME1-NME2	0.500163159	4.996488321	11.73800265	0.0011368	0.0036634
**ENSG00000257337**	LOC283335	0.258611861	7.08260338	11.73237345	0.0011396	0.0036714
**ENSG00000088876**	ZNF343	-0.714667703	3.662604612	11.7297997	0.0011409	0.0036744
**ENSG00000178381**	ZFAND2A	-0.713026328	3.461220456	11.72916153	0.0011413	0.0036744
**ENSG00000107863**	ARHGAP21	0.402107002	5.611449967	11.72226188	0.0011448	0.0036846
**ENSG00000160613**	pcsk7	-0.477636082	8.176722743	12.41080212	0.0011460	0.0036874
**ENSG00000163714**	U2SURP	-0.22069654	8.056059616	11.71871751	0.0011466	0.0036882
**ENSG00000256664**		0.517831761	4.201507185	11.71328159	0.0011494	0.0036960
**ENSG00000077254**	USP33	-0.245455033	7.063324095	11.70756343	0.0011523	0.0037043
**ENSG00000217576**		-0.661100653	4.132585851	11.7063133	0.0011529	0.0037052

**ENSG00000135469**	COQ10A	-0.751642236	3.661331082	11.70492898	0.0011537	0.0037063
**ENSG00000114388**	NPRL2	-0.596965212	4.594167049	11.68806432	0.0011624	0.0037332
**ENSG00000258424**		0.283816471	6.358267955	11.68720708	0.0011628	0.0037334
**ENSG00000148153**	INIP	0.430267503	5.129558877	11.67222103	0.0011706	0.0037573
**ENSG00000218227**		0.752165345	5.747776979	12.37641125	0.0011729	0.0037633
**ENSG00000075415**	SLC25A3	0.299176605	7.039618076	11.66730074	0.0011732	0.0037633
**ENSG00000111196**	MAGOHB	-0.642955213	4.485062784	11.66604354	0.0011738	0.0037642
**ENSG00000260304**		-0.389271554	5.494440327	11.66198442	0.0011760	0.0037699
**ENSG00000213347**	MXD3	0.402304953	4.884429974	11.65558744	0.0011793	0.0037795
**ENSG00000110048**	OSBP	0.300815951	6.215899831	11.6409402	0.0011871	0.0038031
**ENSG00000058063**	ATP11B	0.205995384	8.265159087	11.63878876	0.0011882	0.0038056
**ENSG00000105717**	PBX4	-0.78296727	4.041230248	11.65452317	0.0011934	0.0038210
**ENSG00000115241**	PPM1G	0.338583302	5.768961136	11.62350145	0.0011963	0.0038293
**ENSG00000246334**		-0.59509831	4.467062784	11.6198566	0.0011983	0.0038344
**ENSG00000203879**	GDI1	-0.325570025	6.342474408	11.6166379	0.0012000	0.0038387
**ENSG00000102245**	CD40LG	0.332902165	7.451249151	11.81680289	0.0012015	0.0038412
**ENSG00000123338**	NCKAP1L	0.199602328	7.978460355	11.61379568	0.0012015	0.0038412
**ENSG00000156136**	DCK	0.315231936	6.552159205	11.61075592	0.0012032	0.0038447
**ENSG00000110395**	CBL	0.222902887	7.606924452	11.61042738	0.0012033	0.0038447
**ENSG00000152291**	TGOLN2	-0.197370149	8.509519118	11.60471253	0.0012064	0.0038533
**ENSG00000169087**	HSPBAP1	-0.520765782	4.931927794	11.5999285	0.0012090	0.0038604
**ENSG00000136161**	RCBTB2	-0.572235511	4.587703144	11.59720408	0.0012105	0.0038639
**ENSG00000122482**	ZNF644	0.261331043	7.426535488	11.58181442	0.0012188	0.0038892
**ENSG00000145191**	EIF2B5	-0.356562812	5.643580603	11.58121258	0.0012191	0.0038892
**ENSG00000196313**	pom121	-0.279331453	6.791677709	11.58046867	0.0012195	0.0038893
**ENSG00000146282**	RARS2	-0.354642071	5.750989563	11.57733213	0.0012213	0.0038936
**ENSG00000215472**	RPL17-C18orf32	0.413061782	9.099540708	12.14550004	0.0012301	0.0039205
**ENSG00000151136**	BTBD11	0.488184584	4.940665662	11.55833848	0.0012317	0.0039244
**ENSG00000163444**	TMEM183A	-0.439795816	5.129676486	11.55080923	0.0012358	0.0039364
**ENSG00000211716**		0.744436494	3.394007463	11.54930495	0.0012366	0.0039377
**ENSG00000136059**	VILL	-0.547385171	4.530082868	11.54875324	0.0012370	0.0039377
**ENSG00000185811**	IKZF1	0.191357216	9.562905155	11.54374816	0.0012397	0.0039453
**ENSG00000180817**	PPA1	0.350550371	6.107295413	11.54196222	0.0012407	0.0039472
**ENSG00000269242**		0.505334025	5.804062687	11.8474735	0.0012423	0.0039510
**ENSG00000173692**	PSMD1	0.307785249	5.832942835	11.53787427	0.0012430	0.0039511
**ENSG00000113194**	FAF2	0.331333375	5.741496874	11.53775232	0.0012431	0.0039511
**ENSG00000160959**	LRRC14	-0.486039291	4.663769495	11.53527896	0.0012444	0.0039542

**ENSG00000073584**	SMARCE1	0.229624158	7.096318865	11.53397658	0.0012452	0.0039553
**ENSG00000107819**	SFXN3	0.502736787	5.180587684	11.5315843	0.0012466	0.0039583
**ENSG00000163161**	ERCC3	-0.408065456	5.633760916	11.53093702	0.0012468	0.0039583
**ENSG00000143614**	GATAD2B	0.201303414	7.545902734	11.52164838	0.0012520	0.0039726
**ENSG00000100376**	FAM118A	-0.635878579	5.716490151	12.02717652	0.0012521	0.0039726
**ENSG00000070770**	CSNK2A2	0.290088381	6.319394352	11.51803329	0.0012541	0.0039767
**ENSG00000076351**	SLC46A1	-0.479995722	4.889361578	11.51786873	0.0012542	0.0039767
**ENSG00000204237**	oxld1	-0.748597264	3.592923717	11.51368	0.0012565	0.0039829
**ENSG00000141027**	NCOR1	0.197766125	8.630667145	11.5121722	0.0012574	0.0039836
**ENSG00000214367**	HAUS3	-0.31754265	6.022082552	11.51196993	0.0012575	0.0039836
**ENSG00000111581**	NUP107	-0.339109464	6.013898054	11.50448488	0.0012617	0.0039957
**ENSG00000116649**	SRM	0.516170346	4.159250659	11.49020103	0.0012698	0.0040201
**ENSG00000015133**	CCDC88C	-0.233450954	8.268661874	11.48777072	0.0012712	0.0040233
**ENSG00000241878**	MIR7109	-0.613458764	4.602929067	11.48683895	0.0012717	0.0040237
**ENSG00000101350**	KIF3B	0.399377291	5.334072686	11.46914884	0.0012818	0.0040533
**ENSG00000116809**	ZBTB17	-0.617629047	4.014945156	11.46912417	0.0012818	0.0040533
**ENSG00000242802**	AP5Z1	-0.652035665	4.57854369	11.52145474	0.0012834	0.0040571
**ENSG00000136758**	YME1L1	0.215834421	7.427437191	11.45279123	0.0012912	0.0040806
**ENSG00000174606**	ANGEL2	-0.314616781	6.426309238	11.44421745	0.0012962	0.0040951
**ENSG00000196205**		0.510368712	8.120208713	12.13412253	0.0012971	0.0040968
**ENSG00000155850**	SLC26A2	0.356248543	5.508624504	11.43923084	0.0012991	0.0041018
**ENSG00000213799**	ZNF845	0.401203983	5.743991168	11.43730657	0.0013002	0.0041041
**ENSG00000116237**	ICMT	0.470494884	4.819166768	11.4334967	0.0013024	0.0041098
**ENSG00000166348**	USP54	-0.545663808	4.397369375	11.41348687	0.0013142	0.0041456
**ENSG00000132842**	AP3B1	0.275540058	6.610799248	11.40555007	0.0013189	0.0041591
**ENSG00000166508**	MCM7	-0.505878018	5.417684481	11.49075305	0.0013234	0.0041710
**ENSG00000185920**	PTCH1	0.435452754	5.270396829	11.39782772	0.0013234	0.0041710
**ENSG00000135766**	EGLN1	0.333826172	5.96235688	11.39177289	0.0013270	0.0041811
**ENSG00000120533**	ENY2	-0.370505365	5.399033196	11.38397993	0.0013317	0.0041945
**ENSG00000145365**	TIFA	0.461521397	5.009440979	11.38132265	0.0013333	0.0041970
**ENSG00000231925**	TAPBP	0.255136539	8.557256832	11.38129167	0.0013333	0.0041970
**ENSG00000253276**	CCDC71L	0.539301324	4.150096645	11.37716817	0.0013358	0.0042035
**ENSG00000051620**	HEBP2	-0.355713122	6.033154224	11.36467234	0.0013433	0.0042259
**ENSG00000163785**	RYK	-0.347538521	5.598191507	11.35234449	0.0013507	0.0042481
**ENSG00000080815**	PSEN1	0.294467896	6.0257573	11.35160171	0.0013512	0.0042482
**ENSG00000176928**	GCNT4	-0.493404043	6.075569096	11.67078644	0.0013518	0.0042490
**ENSG00000197008**	ZNF138	-0.468789939	5.119270466	11.34540503	0.0013549	0.0042575

**ENSG00000281103**	TRG-AS1	0.36241403	5.524060365	11.33456227	0.0013615	0.0042770
**ENSG00000112249**	ASCC3	0.254273119	7.348149072	11.3317317	0.0013633	0.0042811
**ENSG00000213203**	GIMAP1	-0.247456717	7.06237565	11.33002214	0.0013643	0.0042831
**ENSG00000123595**	RAB9A	0.612801954	3.806603818	11.32147618	0.0013696	0.0042983
**ENSG00000110429**	FBXO3	-0.300814728	6.93321666	11.3174617	0.0013720	0.0043048
**ENSG00000007168**	PAFAH1B1	0.191746851	7.719542721	11.31227013	0.0013752	0.0043135
**ENSG00000179029**	TMEM107	0.883007343	4.309192678	11.66341286	0.0013770	0.0043176
**ENSG00000100403**	ZC3H7B	-0.398386435	5.739186718	11.30438869	0.0013801	0.0043262
**ENSG00000175470**	PPP2R2D	-0.3896737	5.728717113	11.3035631	0.0013806	0.0043265
**ENSG00000130812**	ANGPTL6	-0.668073043	3.583302693	11.30232134	0.0013814	0.0043269
**ENSG00000271870**		-0.574470464	4.075569346	11.30205394	0.0013816	0.0043269
**ENSG00000228929**		0.974831366	4.245987699	11.82279068	0.0013838	0.0043325
**ENSG00000143951**	WDPCP	-0.752427717	4.027444756	11.29298164	0.0013872	0.004342
**ENSG00000125743**	SNRPD2	0.544992543	5.489502017	11.5653399	0.0013878	0.0043425
**ENSG00000212464**	SNORA12	1.498955584	4.192145205	11.97470336	0.0013896	0.0043468
**ENSG00000144136**	SLC20A1	0.305698783	6.703174234	11.2865842	0.0013912	0.0043505
**ENSG00000152926**	ZNF117	-0.610174941	4.723859616	11.34143899	0.0013935	0.0043564
**ENSG00000234857**	HNRNPUL2-BSC	0.278434394	7.156547405	11.27564124	0.0013981	0.0043694
**ENSG00000144711**	IQSEC1	0.243345448	7.121507893	11.25899372	0.0014086	0.0044009
**ENSG00000276057**		-0.748129004	3.731744851	11.25520302	0.0014110	0.0044071
**ENSG00000197903**	Hist1h2bk	0.822592561	5.357666736	11.90186798	0.0014119	0.0044087
**ENSG00000185219**	ZNF445	-0.302480321	6.344377758	11.24942087	0.0014146	0.0044141
**ENSG00000196700**	ZNF512B	-0.531878263	4.575720406	11.24928633	0.0014147	0.0044141
**ENSG00000023892**	DEF6	-0.282569537	6.697819799	11.24900285	0.0014149	0.0044141
**ENSG00000265681**	RPL17	0.405158159	9.069989223	11.79232159	0.0014204	0.0044299
**ENSG00000232220**		0.51366948	4.837929481	11.23567726	0.0014234	0.0044380
**ENSG00000196247**	ZNF107	-0.293668749	6.981582659	11.23420988	0.0014244	0.0044396
**ENSG00000129696**	TTI2	0.494816011	5.258285485	11.2681301	0.0014273	0.0044473
**ENSG00000158019**	BRE	0.429245724	4.850887086	11.22042469	0.0014332	0.0044646
**ENSG00000101367**	MAPRE1	0.309649555	6.462726551	11.21833844	0.0014346	0.0044674
**ENSG00000182372**	MIR3674	-0.66390919	4.381698985	11.20545751	0.0014429	0.0044910
**ENSG00000130522**	jund	0.46138333	6.537912005	11.62986498	0.0014430	0.0044910
**ENSG00000153989**	NUS1	0.341265874	5.770296103	11.20269118	0.0014447	0.0044950
**ENSG00000105771**	SMG9	-0.562473889	4.527652043	11.20180059	0.0014453	0.0044955
**ENSG00000134324**	LPIN1	-0.263142869	7.037469522	11.20008152	0.0014464	0.0044966
**ENSG00000115539**	PDCL3	0.533411308	4.302724069	11.19992828	0.0014465	0.0044966
**ENSG00000173482**	PTPRM	0.512498958	5.023115209	11.19380403	0.0014505	0.0045077

**ENSG00000213523**	SRA1	-0.493216941	4.887380973	11.18262277	0.0014578	0.0045291
**ENSG00000172725**	CORO1B	0.374683845	6.545861622	11.29490119	0.0014597	0.0045334
**ENSG00000197536**	C5orf56	-0.320348061	7.017159927	11.21366606	0.0014614	0.0045375
**ENSG00000207165**	Snora70	1.030566211	3.725166691	11.50044381	0.0014627	0.0045401
**ENSG00000151651**	ADAM8	0.441899086	5.478667245	11.1730416	0.0014641	0.0045433
**ENSG00000162775**	RBM15	0.313172236	6.071006824	11.17202548	0.0014648	0.0045440
**ENSG00000235944**		-0.556364591	4.526999166	11.16293732	0.0014708	0.0045613
**ENSG00000113163**	COL4A3BP	0.297439765	6.459382192	11.15090621	0.0014788	0.0045847
**ENSG00000144228**	SPOPL	0.424923714	5.430556787	11.14784936	0.0014808	0.0045897
**ENSG00000146007**	ZMAT2	0.385212471	5.629925483	11.14400436	0.0014834	0.0045963
**ENSG00000262246**	CORO7	-0.438831526	6.6131058	11.48293063	0.0014861	0.0046032
**ENSG00000088970**	KIZ	-0.409188586	5.216420517	11.13597148	0.0014888	0.0046098
**ENSG00000154889**	MPPE1	-0.493947197	4.829756119	11.13552677	0.0014891	0.0046098
**ENSG00000136146**	MED4	0.30404019	5.597350747	11.13285185	0.0014909	0.0046140
**ENSG00000188026**	RILPL1	-0.670985865	3.820728303	11.12960067	0.0014931	0.0046194
**ENSG00000198744**		0.809382867	6.184975952	11.8019795	0.0014942	0.0046214
**ENSG00000277791**	PSMB3	0.577082279	4.768051465	11.17565583	0.0014992	0.0046355
**ENSG00000091157**	WDR7	0.261035769	6.717767191	11.11831367	0.0015007	0.0046388
**ENSG00000164494**	PDSS2	0.506236551	4.063455778	11.10974047	0.0015065	0.0046554
**ENSG00000136938**	ANP32B	0.329996641	7.786719035	11.37086208	0.0015089	0.0046606
**ENSG00000188234**	AGAP4	-0.913331426	3.398284319	11.15656418	0.0015091	0.0046606
**ENSG00000147471**	PROSC	0.377496659	5.63712475	11.09977266	0.0015133	0.0046722
**ENSG00000225067**		0.990918541	4.74041249	11.7388273	0.0015137	0.0046722
**ENSG00000273025**	CELF6	-0.752124827	3.574747305	11.07987599	0.0015269	0.0047116
**ENSG00000144635**	DYNC1LI1	0.34107938	5.309029976	11.0755534	0.0015299	0.0047194
**ENSG00000124406**	ATP8A1	-0.370615856	6.732676109	11.22402803	0.0015353	0.0047346
**ENSG00000151093**	OXSM	-0.729089148	3.539365652	11.06551139	0.0015369	0.0047381
**ENSG00000196712**	NF1	0.235601631	7.318511884	11.06421921	0.0015378	0.0047394
**ENSG00000112294**	ALDH5A1	-0.450509085	4.781437401	11.04712233	0.0015497	0.0047747
**ENSG00000114127**	XRN1	0.207568313	8.370784702	11.04486638	0.0015513	0.0047782
**ENSG00000155380**	SLC16A1	0.494413646	4.561509189	11.03718918	0.0015567	0.0047934
**ENSG00000006025**	OSBPL7	-0.747258334	4.197467247	11.11084016	0.0015609	0.0048051
**ENSG00000111596**	CNOT2	-0.25555452	7.057521358	11.02725341	0.0015637	0.0048121
**ENSG00000104472**	CHRAC1	0.412985737	5.33024064	11.01516435	0.0015722	0.0048371
**ENSG00000049245**	VAMP3	0.394429032	5.376542302	11.00879861	0.0015768	0.0048496
**ENSG00000142856**	ITGB3BP	-0.479255234	4.904688968	11.00723737	0.0015779	0.0048516
**ENSG00000103248**	MTHFSD	-0.553671755	4.087835097	11.00173533	0.0015818	0.0048622

**ENSG00000075539**	FRYL	0.209113646	8.354472247	10.99825282	0.0015843	0.0048684
**ENSG00000074054**	CLASP1	0.214696624	7.058209375	10.99683191	0.0015853	0.0048701
**ENSG00000179832**	MROH1	-0.544812853	5.426998337	11.18174441	0.0015887	0.0048790
**ENSG00000236778**	INTS6-AS1	-0.629858287	3.8607338	10.991205	0.0015893	0.0048797
**ENSG00000168724**	DNAJC21	0.27757497	6.533485856	10.98904803	0.0015909	0.0048827
**ENSG00000018510**	AGPS	0.330728477	5.995830531	10.98852913	0.0015913	0.0048827
**ENSG00000136169**	SETDB2	-0.298842616	6.194546409	10.98627981	0.0015929	0.0048862
**ENSG00000188242**	PP7080	-0.679442258	4.28168859	10.97378663	0.0016019	0.0049118
**ENSG00000144283**	PKP4	0.417229918	5.196057203	10.97338374	0.0016022	0.0049118
**ENSG00000177885**	GRB2	0.269742002	6.519249032	10.9728119	0.0016026	0.0049118
**ENSG00000171793**	CTPS1	-0.492357871	4.49310729	10.96709782	0.0016068	0.0049230
**ENSG00000186532**	SMYD4	-0.41606372	4.983391841	10.96521928	0.0016081	0.0049258
**ENSG00000165282**	PIGO	-0.499064282	4.531702756	10.96140791	0.0016109	0.0049328
**ENSG00000134744**	ZCCHC11	-0.231403984	7.980346767	10.96048957	0.0016116	0.0049334
**ENSG00000110011**	DNAJC4	-0.716523696	3.755375549	10.95914597	0.0016126	0.0049350
**ENSG00000279119**		-0.467253577	5.057697068	10.95672828	0.0016143	0.0049390
**ENSG00000259316**	KIAA0101	0.471864987	4.463134304	10.95194327	0.0016178	0.0049482
**ENSG00000083097**	DOPEY1	-0.262233844	6.761422627	10.94545	0.0016226	0.0049613
**ENSG00000120254**	MTHFD1L	0.694112746	3.671324717	10.94232446	0.0016249	0.0049669
**ENSG00000134574**	ddb2	-0.564388811	5.381056277	11.17254783	0.0016260	0.0049688
**ENSG00000082074**	FYB	-0.204933144	10.2556779	10.94016395	0.0016265	0.0049689
**ENSG00000224078**		-0.288638783	7.513027153	10.96339715	0.0016273	0.0049698
**ENSG00000105821**	DNAJC2	-0.335552486	6.218987953	10.93753683	0.0016284	0.0049719
**ENSG00000108389**	MTMR4	-0.326149365	6.073743824	10.93601135	0.0016295	0.0049738
**ENSG00000173540**	GMPPB	-0.538946886	4.902057966	10.93177343	0.0016327	0.0049819
**ENSG00000214922**	HLA-F-AS1	-0.495253942	4.959434064	10.92528462	0.0016375	0.0049951
**ENSG00000149480**	MTA2	0.236015093	6.535019159	10.92275427	0.0016393	0.0049981
**ENSG00000235703**	LINC00894	-0.785962563	4.252956465	11.08164574	0.0016394	0.0049981
**ENSG00000198707**	CEP290	-0.343589472	6.577718162	10.94102242	0.0016420	0.0050047
**ENSG00000176903**	PNMA1	0.412158478	4.840610776	10.91452583	0.0016455	0.0050137
**ENSG00000151135**	TMEM263	-0.328789045	5.750994097	10.91204926	0.0016473	0.0050178
**ENSG00000013563**	DNASE1L1	-0.660525566	4.124048898	10.90276928	0.0016542	0.0050375
**ENSG00000178502**	KLHL11	0.440835717	4.469442569	10.90112115	0.0016555	0.0050398
**ENSG00000128928**	IVD	-0.497923843	4.621985949	10.90035537	0.0016561	0.0050401
**ENSG00000139154**	AEBP2	0.268176283	6.439808884	10.89692462	0.0016586	0.0050465
**ENSG00000147130**	ZMYM3	-0.373091058	5.359043495	10.8936351	0.0016611	0.0050525
**ENSG00000163964**	PIGX	0.522263387	4.14288665	10.89059939	0.0016634	0.0050580

**ENSG00000173762**	CD7	-0.472269655	5.385507483	10.92299442	0.0016723	0.0050836
**ENSG00000107897**	ACBD5	0.340689264	5.338378064	10.87697453	0.0016737	0.0050864
**ENSG00000086189**	DIMT1	0.23037829	6.884974973	10.87536633	0.0016749	0.0050886
**ENSG00000240567**	LOC101243545	0.675488454	6.871613013	11.5047018	0.0016848	0.0051167
**ENSG00000105835**	NAMPT	0.413957407	5.024761512	10.8619126	0.0016852	0.0051167
**ENSG00000232729**	LOC101926943	-0.461034421	5.115112773	10.85623717	0.0016895	0.0051284
**ENSG00000196150**	ZNF250	0.572841853	4.139511317	10.85542016	0.0016901	0.0051288
**ENSG00000040199**	PHLPP2	0.48468947	5.085288251	10.85402477	0.0016912	0.0051306
**ENSG00000137135**	ARHGEF39	0.568921974	3.932644716	10.85171015	0.0016930	0.0051345
**ENSG00000284135**		0.521401374	4.937749489	10.88090933	0.0016955	0.0051407
**ENSG00000077782**	FGFR1	0.611606828	4.053806571	10.81733819	0.0017196	0.0052121
**ENSG00000275131**		-0.522572347	5.647010428	11.05177897	0.0017202	0.0052126
**ENSG00000162636**	FAM102B	0.374460773	5.368207235	10.81407819	0.0017221	0.0052164
**ENSG00000269292**		0.40383446	5.122893218	10.81360887	0.0017225	0.0052164
**ENSG00000053254**	FOXN3	0.248342177	7.556573059	10.80216689	0.0017315	0.0052404
**ENSG00000163738**	MTHFD2L	-0.714171023	3.624462212	10.80192871	0.0017316	0.0052404
**ENSG00000146433**	TMEM181	0.341718228	6.488800678	10.80932348	0.0017319	0.0052404
**ENSG00000132153**	DHX30	-0.307409331	5.782796713	10.79962806	0.0017334	0.0052436
**ENSG00000100207**	tcf20	0.243946244	7.143285333	10.79718463	0.0017354	0.0052479
**ENSG00000105321**	CCDC9	-0.531244673	3.820352822	10.79361951	0.0017382	0.0052549
**ENSG00000071889**	FAM3A	-0.711268837	3.926054236	10.79083844	0.0017404	0.0052600
**ENSG00000196233**	C10orf12	0.235967149	7.497431542	10.77740203	0.0017510	0.0052906
**ENSG00000011114**	BTBD7	0.305864951	6.110502689	10.77016055	0.0017568	0.0053065
**ENSG00000182134**	TDRKH	0.488510457	4.442376625	10.75788296	0.0017666	0.0053347
**ENSG00000274012**	RN7SL2	0.99292243	10.00373655	11.37666773	0.0017783	0.0053685
**ENSG00000280046**		-0.654366488	4.067193188	10.7425272	0.0017790	0.0053689
**ENSG00000164305**	CASP3	0.382063711	5.343494182	10.74156776	0.0017798	0.0053697
**ENSG00000245552**	LOC101929295	-0.544112094	4.090746882	10.72806912	0.0017907	0.0054012
**ENSG00000113621**	TXNDC15	0.419459374	5.24634973	10.71977677	0.0017975	0.0054200
**ENSG00000204856**	FAM216A	-0.683819726	4.155535478	10.71712331	0.0017997	0.0054250
**ENSG00000157350**	ST3GAL2	-0.430532588	5.440954139	10.71535845	0.0018011	0.0054278
**ENSG00000120925**	MIR4469	-0.520253671	4.663099741	10.70800857	0.0018071	0.0054437
**ENSG00000210191**		-0.642974403	4.178423622	10.70764462	0.0018074	0.0054437
**ENSG00000211459**		0.710769298	14.10263101	11.3241278	0.0018114	0.0054528
**ENSG00000134802**	SLC43A3	0.623941857	3.668062531	10.70270634	0.0018115	0.0054528
**ENSG00000153898**	MCOLN2	0.777040792	4.436402983	10.93629091	0.0018152	0.0054613
**ENSG00000100580**	TMED8	0.375408985	5.10946516	10.6980255	0.0018154	0.0054613

**ENSG00000259185**		0.486902429	4.456000148	10.69138033	0.0018209	0.0054763
**ENSG00000161203**	AP2M1	0.307574257	6.269933411	10.6836865	0.0018272	0.0054939
**ENSG00000172663**	TMEM134	-0.536994984	4.569726879	10.67894149	0.0018312	0.0055042
**ENSG00000101558**	VAPA	0.230582185	7.128519898	10.67478961	0.0018346	0.0055130
**ENSG00000175895**	PLEKHF2	0.480890894	5.060282715	10.67311406	0.0018360	0.0055146
**ENSG00000117385**	P3H1	-0.531367131	4.520163179	10.67287953	0.0018362	0.0055146
**ENSG00000003402**	CFLAR	-0.208296691	8.762067828	10.67076121	0.0018380	0.0055184
**ENSG00000124374**	PAIP2B	-0.575432515	4.024139609	10.66636703	0.0018417	0.0055278
**ENSG00000077097**	MIR4442	0.231695727	8.647589656	10.66331563	0.0018443	0.0055339
**ENSG00000155330**	c16orf87	0.448907877	4.337979592	10.65281056	0.0018531	0.0055589
**ENSG00000225528**		-0.760765762	3.647929205	10.64804146	0.0018571	0.0055693
**ENSG00000122550**	KLHL7	0.41672081	4.834014706	10.64193406	0.0018623	0.0055821
**ENSG00000158769**	F11R	0.474868858	4.572692133	10.64176119	0.0018624	0.0055821
**ENSG00000174579**	MSL2	0.219377627	7.505227544	10.64076541	0.0018633	0.0055830
**ENSG00000267426**		-0.481530626	4.641198458	10.63866042	0.0018651	0.0055868
**ENSG00000171425**	ZNF581	-0.637679566	3.884965243	10.63414032	0.0018689	0.0055967
**ENSG00000114503**	NCBP2	-0.29241401	6.112231648	10.63178462	0.0018709	0.0056011
**ENSG00000090905**	TNRC6A	-0.247059566	7.446809923	10.62969027	0.0018727	0.0056048
**ENSG00000213949**	ITGA1	0.936108303	3.79793412	10.91193837	0.0018753	0.0056109
**ENSG00000137221**	TJAP1	-0.534089087	4.857908479	10.62031306	0.0018807	0.0056256
**ENSG00000125755**	SYMPK	-0.440364127	6.335986226	10.90391351	0.0018848	0.0056363
**ENSG00000105700**	KXD1	-0.402048372	5.191775697	10.60827866	0.0018911	0.0056533
**ENSG00000259589**		-0.379979332	5.045080991	10.60485519	0.0018940	0.0056605
**ENSG00000153310**	FAM49B	0.245887883	6.818316528	10.60360519	0.0018951	0.0056621
**ENSG00000259357**		0.450224056	4.697949982	10.60298999	0.0018956	0.0056621
**ENSG00000121417**	ZNF211	-0.490011914	4.884824778	10.59772032	0.0019002	0.0056741
**ENSG00000246922**	UBAP1L	-0.681918054	4.209212171	10.58989631	0.0019070	0.0056927
**ENSG00000259040**	BLOC1S5-TXND	0.527373784	4.672020572	10.58733047	0.0019092	0.0056978
**ENSG00000151883**	PARP8	0.228142102	8.470312325	10.58621953	0.0019102	0.0056990
**ENSG00000186104**	CYP2R1	-0.561747161	5.022039162	10.67485304	0.0019125	0.0057045
**ENSG00000165732**	DDX21	0.244547166	7.217740569	10.58072079	0.0019150	0.0057101
**ENSG00000116205**	MIR4781	-0.42980008	4.974534736	10.56951218	0.0019248	0.0057377
**ENSG00000081320**	STK17B	0.210625346	9.663646682	10.56490975	0.0019288	0.0057481
**ENSG00000133250**	ZNF414	-0.678838904	3.527298424	10.56095828	0.0019323	0.0057568
**ENSG00000214655**	ZSWIM8	-0.358418157	5.840831554	10.54427212	0.0019471	0.0057992
**ENSG00000134419**	rps15a	0.512429435	9.010778199	11.13046363	0.0019578	0.0058294
**ENSG00000148484**	RSU1	0.311858305	6.150007208	10.52575892	0.0019636	0.0058451

**ENSG00000108599**	AKAP10	-0.280931029	6.365535833	10.52007481	0.0019687	0.0058586
**ENSG00000229119**		0.840872718	3.889057384	10.73323362	0.0019706	0.0058625
**ENSG00000254876**	LOC100499484	-0.670801608	3.949224804	10.51400854	0.0019742	0.0058715
**ENSG00000172840**	PDP2	0.608551631	4.33567006	10.51134536	0.0019766	0.0058770
**ENSG00000232119**	MCTS1	-0.49407408	4.266142563	10.50502151	0.0019823	0.0058923
**ENSG00000197714**	ZNF460	0.28917505	5.951200644	10.50052177	0.0019864	0.0059027
**ENSG00000148985**	PGAP2	-0.501186382	3.933218924	10.49892747	0.0019878	0.0059054
**ENSG00000170185**	USP38	0.29553036	6.32571183	10.49700185	0.0019896	0.0059079
**ENSG00000105699**	LSR	0.445814088	4.714620299	10.49675565	0.0019898	0.0059079
**ENSG00000163938**	GNL3	-0.297933691	6.205458698	10.49235772	0.0019938	0.0059181
**ENSG00000127957**	PMS2P3	-0.660589905	3.63038951	10.49050456	0.0019955	0.0059214
**ENSG00000158406**	Hist1h4h	0.832988993	4.422833792	10.84907393	0.0020045	0.0059466
**ENSG00000166923**	GREM1	1.319144709	4.261076159	11.07106543	0.0020072	0.0059529
**ENSG00000104518**	GSDMD	-0.528831939	4.698559497	10.4712802	0.0020131	0.0059686
**ENSG00000009307**	CSDE1	0.220365437	9.291865515	10.47036903	0.0020139	0.0059693
**ENSG00000078140**	UBE2K	0.311616145	6.528302844	10.46669956	0.0020173	0.0059777
**ENSG00000184428**	TOP1MT	-0.632136923	3.804553707	10.46261578	0.0020211	0.0059871
**ENSG00000140153**	WDR20	0.315680189	5.358043996	10.452272	0.0020306	0.0060126
**ENSG00000079246**	XRCC5	0.196410532	8.133805362	10.45209056	0.0020308	0.0060126
**ENSG00000101695**	RNF125	0.22811667	7.236557937	10.44811957	0.0020345	0.0060218
**ENSG00000244701**		-0.765462108	3.764842285	10.44414097	0.0020382	0.0060311
**ENSG00000180879**	SSR4	0.500814873	5.499443677	10.61750284	0.0020404	0.0060358
**ENSG00000109618**	SEPSECS	-0.428190732	5.072035848	10.43456515	0.0020472	0.0060541
**ENSG00000135541**	AHI1	-0.709046365	4.234955061	10.4318396	0.0020497	0.0060591
**ENSG00000172575**	RASGRP1	0.183185393	8.549147116	10.43152665	0.0020500	0.0060591
**ENSG00000103657**	HERC1	-0.212306925	8.728377394	10.42934885	0.0020520	0.0060635
**ENSG00000260342**		0.507320772	8.643092299	11.01196989	0.0020554	0.0060714
**ENSG00000261884**		-0.454158885	5.519478307	10.47300944	0.0020559	0.0060714
**ENSG00000229127**		-0.731120008	3.981798131	10.41729417	0.0020634	0.0060918
**ENSG00000138796**	HADH	-0.407905205	4.850400681	10.41663175	0.0020640	0.0060920
**ENSG00000161791**	FMNL3	0.289735373	6.815565613	10.41292861	0.0020675	0.0061006
**ENSG00000099875**	MKNK2	0.313427991	6.574329359	10.40931828	0.0020709	0.0061089
**ENSG00000163795**	ZNF513	-0.710235949	3.625903478	10.40850789	0.0020717	0.0061095
**ENSG00000160336**	ZNF761	-0.400882858	5.243391281	10.406487	0.0020736	0.0061134
**ENSG00000272529**		-0.683790527	4.13195834	10.40470377	0.0020753	0.0061167
**ENSG00000109332**	UBE2D3	0.168862009	8.509287517	10.39941804	0.0020803	0.0061298
**ENSG00000198833**	UBE2J1	0.292228049	5.906099479	10.3852555	0.0020939	0.0061679

**ENSG00000196588**	MKL1	-0.295771884	5.910099612	10.38178758	0.0020972	0.0061759
**ENSG00000163312**	HELQ	-0.32827029	5.49996871	10.37365212	0.0021050	0.0061973
**ENSG00000240376**		0.743215649	3.786131669	10.4236933	0.0021062	0.0061988
**ENSG00000260465**		-0.434431691	4.57330019	10.36864733	0.0021099	0.0062080
**ENSG00000196371**	FUT4	0.685463899	3.961139488	10.36613992	0.0021123	0.0062134
**ENSG00000269858**	EGLN2	-0.328652537	5.973563673	10.36367436	0.0021147	0.0062170
**ENSG00000277209**	rpph1	1.139920819	6.804504642	10.95532655	0.0021147	0.0062170
**ENSG00000125676**	THOC2	-0.25268371	7.593805799	10.36067644	0.0021176	0.0062237
**ENSG00000159733**	ZFYVE28	0.501802738	4.656900101	10.35703572	0.0021211	0.0062323
**ENSG00000198780**	FAM169A	-0.386893936	5.122170896	10.35211273	0.0021259	0.0062436
**ENSG00000204516**	MICB	0.537874778	4.261238655	10.35185731	0.0021262	0.0062436
**ENSG00000237765**	FAM200B	-0.484132382	4.718653869	10.34347815	0.0021343	0.0062654
**ENSG00000198034**	RPS4X	0.332372249	10.11768641	10.6152476	0.0021348	0.0062654
**ENSG00000133739**	LRRCC1	-0.729218709	3.576156543	10.33675637	0.0021409	0.0062817
**ENSG00000112079**	STK38	-0.182260221	8.10111007	10.33408862	0.0021435	0.0062876
**ENSG00000225864**		-0.531694771	5.254854885	10.4417163	0.0021468	0.0062955
**ENSG00000175216**	CKAP5	0.258817859	6.657660805	10.32120487	0.0021562	0.0063213
**ENSG00000234072**		-0.733432206	3.703215612	10.31392457	0.0021634	0.0063407
**ENSG00000136824**	SMC2	0.381731894	5.293864138	10.31044612	0.0021669	0.0063490
**ENSG00000153179**	RASSF3	0.234261361	7.734857598	10.30460135	0.0021727	0.0063628
**ENSG00000094916**	MIR3198-2	0.241128292	7.711420392	10.30448058	0.0021728	0.0063628
**ENSG00000156860**	FBRS	-0.359278263	5.90174367	10.29842046	0.0021789	0.0063779
**ENSG00000171862**	PTEN	0.220484406	7.88890496	10.29762171	0.0021797	0.0063779
**ENSG00000167524**	SGK494	-0.740799775	4.15168613	10.35292949	0.0021798	0.0063779
**ENSG00000186919**	ZACN	-0.600818051	4.366079907	10.29136885	0.0021859	0.0063940
**ENSG00000160908**	ZNF394	-0.354772866	6.015643236	10.28133242	0.0021960	0.0064218
**ENSG00000028528**	SNX1	-0.251425736	6.867461998	10.26834761	0.0022092	0.0064575
**ENSG00000259407**		0.391162357	4.704245813	10.2680351	0.0022095	0.0064575
**ENSG00000134759**	ELP2	-0.241009029	7.062344467	10.26284533	0.0022147	0.0064711
**ENSG00000275700**	AATF	0.336520978	5.647029242	10.26130877	0.0022163	0.0064723
**ENSG00000168264**	IRF2BP2	0.242819627	7.489446227	10.26121248	0.0022164	0.0064723
**ENSG00000067334**	DNTTIP2	0.289788289	6.422735673	10.25842171	0.0022192	0.0064788
**ENSG00000173281**	PPP1R3B	0.618322163	4.366085199	10.25878179	0.0022303	0.0065092
**ENSG00000158711**	ELK4	0.211221837	8.950072445	10.24401908	0.0022340	0.0065181
**ENSG00000238221**		0.528612717	4.205496682	10.23973998	0.0022384	0.0065291
**ENSG00000259001**		1.118302024	6.813766012	10.81645179	0.0022399	0.0065318
**ENSG00000156990**	RPUSD3	-0.477122774	4.328960518	10.22152964	0.0022572	0.0065800

**ENSG00000089012**	SIRPG	-0.366483899	5.480866587	10.22102654	0.0022577	0.0065800
**ENSG00000133466**	C1QTNF6	-0.708408245	3.823080156	10.21733246	0.0022615	0.0065893
**ENSG00000022976**	ZNF839	-0.52596667	4.220515649	10.21664922	0.0022622	0.0065896
**ENSG00000167863**	ATP5H	0.420999119	5.685156935	10.26827278	0.0022653	0.0065968
**ENSG00000229994**		0.623031644	3.584796789	10.21104075	0.0022681	0.0066029
**ENSG00000139505**	MTMR6	0.296892941	6.204908417	10.20293854	0.0022765	0.0066257
**ENSG00000076003**	MCM6	0.388871708	5.356153653	10.20160634	0.0022779	0.0066279
**ENSG00000164134**	NAA15	0.231792058	6.994373918	10.19204843	0.0022880	0.0066552
**ENSG00000227124**	ZNF717	-0.644665389	3.89094363	10.19010594	0.0022900	0.0066593
**ENSG00000082258**	CCNT2	-0.247028462	7.150206564	10.18918154	0.0022910	0.0066603
**ENSG00000175787**	ZNF169	-0.667669691	4.010928372	10.18779263	0.0022924	0.0066627
**ENSG00000100124**	ANKRD54	-0.651766221	3.456890215	10.18391127	0.0022965	0.0066727
**ENSG00000055044**	NOP58	-0.266740306	7.058505797	10.1830758	0.0022974	0.0066735
**ENSG00000136908**	DPM2	-0.580600451	4.25894618	10.17934864	0.0023014	0.0066830
**ENSG00000215305**	VPS16	-0.442241592	4.809373795	10.1765342	0.0023043	0.0066898
**ENSG00000264772**		0.213997839	7.562343283	10.16789114	0.0023135	0.0067146
**ENSG00000034693**	PEX3	-0.453614735	4.730970045	10.16643911	0.0023151	0.0067172
**ENSG00000152683**	SLC30A6	0.290475929	5.827866947	10.1641739	0.0023175	0.0067224
**ENSG00000260017**		-0.53397519	4.290154061	10.16348255	0.0023182	0.0067227
**ENSG00000172687**		-0.690133546	3.376290655	10.15042841	0.0023322	0.0067601
**ENSG00000266820**		-0.956533109	3.885917243	10.45134875	0.0023324	0.0067601
**ENSG00000132952**	USPL1	-0.362729369	5.906173744	10.14231114	0.0023409	0.0067828
**ENSG00000241741**		0.639908315	3.844801267	10.13991839	0.0023435	0.0067884
**ENSG00000157426**	AASDH	-0.434886886	4.995894085	10.13865142	0.0023449	0.0067905
**ENSG00000154727**	GABPA	0.264711684	6.394164966	10.13579303	0.0023479	0.0067976
**ENSG00000140319**	SRP14	0.285398898	6.844863754	10.1332922	0.0023506	0.0068035
**ENSG00000129083**	COPB1	0.193962339	7.176312441	10.12267183	0.0023622	0.0068331
**ENSG00000198919**	DZIP3	-0.423115693	5.690803032	10.14738217	0.0023622	0.0068331
**ENSG00000200320**	SNORA63	0.937231152	5.489817831	10.6820904	0.0023644	0.0068375
**ENSG00000204209**	DAXX	0.345509811	5.69267583	10.11738815	0.0023679	0.0068459
**ENSG00000137309**	HMGA1	0.393037378	5.632710938	10.11587447	0.0023696	0.0068482
**ENSG00000133393**	FOPNL	0.371895946	5.090939988	10.11544819	0.0023700	0.0068482
**ENSG00000235288**		0.704641773	3.461028941	10.11362017	0.0023720	0.0068521
**ENSG00000144224**	UBXN4	-0.19922507	7.524662284	10.11163013	0.0023742	0.0068559
**ENSG00000101182**	PSMA7	0.309301581	6.021305082	10.11121052	0.0023747	0.0068559
**ENSG00000149483**	TMEM138	-0.409735599	4.939457547	10.10975583	0.0023763	0.0068586
**ENSG00000178764**	ZHX2	0.302396958	5.93281232	10.10425838	0.0023823	0.0068741

**ENSG00000253719**	ATXN7L3B	-0.237030373	6.815196405	10.1007701	0.0023861	0.0068833
**ENSG00000274265**		0.646556911	3.439323972	10.09371052	0.0023939	0.0069011
**ENSG00000115268**	RPS15	0.44968207	7.972972515	10.61967815	0.0023942	0.0069011
**ENSG00000117000**	RLF	0.313736881	6.195171046	10.09314449	0.0023945	0.0069011
**ENSG00000270316**	BORCS7	-0.700527378	3.416635464	10.09276241	0.0023949	0.0069011
**ENSG00000069956**	MAPK6	0.365565306	5.564636212	10.08060923	0.0024084	0.0069380
**ENSG00000158417**	EIF5B	0.33007657	7.416928487	10.2975868	0.0024107	0.0069428
**ENSG00000127526**	SLC35E1	0.37241545	5.523443437	10.07743298	0.0024119	0.0069443
**ENSG00000139239**		0.621204936	6.091234032	10.60691928	0.0024136	0.0069471
**ENSG00000246705**	H2AFJ	-0.719197328	3.443280561	10.07535028	0.0024142	0.0069471
**ENSG00000163545**	NUAK2	-0.418175059	5.117797518	10.06487463	0.0024259	0.0069788
**ENSG00000131127**	ZNF141	-0.295827542	6.241688697	10.06254589	0.0024285	0.0069844
**ENSG00000066933**	MYO9A	0.280577836	6.896960696	10.05527178	0.0024367	0.0070060
**ENSG00000151665**	PIGF	-0.400351503	4.875129735	10.03789431	0.0024563	0.0070604
**ENSG00000136149**		0.84109896	6.31768033	10.5935566	0.0024572	0.0070610
**ENSG00000118412**	CASP8AP2	0.243083704	7.378896697	10.03347424	0.0024613	0.0070709
**ENSG00000131069**	ACSS2	-0.722852763	3.495930455	10.02690193	0.0024688	0.0070904
**ENSG00000226167**	AP4B1-AS1	-0.662903849	4.247234509	10.03573609	0.0024699	0.0070916
**ENSG00000233426**		0.624212182	4.204228719	10.02352043	0.0024726	0.0070976
**ENSG00000127337**	YEATS4	-0.622926205	4.115880809	10.01964747	0.0024770	0.0071084
**ENSG00000100714**	MTHFD1	-0.353098408	5.571631455	10.01364624	0.0024839	0.0071261
**ENSG00000271092**	TMEM56-RWDD	-0.579057086	3.48541361	10.01001653	0.0024881	0.0071361
**ENSG00000136044**	appl2	-0.334094853	5.937955465	10.00673066	0.0024919	0.0071450
**ENSG00000137145**	DENND4C	0.198016598	7.407475943	9.99976537	0.0024999	0.0071660
**ENSG00000135521**	LTV1	-0.380396756	5.150160008	9.992481767	0.0025083	0.0071882
**ENSG00000255823**	MTRNR2L8	1.122090406	5.778768383	10.53921654	0.0025140	0.0072012
**ENSG00000179262**	RAD23A	0.321999191	5.50857949	9.987375719	0.0025142	0.0072012
**ENSG00000092330**	TINF2	0.317568624	5.616881219	9.985924994	0.0025159	0.0072041
**ENSG00000188732**	FAM221A	-0.635613043	3.711607315	9.979677547	0.0025232	0.0072229
**ENSG00000124614**	RPS10	0.443329135	8.589066268	10.49633228	0.0025259	0.0072288
**ENSG00000231500**	RPS18	0.497410988	9.821402558	10.50830939	0.0025323	0.0072449
**ENSG00000143157**	POGK	0.295653824	6.306933812	9.9689046	0.0025358	0.0072530
**ENSG00000151422**	FER	-0.467608755	4.557016316	9.967205689	0.0025378	0.0072558
**ENSG00000064393**	HIPK2	-0.252322374	7.189373358	9.966877659	0.0025381	0.0072558
**ENSG00000231310**		0.478845878	4.347556501	9.964092454	0.0025414	0.0072632
**ENSG00000236439**		0.881499919	5.241738959	10.49910842	0.0025422	0.0072634
**ENSG00000180787**	ZFP3	-0.48519893	4.649842288	9.959947029	0.0025463	0.0072731

**ENSG00000188227**	ZNF793	-0.560813187	4.483329311	9.955387962	0.0025517	0.0072864
**ENSG00000250182**		0.699795776	6.368653523	10.49811617	0.0025533	0.0072891
**ENSG00000171466**	ZNF562	0.378032457	5.57270863	9.950587237	0.0025573	0.0072967
**ENSG00000184260**	HIST2H2AC	0.941897348	5.424002892	10.49341719	0.0025573	0.0072967
**ENSG00000127124**	HIVEP3	0.385874865	4.981477993	9.946014182	0.0025627	0.0073101
**ENSG00000096063**	SRPK1	0.277811721	6.201217442	9.943970137	0.0025652	0.0073134
**ENSG00000166341**	DCHS1	-0.58836348	4.627036875	9.962913695	0.0025653	0.0073134
**ENSG00000275791**		0.648841351	3.576590733	9.940373499	0.0025694	0.0073232
**ENSG00000111875**	ASF1A	0.303864601	6.221361308	9.939776555	0.0025701	0.0073232
**ENSG00000115145**	STAM2	0.347422966	5.694436403	9.938191708	0.0025720	0.0073256
**ENSG00000141252**	VPS53	-0.265189355	6.351958813	9.93778533	0.0025725	0.0073256
**ENSG00000085382**	HACE1	-0.436896005	4.987065375	9.937234851	0.0025732	0.0073256
**ENSG00000176055**	MBLAC2	-0.465212121	4.635551035	9.936131745	0.0025745	0.0073256
**ENSG00000263264**		-0.323806057	5.88464103	9.936119184	0.0025745	0.0073256
**ENSG00000120158**	RCL1	-0.515909324	4.192045133	9.933340845	0.0025778	0.0073330
**ENSG00000181852**	RNF41	0.344103018	5.243198561	9.925613385	0.0025870	0.0073555
**ENSG00000154310**	TNIK	0.218818453	7.506447663	9.925546477	0.0025871	0.0073555
**ENSG00000283930**		-0.603876283	3.709651407	9.915322543	0.0025994	0.0073884
**ENSG00000176208**	ATAD5	-0.45261258	4.965716877	9.91176398	0.0026036	0.0073985
**ENSG00000130066**	SAT1	-0.382884905	5.852549227	9.905342889	0.0026114	0.0074185
**ENSG00000171960**	PPIH	-0.61055086	3.968248027	9.901187408	0.0026164	0.0074308
**ENSG00000149273**	RPS3	0.454934219	9.194375225	10.41680989	0.0026195	0.0074375
**ENSG00000267135**		0.914803796	4.163650271	10.28940172	0.0026213	0.0074407
**ENSG00000111144**	LTA4H	-0.293705065	6.170035823	9.894847113	0.0026241	0.0074465
**ENSG00000107341**	UBE2R2	0.332426195	5.907331909	9.893687929	0.0026255	0.0074473
**ENSG00000260032**	NORAD	0.215287895	8.668676971	9.893454302	0.0026258	0.0074473
**ENSG00000164038**	SLC9B2	-0.481108984	4.518804595	9.890334332	0.0026296	0.0074560
**ENSG00000123815**	COQ8B	-0.724161919	3.517780755	9.88236824	0.0026393	0.0074815
**ENSG00000211721**		0.707880526	3.610491586	9.864106076	0.0026617	0.0075430
**ENSG00000162542**	TMCO4	-0.568266309	4.090943114	9.857750901	0.0026695	0.0075632
**ENSG00000092931**	MFSD11	-0.348656336	6.040409907	9.839833683	0.0026918	0.0076224
**ENSG00000001084**	GCLC	0.455735417	4.953398089	9.839754606	0.0026919	0.0076224
**ENSG00000260537**	DDX19A	-0.320236576	5.378360173	9.837425077	0.0026948	0.0076285
**ENSG00000198538**	ZNF28	0.369477722	5.163821557	9.826448206	0.0027085	0.0076654
**ENSG00000130724**	CHMP2A	0.386009842	4.999807383	9.812128715	0.0027266	0.0077143
**ENSG00000254462**		0.519491275	3.998091386	9.810158128	0.0027291	0.0077193
**ENSG00000251201**	TMED7-TICAM2	0.506891832	4.404433588	9.808345344	0.0027314	0.0077237

**ENSG00000170525**	PFKFB3	0.474795482	4.771575943	9.801220268	0.0027404	0.0077471
**ENSG00000198331**	HYLS1	0.653558722	3.357244086	9.798215345	0.0027442	0.0077550
**ENSG00000166181**	API5	0.226136457	7.045869343	9.797854202	0.0027447	0.0077550
**ENSG00000273088**		0.498162128	4.859146248	9.794955092	0.0027484	0.0077634
**ENSG00000198876**	DCAF12	0.387494119	4.99787831	9.793417791	0.0027503	0.0077668
**ENSG00000005812**	FBXL3	0.225374161	7.458960894	9.789126356	0.0027558	0.0077802
**ENSG00000105438**	KDELR1	0.466802509	4.784449341	9.787593395	0.0027578	0.0077836
**ENSG00000243305**		-0.720399355	3.751365034	9.78202382	0.0027649	0.0077999
**ENSG00000151148**	UBE3B	-0.312199402	5.843565637	9.781918229	0.0027650	0.0077999
**ENSG00000132646**	PCNA	0.454187239	4.864532464	9.780334264	0.0027671	0.0078036
**ENSG00000233016**	Snhg7	-0.575388164	4.337373414	9.77966532	0.0027679	0.0078039
**ENSG00000164074**	ABHD18	-0.365847309	5.619185029	9.7752981	0.0027735	0.0078176
**ENSG00000175220**	ARHGAP1	0.328870613	6.025145645	9.760562787	0.0027926	0.0078691
**ENSG00000101019**	UQCC1	-0.445243644	4.831643147	9.756758732	0.0027975	0.0078809
**ENSG00000126698**	DNAJC8	0.333341178	6.144762369	9.752750221	0.0028027	0.0078916
**ENSG00000069869**	NEDD4	0.647914757	4.082090534	9.752663003	0.0028028	0.0078916
**ENSG00000113761**	ZNF346	-0.513497556	4.196664332	9.750730979	0.0028053	0.0078966
**ENSG00000115275**	MOGS	-0.417382189	5.391082583	9.745137658	0.0028126	0.0079150
**ENSG00000163519**	trat1	-0.26588251	7.699702676	9.771297094	0.0028185	0.0079295
**ENSG00000188092**	GPR89B	-0.685148714	3.751963695	9.735966879	0.0028246	0.0079445
**ENSG00000277778**	PGM5P2	-0.590524232	3.789848627	9.735277335	0.0028255	0.0079449
**ENSG00000268205**		0.326622613	5.872982416	9.733868775	0.0028274	0.0079469
**ENSG00000196312**	MFSD14C	-0.591178811	4.173768149	9.733567828	0.0028278	0.0079469
**ENSG00000111642**	CHD4	0.207547152	7.844246012	9.717770343	0.0028486	0.0080033
**ENSG00000187954**	CYHR1	-0.615619512	4.346362761	9.715687139	0.0028514	0.0080089
**ENSG00000279696**		-0.468927888	4.345030683	9.712797557	0.0028552	0.0080175
**ENSG00000138430**	OLA1	0.426148636	5.450029079	9.720307296	0.0028591	0.0080262
**ENSG00000228144**	TMBIM4	0.342350096	5.922911426	9.699479464	0.0028729	0.0080629
**ENSG00000254635**		-0.445030149	4.720361572	9.696201139	0.0028773	0.0080730
**ENSG00000151332**	MBIP	-0.408187678	4.845241141	9.692403625	0.0028824	0.0080840
**ENSG00000104823**	ECH1	0.367586057	5.262854276	9.69213154	0.0028827	0.0080840
**ENSG00000048828**	FAM120A	0.179366919	7.763297773	9.686372884	0.0028905	0.0081035
**ENSG00000151779**	NBAS	0.292528338	6.686213726	9.684342018	0.0028932	0.0081083
**ENSG00000255125**		1.026828623	4.023401755	10.07793686	0.0028938	0.0081083
**ENSG00000249855**		0.783899829	4.029580795	9.88138429	0.0028965	0.0081138
**ENSG00000167202**	TBC1D2B	0.227687672	6.64734948	9.672047238	0.0029098	0.0081489
**ENSG00000141971**	MVB12A	-0.675625225	3.86075638	9.670822106	0.0029114	0.0081514

**ENSG00000122481**	RWDD3	-0.511862156	4.006361864	9.663108917	0.0029219	0.0081785
**ENSG00000230629**		0.747532651	4.596816105	9.991493296	0.0029345	0.0082116
**ENSG00000263826**		-0.440584067	5.117016679	9.652610084	0.0029362	0.0082133
**ENSG00000160213**	CSTB	0.442987329	5.378714361	9.678591651	0.0029367	0.0082133
**ENSG00000149541**	B3GAT3	-0.603984733	3.885577168	9.646605963	0.0029444	0.0082327
**ENSG00000273329**		-0.641767384	4.406197689	9.671887342	0.0029719	0.0083074
**ENSG00000272589**	ZSWIM8-AS1	-0.539599224	4.221922847	9.625604548	0.0029733	0.0083091
**ENSG00000145247**	OCIAD2	0.47589622	4.597249996	9.62211383	0.0029782	0.0083204
**ENSG00000181804**	SLC9A9	0.440743495	4.794696248	9.614518422	0.0029887	0.0083476
**ENSG00000111843**	TMEM14C	0.576310768	3.97556441	9.603651903	0.0030039	0.0083877
**ENSG00000115649**	CNPPD1	0.391442009	4.971159825	9.598056228	0.0030117	0.0084073
**ENSG00000096093**	EFHC1	-0.651096084	4.107875226	9.595388563	0.0030155	0.0084156
**ENSG00000105516**	DBP	-0.366162618	4.88286294	9.587305947	0.0030268	0.0084425
**ENSG00000205542**	TMSB4X	0.306517103	10.53505713	9.752935033	0.0030273	0.0084425
**ENSG00000169714**	CNBP	0.21530602	8.180659835	9.586816261	0.0030275	0.0084425
**ENSG00000063244**	U2AF2	-0.273308596	6.343315299	9.583869597	0.0030317	0.0084518
**ENSG00000227775**		-0.382344671	5.548288763	9.579681359	0.0030376	0.0084661
**ENSG00000109180**	OCIAD1	-0.278593768	6.41694235	9.576951955	0.0030415	0.0084727
**ENSG00000115286**	NDUFS7	-0.582902469	4.128540746	9.576844998	0.0030416	0.0084727
**ENSG00000119013**	NDUFB3	0.592687722	4.065840485	9.5630668	0.0030612	0.0085250
**ENSG00000233476**		0.623988772	6.523068501	10.06144926	0.0030638	0.0085299
**ENSG00000197608**	ZNF841	-0.388141889	5.392854021	9.555229462	0.0030724	0.0085517
**ENSG00000111832**	RWDD1	0.326014059	5.759188114	9.551646348	0.0030776	0.0085637
**ENSG00000069712**	KIAA1107	-0.715918064	3.640606023	9.547956847	0.0030829	0.0085757
**ENSG00000213903**	LTB4R	-0.626409748	4.527306578	9.620867094	0.0030838	0.0085757
**ENSG00000176407**	KCMF1	0.305435135	5.746653531	9.546937085	0.0030843	0.0085757
**ENSG00000107959**	PITRM1	-0.27857456	6.53890851	9.539039671	0.0030957	0.0086050
**ENSG00000070214**	SLC44A1	0.448171224	5.560732334	9.630059818	0.0031011	0.0086178
**ENSG00000238622**	SNORD97	1.081849247	3.980906761	9.940675504	0.0031050	0.0086264
**ENSG00000148400**	notch1	0.295663136	6.240320912	9.526319417	0.0031141	0.0086474
**ENSG00000185033**	SEMA4B	0.613612438	3.465286092	9.526226453	0.0031143	0.0086474
**ENSG00000177600**	RPLP2	0.542653904	9.437299638	10.02189856	0.0031219	0.0086663
**ENSG00000138802**	SEC24B	0.217801522	7.2298959	9.513691789	0.0031325	0.0086935
**ENSG00000004139**	SARM1	-0.389791679	5.499657607	9.486999476	0.0031718	0.0088002
**ENSG00000057757**	PITHD1	0.357736725	4.9274652	9.48576539	0.0031736	0.0088029
**ENSG00000265784**		0.441582767	4.831124864	9.482237179	0.0031789	0.0088151
**ENSG00000184357**	HIST1H1B	0.718170416	3.80244754	9.501993085	0.0031822	0.0088220

**ENSG00000162704**	ARPC5	0.225350914	7.46228582	9.477350389	0.0031861	0.0088306
**ENSG00000164587**	RPS14	0.509528283	8.748037765	9.970635665	0.0031874	0.0088317
**ENSG00000164944**	KIAA1429	0.21976913	6.821194341	9.470266253	0.0031967	0.0088551
**ENSG00000101199**	ARFGAP1	-0.54796018	4.760773623	9.481865495	0.0031980	0.0088564
**ENSG00000072756**	TRNT1	-0.282645222	6.434491993	9.458491313	0.0032143	0.0088987
**ENSG00000277203**	f8a1	0.624868703	3.839659401	9.457802445	0.0032154	0.0088987
**ENSG00000106829**	TLE4	0.236971249	6.739346504	9.457497213	0.0032158	0.0088987
**ENSG00000267074**		0.438365893	5.27494636	9.44507936	0.0032345	0.0089481
**ENSG00000243667**	WDR92	-0.56439903	3.89610725	9.437766196	0.0032456	0.0089764
**ENSG00000168612**	ZSWIM1	-0.594440413	4.393369467	9.429530217	0.0032581	0.0090086
**ENSG00000109944**	C11orf63	0.639997391	3.549098586	9.427353352	0.0032614	0.0090154
**ENSG00000080200**	CRYBG3	0.322770268	5.737953662	9.426782956	0.0032623	0.0090154
**ENSG00000177888**	ZBTB41	0.2960502	5.965318946	9.425125767	0.0032648	0.0090177
**ENSG00000196262**	PPIA	0.266903828	7.705101303	9.468446271	0.0032649	0.0090177
**ENSG00000155545**	MIER3	0.307793793	5.628496559	9.42424342	0.0032662	0.0090190
**ENSG00000171606**	ZNF274	-0.318704759	5.512030278	9.419243098	0.0032738	0.0090377
**ENSG00000111674**	ENO2	-0.424747121	4.932344898	9.412918242	0.0032835	0.0090621
**ENSG00000150477**	KIAA1328	-0.515462796	4.579773967	9.412135324	0.0032847	0.0090630
**ENSG00000212664**		0.680966967	4.588114582	9.644483736	0.0032897	0.0090744
**ENSG00000167778**	spryd3	0.487573401	4.681670901	9.40761587	0.0032917	0.0090769
**ENSG00000223547**	ZNF844	-0.619979961	4.420096123	9.447774214	0.0032924	0.0090769
**ENSG00000187109**	NAP1L1	0.244095017	9.76327916	9.404320822	0.0032968	0.0090866
**ENSG00000008710**	MIR6511B1	-0.589041849	5.669776361	9.772325864	0.0032990	0.0090903
**ENSG00000123353**	ORMDL2	0.428207556	4.596680629	9.397469035	0.0033073	0.0091103
**ENSG00000139675**	HNRNPA1L2	-0.508899732	4.255102015	9.397060371	0.0033080	0.0091103
**ENSG00000197555**	SIPA1L1	0.276008096	6.042255943	9.394650485	0.0033117	0.0091182
**ENSG00000183137**	CEP57L1	-0.635538408	3.603371272	9.389490818	0.0033197	0.0091378
**ENSG00000166261**	ZNF202	-0.461840479	4.366886464	9.378065934	0.0033375	0.0091840
**ENSG00000196510**	ANAPC7	-0.343089265	5.900511515	9.377599696	0.0033382	0.0091840
**ENSG00000118640**	VAMP8	0.585952588	4.552002896	9.430501246	0.0033495	0.0092126
**ENSG00000256966**	FBXO10	0.538962342	4.241504133	9.36442994	0.0033589	0.0092359
**ENSG00000173366**		0.397270437	4.688952536	9.362562556	0.0033618	0.0092416
**ENSG00000180104**	EXOC3	-0.279744443	6.128750711	9.3612728	0.0033639	0.0092447
**ENSG00000155115**	GTF3C6	0.564392827	4.183347866	9.359532041	0.0033666	0.0092498
**ENSG00000256453**	DND1	-0.638425666	3.496892014	9.356177418	0.0033719	0.0092619
**ENSG00000070540**	WIPI1	-0.575159835	4.300182761	9.34146287	0.0033952	0.0093235
**ENSG00000110583**	NAA40	-0.652172214	4.042550672	9.320162128	0.0034293	0.0094146

**ENSG00000242372**	EIF6	0.555980725	3.66105401	9.311150018	0.0034438	0.0094519
**ENSG00000085377**	PREP	0.374019251	5.372738785	9.308894333	0.0034474	0.0094595
**ENSG00000068745**	IP6K2	-0.336709347	5.358073846	9.303137575	0.0034567	0.0094825
**ENSG00000171858**	RPS21	0.604821002	8.103595676	9.784192369	0.0034579	0.0094833
**ENSG00000116171**	SCP2	0.323040577	6.018843772	9.300672042	0.0034607	0.0094885
**ENSG00000235174**		0.788952054	5.630086336	9.768032853	0.0034721	0.0095173
**ENSG00000180902**	D2HGDH	-0.928467903	4.127800871	9.584190361	0.0034766	0.0095271
**ENSG00000172340**	SUCLG2	-0.267349983	6.061000052	9.286781972	0.0034834	0.0095430
**ENSG00000143093**	STRIP1	-0.422926647	5.373193128	9.284851037	0.0034865	0.0095492
**ENSG00000093000**	NUP50	0.207870341	7.592052043	9.278798783	0.0034964	0.0095738
**ENSG00000158092**	NCK1	0.301673504	5.764086851	9.277690566	0.0034982	0.0095763
**ENSG00000155959**	VBP1	0.341917969	5.061009692	9.272140686	0.0035074	0.0095987
**ENSG00000205236**		-0.598089277	3.954371394	9.271136288	0.0035090	0.0096007
**ENSG00000250903**		-0.597738467	4.175844203	9.267851378	0.0035144	0.0096130
**ENSG00000146842**	TMEM209	0.279776006	5.938715669	9.266581576	0.0035165	0.0096163
**ENSG00000265479**	DTX2P1-UPK3BI	-0.498927626	4.467683511	9.258529861	0.0035298	0.0096501
**ENSG00000170638**	TRABD	-0.60165524	5.402651932	9.57417125	0.0035412	0.0096779
**ENSG00000140990**	NDUFB10	0.428831237	4.468971253	9.251297934	0.0035418	0.0096779
**ENSG00000129355**	CDKN2D	0.625810747	3.423241018	9.248124076	0.0035471	0.0096898
**ENSG00000124459**	ZNF45	0.395771331	4.74457148	9.245216737	0.0035520	0.0097005
**ENSG00000133706**	LARS	-0.21168007	7.281387351	9.243426685	0.0035549	0.0097061
**ENSG00000181666**	HKR1	-0.374738428	5.130394606	9.241471933	0.0035582	0.0097085
**ENSG00000120370**	GORAB	-0.550943758	4.313733447	9.241307869	0.0035585	0.0097085
**ENSG00000144741**	SLC25A26	0.302901841	5.531754252	9.241238977	0.0035586	0.0097085
**ENSG00000268043**	NBPF12	-0.31461675	5.668280437	9.239908652	0.0035608	0.0097120
**ENSG00000159640**	ACE	0.670908318	3.601028523	9.231424629	0.0035750	0.0097482
**ENSG00000168710**	AHCYL1	0.310544879	5.848602703	9.229851493	0.0035777	0.0097529
**ENSG00000263212**		-0.673149571	3.508697508	9.220239464	0.0035939	0.0097945
**ENSG00000185085**	INTS5	0.50673528	3.998328513	9.215219855	0.0036023	0.0098150
**ENSG00000247137**		-0.417516265	4.807892128	9.207517713	0.0036154	0.0098480
**ENSG00000203876**		-0.657776328	3.607591593	9.205004241	0.0036197	0.0098571
**ENSG00000101911**	PRPS2	0.439401419	4.79301331	9.200804113	0.0036268	0.0098740
**ENSG00000176022**	B3GALT6	0.659271549	3.584576607	9.193959876	0.0036385	0.0099033
**ENSG00000120784**	ZFP30	0.397181853	4.683430632	9.192494533	0.0036410	0.0099075
**ENSG00000125971**	DYNLRB1	0.365662806	5.413809341	9.188672782	0.0036476	0.0099227
**ENSG00000135597**	REPS1	-0.291271583	6.655977403	9.182532954	0.0036581	0.0099488
**ENSG00000129562**	DAD1	0.448467374	4.637839068	9.177622115	0.0036666	0.0099692

**ENSG00000100364**	KIAA0930	0.502092979	4.103727965	9.174835316	0.0036714	0.0099797
**ENSG00000008952**	SEC62	0.290623545	7.490061729	9.273089032	0.0037034	0.0100641
**ENSG00000156026**	MCU	0.526672684	3.792169427	9.151680071	0.0037116	0.0100834
**ENSG00000237054**	PRMT5-AS1	-0.475928841	4.192737823	9.150960353	0.0037128	0.0100834
**ENSG00000278540**	ACACA	0.550275463	5.387608868	9.41401954	0.0037143	0.0100834
**ENSG00000189043**	NDUFA4	0.344531315	5.984559188	9.150102926	0.0037143	0.0100834
**ENSG00000109466**	KLHL2	-0.354133463	5.117559423	9.1490489	0.0037162	0.0100858
**ENSG00000108349**	MIR6866	-0.245819268	6.493299752	9.14732898	0.0037192	0.0100875
**ENSG00000231999**	FLJ27354	-0.566126278	4.144230384	9.147268154	0.0037193	0.0100875
**ENSG00000061987**	MON2	-0.261004126	7.590120965	9.162561531	0.0037197	0.0100875
**ENSG00000074696**	HACD3	0.337704319	5.416661224	9.13558986	0.0037398	0.0101393
**ENSG00000123136**	DDX39A	-0.356210732	5.594178703	9.134183853	0.0037423	0.0101434
**ENSG00000135441**	BLOC1S1	0.549345491	4.046036915	9.133528652	0.0037434	0.0101439
**ENSG00000113360**	DROSHA	-0.263855563	6.39389901	9.130250767	0.0037492	0.0101569
**ENSG00000182400**	TRAPPC6B	-0.315728471	5.65725884	9.126386353	0.0037560	0.0101728
**ENSG00000160321**	znf208	-0.634314094	4.107791333	9.113307086	0.0037792	0.0102322
**ENSG00000204463**	BAG6	-0.275806636	6.070040561	9.112910241	0.0037799	0.0102322
**ENSG00000143543**	JTB	0.354685147	5.421089583	9.110536989	0.0037842	0.0102410
**ENSG00000163682**	RPL9	0.344270531	9.015042268	9.44230985	0.0037909	0.0102566
**ENSG00000058262**	SEC61A1	0.256435432	6.935861936	9.103501952	0.0037967	0.0102697
**ENSG00000071243**	ING3	-0.390687564	5.110818015	9.099044993	0.0038047	0.0102886
**ENSG00000117620**	SLC35A3	-0.265575274	6.331826767	9.095402062	0.0038112	0.0103036
**ENSG00000142065**	ZFP14	-0.354328652	5.194810492	9.093918103	0.0038139	0.0103067
**ENSG00000172732**	MUS81	-0.42220063	4.649548918	9.093499523	0.0038147	0.0103067
**ENSG00000155100**	OTUD6B	-0.382100852	4.811662929	9.093128387	0.0038153	0.0103067
**ENSG00000198276**	MIR1914	-0.57839867	4.725361287	9.151491315	0.0038175	0.0103100
**ENSG00000105656**	ELL	0.440591122	4.457031025	9.0900699	0.0038208	0.0103162
**ENSG00000203709**	Mir29b2	-0.426672053	6.264950366	9.322413514	0.0038367	0.0103564
**ENSG00000174405**	LIG4	-0.511444447	4.436046333	9.078078882	0.0038425	0.0103693
**ENSG00000100852**	ARHGAP5	0.230989041	7.687167001	9.073646182	0.0038505	0.0103883
**ENSG00000158604**	TMED4	-0.287810537	6.276664922	9.070297007	0.0038566	0.0104020
**ENSG00000155974**	GRIP1	-0.569273289	4.36873081	9.068074912	0.0038606	0.0104102
**ENSG00000169967**	MAP3K2	0.222304198	7.47313163	9.06256314	0.0038707	0.0104300
**ENSG00000117543**	DPH5	-0.402161595	4.974663422	9.062435194	0.0038709	0.0104300
**ENSG00000159579**	RSPRY1	0.307669021	5.816225121	9.062318519	0.0038711	0.0104300
**ENSG00000130475**	FCHO1	-0.362002009	5.40757251	9.061860344	0.0038720	0.0104300
**ENSG00000111540**	RAB5B	0.288229699	6.421537346	9.057409677	0.0038801	0.0104492

**ENSG00000280148**		-0.609616051	3.985305459	9.056152854	0.0038824	0.0104528
**ENSG00000126215**	XRCC3	-0.577206724	4.457982181	9.049081323	0.0038954	0.0104850
**ENSG00000129292**	PHF20L1	-0.281334067	7.174029333	9.083654551	0.0038992	0.0104927
**ENSG00000187531**	SIRT7	-0.573964178	4.865138587	9.168249828	0.0039023	0.0104981
**ENSG00000175105**	ZNF654	0.316632546	6.182367589	9.044793622	0.0039032	0.0104981
**ENSG00000220205**	VAMP2	-0.291357755	6.735722856	9.042947651	0.0039066	0.0105045
**ENSG00000167257**	RNF214	0.339133511	5.832264295	9.032137875	0.0039266	0.0105555
**ENSG00000189114**	BLOC1S3	0.619942764	3.918660442	9.029116625	0.0039322	0.0105661
**ENSG00000187735**	TCEA1	0.284299208	6.348799759	9.028915035	0.0039326	0.0105661
**ENSG00000213995**	NAXD	-0.493319629	4.568236211	9.027043134	0.0039361	0.0105728
**ENSG00000250186**		0.617395277	3.578533187	9.021810942	0.0039458	0.0105938
**ENSG00000248923**		0.671871763	4.079397659	9.087937139	0.0039459	0.0105938
**ENSG00000183576**	SETD3	0.301858489	5.792430887	9.018136268	0.0039526	0.0106091
**ENSG00000145439**	CBR4	-0.419361024	4.955392049	9.015351966	0.0039578	0.0106203
**ENSG00000155506**	LARP1	0.196916242	7.701930602	9.00918795	0.0039694	0.0106485
**ENSG00000105993**	DNAJB6	-0.258706591	6.519139155	9.004365601	0.0039784	0.0106701
**ENSG00000263266**		0.647636956	6.303321834	9.445700775	0.0039887	0.0106949
**ENSG00000177733**	HNRNPA0	0.271542342	6.322172049	8.990693394	0.0040042	0.0107337
**ENSG00000158987**	RAPGEF6	-0.205042175	8.532399874	8.987082962	0.0040110	0.0107492
**ENSG00000116455**	WDR77	-0.436851734	4.860080243	8.980304832	0.0040239	0.0107809
**ENSG00000124151**	NCOA3	0.177277282	7.920652516	8.970618041	0.0040423	0.0108276
**ENSG00000225972**		0.858694457	4.536876431	9.372669356	0.0040458	0.0108340
**ENSG00000229036**		-0.727132578	3.443014019	8.964776058	0.0040535	0.0108520
**ENSG00000188554**	NBR1	0.204760127	7.03940372	8.960375163	0.0040619	0.0108711
**ENSG00000152942**	RAD17	-0.343731377	5.396505242	8.959967949	0.0040627	0.0108711
**ENSG00000104824**	HNRNPL	-0.195831667	7.375099967	8.957071598	0.0040683	0.0108832
**ENSG00000127184**	MIR3607	0.485017745	6.683437156	9.347808747	0.0040892	0.0109365
**ENSG00000135974**	c2orf49	-0.378832671	5.353586987	8.940151002	0.0041010	0.0109650
**ENSG00000107779**	BMPR1A	0.413440104	4.69380558	8.93380918	0.0041133	0.0109951
**ENSG00000167207**	NOD2	0.592731483	3.495652535	8.931960362	0.0041169	0.0110019
**ENSG00000183562**		-0.623055814	4.654578282	9.039250852	0.0041208	0.0110096
**ENSG00000177570**	SAMD12	-0.763676952	3.943823239	9.001916901	0.0041252	0.0110185
**ENSG00000196756**	SNHG17	-0.631819114	3.989795107	8.922051238	0.0041362	0.0110449
**ENSG00000112787**	FBRSL1	-0.425478462	5.61602918	8.97969544	0.0041377	0.0110449
**ENSG00000123505**	AMD 1.00	0.234733304	7.134585133	8.921006517	0.0041383	0.0110449
**ENSG00000119328**	FAM206A	-0.632856673	3.58711788	8.917542391	0.0041450	0.0110602
**ENSG00000147874**	HAUS6	-0.276195342	5.892157631	8.915682149	0.0041487	0.0110671

**ENSG00000196950**	SLC39A10	0.223904518	6.796384897	8.915131605	0.0041498	0.0110672
**ENSG00000188994**	ZNF292	0.227577333	8.145347693	8.912102839	0.0041557	0.0110802
**ENSG00000167967**	E4F1	-0.572486406	4.039376865	8.909208641	0.0041614	0.0110926
**ENSG00000087263**	OGFOD1	0.28601046	5.784168704	8.908290303	0.0041632	0.0110946
**ENSG00000178922**	HYI	-0.571582158	3.961091161	8.907393955	0.0041650	0.0110965
**ENSG00000204410**	MSH5	-0.700651264	3.836310101	8.901965156	0.0041757	0.0111222
**ENSG00000143379**	SETDB1	-0.301986892	5.83993145	8.899175442	0.0041812	0.011132
**ENSG00000245748**	LOC100129931	0.376549191	5.321760686	8.898963941	0.0041816	0.011132
**ENSG00000258441**	LINC00641	-0.424905391	6.190049885	9.118959248	0.0041826	0.011132
**ENSG00000163947**	ARHGEF3	0.200983242	7.745554928	8.894968404	0.0041896	0.0111477
**ENSG00000151247**	EIF4E	0.305434565	5.890898689	8.892138696	0.0041952	0.0111583
**ENSG00000264364**	DYNLL2	0.260077866	6.922787996	8.891893821	0.0041957	0.0111583
**ENSG00000101452**	DHX35	-0.451689909	4.337294555	8.87634812	0.0042266	0.0112378
**ENSG00000005022**	SLC25A5	0.369407752	5.717125531	8.874149609	0.0042310	0.0112467
**ENSG00000088035**	ALG6	-0.397572452	5.176150539	8.866777945	0.0042458	0.0112831
**ENSG00000126432**	PRDX5	0.690673873	3.386121856	8.863905621	0.0042516	0.0112956
**ENSG00000149474**	KAT14	-0.364760991	4.989401292	8.855773126	0.0042680	0.0113363
**ENSG00000159176**	CSRP1	0.397988154	4.704853557	8.850576378	0.0042786	0.0113614
**ENSG00000270704**		-0.614774628	4.239818372	8.845640015	0.0042886	0.0113851
**ENSG00000160799**	CCDC12	0.374813643	4.61852546	8.843887595	0.0042921	0.0113917
**ENSG00000116604**	MEF2D	0.310317414	6.012390285	8.841385941	0.0042972	0.0114023
**ENSG00000164068**	RNF123	-0.464594698	5.284685676	8.892282122	0.0043010	0.0114070
**ENSG00000172765**	TMCC1	0.334808321	5.204436613	8.839441401	0.0043012	0.0114070
**ENSG00000229659**		0.842569436	3.833236534	9.056868132	0.0043028	0.0114074
**ENSG00000136811**	ODF2	-0.363214256	5.435917178	8.838296121	0.0043035	0.0114074
**ENSG00000273221**		-0.391603865	5.617074568	8.837334727	0.0043057	0.0114104
**ENSG00000283041**		0.479375802	6.877453399	9.241062711	0.0043172	0.0114379
**ENSG00000184575**	XPOT	0.217012713	6.850240087	8.828029212	0.0043245	0.0114544
**ENSG00000197892**	KIF13B	0.320652712	5.54911746	8.826552519	0.0043275	0.0114595
**ENSG00000138386**	NAB1	0.341606966	5.374198693	8.82338875	0.0043340	0.0114738
**ENSG00000104866**	PPP1R37	-0.546459229	3.896947394	8.815692613	0.0043499	0.0115128
**ENSG00000166226**	CCT2	-0.247250373	6.336979762	8.815151334	0.0043510	0.0115128
**ENSG00000144426**	NBEAL1	0.287613594	6.421798224	8.813193886	0.0043550	0.0115156
**ENSG00000133313**	CNDP2	-0.281156938	5.723488885	8.813069046	0.0043553	0.0115156
**ENSG00000143374**	MIR6878	-0.517938703	4.196287675	8.81305247	0.0043553	0.0115156
**ENSG00000173451**	THAP2	-0.36117631	5.192889844	8.808874989	0.0043640	0.0115355
**ENSG00000168286**	THAP11	0.371470095	4.9681345	8.808342638	0.0043651	0.0115355

**ENSG00000076321**	KLHL20	-0.352649486	5.370845412	8.802874983	0.0043764	0.0115625
**ENSG00000196497**	IPO4	-0.474008355	4.500104668	8.801681381	0.0043789	0.0115660
**ENSG00000138035**	PNPT1	-0.467394298	5.614417996	8.962438981	0.0043799	0.0115660
**ENSG00000148341**	SH3GLB2	-0.489309594	4.815910305	8.798673846	0.0043851	0.0115768
**ENSG00000168394**	TAP1	-0.24316075	6.880110165	8.796733378	0.0043892	0.0115845
**ENSG00000100441**	KHNYN	-0.260259788	6.816197601	8.792136673	0.0043988	0.0116069
**ENSG00000135049**	AGTPBP1	-0.272920803	6.515402914	8.79120696	0.0044007	0.0116091
**ENSG00000107951**	MIR7162	-0.304375598	5.424793984	8.790457659	0.0044023	0.0116103
**ENSG00000219626**	FAM228B	-0.595408971	4.051130239	8.787881204	0.0044076	0.0116216
**ENSG00000224186**		-0.419339013	5.398001917	8.79172038	0.0044120	0.0116301
**ENSG00000279344**		-0.27092462	6.854407833	8.784606995	0.0044145	0.0116338
**ENSG00000138031**	ADCY3	0.401014884	4.895808943	8.782448914	0.0044190	0.0116427
**ENSG00000173744**	AGFG1	0.216915475	7.139819238	8.78069139	0.0044227	0.0116495
**ENSG00000173083**	HPSE	0.416901832	4.192604064	8.775513754	0.0044336	0.0116747
**ENSG00000177700**	POLR2L	0.663173232	3.757607198	8.775079201	0.0044345	0.0116747
**ENSG00000052841**	TTC17	-0.303708339	7.277946554	8.901422976	0.0044359	0.0116754
**ENSG00000006194**	ZNF263	-0.368377754	5.22512842	8.77306075	0.0044388	0.0116800
**ENSG00000178927**	c17orf62	-0.312799514	6.050840661	8.769939356	0.0044453	0.0116944
**ENSG00000163781**	TOPBP1	-0.284115864	6.456593327	8.769340609	0.0044466	0.0116948
**ENSG00000009954**	BAZ1B	0.210967643	7.653246292	8.766682119	0.0044522	0.0117040
**ENSG00000252835**	SCARNA21	0.781827622	6.785926754	9.198070707	0.0044523	0.0117040
**ENSG00000239521**	GATS	-0.523306076	4.436651364	8.765500593	0.0044547	0.0117067
**ENSG00000196074**	SYCP2	-0.57772463	4.041557534	8.764570536	0.0044567	0.0117067
**ENSG00000104731**	LOC105371397	-0.419919519	5.506629692	8.799139836	0.0044567	0.0117067
**ENSG00000211794**		0.578342612	3.758598837	8.758021295	0.0044706	0.0117401
**ENSG00000258989**		0.347354834	5.471738945	8.757044493	0.0044726	0.0117426
**ENSG00000269656**	GLTSCR2-AS1	0.482578251	5.001491022	8.800815443	0.0044741	0.0117434
**ENSG00000205765**	C5orf51	0.25012339	6.139554561	8.752565331	0.0044822	0.0117612
**ENSG00000163463**	KRTCAP2	0.394781619	5.143499916	8.752125003	0.0044831	0.0117612
**ENSG00000227939**		0.707732974	3.467935809	8.771592989	0.0044935	0.0117843
**ENSG00000273841**	TAF9	-0.350192854	5.168200508	8.746928156	0.0044942	0.0117843
**ENSG00000177954**	RPS27	0.432822815	10.0801411	9.153008406	0.0044967	0.0117881
**ENSG00000074319**	TSG101	0.369310402	5.072381114	8.742731149	0.0045031	0.0118019
**ENSG00000119396**	RAB14	0.223214466	6.844994307	8.741292609	0.0045062	0.0118070
**ENSG00000082269**	FAM135A	-0.49802641	4.590787905	8.729808476	0.0045309	0.0118687
**ENSG00000100258**	LMF2	-0.549190741	5.267068616	8.934001056	0.0045322	0.0118692
**ENSG00000185024**	BRF1	-0.412462738	4.898458135	8.722392913	0.0045469	0.0119046

**ENSG00000090020**	SLC9A1	0.487672354	4.462039056	8.720762903	0.0045504	0.0119108
**ENSG00000140395**	WDR61	-0.455780564	4.683672923	8.71814603	0.0045561	0.0119227
**ENSG00000170903**	MSANTD4	0.380279153	5.001825707	8.717217392	0.0045581	0.0119249
**ENSG00000002919**	SNX11	-0.398532956	4.784287025	8.715072183	0.0045627	0.0119341
**ENSG00000182117**	NOP10	0.572856477	4.702336882	8.818333173	0.0045657	0.0119368
**ENSG00000162066**	AMDHD2	-0.67898262	3.692522672	8.713545348	0.0045660	0.0119368
**ENSG00000106153**	CHCHD2	0.449668221	4.984838185	8.708142441	0.0045778	0.0119645
**ENSG00000256618**	MTRNR2L1	1.006712625	5.419058855	9.127008676	0.0045910	0.0119961
**ENSG00000184203**	PPP1R2	-0.220666124	7.583929173	8.691224788	0.0046147	0.0120535
**ENSG00000282246**		-0.350592452	5.113973394	8.690989886	0.0046153	0.0120535
**ENSG00000198783**	ZNF830	-0.375024104	4.936058543	8.689319142	0.0046189	0.0120600
**ENSG00000062716**	MIR21	0.358208904	7.009259691	8.938770514	0.0046232	0.0120683
**ENSG00000226849**		-0.457211925	4.509731098	8.686480426	0.0046252	0.0120703
**ENSG00000093100**		0.658448752	3.730724386	8.679529935	0.0046405	0.0121073
**ENSG00000010803**	SCMH1	0.351757499	5.042439134	8.677844744	0.0046442	0.0121118
**ENSG00000135976**	ANKRD36	-0.382080614	7.497370632	9.012736234	0.0046446	0.0121118
**ENSG00000107854**	TNKS2	0.170333246	7.499019441	8.675610675	0.0046491	0.0121208
**ENSG00000244462**	rbm12	0.203788036	7.299336345	8.671908023	0.0046573	0.0121362
**ENSG00000099783**	HNRNPM	0.202867466	7.686281684	8.67189794	0.0046574	0.0121362
**ENSG00000120314**	WDR55	-0.332332639	5.532374285	8.668682025	0.0046645	0.0121517
**ENSG00000138326**	RPS24	0.390510238	9.416129291	9.048110432	0.0046666	0.0121541
**ENSG00000211767**		0.477776868	4.337626585	8.662577764	0.0046781	0.0121810
**ENSG00000196235**	SUPT5H	-0.236000132	6.268834465	8.661712767	0.0046800	0.0121830
**ENSG00000169964**	TMEM42	-0.624510314	3.631838935	8.646279738	0.0047145	0.0122697
**ENSG00000218537**		0.68122119	5.328258044	9.002700496	0.0047196	0.0122799
**ENSG00000136940**	PDCL	0.508268738	4.394638849	8.642449709	0.0047231	0.0122860
**ENSG00000159140**	MIR6501	0.159529285	9.717441173	8.63992307	0.0047288	0.0122977
**ENSG00000057935**	mta3	-0.442472529	4.833741273	8.634228114	0.0047416	0.0123281
**ENSG00000109762**	SNX25	0.403020683	4.870656302	8.633618342	0.0047430	0.0123286
**ENSG00000236051**	MYCBP2-AS1	0.224495673	6.666050936	8.623003641	0.0047670	0.0123880
**ENSG00000176973**	FAM89B	0.515373339	4.472926274	8.620658389	0.0047724	0.0123988
**ENSG00000156931**	VPS8	-0.290432681	6.611367583	8.619199252	0.0047757	0.0124043
**ENSG00000103426**	CORO7-PAM16	-0.437618142	6.320712752	8.887626747	0.0047785	0.0124086
**ENSG00000281028**	PI4K2B	0.434171378	4.523266031	8.6145968	0.0047862	0.0124254
**ENSG00000162599**	NFIA	0.548246569	4.348925932	8.601899014	0.0048152	0.0124965
**ENSG00000135446**	CDK4	0.402051864	4.680349214	8.601576604	0.0048160	0.0124965
**ENSG00000065427**	KARS	0.24179348	6.350256826	8.596469798	0.0048277	0.0125208

**ENSG00000205045**	SLFN12L	0.296520477	7.482965268	8.744231159	0.0048277	0.0125208
**ENSG00000273217**	RAPGEF6	-0.205585089	8.176075992	8.594488641	0.0048323	0.0125295
**ENSG00000002016**	RAD52	-0.428297695	5.068913938	8.590073944	0.0048424	0.0125527
**ENSG00000176731**	C8orf59	-0.396353668	5.532197776	8.588832801	0.0048453	0.0125570
**ENSG00000132406**	TMEM128	-0.469830198	3.808831652	8.58832764	0.0048465	0.0125570
**ENSG00000148835**	TAF5	0.477863537	4.739572089	8.587800164	0.0048477	0.0125570
**ENSG00000107372**	ZFAND5	0.209489381	6.934165649	8.584986721	0.0048542	0.0125708
**ENSG00000163806**	spdya	0.336890948	6.296439839	8.644427712	0.0048615	0.0125864
**ENSG00000145623**	OSMR	-0.532245641	3.936694868	8.580509245	0.0048646	0.0125914
**ENSG00000131100**	ATP6V1E1	0.282037536	5.660615281	8.577590209	0.0048714	0.0126058
**ENSG00000163755**	HPS3	0.281218186	6.02864438	8.576783962	0.0048732	0.0126075
**ENSG00000111215**	PRR4	-0.517593896	4.209093585	8.574777338	0.0048779	0.0126165
**ENSG00000136451**	VEZF1	0.232548514	6.907183105	8.574107258	0.0048794	0.0126174
**ENSG00000261644**		-0.528626496	3.957768123	8.562962084	0.0049055	0.0126815
**ENSG00000104714**	erich1	-0.325871648	6.172823343	8.553049683	0.0049287	0.0127385
**ENSG00000161939**	RNASEK-C17orf	0.353258997	5.82694294	8.549714724	0.0049485	0.0127866
**ENSG00000168246**	UBTD2	0.483426867	3.802601218	8.540670877	0.0049579	0.0128076
**ENSG00000154723**	ATP5J	0.520169545	4.609856436	8.542056197	0.0049658	0.0128244
**ENSG00000187764**	SEMA4D	-0.18660602	8.668859699	8.536892051	0.0049669	0.0128244
**ENSG00000038210**	PI4K2B	0.327007356	5.199124829	8.534284266	0.0049731	0.0128372
**ENSG00000177189**	RPS6KA3	0.16221458	8.40717387	8.532154611	0.0049781	0.0128471
**ENSG00000165684**	SNAPC4	-0.577065737	3.742846272	8.529122568	0.0049853	0.0128625
**ENSG00000132471**	WBP2	0.308277516	5.979074798	8.52485542	0.0049955	0.0128856
**ENSG00000167393**	PPP2R3B	-0.704173742	3.488658782	8.522118131	0.0050020	0.0128992
**ENSG00000138085**	ATRAID	0.344663273	5.10114743	8.521145652	0.0050043	0.0129021
**ENSG00000015153**	YAF2	0.312966458	5.842047106	8.518178198	0.0050114	0.0129161
**ENSG00000204316**	MRPL38	-0.47900872	4.498130263	8.517834529	0.0050123	0.0129161
**ENSG00000120709**	fam53c	-0.449563091	4.35018588	8.514555991	0.0050201	0.0129332
**ENSG00000266094**	RASSF5	0.192418036	8.101001101	8.513160328	0.0050235	0.0129357
**ENSG00000197321**	svil	-0.242277904	6.46890466	8.513105311	0.0050236	0.0129357
**ENSG00000241553**	ARPC4	0.275268243	6.248235435	8.503559256	0.0050466	0.0129895
**ENSG00000166439**	RNF169	0.247249446	6.723681191	8.503394298	0.0050470	0.0129895
**ENSG00000258959**		0.336448318	5.093332637	8.498512046	0.0050587	0.0130166
**ENSG00000102879**	coro1a	0.259068461	8.028725436	8.555529937	0.0050914	0.0130974
**ENSG00000132768**	DPH2	-0.576957261	3.618136234	8.480222143	0.0051032	0.0131244
**ENSG00000152455**	SUV39H2	-0.531653465	4.039012511	8.475888461	0.0051137	0.0131484
**ENSG00000219747**		0.813289873	3.447014604	8.603142774	0.0051274	0.0131804

**ENSG00000004455**	AK2	-0.290018793	5.88044229	8.468310448	0.0051323	0.0131869
**ENSG00000211786**		0.520412627	4.274899382	8.468232679	0.0051325	0.0131869
**ENSG00000078269**	SYNJ2	-0.322424041	5.598256088	8.464056173	0.0051427	0.0132100
**ENSG00000183665**	TRMT12	-0.639381858	3.514620503	8.463158844	0.0051450	0.0132124
**ENSG00000056277**	ZNF280C	0.429713161	4.583594494	8.461522099	0.0051490	0.0132195
**ENSG00000283774**		-0.455277964	4.109571038	8.459258382	0.0051546	0.0132298
**ENSG00000181929**	PRKAG1	0.337823364	4.912161672	8.458872418	0.0051555	0.0132298
**ENSG00000100109**	TFIP11	0.31497802	5.585623745	8.457539515	0.0051588	0.0132350
**ENSG00000117569**	PTBP2	-0.412149248	5.757616664	8.540281919	0.0051650	0.0132478
**ENSG00000081913**	PHLPP1	0.47145755	3.848393429	8.452785037	0.0051705	0.0132586
**ENSG00000184840**	TMED9	0.410800767	4.886115766	8.445409262	0.0051888	0.0133013
**ENSG00000264058**	krt222	0.271108281	6.110006177	8.445047881	0.0051897	0.0133013
**ENSG00000080822**	CLDND1	0.266006361	7.294430665	8.476793559	0.0051910	0.0133014
**ENSG00000141564**	RPTOR	-0.290834752	6.187579925	8.44322801	0.0051942	0.0133063
**ENSG00000243678**	NME2	0.438796044	4.921657089	8.4317599	0.0052228	0.0133763
**ENSG00000156515**	HK1	0.27226783	6.444838235	8.428091646	0.0052320	0.0133965
**ENSG00000104946**	MIR4750	-0.434755182	4.972218834	8.421147641	0.0052494	0.0134349
**ENSG00000118058**	KMT2A	0.163433131	9.454377984	8.421082849	0.0052496	0.0134349
**ENSG00000274020**	LINC01138	-0.377449025	4.990424096	8.420085734	0.0052521	0.0134380
**ENSG00000239969**		-0.778271001	3.52816935	8.460746439	0.0052570	0.0134473
**ENSG00000139826**	ABHD13	0.267689046	6.15536014	8.417360428	0.0052589	0.0134490
**ENSG00000162664**	ZNF326	-0.384404657	6.086990463	8.53022476	0.0052744	0.0134853
**ENSG00000243749**	Tmem35b	0.405922697	4.40972757	8.41016428	0.0052771	0.0134888
**ENSG00000004779**	NDUFAB1	0.57579456	4.021866923	8.397763057	0.0053085	0.0135659
**ENSG00000066583**	ISOC1	0.517409222	3.9053699	8.395394545	0.0053145	0.0135779
**ENSG00000113312**	TTC1	0.32540855	5.248820301	8.390434131	0.0053272	0.0136023
**ENSG00000171314**	PGAM1	0.432075878	4.318906923	8.390195866	0.0053278	0.0136023
**ENSG00000197530**	MIB2	-0.644280216	4.194531896	8.412871009	0.0053280	0.0136023
**ENSG00000134748**	PRPF38A	-0.209517658	6.808032888	8.377117293	0.0053613	0.0136806
**ENSG00000170515**	PA2G4	0.253838807	6.523428216	8.377116827	0.0053613	0.0136806
**ENSG00000078687**	TNRC6C	-0.192533784	7.784996469	8.372860092	0.0053722	0.0137052
**ENSG00000126067**	PSMB2	0.269852025	5.756556553	8.367398464	0.0053863	0.0137378
**ENSG00000047457**	CP	0.463907645	4.403766926	8.364970935	0.0053926	0.0137504
**ENSG00000182986**	ZNF320	-0.412165263	4.687612269	8.362796079	0.0053982	0.0137614
**ENSG00000067365**	METTL22	-0.56284316	4.607070321	8.413813051	0.0054002	0.0137632
**ENSG00000168405**		-0.415482947	5.368788254	8.359623717	0.0054067	0.0137764
**ENSG00000264538**		-0.479459336	5.281064556	8.459499875	0.0054146	0.0137932

**ENSG00000204348**	DXO	-0.635954796	3.464461836	8.355101359	0.0054182	0.0137988
**ENSG00000149761**	NUDT22	-0.719486703	3.502964576	8.354382732	0.0054200	0.0138002
**ENSG00000112096**	LOC100129518	0.210272986	7.14783922	8.350510732	0.0054301	0.0138225
**ENSG00000101152**	DNAJC5	0.26891727	5.732616154	8.349072254	0.0054338	0.0138287
**ENSG00000253607**		0.551239504	3.40724408	8.34444609	0.0054459	0.0138560
**ENSG00000238121**	LINC00426	0.392644946	5.36998593	8.342903611	0.0054499	0.0138629
**ENSG00000269590**		0.367669037	4.816830966	8.338395682	0.0054617	0.0138895
**ENSG00000268750**		-0.624999203	3.35073153	8.334382709	0.0054723	0.0139118
**ENSG00000157014**	TATDN2	0.212493116	6.586848714	8.33354151	0.0054745	0.0139118
**ENSG00000137496**	IL18BP	-0.432437516	5.257956121	8.35359832	0.0054745	0.0139118
**ENSG00000232869**		0.450306827	4.657132726	8.331543256	0.0054797	0.0139217
**ENSG00000125912**	NCLN	-0.497183524	4.47146982	8.325585608	0.0054954	0.0139582
**ENSG00000224383**	PRR29	0.335051769	5.30886185	8.322855472	0.0055026	0.0139731
**ENSG00000101782**	RIOK3	-0.233400096	6.475572144	8.318073039	0.0055153	0.0140018
**ENSG00000125447**	GGA3	-0.302550161	5.870071749	8.316878729	0.0055184	0.0140064
**ENSG00000086619**	ERO1B	-0.434350716	5.133031525	8.302514543	0.0055566	0.0140999
**ENSG00000173456**	RNF26	0.549700829	3.8555967	8.295069582	0.0055765	0.0141469
**ENSG00000135334**	AKIRIN2	0.317076231	5.248066587	8.290045151	0.0055900	0.0141777
**ENSG00000136518**	ACTL6A	-0.354880729	5.315759929	8.289093955	0.0055925	0.0141807
**ENSG00000186517**	ARHGAP30	0.184878897	7.881540521	8.288367236	0.0055945	0.0141822
**ENSG00000139974**	SLC38A6	-0.597255023	4.149380822	8.287437366	0.0055970	0.0141851
**ENSG00000123200**	ZC3H13	0.248835096	7.807801906	8.323743904	0.0055993	0.0141876
**ENSG00000126903**	SLC10A3	0.654248915	3.728377782	8.280333019	0.0056161	0.0142267
**ENSG00000163599**	CTLA4	0.459580058	5.105055965	8.293243045	0.0056355	0.0142725
**ENSG00000071462**	WBSCR22	-0.35653397	5.104724823	8.268397281	0.0056484	0.0143016
**ENSG00000259595**		0.422677857	4.472910198	8.267819161	0.0056500	0.0143021
**ENSG00000001461**	NIPAL3	-0.235545385	6.840908459	8.258139315	0.0056763	0.0143653
**ENSG00000185507**	IRF7	-0.591980426	4.040788763	8.254660838	0.0056858	0.0143859
**ENSG00000031003**	FAM13B	0.235520319	6.893821708	8.254089585	0.0056874	0.0143863
**ENSG00000196787**	Hist1h2ag	0.765967889	4.629869765	8.552220002	0.0056964	0.0144056
**ENSG00000134198**	TSPAN2	0.748278517	3.759092842	8.359526233	0.0057085	0.0144329
**ENSG00000166377**	ATP9B	-0.267235952	6.522190898	8.24422969	0.0057144	0.0144442
**ENSG00000215041**	neurl4	-0.52211755	4.341860453	8.241860426	0.0057209	0.0144572
**ENSG00000148411**	NACC2	0.488861481	4.151842846	8.238997122	0.0057288	0.0144736
**ENSG00000138835**	RGS3	0.517939557	3.84367404	8.233020554	0.0057453	0.0145117
**ENSG00000237550**		0.607612373	7.153106809	8.611661188	0.0057557	0.0145345
**ENSG00000282851**	BISPR	-0.482190531	4.377733126	8.221506356	0.0057772	0.0145853

**ENSG00000119523**	ALG2	0.355552136	4.66048045	8.219478865	0.0057828	0.0145960
**ENSG00000110719**	TCIRG1	-0.654689593	5.474352636	8.55803311	0.0057851	0.0145981
**ENSG00000148688**	RPP30	-0.392925526	5.0712015	8.214292075	0.0057973	0.0146254
**ENSG00000161267**	BDH1	-0.395874741	4.793519195	8.210974562	0.0058065	0.0146452
**ENSG00000141384**	TAF4B	-0.51188774	4.535948738	8.204438439	0.0058248	0.0146878
**ENSG00000106459**	NRF1	0.290433095	5.18293752	8.19957399	0.0058385	0.0147187
**ENSG00000264187**		-0.539098566	3.742138517	8.19728513	0.0058449	0.0147304
**ENSG00000171657**	GPR82	0.761692864	3.921162258	8.324841266	0.0058459	0.0147304
**ENSG00000144935**	TRPC1	-0.591504493	3.545540303	8.196320419	0.0058476	0.0147311
**ENSG00000283341**		0.557677209	3.443096237	8.19512687	0.0058510	0.0147334
**ENSG00000198382**	UVRAG	0.312255431	5.27885056	8.194999842	0.0058513	0.0147334
**ENSG00000171204**	TMEM126B	-0.365335438	5.063069967	8.192013546	0.0058598	0.0147510
**ENSG00000108107**	MIR6805	0.380056377	9.579053764	8.530168325	0.0058648	0.0147603
**ENSG00000092203**	TOX4	-0.22365107	6.466215892	8.187488982	0.0058725	0.0147761
**ENSG00000116771**	AGMAT	-0.62052093	3.570085279	8.185401244	0.0058784	0.0147874
**ENSG00000144152**	fbln7	0.551328595	3.640283751	8.184744953	0.0058803	0.0147885
**ENSG00000100239**	PPP6R2	-0.358113509	6.050091278	8.241287103	0.0058855	0.0147979
**ENSG00000231721**	LINC-PINT	-0.362826617	6.716805092	8.370599388	0.0058895	0.0148045
**ENSG00000115282**	TTC31	-0.50183892	4.79667702	8.208447698	0.0058965	0.0148149
**ENSG00000134001**	EIF2S1	0.273302362	6.149523728	8.179038466	0.0058965	0.0148149
**ENSG00000159314**	ARHGAP27	-0.369987908	5.440144176	8.174919192	0.0059082	0.0148391
**ENSG00000151806**	GUF1	-0.293498114	5.838053812	8.174661102	0.0059089	0.0148391
**ENSG00000122218**	COPA	0.210046636	7.07532101	8.163782407	0.0059400	0.0149134
**ENSG00000249141**		-0.272509518	6.202413432	8.162582455	0.0059434	0.0149185
**ENSG00000175611**	LINC00476	-0.457660202	4.010905154	8.147308116	0.0059873	0.0150251
**ENSG00000110074**	FOXRED1	-0.603405987	3.671821659	8.143410251	0.0059986	0.0150488
**ENSG00000068024**	HDAC4	0.248021002	6.330269171	8.143033857	0.0059996	0.0150488
**ENSG00000149600**	COMMD7	0.459115363	4.747511771	8.136250123	0.0060193	0.0150945
**ENSG00000173674**	EIF1AX	0.21740788	6.527849128	8.12896812	0.0060405	0.0151440
**ENSG00000165006**	UBAP1	0.321850721	5.365536067	8.122775661	0.0060585	0.0151856
**ENSG00000075975**	MKRN2	-0.312375173	5.148709885	8.119861632	0.0060670	0.0152033
**ENSG00000196683**	TOMM7	0.415039297	6.317745511	8.360640861	0.0060727	0.0152139
**ENSG00000173141**	MRPL57	0.492137761	4.384728031	8.11494321	0.0060815	0.0152321
**ENSG00000178761**	FAM219B	-0.382981357	5.474727073	8.110283208	0.0060951	0.0152627
**ENSG00000186020**	ZNF529	-0.296149975	5.82356939	8.105294495	0.0061098	0.0152959
**ENSG00000182359**	kbtbd3	0.492228096	4.099553097	8.10092714	0.0061227	0.0153244
**ENSG00000142208**	AKT1	0.30580836	5.561607401	8.099090374	0.0061281	0.0153344

**ENSG00000142534**	RPS11	0.350159778	9.779779191	8.393110746	0.0061426	0.0153669
**ENSG00000130749**	ZC3H4	0.273386788	6.122511457	8.091527522	0.0061506	0.0153831
**ENSG00000164663**	USP49	-0.563728552	4.053014188	8.086186011	0.0061664	0.0154191
**ENSG00000152443**	ZNF776	-0.316079802	5.523953337	8.080003008	0.0061849	0.0154615
**ENSG00000189362**	NEMP2	-0.338231761	5.762026425	8.076921376	0.0061941	0.0154808
**ENSG00000186635**	ARAP1	-0.253593761	6.210327597	8.072680686	0.0062068	0.0155088
**ENSG00000093183**	SEC22C	-0.279584017	5.829916924	8.067322342	0.0062228	0.0155453
**ENSG00000108960**	MMD	0.60812351	3.946372399	8.063186445	0.0062353	0.0155726
**ENSG00000260404**		-0.510123869	5.053143782	8.151749662	0.0062545	0.0156168
**ENSG00000188033**	ZNF490	0.439307665	4.614974328	8.056185857	0.0062564	0.0156179
**ENSG00000164284**	grpel2	0.273154524	6.386181995	8.051224349	0.0062714	0.0156517
**ENSG00000245970**		0.454879655	9.085186556	8.412832485	0.0062734	0.0156529
**ENSG00000168792**	ABHD15	0.486375729	4.177908544	8.049025606	0.0062781	0.0156608
**ENSG00000181035**	SLC25A42	-0.531846477	3.899106005	8.048128709	0.0062808	0.0156639
**ENSG00000132467**	UTP3	0.349424996	5.162771445	8.044023896	0.0062933	0.0156912
**ENSG00000165995**	CACNB2	-0.602818868	3.833992198	8.038395448	0.0063104	0.0157302
**ENSG00000058272**	PPP1R12A	0.196550157	8.25325455	8.022733096	0.0063584	0.0158446
**ENSG00000107798**	lipA	0.328890317	5.652961122	8.0224158	0.0063593	0.0158446
**ENSG00000085872**	CHERP	-0.258315293	5.765050142	8.020105829	0.0063664	0.0158586
**ENSG00000139323**	POC1B	0.379856478	5.169560169	8.018858155	0.0063703	0.0158643
**ENSG00000260719**		-0.637228361	3.620415927	8.017443967	0.0063746	0.0158714
**ENSG00000141469**	SLC14A1	-0.757469048	3.9689726	8.115482433	0.0063777	0.0158752
**ENSG00000242028**	HYPK	-0.393590647	5.004018712	8.01548097	0.0063807	0.0158789
**ENSG00000149218**	ENDOD1	0.473885203	4.538516238	8.012982498	0.0063884	0.0158943
**ENSG00000099331**	MYO9B	-0.231822409	7.399917725	8.010060304	0.0063974	0.0159130
**ENSG00000234616**	JRK	-0.372638351	4.883979161	8.008159706	0.0064033	0.0159239
**ENSG00000120802**	TMPO	0.203789942	7.774953008	7.998859593	0.0064322	0.0159919
**ENSG00000099341**	PSMD8	0.325179715	4.917893366	7.993682637	0.0064483	0.0160282
**ENSG00000074582**	Bcs1l	-0.569973667	4.349324756	7.996964208	0.0064609	0.0160557
**ENSG00000213598**		0.774893763	3.686932895	8.134845966	0.0064829	0.0161063
**ENSG00000272325**	NUDT3	0.205900056	7.015298149	7.979642935	0.0064923	0.0161259
**ENSG00000197061**	hist1h4c	0.921716601	4.251341512	8.271025413	0.0065536	0.0162744
**ENSG00000107560**	RAB11FIP2	-0.292485076	5.777731184	7.948208978	0.0065918	0.0163654
**ENSG00000114446**	IFT57	-0.39168066	4.738509744	7.946835357	0.0065962	0.0163724
**ENSG00000135423**	GLS2	-0.527664625	3.732645053	7.943249898	0.0066077	0.0163970
**ENSG00000088682**	COQ9	-0.580149079	3.997673182	7.940344661	0.0066170	0.0164162
**ENSG00000126709**	IFI6	0.471038518	4.289069831	7.938328219	0.0066235	0.0164284

**ENSG00000176390**	CRLF3	-0.215284508	7.201166128	7.935551623	0.0066324	0.0164466
**ENSG00000198198**	SZT2	-0.286571481	7.22672206	8.024813491	0.0066477	0.0164807
**ENSG00000108219**	TSPAN14	-0.170544012	7.750891951	7.929041099	0.0066533	0.0164907
**ENSG00000166479**	TMX3	-0.255424029	6.733810053	7.927849747	0.0066572	0.0164963
**ENSG00000170089**	LOC728554	-0.518044846	4.254145904	7.922250442	0.0066753	0.0165372
**ENSG00000167977**	KCTD5	0.513592939	3.776237439	7.919372915	0.0066846	0.0165564
**ENSG00000115459**	ELMOD3	-0.464669129	4.706734762	7.918812401	0.0066864	0.0165570
**ENSG00000134313**	KIDINS220	0.184146779	7.398274025	7.90606288	0.0067279	0.0166557
**ENSG00000185019**	UBOX5	-0.499154127	3.943398722	7.901470227	0.0067429	0.0166889
**ENSG00000119953**	SMNDC1	0.235089084	6.099737176	7.898828666	0.0067515	0.0167063
**ENSG00000185829**	ARL17A	-0.511845316	4.335068635	7.886009964	0.0067936	0.0168065
**ENSG00000143507**	DUSP10	0.579760479	3.820049164	7.87939962	0.0068154	0.0168565
**ENSG00000227081**		0.61678383	6.609091648	8.225610129	0.0068306	0.0168900
**ENSG00000174744**	brms1	-0.495187734	3.653084706	7.868089678	0.0068529	0.0169413
**ENSG00000122376**	FAM35A	0.301564083	5.628506665	7.864927601	0.0068635	0.0169633
**ENSG00000176148**	TCP11L1	0.337153698	4.788803619	7.859767471	0.0068807	0.0170018
**ENSG00000146535**	GNA12	0.460384988	4.086045352	7.858692851	0.0068843	0.0170067
**ENSG00000071127**	WDR1	0.171835604	7.626984044	7.842016687	0.0069402	0.0171409
**ENSG00000212127**	TAS2R14	-0.652437844	3.895135548	7.840654332	0.0069448	0.0171482
**ENSG00000277957**	SENP3	0.178709906	7.602391471	7.838088586	0.0069535	0.0171655
**ENSG00000151718**	WWC2	-0.624205221	3.745055736	7.836378435	0.0069593	0.0171757
**ENSG00000184381**	PLA2G6	-0.612720616	4.591817537	7.944292881	0.0069878	0.0172419
**ENSG00000089639**	GMIP	-0.283070446	5.707392379	7.827197503	0.0069904	0.0172444
**ENSG00000065665**	SEC61A2	-0.469693106	4.696042513	7.823608734	0.0070026	0.0172704
**ENSG00000164975**	SNAPC3	-0.254840087	6.816907616	7.822759042	0.0070055	0.0172734
**ENSG00000105607**	GCDH	-0.565004884	3.698068324	7.8214614	0.0070099	0.0172803
**ENSG00000046653**	GPM6B	-0.618594522	4.459669658	7.905337775	0.0070258	0.0173153
**ENSG00000180626**	ZNF594	-0.598817822	4.224224573	7.84293869	0.0070289	0.0173189
**ENSG00000091009**	RBM27	0.219823432	7.201238069	7.814044843	0.0070352	0.0173304
**ENSG00000269243**		0.281297646	5.305553292	7.806583724	0.0070608	0.0173893
**ENSG00000179933**	c14orf119	0.382023191	4.862751204	7.80297881	0.0070732	0.0174157
**ENSG00000213442**		0.571568601	5.986989323	8.133095508	0.0070755	0.0174172
**ENSG00000117906**	RCN2	-0.266470777	5.551673357	7.799629575	0.0070847	0.0174358
**ENSG00000263276**		0.311940634	5.682086149	7.796841134	0.0070943	0.0174554
**ENSG00000271730**		-0.527475078	4.123054744	7.79303933	0.0071074	0.0174836
**ENSG00000130695**	CEP85	-0.419819309	4.607353945	7.790085957	0.0071176	0.0175046
**ENSG00000215012**	C22orf29	-0.377946733	4.846350228	7.789213923	0.0071207	0.0175079

**ENSG00000210112**		-0.542921056	4.19114686	7.7854373	0.0071338	0.0175359
**ENSG00000114737**	CISH	0.658382661	6.939515509	8.127390474	0.0071442	0.0175576
**ENSG00000142875**	PRKACB	0.214626788	8.345194746	7.781825088	0.0071463	0.0175585
**ENSG00000155313**	USP25	0.224027323	7.491029509	7.779421467	0.0071547	0.0175749
**ENSG00000181652**	ATG9B	-0.637529428	3.945993757	7.772878187	0.0071778	0.0176276
**ENSG00000137411**	VARS2	-0.478466654	4.371001573	7.767141462	0.0071975	0.0176720
**ENSG00000226438**		0.466807968	4.074444237	7.76581451	0.0072022	0.0176792
**ENSG00000052749**	RRP12	-0.545487451	3.737684167	7.764536157	0.0072067	0.0176861
**ENSG00000037749**	MFAP3	0.378466396	4.692333365	7.763428101	0.0072105	0.0176913
**ENSG00000172273**	HINFP	-0.381777526	4.817258523	7.762967956	0.0072122	0.0176913
**ENSG00000065809**	FAM107B	0.193143643	8.117610037	7.759327612	0.0072250	0.0177185
**ENSG00000096433**	ITPR3	-0.33532447	7.194133411	7.963238316	0.0072281	0.0177212
**ENSG00000166987**	mbd6	-0.462468395	5.258790164	7.842659245	0.0072309	0.0177212
**ENSG00000180354**	MTURN	-0.450407183	4.155856071	7.757568612	0.0072311	0.0177212
**ENSG00000188186**	LAMTOR4	-0.455616752	4.632077995	7.752920649	0.0072475	0.0177553
**ENSG00000148384**	INPP5E	-0.538398669	4.016030181	7.752656138	0.0072485	0.0177553
**ENSG00000122484**	RPAP2	-0.313641587	6.552526047	7.817272659	0.0072504	0.0177560
**ENSG00000156502**	SUPV3L1	-0.371270943	5.172187581	7.750327451	0.0072567	0.0177671
**ENSG00000140543**	DET1	-0.547239158	4.219787415	7.736796816	0.0073046	0.0178775
**ENSG00000282320**		0.571718331	3.455040363	7.736645924	0.0073052	0.0178775
**ENSG00000088205**	DDX18	-0.214832124	7.144995935	7.734055145	0.0073144	0.0178959
**ENSG00000160633**	SAFB	-0.21525815	6.875944699	7.730971659	0.0073254	0.0179186
**ENSG00000160688**	FLAD1	-0.54654843	3.929888405	7.727797105	0.0073367	0.0179421
**ENSG00000135250**	SRPK2	0.209718767	7.0080192	7.71243045	0.0073919	0.0180727
**ENSG00000185129**	PURA	0.214162656	7.211634967	7.711701571	0.0073945	0.0180749
**ENSG00000165156**	ZHX1	0.306698186	5.699499902	7.710520262	0.0073987	0.0180806
**ENSG00000204619**	PPP1R11	0.320311248	5.66715346	7.710098325	0.0074003	0.0180806
**ENSG00000166747**	AP1G1	0.178856149	7.293409134	7.703786341	0.0074231	0.0181321
**ENSG00000109670**	FBXW7	0.246466073	6.825599308	7.702542062	0.0074276	0.0181368
**ENSG00000197324**	LRP10	0.226809371	6.874462249	7.702295896	0.0074285	0.0181368
**ENSG00000143418**	CERS2	0.26563216	6.290810085	7.695797366	0.0074520	0.0181901
**ENSG00000129353**	SLC44A2	-0.227218577	7.33379155	7.691927063	0.0074661	0.0182202
**ENSG00000214022**	REPIN1	-0.350291359	4.732117366	7.689101045	0.0074764	0.0182410
**ENSG00000100991**	TRPC4AP	-0.276247241	5.736618288	7.682817395	0.0074993	0.0182927
**ENSG00000212802**		0.516098629	5.602244605	7.934298609	0.0075135	0.0183231
**ENSG00000166275**	BORCS7	-0.449703465	4.517936873	7.675032135	0.0075279	0.0183538
**ENSG00000228363**		-0.395814226	4.880413048	7.667692116	0.0075549	0.0184153

**ENSG00000170871**	KIAA0232	0.265543117	5.920062529	7.660952941	0.0075798	0.0184717
**ENSG00000166225**	FRS2	0.257279584	5.914081949	7.659571511	0.0075849	0.0184798
**ENSG00000127995**	CASD1	-0.253674845	6.181449624	7.657983872	0.0075908	0.0184898
**ENSG00000225630**		0.45715236	8.106562641	7.987308701	0.0075974	0.0184992
**ENSG00000087448**	KLHL42	-0.371091559	5.127876368	7.655994494	0.0075981	0.0184992
**ENSG00000126003**	PLAGL2	0.358902286	5.414493372	7.649995406	0.0076204	0.0185491
**ENSG00000083799**	CYLD	-0.167851871	9.25444567	7.648358838	0.0076265	0.0185596
**ENSG00000143321**	HDGF	0.325526906	5.573969778	7.647194258	0.0076308	0.0185658
**ENSG00000172977**	KAT5	-0.313373027	5.318850655	7.645285498	0.0076379	0.0185769
**ENSG00000186376**	ZNF75D	-0.34189655	5.165299103	7.645014974	0.0076390	0.0185769
**ENSG00000140564**	FURIN	0.442751138	4.56731201	7.637359983	0.0076676	0.0186422
**ENSG00000088038**	CNOT3	-0.291980202	5.992188885	7.62977615	0.0076960	0.0187053
**ENSG00000225032**	LOC102723566	-0.751491706	3.629348033	7.67320277	0.0076971	0.0187053
**ENSG00000226803**		-0.359414959	4.860325467	7.621392849	0.0077276	0.0187717
**ENSG00000048544**	MRPS10	0.32571725	5.042424683	7.621277761	0.0077280	0.0187717
**ENSG00000197857**	ZNF44	-0.265342451	5.749074725	7.619999349	0.0077328	0.0187791
**ENSG00000117614**	SYF2	0.272307046	6.325604872	7.617943121	0.0077406	0.0187936
**ENSG00000171608**	PIK3CD	-0.21206748	7.671108334	7.616317196	0.0077468	0.0188041
**ENSG00000100027**	YPEL1	0.439884919	4.515496403	7.605730291	0.0077869	0.0188973
**ENSG00000165591**	FAAH2	-0.528978833	3.879418214	7.604124818	0.0077931	0.0189077
**ENSG00000272367**		-0.408916011	4.561666881	7.597641583	0.0078178	0.0189633
**ENSG00000203705**	TATDN3	-0.571484251	3.825588524	7.589722516	0.0078481	0.0190325
**ENSG00000137822**	TUBGCP4	-0.266283475	5.974242679	7.589155688	0.0078503	0.0190333
**ENSG00000096401**	CDC5L	0.222398878	6.405423384	7.584878266	0.0078667	0.0190687
**ENSG00000177239**	MAN1B1	-0.408593698	5.777461215	7.697446724	0.0078857	0.0191102
**ENSG00000015479**	MATR3	0.159423383	9.113815288	7.574464813	0.0079069	0.0191572
**ENSG00000187325**	TAF9B	0.290396119	5.651643903	7.566794632	0.0079366	0.0192248
**ENSG00000213462**	ERV3-1	-0.516824005	3.954231518	7.56320667	0.0079505	0.0192541
**ENSG00000183530**	PRR14L	0.207884096	7.099136499	7.562605355	0.0079529	0.0192553
**ENSG00000157693**	TMEM268	-0.476815324	4.420356429	7.562015836	0.0079552	0.0192564
**ENSG00000076662**	ICAM3	0.265384261	6.18998394	7.559226573	0.0079660	0.0192742
**ENSG00000071205**	ARHGAP10	0.590030745	3.926793382	7.559176101	0.0079662	0.0192742
**ENSG00000263731**		0.553108654	3.423254151	7.556535717	0.0079765	0.0192947
**ENSG00000122512**	PMS2	-0.412620236	4.513978225	7.550320084	0.0080008	0.0193490
**ENSG00000115042**	FAHD2A	-0.713271747	4.070435556	7.665638876	0.0080050	0.0193546
**ENSG00000269929**		-0.576338042	3.649304865	7.54721517	0.0080130	0.0193655
**ENSG00000247315**	ZCCHC3	0.381672739	4.993884897	7.547161163	0.0080132	0.0193655

**ENSG00000139370**	SLC15A4	0.482454427	4.249153465	7.546448949	0.0080160	0.0193678
**ENSG00000174903**	RAB1B	0.322751885	5.21236793	7.542028102	0.0080334	0.0194052
**ENSG00000107789**	MINPP1	0.453456046	3.648434318	7.540301355	0.0080402	0.0194172
**ENSG00000166200**	COPS2	0.227035527	6.709165849	7.538711896	0.0080464	0.0194278
**ENSG00000143702**	CEP170	-0.300184849	5.621611324	7.534896925	0.0080615	0.0194596
**ENSG00000210196**		-0.374556271	6.798479386	7.758507814	0.0080783	0.0194958
**ENSG00000185963**	BICD2	0.251116119	5.691103919	7.529751543	0.0080818	0.0194997
**ENSG00000075151**	EIF4G3	0.277551838	6.050074719	7.528950413	0.0080850	0.0195005
**ENSG00000282939**		0.590829274	4.113303894	7.553588059	0.0080859	0.0195005
**ENSG00000260565**		-0.321062375	6.171412117	7.553130279	0.0080951	0.0195183
**ENSG00000197302**	LOC107983990	-0.286751924	5.48413627	7.524907596	0.0081010	0.0195280
**ENSG00000114391**	RPL24	0.411985017	8.821400556	7.835777243	0.0081139	0.0195545
**ENSG00000136631**	VPS45	-0.395709667	5.09717776	7.517466771	0.0081306	0.0195902
**ENSG00000064419**	TNPO3	0.227667587	6.357047526	7.513695646	0.0081456	0.0196219
**ENSG00000125485**	DDX31	-0.479137956	4.569985214	7.5131919	0.0081476	0.0196223
**ENSG00000169217**	CD2BP2	-0.368849335	4.863959465	7.511400418	0.0081548	0.0196350
**ENSG00000163930**	BAP1	-0.300626546	5.236143927	7.510662274	0.0081577	0.0196375
**ENSG00000137193**	PIM1	0.38201748	7.266986551	7.783929863	0.0081837	0.0196947
**ENSG00000162341**	TPCN2	-0.668761105	3.876697215	7.534509076	0.0081857	0.0196947
**ENSG00000130723**	PRRC2B	-0.198219521	8.216916322	7.503316474	0.0081871	0.0196947
**ENSG00000134153**	EMC7	0.545006705	3.947320951	7.501455704	0.0081946	0.0197082
**ENSG00000145817**	YIPF5	0.325295065	5.600769319	7.49338611	0.0082271	0.0197817
**ENSG00000136021**	SCYL2	0.239894122	6.482360071	7.491354745	0.0082353	0.0197969
**ENSG00000174748**	RPL15	0.217186322	9.189372093	7.488471056	0.0082469	0.0198203
**ENSG00000116337**	AMPD2	-0.408097961	5.006385526	7.485343662	0.0082596	0.0198462
**ENSG00000269086**		-0.424453777	4.790103176	7.479474913	0.0082834	0.0198988
**ENSG00000076685**	NT5C2	-0.263931498	5.95566875	7.476613758	0.0082950	0.0199221
**ENSG00000274211**	SOCS7	-0.440496218	4.298592151	7.474821943	0.0083023	0.0199351
**ENSG00000143669**	LYST	0.250014597	7.072031272	7.471480687	0.0083159	0.0199632
**ENSG00000125503**	PPP1R12C	-0.592181904	5.029790786	7.670633993	0.0083308	0.0199898
**ENSG00000101940**	WDR13	-0.5417724	3.75857207	7.467827189	0.0083308	0.0199898
**ENSG00000197329**	PELI1	-0.277458479	5.779412499	7.465979166	0.0083384	0.0200033
**ENSG00000099949**	LZTR1	-0.422567819	5.154843082	7.464429895	0.0083451	0.0200148
**ENSG00000168813**	ZNF507	-0.207553859	6.858253406	7.462905494	0.0083509	0.0200243
**ENSG00000130775**	THEMIS2	0.285204515	5.270148018	7.459095007	0.0083666	0.0200572
**ENSG00000124207**	CSE1L	0.218142862	6.545991892	7.450659642	0.0084013	0.0201352
**ENSG00000153879**	CEBPG	0.331983438	5.189827098	7.450252709	0.0084029	0.0201352

**ENSG00000142528**	ZNF473	-0.575063689	3.818927773	7.448567549	0.0084099	0.0201472
**ENSG00000164609**	SLU7	0.276811845	6.420937156	7.447982399	0.0084123	0.0201484
**ENSG00000143365**	rorc	0.593324545	4.199343528	7.483587154	0.0084167	0.0201528
**ENSG00000234511**	C5orf58	-0.293090415	5.77287342	7.446604229	0.0084180	0.0201528
**ENSG00000185379**	RAD51D	-0.4098356	4.82087353	7.442679215	0.0084342	0.0201870
**ENSG00000255857**	PXN-AS1	0.305123216	5.302558504	7.440657955	0.0084426	0.0202024
**ENSG00000118515**	SGK1	0.606046688	3.668672257	7.43753423	0.0084556	0.0202288
**ENSG00000178988**	MRFAP1L1	0.236164223	6.550086419	7.429845305	0.0084875	0.0203007
**ENSG00000100393**	MIR1281	0.160018752	8.327071725	7.426487553	0.0085015	0.0203295
**ENSG00000136878**	USP20	-0.255517182	6.494928582	7.412145349	0.0085616	0.0204685
**ENSG00000128534**	LSM 8.00	-0.26025937	5.959901492	7.410934357	0.0085667	0.0204760
**ENSG00000174996**	KLC2	-0.444997408	3.594400163	7.403260616	0.0085991	0.0205487
**ENSG00000111711**	GOLT1B	-0.278358531	5.517625082	7.402508246	0.0086023	0.0205493
**ENSG00000176593**	LOC100128398	-0.445036199	4.440447951	7.402264565	0.0086033	0.0205493
**ENSG00000034152**	MAP2K3	0.382446148	4.493703617	7.400052446	0.0086126	0.0205670
**ENSG00000100138**	SNU13	0.324886185	5.682957457	7.398356055	0.0086198	0.0205794
**ENSG00000244038**	DDOST	0.283010663	5.473907815	7.397211111	0.0086247	0.0205863
**ENSG00000213699**	SLC35F6	0.422341303	4.981850223	7.393186082	0.0086418	0.0206224
**ENSG00000006744**	ELAC2	-0.299599857	5.540698759	7.389633131	0.0086569	0.0206537
**ENSG00000125482**	TTF1	-0.343967928	5.083180443	7.388998268	0.0086596	0.0206554
**ENSG00000225470**	Jpx	-0.337687798	6.27749533	7.480550984	0.0086628	0.0206585
**ENSG00000119041**	GTF3C3	-0.277646032	5.689100132	7.380610751	0.0086953	0.0207313
**ENSG00000161618**	ALDH16A1	-0.659837543	3.867814435	7.397030793	0.0086983	0.0207336
**ENSG00000052723**	SIKE1	-0.224806684	6.399115839	7.379200594	0.0087014	0.0207362
**ENSG00000166750**	SLFN5	0.172769849	9.783847177	7.373249464	0.0087269	0.0207922
**ENSG00000245571**	LOC101927204	-0.548893197	3.497264088	7.36535531	0.0087608	0.0208684
**ENSG00000281490**		-0.732448559	3.699879687	7.418798625	0.0087911	0.0209358
**ENSG00000198231**	DDX42	-0.200076311	7.187449166	7.351960521	0.0088187	0.0209967
**ENSG00000164442**	CITED2	0.269846286	6.086455854	7.351079679	0.0088225	0.0210011
**ENSG00000135624**	CCT7	0.25478745	6.326708458	7.348304518	0.0088346	0.0210250
**ENSG00000105829**	BET1	-0.465706592	4.294423432	7.340121681	0.0088702	0.0211050
**ENSG00000278963**		-0.614397543	4.004383946	7.345793733	0.0088726	0.0211059
**ENSG00000177932**	ZNF354C	-0.369315067	4.699755455	7.338941654	0.0088754	0.0211077
**ENSG00000227766**		0.291469049	7.595656585	7.492043275	0.0088879	0.0211327
**ENSG00000160199**	PKNOX1	0.309871384	5.125964096	7.32983604	0.0089153	0.0211928
**ENSG00000111737**	RAB35	0.326180237	4.873549898	7.327927439	0.0089237	0.0212079
**ENSG00000138029**	HADHB	-0.262373016	5.785549865	7.324643842	0.0089381	0.0212374

**ENSG00000005889**	ZFX	0.182167318	7.470085931	7.321300048	0.0089528	0.0212676
**ENSG00000175324**	LSM 1.00	0.499584444	4.003987162	7.320459154	0.0089565	0.0212716
**ENSG00000169951**	ZNF764	0.477166629	4.294280243	7.311528117	0.0089960	0.0213561
**ENSG00000168096**	ANKS3	-0.656901903	3.88637506	7.33310863	0.0089962	0.0213561
**ENSG00000171223**	JUNB	0.517879026	6.051961678	7.58405919	0.0090078	0.0213789
**ENSG00000139597**	N4BP2L1	-0.281485722	5.573569142	7.307850325	0.0090123	0.0213846
**ENSG00000144161**	ZC3H8	-0.37849454	5.845643907	7.398227977	0.0090180	0.0213933
**ENSG00000203896**	lime1	0.472011322	5.196760626	7.392144391	0.0090392	0.0214386
**ENSG00000151413**	NUBPL	-0.569634825	3.424402914	7.295299522	0.0090682	0.0215026
**ENSG00000204397**	CARD16	0.413737002	4.408711012	7.292961482	0.0090787	0.0215225
**ENSG00000204196**		0.413735625	5.423590469	7.367066685	0.0090893	0.0215429
**ENSG00000164933**	SLC25A32	0.306986267	5.427857645	7.283006778	0.0091233	0.0216186
**ENSG00000261552**		-0.387694557	4.583796899	7.275347901	0.0091578	0.0216954
**ENSG00000225339**		0.226242291	6.537976708	7.2747857	0.0091604	0.0216965
**ENSG00000206028**		-0.756459985	4.667906317	7.501223546	0.0091860	0.0217523
**ENSG00000274925**		-0.551937927	3.560415275	7.265665543	0.0092017	0.0217845
**ENSG00000095209**	TMEM38B	-0.593908954	3.764780285	7.262162692	0.0092176	0.0218172
**ENSG00000104904**	OAZ1	0.220628887	6.902349769	7.261000795	0.0092228	0.0218248
**ENSG00000131508**	UBE2D2	0.214875362	6.454510797	7.259547756	0.0092295	0.0218355
**ENSG00000196505**	GDAP2	0.289526266	6.170362	7.25672558	0.0092423	0.0218609
**ENSG00000135124**	P2RX4	-0.385031822	5.031859944	7.24741087	0.0092849	0.0219567
**ENSG00000066044**	ELAVL1	0.282720806	5.753200974	7.246911934	0.0092872	0.0219571
**ENSG00000256229**	ZNF486	0.413557762	4.417236071	7.246349072	0.0092897	0.0219582
**ENSG00000124333**	VAMP7	0.331155127	5.156727375	7.242018316	0.0093096	0.0220002
**ENSG00000136286**	MYO1G	0.293460147	7.272429582	7.370421811	0.0093442	0.0220752
**ENSG00000115128**	SF3B6	0.480271859	4.531720979	7.233872812	0.0093471	0.0220752
**ENSG00000104388**	RAB2A	0.257869267	6.211142245	7.233750836	0.0093477	0.0220752
**ENSG00000122299**	ZC3H7A	-0.230605702	6.667352594	7.228307719	0.0093728	0.0221296
**ENSG00000257065**		0.485685212	3.867348883	7.215504071	0.0094323	0.0222649
**ENSG00000247627**		0.597914347	6.354523165	7.508211064	0.0094501	0.0223020
**ENSG00000118046**	STK11	-0.316137927	5.032777508	7.20939596	0.0094608	0.0223221
**ENSG00000272368**		-0.59942491	3.42278583	7.205466038	0.0094792	0.0223604
**ENSG00000114745**	GORASP1	-0.508961826	3.888650124	7.196562009	0.0095209	0.0224539
**ENSG00000184178**	SCFD2	-0.509085838	4.206144087	7.186519748	0.0095683	0.0225606
**ENSG00000283977**		-0.410712744	4.21266676	7.18417624	0.0095794	0.0225807
**ENSG00000279407**		-0.570401493	3.533187767	7.183805446	0.0095811	0.0225807
**ENSG00000172794**	RAB37	-0.426345459	4.732837559	7.17915247	0.0096032	0.0226275

**ENSG00000102178**	UBL4A	0.474944517	4.141750521	7.175070395	0.0096226	0.0226653
**ENSG00000106868**	SUSD1	0.554582938	3.849082134	7.174871287	0.0096236	0.0226653
**ENSG00000144655**	CSRNP1	0.489890834	3.740888366	7.160400896	0.0096927	0.0228229
**ENSG00000177879**	AP3S1	0.425278497	4.766862198	7.158561634	0.0097015	0.0228339
**ENSG00000166136**	NDUFB8	-0.289362927	5.408390582	7.158516833	0.0097017	0.0228339
**ENSG00000091483**	FH	-0.370779954	4.388680476	7.156538822	0.0097112	0.0228511
**ENSG00000156052**	GNAQ	0.197253326	6.882253981	7.155120632	0.0097180	0.0228620
**ENSG00000143149**	ALDH9A1	0.256003348	5.642729565	7.153072594	0.0097279	0.0228800
**ENSG00000182405**	PGBD4	-0.563166897	3.744342718	7.151498906	0.0097354	0.0228927
**ENSG00000130303**	BST2	0.468127202	4.022984474	7.147260464	0.0097559	0.0229356
**ENSG00000118420**	UBE3D	-0.636883229	3.77643671	7.146070982	0.0097616	0.0229439
**ENSG00000096060**	FKBP5	-0.229212207	7.839507211	7.163195827	0.0097850	0.0229936
**ENSG00000006652**	IFRD1	-0.307081549	5.61656438	7.136178545	0.0098095	0.0230461
**ENSG00000148943**	LIN7C	0.256055369	6.853485252	7.139480243	0.0098221	0.0230706
**ENSG00000140280**	LYSMD2	0.43230067	4.357663713	7.132763714	0.0098261	0.0230748
**ENSG00000132383**	RPA1	0.251434102	6.287804842	7.131115958	0.0098341	0.0230884
**ENSG00000268220**		-0.270311586	5.694460246	7.128489213	0.0098469	0.0231132
**ENSG00000228060**	LOC101929516	-0.361157922	4.605264092	7.126710187	0.0098556	0.0231284
**ENSG00000171311**	EXOSC1	-0.301241507	5.158909567	7.124094961	0.0098684	0.0231532
**ENSG00000105323**	HNRNPUL1	0.184113357	7.914227398	7.12312652	0.0098731	0.0231591
**ENSG00000210144**		0.367959059	9.17488505	7.392729427	0.0098902	0.0231936
**ENSG00000169019**	COMMD8	0.449590214	4.073580127	7.119217706	0.0098922	0.0231936
**ENSG00000116922**	C1ORF109	-0.533562721	3.646415966	7.116823603	0.0099040	0.0232159
**ENSG00000181045**	SLC26A11	-0.799787991	4.449763044	7.338220232	0.0099116	0.0232287
**ENSG00000168434**	COG7	-0.415134076	4.598488511	7.11361472	0.0099197	0.0232424
**ENSG00000145293**	ENOPH1	0.343200212	4.870961165	7.112332646	0.0099260	0.0232519
**ENSG00000077312**	SNRPA	-0.393534984	4.771901039	7.102068514	0.0099766	0.0233652
**ENSG00000244720**		-0.660342728	4.322392355	7.217144031	0.0099876	0.0233857
**ENSG00000270157**		-0.646153504	3.897423932	7.12142205	0.0100184	0.0234526
**ENSG00000129534**	MIS18BP1	0.276904865	6.206589548	7.088985198	0.0100415	0.0235013
**ENSG00000175087**	PDIK1L	0.357119659	5.168436229	7.086300123	0.0100548	0.0235274
**ENSG00000171940**	ZNF217	0.170041737	7.947154188	7.083651013	0.0100681	0.0235530
**ENSG00000196458**	znf605	-0.418439139	4.888672224	7.083078069	0.0100709	0.0235544
**ENSG00000100360**	IFT27	-0.636632437	3.86100204	7.081775963	0.0100774	0.0235644
**ENSG00000163001**	CFAP36	-0.318220937	5.487039938	7.078377932	0.0100944	0.0235988
**ENSG00000113119**	TMCO6	-0.446267362	4.344419791	7.070337276	0.0101347	0.0236878
**ENSG00000097096**	SYDE2	-0.557941728	3.875760623	7.068332816	0.0101448	0.0237060

**ENSG00000256223**	ZNF10	-0.474671025	4.151277249	7.064775085	0.0101627	0.0237407
**ENSG00000270022**		0.811061982	4.744787228	7.342114476	0.0101642	0.0237407
**ENSG00000080947**	CROCCP3	-0.502415147	4.870960638	7.135691987	0.0101809	0.0237733
**ENSG00000132341**	RAN	0.224095553	6.978371551	7.060813394	0.0101827	0.0237733
**ENSG00000135315**	CEP162	-0.389874713	4.712947583	7.058937715	0.0101922	0.0237902
**ENSG00000123737**	EXOSC9	-0.274300594	5.886418157	7.055653572	0.0102088	0.0238236
**ENSG00000135828**	RNASEL	-0.29996115	5.274345288	7.047957192	0.0102478	0.0239094
**ENSG00000176978**	DPP7	-0.439901615	4.857281862	7.042828974	0.0102743	0.0239659
**ENSG00000139687**	RB1	0.273213852	6.161714632	7.03784887	0.0102994	0.0240189
**ENSG00000165672**	PRDX3	0.374238836	5.252544654	7.036508174	0.0103062	0.0240295
**ENSG00000160741**	CRTC2	-0.294631455	5.501550852	7.034908557	0.0103144	0.0240433
**ENSG00000108433**	GOSR2	-0.251538385	6.407592335	7.031696432	0.0103309	0.0240763
**ENSG00000256646**	PSMA2	0.33827325	5.513852993	7.028284645	0.0103484	0.0241117
**ENSG00000160131**	VMA21	0.248797942	6.513688157	7.025983771	0.0103602	0.0241326
**ENSG00000187189**	TSPYL4	-0.37667376	4.953778346	7.0256406	0.0103620	0.0241326
**ENSG00000031698**	SARS	0.222952337	6.318094017	7.02067922	0.0103875	0.0241867
**ENSG00000034510**	TMSB10	0.329785889	7.800512572	7.252339675	0.0103904	0.0241879
**ENSG00000180901**	KCTD2	0.310656004	5.467234777	7.011755395	0.0104337	0.0242833
**ENSG00000263126**		-0.402166416	4.652750643	7.008298532	0.0104516	0.0243197
**ENSG00000243927**	mrps6	0.328341727	5.169053419	7.00206963	0.0104840	0.0243896
**ENSG00000153827**	TRIP12	0.161468317	8.504202226	7.001057651	0.0104892	0.0243913
**ENSG00000154127**	UBASH3B	0.290801555	5.928601822	7.001037013	0.0104894	0.0243913
**ENSG00000140463**	BBS4	-0.54514152	3.985764396	6.999225716	0.0104988	0.0244078
**ENSG00000187144**	SPATA21	0.382096782	4.423467358	6.995964338	0.0105158	0.0244419
**ENSG00000179454**	KLHL28	0.192131816	6.761910287	6.988158236	0.0105567	0.0245315
**ENSG00000240972**	MIF	0.652683283	5.288572953	7.240268837	0.0105702	0.0245575
**ENSG00000113013**	HSPA9	0.180716624	7.266440024	6.984320397	0.0105768	0.0245674
**ENSG00000199568**	RNU5A-1	1.203389051	6.899435423	7.263212847	0.0105927	0.0245989
**ENSG00000284526**		0.300347696	5.983979315	6.978397144	0.0106080	0.0246289
**ENSG00000160194**	NDUFV3	-0.31964191	5.052606805	6.976876387	0.0106161	0.0246420
**ENSG00000109323**	MANBA	-0.36565956	4.799123077	6.972005325	0.0106418	0.0246940
**ENSG00000182253**	SYNM	0.669593327	3.840610285	7.020449235	0.0106432	0.0246940
**ENSG00000105982**	RNF32	-0.628430399	3.745528614	6.970725304	0.0106486	0.0247011
**ENSG00000136874**	STX17	-0.322126776	5.491462974	6.964539423	0.0106814	0.0247717
**ENSG00000167491**	GATAD2A	0.236210823	6.742901495	6.963474076	0.0106870	0.0247793
**ENSG00000151116**	UEVLD	0.462037362	4.096495665	6.96261071	0.0106916	0.0247797
**ENSG00000173812**	EIF1	0.169598145	8.629086964	6.962551853	0.0106919	0.0247797

**ENSG00000143919**	CAMKMT	-0.635062707	4.042519622	6.98817767	0.0106984	0.0247893
**ENSG00000066697**	MSANTD3	0.465470021	3.563138758	6.960003814	0.0107055	0.0248001
**ENSG00000132780**	NASP	-0.216340533	6.337545474	6.958364309	0.0107142	0.0248148
**ENSG00000123154**	WDR83	-0.392192077	4.429479377	6.956668589	0.0107233	0.0248303
**ENSG00000196290**	NIF3L1	-0.455193846	4.273351869	6.954095609	0.0107370	0.0248566
**ENSG00000136891**	TEX10	-0.267483938	6.128889427	6.951121325	0.0107529	0.0248847
**ENSG00000145782**	ATG12	-0.252273214	6.559752881	6.950928999	0.0107539	0.0248847
**ENSG00000100811**	MIR6764	0.189512593	7.389167399	6.949584719	0.0107611	0.0248927
**ENSG00000166401**	SERPINB8	0.410403712	4.17660541	6.949392527	0.0107621	0.0248927
**ENSG00000141506**	PIK3R5	0.216168244	6.818566554	6.942957559	0.0107967	0.0249670
**ENSG00000276027**	rnu12	0.855060351	4.679239835	7.209907127	0.0108223	0.0250208
**ENSG00000151503**	NCAPD3	-0.301223124	5.819033873	6.934705719	0.0108411	0.0250587
**ENSG00000170502**	NUDT9	-0.45323763	4.053713484	6.933916327	0.0108453	0.0250630
**ENSG00000158793**	NIT1	-0.38729061	4.766510875	6.932121341	0.0108550	0.0250799
**ENSG00000140553**	UNC45A	0.288052233	5.085743656	6.926616844	0.0108848	0.0251431
**ENSG00000255443**		0.309486711	5.519262748	6.92469081	0.0108953	0.0251617
**ENSG00000168685**	IL7R	0.208280366	11.3776886	6.919629136	0.0109228	0.0252196
**ENSG00000196081**		0.483861787	4.149321137	6.917759388	0.0109329	0.0252375
**ENSG00000124541**	RRP36	0.323228396	4.702691465	6.914320557	0.0109517	0.0252752
**ENSG00000117625**	RCOR3	-0.226986298	6.710398149	6.912041482	0.0109641	0.0252983
**ENSG00000166260**	cox11	-0.292063534	5.475048832	6.911253617	0.0109684	0.0253027
**ENSG00000148154**	UGCG	0.280829077	6.013775943	6.903635725	0.0110101	0.0253932
**ENSG00000105497**	ZNF175	-0.35263621	4.413286345	6.903076867	0.0110132	0.0253947
**ENSG00000013561**	RNF14	0.318862686	5.115939489	6.895410795	0.0110553	0.0254863
**ENSG00000145781**	COMMD10	-0.354496243	4.859936238	6.891983772	0.0110742	0.0255242
**ENSG00000226950**	DANCR	-0.368909285	4.603832757	6.890168998	0.0110842	0.0255417
**ENSG00000279089**		-0.684119962	3.698167123	6.930824659	0.0110885	0.0255459
**ENSG00000103994**	ZNF106	0.201247257	7.078538732	6.887683739	0.0110980	0.0255621
**ENSG00000129317**	PUS7L	-0.218923784	6.503869354	6.876237367	0.0111615	0.0257027
**ENSG00000147050**	KDM6A	0.209024598	7.043108484	6.875413551	0.0111661	0.0257075
**ENSG00000135052**	GOLM1	-0.529535547	3.734134083	6.870370676	0.0111942	0.0257666
**ENSG00000237854**		0.53659329	3.366392829	6.868664064	0.0112037	0.0257829
**ENSG00000197062**	ZSCAN26	-0.389454215	4.597874841	6.867149104	0.0112122	0.0257967
**ENSG00000149212**	SESN3	-0.208458937	8.946576305	6.866642845	0.0112150	0.0257975
**ENSG00000124784**	RIOK1	-0.326140544	5.068416821	6.859595622	0.0112545	0.0258826
**ENSG00000198856**	OSTC	0.423470851	4.908872922	6.862863087	0.0112672	0.0259061
**ENSG00000106105**	GARS	0.219129759	6.000576706	6.853405998	0.0112893	0.0259513

**ENSG00000104635**	SLC39A14	0.49028929	3.304443687	6.852760961	0.0112929	0.0259539
**ENSG00000111676**	ATN1	-0.335033701	5.431451737	6.849458692	0.0113115	0.0259910
**ENSG00000163874**	MIR6732	0.480174663	4.070512324	6.848907782	0.0113146	0.0259924
**ENSG00000215030**		0.462963891	6.684184972	7.103416025	0.0113495	0.0260668
**ENSG00000165671**	NSD1	0.181166668	7.89517869	6.842295908	0.0113520	0.0260669
**ENSG00000108679**	LGALS3BP	-0.366889766	4.676308164	6.836489179	0.0113850	0.0261360
**ENSG00000073536**	NLE1	-0.555832874	3.728469118	6.836104281	0.0113872	0.0261360
**ENSG00000112308**	c6orf62	0.154000787	8.083404356	6.832935041	0.0114052	0.0261676
**ENSG00000132953**	XPO4	0.184169366	7.041934201	6.832808322	0.0114059	0.0261676
**ENSG00000267702**		0.586355805	4.328300848	6.885270137	0.0114415	0.0262436
**ENSG00000179409**	GEMIN4	-0.428232309	4.193279788	6.823093672	0.0114614	0.0262833
**ENSG00000110700**	RPS13	0.418571616	9.230160036	7.085409292	0.0114807	0.0263218
**ENSG00000235453**	TOPORS-AS1	-0.579621709	3.431094028	6.818451948	0.0114880	0.0263327
**ENSG00000185834**		0.57201651	4.11483267	6.852225035	0.0114973	0.0263483
**ENSG00000261684**		-0.38468913	4.744263418	6.809815758	0.0115376	0.0264349
**ENSG00000185664**	PMEL	-0.469630651	4.929839572	6.845158759	0.0115688	0.0264978
**ENSG00000175575**	PAAF1	-0.54079773	3.77469105	6.804186941	0.0115701	0.0264978
**ENSG00000210140**		0.382232067	8.855890684	7.058644077	0.0115948	0.0265485
**ENSG00000233927**	RPS28	0.409913939	7.327882267	7.049900049	0.0116299	0.0266230
**ENSG00000064601**	CTSA	0.363364665	5.182114732	6.787472215	0.0116671	0.0267025
**ENSG00000173473**	SMARCC1	0.203934859	7.386870426	6.784045076	0.0116871	0.0267424
**ENSG00000182481**	KPNA2	0.480139347	4.178031602	6.782347528	0.0116971	0.0267592
**ENSG00000204120**	GIGYF2	0.177504529	7.252576224	6.780356398	0.0117087	0.0267800
**ENSG00000147174**	ACRC	-0.694789881	3.698387399	6.808954247	0.0117245	0.0268102
**ENSG00000146066**	HIGD2A	0.419309468	5.192350666	6.825070741	0.0117285	0.0268135
**ENSG00000271980**		0.447426781	4.097723629	6.77560901	0.0117365	0.0268261
**ENSG00000115947**	ORC4	-0.22587871	6.29436328	6.771692655	0.0117595	0.0268728
**ENSG00000181026**	AEN	0.390633442	4.656471916	6.770226091	0.0117682	0.0268866
**ENSG00000100652**	SLC10A1	0.615523436	3.435278079	6.757482366	0.0118434	0.0270508
**ENSG00000066739**	atg2b	-0.221723632	6.940691885	6.757175774	0.0118452	0.0270508
**ENSG00000162888**		-0.560571678	3.487343284	6.756263945	0.0118506	0.0270530
**ENSG00000196177**	ACADSB	-0.310834697	5.609084868	6.756146099	0.0118513	0.0270530
**ENSG00000154122**	ANKH	0.198293661	7.143864988	6.755586401	0.0118546	0.0270543
**ENSG00000183688**	RFLNB	0.23553643	6.468248811	6.75517315	0.0118571	0.0270543
**ENSG00000198900**	TOP 1.00	0.238788198	6.870184864	6.753737073	0.0118656	0.0270673
**ENSG00000187066**	TMEM262	-0.565160326	3.675588429	6.7532765	0.0118684	0.0270673
**ENSG00000154767**	XPC	-0.211517496	6.399309214	6.752908367	0.0118705	0.0270673

**ENSG00000111678**	C12orf57	0.391751683	5.410234111	6.793838785	0.0118861	0.0270955
**ENSG00000085760**	MTIF2	-0.328070362	5.218979001	6.749951502	0.0118881	0.0270955
**ENSG00000167380**	ZNF226	-0.293216897	5.434243706	6.746903517	0.0119063	0.0271291
**ENSG00000104447**	TRPS1	0.274999693	5.880983098	6.746608715	0.0119080	0.0271291
**ENSG00000140264**	SERF2	0.247426298	6.690637358	6.74518278	0.0119165	0.0271425
**ENSG00000167193**	CRK	0.330905643	5.238583492	6.743779099	0.0119249	0.0271557
**ENSG00000122122**	SASH3	0.249505556	6.514618976	6.738919482	0.0119539	0.0272159
**ENSG00000136816**	TOR1B	0.340766903	4.661063674	6.732826686	0.0119904	0.0272931
**ENSG00000163110**	PDLIM5	0.277533829	5.554745929	6.732381475	0.0119931	0.0272932
**ENSG00000115685**	PPP1R7	0.287184496	5.073588598	6.723491192	0.0120466	0.0274090
**ENSG00000100897**	DCAF11	-0.255871401	5.772406356	6.715864838	0.0120927	0.0275079
**ENSG00000005339**	CREBBP	0.15466566	8.395867298	6.711382691	0.0121199	0.0275637
**ENSG00000062485**	CS	-0.208889222	6.764532904	6.710521206	0.0121251	0.0275652
**ENSG00000177426**	TGIF1	0.430355913	4.662992944	6.710404969	0.0121258	0.0275652
**ENSG00000113595**	TRIM23	-0.27248142	5.534118232	6.709329498	0.0121324	0.0275741
**ENSG00000087111**	PIGS	0.330086746	4.946002051	6.708025524	0.0121403	0.0275861
**ENSG00000123609**	NMI	0.313956368	5.327393794	6.705507501	0.0121556	0.0276132
**ENSG00000083845**	RPS5	0.380543828	8.154706335	6.95665414	0.0121575	0.0276132
**ENSG00000160410**	SHKBP1	0.328928208	5.41636045	6.698276578	0.0121997	0.0277031
**ENSG00000146083**	RNF44	-0.235113295	6.873354293	6.693874671	0.0122267	0.0277583
**ENSG00000182446**	NPLOC4	-0.249153107	6.264362671	6.689534441	0.0122533	0.0278127
**ENSG00000143811**	MIR6741	-0.32488825	5.022035046	6.688897804	0.0122572	0.0278155
**ENSG00000161011**	SQSTM1	0.192021853	6.457031697	6.686216806	0.0122737	0.0278460
**ENSG00000167548**	KMT2D	0.179064889	8.341899816	6.685850674	0.0122759	0.0278460
**ENSG00000228237**	EFCAB14-AS1	0.453098819	4.258846072	6.684621601	0.0122835	0.0278571
**ENSG00000150753**	CCT5	0.222831636	6.375887998	6.682152313	0.0122987	0.0278855
**ENSG00000133134**	BEX2	-0.497906123	3.6306445	6.67992149	0.0123125	0.0279107
**ENSG00000117640**	MTFR1L	-0.401428943	4.544738805	6.668196668	0.0123851	0.0280692
**ENSG00000145349**	CAMK2D	0.199955962	6.335434136	6.666934843	0.0123929	0.0280809
**ENSG00000100351**	GRAP2	-0.196107547	6.93248404	6.660451178	0.0124333	0.0281662
**ENSG00000205808**	plpp6	-0.383689187	4.547166558	6.655319521	0.0124654	0.0282327
**ENSG00000146701**	MDH2	0.268794967	5.709333729	6.654435835	0.0124709	0.0282391
**ENSG00000196236**	XPNPEP3	-0.445693023	5.227027387	6.730252498	0.0124753	0.0282430
**ENSG00000100528**	CNIH1	0.272902647	5.688965838	6.651768995	0.0124876	0.0282647
**ENSG00000255036**	LOC100499484-	-0.367169239	4.85929706	6.651084687	0.0124919	0.0282683
**ENSG00000176095**	IP6K1	0.347831586	4.871034307	6.648668138	0.0125070	0.0282965
**ENSG00000255642**		0.501936309	3.724569174	6.645220518	0.0125287	0.0283393

**ENSG00000177646**	ACAD9	-0.380991335	4.768657183	6.644696559	0.0125320	0.0283407
**ENSG00000167985**	SDHAF2	0.323711546	4.988872592	6.643957606	0.0125366	0.0283450
**ENSG00000115170**	ACVR1	0.322537742	5.399504119	6.642567236	0.0125454	0.0283574
**ENSG00000197312**	DDI2	0.181210996	6.808524681	6.642226139	0.0125475	0.0283574
**ENSG00000100982**	PCIF1	0.266777487	5.502117811	6.639874837	0.0125623	0.0283848
**ENSG00000255987**		0.396808729	4.609233982	6.638816418	0.0125690	0.0283937
**ENSG00000145743**	FBXL17	0.355485511	5.587832209	6.666382947	0.0125778	0.0284075
**ENSG00000259248**	USP3-AS1	-0.208079066	6.184120085	6.635504883	0.0125899	0.0284287
**ENSG00000160953**	MUM1	-0.466084106	4.574874641	6.627016092	0.0126437	0.0285439
**ENSG00000082516**	GEMIN5	0.303837136	5.069575156	6.624223323	0.0126615	0.0285778
**ENSG00000203668**	CHML	-0.384823046	5.030712478	6.622952125	0.0126695	0.0285899
**ENSG00000165119**	HNRNPK	0.139627084	8.978418572	6.617911314	0.0127017	0.0286562
**ENSG00000266076**		-0.497066622	4.247418615	6.616973869	0.0127076	0.0286589
**ENSG00000140993**	TIGD7	-0.446072271	4.039559119	6.616595898	0.0127100	0.0286589
**ENSG00000174292**	TNK1	-0.539380303	4.104117384	6.616437211	0.0127111	0.0286589
**ENSG00000100523**	DDHD1	-0.257757953	7.215763252	6.668825281	0.0127225	0.0286784
**ENSG00000081014**	AP4E1	0.25805317	5.515514045	6.614133014	0.0127258	0.0286797
**ENSG00000213443**		-0.211095602	6.739011683	6.607949477	0.0127654	0.0287627
**ENSG00000262560**		-0.454353123	3.870211184	6.599319487	0.0128209	0.0288815
**ENSG00000185989**	RASA3	-0.159493367	7.876676968	6.595932981	0.0128427	0.0289245
**ENSG00000164405**	Uqcrq	0.529105285	3.799673983	6.593129845	0.0128608	0.0289590
**ENSG00000160877**	NACC1	0.37186016	4.292741883	6.591567472	0.0128709	0.0289755
**ENSG00000161970**	RPL26	0.366662923	9.713754163	6.828415929	0.0128898	0.0290117
**ENSG00000116288**	PARK7	0.295505579	5.588465711	6.58450747	0.0129167	0.0290660
**ENSG00000066427**	ATXN3	-0.221809838	6.656829742	6.57984022	0.0129470	0.0291281
**ENSG00000183666**	GUSBP1	-0.556179494	4.284426536	6.59185343	0.0129626	0.0291568
**ENSG00000214265**	SNURF	-0.211307511	6.303485483	6.576752667	0.0129671	0.0291608
**ENSG00000126214**	KLC1	-0.242559627	7.205416002	6.595224857	0.0129973	0.0292188
**ENSG00000111790**	FGFR1OP2	0.186263424	7.156756963	6.571944729	0.0129985	0.0292188
**ENSG00000244625**		-0.571118288	4.767276726	6.677599833	0.0130858	0.0294086
**ENSG00000248487**	ABHD14A	0.45471142	4.113763741	6.553357614	0.0131206	0.0294806
**ENSG00000096872**	IFT74	-0.492354646	4.132082161	6.551752222	0.0131313	0.0294981
**ENSG00000153406**	NMRAL1	-0.409187712	4.333565694	6.550189787	0.0131416	0.0295150
**ENSG00000105088**	OLFM2	0.482326906	3.415289911	6.545485592	0.0131727	0.0295786
**ENSG00000143751**	SDE2	0.281594159	5.447994911	6.539979042	0.0132093	0.0296543
**ENSG00000168137**	SETD5	-0.206111885	7.57875157	6.539136856	0.0132149	0.0296592
**ENSG00000162924**	REL	0.235460518	6.868956592	6.538597667	0.0132185	0.0296592

**ENSG00000197816**	CCDC180	-0.423832044	4.677102772	6.537917397	0.0132230	0.0296592
**ENSG00000117118**	SDHB	0.353332675	5.093754811	6.537872473	0.0132233	0.0296592
**ENSG00000085433**	WDR47	0.392738828	4.435972763	6.537520201	0.0132257	0.0296592
**ENSG00000283149**		0.51769446	3.618371802	6.533781603	0.0132506	0.0297088
**ENSG00000117724**	CENPF	-0.703736476	3.984232077	6.638689086	0.0132630	0.0297301
**ENSG00000256164**	CCND2-AS1	0.464017007	4.123904871	6.529558027	0.0132788	0.0297592
**ENSG00000189339**	slc35e2b	-0.206723717	6.741455933	6.528402676	0.0132865	0.0297702
**ENSG00000137501**	SYTL2	0.264097516	6.080629483	6.52770639	0.0132912	0.0297711
**ENSG00000141084**	RANBP10	-0.323495094	5.197816662	6.527488159	0.0132926	0.0297711
**ENSG00000173960**	UBXN2A	0.362354753	4.578166371	6.52669332	0.0132980	0.0297741
**ENSG00000105085**	MED26	0.418251594	3.889919117	6.526440696	0.0132997	0.0297741
**ENSG00000143390**	RFX5	-0.319463137	5.231757712	6.525344757	0.0133070	0.0297842
**ENSG00000073050**	XRCC1	-0.455888273	4.573499052	6.522992238	0.0133228	0.0298131
**ENSG00000225636**		-0.556478042	3.540753068	6.522275588	0.0133276	0.0298175
**ENSG00000203965**	EFCAB7	-0.493420032	4.049662856	6.517452101	0.0133600	0.0298792
**ENSG00000242247**	ARFGAP3	0.283250929	5.479805926	6.517317891	0.0133609	0.0298792
**ENSG00000275342**	SGK223	-0.368852616	4.965825498	6.512712864	0.0133919	0.0299422
**ENSG00000140612**	SEC11A	0.292813686	5.627589617	6.50791992	0.0134243	0.0300082
**ENSG00000158864**	NDUFS2	-0.2825207	5.169729554	6.496646364	0.0135008	0.0301728
**ENSG00000176171**	BNIP3	-0.32771765	5.084793649	6.492092006	0.0135319	0.0302357
**ENSG00000116580**	GON4L	-0.16154154	7.088276682	6.485893891	0.0135742	0.0303238
**ENSG00000050748**	MAPK9	0.218482462	6.043442273	6.484868982	0.0135812	0.0303330
**ENSG00000180071**	ANKRD18A	-0.462942343	3.853412278	6.482473293	0.0135976	0.0303632
**ENSG00000268861**	ARHGEF18	-0.193901057	7.321129382	6.481511176	0.0136042	0.0303715
**ENSG00000257824**		0.440332939	4.114026244	6.479859396	0.0136156	0.0303903
**ENSG00000141552**	ANAPC11	0.514606734	3.360935167	6.479244204	0.0136198	0.0303932
**ENSG00000154640**	btg3	0.470990524	3.948547585	6.47710858	0.0136345	0.0304174
**ENSG00000134285**	FKBP11	-0.36089351	5.129406161	6.476823096	0.0136365	0.0304174
**ENSG00000108639**	SYNGR2	0.368409097	4.400603019	6.476267661	0.0136403	0.0304194
**ENSG00000176092**	AIM1L	0.514832	3.423914249	6.473358302	0.0136603	0.0304576
**ENSG00000270136**	MINOS1-NBL1	0.457372854	4.512843534	6.471117675	0.0136758	0.0304843
**ENSG00000136802**	LRRC8A	0.257391109	5.21767101	6.470690228	0.0136787	0.0304843
**ENSG00000182010**	RTKN2	-0.411548078	6.363414355	6.662787634	0.0136811	0.0304843
**ENSG00000171792**	RHNO1	0.34149486	4.689311517	6.465784133	0.0137126	0.0305469
**ENSG00000149591**	TAGLN	-0.605267023	3.497610639	6.464956602	0.0137183	0.0305469
**ENSG00000177105**	RHOG	0.347794607	5.22458783	6.464778299	0.0137196	0.0305469
**ENSG00000053501**	USE1	-0.55012645	3.777887021	6.464599878	0.0137208	0.0305469

**ENSG00000215492**		0.516390096	3.863383936	6.461585153	0.0137417	0.0305869
**ENSG00000227543**		-0.422699043	4.299438747	6.452114694	0.0138075	0.0307269
**ENSG00000238164**		-0.444169794	4.167355614	6.450402032	0.0138195	0.0307469
**ENSG00000113916**	BCL6	0.577712489	3.61018505	6.449436939	0.0138262	0.0307553
**ENSG00000096384**	HSP90AB1	0.179125607	8.753481259	6.448408096	0.0138334	0.0307648
**ENSG00000085978**	ATG16L1	-0.257711127	6.046775815	6.446941862	0.0138436	0.0307810
**ENSG00000111684**	LPCAT3	-0.317389911	5.129772067	6.445266252	0.0138553	0.0308005
**ENSG00000128335**	APOL2	-0.29962506	5.138942739	6.444820656	0.0138585	0.0308009
**ENSG00000144895**	EIF2A	-0.200637748	6.955677638	6.436773918	0.0139149	0.0309197
**ENSG00000142751**	GPN2	-0.354767325	4.586760458	6.434280314	0.0139324	0.0309521
**ENSG00000162402**	USP24	0.156173122	8.313892743	6.433248235	0.0139397	0.0309617
**ENSG00000182973**	CNOT10	-0.267984062	5.407261346	6.429033436	0.0139694	0.0310211
**ENSG00000213757**		0.627245627	5.332258778	6.654726097	0.0139882	0.0310562
**ENSG00000280143**		-0.305571158	6.290281478	6.473769287	0.0140006	0.0310771
**ENSG00000176749**	CDK5R1	-0.455287668	4.096273067	6.421237063	0.0140245	0.0311237
**ENSG00000225783**	Miat	-0.606505397	6.906856121	6.661787227	0.0140278	0.0311243
**ENSG00000133943**	c14orf159	-0.308722685	5.461554149	6.419768775	0.0140349	0.0311335
**ENSG00000143353**	LYPLAL1	-0.378414809	4.181139894	6.419348097	0.0140379	0.0311335
**ENSG00000232956**	Snhg15	-0.471782777	4.380166491	6.416273688	0.0140597	0.0311753
**ENSG00000006715**	VPS41	0.252663861	5.928836019	6.414035103	0.0140756	0.0312040
**ENSG00000065243**	PKN2	-0.21730453	6.619177701	6.411227579	0.0140956	0.0312417
**ENSG00000154957**	ZNF18	-0.537083896	3.705627992	6.408116252	0.0141178	0.0312842
**ENSG00000128513**	POT1	-0.336501808	5.052002149	6.40633146	0.0141306	0.0313058
**ENSG00000261771**	DYX1C1-CCPG1	0.255889415	5.679948289	6.403054831	0.0141540	0.0313511
**ENSG00000132740**	IGHMBP2	-0.412526718	4.322845661	6.399465255	0.0141797	0.0314014
**ENSG00000146109**	ABT1	0.326468611	4.973586612	6.39597081	0.0142048	0.0314502
**ENSG00000251992**		-0.52078811	6.926005735	6.633467311	0.0142088	0.0314526
**ENSG00000121988**	ZRANB3	-0.44039321	4.140656244	6.394805477	0.0142131	0.0314555
**ENSG00000259544**		0.302899343	5.151149764	6.392679365	0.0142284	0.0314826
**ENSG00000186063**	AIDA	0.228387866	5.850254694	6.38183206	0.0143067	0.0316491
**ENSG00000019995**	ZRANB1	0.211463898	6.188266151	6.380677851	0.0143151	0.0316609
**ENSG00000131943**	c19orf12	0.320735098	5.075475284	6.378761353	0.0143289	0.0316849
**ENSG00000008838**	MIR6884	-0.293819341	5.398465271	6.378161481	0.0143333	0.0316878
**ENSG00000123384**	LRP1	-0.45223072	4.153838656	6.370594833	0.0143883	0.0318027
**ENSG00000215190**	LINC00680	-0.573894029	3.741251656	6.37011408	0.0143918	0.0318037
**ENSG00000279767**		-0.500813129	4.136063669	6.36802181	0.0144070	0.0318306
**ENSG00000135870**	RC3H1	0.160405257	7.62295014	6.366677328	0.0144168	0.0318456

**ENSG00000267128**	RNF157-AS1	-0.485547951	3.975701565	6.364524306	0.0144325	0.0318736
**ENSG00000039319**	ZFYVE16	-0.318260605	5.51991712	6.36372843	0.0144383	0.0318797
**ENSG00000068120**	COASY	-0.403553796	4.412359071	6.362256332	0.0144491	0.0318939
**ENSG00000102931**	ARL2BP	0.266401564	5.74419256	6.362010745	0.0144509	0.0318939
**ENSG00000125449**	ARMC7	0.503499022	3.577932791	6.359637826	0.0144683	0.0319255
**ENSG00000173914**	RBM4B	-0.327051976	4.974402079	6.358445975	0.0144770	0.0319380
**ENSG00000137478**	FCHSD2	-0.216248822	6.389863564	6.355913407	0.0144956	0.0319723
**ENSG00000104783**	KCNN4	-0.522511174	3.866690189	6.354412615	0.0145066	0.0319898
**ENSG00000198498**	TMA16	-0.443499692	4.210788829	6.353240615	0.0145152	0.0320021
**ENSG00000101856**	PGRMC1	0.501369724	3.931253848	6.351221995	0.0145300	0.0320280
**ENSG00000162419**	GMEB1	0.36767225	4.871722304	6.349939156	0.0145395	0.0320421
**ENSG00000132478**	UNK	-0.289458468	5.324559607	6.346087841	0.0145679	0.0320979
**ENSG00000157741**	UBN2	-0.228917689	6.473803133	6.33975351	0.0146147	0.0321942
**ENSG00000116497**	S100PBP	0.254836321	6.229664286	6.332085833	0.0146716	0.0323127
**ENSG00000133275**	CSNK1G2	-0.262602831	5.696712155	6.328902263	0.0146952	0.0323580
**ENSG00000185000**	MIR6848	-0.363957531	4.950888045	6.327256166	0.0147075	0.0323740
**ENSG00000132361**	CLUH	-0.47189907	4.439196622	6.327097999	0.0147087	0.0323740
**ENSG00000137970**		0.506393539	6.11913587	6.552726088	0.0147121	0.0323747
**ENSG00000124496**	TRERF1	-0.213510135	6.318549805	6.325024983	0.0147241	0.0323880
**ENSG00000186704**		-0.547547239	4.014799345	6.324998268	0.0147243	0.0323880
**ENSG00000258704**		-0.565296602	3.636454717	6.324184371	0.0147304	0.0323946
**ENSG00000223803**		0.810531835	3.751130364	6.479131091	0.0147536	0.0324388
**ENSG00000200156**	RNU5B-1	1.240712895	6.766133989	6.552176421	0.0147771	0.0324836
**ENSG00000136854**	STXBP1	-0.527608045	3.75664847	6.315509836	0.0147953	0.0325141
**ENSG00000125875**	TBC1D20	-0.290975333	5.759493305	6.315254558	0.0147972	0.0325141
**ENSG00000093072**	CECR1	-0.183870508	7.065874052	6.312785613	0.0148158	0.0325480
**ENSG00000250251**		-0.742603477	3.803726371	6.407919343	0.0148392	0.0325927
**ENSG00000174749**	C4orf32	-0.513617115	4.374867105	6.327186535	0.0148493	0.0326080
**ENSG00000112511**	PHF1	-0.278384424	5.74340235	6.306644394	0.0148619	0.0326289
**ENSG00000272173**		0.378088775	5.087588931	6.303870498	0.0148829	0.0326680
**ENSG00000062822**	POLD1	-0.600280208	3.470211749	6.30159043	0.0149001	0.0326989
**ENSG00000159596**	TMEM69	-0.367390393	4.558913142	6.291744007	0.0149747	0.0328552
**ENSG00000100092**	sh3bp1	0.266310254	5.803799645	6.291229476	0.0149786	0.0328552
**ENSG00000182934**	SRPRA	0.170454334	6.802617535	6.290951915	0.0149807	0.0328552
**ENSG00000105612**	dnase2	0.44426958	3.819622611	6.286413541	0.0150152	0.0329240
**ENSG00000063176**	SPHK2	-0.50719945	4.018215422	6.276952142	0.0150875	0.0330755
**ENSG00000064763**	FAR2	0.260988169	5.341927904	6.276177145	0.0150934	0.0330775

**ENSG00000269028**	MTRNR2L2	0.880025442	6.979235917	6.507376216	0.0150947	0.0330775
**ENSG00000273189**		0.213363407	8.365852793	6.300658243	0.0151110	0.0331062
**ENSG00000143924**	EML4	-0.147722375	8.480436417	6.272768989	0.0151195	0.0331180
**ENSG00000008988**	RPS20	0.38442022	9.967616126	6.496385567	0.0151229	0.0331183
**ENSG00000136937**	NCBP1	-0.197138201	6.868808544	6.269930478	0.0151413	0.0331487
**ENSG00000258864**		0.35912042	4.691152829	6.269703542	0.0151431	0.0331487
**ENSG00000154174**	TOMM70	0.193895123	6.420772181	6.263079131	0.0151941	0.0332534
**ENSG00000136104**	RNASEH2B	0.241313792	6.405998278	6.255626701	0.0152517	0.0333725
**ENSG00000267303**		0.363267156	4.890930755	6.25110453	0.0152868	0.0334422
**ENSG00000040341**	STAU2	0.248694119	5.819340829	6.243711015	0.0153443	0.0335610
**ENSG00000124571**	XPO5	-0.272705457	5.950208786	6.242302588	0.0153553	0.0335780
**ENSG00000232472**		0.583103258	5.110704615	6.432384037	0.0153875	0.0336416
**ENSG00000103495**	MAZ	0.245224629	6.226793436	6.237578935	0.0153922	0.0336447
**ENSG00000006007**	GDE1	0.40883256	4.729198073	6.235569162	0.0154079	0.0336720
**ENSG00000245105**	A2M-AS1	0.541086977	4.636286274	6.325418224	0.0154132	0.0336765
**ENSG00000164118**	CEP44	-0.357602724	5.285957474	6.228502354	0.0154633	0.0337790
**ENSG00000156642**	NPTN	0.241586891	5.336350478	6.227380683	0.0154721	0.0337912
**ENSG00000261717**		0.496915368	3.668102224	6.225745354	0.0154850	0.0338122
**ENSG00000113282**	CLINT1	0.18694554	6.837158678	6.220408868	0.0155271	0.0338970
**ENSG00000100949**	RABGGTA	-0.438982607	4.399362002	6.219267394	0.0155361	0.0339096
**ENSG00000151623**	NR3C2	-0.227433971	6.257565267	6.216525739	0.0155577	0.0339478
**ENSG00000151292**	csnk1g3	0.310488078	6.513058342	6.313693607	0.0155601	0.0339478
**ENSG00000181800**	CELF2-AS1	-0.408539418	5.559406909	6.302018848	0.0155871	0.0339997
**ENSG00000114126**	TFDP2	-0.27030781	5.626951678	6.208561142	0.0156209	0.0340663
**ENSG00000156482**	RPL30	0.386740807	9.49878321	6.429091864	0.0156283	0.0340754
**ENSG00000116852**	KIF21B	0.187621652	7.406145169	6.201186929	0.0156796	0.0341800
**ENSG00000169032**	MAP2K1	0.247956066	5.992014325	6.200202162	0.0156874	0.0341900
**ENSG00000107949**	BCCIP	-0.306152216	5.209902205	6.195901919	0.0157218	0.0342577
**ENSG00000279277**		-0.504649386	3.974557857	6.191926807	0.0157536	0.0343199
**ENSG00000130559**	CAMSAP1	0.293051678	5.596218588	6.188781965	0.0157788	0.0343677
**ENSG00000129518**	EAPP	0.295830229	5.494168491	6.186612313	0.0157963	0.0343962
**ENSG00000147684**	NDUFB9	0.342052828	5.388809452	6.186337481	0.0157985	0.0343962
**ENSG00000230262**	MIRLET7DHG	-0.538296243	3.498818395	6.181696785	0.0158358	0.0344704
**ENSG00000026652**	AGPAT4	-0.429957819	4.298699837	6.179544424	0.0158532	0.0345010
**ENSG00000125826**	RBCK1	-0.324447176	4.977944778	6.179084273	0.0158569	0.0345019
**ENSG00000262477**		0.499436419	3.755111994	6.178673556	0.0158602	0.0345019
**ENSG00000131979**	GCH1	0.303810192	5.167410869	6.176286213	0.0158795	0.0345367

**ENSG00000148824**	MTG1	-0.462284455	4.420406463	6.1711571	0.0159210	0.0346198
**ENSG00000015532**	XYLT2	-0.336295126	4.754214489	6.165145801	0.0159698	0.0347187
**ENSG00000172164**	SNTB1	0.317926305	5.137720499	6.160165695	0.0160104	0.0347996
**ENSG00000210156**		-0.444546292	4.175090324	6.159327921	0.0160172	0.0348072
**ENSG00000177125**	zbtb34	0.37081384	4.735575396	6.158395558	0.0160248	0.0348165
**ENSG00000282804**		-0.296228078	5.222117121	6.152712729	0.0160713	0.0349102
**ENSG00000266967**	AARSD1	-0.504560001	3.830810791	6.148779893	0.0161035	0.0349678
**ENSG00000130725**	UBE2M	0.387614909	4.182738285	6.148661058	0.0161045	0.0349678
**ENSG00000101138**	CSTF1	0.253830722	5.755880859	6.147260333	0.0161160	0.0349791
**ENSG00000114383**	TUSC2	0.52705989	3.408195095	6.147212673	0.0161164	0.0349791
**ENSG00000116641**	DOCK7	0.375396815	5.007211208	6.128081153	0.0162743	0.0353145
**ENSG00000131475**	VPS25	-0.531138075	3.537332099	6.127299025	0.0162808	0.0353201
**ENSG00000163466**	ARPC2	0.157318131	8.172815037	6.126869896	0.0162843	0.0353201
**ENSG00000101109**	STK4	0.141036214	9.312459901	6.126548043	0.0162870	0.0353201
**ENSG00000171150**	SOCS5	0.310002061	5.041416934	6.124172815	0.0163068	0.0353556
**ENSG00000168672**	FAM84B	0.262837328	5.495054115	6.122744704	0.0163186	0.0353740
**ENSG00000111752**	PHC1	-0.237822684	5.870950091	6.118583771	0.0163533	0.0354418
**ENSG00000143183**	TMCO1	0.225256432	6.123140053	6.116928151	0.0163671	0.0354644
**ENSG00000183808**	RBM12B	0.18447261	6.675909537	6.115978724	0.0163750	0.0354742
**ENSG00000270629**	LOC100996763	-0.40219405	4.066808815	6.113160392	0.0163986	0.0355179
**ENSG00000103351**	CLUAP1	-0.243416727	6.034600224	6.110887924	0.0164176	0.0355517
**ENSG00000178950**	GAK	-0.301848444	6.365325046	6.167985298	0.0164387	0.0355901
**ENSG00000173681**	cxorf23	-0.337188633	5.234645513	6.105015786	0.0164669	0.0356436
**ENSG00000196743**	GM2A	0.423356488	4.273193554	6.100336073	0.0165062	0.0357214
**ENSG00000135048**	TMEM2	-0.21305103	7.180297273	6.094979548	0.0165514	0.0358118
**ENSG00000114062**	UBE3A	0.171288436	7.238579035	6.093147843	0.0165669	0.0358379
**ENSG00000104880**	ARHGEF18	-0.192917655	7.302487331	6.090337065	0.0165907	0.0358772
**ENSG00000164535**	DAGLB	-0.318678265	4.827055005	6.090187336	0.0165919	0.0358772
**ENSG00000166685**	COG1	-0.214254206	6.351796601	6.086943486	0.0166194	0.0359293
**ENSG00000147133**	TAF1	-0.183386947	7.382628169	6.084526814	0.0166400	0.0359662
**ENSG00000135972**	MRPS9	-0.377260456	4.42585318	6.08360315	0.0166478	0.0359757
**ENSG00000100722**	ZC3H14	-0.209920509	6.451993968	6.083130143	0.0166518	0.0359770
**ENSG00000268471**	MIR4453	-0.543518561	3.87182443	6.076791084	0.0167058	0.0360861
**ENSG00000133703**	kras	0.204044345	6.706626141	6.074953184	0.0167215	0.0361126
**ENSG00000101236**	RNF24	0.302540277	5.305321163	6.074382562	0.0167264	0.0361156
**ENSG00000068383**	INPP5A	-0.476075357	3.965364284	6.06993907	0.0167644	0.0361873
**ENSG00000088298**	EDEM2	-0.431795228	3.733783752	6.069693632	0.0167665	0.0361873

**ENSG00000141646**	SMAD4	0.143548866	7.671000292	6.069237546	0.0167704	0.0361882
**ENSG00000257411**		0.311835889	5.025471591	6.068143921	0.0167798	0.0361940
**ENSG00000164615**	CAMLG	0.376742751	4.880848817	6.068116616	0.0167800	0.0361940
**ENSG00000136827**	TOR1A	0.28993551	4.784318548	6.065242733	0.0168046	0.0362397
**ENSG00000132024**	cc2d1a	-0.557883349	3.755802394	6.062697667	0.0168265	0.0362794
**ENSG00000172500**	FIBP	0.486643413	4.002727433	6.061933037	0.0168331	0.0362861
**ENSG00000108828**	VAT1	0.39505502	4.3139388	6.058622803	0.0168616	0.0363400
**ENSG00000153975**	ZUFSP	-0.464390009	3.952431918	6.057218084	0.0168737	0.0363575
**ENSG00000186716**	BCR	0.224360079	6.283520315	6.056875168	0.0168766	0.0363575
**ENSG00000276182**		-0.543387532	3.89838066	6.05371502	0.0169039	0.0364064
**ENSG00000173465**	SSSCA1	0.368616239	4.647968255	6.053436465	0.0169063	0.0364064
**ENSG00000133193**	FAM104A	0.230789386	6.136726362	6.046283088	0.0169682	0.0365322
**ENSG00000100938**	GMPR2	-0.279644671	5.398263518	6.045356828	0.0169763	0.0365355
**ENSG00000196693**	ZNF33B	-0.24301585	6.337851264	6.045305439	0.0169767	0.0365355
**ENSG00000085224**	ATRX	0.175382741	8.772899843	6.04335511	0.0169937	0.0365644
**ENSG00000185813**	PCYT2	0.475922426	3.743329242	6.042337364	0.0170025	0.0365759
**ENSG00000139496**	NUP58	-0.233983084	6.255151844	6.041342383	0.0170111	0.0365870
**ENSG00000241014**	LOC653160	-0.52216088	4.092483899	6.038211773	0.0170384	0.0366380
**ENSG00000138434**	SSFA2	-0.278880802	5.450006332	6.036777098	0.0170509	0.0366574
**ENSG00000115998**	c2orf42	-0.33571879	4.803163199	6.033212458	0.0170820	0.0367167
**ENSG00000007545**	CRAMP1	-0.336559708	5.025384766	6.032425544	0.0170889	0.0367196
**ENSG00000252759**		-0.621029777	3.608980295	6.032253553	0.0170904	0.0367196
**ENSG00000151715**	TMEM45B	-0.563838469	3.595979748	6.030385489	0.0171067	0.0367472
**ENSG00000113966**	ARL 6.00	-0.44166831	3.54704554	6.023732786	0.0171650	0.0368648
**ENSG00000174106**	lemd3	0.183426284	6.453848434	6.022881591	0.0171725	0.0368680
**ENSG00000174514**	MFSD4A	-0.509687922	3.587892311	6.022763694	0.0171735	0.0368680
**ENSG00000146281**	PM20D2	-0.330182849	5.031486433	6.021156043	0.0171877	0.0368907
**ENSG00000273269**		0.325812009	5.222108498	6.019582705	0.0172015	0.0369084
**ENSG00000243107**		-0.569442506	3.782313468	6.018952843	0.0172070	0.0369084
**ENSG00000078177**	N4BP2	-0.219764103	6.885539172	6.018834289	0.0172081	0.0369084
**ENSG00000267046**		0.605857161	3.688197035	6.041322192	0.0172100	0.0369084
**ENSG00000260114**		-0.583383876	3.949269514	6.020975888	0.0172307	0.0369453
**ENSG00000229180**		-0.335603661	5.468043375	6.012817673	0.0172611	0.0369960
**ENSG00000163818**	LZTFL1	-0.421563918	4.09055346	6.012669833	0.0172624	0.0369960
**ENSG00000103966**	EHD4	0.322072359	4.766874195	6.012083019	0.0172676	0.0369960
**ENSG00000164190**	NIPBL	0.150508555	8.783346182	6.011979163	0.0172686	0.0369960
**ENSG00000149932**	TMEM219	0.383604095	4.547379735	6.010158899	0.0172846	0.0370229

**ENSG00000247228**	LOC400541	-0.303809108	5.038545121	6.00971205	0.0172886	0.0370238
**ENSG00000169299**	PGM2	0.315723894	4.823373156	6.006376703	0.0173181	0.0370794
**ENSG00000189077**	tmem120a	0.543614143	3.3452686	6.005834051	0.0173229	0.0370821
**ENSG00000078043**	PIAS2	-0.259943775	6.152402392	6.005119822	0.0173293	0.0370881
**ENSG00000153066**	TXNDC11	0.270119656	5.514015007	6.004286348	0.0173367	0.0370963
**ENSG00000090857**	PDPR	-0.203621106	6.391245326	6.002260714	0.0173547	0.0371272
**ENSG00000167986**	DDB1	0.170945708	6.953875026	6.000698859	0.0173685	0.0371453
**ENSG00000166477**	LEO1	0.260988242	5.547532469	6.000279082	0.0173723	0.0371453
**ENSG00000237399**	PITRM1-AS1	-0.395031896	4.651678401	6.000111078	0.0173738	0.0371453
**ENSG00000154059**	IMPACT	-0.424197018	4.319009876	5.995439541	0.0174154	0.0372266
**ENSG00000115514**	TXNDC9	0.314254632	4.775101182	5.993372547	0.0174338	0.0372584
**ENSG00000090372**	STRN4	-0.417883072	4.735653815	5.990960704	0.0174553	0.0372969
**ENSG00000281593**		0.454659127	3.748004759	5.98952543	0.0174682	0.0373167
**ENSG00000173530**	TNFRSF10D	-0.400649203	4.335273611	5.982543616	0.0175307	0.0374427
**ENSG00000207304**	SNORA8	0.836161178	5.071097221	6.192065905	0.0175394	0.0374534
**ENSG00000101421**	CHMP4B	0.292046272	5.7220101	5.981158021	0.0175432	0.0374540
**ENSG00000086061**	DNAJA1	0.199260143	7.053047227	5.980035712	0.0175533	0.0374654
**ENSG00000133740**	E2F5	-0.361180454	5.170856316	5.979764139	0.0175557	0.0374654
**ENSG00000145715**	RASA1	-0.19489939	7.327381832	5.97683296	0.0175821	0.0375140
**ENSG00000043143**	JADE2	0.170527219	7.495714468	5.976036683	0.0175893	0.0375217
**ENSG00000273373**		-0.694793837	3.573466181	6.022326609	0.0176058	0.0375492
**ENSG00000116830**	TTF2	-0.360566645	5.217639149	5.973786567	0.0176096	0.0375497
**ENSG00000148019**	CEP78	0.272108729	6.090347106	5.969833744	0.0176453	0.0376181
**ENSG00000134644**	PUM1	0.133656022	7.727036456	5.968812477	0.0176545	0.0376301
**ENSG00000177156**	TALDO1	0.325730727	5.214148432	5.966185588	0.0176783	0.0376731
**ENSG00000180884**	ZNF792	0.306629406	4.966072638	5.965167138	0.0176875	0.0376851
**ENSG00000186687**	lyrm7	-0.283170252	5.830696191	5.961702799	0.0177189	0.0377444
**ENSG00000227615**		0.636676888	5.371542473	6.165116684	0.0177229	0.0377452
**ENSG00000119979**	FAM45A	-0.297686446	4.778245734	5.959922893	0.0177351	0.0377635
**ENSG00000198015**	MRPL42	-0.200045791	6.678502911	5.958869558	0.0177447	0.0377762
**ENSG00000137502**	RAB30	-0.418733944	4.885434688	5.95727399	0.0178577	0.0380090
**ENSG00000009335**	UBE3C	0.19195963	6.705861725	5.94325693	0.0178873	0.0380643
**ENSG00000175104**	TRAF6	0.286195242	5.539427727	5.941169241	0.0179065	0.0380973
**ENSG00000167528**	ZNF641	-0.257708837	5.886383539	5.937928766	0.0179363	0.0381529
**ENSG00000106603**	coa1	-0.258508189	6.715189379	5.960902313	0.0179474	0.0381654
**ENSG00000234912**	Mir6516	-0.522656728	3.937574405	5.936495813	0.0179494	0.0381654
**ENSG00000258289**	CHURC1	-0.169821329	7.163516893	5.935311987	0.0179603	0.0381809

**ENSG00000130985**	UBA1	0.199377638	7.243773484	5.933718426	0.0179750	0.0382043
**ENSG00000113273**	ARSB	0.386916331	3.958317412	5.925497436	0.0180510	0.0383580
**ENSG00000263001**	GTF2I	-0.214523459	6.023775773	5.921165618	0.0180912	0.0384347
**ENSG00000130340**	SNX9	-0.299578897	5.674882679	5.920811469	0.0180945	0.0384347
**ENSG00000170043**	TRAPPC1	0.327287473	4.590027289	5.919415321	0.0181074	0.0384545
**ENSG00000268583**		-0.401483575	4.016274177	5.918792913	0.0181132	0.0384589
**ENSG00000163041**	H3F3A	-0.263607224	5.910912064	5.917146159	0.0181285	0.0384836
**ENSG00000083750**	RRAGB	-0.601175985	3.867641075	5.93988432	0.0181346	0.0384887
**ENSG00000158467**	AHCYL2	-0.328030554	4.770850338	5.913645376	0.0181611	0.0385372
**ENSG00000163913**	IFT122	-0.524062171	3.851169684	5.905990505	0.0182327	0.0386753
**ENSG00000109065**	NAT9	-0.422794638	4.280206621	5.9058886	0.0182336	0.0386753
**ENSG00000170606**	HSPA4	0.213302075	6.637057827	5.905106503	0.0182409	0.0386830
**ENSG00000254838**	GVINP1	-0.177205472	8.483908288	5.901400202	0.0182757	0.0387488
**ENSG00000115271**	GCA	-0.444877602	3.726732232	5.894037241	0.0183449	0.0388877
**ENSG00000210195**		-0.327862305	5.181232741	5.890142695	0.0183817	0.0389577
**ENSG00000284292**		0.208598984	6.806083659	5.87646241	0.0185113	0.0392246
**ENSG00000169249**	zrsr2	-0.31238157	5.477981374	5.865423252	0.0186167	0.0394398
**ENSG00000228794**	LINC01128	-0.318512964	4.925049464	5.862331929	0.0186463	0.0394945
**ENSG00000069020**	MAST4	0.254070068	6.259146966	5.857994584	0.0186879	0.0395747
**ENSG00000112651**	MRPL2	-0.559310531	3.315941037	5.857460784	0.0186931	0.0395776
**ENSG00000280498**	Snora16a	0.918107368	3.983208971	6.035295523	0.0187118	0.0396091
**ENSG00000164031**	DNAJB14	0.165814038	7.532827926	5.8470025	0.0187939	0.0397749
**ENSG00000063601**	MTMR1	0.213940659	6.366856388	5.844379853	0.0188193	0.0398166
**ENSG00000214485**		0.561285578	4.786441743	5.991391688	0.0188212	0.0398166
**ENSG00000171100**	MTM1	0.251024883	5.651760798	5.842734136	0.0188352	0.0398320
**ENSG00000172183**	ISG20	-0.214302059	6.443754276	5.842642885	0.0188361	0.0398320
**ENSG00000168906**	MAT2A	-0.16048088	7.192991615	5.840556426	0.0188563	0.0398667
**ENSG00000198455**	ZXDB	0.305344312	5.42969425	5.839227286	0.0188692	0.0398859
**ENSG00000151474**	FRMD4A	-0.462764583	4.333437745	5.837245581	0.0188885	0.0399186
**ENSG00000140326**	CDAN1	-0.386345218	4.241084628	5.829563871	0.0189633	0.0400657
**ENSG00000196418**	ZNF124	0.336003499	4.520778593	5.829311981	0.0189658	0.0400657
**ENSG00000111224**	PARP11	-0.256249217	5.81244743	5.824138542	0.0190163	0.0401644
**ENSG00000141404**	GNAL	-0.483231683	3.80892823	5.820377142	0.0190532	0.0402342
**ENSG00000265735**		0.881588614	6.016280887	6.020764186	0.0190617	0.0402440
**ENSG00000100503**	NIN	-0.195964705	8.481705625	5.820820531	0.0190887	0.0402928
**ENSG00000066084**	DIP2B	-0.232108336	6.827916353	5.816319255	0.0190930	0.0402939
**ENSG00000119640**	ACYP1	-0.530180243	3.814048404	5.813347995	0.0191223	0.0403475

**ENSG00000163428**	LRRC58	0.201318606	6.862172369	5.812631686	0.0191293	0.0403507
**ENSG00000162923**	WDR26	-0.148499635	7.868701416	5.812410628	0.0191315	0.0403507
**ENSG00000168397**	ATG4B	-0.371838019	4.963756957	5.811658391	0.0191389	0.0403582
**ENSG00000141905**	nfic	0.287064704	5.6839081	5.806721339	0.0191877	0.0404528
**ENSG00000075407**	ZNF37A	-0.228658453	6.085059981	5.804699716	0.0192077	0.0404868
**ENSG00000101166**	PRELID3B	0.264167469	5.957438253	5.801839462	0.0192360	0.0405383
**ENSG00000170471**	RALGAPB	0.182658161	7.536774224	5.800125814	0.0192530	0.0405659
**ENSG00000178449**	COX14	-0.454052641	3.581901747	5.798788931	0.0192662	0.0405857
**ENSG00000182923**	CEP63	-0.334402452	5.370423202	5.794551123	0.0193084	0.0406662
**ENSG00000274582**	Snora16a	0.914004701	3.986059928	5.970078634	0.0193175	0.0406772
**ENSG00000060642**	PIGV	0.587768305	3.615823286	5.801722278	0.0193857	0.0408127
**ENSG00000135407**	AVIL	-0.457693681	3.56511752	5.786183786	0.0193918	0.0408173
**ENSG00000214517**	PPME1	-0.366629843	4.426251506	5.785718516	0.0193965	0.0408189
**ENSG00000118873**	RAB3GAP2	0.184368344	6.826501374	5.783199714	0.0194217	0.0408598
**ENSG00000132664**	POLR3F	-0.351922397	4.100985336	5.782813637	0.0194255	0.0408598
**ENSG00000090061**	CCNK	-0.169134047	6.625518214	5.782416486	0.0194295	0.0408598
**ENSG00000169621**	PROKR1	-0.429678004	4.376141368	5.782215213	0.0194315	0.0408598
**ENSG00000270757**	HSPE1-MOB4	0.265485222	5.282233527	5.780135603	0.0194524	0.0408954
**ENSG00000240065**	PSMB9	-0.31978663	5.500525685	5.776934416	0.0194845	0.0409547
**ENSG00000184584**	TMEM173	0.245454787	6.323225066	5.776272994	0.0194911	0.0409605
**ENSG00000083937**	CHMP2B	0.289951997	5.473061028	5.771725391	0.0195369	0.0410484
**ENSG00000112877**	CEP72	-0.507218206	3.957390711	5.768667978	0.0195677	0.0411049
**ENSG00000267469**		0.203728355	7.164657865	5.767679285	0.0195777	0.0411176
**ENSG00000137207**	YIPF3	0.328085	4.611792431	5.766777166	0.0195868	0.0411285
**ENSG00000180257**	ZNF816	0.376262338	4.72880232	5.764787713	0.0196069	0.0411624
**ENSG00000198712**	COX2	0.262610729	11.99724036	5.834548002	0.0196561	0.0412555
**ENSG00000266469**		0.461131798	3.668266217	5.759633391	0.0196592	0.0412555
**ENSG00000149182**	ARFGAP2	-0.258267973	5.686283251	5.755674148	0.0196994	0.0413298
**ENSG00000114742**	WDR48	-0.2008471	6.304827506	5.755369127	0.0197025	0.0413298
**ENSG00000164347**	GFM2	-0.229546489	5.909592529	5.753331851	0.0197232	0.0413649
**ENSG00000105819**	PMPCB	-0.210922577	6.453711905	5.743120656	0.0198274	0.0415752
**ENSG00000229186**		-0.556810041	3.668365219	5.730217877	0.0199599	0.0418447
**ENSG00000260007**		-0.245896556	5.502464127	5.728509478	0.0199776	0.0418732
**ENSG00000145495**	38777	-0.12848996	8.414463499	5.726996356	0.0199932	0.0418976
**ENSG00000126001**	CEP250	-0.175762792	6.676206233	5.721996288	0.0200449	0.0419975
**ENSG00000113615**	sec24a	0.226406798	6.186435086	5.717815393	0.0200882	0.0420799
**ENSG00000112941**	PAPD7	-0.240719371	5.860972023	5.713550957	0.0201325	0.0421643

**ENSG00000160803**	UBQLN4	0.320731325	4.533988461	5.710255677	0.0201668	0.0422277
**ENSG00000146386**	ABRACL	0.355284565	5.326804508	5.727784405	0.0201775	0.0422414
**ENSG00000175970**	MIR4700	-0.378435711	4.447389627	5.699713792	0.0202770	0.0424414
**ENSG00000198931**	APRT	0.382211096	4.559161671	5.698531258	0.0202894	0.0424588
**ENSG00000182173**	TSEN54	-0.518902519	4.397531074	5.734959233	0.0203070	0.0424871
**ENSG00000119446**	RBM18	0.286143581	5.338106307	5.696217904	0.0203137	0.0424926
**ENSG00000182919**	C11orf54	-0.326859671	5.049922577	5.694818902	0.0203284	0.0425093
**ENSG00000259948**		0.484572533	3.518022449	5.694686926	0.0203298	0.0425093
**ENSG00000134108**	ARL8B	0.217857899	6.096745722	5.69247348	0.0203531	0.0425495
**ENSG00000165138**	ANKS6	-0.513122708	3.704479144	5.688661064	0.0203932	0.0426249
**ENSG00000167077**	MEI1	-0.382053774	4.408926842	5.677729412	0.0205089	0.0428580
**ENSG00000087266**	SH3BP2	0.285132073	5.998615827	5.675415622	0.0206985	0.0432431
**ENSG00000104412**	EMC2	-0.30028503	5.050156771	5.659318944	0.0207052	0.0432431
**ENSG00000167395**	ZNF646	0.256292133	5.274636781	5.659113413	0.0207074	0.0432431
**ENSG00000213676**	ATF6B	-0.284554009	5.82881933	5.658903644	0.0207097	0.0432431
**ENSG00000164164**	OTUD4	0.186642069	7.421387973	5.657478922	0.0207249	0.0432663
**ENSG00000205885**	C1RL-AS1	-0.463994974	4.397779008	5.655065582	0.0207509	0.0433118
**ENSG00000152684**	PELO	0.515365424	3.541615374	5.650648405	0.0207984	0.0434012
**ENSG00000114779**	ABHD14B	0.309734631	5.15868013	5.650311352	0.0208020	0.0434012
**ENSG00000095015**	MAP3K1	0.150876704	8.707111986	5.642520683	0.0208861	0.0435680
**ENSG00000106348**	IMPDH1	0.348992485	4.611857747	5.635629976	0.0209608	0.0437145
**ENSG00000113048**	MRPS27	0.22936293	5.552925855	5.635267643	0.0209647	0.0437145
**ENSG00000032219**	ARID4A	0.180474569	7.225242012	5.633849075	0.0209801	0.0437380
**ENSG00000197258**		0.421406763	4.364072072	5.631622809	0.0210044	0.0437797
**ENSG00000157890**	MEGF11	0.499238758	3.793122346	5.630199589	0.0210198	0.0438033
**ENSG00000184988**	TMEM106A	-0.463386769	3.844542321	5.629784602	0.0210244	0.0438040
**ENSG00000109103**	UNC119	-0.401947038	3.97233614	5.628357905	0.0210399	0.0438195
**ENSG00000175334**	BANF1	0.557313884	3.423245577	5.628329345	0.0210402	0.0438195
**ENSG00000264522**	OTUD7B	0.350385713	4.624877294	5.627333315	0.0210511	0.0438334
**ENSG00000273734**		-0.300967506	5.637663448	5.616529524	0.0211693	0.0440708
**ENSG00000170037**	CNTROB	-0.470236499	4.041423856	5.614507105	0.0211915	0.0441033
**ENSG00000197841**	ZNF181	-0.359993992	4.795364688	5.614341101	0.0211933	0.0441033
**ENSG00000172432**	GTPBP2	-0.286971311	5.083874375	5.613551305	0.0212020	0.0441126
**ENSG00000119285**	HEATR1	-0.1449259	7.577533894	5.610365771	0.0212371	0.0441767
**ENSG00000126756**	UXT	0.467003732	4.736088	5.67195606	0.0212588	0.0442131
**ENSG00000239883**		-0.403527489	4.544195134	5.605530406	0.0212904	0.0442700
**ENSG00000152348**	ATG10	0.358869155	8.931962264	5.787880299	0.0212980	0.0442770

**ENSG00000136206**	Spdye1	-0.537111348	3.465675647	5.603801243	0.0213095	0.0442921
**ENSG00000145860**	RNF145	0.19210049	6.520808909	5.602014735	0.0213292	0.0443244
**ENSG00000064933**	PMS1	-0.294228889	5.350931352	5.600855392	0.0213421	0.0443422
**ENSG00000106049**	HIBADH	-0.401446395	4.002940836	5.599917558	0.0213525	0.0443550
**ENSG00000183060**	LYSMD4	-0.404914995	4.383431594	5.596727806	0.0213878	0.0444196
**ENSG00000178229**	ZNF543	0.405686751	4.046946342	5.594364962	0.0214141	0.0444653
**ENSG00000221914**	PPP2R2A	0.204864811	6.312791776	5.586556949	0.0215010	0.0446369
**ENSG00000196466**	ZNF799	0.448332556	3.818296474	5.58330611	0.0215373	0.0447034
**ENSG00000138079**	SLC3A1	-0.302719837	4.844575387	5.582131569	0.0215504	0.0447218
**ENSG00000246084**	LOC101929241	-0.52583204	3.619279632	5.57584678	0.0216208	0.0448590
**ENSG00000101146**	RAE1	-0.34757224	4.700031077	5.571979241	0.0216643	0.0449403
**ENSG00000198393**	ZNF26	-0.245437978	6.004377527	5.570137911	0.0216850	0.0449743
**ENSG00000101442**	ACTR5	-0.507708537	3.85266877	5.565929535	0.0217325	0.0450638
**ENSG00000067225**	PKM	0.164115117	7.491403394	5.564911918	0.0217440	0.0450754
**ENSG00000100324**	TAB1	-0.417158478	4.008391792	5.56441747	0.0217495	0.0450754
**ENSG00000136932**	TRMO	-0.254608055	5.432526583	5.564286065	0.0217510	0.0450754
**ENSG00000160209**	LOC105372824	0.273341815	5.748890935	5.559421566	0.0218060	0.0451805
**ENSG00000152818**	UTRN	0.148833813	9.437276267	5.554852375	0.0218579	0.0452789
**ENSG00000005884**	ITGA3	0.597063453	3.430640745	5.553609357	0.0218720	0.0452992
**ENSG00000177409**	SAMD9L	-0.295334217	7.428684249	5.681606447	0.0219871	0.0455255
**ENSG00000111653**	ING4	-0.457008699	3.990337156	5.543256604	0.0219900	0.0455255
**ENSG00000164070**	HSPA4L	-0.650750936	3.52016674	5.561373152	0.0219972	0.0455256
**ENSG00000185885**	ifitm1	0.318975709	7.653370236	5.707293425	0.0219987	0.0455256
**ENSG00000268362**		0.378272751	4.550941086	5.53709665	0.0220605	0.0456444
**ENSG00000233360**	LOC101927051	0.294028943	4.808175978	5.536613978	0.0220660	0.0456468
**ENSG00000223496**	EXOSC6	-0.231535523	6.219204477	5.534968525	0.0220849	0.0456769
**ENSG00000257386**		-0.340284865	4.260721321	5.532926595	0.0221084	0.0457130
**ENSG00000071564**	TCF3	-0.338113283	5.109846221	5.532685802	0.0221111	0.0457130
**ENSG00000168280**	KIF5C	0.353327066	5.227012454	5.540996829	0.0221826	0.0458467
**ENSG00000280195**		0.246493913	8.08206312	5.626624662	0.0221846	0.0458467
**ENSG00000284697**		-0.442747555	3.653886049	5.525829482	0.0221901	0.0458491
**ENSG00000135632**	SMYD5	-0.460469761	3.703610252	5.523254395	0.0222198	0.0459014
**ENSG00000119004**	CYP20A1	0.230736382	5.586172306	5.522119114	0.0222330	0.0459195
**ENSG00000182774**	RPS17	0.278461489	9.201625465	5.660867079	0.0222882	0.0460245
**ENSG00000137513**	NARS2	0.400374859	3.959974551	5.512388648	0.0223458	0.0461343
**ENSG00000135457**	tfcp2	0.232463002	5.462003658	5.510517975	0.0223675	0.0461701
**ENSG00000149476**	TKFC	-0.435590808	4.106763001	5.508630001	0.0223895	0.0462064

**ENSG00000136522**	MRPL47	0.338101321	4.648676455	5.508021953	0.0223966	0.0462119
**ENSG00000105298**	CACTIN	-0.425294194	4.077371297	5.506610239	0.0224131	0.0462300
**ENSG00000173275**	ZNF449	-0.512164543	3.56943061	5.506508177	0.0224143	0.0462300
**ENSG00000114416**	FXR1	-0.141625972	7.635477627	5.501672173	0.0224707	0.0463374
**ENSG00000169131**	ZNF354A	-0.463261817	4.199353698	5.499803656	0.0224926	0.0463733
**ENSG00000073614**	KDM5A	0.159356538	8.093143664	5.498636681	0.0225063	0.0463835
**ENSG00000267419**		-0.398678183	3.951513472	5.498625961	0.0225064	0.0463835
**ENSG00000240571**		0.422467424	3.593000132	5.495255285	0.0225459	0.0464558
**ENSG00000145569**	FAM105A	0.288708183	5.194829495	5.494622593	0.0225534	0.0464619
**ENSG00000169905**	TOR1AIP2	0.171760774	6.836589959	5.490583782	0.0226008	0.0465506
**ENSG00000130332**	LSM 7.00	-0.372652334	4.627022654	5.489947632	0.0226083	0.0465568
**ENSG00000176396**	EID2	0.409921152	3.786079713	5.487945544	0.0226319	0.0465962
**ENSG00000267618**		0.319121422	4.942806115	5.487152943	0.0226412	0.0466062
**ENSG00000125459**	MSTO1	-0.468906136	3.732589118	5.486160964	0.0226529	0.0466212
**ENSG00000248871**	TNFSF12-TNFSF	0.519680616	3.468601083	5.484942388	0.0226673	0.0466416
**ENSG00000071553**	ATP6AP1	0.314234156	5.079014996	5.484005165	0.0226784	0.0466536
**ENSG00000258430**		0.398852501	3.818470529	5.483547527	0.0226838	0.0466536
**ENSG00000167522**	ANKRD11	-0.167198259	7.777888841	5.482777852	0.0226929	0.0466536
**ENSG00000159023**	EPB41	0.181403568	8.979420437	5.482746371	0.0226933	0.0466536
**ENSG00000170088**	TMEM192	0.358493884	4.653613487	5.482539417	0.0226957	0.0466536
**ENSG00000157036**	EXOG	-0.340780088	4.849428427	5.481842118	0.0227039	0.0466536
**ENSG00000105447**	GRWD1	0.401820501	3.853402164	5.481803151	0.0227044	0.0466536
**ENSG00000119403**	PHF19	-0.265791778	5.577657261	5.479694887	0.0227294	0.0466957
**ENSG00000272356**		0.438928895	3.851262274	5.474815209	0.0227872	0.0468054
**ENSG00000115966**	atf2	0.16195358	7.047124909	5.474186662	0.0227947	0.0468115
**ENSG00000110955**	ATP5B	0.165354744	7.016776353	5.472566317	0.0228139	0.0468418
**ENSG00000123992**	DNPEP	-0.368994223	4.679190014	5.465501565	0.0228981	0.0470053
**ENSG00000075790**	BCAP29	0.330438179	4.784445656	5.459744287	0.0229669	0.0471373
**ENSG00000172809**	RPL38	0.403117224	9.434506024	5.635254066	0.0229924	0.0471805
**ENSG00000260618**		-0.416167521	4.272475495	5.450487271	0.0230780	0.0473364
**ENSG00000154222**	CC2D1B	-0.226867745	5.917540293	5.450198655	0.0230814	0.0473364
**ENSG00000141956**	PRDM15	-0.37814047	4.742972448	5.450153913	0.0230820	0.0473364
**ENSG00000075292**	ZNF638	-0.164553901	8.172839617	5.439289277	0.0232131	0.0475961
**ENSG00000085788**	DDHD2	-0.187572892	6.306339662	5.437760417	0.0232317	0.0476247
**ENSG00000255835**		-0.425444754	3.907057951	5.436937817	0.0232416	0.0476358
**ENSG00000175130**	MARCKSL1	0.446944109	4.02327919	5.435679556	0.0232569	0.0476577
**ENSG00000270011**	ZNF559-ZNF177	-0.535413535	3.77826576	5.4328283	0.0232915	0.0477108

**ENSG00000255585**		-0.516247561	3.676259352	5.432765592	0.0232923	0.0477108
**ENSG00000116044**	NFE2L2	0.162573991	6.929206142	5.432067185	0.0233008	0.0477108
**ENSG00000179364**	PACS2	-0.302449404	5.023213148	5.432044303	0.0233010	0.0477108
**ENSG00000100784**	RPS6KA5	0.258728134	6.69445355	5.473106903	0.0233112	0.0477224
**ENSG00000118181**	RPS25	0.314777089	9.212585494	5.592321287	0.0233471	0.0477865
**ENSG00000142453**	CARM1	-0.379539655	4.46733239	5.426424534	0.0233695	0.0478173
**ENSG00000116704**	SLC35D1	-0.247856674	5.415495484	5.426270196	0.0233713	0.0478173
**ENSG00000105698**	USF2	-0.241885589	6.083975402	5.425751736	0.0233777	0.0478209
**ENSG00000122188**	LAX1	0.241161325	5.847830457	5.420298827	0.0234443	0.0479478
**ENSG00000174021**	GNG5	0.336355725	4.997923519	5.415183249	0.0235070	0.0480666
**ENSG00000259954**	IL21R-AS1	0.442820713	3.844336482	5.414239034	0.0235186	0.0480721
**ENSG00000263956**	NBPF11	-0.291951318	5.104224265	5.414214939	0.0235189	0.0480721
**ENSG00000147121**	KRBOX4	-0.419537298	4.037771638	5.407798223	0.0235978	0.0482240
**ENSG00000213300**		0.425721619	4.036955453	5.404179864	0.0236424	0.0483058
**ENSG00000270149**		0.39195396	3.992969368	5.394577792	0.0237613	0.0485375
**ENSG00000211795**		0.491998159	3.941568978	5.394271382	0.0237651	0.0485375
**ENSG00000100353**	EIF3D	0.164819104	7.2873939	5.393533082	0.0237743	0.0485467
**ENSG00000248751**		-0.452884589	3.967814218	5.391261978	0.0238025	0.0485949
**ENSG00000088854**	c20orf194	0.43867568	4.228226014	5.390003953	0.0238182	0.0486173
**ENSG00000049239**	H6PD	0.25865119	5.851678583	5.388523702	0.0238366	0.0486455
**ENSG00000111850**	SMIM8	-0.420312338	3.995412881	5.387987495	0.0238433	0.0486496
**ENSG00000136436**	CALCOCO2	-0.17974483	6.61314001	5.387250909	0.0238524	0.0486588
**ENSG00000047621**	c12orf4	-0.366001588	4.755951537	5.386303298	0.0238643	0.0486734
**ENSG00000128272**	ATF4	-0.222498503	6.177238421	5.381676644	0.0239220	0.0487818
**ENSG00000160408**	ST6GALNAC6	-0.349172496	5.498804639	5.411135435	0.0239453	0.0488196
**ENSG00000103415**	HMOX2	0.262264444	5.126008105	5.377607966	0.0239730	0.0488666
**ENSG00000210082**		0.483388397	15.31550363	5.544150073	0.0239879	0.0488875
**ENSG00000169221**	TBC1D10B	0.306613143	4.521729496	5.370604763	0.0240609	0.0490267
**ENSG00000108666**	c17orf75	-0.433976445	3.933803928	5.369830956	0.0240706	0.0490370
**ENSG00000051596**	THOC3	-0.424354092	3.828677949	5.366937488	0.0241071	0.0491017
**ENSG00000197128**	ZNF772	-0.510956553	3.660192451	5.364775661	0.0241343	0.0491476
**ENSG00000001460**	stpg1	-0.461057874	3.474917701	5.364161125	0.0241421	0.0491530
**ENSG00000162585**	FAAP20	-0.405613689	4.208981581	5.363822267	0.0241464	0.0491530
**ENSG00000105778**	AVL9	0.194005592	6.500912518	5.362175707	0.0241672	0.0491858
**ENSG00000261468**		-0.45020639	4.28024244	5.361761921	0.0241724	0.0491868
**ENSG00000119778**	ATAD2B	-0.207532757	6.714742008	5.361021766	0.0241818	0.0491963
**ENSG00000262302**		0.407914521	4.398194238	5.358874693	0.0242089	0.0492420

**ENSG00000109445**	ZNF330	-0.285813372	5.150106033	5.356863655	0.0242344	0.0492833
**ENSG00000188603**	CLN3	-0.272474042	5.305842145	5.356528048	0.0242387	0.0492833
**ENSG00000173588**	cep83	-0.320429307	4.969655236	5.351496782	0.0243026	0.0493930
**ENSG00000151240**	DIP2C	-0.401140959	4.21109213	5.351203911	0.0243063	0.0493930
**ENSG00000215251**	FASTKD5	0.278662744	4.953006439	5.351165832	0.0243068	0.0493930
**ENSG00000099810**	MTAP	0.238963362	5.941386528	5.348390381	0.0243421	0.0494551
**ENSG00000226979**	LTA	0.479704299	3.565463048	5.346710629	0.0243635	0.0494890
**ENSG00000078070**	MCCC1	-0.353408977	4.674305824	5.34614428	0.0243707	0.0494941
**ENSG00000138641**	LOC101929134	-0.153518593	7.213469649	5.332887616	0.0245404	0.0498124
**ENSG00000047056**	WDR37	-0.225172397	6.05020586	5.332847849	0.0245409	0.0498124
**ENSG00000185721**	DRG1	-0.260307864	5.363341566	5.332783965	0.0245418	0.0498124
**ENSG00000183091**	NEB	-0.54009615	4.46898493	5.397309457	0.0245867	0.0498940
**ENSG00000131378**	RFTN1	-0.20679075	6.822484287	5.328606786	0.0245955	0.0498993
**ENSG00000105193**	RPS16	0.340268769	9.337647753	5.493327833	0.0245989	0.0498993
**ENSG00000131507**	NDFIP1	-0.184041841	7.211214645	5.327054681	0.0246155	0.0499233
