# Supplementary material for: CSF-resident CD4+ T-cells display a distinct gene expression profile with relevance to immune surveillance and multiple sclerosis
Source: Brain Commun. 2021 Jul 13;3(3):fcab155. doi: 10.1093/braincomms/fcab155 (PMC8574295; doi:10.1093/braincomms/fcab155)
Supplement: fcab155_Supplementary_Data [file fcab155_Supplementary_Data.zip › Supplementary_Material.pdf]

Supplementary material

Diagnosis	Number	Diagnostic group	Number
Multiple sclerosis	41	Multiple sclerosis	41
Cerebellar degeneration	1	Non-inflammatory disorder (NID) controls	38
Disequilibrium	1		
Fibromyalgia	1		
Functional disorder	5		
Idiopathic intracranial hypertension	16		
Inclusion body myositis	1		
Ischaemic myelopathy	2		
Leukodystrophy	1		
Migraine	3		
Peripheral neuropathy	2		
Cerebral small vessel disease	5		

Supplementary table 1. Table showing the eventual diagnoses of patients included in the analyses.

Characteristic	Multiple sclerosis	Non-inflammatory controls	P-value for significant difference
N	41	38	N/A
Number female (%)	26 (63.4%)	33 (86.8)	0.05[b]
Age at sampling[a]	44.0 (13.1)	40.3 (15.3)	0.26[c]
Ethnicity (% Caucasian)	95%	97%	N/A

Supplementary table 2. Demographic characteristics of patients with multiple sclerosis and non-inflammatory controls that were included in analyses. [a]Data are expressed as mean (standard deviation); [b]P-value from Chi-squared test; [c]P-value from t-test.

**Characteristic in multiple sclerosis patients**	
Relapsing-onset (%)	90.3
Time since symptom onset (months)[a]	69.2 (115.3)
EDSS at sampling[b]	3.0 (0-6.5)

Supplementary table 3. Disease-specific characteristics of patients with multiple sclerosis that underwent RNA sequencing. [a]Data are expressed as mean (standard deviation); [b]Data are expressed as median (range).

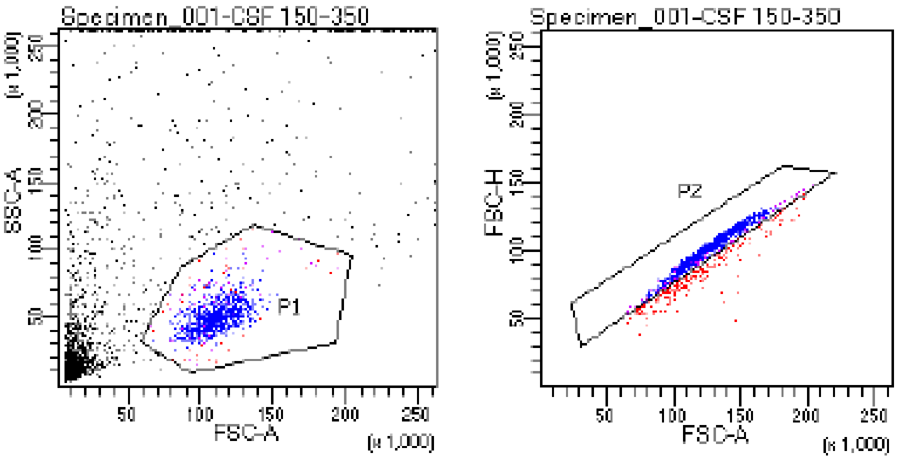

Supplementary figure 1. Example plot of the first (left) and second (right) FACS gates. The first gate (P1) selects cells based on size (forward scatter, x-axis, FSC-A) and granularity (side scatter, y-axis, SSC-A). The second gate (P2) excludes doublets (two cells stuck together). The blue dots are lymphocytes and the black and red dots are other cell types and debris.

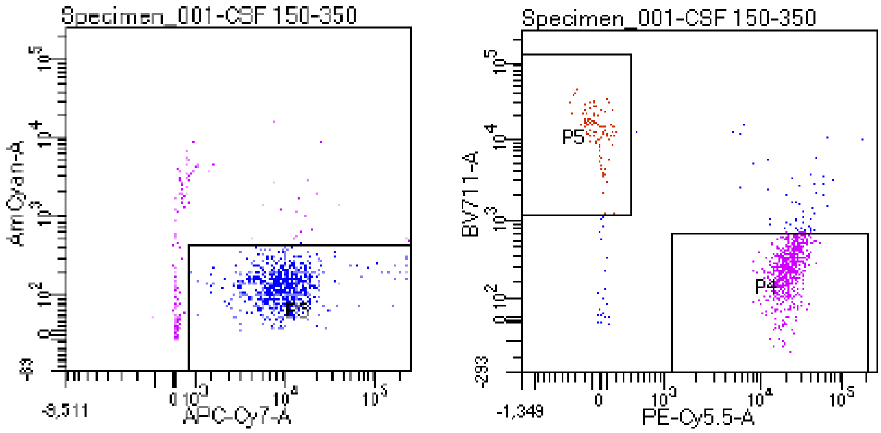

Supplementary figure 2. Example plots of the FACS gates used to select cells based on fluorescence. The plot on the fleft shows gate P3, which selects cells (blue dots) that are APC-H7 positive (x-axis, labelled APC-Cy7-A), and negative for LIVE/DEAD® and V500 (y-axis, labelled AmCyan-A). The plot on the right shows gates P4 and P5, which select cells that are PE-Cy5.5 (purple dots, $CD4^+$ T-cells) or BV711 (orange dots, $CD8^+$ T-cells) positive, respectively. Double positives and double negatives are excluded (blue dots).

Fluorescently labelled antibody/stain	Manufacturer and local supply origin	Laser, peak emission	Function
LIVE/DEAD® ViViD aqua	Invitrogen/ThermoFisher, Paisley, UK.	Blue, 526nm	Exclusion of dead cells
Anti-CD14 V500	BD Biosciences, Oxford, UK.	Violet, 500nm	Exclusion of monocytes, macrophages, neutrophils and dendritic cells
Anti-CD19 V500	BD Biosciences, Oxford, UK.	Violet, 500nm	Exclusion of B-cells
Anti-CD3 APC-H7	BD Biosciences, Oxford, UK.	Red, 767nm	Selection of T-cells
Anti-CD4 PE-Cy5.5	ThermoFisher Scientific, Paisley, UK.	Blue, 694nm	Selection of $CD4^+$ T-cells
Anti-CD8 BV711	Biolegend, London, UK.	Blue, 711nm	Selection of $CD8^+$ T-cells

Supplementary table 4. Fluorescently labelled antibodies used in the protocol to isolate purified cell populations.

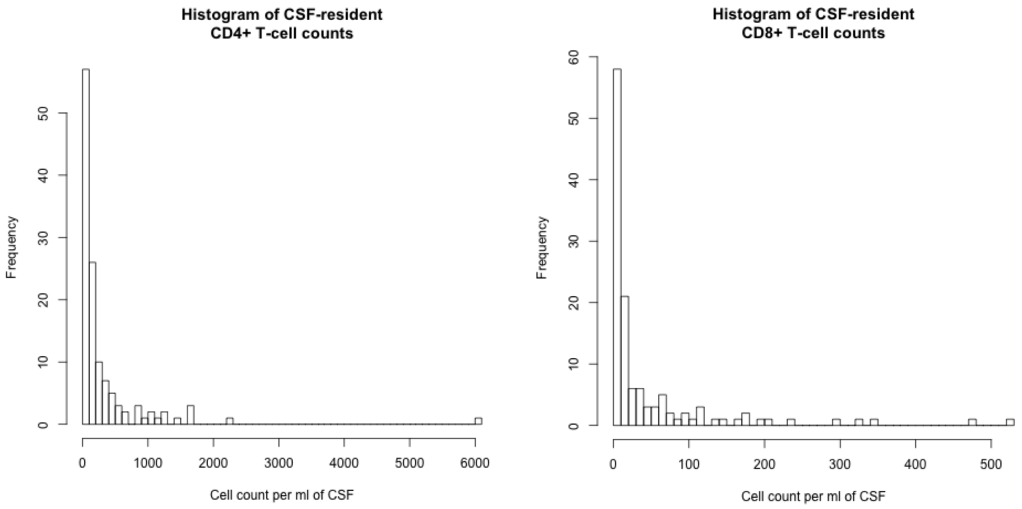

Supplementary figure 3. Histograms showing the distribution of CSF-resident $CD4^+$ (left) and $CD8^+$ (right) T-cell counts from FACS.

Median total number of reads (range)	Median percentage mapped reads (range)	Median percentage duplicates (range)
157417402 (62518178-254517988)	77.3 (7.9-94.2)	73.5 (7.4-87.2)

Supplementary table 5. Mapping statistics for all CSF samples.

Cell source	Median total reads in NIC	Median total reads in MS	Median % mapped reads in NIC	Median % mapped reads in MS	Median % duplicates in NIC	Median % duplicates in MS
CSF	140862002 (63674198-202272960)	145628814 (62518178-185119856)	80.7 (36.4-89.8)	88.5 (77.5-94.2)	76.4 (34.1-86.2)	82.3 (73.3-86.4)
Blood	153714206 (37549362-194255940)	155265094 (94477568-193171382)	90.5 (59.8-94.6)	89.9 (76.8-94.3)	81.8 (34.1-87.6)	82.1 (68.3-86.6)

Supplementary table 6. Mapping statistics for the 41 patients' samples used in differential expression analysis.

ID	Tissue	Sex	Age at sampling	Diagnostic group	Batch	RIN	Mapping %
78012	Blood	Female	22.1	NID	9	7.8	59.8
77314	CSF	Female	23.2	NID	11	4.2	53.2

Supplementary table 7. Excluded outliers in the CSF vs. blood $CD4^+$ T-cells analysis; leaving 21 patients with multiple sclerosis and 20 non-inflammatory disorders (NID) controls.

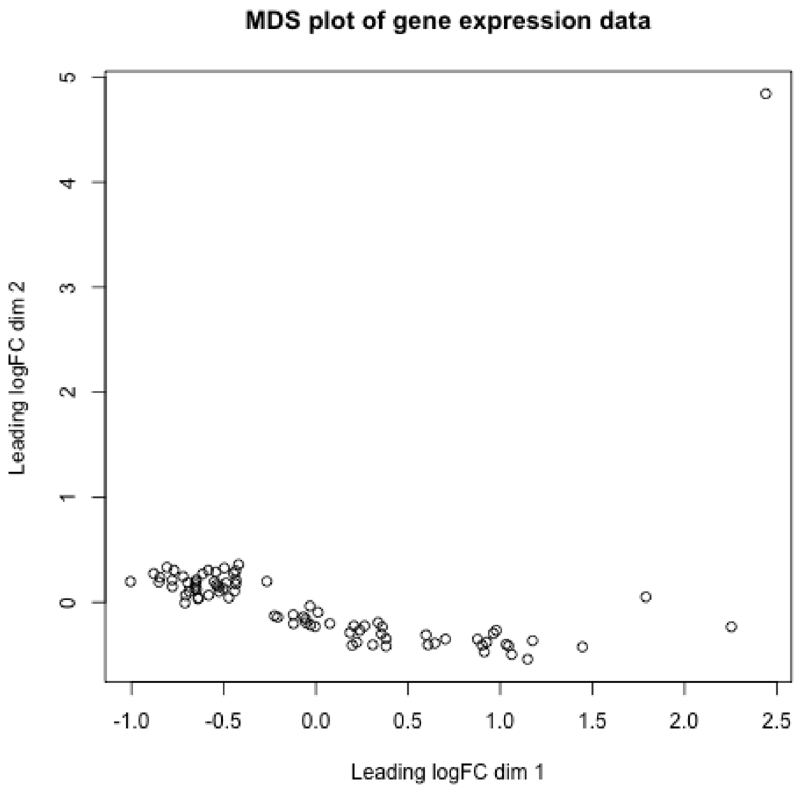

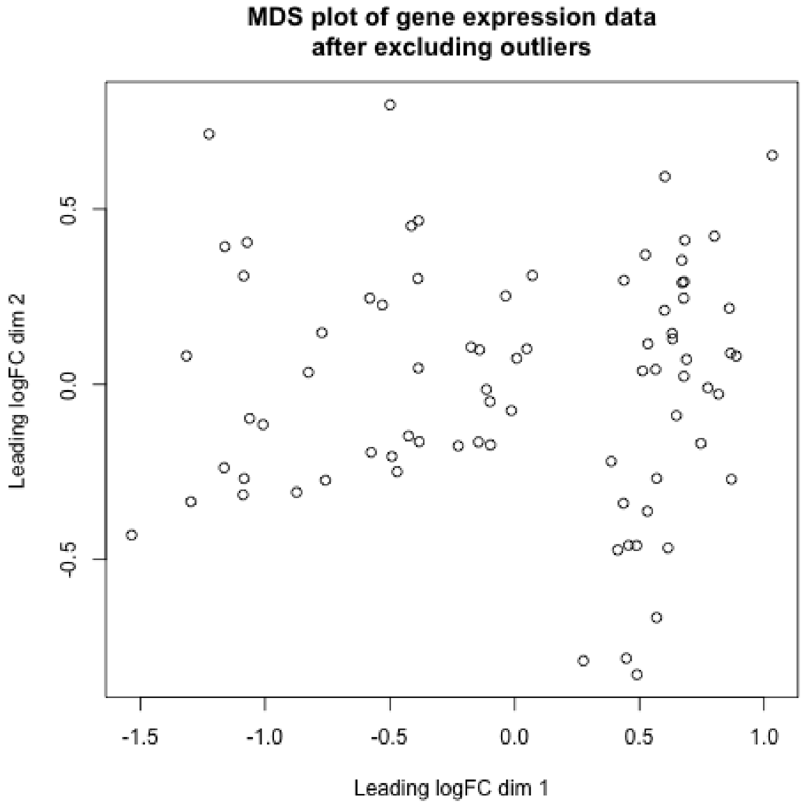

Supplementary figure 4. Multi-dimensional scaling (MDS) plots of CSF and blood $CD4^+$ T-cell gene expression data: Before exclusions (top) and after exclusion of outliers (bottom).

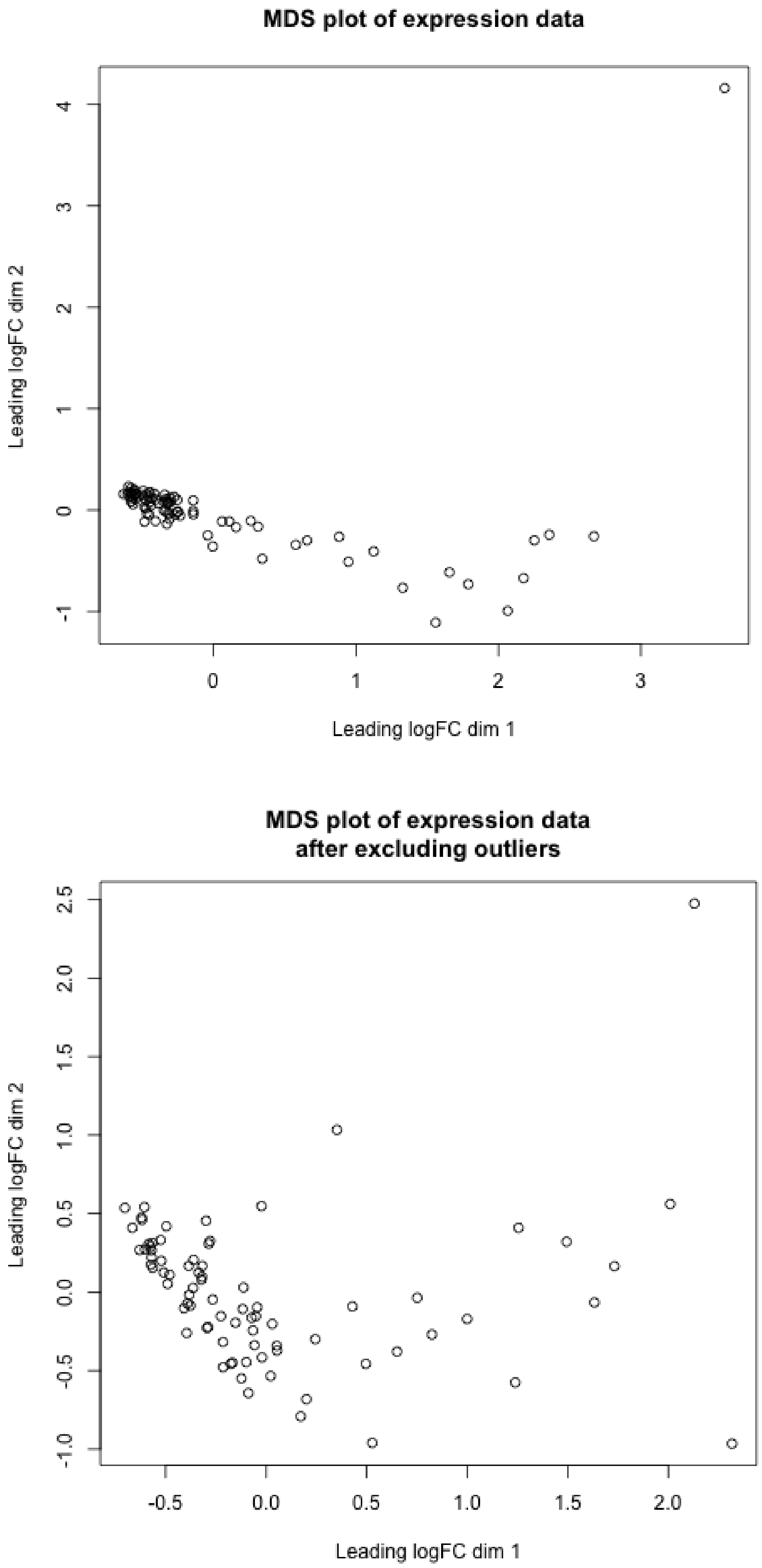

Supplementary figure 5. Multi-dimensional scaling (MDS) plots of CSF CD4$^+$ T-cell gene expression data: Before exclusions (top) and after exclusion of outliers (bottom).

ID	Tissue	Sex	Age at sampling	Diagnostic group	Batch	RIN	Mapping %
66834	CSF	Male	66.8	MS	18	1.2	7.9
13368	CSF	Male	48.7	MS	19	0	70.2
70821	CSF	Female	44.0	NID	14	5.7	55.2
39093	CSF	Female	56.9	NID	19	5.5	82.4
27461	CSF	Female	30.5	NID	16	1.1	27.1
48544	CSF	Female	40.3	NID	17	1.0	45.9
78273	CSF	Female	55.2	NID	18	1.0	57.1

Supplementary table 8. Excluded outliers in the analysis of CSF $CD4^+$ T-cells in patients with multiple sclerosis (MS) compared with non-inflammatory controls; leaving 41 patients with multiple sclerosis and 38 non-inflammatory disorder (NID) controls.

Gene	Log$_2$ fold-change NIC	FDR in NIC	Log$_2$ fold-change MS	FDR in MS	Known immunological functions
CCR7	-1.3	1.94×10^{-7}	-1.2	2.86×10^{-6}	Associated with central memory $CD4^+$ T-cells (Kivisakk *et al*., 2004; Mullen *et al*., 2012).
CCR4	0.9	0.005	1.0	0.0002	Receptor for CCL2, CCL4, CCL17, CCL22 and RANTES (chemokine elevated in MS relapse CSF and inflamed tissue); required for T regulatory cell (T_{reg}) migration into nonlymphoid tissues; associated with Th2 phenotype (Sather *et al*., 2007).
CCR2	2.4	2.54×10^{-11}	2.7	2.85×10^{-10}	Receptor for CCL2; high expression on activated memory T-cells and T-cells in MS lesions (Kivisakk *et al*., 2002; Murphy *et al*., 2000; Simpson *et al*., 2000).
CCR5	2.9	3.06×10^{-15}	2.7	1.07×10^{-15}	Receptor for CCL3, CCL3L1, CCL4 and RANTES; elevated in MS relapse CSF and inflamed tissue; associated with Th1 phenotype; high expression on T-cells in MS lesions and in perivascular cuff (Kivisakk *et al*., 2002; Herich *et al*., 2019; Qin *et al*., 1998; Norri *et al*., 2006; Balashov *et al*., 2009).
CCR6	1.1	0.002	NA	NA	Receptor for CCL20; associated with Th1 phenotype (Kivisakk *et al*., 2002).
ITGA4	0.7	0.002	0.8	8.35×10^{-5}	Lymphocyte homing receptor for CNS (Holzmann *et al*., 1989; Takada *et al*., 1989).
ITGB1	1.2	3.73×10^{-7}	0.9	0.002	Lymphocyte homing receptor for CNS (Holzmann *et al*., 1989; Takada *et al*., 1989).
ITGB2-AS1	-1.6	0.0003	-1.4	0.001	Antisense to ITGB2, which forms LFA-1 with integrin-□X; LFA-1 signalling involved in migration, activation and differentiation (Verma *et al*., 2017).
CXCR6	2.0	2.36×10^{-8}	1.7	8.34×10^{-8}	Receptor for CCL16: chemotaxis, adhesion, scavenging LDL. Homing marker upregulated in $CD8^+$ T-cells in MS lesions (Shimaoka *et al*., 2000; Shimaoka *et al*., 2004; Fransen *et al*., 2020).
CXCR3	2.6	3.24×10^{-12}	2.4	3.69×10^{-12}	Chemokine receptor for IP-10 and Mig (elevated in MS relapse CSF and inflamed tissue), associated with Th1 phenotype (Kivisakk *et al*., 2002; Sorensen *et al*., 1999; Qin *et al*., 1998; Balashov *et al*., 2009).

CD2 (LFA-2)	0.8	2.97 x 10^{-5}	0.8	0.001	Adhesion and co-stimulatory signalling molecule on surface of T-cells; interacts with CD58 during T-cell activation (MS risk locus); enhances T_{reg} activity (De Jager *et al*., 2009).
CD99	0.9	6.74 x 10^{-5}	0.8	0.008	T-cell adhesion and apoptosis of double positive T-cells; co-stimulatory signal that leads to recruitment of TCR and CD3 into lipid raft and increases stimulatory signal from TCR-peptide-MHC interaction only when a suboptimal level of signal is provided by TCR-peptide-MHC interaction alone (Oh *et al*., 2007).
CD38	-1.9	8.59 x 10^{-6}	-1.2	0.01	Multifunctional enzyme essential for regulation of intracellular calcium and marker of T-cell activation (Malavasi *et al*., 2008).
CD81	1.0	0.0002	0.8	0.02	Co-stimulatory molecule (Levy *et al*., 1998).
LFA-3 (CD58)	NA	NA	0.9	0.04	Adhesion molecule expressed on APCs that strengthens adhesion to T-cell (Selvaraj *et al*., 1987).
GZMK	2.9	1.11 x 10^{-16}	2.9	1.93 x 10^{-17}	Stored in granules in T-cells and released at the onset of extravasation to stimulate endothelial ICAM-1 expression, which stimulates transcellular diapedesis (Herich *et al*., 2019).

Supplementary table 11. Molecules involved in cellular migration that were found to be significantly differentially expressed between CSF and blood $CD4^+$ T-cells in non-inflammatory controls and/or multiple sclerosis; NIC = non-inflammatory controls, MS = multiple sclerosis, FDR = false discovery rate.

GO term	Fold enrichment	FDR
Movement of cell or subcellular component	1.23	2.5×10^{-6}
Locomotion	1.23	4.6×10^{-5}
Biological adhesion	1.20	4.6×10^{-5}
Cell adhesion	1.20	4.6×10^{-5}
Localisation of cell	1.23	2.6×10^{-4}
Cell motility	1.23	2.6×10^{-4}
Cell migration	1.23	2.6×10^{-4}
Actin filament-based process	1.30	2.6×10^{-4}
Cell-cell adhesion	1.20	7.7×10^{-4}
Actin filament organisation	1.38	9.3×10^{-4}

Supplementary table 12. Ten most significant GO terms for the 5,156 genes significantly differentially expressed between CSF $CD4^+$ T-cells and blood $CD4^+$ T-cells in non-inflammatory controls.

GO term	Fold enrichment	FDR
Movement of cell or subcellular component	1.32	2.7×10^{-10}
Single organismal cell-cell adhesion	1.43	4.4×10^{-8}
Cell migration	1.38	1.9×10^{-7}
Cell activation	1.36	2.0×10^{-7}
Locomotion	1.31	2.1×10^{-7}
Single organism cell adhesion	1.39	4.4×10^{-7}
Immune response	1.30	6.7×10^{-7}
Localisation of a cell	1.31	1.4×10^{-6}
Cell motility	1.31	1.4×10^{-6}
Biological adhesion	1.25	4.5×10^{-6}

Supplementary table 13. Ten most significant GO terms for the 4,263 genes significantly differentially expressed between CSF $CD4^+$ T-cells and blood $CD4^+$ T-cells in multiple sclerosis patients.

GO term	Fold enrichment	FDR
Rho protein signal transduction	1.65	0.021
Tissue homeostasis	1.43	0.046
Enzyme linked receptor protein signalling pathway	1.17	0.048

Supplementary table 14. Gene ontology terms significant only in non-inflammatory disorder controls.

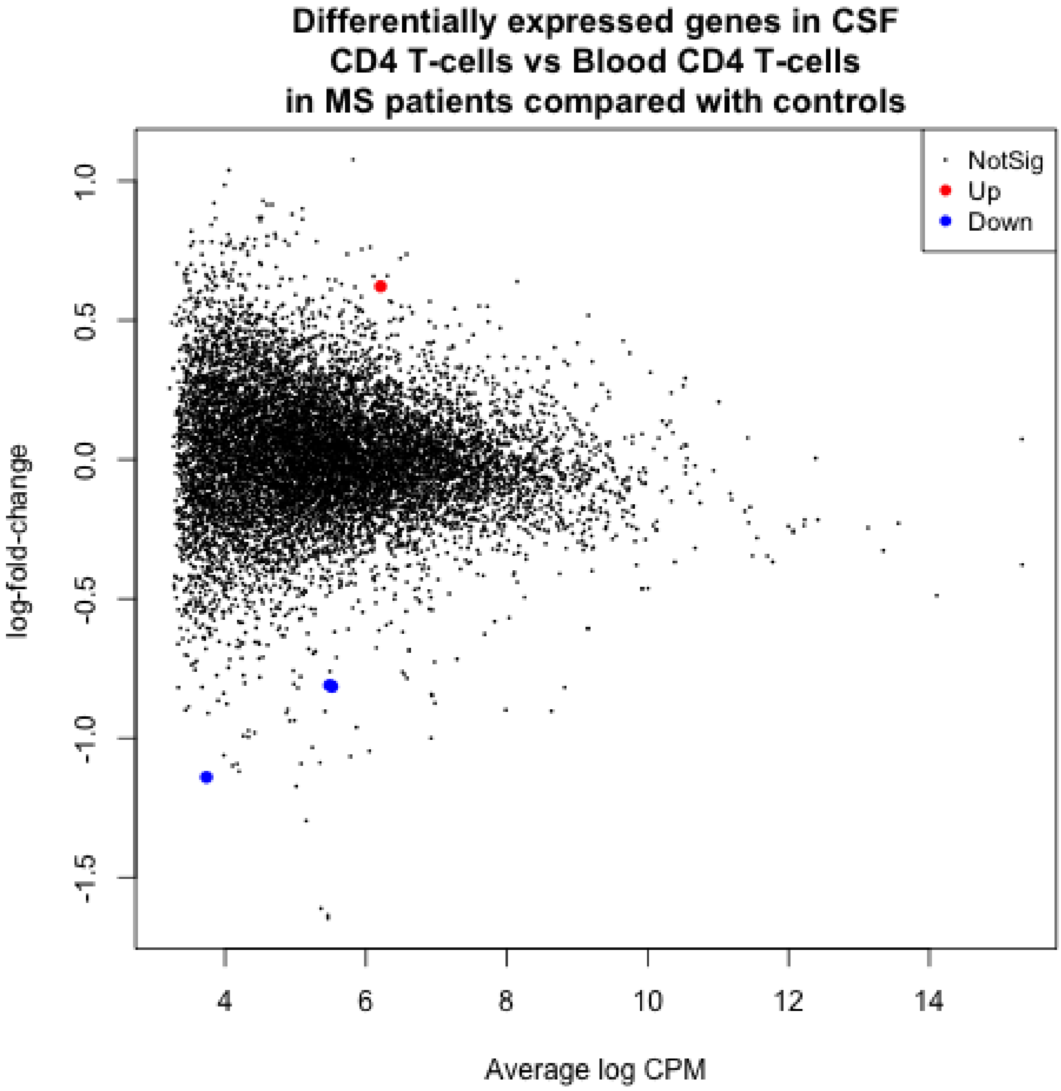

Supplementary figure 6. Mean-difference plot of genes differentially expressed in CSF CD4$^+$ T-cells vs. blood CD4$^+$ T-cells in patients with multiple sclerosis compared with non-inflammatory controls); CPM = count per million bp (gene size), DE = differentially expressed.

GO term	Fold enrichment	FDR
Purine ribonucleotide metabolic process	5.82	9.04×10^{-5}
Ribonucleotide metabolic process	5.62	1.46×10^{-4}
Purine nucleotide metabolic process	5.52	1.84×10^{-4}
ATP metabolic process	7.35	2.16×10^{-4}
Ribose phosphate metabolic process	5.43	2.31×10^{-4}
Purine-containing compound metabolic process	5.23	3.78×10^{-4}
Purine ribonucleoside triphosphate metabolic process	6.91	4.27×10^{-4}
Ribonucleoside triphosphate metabolic process	6.82	4.94×10^{-4}
Purine nucleoside triphosphate metabolic process	6.78	5.30×10^{-4}
Nucleoside triphosphate metabolic process	6.47	8.64×10^{-4}

Supplementary table 17. Ten most significant GO terms for the 136 genes significantly differentially expressed between multiple sclerosis CSF $CD4^+$ T-cells and non-inflammatory control CSF $CD4^+$ T-cells.
